# Hospital-treated infectious diseases and the risk of dementia: a large, multicohort, observational study with a replication cohort

**DOI:** 10.1016/S1473-3099(21)00144-4

**Published:** 2021-11

**Authors:** Pyry N Sipilä, Nelli Heikkilä, Joni V Lindbohm, Christian Hakulinen, Jussi Vahtera, Marko Elovainio, Sakari Suominen, Ari Väänänen, Aki Koskinen, Solja T Nyberg, Jaana Pentti, Timo E Strandberg, Mika Kivimäki

**Affiliations:** a Clinicum, Department of Public Health, University of Helsinki, Helsinki, Finland; b Helsinki Institute of Life Science, University of Helsinki, Helsinki, Finland; c Medicum, Department of Bacteriology and Immunology, University of Helsinki, Helsinki, Finland; d Translational Immunology Research Program, University of Helsinki, Helsinki, Finland; e Department of Psychology and Logopedics, University of Helsinki, Helsinki, Finland; f Research Programs Unit, University of Helsinki, Helsinki, Finland; g Finnish Institute of Occupational Health, Helsinki, Finland; h Department of Epidemiology and Public Health, University College London, London, UK; i Finnish Institute for Health and Welfare, Helsinki, Finland; j Department of Public Health, University of Turku, Turku, Finland; k Centre for Population Health Research, University of Turku and Turku University Hospital, Turku, Finland; l Research Services, Turku University Hospital, Turku, Finland; m School of Health Sciences, University of Skövde, Skövde, Sweden; n Department of Medicine, Helsinki University Hospital, Helsinki, Finland; o Center for Life Course Health Research, University of Oulu, Oulu, Finland

## Abstract

**Background:**

Infections have been hypothesised to increase the risk of dementia. Existing studies have included a narrow range of infectious diseases, relied on short follow-up periods, and provided little evidence for whether the increased risk is limited to specific dementia subtypes or attributable to specific microbes rather than infection burden. We aimed to compare the risk of Alzheimer's disease and other dementias across a wide range of hospital-treated bacterial and viral infections in two large cohorts with long follow-up periods.

**Methods:**

In this large, multicohort, observational study, the analysis was based on a primary cohort consisting of pooled individual-level data from three prospective cohort studies in Finland (the Finnish Public Sector study, the Health and Social Support study, and the Still Working study) and an independent replication cohort from the UK Biobank. Community-dwelling adults (≥18 years) with no dementia at study entry were included. Follow-up was until Dec 31, 2012, in the Health and Social Support study, Dec 31, 2016, in the public sector study and the Still Working study, and Feb 7, 2018, in the replication cohort. Through record linkage to national hospital inpatient registers, we ascertained exposure to 925 infectious diseases (using the International Classification of Diseases 10th Revision codes) before dementia onset, and identified incident dementia from hospital records, medication reimbursement entitlements, and death certificates. Hazard ratios (HRs) for the associations of each infectious disease or disease group (index infection) with incident dementia were assessed by use of Cox proportional hazards models. We then repeated the analysis after excluding incident dementia cases that occurred during the first 10 years after initial hospitalisation due to the index infection.

**Findings:**

From March 1, 1986, to Jan 1, 2005, 260 490 people were included in the primary cohort, and from Dec 19, 2006, to Oct 1, 2010, 485 708 people were included in the replication cohort. In the primary cohort analysis based on 3 947 046 person-years at risk (median follow-up 15·4 years [IQR 9·8–21·0]), 77 108 participants had at least one hospital-treated infection before dementia onset and 2768 developed dementia. Hospitalisation for any infectious disease was associated with increased dementia risk in the primary cohort (adjusted HR [aHR] 1·48 [95% CI 1·37–1·60]) and replication cohort (2·60 [2·38-2·83]). The association remained when analyses were restricted to new dementia cases that occurred more than 10 years after infection (aHR 1·22 [95% CI 1·09–1·36] in the primary cohort, the replication cohort had insufficient follow-up data for this analysis), and when comorbidities and other dementia risk factors were considered. There was evidence of a dose-response association between the number of episodes of hospital-treated infections and dementia risk in both cohorts (p_trend_=0·0007). Although the greatest dementia risk was seen for central nervous system (CNS) infections versus no infection (aHR 3·01 [95% CI 2·07–4·37]), excess risk was also evident for extra-CNS infections (1·47 [1·36–1·59]). Although we found little difference in the infection-dementia association by type of infection, associations were stronger for vascular dementia than for Alzheimer's disease (aHR 2·09 [95% CI 1·59–2·75] versus aHR 1·20 [1·08–1·33] in the primary cohort and aHR 3·28 [2·65–4·04] versus aHR 1·80 [1·53–2·13] in the replication cohort).

**Interpretation:**

Severe infections requiring hospital treatment are associated with long-term increased risk of dementia, including vascular dementia and Alzheimer's disease. This association is not limited to CNS infections, suggesting that systemic effects are sufficient to affect the brain. The absence of infection specificity combined with evidence of dose-response relationships between infectious disease burden and dementia risk support the hypothesis that increased dementia risk is driven by general inflammation rather than specific microbes.

**Funding:**

UK Medical Research Council, US National Institute on Aging, Wellcome Trust, NordForsk, Academy of Finland, and Helsinki Institute of Life Science.

**Research in context**

**Evidence before this study**

Infectious diseases are hypothesised to be involved in the aetiology of dementia, but evidence from studies that simultaneously examine a wide range of infections is inadequate. We searched PubMed on April 2, 2020, for observational studies and systematic reviews using the search terms “((Alzheimer* OR dementia)” AND “infectio*” AND “(systematic[sb]) OR (Observational Study[ptyp]))” without restrictions on language or publication date. In observational studies, infectious diseases in general, specific bacterial infections (eg, sepsis, pneumonia, osteomyelitis, urinary tract infection, and cellulitis), and viral infections (hepatitis C, HIV) have been linked to an increased risk of dementia. Additionally, there was suggestive evidence for associations of herpes virus infections, *Toxoplasma gondii* infection, and poor oral health with dementia. No large-scale studies assessed a wide range of infectious diseases in a single analytical setting with adequate control for potential ascertainment bias and reverse causation resulting from the effects of preclinical dementia on susceptibility to infectious diseases.

**Added value of this study**

In this multicohort study, we focused on hospital-treated infections to compare effect sizes across types of infection. The primary analysis was based on individual-level data from three Finnish cohort studies and included 260 490 dementia-free community-dwelling individuals with a median follow-up of 15 years. The main findings were replicated in an independent cohort of 485 708 individuals from UK Biobank (median follow-up 7·7 years). We tracked 925 infectious diseases before dementia onset from national hospital inpatient records. Infectious disease hospitalisations were associated with a 1·5-fold increased risk of dementia, with infections occurring more than 10 years before dementia onset also associated with excess risk. We observed a dose-response relationship between infection burden (number of infection episodes over time and number of co-occurring infections) and dementia (p_trend_=0·0007). Although the greatest risk was observed for infections of the CNS (adjusted hazard ratio [aHR] 3·01), extra-CNS infections were also associated with dementia (aHR 1·47). Dementia risk did not vary substantially by type of infection: bacterial versus viral (aHR 1·50 *vs* 1·70); bacterial infections with sepsis versus without sepsis, extracellular versus intracellular, Gram-positive versus Gram-negative; or herpes virus infection versus other persistent viral infections, although associations with acute viral infections were weaker. Both bacterial and viral infections were more strongly related to vascular dementia than Alzheimer's disease.

**Implications of all the available evidence**

Infectious diseases are associated with increased long-term risk of dementia, including Alzheimer's disease, the strongest risk being for vascular dementia. This increased risk is not limited to CNS infections, suggesting that systemic infections are sufficient to affect the brain. Analyses stratified by severity of infection, location (extracellular *vs* intracellular) or Gram stain of bacteria, and type of virus provide no support for the hypothesis that specific pathogens underlie the infection-dementia association. The dose-response relationship observed between the number of episodes of hospital-treated infection and dementia suggests the increased risk might be attributable to general inflammation. These findings show the potentially important role of severe infections in the cause of Alzheimer's disease and other dementias. Further studies should determine whether strategies to improve infection control could prevent or delay dementia.

## Introduction

Although dementia is the fifth leading cause of death worldwide, poor understanding of its aetiology hampers prevention.^1^ Several lines of research suggest a role for inflammation and infectious disease.^2^, ^3^, ^4^ Genes that predispose individuals to dementia are involved in inflammatory pathways;^2^ systemic inflammation has been associated with accelerated cognitive decline and dementia in prospective studies;^5^, ^6^, ^7^ and associations between infection and dementia have been found in several independent cohorts.^8^, ^9^, ^10^, ^11^, ^12^, ^13^

Alternative explanations of these findings exist. The germ hypothesis, supported by animal models of herpes simplex virus type 1,^14^, ^15^ proposes that specific microbes can cause Alzheimer's disease;^3^, ^4^, ^16^ however, there is little, inconsistent evidence for herpes viruses in the human brain.^3^, ^16^, ^17^, ^18^, ^19^ An extension to the germ hypothesis—ie, the antimicrobial protection model of Alzheimer's disease—suggests that accumulation of amyloid β (a diagnostic biomarker of Alzheimer's disease and an antimicrobial peptide) is a physiological response against invading pathogens.^20^ A broader inflammation hypothesis suggests that systemic inflammation more generally contributes to the development of Alzheimer's disease and other dementias.^3^, ^16^ Consistent with this explanation, people with severe acute events, such as sepsis and delirium, have an increased risk of cognitive decline,^7^, ^21^ and many infectious diseases (eg, pneumonia, osteomyelitis, cellulitis, urinary tract infections, and herpes virus infections) are associated with higher subsequent risk of dementia.^8^, ^9^, ^10^, ^12^, ^22^

The narrow focus of most human studies has not provided strong evidence for whether certain infections—or factors specific to the microbes that cause these infections—are linked to Alzheimer's disease or other dementias, or whether the association between infection and dementia is driven by general inflammation and, thus, infectious diseases in general. A further limitation of current evidence is presented by studies with short follow-up periods. Given the long preclinical phase of dementia, such study designs can lead to inflated effect estimates because of reverse causation (systemic changes related to preclinical dementia increase susceptibility to infection) and ascertainment bias (an infectious disease diagnosis increases the likelihood of a dementia diagnosis).^3^, ^8^, ^19^ Long follow-up periods minimise these biases and help to determine whether infections trigger the early stages of neurodegeneration.

In this large-scale study, we systematically assessed the short-term and long-term dementia risk associated with infectious diseases in general and with specific types of infectious disease in two large cohorts. We addressed this aim by attempting to answer the following questions: is the association between infectious diseases and dementia specific to certain microbes or dementia subtypes, or attributable to inflammation and dementias more generally; is systemic inflammation sufficient to affect the brain or is central nervous system (CNS) involvement necessary for an infection to increase dementia risk; and are infectious diseases associated with dementia long-term when the likelihood for reverse causation and ascertainment bias is reduced.

## Methods

### Study design and population

In this large, multicohort, observational study, the analysis was based on a primary cohort consisting of pooled individual-level data from three harmonised prospective cohort studies linked to national health registries in Finland (the Finnish Public Sector study, the Health and Social Support study, and the Still Working study) and an independent replication cohort from the UK Biobank. Full study details and a flowchart describing participant selection are provided in the appendix (pp 4–8). Briefly, we included adult individuals (≥18 years) who were free of known dementia on study entry. Follow-up was until Dec 31, 2012, in the Health and Social Support study, Dec 31, 2016 in the Finnish Public Sector study and the Still Working study, and Feb 7, 2018, in the replication cohort.

Data collection and analysis in the primary cohort were approved by the ethics committees of the Helsinki and Uusimaa Hospital District, Turku University Central Hospital, and the Finnish Institute of Occupational Health. The replication analysis was done under a generic approval from the National Health Service National Research Ethics Service (11/NW/0382).

### Exposure to hospital-treated infectious diseases

We linked participants of the primary cohort to the Care Register for Health Care (Finland). Participants of the replication cohort were linked to hospital admission data from Hospital Episode Statistics–Admitted Patient Care (England), Scottish Morbidity Records–General/Acute Inpatient and Day Case Admissions (Scotland), and Patient Episode Database for Wales. We retrieved primary and secondary diagnoses of infectious disease from inpatient hospital discharge information from these registries using the International Classification of Diseases 10th Revision (ICD-10) codes. Diagnostic codes for the 8th and 9th revisions (ICD-8 and ICD-9) were converted into ICD-10 codes.

**Figure 1:**
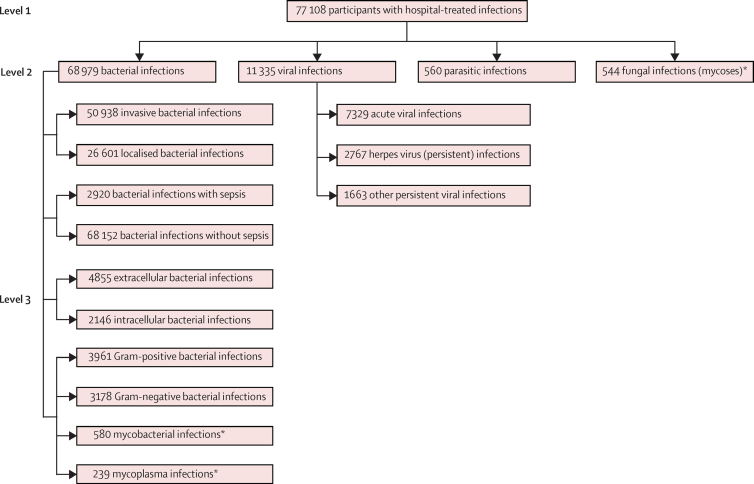
Classification of hospital-treated infectious diseases and the number of cases in the primary cohort The number of cases of different infectious diseases add up to more than the total number of infection cases, because some participants were admitted to hospital for more than one infectious disease. *Too rare to be analysed separately.

For the primary analysis, we classified hospital-treated infectious diseases hierarchically to reflect the type of pathogen and severity of infection (figure 1). Level 1 includes all infectious diseases—ie, a total of 925 ICD-10 codes—except for mild upper respiratory tract infections. At level 2, infectious diseases are divided into bacterial, viral, parasitic, or fungal infections. In level 3, we further classified bacterial infections to reflect properties of the infection and pathogen: disease invasiveness and severity ([potentially] invasive *vs* [mostly] localised, and with sepsis *vs* without sepsis); bacterial location and related adaptive immune responses (extracellular *vs* obligate or facultative intracellular [extracellular bacteria tend to trigger type 17 T-helper responses, intracellular bacteria and viruses tend to trigger type 1 T-helper responses]);^23^ and cell wall structure (Gram-positive *vs* Gram-negative bacteria *vs* mycobacteria *vs* mycoplasma [lipopolysaccharides produced by Gram-negative bacteria strongly stimulate the immune system]).^24^ Classifications of bacterial location and cell wall structure were based only on ICD-10 codes that defined the causative microorganism unambiguously (eg, shigellosis, legionnaires disease, pneumonia due to *Haemophilus influenzae*). Examples of invasive bacterial infections included appendicitis, pneumonia, and pyelonephritis; and localised bacterial infections included gastroenteritis, tonsillitis, and cystitis. We also classified viral infections into acute infections typically eradicated by the immune system, herpes virus infections that persist in the body after primary infection, and other persistent viral infections such as HIV. Mycobacterial, mycoplasma, parasitic, and fungal infections were too rare to be analysed separately, but they were included in the analyses of the broader categories of infections to which they belonged.

In additional analyses, we further divided infections into CNS versus extra-CNS infections; infections predisposed towards entering the CNS versus not predisposed; and chronic versus acute infections. The appendix (pp 29–193) provides the ICD codes for these disease categories as well as the distribution of infections (pp 194–298).

### Ascertainment of incident dementia after exposure to infection

In the primary cohort, we retrieved diagnoses of incident dementia from four sources: hospital inpatient records from the Care Register for Health Care (Finland); reimbursement for the treatment of dementia recorded by the Finnish Social Insurance Institution (these require verification by neurological examination, cognitive testing, clinical follow-up, and, for Alzheimer's disease, CT or MRI scans); causes of death recorded by Statistics Finland; and, in the Finnish Public Sector study and the Still Working study, hospital outpatient records from the Care Register for Health Care (appendix pp 5–7). The first dementia diagnosis, whether primary or secondary in any of these sources, defined the date of incident dementia.

A diagnosis of all-cause dementia consisted of the following ICD-10 codes: F00–F03, F05.1, G30, G31.0, G31.1, G31.8, and the corresponding ICD-8 (29000–29019, 34791, 34792) and ICD-9 (290, 2900A, 2941A, 3310A, 3311A, 3312X, 4378A) codes in the Finnish national editions of the ICDs. We also considered subtypes of dementia; Alzheimer's disease (F00, G30, 29010, 3310A) versus other types of dementia further divided into frontotemporal dementia (G31.0, F02.0, 29011, 34791, 3311A), Parkinson's disease dementia (F02.3), vascular dementia (F01, 4378A), other specified dementia (G31.8, F02.1, F02.2, F02.4, F02.8), and unspecified dementia (F03, G31.1, F05.1, F02.39, 29000, 29019, 290, 2900A, 2941A, 34792, 3312X).

In the replication cohort, we defined all-cause dementia and its subtypes (Alzheimer's disease, vascular dementia, and frontotemporal dementia) using validated cohort algorithms (appendix p 8). For Parkinson's disease dementia (F02.3) and other specified dementia (G31.8, F02.1, F02.2, F02.4, F02.8), we used diagnoses from hospital admission records as no algorithm was available. Unspecified dementia comprised F03, G31.1, F05.1, F02.39, and algorithm-based all-cause dementia without specified cause.

### Assessment of covariates and comorbidities

Covariates included common risk factors for infection and dementia^25^, ^26^, ^27^ and comorbidities. Sex, socioeconomic status (low, intermediate, high), smoking (never smokers, ex-smokers, current smokers), and alcohol (non-drinkers, moderate drinkers, intermediate drinkers, and heavy drinkers [in the Still Working study, drinking data were available in three classes: non-drinkers, moderate drinkers, and heavy drinkers]) were considered in both the primary and replication cohorts at study entry. In the replication cohort, we additionally included body-mass index ([BMI] normal weight, overweight, obese) and apolipoprotein E genotype (none, one, or, two ε4 alleles), based on two single nucleotide polymorphisms (rs7412 and rs429358) genotyped using UK BiLEVE Axiom array (Affymetrix; Santa Clara, CA, USA) and UK Biobank Axiom array (Affymetrix; Santa Clara, CA, USA).

Hypertension, diabetes, ischaemic heart disease, cerebrovascular disease, and Parkinson's disease at baseline were considered as comorbidities potentially increasing the risk of infections and dementia. These comorbidities were defined using primary and secondary diagnoses from hospital inpatient discharge information supplemented by reimbursement records in the primary cohort and measurements and self-reports in the replication cohort.

Exact definitions and distributions of the covariates and comorbidities in each cohort are provided in the appendix (pp 5–10).

### Statistical analysis

We used Cox proportional hazards models to compute hazard ratios (HRs) for the associations of each infectious disease or disease group (index infection) with incident dementia. All CIs are reported at the 95% level. Participants with infection at or before study entry or during the study were considered exposed and the other participants with no infection were considered unexposed. Among the exposed, follow-up for incident dementia lasted from study entry or from the date of hospitalisation for infection to dementia diagnosis, death, or end of follow-up, whichever came first. To ensure comparable dementia follow-up between exposed and unexposed individuals, proportions of participants in the two groups were matched for those exposed before and after study entry within each cohort, sex, and 10-year age group. Follow-up for participants exposed before study entry commenced on entry. Follow-up for the remaining unexposed participants corresponded with the later start of follow-up in participants exposed after study entry.

In the primary analysis (ie, the primary cohort), we pooled individual-level data from the Finnish cohort studies and accounted for the within-study clustering of participants using cohort-specific baseline hazards and cohort-specific adjustment terms for covariates.^28^ We adjusted the analyses for sex and socioeconomic status and used age as the timescale. We used Wald tests to compute p values for differences between the dementia risk related to different infections. The proportional hazards assumption was examined using scaled Schoenfeld residuals (appendix pp 11, 13–15).

To reduce the risk of reverse causation and ascertainment bias, we repeated the analysis after excluding incident dementia cases that occurred during the first 10 years after initial hospitalisation due to the index infection. For those unexposed to any hospital-treated infection, we used a similar distribution of lag-times between study entry and start of dementia follow-up. We tested the interaction between time since infection and risk of dementia in Cox models adjusted for age, age squared, sex, and socioeconomic status, using follow-up time as the timescale. We computed Fine-Gray models with death and, for analysis of late-onset dementia, also early onset dementia (dementia onset before 65 years) as competing risks. Furthermore, to test the robustness of the infection–dementia association, we adjusted models for smoking, alcohol drinking, year of birth, and, in the replication cohort, BMI, diabetes, hypertension, and apolipoprotein E genotype. We repeated the analyses after excluding those with comorbidities (including HIV infection).

Finally, to test whether the results can be replicated using other statistical approaches, we repeated the main analyses using time-dependent Cox regression with infections treated as time-varying measures.

We did all data analyses using Stata MP (version 16). The syntax for the analyses is available in the appendix (pp 299–441).

### Role of the funding source

The funders of the study had no role in study design, data collection, data analysis, data interpretation, or writing of the report.

## Results

In the primary analysis—ie, the primary cohort consisting of the three Finnish studies—260 490 participants were enrolled between March 1, 1986, and Jan 1, 2005, of whom 153 461 (58·9%) were in the 18–39 age group when the dementia follow-up commenced (table). In the replication analysis—ie, replication cohort comprising the UK Biobank—a total of 485 708 participants were enrolled from Dec 19, 2006, to Oct 1, 2010, of whom only two (<0·1%) were in the 18–39 age group when the dementia follow-up commenced. Conversely, the replication cohort had 250 792 (51·6%) participants in the 60–87 age group, compared with 24 131 (9·3%) in the primary cohort. There were 182 976 (70·2%) women in the primary cohort and 264 682 (54·5%) in the replication cohort. The cohorts were from diverse socioeconomic backgrounds. In the primary analysis, 126 815 (55·7%) of the 227 673 participants were from high socioeconomic positions in the Finnish Public Sector study, 3876 (16·5%) of 23 541 in the Health and Social Support study, and 663 (7·1%) of 9276 in the Still Working study; in the replication cohort, 158 997 (32·7%) of 485 708 were from high socioeconomic positions (appendix pp 9–10).

**Table:** Baseline characteristics of the primary and replication cohorts

	**Primary cohort (n=260 490)**	**Replication cohort (n=485 708)**
**Age at baseline (years)**		
18–39	153 461 (58·9%)	2 (<0·1%)
40–49	48 221 (18·5%)	88 472 (18·2%)
50–59	34 677 (13·3%)	146 442 (30·2%)
60–87	24 131 (9·3%)	250 792 (51·6%)
**Sex**		
Male	77 514 (29·8%)	221 026 (45·5%)
Female	182 976 (70·2%)	264 682 (54·5%)
**Socioeconomic status**		
Low	39 878 (15·3%)	83 984 (17·3%)
Intermediate	89 258 (34·3%)	242 727 (50·0%)
High	131 354 (50·4%)	158 997 (32·7%)
**Follow-up (years)**		
Median	15·4 (9·8–21·0)	7·7 (4·0–8·9)
**Dementia by the end of follow-up**		
No	257 722 (98·9%)	483 576 (99·6%)
Yes	2768 (1·1%)	2132 (0·4%)
**Age at dementia diagnosis**		
Median	73·0 (66·8–77·7)	72·0 (68·2–74·8)

Data are n (%) or median (IQR).

In the primary cohort, 77 108 participants were hospitalised because of an infection (figure 1). Of them, 40 145 (52·1%) were infected at or before study entry and 36 963 (47·9%) developed an infection after study entry (incidence 922 cases per 100 000 person-years [95% CI 912–932]). During 3 947 046 person-years at risk (median follow-up 15·4 years [IQR 9·8–21·0]), we identified 2768 incident cases of dementia. Of these, 1730 (62·5%) were diagnosed as Alzheimer's disease, 209 (7·6%) as vascular dementia, 102 (3·7%) as frontotemporal dementia, 114 (4·1%) as Parkinson's disease dementia, and 613 (22·1%) as other or unspecified dementias. We identified 1018 (36·8%) of 2768 incident dementia cases from inpatient hospital discharge records, 786 (28·4%) from other hospital records, 910 (32·9%) from reimbursement for the treatment of Alzheimer's disease or Parkinson's disease dementia, and 54 (2·0%) from death certificates. 2226 (80.4%) of the dementia cases were diagnosed at or after age 65 years (appendix p 16).

**Figure 2:**
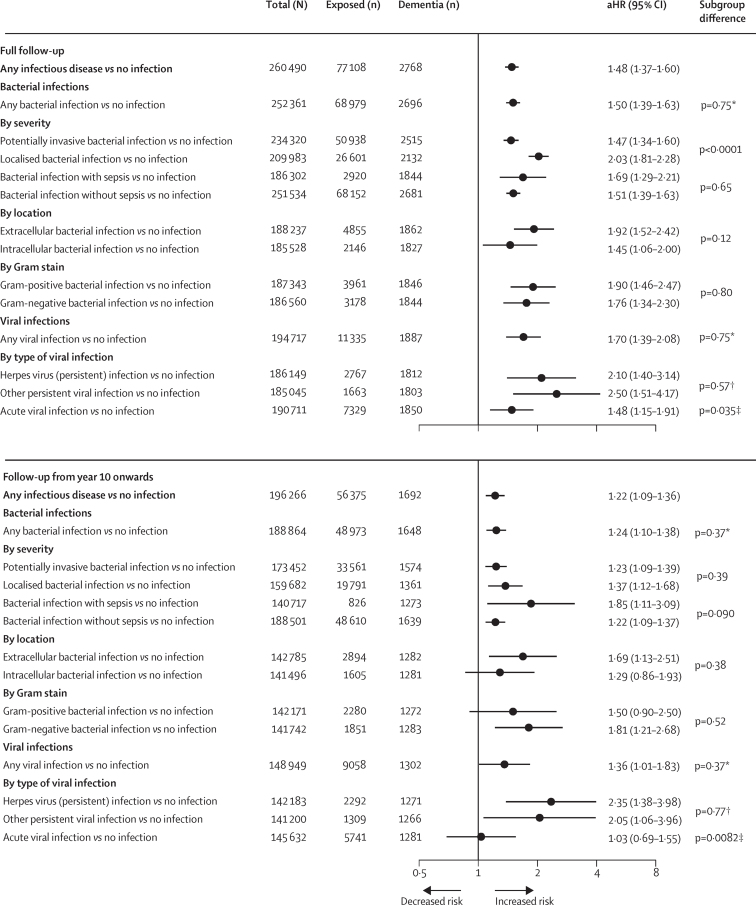
Risk of dementia associated with hospital-treated infectious diseases in the full follow-up and after 10 years or more from the onset of infection in the primary cohort Error bars are 95% CIs. HRs are adjusted for sex and socioeconomic status, and age is the timescale. aHR=adjusted hazard ratio. *Difference in the association of bacterial and viral infections with dementia. †Difference in the association of herpes virus infections and other persistent viral infections with dementia. ‡Difference in the association of acute viral infection and that of herpes and other persistent viral infections with dementia.

The adjusted HR (aHR) for admission to hospital for any infectious disease was 1·48 (95% CI 1·37–1·60) compared with no such hospitalisation (figure 2). The cumulative hazard estimate showed concurring evidence (appendix p 17). Associations between any bacterial or any viral infection and dementia were of comparable strength. HRs varied between 1·45 and 2·50 for extracellular versus intracellular bacteria, Gram-positive versus Gram-negative bacteria, invasive versus localised infection, status of sepsis, and type of virus (herpes virus *vs* other persistent virus *vs* acute viral infection; figure 2). Depending on infection, dementia incidence was 92·3 cases to 135·3 cases per 100 000 person-years for those exposed to infection versus 62·8 cases per 100 000 person-years for those not exposed to infection when the data were standardised to match the age-distribution of the unexposed (appendix p 18). Analyses of the most common Gram-positive and Gram-negative infections and analyses by category of herpes virus infections showed no difference in dementia risk (appendix pp 19, 20). For herpes viruses, dementia risk remained increased after excluding severe infections (appendix p 20).

Despite an interaction between infection and follow-up time (aHR for interaction 0.79 [95% CI 0·74–0·85] per ln [time in years]), the infection–dementia association was not attributable to infections near the time of dementia diagnosis (figure 2). In analyses including only dementia cases that occurred more than 10 years after infection, HRs were significant for all infections combined (aHR 1·22, 95% CI 1·09–1·36), for bacterial and viral infections separately, and for most subtypes (figure 2). Infections were also associated with dementia when competing risk of death and early onset dementia were considered in the analysis (appendix p 21).

**Figure 3:**
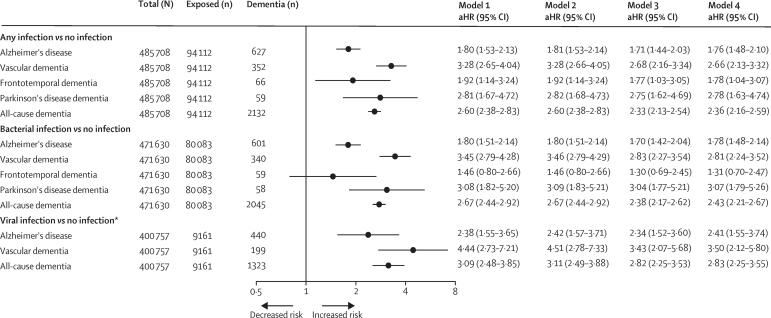
Multivariable-adjusted associations between hospital-treated infections and dementia by dementia type in the replication cohort Data are adjusted HRs (95% CIs), unless otherwise specified. Error bars are 95% CIs. Model 1 was adjusted for age (as the timescale), sex, and socioeconomic status. Numbers of participants, dementia cases, and the forest plot are for this model. Model 2 used the same adjustment criteria as model 1 and excluded participants with HIV infection; it was based on 485 453 participants (2131 [0·4%] with dementia) with complete information for analysis of any infection, 471 511 participants (2044 [0·4%] with dementia) for analysis of bacterial infections, and 400 502 participants (1322 [0·3%] with dementia) for analysis of viral infections. Model 3 used the same criteria as model 2 and additionally adjusted for alcohol drinking, smoking, body-mass index, hypertension, and diabetes; it was based on 480 842 participants (2080 [0·4%] with dementia) with complete information for analysis of any infection, 467 058 participants (1995 [0·4%] with dementia) for analysis of bacterial infections, and 397 333 participants (1304 [0·3%] with dementia) for analysis of viral infections. Model 4 used the same criteria as model 3 and additionally adjusted for apolipoprotein E genotype; it was based on 470 551 participants (2025 [0·4%] with dementia) with complete information for analysis of any infection, 457 104 participants (1945 [0·4%] with dementia) for analysis of bacterial infections, and 389 067 participants (1267 [0·3%] with dementia) for analysis of viral infections. aHR=adjusted hazard ratio. *The number of patients with dementia and viral infection was less than five for frontotemporal dementia and Parkinson's disease dementia.

Infection was associated with an increased risk of Alzheimer's disease, but associations were stronger for non-Alzheimer's dementias (appendix pp 22–23). For example, for any hospital-treated infection, the aHR was 2·09 [95% CI 1·59–2·75] for vascular dementia versus aHR 1·20 [95% CI 1·08–1·33] for Alzheimer's disease. This finding of the primary cohort was also observed in the replication cohort, in which 2132 patients with dementia were recorded over 3 172 717 person-years at risk (median follow-up 7·7 years [IQR 4·0–8·9]). For any hospital-treated infection, the strongest association was observed for vascular dementia (aHR 3·28 [95% CI 2·65–4·04]), followed by Parkinson's disease dementia (aHR 2·81 [1·67–4·72]), frontotemporal dementia (aHR 1·92 [95% CI 1·14–3·24]), and Alzheimer's disease (aHR 1·80 [95% CI 1·53–2·13]). For all-cause dementia, the aHR was 2·60 (95% CI 2·38–2·83). Infections were associated with all-cause dementia and dementia subtypes after adjustment for dementia risk factors and comorbidities (age, sex, socioeconomic status, alcohol drinking, smoking, BMI, hypertension, diabetes, and apolipoprotein E genotype) and after exclusion of participants with HIV infection, ischaemic heart disease, cerebrovascular disease, and Parkinson's disease (figure 3; appendix p 23).

**Figure 4:**
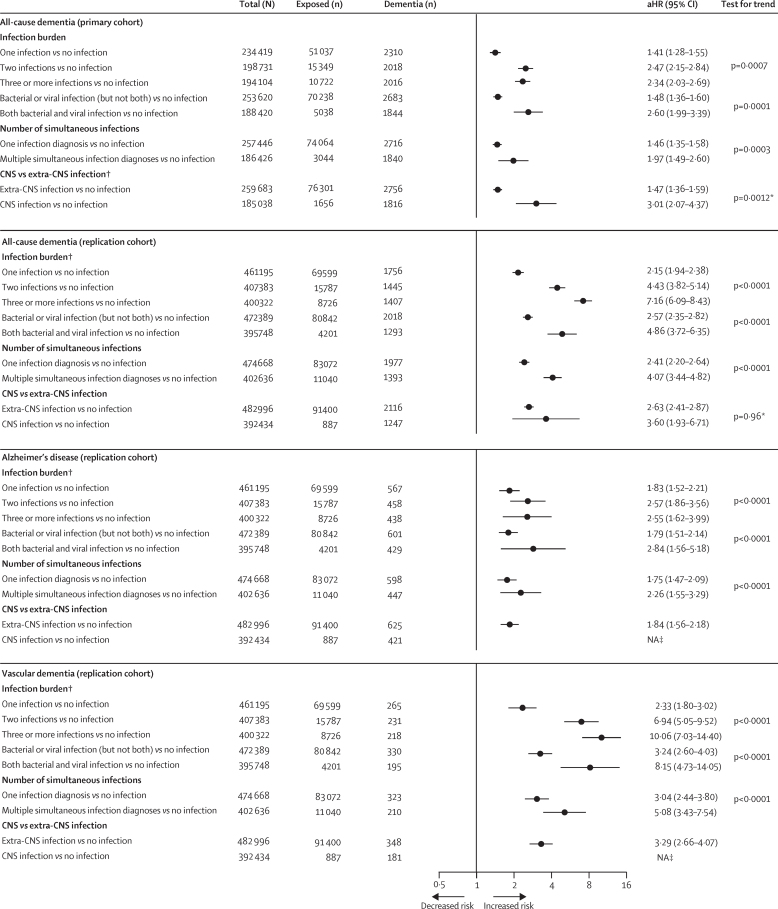
Risk of dementias associated with infection burden, simultaneous infections, and CNS *vs* extra-CNS infections in the primary cohort and replication cohort Infections are hospital-treated. Error bars are 95% CIs. HRs are adjusted for sex and socioeconomic status, and age is the timescale. aHR=adjusted hazard ratio. NA=not applicable. *For subgroup difference. †If the participant was admitted to hospital several times for exactly the same diagnosis, only the first counted towards infection burden because the UK Biobank data included only the first hospitalisation for each diagnosis. ‡Fewer than five Alzheimer's disease and vascular dementia cases among those exposed to CNS infections.

In the primary cohort, the aHR for all-cause dementia was 1·41 (95% CI 1·28–1·55) for one hospital-treated infection versus no infection, 2·47 (2·15–2·84) for two hospital-treated infections versus no infection, and 2·34 (2·03–2·69) for three or more hospital-treated infections versus no infection (p_trend_=0·0007; figure 4). Corresponding aHRs in the replication cohort were 2·15 (95% CI 1·94–2·38) for one infection versus no infection, 4·43 (3·82–5·14) for two infections versus no infection, and 7·16 (6·09–8·43) for three or more hospital-treated infections versus no infection. A similar dose-response relationship was noted for Alzheimer's disease and vascular dementia in the replication cohort (figure 4). The increased dementia risk associated with multiple versus single infection at hospitalisation, compared with no infection, provided further support for a dose-response relationship. Although CNS infections were strongly related to all-cause dementia (aHR 3·01 [95% CI 2·07–4·37] in the primary cohort and aHR 3·60 [1·93–6·71] in the replication cohort) extra-CNS infections were also associated with an increased risk (1·47 [1·36–1·59] in the primary cohort and 2·63 [2·41–2·87] in the replication cohort).

In the primary cohort, when including only dementia cases diagnosed at least 10 years after infection, the aHRs for incident dementia were 1·12 (95% CI 0·97–1·29) for one infection, 1·15 (0·83–1·60) for two infections, and 1·68 (1·25–2·25) for three or more infections; 1·22 (1·09–1·37) for bacterial or viral infection (but not both) and 1·88 (1·09–3·26) for both bacterial and viral infection; 1·21 (1·08–1·35) for one diagnosis and 1·47 (0·86–2·49) for multiple simultaneous infection diagnoses; 1·21 (1·09–1·35) for extra-CNS and 1·44 (0·72–2·89) for CNS infections. These analyses were not possible to repeat in the replication cohort due to insufficient follow-up time. In the primary cohort, the most common CNS infections were unspecified viral meningitis (n=280 [16·9%] of 1656), unspecified viral infection of the CNS (n=167 [10·1%] of 1656), and unspecified viral encephalitis (n=152 [9·2%] of 1656); followed by unspecified bacterial meningitis (n=147 [8·9%] of 1656) and enteroviral meningitis (n=145 [8·8%] of 1656)**]**. Common extra-CNS infections were acute appendicitis (n=11 903 [15·6%] of 76 301), unspecified pneumonia (n=6088 [8·0%] of 76 301), other gastroenteritis and colitis of infectious and unspecified origin (n=5639 [7·4%] of 76 301; appendix pp 252–67).

In the supplementary analyses, infection–dementia associations did not differ by characteristics such as chronicity (acute *vs* chronic, including periods when the pathogen was inactive) and capacity of the infection to enter the CNS (appendix p 24). Finally, all the main findings in both the primary and replication cohorts remained consistent when infections were treated as time-varying measures (appendix pp 25–28).

## Discussion

We assessed associations between a comprehensive set of hospital-treated infections and dementia risk in a pooled analysis of about 260 000 Finnish adults followed-up for about 15 years and about 485 000 UK Biobank participants followed-up for about 8 years. Those with infections had a 1·5-fold increased risk of dementia in the primary cohort and a 2·6-fold increased risk over the shorter follow-up in the replication cohort. These associations were similar for bacterial and viral infections and showed little specificity by type or severity of infection. However, there was a dose-response relationship between multiple episodes of hospital-treated infection and increased dementia risk. This increased dementia risk was observed in sensitivity analyses restricted to extra-CNS infections, when reverse causation was minimised in analyses restricted to infections more than 10 years before dementia onset, and after adjustments for comorbidities, lifestyle-related factors, and apolipoprotein E genotype.

Collectively, our findings suggest that systemic inflammation rather than specific infections or pathogens is driving the development of dementia. This inference is supported by evidence that a range of different infectious diseases is associated with increased risk of cognitive decline and dementia^7^, ^8^, ^9^, ^10^, ^22^, ^29^, ^30^ and by investigations linking systemic inflammation to faster cognitive decline in Alzheimer's disease.^31^ In animal models, progressing neurodegeneration has been associated with long-term priming of the microglia (the resident macrophages of the brain) to a proinflammatory state.^32^, ^33^, ^34^ Microglial priming might also be initiated by inflammatory stimuli, such as lipopolysaccharides produced by Gram-negative bacteria, and might increase the deposition of amyloid plaques that characterises Alzheimer's disease.^35^ In the present study, the strongest associations between infection and dementia were seen for vascular dementia, suggesting a role for vascular mechanisms in the infection-related neuropathology. Animal and in-vitro studies suggest that systemic inflammation can adversely affect brain capillaries causing blood–brain barrier dysfunction and related entry of neurotoxic plasma components, blood cells, and pathogens into the brain, a process leading to neuroinflammation and neuron loss.^36^, ^37^, ^38^ Blood–brain barrier dysfunction might also induce microbleeds and perivascular oedema, compromise microcirculation, and subsequently increase ischaemic damage.^36^, ^39^

We found stronger associations between infection and dementia in the short term than in the long term. Reverse causation and ascertainment bias can contribute to short-term associations, but infections might also accelerate or exacerbate existing neuropathology.^3^, ^8^, ^31^ Robust—albeit weaker—long-term associations, involving infections that occurred at least 10 years before dementia, suggest that infections might also trigger early stages of neurodegeneration. This possibility is supported by other studies with long-term follow-up periods,^11^, ^12^ and an infection-dementia risk of 1·2 after exclusion of the first 2 years of follow-up.^9^

We found no significant difference in dementia risk between acute and chronic infections. Although chronic infections plausibly cause a greater inflammatory burden than acute infections, the most common chronic infections tend to be milder (eg, anogenital warts, chronic periodontitis) than the most common acute infections (eg, acute appendicitis, pneumonia), or primarily remain in a latent state (eg, mononucleosis).

This study has important strengths. With more than 900 infectious diseases and about 700 000 participants, our study is, to our knowledge, the largest and most comprehensive examination of the infectious disease–dementia association to date. Although the primary and replication cohorts were different, findings were largely consistent, supporting the generalisability of our observations. Our analyses of long-term associations with dementia minimised reverse causation and ascertainment biases. As disease ascertainment was from nationwide register data, follow-up was virtually complete and independent of active participation in the studies.

Limitations include potential residual confounding by frailty and undiagnosed comorbidities; ascertainment of dementia from electronic health records, which miss undiagnosed and milder cases;^40^ and a lack of information about biomarkers, detailed neuropathology, and infection treatments that might affect dementia risk. Response from the UK Biobank was low (503 317 [5·5%] of 9 238 453).^41^ This effect might have contributed to underestimation of dementia incidence, but new analyses suggest close agreement between findings from the UK Biobank and representative UK samples for risk factor-disease associations.^42^

In conclusion, our findings support the hypothesis that associations between infectious diseases and dementia are attributable to general inflammation rather than to specific microbes or infections in the CNS. Our data also suggest that mechanisms contributing to vascular dementia might be particularly important drivers of the infection-dementia association.

## Data sharing

Statistical code is provided in the appendix (pp 299–441) and it is also downloadable from GitHub. Data, protocols, and other metadata of the UK Biobank are available to the scientific community. Please refer to the UK Biobank data sharing policy. In the Finnish cohort studies, the pseudonymised questionnaire data used in this study can be shared by request to the investigators. Linked health records require separate permission from the Finnish Institute of Health and Welfare and Statistics Finland.

## Declaration of interests

PNS reports funding from the Helsinki Institute of Life Science, NordForsk, and the Academy of Finland during the conduct of the study, and from the Finnish Foundation for Alcohol Studies outside of the submitted work. JVL reports funding from the Academy of Finland during the conduct of the study. STN reports funding from NordForsk during the conduct of the study. TES reports funding from the Academy of Finland; consultation fees from Servier, Orion, and Novartis outside the submitted work; and is a member of the European Geriatric Medicine Society special interest group on Cardiovascular Medicine in Older People and Diabetes in older people. MK reports funding from the Helsinki Institute of Life Science, the Academy of Finland, NordForsk, UK Medical Research Council, the US National Institute on Aging, and the Wellcome Trust during the conduct of the study. CH, JV, and ME report funding from the Academy of Finland. All other authors declare no competing interests.
