# Supplementary material for: Hospital-treated infectious diseases and the risk of dementia: a large, multicohort, observational study with a replication cohort
Source: Lancet Infect Dis. 2021 Nov;21(11):1557–67. doi: 10.1016/S1473-3099(21)00144-4 (PMC8592915; doi:10.1016/S1473-3099(21)00144-4)
Supplement: Supplementary appendix [file mmc1.pdf]

THE LANCET
Infectious Diseases

Supplementary appendix

This appendix formed part of the original submission and has been peer reviewed. We post it as supplied by the authors.

Supplement to: Sipilä PN, Heikkilä N, Lindbohm JV, et al. Hospital-treated infectious diseases and the risk of dementia: a large, multicohort, observational study with a replication cohort. *Lancet Infect Dis* 2021; published online June 21. https://doi.org/10.1016/S1473-3099(21)00144-4.

Supplementary appendix

Sipilä PN, Heikkilä N, Lindbohm JV, Hakulinen C, Vahtera J, Elovainio M, Suominen S, Väänänen A, Koskinen A, Nyberg ST, Pentti J, Strandberg TE, Kivimäki M. Hospital-treated infectious diseases and the risk of dementia: a large, multicohort, observational study with a replication cohort in the UK Biobank

CONTENTS

eFigure 1. Selection of participants in the primary analysis (Finnish multicohort sample) 4

eMethods 1. Study cohorts and data collection 5

The Finnish Public Sector study (FPS) 5

The Health and Social Support study (HeSSup)... 6

The Still Working study (STW) 6

UK Biobank... 7

eTable 1. Characteristics of participants at baseline by cohort in the primary analysis (Finnish multicohort sample) 9

eTable 2. Characteristics of participants at baseline in the replication cohort (UK Biobank) 10

eMethods 2. Proportionality of hazards 11

Supplementary references 12

eFigure 2. Visualisation of hazard ratios over time using exponentiated scaled Schoenfeld residuals in the primary cohort (Finnish multicohort sample)...13

eFigure 3. Distribution of age at dementia diagnosis in the primary cohort (Finnish multicohort sample).....16

eFigure 4. Nelson-Aalen cumulative hazard estimate for the association of any hospital-treated infectious disease with dementia in the primary cohort (Finnish multicohort sample) 17

eTable 3. N (total), N (dementia), and age-standardised dementia incidence rate for the groups with vs without hospital-treated infectious diseases in the primary cohort (Finnish multicohort sample) 18

eFigure 5. Risk of dementia associated with the most common Gram-positive and Gram-negative infections in the primary cohort (Finnish multicohort sample) 19

eFigure 6. Risk of dementia associated with herpes simplex virus 1 or 2 infection, other herpesvirus infection, and any mild herpesvirus infection in the primary cohort (Finnish multicohort sample)................20

eFigure 7. Competing-risk models for the risk of dementia associated with any hospital-treated infectious disease, bacterial infections, and viral infections in the primary cohort (Finnish multicohort sample)...........21

eFigure 8. Risk of Alzheimer's disease (AD) and non-AD dementia associated with any hospital-treated infectious disease and with specific bacterial and viral infections in the primary cohort (Finnish multicohort sample)...22

eTable 4. Risk of dementia associated with any hospital-treated infectious disease in relation to follow ups, types of dementia, adjustments, and exclusions of participant groups in the primary and replication cohorts (Finnish multicohort sample and UK Biobank) 23

eFigure 9. Risk of dementia associated with chronicity and predisposition of the infection towards entering the central nervous system (CNS) in the primary cohort (Finnish multicohort sample)................................24

eFigure 10. Selection of participants for time-dependent analysis in the primary analysis (Finnish multicohort sample)...25

eFigure 11. Risk of dementia associated with hospital-treated infectious diseases in time-dependent analysis in the full follow up and after 10 or more years from the onset of infection in the primary cohort (Finnish multicohort sample) 26

eFigure 12. Multivariable-adjusted associations between hospital-treatd infections and dementia by dementia type using time-dependent analysis in the replication cohort (UK Biobank) 27

eFigure 13. Risk of dementias associated with infection burden, simultaneous infections, and central nervous system (CNS) vs extra-CNS infections using time-dependent analysis in the primary and replication cohorts (Finnish multicohort sample and UK Biobank) 28

eTable 5. Classification of hospital-treated infectious diseases in the study on diagnosis level 29

- All infectious diseases 29
- All bacterial infections 53
- Invasive bacterial infections 63
- Localised bacterial infections 71
- Bacterial infections with sepsis 73
- Bacterial infections without sepsis 74
- Intracellular bacterial infections 83
- Extracellular bacterial infections 86
- Gram-positive bacterial infections 89
- Gram-negative bacterial infections 91
- Mycobacterial infections 95
- Mycoplasma infections 96
- Central nervous system infections 96
- Extra-central nervous system infections 97
- Acute infections 115
- Chronic infections 128
- Infections predisposed towards entering the central nervous system 134
- Infections not predisposed towards entering the central nervous system 143
- All viral infections 153
- Acute viral infections 157
- Herpesvirus infections 159
- Other potentially persistent viral infections 160
- Parasitic infections 161
- Fungal infections (mycoses) 163

eTable 6. ICD-10 codes for infectious diseases with the corresponding ICD-8 and ICD-9 codes 166

eTable 7. Distribution of all infections in primary analysis (Finnish multicohort sample) 194

eTable 8. Distribution of bacterial infections in primary analysis (Finnish multicohort sample) 208

eTable 9. Distribution of invasive bacterial infections in primary analysis (Finnish multicohort sample) 217

eTable 10. Distribution of localised bacterial infections in primary analysis (Finnish multicohort sample) 224

eTable 11. Distribution of bacterial infections with sepsis in primary analysis (Finnish multicohort sample) 226

eTable 12. Distribution of bacterial infections without sepsis in primary analysis (Finnish multicohort sample) 227
eTable 13. Distribution of extracellular bacterial infections in primary analysis (Finnish multicohort sample) 235
eTable 14. Distribution of intracellular bacterial infections in primary analysis (Finnish multicohort sample) 238
eTable 15. Distribution of Gram-positive bacterial infections in primary analysis (Finnish multicohort sample) 241
eTable 16. Distribution of Gram-negative bacterial infections in primary analysis (Finnish multicohort sample) 242
eTable 17. Distribution of viral infections in primary analysis (Finnish multicohort sample) 245
eTable 18. Distribution of herpesvirus infections in primary analysis (Finnish multicohort sample) 249
eTable 19. Distribution of other potentially persistent viral infections in primary analysis (Finnish multicohort sample) 249
eTable 20. Distribution of acute viral infections in primary analysis (Finnish multicohort sample) 250
eTable 21. Distribution of cenral nervous system infections in primary analysis (Finnish multicohort sample) 252
eTable 22. Distribution of extra-central nervoys system infections in primary analysis (Finnish multicohort sample) 254
eTable 23. Distribution of acute infections in primary analysis (Finnish multicohort sample) 268
eTable 24. Distribution of chronic infections in primary analysis (Finnish multicohort sample) 278
eTable 25. Distribution of infections predisposed towards entering the central nervous system in primary analysis (Finnish multicohort sample) 283
eTable 26. Distribution of infections not predisposed towards entering the central nervous system in primary analysis (Finnish multicohort sample) 290
eMethods 3. Stata code for data analysis 299

eFigure 1. Selection of participants in the primary analysis (Finnish multicohort sample)

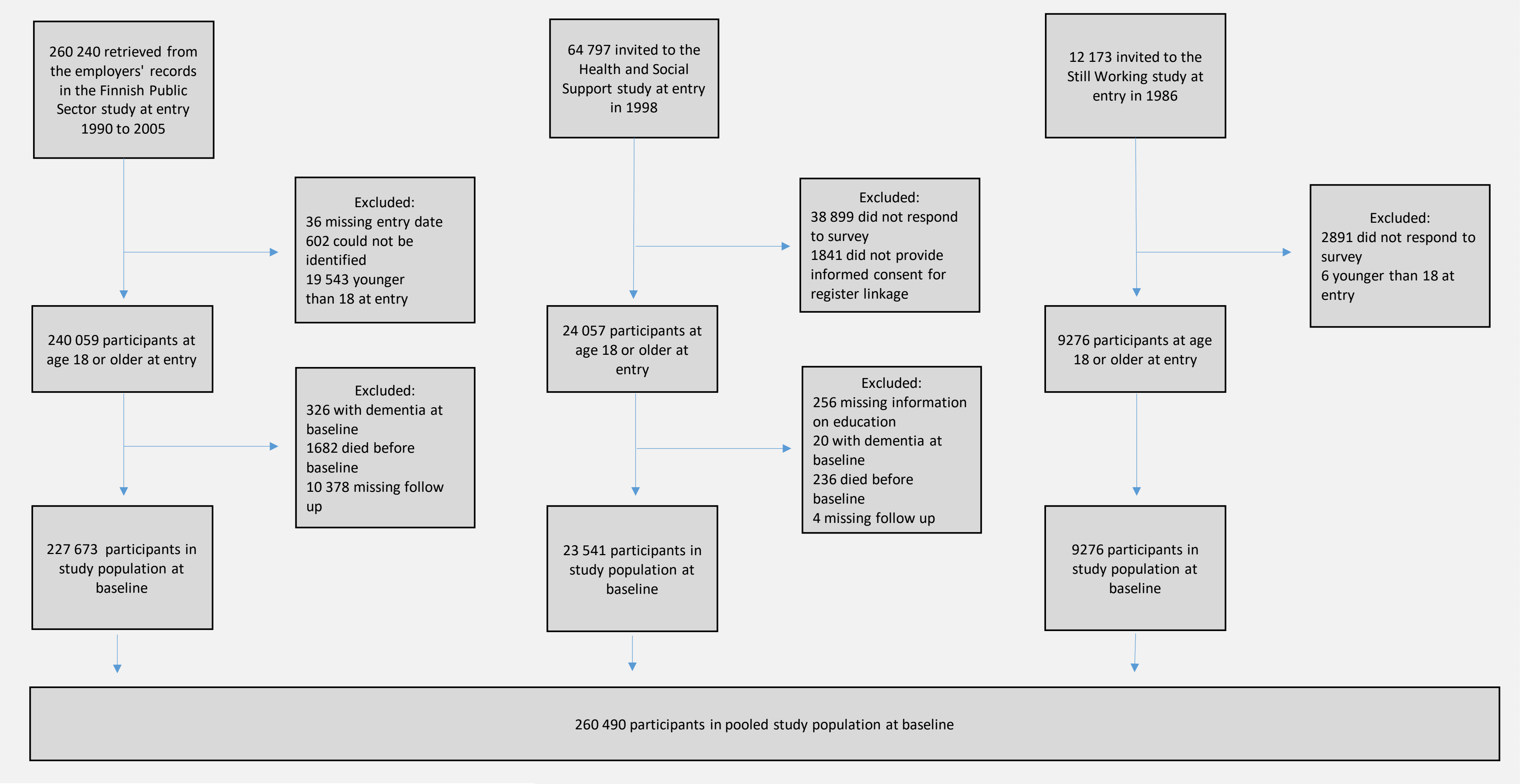

eMethods 1. Study cohorts and data collection

The Finnish Public Sector study (FPS)

The prospective Finnish Public Sector cohort study comprises 260 240 public sector employees from 10 Finnish towns and 6 Finnish hospital districts identified from the employers' registers in 1990-2005.[5] 240 048 of them were available for this study and were linked to national registries of hospital discharge information (dates and diagnoses recorded by the Finnish Institute for Health and Welfare) and medication reimbursement entitlements (dates and indications recorded by the Finnish Social Insurance Institution) using the personal identification codes unique to each Finnish citizen. We also linked the participants to records on dates and causes of death from Statistics Finland. The ethics committee of Helsinki and Uusimaa Hospital District approved the register linkage and data analysis.

Diagnoses of hospital-treated infectious diseases (index infections)

We retrieved both primary and secondary diagnoses of hospital-treated diseases from inpatient hospital discharge information from 1 January 1980 to 31 December 2016, using the International Classification of Diseases, 10th Revision (ICD-10). The diagnosis codes from the 8th and 9th revisions (ICD-8 and ICD-9) were converted into the corresponding ICD-10 codes according to the national editions of the International Classification of Diseases (eTable 6 in this Supplement).[2–4]

Covariates

Participants' birthdate and sex were retrieved from the Finnish Population Information System. Information on the participants' education was available from Statistics Finland and analysed in three classes: low (basic education), intermediate (high school or vocational school), and high (university degree or other tertiary degree).

We defined comorbidities as follows: hypertension, ICD-10 codes I10–I15.9, ICD-8 and -9 codes 400–40499; diabetes mellitus, ICD-10 codes E10–E14.9, ICD-8 and -9 codes 250–25099; ischaemic heart disease, ICD-10 codes I20–I25.9, ICD-8 and -9 codes 410–41499; cerebrovascular disease, ICD-10 codes I60–I69.9, ICD-8 and -9 codes 430–43899; Parkinson's disease, ICD-10 code G20, ICD-8 and -9 codes 332, 3320, 3320A, 34200. Additionally, we retrieved information from prescription medication reimbursement entitlements for the treatment of hypertension, diabetes mellitus, ischaemic heart disease, and Parkinson's disease (no reimbursement entitlements were granted for cerebrovascular disease during the study).

A subsample of the study was invited to respond to mailed questionnaires (N = 114 835), and 92 727 of them responded to at least one questionnaire. Information on smoking status and alcohol drinking was retrieved from the earliest available self-report during the study from June 1997 to November 2013 and was available for 86 021 participants on smoking and 86 435 participants on alcohol drinking. To avoid immortal time bias, in the analyses adjusted for these variables (eTable 4 on p. 23 of this appendix), dementia follow-up commenced from the day of the self-report for the participants whose self-report occurred after baseline. Smoking status was analysed in three categories (never smokers, ex-smokers, current smokers). The participants reported the number of different types of alcoholic drinks they consumed within a week. These reports were summed up to the total amount of alcoholic drinks consumed per week, with one drink estimated to contain approximately 10 grams of pure alcohol, and futher categorized to four categories:[6] non-drinkers, moderate drinkers (women consuming 1–14 drinks per week and men consuming 1–21 drinks per week), intermediate drinkers (women consuming 15–20 drinks per week and men consuming 22–27 drinks per week), and heavy drinkers (women consuming 21 drinks per week or more and men consuming 28 drinks per week or more).

Ascertainment of incident dementia

We compiled dementia diagnoses from hospital discharge information, prescription medication reimbursement entitelments, and causes of death. Medication reimbursement entitlements were granted for the treatment of Alzheimer's disease and Parkinson's disease dementia and required verification of the diagnosis with clinical neurological examination, cognitive testing, clinical follow up, and, in the case of Alzheimer's disease, computed tomography or magnetic resonance imaging. Inpatient discharge information from hospitals and health center wards was available from 1 January 1980 to 31 December 2016, causes of death from 1 January 1980 to 31 December 2016, medication reimbursement entitlements for the treatment of dementia from February 1999 to 31 December 2011, and outpatient hospital visits (including outpatient clinic, emergency room, and day ward visits) from 1 January 2012 to 31 December 2016. The date of incident dementia was defined as the first occurrence of dementia diagnosis, whether primary or secondary, in any of these information sources. A diagnosis of all-cause dementia consisted of ICD-10 codes F00-F03, F05.1, G30, G31.0, G31.1, G31.8, and the corresponding ICD-8 (29000-29019, 34791, 34792) and ICD-9 (290, 2900A, 2941A, 3310A, 3311A, 3312X, 4378A) codes in the Finnish national editions of the International Classification of Diseases. We also considered subtypes of dementia; Alzheimer's disease (F00, G30, 29010, 3310A) versus other types of dementia further divided into frontotemporal dementia (G31.0, F02.0, 29011, 34791, 3311A), Parkinson's disease dementia (F02.3), vascular

dementia (F01, 4378A), other specified dementia (G31.8, F02.1, F02.2, F02.4, F02.8), and unspecified dementia (F03, G31.1, F05.1, F02.39, 29000, 29019, 290, 2900A, 2941A, 34792, 3312X).

The Health and Social Support study (HeSSup)

The Health and Social Support study is a prospective cohort study representative of the Finnish population.[8] A random sample from the Finnish Population Information System, stratified by sex and age group (20–24, 30–34, 40–44, and 50–54 years), was invited to respond a mailed questionnaire in 1998 (N = 64 797). This study comprises 24 057 respondents who returned the questionnaire and provided informed consent for register linkage. They were linked to national registries of hospital discharge information (dates and diagnoses recorded by the Finnish Institute for Health and Welfare) and medication reimbursement entitlements (dates and indications recorded by the Finnish Social Insurance Institution) using the personal identification codes unique to each Finnish citizen. The participants were also linked to records on dates and causes of death from Statistics Finland. The Turku University Central Hospital Ethics Committee approved the study.

Diagnoses of hospital-treated diseases (index diseases)

We retrieved both primary and secondary diagnoses of hospital-treated diseases from inpatient hospital discharge information available from 1 January 1998 to 31 December 2012 (with occasional information available from 4 August 1997 to 31 December 1997). We used the International Classification of Diseases, 10th Revision (ICD-10). All available data were coded according to ICD-10.

Covariates

Participants' birth year and sex were retrieved from the Finnish Population Information System. Information on education was retrieved from self-reports and was analysed in three classes: low (basic education), intermediate (apprenticeship, vocational school, or college degree), and high (university or polytechnic degree). Self-reported smoking status was analysed in three categories (never smokers, ex-smokers, current smokers). The participants reported the number of different types of alcoholic drinks they consumed within a week. These reports were summed up to the total amount of alcoholic drinks consumed per week, with one drink estimated to contain approximately 10 grams of pure alcohol, and futher categorized to four categories:[6] non-drinkers, moderate drinkers (women consuming 1–14 drinks per week and men consuming 1–21 drinks per week), intermediate drinkers (women consuming 15–20 drinks per week and men consuming 22–27 drinks per week), and heavy drinkers (women consuming 21 drinks per week or more and men consuming 28 drinks per week or more).

We defined comorbidities as follows: hypertension, ICD-10 codes I10–I15.9; diabetes mellitus, ICD-10 codes E10–E14.9; ischaemic heart disease, ICD-10 codes I20–I25.9; cerebrovascular disease, ICD-10 codes I60–I69.9; Parkinson's disease, ICD-10 code G20. All available data were coded according to ICD-10. Additionally, we considered prescription medication reimbursement entitlements for the treatment of hypertension, diabetes mellitus, ischaemic heart disease, and Parkinson's disease (no reimbursement entitlements were granted for cerebrovascular disease during the study).

Ascertainment of incident dementia

We compiled dementia diagnoses from inpatient hospital discharge information, reimbursed prescription medication purchases, and causes of death. Medication reimbursement entitlements were granted for the treatment of Alzheimer's disease and Parkinson's disease dementia and required verification of the diagnosis with clinical neurological examination, cognitive testing, clinical follow up, and, in the case of Alzheimer's disease, computed tomography or magnetic resonance imaging. Inpatient discharge information from hospitals and health center wards was available from 1 January 1998 to 31 December 2012 (with occasional information available from 4 August 1997 to 31 December 1997), causes of death from cohort entry (1 January 1998) to 31 December 2012, and medication reimbursement entitlements for the treatment of dementia from February 1999 to 31 December 2012. The date of incident dementia was defined as the first occurrence of dementia diagnosis, whether primary or secondary, in any of these information sources. A diagnosis of all-cause dementia consisted of ICD-10 codes F00-F03, F05.1, G30, G31.0, G31.1, and G31.8. We also considered subtypes of dementia; Alzheimer's disease (F00, G30) versus other types of dementia further divided into frontotemporal dementia (G31.0, F02.0), Parkinson's disease dementia (F02.3), vascular dementia (F01), other specified dementia (G31.8, F02.1, F02.2, F02.4, F02.8), and unspecified dementia (F03, G31.1, F05.1, F02.39). All available data were coded according to ICD-10.

The Still Working study (STW)

The Still Working study is an ongoing prospective cohort study.[9] In 1986, all employees of Enso Gutzeit (a large forest industry company, which nowadays is part of Stora Enso) in Finland were invited to respond to a mailed questionnaire (N = 12 173). 9276 of them were available for this study and were linked to national registries of hospital discharge information (dates and diagnoses recorded by the Finnish

Institute for Health and Welfare) and medication reimbursement entitlements (dates and indications recorded by the Finnish Social Insurance Institution) using the personal identification codes unique to each Finnish citizen. Participants were also linked to records on dates and causes of death from Statistics Finland. The ethics committee of the Finnish Institute of Occupational Health approved the study.

Diagnoses of hospital-treated diseases (index diseases)

We retrieved both primary and secondary diagnoses of hospital-treated diseases from inpatient hospital discharge information from 1971 to 31 December 2016, using the International Classification of Diseases, 10th Revision (ICD-10). The diagnosis codes from the 8th and 9th revisions (ICD-8 and ICD-9) were converted into the corresponding ICD-10 codes according to the national editions of the International Classification of Diseases (eTable 1 in this Supplement).[2–4]

Covariates

Participants' birth year and sex were retrieved from the Finnish Population Information System. Information on occupational grade, smoking status, and alcohol drinking was retrieved from self-reports. Occupational grade was analysed in three categories (low, intermediate, high). Self-reported smoking status was analysed in three categories (never smokers, ex-smokers, current smokers). Based on self-reports on the frequency of alcohol use and whether the amount of alcohol used led to any symptoms, three categories of alcohol consumption were formed: non-drinkers, moderate drinkers, and heavy drinkers.

We defined comorbidities as follows: hypertension, ICD-10 codes I10–I15.9, ICD-8 and -9 codes 400–40499; diabetes mellitus, ICD-10 codes E10–E14.9, ICD-8 and -9 codes 250–25099; ischaemic heart disease, ICD-10 codes I20–I25.9, ICD-8 and -9 codes 410–41499; cerebrovascular disease, ICD-10 codes I60–I69.9, ICD-8 and -9 codes 430–43899; Parkinson's disease, ICD-10 code G20, ICD-8 and -9 codes 332, 3320, 3320A, and 34200. Additionally, we considered prescription medication reimbursement entitlements for the treatment of hypertension, diabetes mellitus, ischaemic heart disease, and Parkinson's disease (no reimbursement entitlements were granted for cerebrovascular disease during the study).

Ascertainment of incident dementia

We compiled dementia diagnoses from hospital discharge information, reimbursed prescription medication purchases, and causes of death. Medication reimbursement entitlements were granted for the treatment of Alzheimer's disease and Parkinson's disease dementia and required verification of the diagnosis with clinical neurological examination, cognitive testing, clinical follow up, and, in the case of Alzheimer's disease, computed tomography or magnetic resonance imaging. Inpatient discharge information from hospitals and health center wards was available from 1971 to 31 December 2016, causes of death from study entry (1 March 1986) to 31 December 2016, medication reimbursement entitlements for the treatment of dementia from February 1999 to 31 December 2016, and outpatient hospital visits (including outpatient clinic, emergency room, and day ward visits) from 1 January 2009 to 31 December 2016. The date of incident dementia was defined as the first occurrence of dementia diagnosis, whether primary or secondary, in any of these information sources. A diagnosis of all-cause dementia consisted of ICD-10 codes F00-F03, F05.1, G30, G31.0, G31.1, G31.8, and the corresponding ICD-8 (29000-29019, 34791, 34792) and ICD-9 (290, 2900A, 2941A, 3310A, 3311A, 3312X, 4378A) codes in the Finnish national editions of the International Classification of Diseases. We also considered subtypes of dementia; Alzheimer's disease (F00, G30, 29010, 3310A) versus other types of dementia further divided into frontotemporal dementia (G31.0, F02.0, 29011, 34791, 3311A), Parkinson's disease dementia (F02.3), vascular dementia (F01, 4378A), other specified dementia (G31.8, F02.1, F02.2, F02.4, F02.8), and unspecified dementia (F03, G31.1, F05.1, F02.39, 29000, 29019, 290, 2900A, 2941A, 34792, 3312X).

UK Biobank

UK Biobank is an ongoing prospective cohort study (https://www.ukbiobank.ac.uk/). In 2006–2010, 500 000 participants aged 40–69 years were recruited from the United Kingdom. The data collection has involved measures, blood, urine and saliva samples, and questionnaires. The participants have agreed to have their health followed. The UK Biobank study has been approved by the North West Multi-centre Research Ethics Committee (MREC). It has also been approved by the Patient Information Advisory Group (PIAG) in England and Wales and the Community Health Index Advisory Group (CHIAG) in Scotland.

Diagnoses of hospital-treated diseases (index diseases)

We retrieved both primary and secondary diagnoses of hospital-treated diseases from linked hospital admission data from HES APC (Hospital Episode Statistics–Admitted Patient Care [England]), SMR01 (Scottish Morbidity Records–General/Acute Inpatient and Day Case Admissions [Scotland]) and PEDW (Patient Episode Database for Wales), using the International Classification of Diseases, 10th Revision (ICD-10).

Covariates

Covariates included participants' birth year and sex, education, smoking status, alcohol drinking, body mass index and apolipoprotein E genotype. Body mass index was measured at baseline and the other covariates were based on self-reports. Education was analysed in three categories (low, intermediate, high). Smoking status was also analysed in three categories (never smokers, ex-smokers, current smokers). Alcohol consumption was self-reported using the question "About how often do you drink alcohol?", and the categories for alcohol drinking were non-drinkers, moderate drinkers (one to three times a month or once or twice a week), intermediate drinkers (three or four times a week), and heavy drinkers (daily or almost daily). Apolipoprotein E genotype (0, 1 or 2 ε4 allelles) was derived from single-nucleotide polymorphism in rs429358 and rs7412 that were genotyped using Affymetrix UK BiLEVE Axiom and Affymetrix UK Biobank Axiom® arrays (https://www.ukbiobank.ac.uk/scientists-3/genetic-data/).

Diabetes mellitus was defined by self-report or hospitalisation for it (ICD-10 codes E10–E14 as primary or secondary diagnosis). Hypertension was defined by measurement at baseline (systolic blood pressure ≥140mmHg) or hospitalisation for it (ICD-10 codes I10–I15 as primary or secondary diagnosis). Ischaemic heart disease was defined by ICD-10 codes I20–I25.9 and ICD-8 and -9 codes 410–41499. Cerebrovascular disease was defined by ICD-10 codes I60–I69.9 and ICD-8 and -9 codes 430–43899. Parkinson's disease was defined by the UK Biobank algorithm (http://biobank.ndph.ox.ac.uk/showcase/showcase/docs/alg_outcome_pdp.pdf) that uses information from hospital records, death records, and self-reports.

Ascertainment of incident dementia

We defined all-cause dementia, Alzheimer's disease, vascular dementia, and frontotemporal dementia according to the UK Biobank algorithm (http://biobank.ndph.ox.ac.uk/showcase/showcase/docs/alg_outcome_dementia.pdf) that uses information from hospital records, death records, and self-reports. For Parkinson's disease dementia (F02.3) and other specified dementia (G31.8, F02.1, F02.2, F02.4, F02.8), we used diagnoses from hospital admission records as no algorithm was available. Unspecified dementia comprised F03, G31.1, F05.1, F02.39, and algorithm-based all-cause dementia without specified cause. The date of incident dementia was defined as the first occurrence of dementia diagnosis.

eTable 1. Characteristics of participants at baseline by cohort in the primary analysis (Finnish multicohort sample)

Demographic		No. (%) Cohort FPS (N = 227 673)	HeSSup (N = 23 541)	STW (N = 9276)
Age at entry, years				
	18-39	139 566 (61.3%)	9955 (42.3%)	3940 (42.5%)
	40-49	40 549 (17.8%)	5064 (21.5%)	2608 (28.1%)
	50-59	27 483 (12.1%)	5592 (23.8%)	1602 (17.3%)
	60-87	20 075 (8.8%)	2930 (12.4%)	1126 (12.1%)
Sex				
	Women	166 956 (73.3%)	13 912 (59.1%)	2108 (22.7%)
Socioeconomic status				
	Low	26 320 (11.6%)	7121 (30.2%)	6437 (69.4%)
	Intermediate	74 538 (32.7%)	12 544 (53.3%)	2176 (23.5%)
	High	126 815 (55.7%)	3876 (16.5%)	663 (7.1%)
Hypertension				
	Yes	12904 (5.7%)	1854 (7.9%)	943 (10.2%)
Diabetes mellitus				
	Yes	4707 (2.1%)	643 (2.7%)	319 (3.4%)
Ischaemic heart disease				
	Yes	2874 (1.3%)	381 (1.6%)	443 (4.8%)
Cerebrovascular disease				
	Yes	1741 (0.8%)	167 (0.7%)	219 (2.4%)
Parkinson's disease				
	Yes	176 (0.1%)	18 (0.1%)	31 (0.3%)
Smoking status				
	Never smoker	55203 (24.2%)	9769 (41.5%)	3214 (34.6%)
	Ex-smoker	15315 (6.7%)	7684 (32.6%)	2917 (31.4%)
	Current smoker	15503 (6.8%)	5996 (25.5%)	3023 (32.6%)
	(not available)	141652 (62.2%)	92 (0.4%)	122 (1.3%)
Alcohol drinking				
	Non-drinker	12783 (5.6%)	3754 (15.9%)	446 (4.8%)
	Moderate	65313 (28.7%)	17428 (74.0%)	8247 (88.9%)
	Intermediate	3300 (1.4%)	1094 (4.6%)	0 (0.0%)
	Heavy	5039 (2.2%)	1233 (5.2%)	434 (4.7%)
	(not available)	141238 (62.0%)	32 (0.1%)	149 (1.6%)
Follow up, median (interquartile range), years		16.0 (11.5-21.0)	7.6 (2.8-12.6)	30.8 (18.0-30.8)
Dementia by the end of follow up				
	Yes	2161 (0.9%)	49 (0.2%)	558 (6.0%)
Age at dementia diagnosis, median (interquartile range), years		72.2 (66.0-77.3)	63.4 (56.9-65.3)	75.4 (71.8-79.1)

Abbreviations: FPS, Finnish Public Sector study; HeSSup, Health and Social Support study; STW, Still Working study.

eTable 2. Characteristics of participants at baseline in the replication cohort (UK Biobank)

Demographic		No. (%) UK Biobank (N = 485 708)
Age at entry, years		
	18-39	2 (0.0%)
	40-49	88 472 (18.2%)
	50-59	146 442 (30.2%)
	60-73	250 792 (51.6%)
Sex		
	Women	264 682 (54.5%)
Socioeconomic status		
	Low	83 984 (17.3%)
	Intermediate	242 727 (50.0%)
	High	158 997 (32.7%)
Hypertension		
	Yes	220 708 (45.4%)
Diabetes mellitus		
	Yes	28 924 (6.0%)
Ischaemic heart disease		
	Yes	26 802 (5.5%)
Cerebrovascular disease		
	Yes	8732 (1.8%)
Parkinson's disease		
	Yes	1238 (0.3%)
Smoking status		
	Never smoker	265 246 (54.6%)
	Ex-smoker	167 746 (34.5%)
	Current smoker	50 953 (10.5%)
	(not available)	1763 (0.4%)
Alcohol drinking		
	Non-drinker	94 443 (19.4%)
	Moderate	179 459 (36.9%)
	Intermediate	112 350 (23.1%)
	Heavy	99 003 (20.4%)
	(not available)	453 (0.1%)
Body mass index (kg/m^2)		
	≤24.9	160 106 (33.0%)
	25.0-29.9	205 243 (42.3%)
	≥30.0	117 825 (24.3%)
	(not available)	2534 (0.5%)
Apolipoprotein E genotype (number of ε4 alleles)		
	0	351 642 (72.4%)
	1	111 749 (23.0%)
	2	11 252 (2.3%)
	(not available)	11 065 (2.3%)
Follow up, median (interquartile range), years		7.7 (4.0-8.9)
Dementia by the end of follow up		
	Yes	2132 (0.4%)
Age at dementia diagnosis, median (interquartile range), years		72.0 (68.2-74.8)

eMethods 2. Proportionality of hazards

We examined the proportional hazards assumption in the Cox models with scaled Schoenfeld residuals. These tests indicated a violation of the proportional hazards assumption for nine groups of infectious diseases ($p < 0.05$): any infectious disease, invasive bacterial infections, extracellular bacterial infections, intracellular bacterial infections, Gram-negative bacterial infections, any viral infection, herpesvirus infections, other potentially persistent viral infections, and extra-CNS infections. We assessed these diseases plotting exponentiated scaled Schoenfeld residuals using symmetric nearest neighbour smoothing (eFigure 2 on pages 13-15 of this Supplement).[1] The exponentiated scaled Schoenfeld residuals can be interpreted as hazard ratios and therefore visualise that the hazard ratios were reasonably stable between ages 65 and 80 when most incident dementia cases occurred in our data.[1]

Supplementary references

1. Royston P, Lambert PC. *Flexible Parametric Survival Analysis Using Stata: Beyond the Cox Model*. College Station, TX: Stata Press; 2011.

2. Lääkintöhallitus. *Classificatio Morborum et Causarum Mortis: Tauti- Ja Kuolinsyyluokitus [Classification of Diseases and Causes of Death]*. Helsinki: Lääkintöhallitus; 1969. http://urn.fi/URN:NBN:fi-fe201710058910.

3. Lääkintöhallitus. *Tautiluokitus 1987 : Osa 1 : Systemaattinen Osa [Classification of Diseases 1987: Part 1: Systematic Part]*. Helsinki: Lääkintöhallitus; 1986. http://urn.fi/URN:NBN:fi-fe201701261356.

4. Terveyden ja hyvinvoinnin laitos (THL). *Tautiluokitus ICD-10 [Classification of Diseases ICD-10]*. 3rd ed. Helsinki: Terveyden ja hyvinvoinnin laitos (THL); 2011. http://urn.fi/URN:NBN:fi-fe201205085423.

5. Kivimäki M, Lawlor DA, Davey Smith G, et al. Socioeconomic position, co-occurrence of behavior-related risk factors, and coronary heart disease: the Finnish Public Sector study. *Am J Public Health*. 2007;97(5):874-879. doi:10.2105/AJPH.2005.078691

6. Heikkilä K, Nyberg ST, Fransson EI, et al. Job strain and alcohol intake: a collaborative meta-analysis of individual-participant data from 140,000 men and women. *PLoS ONE*. 2012;7(7):e40101. doi:10.1371/journal.pone.0040101

7. Lévesque LE, Hanley JA, Kezouh A, Suissa S. Problem of immortal time bias in cohort studies: example using statins for preventing progression of diabetes. *BMJ*. 2010;340:b5087. doi:10.1136/bmj.b5087

8. Korkeila K, Suominen S, Ahvenainen J, et al. Non-response and related factors in a nation-wide health survey. *Eur J Epidemiol*. 2001;17(11):991-999.

9. Väänänen A, Murray M, Koskinen A, Vahtera J, Kouvonen A, Kivimäki M. Engagement in cultural activities and cause-specific mortality: prospective cohort study. *Prev Med*. 2009;49(2-3):142-147. doi:10.1016/j.ypmed.2009.06.026

eFigure 2. Visualisation of hazard ratios over time using exponentiated scaled Schoenfeld residuals in the primary cohort (Finnish multicohort sample)

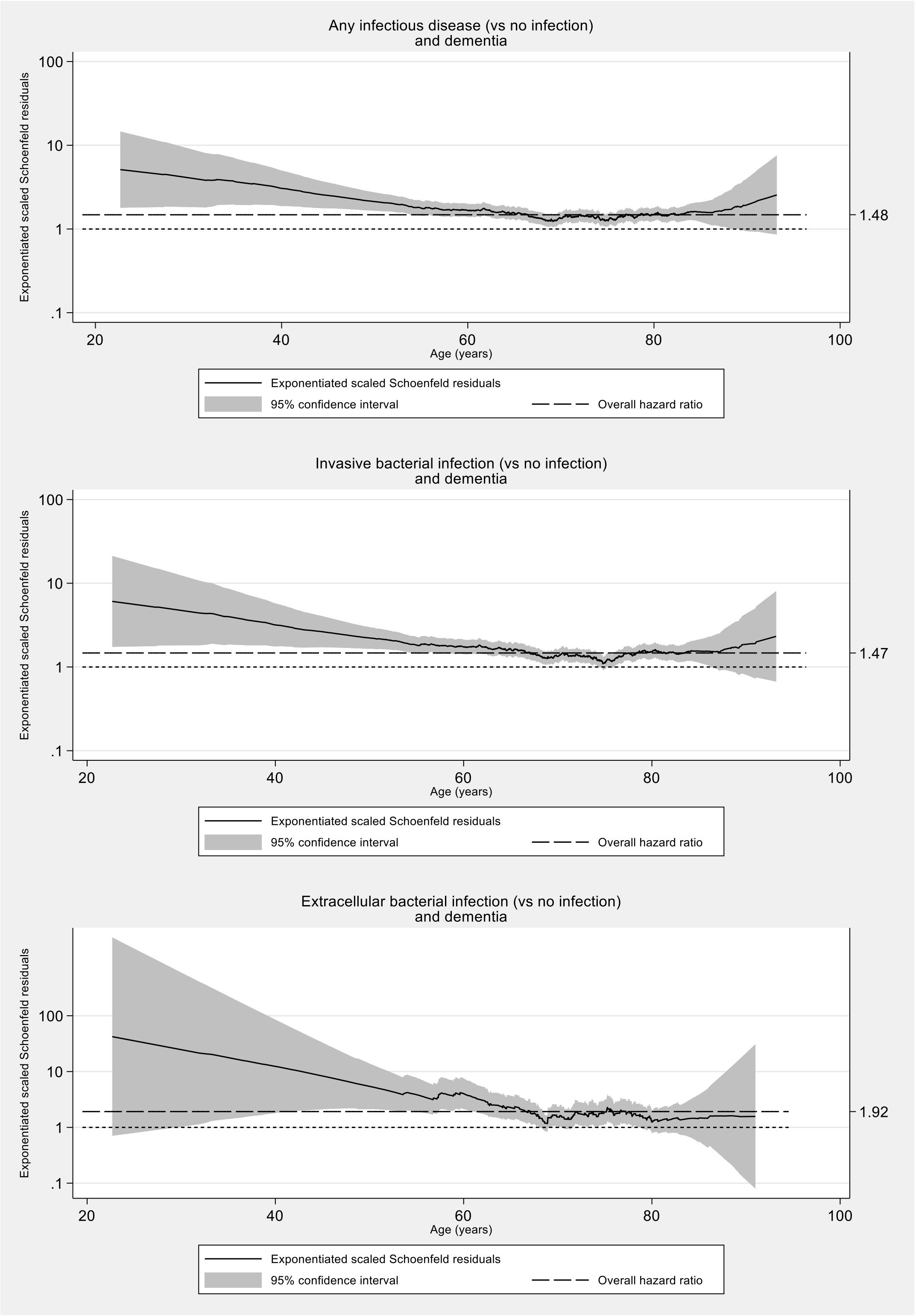

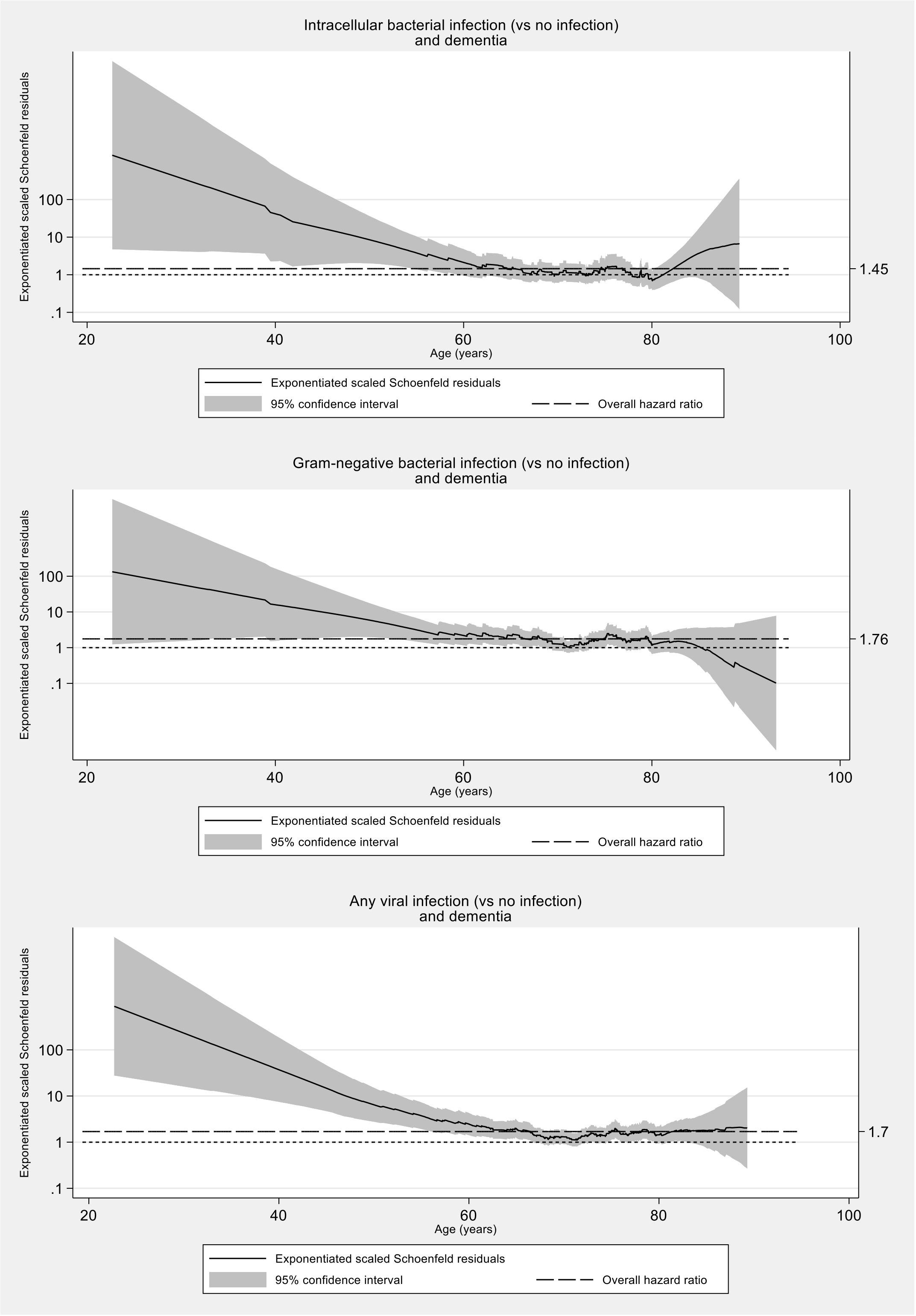
Intracellular bacterial infection (vs no infection)
and dementia
Exponentiated scaled Schoenfeld residuals
100
10
1
.1
1.45
20
40
60
80
100
Age (years)
Exponentiated scaled Schoenfeld residuals
95% confidence interval
Overall hazard ratio
Gram-negative bacterial infection (vs no infection)
and dementia
Exponentiated scaled Schoenfeld residuals
100
10
1
.1
1.76
20
40
60
80
100
Age (years)
Exponentiated scaled Schoenfeld residuals
95% confidence interval
Overall hazard ratio
Any viral infection (vs no infection)
and dementia
Exponentiated scaled Schoenfeld residuals
100
10
1
.1
1.7
20
40
60
80
100
Age (years)
Exponentiated scaled Schoenfeld residuals
95% confidence interval
Overall hazard ratio

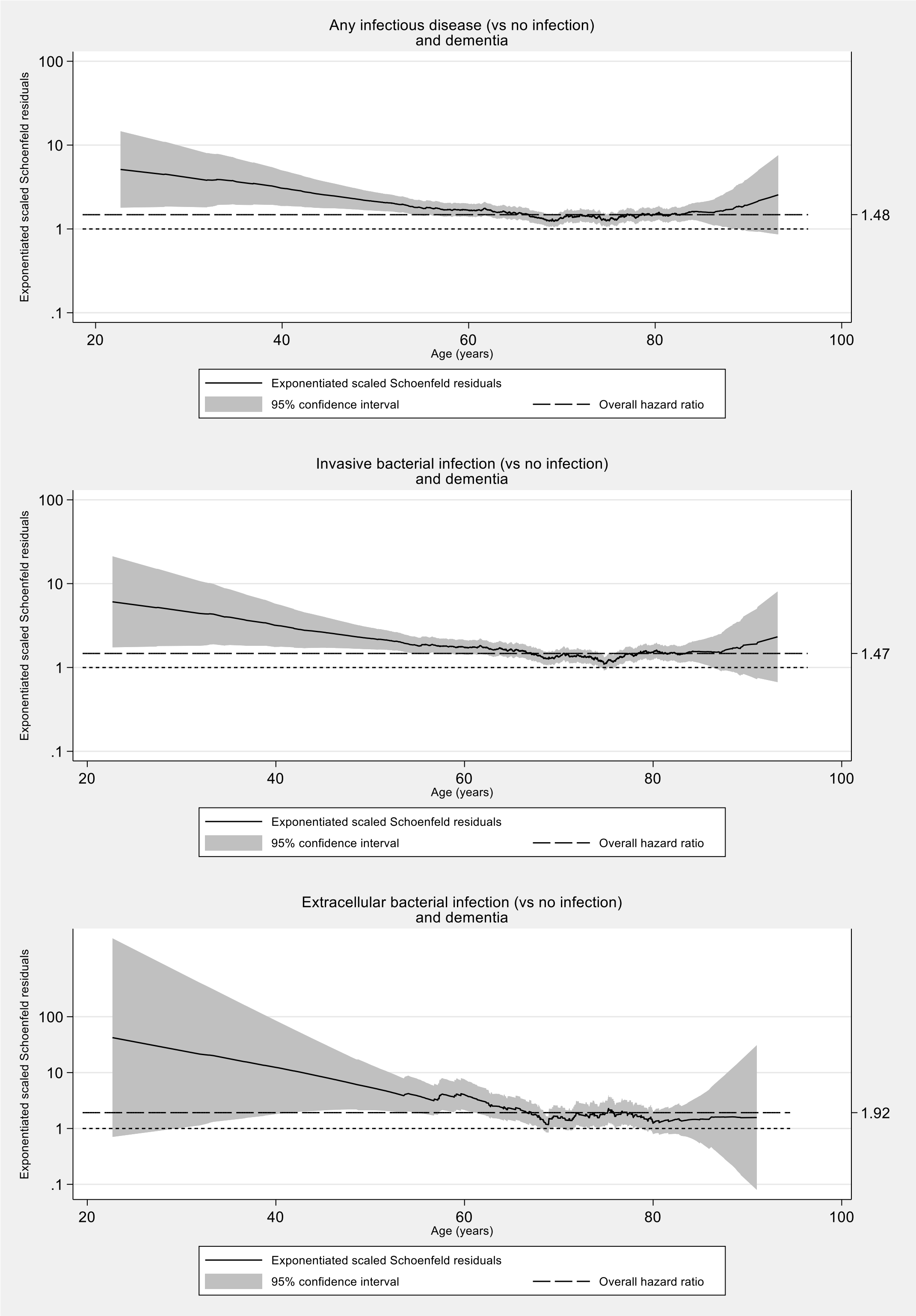

The plots are drawn using symmetric nearest-neighbor smoothing of the scaled Schoenfeld residuals, which can be interpreted as hazard ratios.[1]

eFigure 3. Distribution of age at dementia diagnosis in the primary cohort (Finnish multicohort sample)

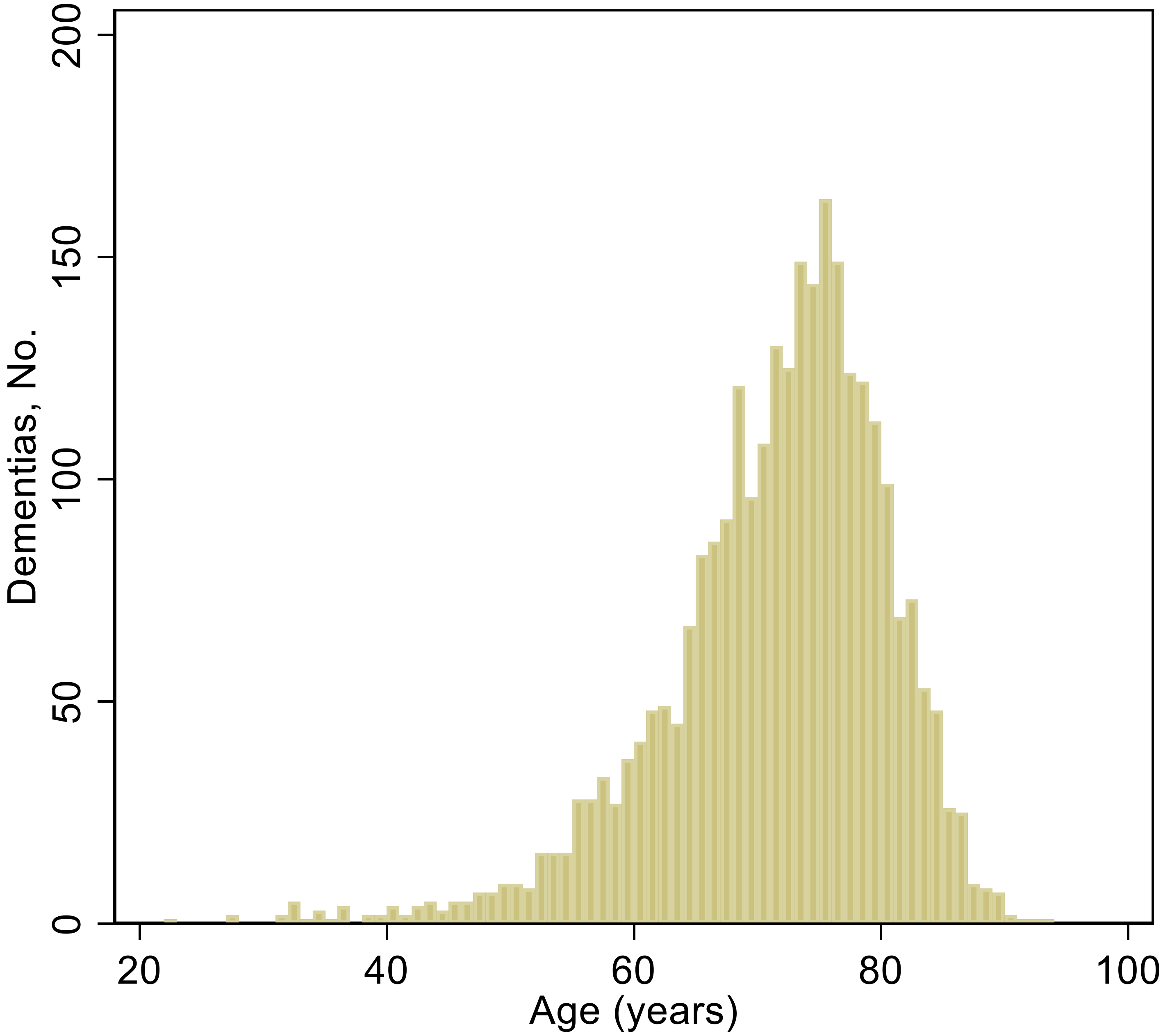

eFigure 4. Nelson-Aalen cumulative hazard estimate for the association of any hospital-treated infectious disease with dementia in the primary cohort (Finnish multicohort sample)

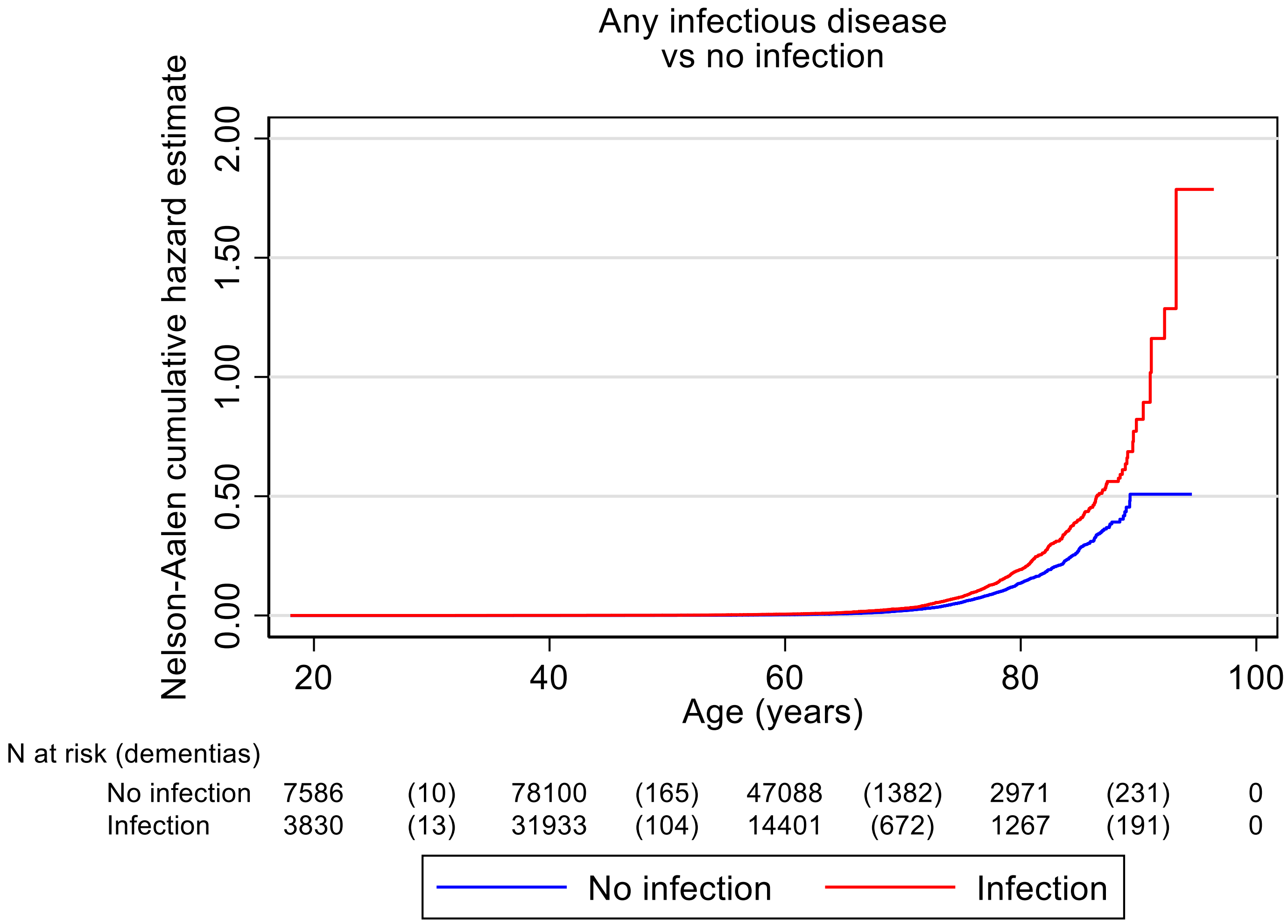

eTable 3. N (total), N (dementia), and age-standardised dementia incidence rate for the groups with vs without hospital-treated infectious diseases in the primary cohort (Finnish multicohort sample)

		Participants with infection			Reference group (no infection)		
**Infection at baseline**	**Dementia follow-up**	**Total n**	**Dementia cases**	**Age-standardised dementia incidence (per 100 000 person-years)**	**Total n**	**Dementia cases**	**Age-standardised dementia incidence (per 100 000 person-years)**
**Any infectious disease vs no infection**	**Full follow-up**	77108	980	95.35	183382	1788	62.81
**Bacterial infections**							
Any bacterial infection vs no infection		68979	908	96.97	183382	1788	62.81
**By severity**							
Potentially invasive bacterial infection vs no		50938	727	95.50	183382	1788	62.81
Localised bacterial infection vs no infection		26601	344	127.29	183382	1788	62.81
Bacterial infection with sepsis vs no infection		2920	56	123.47	183382	1788	62.81
Bacterial infection without sepsis vs no infection		68152	893	97.10	183382	1788	62.81
**By location**							
Extracellular bacterial infection vs no infection		4855	74	127.59	183382	1788	62.81
Intracellular bacterial infection vs no infection		2146	39	97.57	183382	1788	62.81
**By Gram stain**							
Gram-positive bacterial infection vs no infection		3961	58	129.83	183382	1788	62.81
Gram-negative bacterial infection vs no infection		3178	56	115.50	183382	1788	62.81
**Viral infections**							
Any viral infection vs no infection		11335	99	103.80	183382	1788	62.81
**By type of viral infection**							
Herpesvirus (persistent) infection vs no infection		2767	24	123.34	183382	1788	62.81
Other persistent viral infection vs no infection		1663	15	135.30	183382	1788	62.81
Acute viral infection vs no infection		7329	62	92.34	183382	1788	62.81
**Any infectious disease vs no infection**	**From year 10 onwards (infection at least 10 years before dementia diagnosis)**	56375	435	76.08	139891	1257	62.69
**Bacterial infections**							
Any bacterial infection vs no infection		48973	391	77.05	139891	1257	62.69
**By severity**							
Potentially invasive bacterial infection vs no		33561	317	77.69	139891	1257	62.69
Localised bacterial infection vs no infection		19791	104	78.34	139891	1257	62.69
Bacterial infection with sepsis vs no infection		826	16	124.19	139891	1257	62.69
Bacterial infection without sepsis vs no infection		48610	382	76.25	139891	1257	62.69
**By location**							
Extracellular bacterial infection vs no infection		2894	25	105.12	139891	1257	62.69
Intracellular bacterial infection vs no infection		1605	24	87.22	139891	1257	62.69
**By Gram stain**							
Gram-positive bacterial infection vs no infection		2280	15	88.18	139891	1257	62.69
Gram-negative bacterial infection vs no infection		1851	26	114.00	139891	1257	62.69
**Viral infections**							
Any viral infection vs no infection		9058	45	78.12	139891	1257	62.69
**By type of viral infection**							
Herpesvirus (persistent) infection vs no infection		2292	14	129.52	139891	1257	62.69
Other persistent viral infection vs no infection		1309	9	116.57	139891	1257	62.69
Acute viral infection vs no infection		5741	24	61.39	139891	1257	62.69

The incidence rates are standardised according to the age structure of the reference group in full follow-up.

eFigure 5. Risk of dementia associated with the most common Gram-positive and Gram-negative infections in the primary cohort (Finnish multicohort sample)

Infectious exposure	N (total)	N (exposed)	N (dementia)	Hazard ratio (95% CI)	Subgroup difference
**Gram-positive infections**					
Streptococcal infection vs no infection	185881	2499	1804	1.40 (0.85–2.29)	
Enterocolitis due to Clostridium difficile vs no infection	184052	670	1814	2.36 (1.60–3.49)	P = 0.10-0.57*
Other Gram-positive infection vs no infection	184174	792	1804	1.97 (1.20–3.23)	
**Gram-negative infections**					
Salmonella infection vs no infection	183867	485	1794	1.42 (0.63–3.16)	
Lyme disease vs no infection	183738	356	1790	NA†	P = 0.40**
Other Gram-negative infection vs no infection	185719	2337	1836	2.03 (1.52–2.71)	

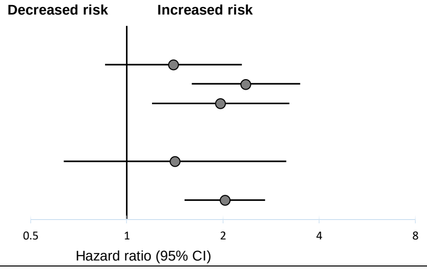

CI, confidence interval. Hazard ratios are adjusted for sex and socioeconomic status, and age is the time scale.
† The number of dementia cases in those with Lyme disease was less than 5.
* P = 0.10 for difference in the association of streptococcal and Clostridium difficile infections with dementia, 0.34 for difference in the association of streptococcal and other Gram-positive infections with dementia, and 0.57 for difference in the association of Clostridium difficile and other Gram-positive infections with dementia
** Difference in the association of salmonella vs other Gram-negative infections with dementia.
Streptococcal infections comprise ICD-10 codes A38, A40, A49.1, B95.0, B95.1, B95.2, B95.3, B95.4, B95.5, G00.1, G00.2, J02.0, J03.0, J13, J15.3, J15.4, J20.2, M00.1, M00.2, P23.3, P36.0, P36.1.
Enterocolitis due to Clostridium difficile was defined using ICD-10 code A04.7.
Other Gram-positive infections comprise all other Gram-positive infections listed in the appendix (pp. 89-91 and 241-242).
Salmonella infections comprise ICD-10 codes A01 and A02.
Lyme disease comprise ICD-10 codes A69.2 and M01.2.
Other Gram-negative infections comprise all other Gram-negative infections listed in the appendix (pp. 91-95 and 242-245).

eFigure 6. Risk of dementia associated with herpes simplex virus 1 or 2 infection, other herpesvirus infection, and any mild herpesvirus infection in the primary cohort (Finnish multicohort sample)

Infectious exposure	N (total)	N (exposed)	N (dementia)	Hazard ratio (95% CI)	Subgroup difference
Herpes simplex virus 1 or 2 infection vs no infection	184124	742	1797	2.82 (1.46–5.44)	P = 0.28*
Other herpesvirus infection vs no infection	185407	2025	1803	1.82 (1.09–3.03)	
Mild herpesvirusinfection vs no infection†	185813	2431	1809	2.33 (1.52–3.59)	

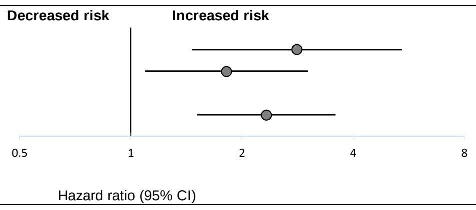

CI, confidence interval. Hazard ratios are adjusted for sex and socioeconomic status, and age is the time scale.
* Difference between herpes simplex virus infections and other herpesviral infections
† Mild herpesvirus infections comprise all herpesvirus infections except those considered severe (eczema herpeticum [B00.0], herpesviral meningitis [B00.3, B01.0, B02.1,], encephalitis [B00.4, B01.1, B02.0], and pneumonia [B01.2], disseminated herpesviral disease [B00.7, B02.7], herpesviral infection with other complications [B01.8, B02.8], Kaposi sarcoma [B21.0, C46], and congenital herpesviral infection [P35.1, P35.2]).
Herpes simplex virus 1 and 2 infections comprise ICD-10 codes A60, B00, H19.1, P35.2.
Other herpesvirus infections comprise all other herpesvirus infections listed in the appendix (pp. 159-160 and 248).

eFigure 7. Competing-risk models for the risk of dementia associated with any hospital-treated infectious disease, bacterial infections, and viral infections in the primary cohort (Finnish multicohort sample)

Infectious exposure<br>Model	N (total)	N (exposed)	N (dementia)	Hazard/sub-hazard ratio<br>(95% CI)
**Any infectious disease vs no infection**				
Cox model	260490	77108	2768	1.48 (1.37–1.60)
Fine-Gray model, competing outcome death	260490	77108	2768	1.27 (1.17–1.38)
Fine-Gray model, competing outcome death + early onset dementia	260490	77108	2226	1.19 (1.10–1.29)
**Bacterial infection vs no infection**				
Cox model	252361	68979	2696	1.50 (1.39–1.63)
Fine-Gray model, competing outcome death	252361	68979	2696	1.28 (1.18–1.40)
Fine-Gray model, competing outcome death + early onset dementia	252361	68979	2172	1.20 (1.10–1.31)
**Viral infection vs no infection**				
Cox model	194717	11335	1887	1.70 (1.39–2.08)
Fine-Gray model, competing outcome death	194717	11335	1887	1.46 (1.15–1.85)
Fine-Gray model, competing outcome death + early onset dementia	194717	11335	1510	1.20 (1.00–1.45)

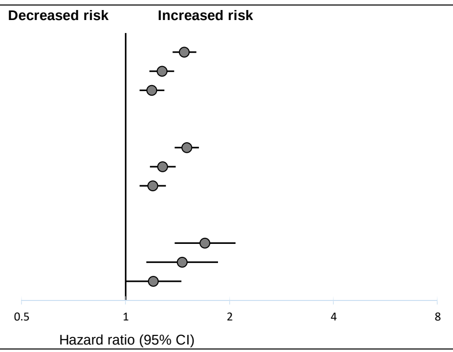

CI, confidence interval. The Cox models are adjusted for age (as the time scale), sex, and socioeconomic status and additionally stratified for cohort using cohort-specific baseline hazards and cohort-specific adjustment terms for covariates. The Fine-Gray models are adjusted for age (as the time scale), sex, socioeconomic status, and cohort and use cohort-specific adjustment terms for covariates and robust standard errors clustered for cohort.
Dementia cases that occurred before age 65 were considered early onset.

eFigure 8. Risk of Alzheimer's disease (AD) and non-AD dementia associated with any hospital-treated infectious disease and with specific bacterial and viral infections in the primary cohort (Finnish multicohort sample)

Infectious exposure	Dementia outcome	N (total)	N (exposed)	N (dementia)	Hazard ratio (95% CI)
**Any infectious disease vs no infection**	AD	260490	77108	1730	1.20 (1.08–1.33)
	Non-AD	260490	77108	1038	2.05 (1.81–2.32)
**Bacterial infections**					
Any bacterial infection vs no infection	AD	252361	68979	1687	1.21 (1.09–1.34)
	Non-AD	252361	68979	1009	2.11 (1.86–2.39)
**By severity**					
Potentially invasive bacterial infection vs no	AD	234320	50938	1586	1.17 (1.04–1.31)
	Non-AD	234320	50938	929	2.08 (1.82–2.39)
Localised bacterial infection vs no infection	AD	209983	26601	1362	1.48 (1.26–1.74)
	Non-AD	209983	26601	770	3.14 (2.65–3.72)
Bacterial infection with sepsis vs no infection	AD	186302	2920	1221	1.20 (0.82–1.76)
	Non-AD	186302	2920	623	2.80 (1.91–4.10)
Bacterial infection without sepsis vs no infection	AD	251534	68152	1683	1.22 (1.10–1.36)
	Non-AD	251534	68152	998	2.09 (1.84–2.37)
**By location**					
Extracellular bacterial infection vs no infection	AD	188237	4855	1223	1.16 (0.81–1.67)
	Non-AD	188237	4855	639	3.46 (2.55–4.72)
Intracellular bacterial infection vs no infection	AD	185528	2146	1214	1.16 (0.75–1.79)
	Non-AD	185528	2146	613	2.05 (1.28–3.28)
**By Gram stain**					
Gram-positive bacterial infection vs no infection	AD	187343	3961	1216	1.13 (0.75–1.71)
	Non-AD	187343	3961	630	3.46 (2.45–4.88)
Gram-negative bacterial infection vs no infection	AD	186560	3178	1221	1.27 (0.87–1.86)
	Non-AD	186560	3178	623	2.81 (1.92–4.10)
**Viral infections**					
Any viral infection vs no infection	AD	194717	11335	1241	1.26 (0.94–1.68)
	Non-AD	194717	11335	646	2.54 (1.90–3.38)
**By type of viral infection**					
Herpesvirus (persistent) infection vs no infection	AD	186149	2767	1203	1.33 (0.72–2.49)
	Non-AD	186149	2767	609	3.52 (2.07–5.98)
Other persistent viral infection vs no infection	AD	185045	1663	1199	1.62 (0.72–3.61)
	Non-AD	185045	1663	604	3.97 (2.05–7.68)
Acute viral infection vs no infection	AD	190711	7329	1226	1.19 (0.84–1.68)
	Non-AD	190711	7329	624	2.05 (1.41–2.98)

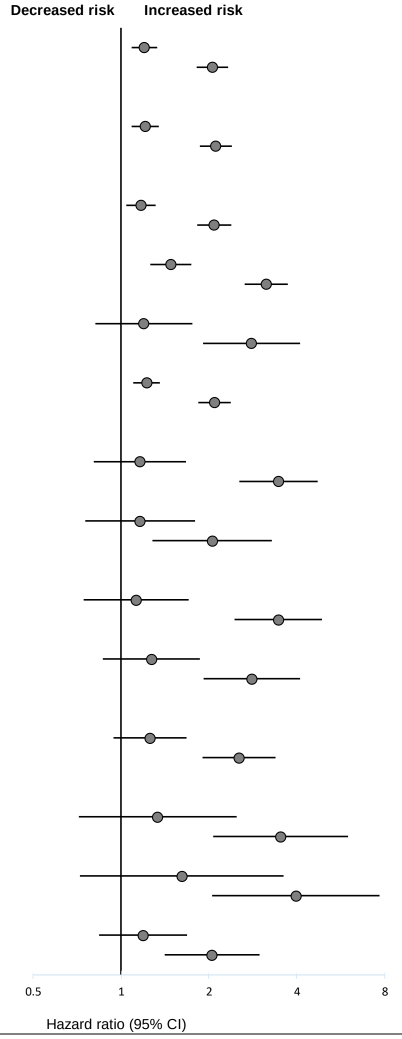

CI, confidence interval; AD, Alzheimer's disease dementia; Non-AD, other dementia. Hazard ratios are adjusted for sex and socioeconomic status, and age is the time scale.

eTable 4. Risk of dementia associated with any hospital-treated infectious disease in relation to follow ups, types of dementia, adjustments, and exclusions of participant groups in the primary and replication cohorts (Finnish multicohort sample and UK Biobank)

	Primary cohort (Finnish multicohort sample)					Replication cohort (UK Biobank)				
	Participants with infection		Reference group (no infection)		Hazard ratio	Participants with infection		Reference group (no infection)		Hazard ratio
Disease	Total n	Dementia cases	Total n	Dementia cases	(95% CI)	Total n	Dementia cases	Total n	Dementia cases	(95% CI)
**Any infectious disease (vs no infection)**										
All participants	77108	980	183382	1788	1.48 (1.37-1.60)	94112	894	391596	1238	2.60 (2.38-2.83)
Early onset dementia (onset before age 65)	71490	198	176091	344	1.76 (1.48-2.10)	58681	131	276965	159	3.70 (2.93-4.68)
Late-onset dementia (onset at or after age 65)	16303	782	43267	1444	1.42 (1.30-1.55)	59302	763	220661	1079	2.46 (2.24-2.70)
Alzheimer's disease	77108	537	183382	1193	1.20 (1.08-1.33)	94112	209	391596	418	1.80 (1.53-2.13)
Vascular dementia	77108	94	183382	115	2.09 (1.59-2.75)	94112	171	391596	181	3.28 (2.65-4.04)
Frontotemporal dementia	77108	33	183382	69	1.38 (0.91-2.09)	94112	21	391596	45	1.92 (1.14-3.24)
Parkinson's disease dementia	77108	44	183382	70	1.75 (1.20-2.56)	94112	26	391596	33	2.81 (1.67-4.72)
Other specified dementia	77108	79	183382	67	3.52 (2.53-4.90)	94112	76	391596	95	3.02 (2.23-4.09)
Unspecified dementia	77108	193	183382	274	1.94 (1.61-2.33)	94112	391	391596	466	3.00 (2.62-3.44)
Participants with hypertension excluded	70456	652	174333	1498	1.34 (1.22-1.47)	43390	254	221610	502	2.27 (1.95-2.64)
Participants with diabetes excluded	74017	856	180804	1730	1.41 (1.30-1.53)	83259	667	373525	1117	2.33 (2.11-2.56)
Participants with ischaemic heart disease excluded	75067	831	181725	1714	1.42 (1.31-1.54)	83113	684	375793	1079	2.56 (2.33-2.82)
Participants with cerebrovascular disease excluded	75813	886	182550	1753	1.42 (1.31-1.54)	90556	771	386420	1173	2.44 (2.23-2.68)
Participants with Parkinson´s disease excluded	76964	957	183301	1769	1.47 (1.35-1.59)	93501	810	390969	1169	2.51 (2.29-2.74)
Additionally adjusted for period effect	77108	980	183382	1788	1.48 (1.37-1.60)	94112	894	391596	1238	2.59 (2.37-2.82)
Data available for heavy drinking	30084	325	88979	730	1.32 (1.16-1.51)	93963	892	391292	1237	2.60 (2.38-2.83)
Additionally adjusted for heavy drinking	30084	325	88979	730	1.32 (1.16-1.51)	93963	892	391292	1237	2.51 (2.30-2.73)
Data available for smoking	29930	321	88687	729	1.31 (1.15-1.50)	93607	885	390338	1233	2.59 (2.37-2.82)
Additionally adjusted for smoking	29930	321	88687	729	1.31 (1.14-1.49)	93607	885	390338	1233	2.55 (2.33-2.78)
Data available for body mass index	--	--	--	--	--	93136	871	390038	1227	2.57 (2.35-2.80)
Additionally adjusted for body mass index	--	--	--	--	--	93136	871	390038	1227	2.58 (2.36-2.81)
Data available for heavy drinking, smoking, and body mass index	--	--	--	--	--	92531	860	388564	1221	2.55 (2.34-2.79)
Additionally adjusted for heavy drinking, smoking, and body mass index.	--	--	--	--	--	92531	860	388564	1221	2.44 (2.23-2.66)

CI, confidence interval. Hazard ratios are adjusted for sex and socioeconomic status, and additionally for period effect (year of birth), heavy drinking, smoking, or body mass index when stated so. Age is the time scale.
All hazard ratios are from the full follow-up.
Exclusions were done to those with comorbidities (hypertension, diabetes, ischaemic heart disease, cerebrovascular disease, or Parkinson's disease) at baseline.

eFigure 9. Risk of dementia associated with chronicity and predisposition of the infection towards entering the central nervous system (CNS) in the primary cohort (Finnish multicohort sample)

Infectious exposure	N (total)	N (exposed)	N (dementia)	Hazard ratio (95% CI)	Subgroup difference
**Chronicity*†**					
Acute infectious disease vs no infection	256945	73563	2734	1.50 (1.39–1.63)	P = 0.14
Chronic infectious disease vs no infection	190607	7225	1874	1.54 (1.24–1.91)	
**Predisposition to CNS infection*†**					
Infection not predisposed towards entering the CNS vs no infection	254412	71030	2699	1.49 (1.37–1.61)	P = 0.40
Infection predisposed towards entering the CNS vs no infection	191356	7974	1889	1.79 (1.46–2.19)	

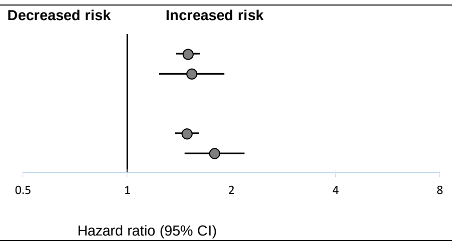

CI, confidence interval. CNS, central nervous system. Hazard ratios are adjusted for sex and socioeconomic status, and age is the time scale.
* Hazard ratios for incident dementia diagnosed at least 10 years after the infection: 1.23 (95% CI 1.10 to 1.37) for acute and 1.33 (95% CI 0.99 to 1.78) for chronic infections;
1.22 (95% CI 1.09 to 1.37) for infections not predisposed to affect the CNS and 1.33 (95% CI 0.99 to 1.78) for infections predisposed to affect the CNS.
† The three most common infections were: acute appendicitis (n = 12 007), unspecified pneumonia (n = 6204), other gastroenteritis and colitis of infectious and unspecified origin (n = 5703) for acute infections;
anogenital warts (n = 1061), gammaherpesviral mononucleosis (n = 547), chronic periodontitis (n = 469) for chronic infections;
Gammaherpesviral mononucleosis (n = 545), unspecified infectious mononucleosis (n = 425), pneumonia due to streptococcus pneumonia (n = 402) for infections predisposed to affect the CNS;
and acute appendicitis (n = 12 102), unspecified pneumonia (n = 6364), other gastroenteritis and colitis of infectious and unspecified origin (n = 5789) for infections not predisposed to affect the CNS.
The numbers reported differ slightly between categories of infections because some participants had multiple diagnoses and only the first diagnosis in each category is reported here.
A full list of the infections and their frequencies is shown in the appendix (pp. 115-152 and 268-298).

eFigure 10. Selection of participants for time-dependent analysis in the primary analysis (Finnish multicohort sample)

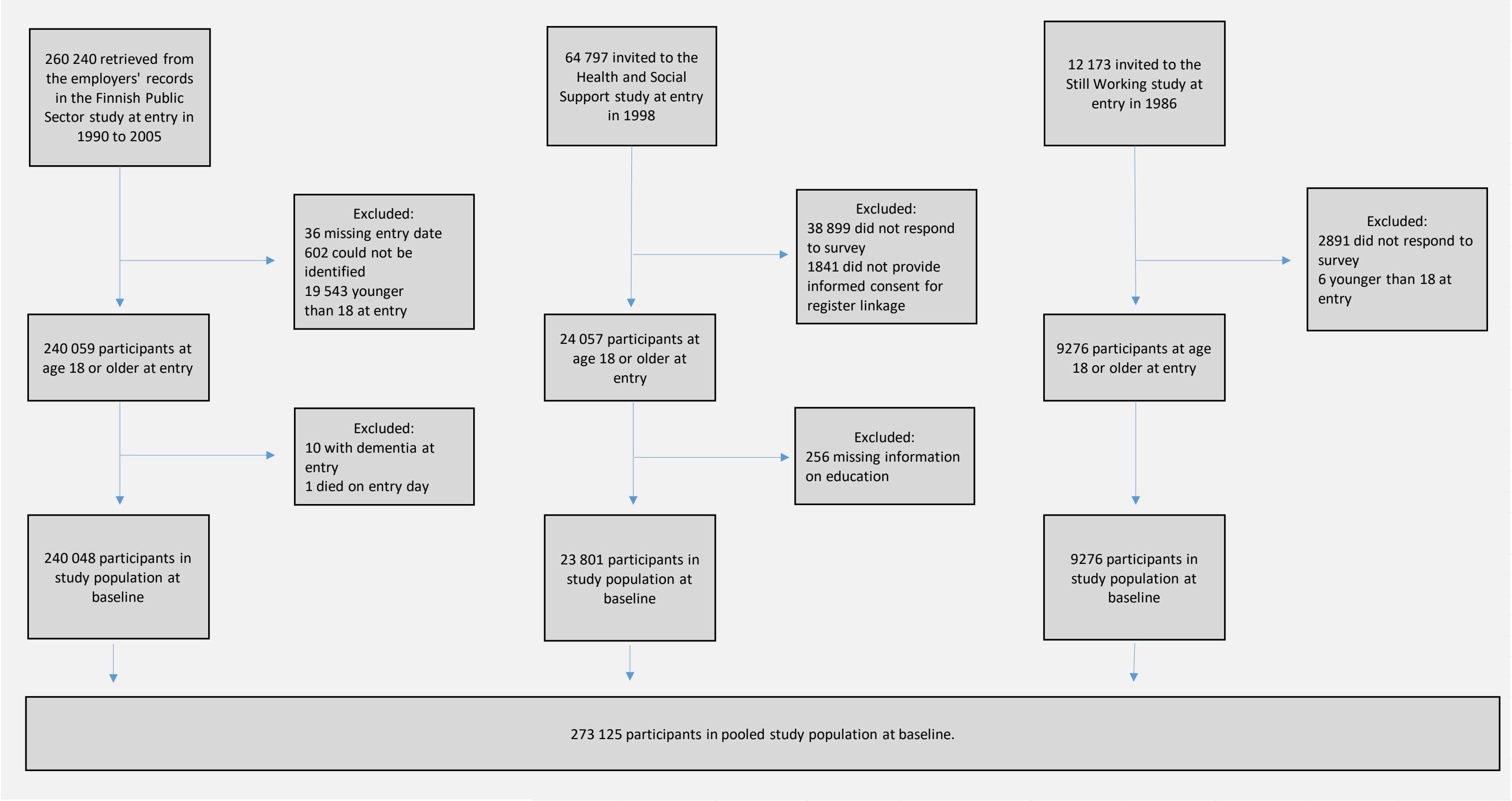

eFigure 11. Risk of dementia associated with hospital-treated infectious diseases in time-dependent analysis in the full follow up and after 10 or more years from the onset of infection in the primary cohort (Finnish multicohort sample)

Infectious exposure	N (total)	N (exposed)	N (dementia)	Hazard ratio (95% CI)	Subgroup difference
**Full follow-up**					
**Any infectious disease vs not**	273 125	77 108	3 104	1.70 (1.58–1.84)	
**Bacterial infections**					
Any bacterial infection vs not	273 125	68 979	3 104	1.71 (1.58–1.85)	P = 0.75*
**By severity**					
Potentially invasive bacterial infection vs not	273 125	50 938	3 104	1.61 (1.48–1.75)	P < 0.0001
Localised bacterial infection vs not	273 125	26 601	3 104	2.14 (1.91–2.39)	
Bacterial infection with sepsis vs not	273 125	2 920	3 104	1.68 (1.28–2.19)	P = 0.69
Bacterial infection without sepsis vs not	273 125	68 152	3 104	1.71 (1.58–1.85)	
**By location**					
Extracellular bacterial infection vs not	273 125	4 855	3 104	1.91 (1.51–2.40)	P = 0.12
Intracellular bacterial infection vs not	273 125	2 146	3 104	1.44 (1.05–1.98)	
**By Gram stain**					
Gram-positive bacterial infection vs not	273 125	3 961	3 104	1.88 (1.45–2.44)	P = 0.80
Gram-negative bacterial infection vs not	273 125	3 178	3 104	1.76 (1.34–2.29)	
**Viral infections**					
Any viral infection vs not	273 125	11 335	3 104	1.71 (1.40–2.09)	P = 0.75*
**By type of viral infection**					
Herpesvirus (persistent) infection vs not	273 125	2 767	3 104	2.08 (1.39–3.11)	
Other persistent viral infection vs not	273 125	1 663	3 104	2.51 (1.51–4.17)	
Acute viral infection vs not	273 125	7 329	3 104	1.48 (1.15–1.90)	P = 0.034†
**Follow-up from year 10 onwards**					
**Any infectious disease vs not**	265 075	56 377	2 377	1.32 (1.19–1.47)	
**Bacterial infections**					
Any bacterial infection vs not	265 218	48 975	2 402	1.32 (1.18–1.47)	P = 0.39*
**By severity**					
Potentially invasive bacterial infection vs not	265 908	33 562	2 502	1.27 (1.13–1.43)	P = 0.42
Localised bacterial infection vs not	267 369	19 792	2 650	1.34 (1.10–1.63)	
Bacterial infection with sepsis vs not	268 521	826	2 841	1.74 (1.05–2.89)	P = 0.089
Bacterial infection without sepsis vs not	265 262	48 612	2 408	1.30 (1.17–1.46)	
**By location**					
Extracellular bacterial infection vs not	268 468	2 894	2 837	1.56 (1.05–2.31)	P = 0.40
Intracellular bacterial infection vs not	268 629	1 605	2 866	1.21 (0.81–1.81)	
**By Gram stain**					
Gram-positive bacterial infection vs not	268 511	2 280	2 842	1.37 (0.83–2.28)	P = 0.51
Gram-negative bacterial infection vs not	268 540	1 851	2 851	1.68 (1.14–2.50)	
**Viral infections**					
Any viral infection vs not	268 336	9 058	2 829	1.27 (0.94–1.70)	P = 0.39*
**By type of viral infection**					
Herpesvirus (persistent) infection vs not	268 671	2 292	2 870	2.12 (1.25–3.59)	
Other persistent viral infection vs not	268 641	1 309	2 876	1.85 (0.96–3.57)	
Acute viral infection vs not	268 488	5 741	2 843	0.96 (0.64–1.43)	P = 0.0095†

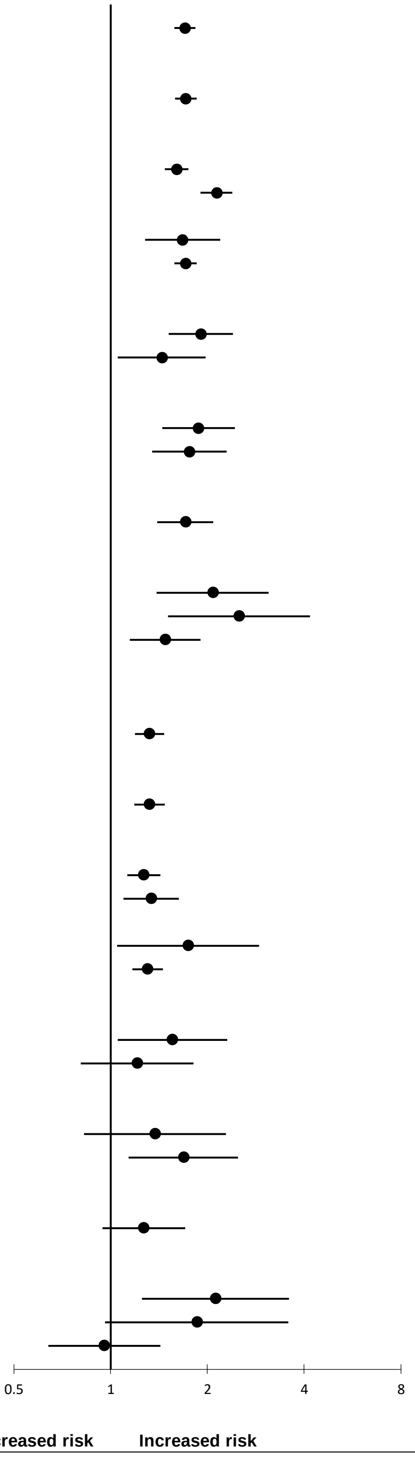

CI, confidence interval. Hazard ratios are adjusted for sex and socioeconomic status, and age is the time scale.
* Difference in the associations of bacterial and viral infections with dementia
† Difference in the association of acute viral infection and that of herpes and other persistent viral infections with dementia. (For difference in the association of herpesvirus infections and other persistent viral infections with dementia, p = 0.55 in the full follow-up and p = 0.77 from year 10 onwards.)

eFigure 12. Multivariable-adjusted associations between hospital-treatd infections and dementia by dementia type using time-dependent analysis in the replication cohort (UK Biobank)

Infectious exposure	Dementia outcome	N (total)	N (exposed)	N (dementia)	Hazard ratio (95% confidence interval)* Model 1	Model 2	Model 3	Model 4
**Any infection vs not**								
	Alzheimer's disease	492149	94112	711	2.00 (1.70–2.35)	2.00 (1.70–2.35)	1.90 (1.61–2.25)	1.94 (1.64–2.30)
	Vascular dementia	492149	94112	386	3.63 (2.96–4.44)	3.63 (2.96–4.45)	2.96 (2.40–3.66)	2.92 (2.36–3.61)
	Frontotemporal dementia	492149	94112	75	2.24 (1.35–3.73)	2.25 (1.35–3.73)	2.06 (1.22–3.48)	2.07 (1.22–3.50)
	Parkinson's disease dementia	492149	94112	66	3.13 (1.90–5.16)	3.14 (1.90–5.17)	3.03 (1.82–5.06)	3.06 (1.83–5.11)
	All-cause dementia	492149	94112	2405	2.85 (2.62–3.10)	2.85 (2.62–3.10)	2.56 (2.35–2.79)	2.59 (2.37–2.83)
**Bacterial infection vs not**								
	Alzheimer's disease	492149	80803	711	1.94 (1.64–2.30)	1.94 (1.64–2.30)	1.84 (1.55–2.19)	1.91 (1.60–2.28)
	Vascular dementia	492149	80803	386	3.72 (3.03–4.56)	3.72 (3.03–4.57)	3.08 (2.49–3.81)	3.04 (2.45–3.77)
	Frontotemporal dementia	492149	80803	75	1.54 (0.86–2.75)	1.54 (0.86–2.76)	1.35 (0.73–2.49)	1.36 (0.74–2.50)
	Parkinson's disease dementia	492149	80803	66	3.40 (2.06–5.63)	3.41 (2.06–5.63)	3.31 (1.97–5.55)	3.34 (1.99–5.60)
	All-cause dementia	492149	80803	2405	2.84 (2.61–3.10)	2.48 (2.27–2.71)	2.55 (2.33–2.78)	2.59 (2.37–2.84)
**Viral infection vs not†**								
	Alzheimer's disease	492149	9161	711	2.28 (1.49–3.48)	2.31 (1.51–3.53)	2.20 (1.44–3.36)	2.24 (1.45–3.47)
	Vascular dementia	492149	9161	386	3.42 (2.13–5.49)	3.48 (2.16–5.58)	2.75 (1.69–4.48)	2.80 (1.71–4.56)
	All-cause dementia	492149	9161	2405	2.61 (2.10–3.24)	2.51 (2.30–2.74)	2.36 (1.89–2.94)	2.35 (1.87–2.94)

Hazard ratio (95% confidence interval) from Model 1

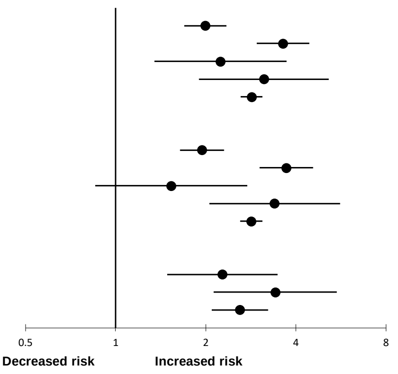

*MODEL 1: Adjusted for age (as the time scale), sex, and socioeconomic status. Numbers of participants and dementia cases are for this model.
MODEL 2: As model 1 + those with HIV excluded. This model was based on 491 894 participants with complete information of whom 2404 developed incident dementia.
MODEL 3: As model 2 + adjusted for alcohol drinking, smoking, body mass index, hypertension, diabetes. This model was based on 487 187 participants with complete information of whom 2344 developed incident dementia.
MODEL 4: As model 3 + adjusted for apolipoprotein E genotype. This model was based on 476 653 participants with complete information of whom 2284 developed incident dementia.
† The number of dementia cases in those with viral infection was less than 5 for frontotemporal dementia and Parkinson's disease dementia.

eFigure 13. Risk of dementias associated with infection burden, simultaneous infections, and central nervous system (CNS) vs extra-CNS infections using time-dependent analysis in the primary and replication cohorts (Finnish multicohort sample and UK Biobank)

Infectious exposure	N (total)	N (exposed)	N (dementia)	Hazard ratio (95% CI)	Test for trend
**Outcome: All-cause dementia (Finnish multicohort)**					
**Infection burden***					
One infection vs not	273125	51037	3104	1.36 (1.23–1.49)	
Two infections vs not	273125	15349	3104	2.22 (1.94–2.55)	p < 0.0001
Three or more infections vs not	273125	10722	3104	2.68 (2.34–3.08)	
Bacterial or viral infection (but not both) vs not	273125	70238	3104	1.66 (1.53–1.79)	p < 0.0001
Both bacterial and viral infection vs not	273125	5038	3104	2.95 (2.26–3.85)	
**Number of simultaneous infections***					
One infection diagnosis vs not	273125	74065	3104	1.68 (1.55–1.82)	p < 0.0001
Multiple simultaneous infection diagnoses vs not	273125	3043	3104	2.26 (1.71–2.98)	
**CNS vs extra-CNS infection*†**					
Extra-CNS infection vs not	273125	76301	3104	1.69 (1.56–1.82)	p = 0.0012¤
CNS infection vs not	273125	1656	3104	3.02 (2.08–4.38)	
**Outcome: All-cause dementia (UK Biobank)**					
**Infection burden††**					
One infection vs not	492149	69599	2405	2.14 (1.93–2.36)	
Two infections vs not	492149	15787	2405	4.27 (3.69–4.94)	p < 0.0001
Three or more infections vs not	492149	8726	2405	7.92 (6.75–9.30)	
Bacterial or viral infection (but not both) vs not	492149	80842	2405	2.73 (2.51–2.98)	p < 0.0001
Both bacterial and viral infection vs not	492149	4201	2405	5.21 (3.99–6.81)	
**Number of simultaneous infections**					
One infection diagnosis vs not	492149	83072	2405	2.65 (2.42–2.89)	p < 0.0001
Multiple simultaneous infection diagnoses vs not	492149	11040	2405	4.49 (3.80–5.30)	
**CNS vs extra-CNS infection**					
Extra-CNS infection vs not	492149	91400	2405	2.87 (2.64–3.12)	p = 0.15¤
CNS infection vs not	492149	887	2405	2.99 (1.61–5.57)	
**Outcome: Alzheimer's disease (UK Biobank)**					
**Infection burden††**					
One infection vs not	492149	69599	711	1.83 (1.53–2.20)	
Two infections vs not	492149	15787	711	2.46 (1.78–3.40)	P < 0.0001
Three or more infections vs not	492149	8726	711	2.81 (1.79–4.39)	
Bacterial or viral infection (but not both) vs not	492149	80842	711	1.94 (1.63–2.30)	P < 0.0001
Both bacterial and viral infection vs not	492149	4201	711	3.10 (1.71–5.64)	
**Number of simultaneous infections**					
One infection diagnosis vs not	492149	83072	711	1.93 (1.63–2.29)	P < 0.0001
Multiple simultaneous infection diagnoses vs not	492149	11040	711	2.51 (1.73–3.66)	
**CNS vs extra-CNS infection**					
Extra-CNS infection vs not	492149	91400	711	2.04 (1.73–2.40)	
CNS infection vs not	492149	887	711	NA¤¤	
**Outcome: Vascular dementia (UK Biobank)**					
**Infection burden††**					
One infection vs not	492149	69599	386	2.33 (1.81–3.00)	
Two infections vs not	492149	15787	386	6.72 (4.93–9.17)	P < 0.0001
Three or more infections vs not	492149	8726	386	11.15 (7.83–15.86)	
Bacterial or viral infection (but not both) vs not	492149	80842	386	3.47 (2.81–4.28)	P < 0.0001
Both bacterial and viral infection vs not	492149	4201	386	8.77 (5.11–15.06)	
**Number of simultaneous infections**					
One infection diagnosis vs not	492149	83072	386	3.39 (2.73–4.20)	P < 0.0001
Multiple simultaneous infection diagnoses vs not	492149	11040	386	5.54 (3.75–8.18)	
**CNS vs extra-CNS infection**					
Extra-CNS infection vs not	492149	91400	386	3.60 (2.93–4.41)	
CNS infection vs not	492149	887	386	NA¤¤	

Hazard ratio (95% confidence interval)

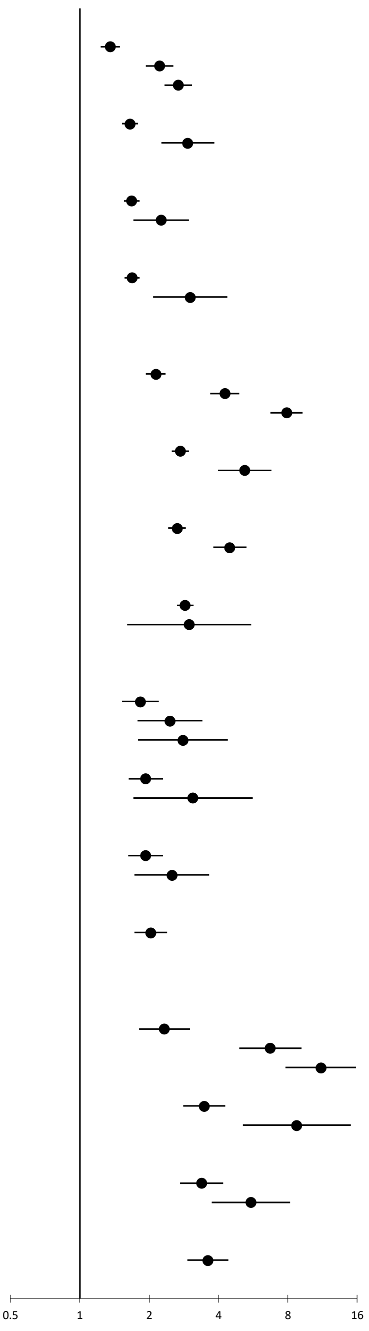

Infections are hospital-treated. CI, confidence interval. CNS, central nervous system. Hazard ratios are adjusted for sex and socioeconomic status, and age is the time scale.

* Hazard ratios for incident dementia diagnosed at least 10 years after the infection: 1.06 (95% CI 0.92-1.21) for one infection, 1.07 (95% CI 0.77 to 1.49) for two infections, and 1.81 (95% CI 1.35 to 2.43) for three or more infections; 1.30 (95% CI 1.16 to 1.44) for bacterial or viral infection (but not both) and 1.99 (95% CI 1.15 to 3.44) for both bacterial and viral infection;
1.31 (95% CI 1.18 to 1.46) for one diagnosis and 1.56 (95% CI 0.92 to 2.64) for multiple simultaneous infection diagnoses;
1.31 (95% CI 1.18 to 1.45) for extra-CNS and 1.34 (95% CI 0.67 to 2.69) for CNS infections.

† The three most common infections were: unspecified viral meningitis (n = 280), unspecified viral infection of central nervous system (n = 167), and unspecified viral encephalitis (n = 152) for CNS infections;
acute appendicitis (n = 11 903), unspecified pneumonia (n = 6088), other gastroenteritis and colitis of infectious and unspecified origin (n = 5639) for extra-CNS infections.
A full list of the infections and their frequencies is shown in the appendix (pp. 255-270).

†† If the participant was hospitalised several times for exactly the same diagnosis, only the first counted towards infection burden because the UK Biobank data included only the first hospitalisation for each diagnosis.

¤ P for subgroup difference

¤¤ Less than five Alzheimer's disease and vascular dementia cases among those exposed to CNS infections.

eTable 5. Classification of hospital-treated infectious diseases in the study on diagnosis level

ICD-10 code	ICD-10 name
**All infectious diseases**	
A00	Cholera
A00.0	Cholera due to Vibrio cholerae 01, biovar cholerae
A00.1	Cholera due to Vibrio cholerae 01, biovar eltor
A00.9	Cholera, unspecified
A01*	Typhoid and paratyphoid fevers
A02	Other salmonella infections
A02.0	Salmonella enteritis
A02.1	Salmonella sepsis
A02.2	Localized salmonella infections
A02.8	Other specified salmonella infections
A02.9	Salmonella infection, unspecified
A03	Shigellosis
A03.0	Shigellosis due to Shigella dysenteriae
A03.1	Shigellosis due to Shigella flexneri
A03.2	Shigellosis due to Shigella boydii
A03.3	Shigellosis due to Shigella sonnei
A03.8	Other shigellosis
A03.9	Shigellosis, unspecified
A04	Other bacterial intestinal infections
A04.0	Enteropathogenic Escherichia coli infection
A04.1	Enterotoxigenic Escherichia coli infection
A04.2	Enteroinvasive Escherichia coli infection
A04.3	Enterohaemorrhagic Escherichia coli infection
A04.4	Other intestinal Escherichia coli infections
A04.5	Campylobacter enteritis
A04.6	Enteritis due to Yersinia enterocolitica
A04.7	Enterocolitis due to Clostridium difficile
A04.8	Other specified bacterial intestinal infections
A04.9	Bacterial intestinal infection, unspecified
A05	Other bacterial foodborne intoxications, not elsewhere classified
A05.0	Foodborne staphylococcal intoxication
A05.1	Botulism
A05.2	Foodborne Clostridium perfringens [Clostridium welchii] intoxication

A05.3	Foodborne Vibrio parahaemolyticus intoxication
A05.4	Foodborne Bacillus cereus intoxication
A05.8	Other specified bacterial foodborne intoxications
A05.9	Bacterial foodborne intoxication, unspecified
A06	Amoebiasis
A06.0	Acute amoebic dysentery
A06.1	Chronic intestinal amoebiasis
A06.2	Amoebic nondysenteric colitis
A06.3	Amoeboma of intestine
A06.4	Amoebic liver abscess
A06.5	Amoebic lung abscess
A06.6	Amoebic brain abscess
A06.7	Cutaneous amoebiasis
A06.8	Amoebic infection of other sites
A06.9	Amoebiasis, unspecified
A07*	Other protozoal intestinal diseases
A08.0	Rotaviral enteritis
A08.1	Acute gastroenteropathy due to Norwalk agent
A08.2	Adenoviral enteritis
A08.3	Other viral enteritis
A08.4	Viral intestinal infection, unspecified
A08.5	Other specified intestinal infections
A09*	Other gastroenteritis and colitis of infectious and unspecified origin
A15*	Respiratory tuberculosis, bacteriologically and histologically confirmed
A16*	Respiratory tuberculosis, not confirmed bacteriologically or histologically
A17	Tuberculosis of nervous system
A17.0	Tuberculous meningitis
A17.1	Meningeal tuberculoma
A17.8	Other tuberculosis of nervous system
A17.9	Tuberculosis of nervous system, unspecified
A18*	Tuberculosis of other organs
A19*	Miliary tuberculosis
A20	Plague
A20.0	Bubonic plague
A20.1	Cellulocutaneous plague
A20.2	Pneumonic plague
A20.3	Plague meningitis
A20.7	Septicaemic plague

A20.8	Other forms of plague
A20.9	Plague, unspecified
A21	Tularaemia
A21.0	Ulceroglandular tularaemia
A21.1	Oculoglandular tularaemia
A21.2	Pulmonary tularaemia
A21.3	Gastrointestinal tularaemia
A21.7	Generalized tularaemia
A21.8	Other forms of tularaemia
A21.9	Tularaemia, unspecified
A22	Anthrax
A22.0	Cutaneous anthrax
A22.1	Pulmonary anthrax
A22.2	Gastrointestinal anthrax
A22.7	Anthrax sepsis
A22.8	Other forms of anthrax
A22.9	Anthrax, unspecified
A23	Brucellosis
A23.0	Brucellosis due to Brucella melitensis
A23.1	Brucellosis due to Brucella abortus
A23.2	Brucellosis due to Brucella suis
A23.3	Brucellosis due to Brucella canis
A23.8	Other brucellosis
A23.9	Brucellosis, unspecified
A24	Glanders and melioidosis
A24.0	Glanders
A24.1	Acute and fulminating melioidosis
A24.2	Subacute and chronic melioidosis
A24.3	Other melioidosis
A25	Rat-bite fevers
A25.0	Spirillosis
A25.1	Streptobacillosis
A25.9	Rat-bite fever, unspecified
A26	Erysipeloid
A26.0	Cutaneous erysipeloid
A26.7	Erysipelothrix sepsis
A26.8	Other forms of erysipeloid
A26.9	Erysipeloid, unspecified

A27*	Leptospirosis
A28	Other zoonotic bacterial diseases, not elsewhere classified
A28.0	Pasteurellosis
A28.1	Cat-scratch disease
A28.2	Extraintestinal yersiniosis
A28.8	Other specified zoonotic bacterial diseases, not elsewhere classified
A28.9	Zoonotic bacterial disease, unspecified
A30*	Leprosy [Hansen disease]
A31*	Infection due to other mycobacteria
A32	Listeriosis
A32.0	Cutaneous listeriosis
A32.1	Listerial meningitis and meningoencephalitis
A32.7	Listerial sepsis
A32.8	Other forms of listeriosis
A32.9	Listeriosis, unspecified
A33*	Tetanus neonatorum
A34*	Obstetrical tetanus
A35*	Other tetanus
A36*	Diphtheria
A37*	Whooping cough
A38*	Scarlet fever
A39	Meningococcal infection
A39.0	Meningococcal meningitis
A39.1	Waterhouse-Friderichsen syndrome
A39.2	Acute meningococcaemia
A39.3	Chronic meningococcaemia
A39.4	Meningococcaemia, unspecified
A39.5	Meningococcal heart disease
A39.8	Other meningococcal infections
A39.9	Meningococcal infection, unspecified
A40	Streptococcal sepsis
A40.0	Sepsis due to streptococcus, group A
A40.1	Sepsis due to streptococcus, group B
A40.2	Sepsis due to streptococcus, group D
A40.3	Sepsis due to Streptococcus pneumoniae
A40.8	Other streptococcal sepsis
A40.9	Streptococcal sepsis, unspecified
A41	Other sepsis

A41.0	Sepsis due to Staphylococcus aureus
A41.1	Sepsis due to other specified staphylococcus
A41.2	Sepsis due to unspecified staphylococcus
A41.3	Sepsis due to Haemophilus influenzae
A41.4	Sepsis due to anaerobes
A41.5	Sepsis due to other Gram-negative organisms
A41.8	Other specified sepsis
A41.9	Sepsis, unspecified
A42	Actinomycosis
A42.0	Pulmonary actinomycosis
A42.1	Abdominal actinomycosis
A42.2	Cervicofacial actinomycosis
A42.7	Actinomycotic sepsis
A42.8	Other forms of actinomycosis
A42.9	Actinomycosis, unspecified
A43	Nocardiosis
A43.0	Pulmonary nocardiosis
A43.1	Cutaneous nocardiosis
A43.8	Other forms of nocardiosis
A44	Bartonellosis
A44.0	Systemic bartonellosis
A44.1	Cutaneous and mucocutaneous bartonellosis
A44.8	Other forms of bartonellosis
A44.9	Bartonellosis, unspecified
A46*	Erysipelas
A48.0	Gas gangrene
A48.1	Legionnaires disease
A48.2	Nonpneumonic Legionnaires disease [Pontiac fever]
A48.3	Toxic shock syndrome
A48.4	Brazilian purpuric fever
A48.8	Other specified bacterial diseases
A49	Bacterial infection of unspecified site
A49.0	Staphylococcal infection, unspecified site
A49.1	Streptococcal infection, unspecified site
A49.2	Haemophilus influenzae infection, unspecified site
A49.3	Mycoplasma infection, unspecified site
A49.8	Other bacterial infections of unspecified site
A49.9	Bacterial infection, unspecified

A50	Congenital syphilis
A50.0	Early congenital syphilis, symptomatic
A50.1	Early congenital syphilis, latent
A50.2	Early congenital syphilis, unspecified
A50.3	Late congenital syphilitic oculopathy
A50.4	Late congenital neurosyphilis [juvenile neurosyphilis]
A50.5	Other late congenital syphilis, symptomatic
A50.6	Late congenital syphilis, latent
A50.7	Late congenital syphilis, unspecified
A50.9	Congenital syphilis, unspecified
A51	Early syphilis
A51.0	Primary genital syphilis
A51.1	Primary anal syphilis
A51.2	Primary syphilis of other sites
A51.3	Secondary syphilis of skin and mucous membranes
A51.4	Other secondary syphilis
A51.5	Early syphilis, latent
A51.9	Early syphilis, unspecified
A52	Late syphilis
A52.0	Cardiovascular syphilis
A52.1	Symptomatic neurosyphilis
A52.2	Asymptomatic neurosyphilis
A52.3	Neurosyphilis, unspecified
A52.7	Other symptomatic late syphilis
A52.8	Late syphilis, latent
A52.9	Late syphilis, unspecified
A53*	Other and unspecified syphilis
A54	Gonococcal infection
A54.0	Gonococcal infection of lower genitourinary tract without periurethral or accessory gland abscess
A54.1	Gonococcal infection of lower genitourinary tract with periurethral and accessory gland abscess
A54.2	Gonococcal pelviperitonitis and other gonococcal genitourinary infections
A54.3	Gonococcal infection of eye
A54.4	Gonococcal infection of musculoskeletal system
A54.5	Gonococcal pharyngitis
A54.6	Gonococcal infection of anus and rectum
A54.8	Other gonococcal infections
A54.9	Gonococcal infection, unspecified
A55*	Chlamydial lymphogranuloma (venereum)

A56.0	Chlamydial infection of lower genitourinary tract
A56.1	Chlamydial infection of pelviperitoneum and other genitourinary organs
A56.2	Chlamydial infection of genitourinary tract, unspecified
A56.3	Chlamydial infection of anus and rectum
A56.4	Chlamydial infection of pharynx
A56.8	Sexually transmitted chlamydial infection of other sites
A57*	Chancroid
A58*	Granuloma inguinale
A59*	Trichomoniasis
A60*	Anogenital herpesviral [herpes simplex] infection
A63.0	Anogenital (venereal) warts
A63.8	Other specified predominantly sexually transmitted diseases
A64*	Unspecified sexually transmitted disease
A65*	Nonvenereal syphilis
A66*	Yaws
A67*	Pinta [carate]
A68*	Relapsing fevers
A69	Other spirochaetal infections
A69.0	Necrotizing ulcerative stomatitis
A69.1	Other Vincent infections
A69.2	Lyme disease
A69.8	Other specified spirochaetal infections
A69.9	Spirochaetal infection, unspecified
A70*	Chlamydia psittaci infection
A71*	Trachoma
A74.0	Chlamydial conjunctivitis
A74.8	Other chlamydial diseases
A74.9	Chlamydial infection, unspecified
A75	Typhus fever
A75.0	Epidemic louse-borne typhus fever due to Rickettsia prowazekii
A75.1	Recrudescent typhus [Brill disease]
A75.2	Typhus fever due to Rickettsia typhi
A75.3	Typhus fever due to Rickettsia tsutsugamushi
A75.9	Typhus fever, unspecified
A77	Spotted fever [tick-borne rickettsioses]
A77.0	Spotted fever due to Rickettsia rickettsii
A77.1	Spotted fever due to Rickettsia conorii
A77.2	Spotted fever due to Rickettsia sibirica

A77.3	Spotted fever due to Rickettsia australis
A77.8	Other spotted fevers
A77.9	Spotted fever, unspecified
A78*	Q fever
A79	Other rickettsioses
A79.0	Trench fever
A79.1	Rickettsialpox due to Rickettsia akari
A79.8	Other specified rickettsioses
A79.9	Rickettsiosis, unspecified
A80*	Acute poliomyelitis
A81.1	Subacute sclerosing panencephalitis
A81.2	Progressive multifocal leukoencephalopathy
A83*	Mosquito-borne viral encephalitis
A84*	Tick-borne viral encephalitis
A85*	Other viral encephalitis, not elsewhere classified
A86*	Unspecified viral encephalitis
A87*	Viral meningitis
A88*	Other viral infections of central nervous system, not elsewhere classified
A89*	Unspecified viral infection of central nervous system
A90*	Dengue fever [classical dengue]
A91*	Dengue haemorrhagic fever
A92	Other mosquito-borne viral fevers
A92.0	Chikungunya virus disease
A92.1	O'nyong-nyong fever
A92.2	Venezuelan equine fever
A92.3	West Nile virus infection
A92.4	Rift Valley fever
A92.8	Other specified mosquito-borne viral fevers
A92.9	Mosquito-borne viral fever, unspecified
A93	Other arthropod-borne viral fevers, not elsewhere classified
A93.0	Oropouche virus disease
A93.1	Sandfly fever
A93.2	Colorado tick fever
A93.8	Other specified arthropod-borne viral fevers
A94*	Unspecified arthropod-borne viral fever
A95*	Yellow fever
A96*	Arenaviral haemorrhagic fever
A97*	Dengue

A98	Other viral haemorrhagic fevers, not elsewhere classified
A98.0	Crimean-Congo haemorrhagic fever
A98.1	Omsk haemorrhagic fever
A98.2	Kyasanur Forest disease
A98.3	Marburg virus disease
A98.4	Ebola virus disease
A98.5	Haemorrhagic fever with renal syndrome
A98.8	Other specified viral haemorrhagic fevers
A99*	Unspecified viral haemorrhagic fever
B00	Herpesviral [herpes simplex] infections
B00.0	Eczema herpeticum
B00.1	Herpesviral vesicular dermatitis
B00.2	Herpesviral gingivostomatitis and pharyngotonsillitis
B00.3	Herpesviral meningitis
B00.4	Herpesviral encephalitis
B00.5	Herpesviral ocular disease
B00.7	Disseminated herpesviral disease
B00.8	Other forms of herpesviral infection
B00.9	Herpesviral infection, unspecified
B01	Varicella [chickenpox]
B01.0	Varicella meningitis
B01.1	Varicella encephalitis
B01.2	Varicella pneumonia
B01.8	Varicella with other complications
B01.9	Varicella without complication
B02	Zoster [herpes zoster]
B02.0	Zoster encephalitis
B02.1	Zoster meningitis
B02.2	Zoster with other nervous system involvement
B02.3	Zoster ocular disease
B02.7	Disseminated zoster
B02.8	Zoster with other complications
B02.9	Zoster without complication
B04*	Monkeypox
B05	Measles
B05.0	Measles complicated by encephalitis
B05.1	Measles complicated by meningitis
B05.2	Measles complicated by pneumonia

B05.3	Measles complicated by otitis media
B05.4	Measles with intestinal complications
B05.8	Measles with other complications
B05.9	Measles without complication
B06	Rubella [German measles]
B06.0	Rubella with neurological complications
B06.8	Rubella with other complications
B06.9	Rubella without complication
B07*	Viral warts
B08.0	Other orthopoxvirus infections
B08.1	Molluscum contagiosum
B08.2	Exanthema subitum [sixth disease]
B08.3	Erythema infectiosum [fifth disease]
B08.4	Enteroviral vesicular stomatitis with exanthem
B08.5	Enteroviral vesicular pharyngitis
B08.8	Other specified viral infections characterized by skin and mucous membrane lesions
B09*	Unspecified viral infection characterized by skin and mucous membrane lesions
B15	Acute hepatitis A
B15.0	Hepatitis A with hepatic coma
B15.9	Hepatitis A without hepatic coma
B16	Acute hepatitis B
B16.0	Acute hepatitis B with delta-agent (coinfection) with hepatic coma
B16.1	Acute hepatitis B with delta-agent (coinfection) without hepatic coma
B16.2	Acute hepatitis B without delta-agent with hepatic coma
B16.9	Acute hepatitis B without delta-agent and without hepatic coma
B17*	Other acute viral hepatitis
B18*	Chronic viral hepatitis
B19	Unspecified viral hepatitis
B19.0	Unspecified viral hepatitis with hepatic coma
B19.9	Unspecified viral hepatitis without hepatic coma
B20*	Human immunodeficiency virus [HIV] disease resulting in infectious and parasitic diseases
B21*	Human immunodeficiency virus [HIV] disease resulting in malignant neoplasms
B21.0	HIV disease resulting in Kaposi sarcoma
B22	Human immunodeficiency virus [HIV] disease resulting in other specified diseases
B22.0	HIV disease resulting in encephalopathy
B22.1	HIV disease resulting in lymphoid interstitial pneumonitis
B22.2	HIV disease resulting in wasting syndrome
B22.7	HIV disease resulting in multiple diseases classified elsewhere

B23*	Human immunodeficiency virus [HIV] disease resulting in other conditions
B24*	Unspecified human immunodeficiency virus [HIV] disease
B25*	Cytomegaloviral disease
B26	Mumps
B26.0	Mumps orchitis
B26.1	Mumps meningitis
B26.2	Mumps encephalitis
B26.3	Mumps pancreatitis
B26.8	Mumps with other complications
B26.9	Mumps without complication
B27	Infectious mononucleosis
B27.0	Gammaherpesviral mononucleosis
B27.1	Cytomegaloviral mononucleosis
B27.8	Other infectious mononucleosis
B27.9	Infectious mononucleosis, unspecified
B30*	Viral conjunctivitis
B33	Other viral diseases, not elsewhere classified
B33.0	Epidemic myalgia
B33.1	Ross River disease
B33.2	Viral carditis
B33.3	Retrovirus infections, not elsewhere classified
B33.4	Hantavirus (cardio-)pulmonary syndrome [HPS] [HCPS]
B33.8	Other specified viral diseases
B34	Viral infection of unspecified site
B34.0	Adenovirus infection, unspecified site
B34.1	Enterovirus infection, unspecified site
B34.2	Coronavirus infection, unspecified site
B34.3	Parvovirus infection, unspecified site
B34.4	Papovavirus infection, unspecified site
B34.8	Other viral infections of unspecified site
B34.9	Viral infection, unspecified
B35*	Dermatophytosis
B36*	Other superficial mycoses
B37	Candidiasis
B37.0	Candidal stomatitis
B37.1	Pulmonary candidiasis
B37.2	Candidiasis of skin and nail
B37.3	Candidiasis of vulva and vagina

B37.4	Candidiasis of other urogenital sites
B37.5	Candidal meningitis
B37.6	Candidal endocarditis
B37.7	Candidal sepsis
B37.8	Candidiasis of other sites
B37.9	Candidiasis, unspecified
B38	Coccidioidomycosis
B38.0	Acute pulmonary coccidioidomycosis
B38.1	Chronic pulmonary coccidioidomycosis
B38.2	Pulmonary coccidioidomycosis, unspecified
B38.3	Cutaneous coccidioidomycosis
B38.4	Coccidioidomycosis meningitis
B38.7	Disseminated coccidioidomycosis
B38.8	Other forms of coccidioidomycosis
B38.9	Coccidioidomycosis, unspecified
B39	Histoplasmosis
B39.0	Acute pulmonary histoplasmosis capsulati
B39.1	Chronic pulmonary histoplasmosis capsulati
B39.2	Pulmonary histoplasmosis capsulati, unspecified
B39.3	Disseminated histoplasmosis capsulati
B39.4	Histoplasmosis capsulati, unspecified
B39.5	Histoplasmosis duboisii
B39.9	Histoplasmosis, unspecified
B40	Blastomycosis
B40.0	Acute pulmonary blastomycosis
B40.1	Chronic pulmonary blastomycosis
B40.2	Pulmonary blastomycosis, unspecified
B40.3	Cutaneous blastomycosis
B40.7	Disseminated blastomycosis
B40.8	Other forms of blastomycosis
B40.9	Blastomycosis, unspecified
B41	Paracoccidioidomycosis
B41.0	Pulmonary paracoccidioidomycosis
B41.7	Disseminated paracoccidioidomycosis
B41.8	Other forms of paracoccidioidomycosis
B41.9	Paracoccidioidomycosis, unspecified
B42	Sporotrichosis
B42.0	Pulmonary sporotrichosis

B42.1	Lymphocutaneous sporotrichosis
B42.7	Disseminated sporotrichosis
B42.8	Other forms of sporotrichosis
B42.9	Sporotrichosis, unspecified
B43	Chromomycosis and phaeomycotic abscess
B43.0	Cutaneous chromomycosis
B43.1	Phaeomycotic brain abscess
B43.2	Subcutaneous phaeomycotic abscess and cyst
B43.8	Other forms of chromomycosis
B43.9	Chromomycosis, unspecified
B44*	Aspergillosis
B45	Cryptococcosis
B45.0	Pulmonary cryptococcosis
B45.1	Cerebral cryptococcosis
B45.2	Cutaneous cryptococcosis
B45.3	Osseous cryptococcosis
B45.7	Disseminated cryptococcosis
B45.8	Other forms of cryptococcosis
B45.9	Cryptococcosis, unspecified
B46	Zygomycosis
B46.0	Pulmonary mucormycosis
B46.1	Rhinocerebral mucormycosis
B46.2	Gastrointestinal mucormycosis
B46.3	Cutaneous mucormycosis
B46.4	Disseminated mucormycosis
B46.5	Mucormycosis, unspecified
B46.8	Other zygomycoses
B46.9	Zygomycosis, unspecified
B47	Mycetoma
B47.0	Eumycetoma
B47.1	Actinomycetoma
B47.9	Mycetoma, unspecified
B48	Other mycoses, not elsewhere classified
B48.0	Lobomycosis
B48.1	Rhinosporidiosis
B48.2	Allescheriasis
B48.3	Geotrichosis
B48.4	Penicillosis

B48.7	Opportunistic mycoses
B48.8	Other specified mycoses
B49*	Unspecified mycosis
B50*	Plasmodium falciparum malaria
B50.0	Plasmodium falciparum malaria with cerebral complications
B50.8	Other severe and complicated Plasmodium falciparum malaria
B50.9	Plasmodium falciparum malaria, unspecified
B51*	Plasmodium vivax malaria
B52*	Plasmodium malariae malaria
B53	Other parasitologically confirmed malaria
B53.0	Plasmodium ovale malaria
B53.1	Malaria due to simian plasmodia
B53.8	Other parasitologically confirmed malaria, not elsewhere classified
B54*	Unspecified malaria
B55.0	Visceral leishmaniasis
B55.1	Cutaneous leishmaniasis
B55.2	Mucocutaneous leishmaniasis
B55.9	Leishmaniasis, unspecified
B56*	African trypanosomiasis
B57*	Chagas disease
B58	Toxoplasmosis
B58.0	Toxoplasma oculopathy
B58.1	Toxoplasma hepatitis
B58.2	Toxoplasma meningoencephalitis
B58.3	Pulmonary toxoplasmosis
B58.8	Toxoplasmosis with other organ involvement
B58.9	Toxoplasmosis, unspecified
B59*	Pneumocystosis
B60.0	Babesiosis
B60.1	Acanthamoebiasis
B60.2	Naegleriasis
B60.8	Other specified protozoal diseases
B64*	Unspecified protozoal disease
B65	Schistosomiasis [bilharziasis]
B65.0	Schistosomiasis due to Schistosoma haematobium [urinary schistosomiasis]
B65.1	Schistosomiasis due to Schistosoma mansoni [intestinal schistosomiasis]
B65.2	Schistosomiasis due to Schistosoma japonicum
B65.3	Cercarial dermatitis

B65.8	Other schistosomiases
B65.9	Schistosomiasis, unspecified
B66	Other fluke infections
B66.0	Opisthorchiasis
B66.1	Clonorchiasis
B66.2	Dicrocoeliasis
B66.3	Fascioliasis
B66.4	Paragonimiasis
B66.5	Fasciolopsiasis
B66.8	Other specified fluke infections
B66.9	Fluke infection, unspecified
B67*	Echinococcosis
B68	Taeniasis
B68.0	Taenia solium taeniasis
B68.1	Taenia saginata taeniasis
B68.9	Taeniasis, unspecified
B69	Cysticercosis
B69.0	Cysticercosis of central nervous system
B69.1	Cysticercosis of eye
B69.8	Cysticercosis of other sites
B69.9	Cysticercosis, unspecified
B70	Diphyllobothriasis and sparganosis
B70.0	Diphyllobothriasis
B70.1	Sparganosis
B71	Other cestode infections
B71.0	Hymenolepiasis
B71.1	Dipylidiasis
B71.8	Other specified cestode infections
B71.9	Cestode infection, unspecified
B72*	Dracunculiasis
B73*	Onchocerciasis
B74*	Filariasis
B75*	Trichinellosis
B76*	Hookworm diseases
B77*	Ascariasis
B78*	Strongyloidiasis
B79*	Trichuriasis
B80*	Enterobiasis

B81	Other intestinal helminthiases, not elsewhere classified
B81.0	Anisakiasis
B81.1	Intestinal capillariasis
B81.2	Trichostrongyliasis
B81.3	Intestinal angiostrongyliasis
B81.4	Mixed intestinal helminthiases
B81.8	Other specified intestinal helminthiases
B82	Unspecified intestinal parasitism
B82.0	Intestinal helminthiasis, unspecified
B82.9	Intestinal parasitism, unspecified
B83	Other helminthiases
B83.0	Visceral larva migrans
B83.1	Gnathostomiasis
B83.2	Angiostrongyliasis due to Parastrongylus cantonensis
B83.3	Syngamiasis
B83.4	Internal hirudiniasis
B83.8	Other specified helminthiases
B83.9	Helminthiasis, unspecified
B85*	Pediculosis and phthiriasis
B86*	Scabies
B87*	Myiasis
B88	Other infestations
B88.0	Other acariasis
B88.1	Tungiasis [sandflea infestation]
B88.2	Other arthropod infestations
B88.3	External hirudiniasis
B88.8	Other specified infestations
B88.9	Infestation, unspecified
B89*	Unspecified parasitic disease
B95*	Streptococcus and staphylococcus as the cause of diseases classified to other chapters
B96.0	Mycoplasma pneumoniae [M. pneumoniae] as the cause of diseases classified to other chapters
B96.1	Klebsiella pneumoniae [K. pneumoniae] as the cause of diseases classified to other chapters
B96.2	Escherichia coli [E. coli] as the cause of diseases classified to other chapters
B96.3	Haemophilus influenzae [H. influenzae] as the cause of diseases classified to other chapters
B96.4	Proteus (mirabilis)(morganii) as the cause of diseases classified to other chapters
B96.5	Pseudomonas (aeruginosa) as the cause of diseases classified to other chapters
B96.6	Bacillus fragilis [B. fragilis] as the cause of diseases classified to other chapters
B96.7	Clostridium perfringens [C. perfringens] as the cause of diseases classified to other chapters

B96.8	Other specified bacterial agents as the cause of diseases classified to other chapters
B97	Viral agents as the cause of diseases classified to other chapters
B97.0	Adenovirus as the cause of diseases classified to other chapters
B97.1	Enterovirus as the cause of diseases classified to other chapters
B97.2	Coronavirus as the cause of diseases classified to other chapters
B97.3	Retrovirus as the cause of diseases classified to other chapters
B97.4	Respiratory syncytial virus as the cause of diseases classified to other chapters
B97.5	Reovirus as the cause of diseases classified to other chapters
B97.6	Parvovirus as the cause of diseases classified to other chapters
B97.7	Papillomavirus as the cause of diseases classified to other chapters
B97.8	Other viral agents as the cause of diseases classified to other chapters
B98.0	Helicobacter pylori [H.pylori] as the cause of diseases classified to other chapters
B98.1	Vibrio vulnificus as the cause of diseases classified to other chapters
B99*	Other and unspecified infectious diseases
C46	Kaposi sarcoma
D73.3	Abscess of spleen
E32.1	Abscess of thymus
G00	Bacterial meningitis, not elsewhere classified
G00.0	Haemophilus meningitis
G00.1	Pneumococcal meningitis
G00.2	Streptococcal meningitis
G00.3	Staphylococcal meningitis
G00.8	Other bacterial meningitis
G00.9	Bacterial meningitis, unspecified
G01*	Meningitis in bacterial diseases classified elsewhere
G02.0	Meningitis in viral diseases classified elsewhere
G02.1	Meningitis in mycoses
G02.8	Meningitis in other specified infectious and parasitic diseases classified elsewhere
G03*	Meningitis due to other and unspecified causes
G04.1	Tropical spastic paraplegia
G04.2	Bacterial meningoencephalitis and meningomyelitis, not elsewhere classified
G05.0	Encephalitis, myelitis and encephalomyelitis in bacterial diseases classified elsewhere
G05.1	Encephalitis, myelitis and encephalomyelitis in viral diseases classified elsewhere
G05.2	Encephalitis, myelitis and encephalomyelitis in other infectious and parasitic diseases classified elsewhere
G06*	Intracranial and intraspinal abscess and granuloma
G07*	Intracranial and intraspinal abscess and granuloma in diseases classified elsewhere
H00*	Hordeolum and chalazion
H01.0	Blepharitis

H05.0	Acute inflammation of orbit
H06.1	Parasitic infestation of orbit in diseases classified elsewhere
H10.0	Mucopurulent conjunctivitis
H10.5	Blepharoconjunctivitis
H13.0	Filarial infection of conjunctiva
H19.0	Scleritis and episcleritis in diseases classified elsewhere
H19.1	Herpesviral keratitis and keratoconjunctivitis
H19.2	Keratitis and keratoconjunctivitis in other infectious and parasitic diseases classified elsewhere
H22.0	Iridocyclitis in infectious and parasitic diseases classified elsewhere
H32.0	Chorioretinal inflammation in infectious and parasitic diseases classified elsewhere
H44.0	Purulent endophthalmitis
H60.0	Abscess of external ear
H60.1	Cellulitis of external ear
H60.2	Malignant otitis externa
H60.3	Other infective otitis externa
H62.0	Otitis externa in bacterial diseases classified elsewhere
H62.1	Otitis externa in viral diseases classified elsewhere
H62.2	Otitis externa in mycoses
H62.3	Otitis externa in other infectious and parasitic diseases classified elsewhere
H66.0	Acute suppurative otitis media
H67.0	Otitis media in bacterial diseases classified elsewhere
H67.1	Otitis media in viral diseases classified elsewhere
H70.0	Acute mastoiditis
H75.0	Mastoiditis in infectious and parasitic diseases classified elsewhere
I30.1	Infective pericarditis
I32.0	Pericarditis in bacterial diseases classified elsewhere
I32.1	Pericarditis in other infectious and parasitic diseases classified elsewhere
I33.0	Acute and subacute infective endocarditis
I40.0	Infective myocarditis
I41.0	Myocarditis in bacterial diseases classified elsewhere
I41.1	Myocarditis in viral diseases classified elsewhere
I41.2	Myocarditis in other infectious and parasitic diseases classified elsewhere
I43.0	Cardiomyopathy in infectious and parasitic diseases classified elsewhere
I52.0	Other heart disorders in bacterial diseases classified elsewhere
I52.1	Other heart disorders in other infectious and parasitic diseases classified elsewhere
I68.1	Cerebral arteritis in infectious and parasitic diseases classified elsewhere
J01.0	Acute maxillary sinusitis
J02	Acute pharyngitis

J02.0	Streptococcal pharyngitis
J02.8	Acute pharyngitis due to other specified organisms
J02.9	Acute pharyngitis, unspecified
J03	Acute tonsillitis
J03.0	Streptococcal tonsillitis
J03.8	Acute tonsillitis due to other specified organisms
J03.9	Acute tonsillitis, unspecified
J04*	Acute laryngitis and tracheitis
J05.1	Acute epiglottitis
J09*	Influenza due to certain identified influenza virus
J10*	Influenza due to other identified influenza virus
J11*	Influenza, virus not identified
J12*	Viral pneumonia, not elsewhere classified
J13*	Pneumonia due to Streptococcus pneumoniae
J14*	Pneumonia due to Haemophilus influenzae
J15	Bacterial pneumonia, not elsewhere classified
J15.0	Pneumonia due to Klebsiella pneumoniae
J15.1	Pneumonia due to Pseudomonas
J15.2	Pneumonia due to staphylococcus
J15.3	Pneumonia due to streptococcus, group B
J15.4	Pneumonia due to other streptococci
J15.5	Pneumonia due to Escherichia coli
J15.6	Pneumonia due to other aerobic Gram-negative bacteria
J15.7	Pneumonia due to Mycoplasma pneumoniae
J15.8	Other bacterial pneumonia
J15.9	Bacterial pneumonia, unspecified
J16	Pneumonia due to other infectious organisms, not elsewhere classified
J16.0	Chlamydial pneumonia
J16.8	Pneumonia due to other specified infectious organisms
J17.0	Pneumonia in bacterial diseases classified elsewhere
J17.1	Pneumonia in viral diseases classified elsewhere
J17.2	Pneumonia in mycoses
J17.3	Pneumonia in parasitic diseases
J17.8	Pneumonia in other diseases classified elsewhere
J18*	Pneumonia, organism unspecified
J20	Acute bronchitis
J20.0	Acute bronchitis due to Mycoplasma pneumoniae
J20.1	Acute bronchitis due to Haemophilus influenzae

J20.2	Acute bronchitis due to streptococcus
J20.3	Acute bronchitis due to coxsackievirus
J20.4	Acute bronchitis due to parainfluenza virus
J20.5	Acute bronchitis due to respiratory syncytial virus
J20.6	Acute bronchitis due to rhinovirus
J20.7	Acute bronchitis due to echovirus
J20.8	Acute bronchitis due to other specified organisms
J20.9	Acute bronchitis, unspecified
J21*	Acute bronchiolitis
J21.0	Acute bronchiolitis due to respiratory syncytial virus
J21.1	Acute bronchiolitis due to human metapneumovirus
J21.8	Acute bronchiolitis due to other specified organisms
J21.9	Acute bronchiolitis, unspecified
J22*	Unspecified acute lower respiratory infection
J36*	Peritonsillar abscess
J39.0	Retropharyngeal and parapharyngeal abscess
J39.1	Other abscess of pharynx
J85.1	Abscess of lung with pneumonia
J85.2	Abscess of lung without pneumonia
J85.3	Abscess of mediastinum
J86*	Pyothorax
K02*	Dental caries
K04.4	Acute apical periodontitis of pulpal origin
K04.5	Chronic apical periodontitis
K04.6	Periapical abscess with sinus
K04.7	Periapical abscess without sinus
K05.0	Acute gingivitis
K05.2	Acute periodontitis
K05.3	Chronic periodontitis
K05.4	Periodontosis
K11.3	Abscess of salivary gland
K12.2	Cellulitis and abscess of mouth
K23.0	Tuberculous oesophagitis
K23.1	Megaoesophagus in Chagas disease
K35*	Acute appendicitis
K57.0	Diverticular disease of small intestine with perforation and abscess
K57.2	Diverticular disease of large intestine with perforation and abscess
K57.4	Diverticular disease of both small and large intestine with perforation and abscess

K57.8	Diverticular disease of intestine, part unspecified, with perforation and abscess
K61*	Abscess of anal and rectal regions
K63.0	Abscess of intestine
K65.0	Acute peritonitis
K67.0	Chlamydial peritonitis
K67.1	Gonococcal peritonitis
K67.2	Syphilitic peritonitis
K67.3	Tuberculous peritonitis
K67.8	Other disorders of peritoneum in infectious diseases classified elsewhere
K75.0	Abscess of liver
K77.0	Liver disorders in infectious and parasitic diseases classified elsewhere
L00*	Staphylococcal scalded skin syndrome
L01*	Impetigo
L02*	Cutaneous abscess, furuncle and carbuncle
L03*	Cellulitis
L04*	Acute lymphadenitis
L05*	Pilonidal cyst
L08*	Other local infections of skin and subcutaneous tissue
L70.1	Acne conglobata
M00	Pyogenic arthritis
M00.0	Staphylococcal arthritis and polyarthritis
M00.1	Pneumococcal arthritis and polyarthritis
M00.2	Other streptococcal arthritis and polyarthritis
M00.8	Arthritis and polyarthritis due to other specified bacterial agents
M00.9	Pyogenic arthritis, unspecified
M01.0	Meningococcal arthritis
M01.1	Tuberculous arthritis
M01.2	Arthritis in Lyme disease
M01.3	Arthritis in other bacterial diseases classified elsewhere
M01.4	Rubella arthritis
M01.5	Arthritis in other viral diseases classified elsewhere
M01.6	Arthritis in mycoses
M01.8	Arthritis in other infectious and parasitic diseases classified elsewhere
M46.2	Osteomyelitis of vertebra
M46.3	Infection of intervertebral disc (pyogenic)
M46.5	Other infective spondylopathies
M49.0	Tuberculosis of spine
M49.1	Brucella spondylitis

M49.2	Enterobacterial spondylitis
M49.3	Spondylopathy in other infectious and parasitic diseases classified elsewhere
M60.0	Infective myositis
M63.0	Myositis in bacterial diseases classified elsewhere
M63.1	Myositis in protozoal and parasitic infections classified elsewhere
M63.2	Myositis in other infectious diseases classified elsewhere
M65.0	Abscess of tendon sheath
M65.1	Other infective (teno)synovitis
M71.0	Abscess of bursa
M71.1	Other infective bursitis
M72.6	Necrotizing fasciitis
M73.0	Gonococcal bursitis
M73.1	Syphilitic bursitis
M86*	Osteomyelitis
M86.0	Acute haematogenous osteomyelitis
M86.1	Other acute osteomyelitis
M86.2	Subacute osteomyelitis
M86.3	Chronic multifocal osteomyelitis
M86.4	Chronic osteomyelitis with draining sinus
M86.5	Other chronic haematogenous osteomyelitis
M86.6	Other chronic osteomyelitis
M86.8	Other osteomyelitis
M86.9	Osteomyelitis, unspecified
N08.0	Glomerular disorders in infectious and parasitic diseases classified elsewhere
N10*	Acute tubulo-interstitial nephritis
N13.6	Pyonephrosis
N15.1	Renal and perinephric abscess
N16.0	Renal tubulo-interstitial disorders in infectious and parasitic diseases classified elsewhere
N29.0	Late syphilis of kidney
N29.1	Other disorders of kidney and ureter in infectious and parasitic diseases classified elsewhere
N30.0	Acute cystitis
N34.0	Urethral abscess
N39.0	Urinary tract infection, site not specified
N41.0	Acute prostatitis
N41.2	Abscess of prostate
N45*	Orchitis and epididymitis
N61*	Inflammatory disorders of breast
N70.0	Acute salpingitis and oophoritis

N71.0	Acute inflammatory disease of uterus
N73.0	Acute parametritis and pelvic cellulitis
N73.3	Female acute pelvic peritonitis
N74	Female pelvic inflammatory disorders in diseases classified elsewhere
N74.0	Tuberculous infection of cervix uteri
N74.1	Female tuberculous pelvic inflammatory disease
N74.2	Female syphilitic pelvic inflammatory disease
N74.3	Female gonococcal pelvic inflammatory disease
N74.4	Female chlamydial pelvic inflammatory disease
N74.8	Female pelvic inflammatory disorders in other diseases classified elsewhere
N75.1	Abscess of Bartholin gland
N76.0	Acute vaginitis
N76.4	Abscess of vulva
N77.0	Ulceration of vulva in infectious and parasitic diseases classified elsewhere
N77.1	Vaginitis, vulvitis and vulvovaginitis in infectious and parasitic diseases classified elsewhere
O03.0	Spontaneous abortion: Incomplete, complicated by genital tract and pelvic infection
O03.5	Spontaneous abortion: Complete or unspecified, complicated by genital tract and pelvic infection
O04.0	Medical abortion: Incomplete, complicated by genital tract and pelvic infection
O04.5	Medical abortion: Complete or unspecified, complicated by genital tract and pelvic infection
O05.0	Other abortion: Incomplete, complicated by genital tract and pelvic infection
O05.5	Other abortion: Complete or unspecified, complicated by genital tract and pelvic infection
O06.0	Unspecified abortion: Incomplete, complicated by genital tract and pelvic infection
O06.5	Unspecified abortion: Complete or unspecified, complicated by genital tract and pelvic infection
O07.0	Failed medical abortion, complicated by genital tract and pelvic infection
O07.5	Other and unspecified failed attempted abortion, complicated by genital tract and pelvic infection
O08.0	Genital tract and pelvic infection following abortion and ectopic and molar pregnancy
O23.0	Infections of kidney in pregnancy
O23.1	Infections of bladder in pregnancy
O23.2	Infections of urethra in pregnancy
O23.3	Infections of other parts of urinary tract in pregnancy
O23.4	Unspecified infection of urinary tract in pregnancy
O23.5	Infections of the genital tract in pregnancy
O23.9	Other and unspecified genitourinary tract infection in pregnancy
O75.3	Other infection during labour
O85*	Puerperal sepsis
O86*	Other puerperal infections
O91.0	Infection of nipple associated with childbirth
O91.1	Abscess of breast associated with childbirth

O98	Maternal infectious and parasitic diseases classifiable elsewhere but complicating pregnancy, childbirth and the puerperium
O98.0	Tuberculosis complicating pregnancy, childbirth and the puerperium
O98.1	Syphilis complicating pregnancy, childbirth and the puerperium
O98.2	Gonorrhoea complicating pregnancy, childbirth and the puerperium
O98.3	Other infections with a predominantly sexual mode of transmission complicating pregnancy, childbirth and the puerperium
O98.4	Viral hepatitis complicating pregnancy, childbirth and the puerperium
O98.5	Other viral diseases complicating pregnancy, childbirth and the puerperium
O98.6	Protozoal diseases complicating pregnancy, childbirth and the puerperium
O98.7	Human immunodeficiency virus [HIV] disease complicating pregnancy, childbirth and the puerperium
O98.8	Other maternal infectious and parasitic diseases complicating pregnancy, childbirth and the puerperium
O98.9	Unspecified maternal infectious or parasitic disease complicating pregnancy, childbirth and the puerperium
P23	Congenital pneumonia
P23.0	Congenital pneumonia due to viral agent
P23.1	Congenital pneumonia due to Chlamydia
P23.2	Congenital pneumonia due to staphylococcus
P23.3	Congenital pneumonia due to streptococcus, group B
P23.4	Congenital pneumonia due to Escherichia coli
P23.5	Congenital pneumonia due to Pseudomonas
P23.6	Congenital pneumonia due to other bacterial agents
P23.8	Congenital pneumonia due to other organisms
P23.9	Congenital pneumonia, unspecified
P35.0	Congenital rubella syndrome
P35.1	Congenital cytomegalovirus infection
P35.2	Congenital herpesviral [herpes simplex] infection
P35.3	Congenital viral hepatitis
P36	Bacterial sepsis of newborn
P36.0	Sepsis of newborn due to streptococcus, group B
P36.1	Sepsis of newborn due to other and unspecified streptococci
P36.2	Sepsis of newborn due to Staphylococcus aureus
P36.3	Sepsis of newborn due to other and unspecified staphylococci
P36.4	Sepsis of newborn due to Escherichia coli
P36.5	Sepsis of newborn due to anaerobes
P36.8	Other bacterial sepsis of newborn
P36.9	Bacterial sepsis of newborn, unspecified
P37	Other congenital infectious and parasitic diseases
P37.0	Congenital tuberculosis
P37.1	Congenital toxoplasmosis

P37.2	Neonatal (disseminated) listeriosis
P37.3	Congenital falciparum malaria
P37.4	Other congenital malaria
P37.5	Neonatal candidiasis
P37.8	Other specified congenital infectious and parasitic diseases
P37.9	Congenital infectious and parasitic disease, unspecified
P38*	Omphalitis of newborn with or without mild haemorrhage
P39	Other infections specific to the perinatal period
P39.0	Neonatal infective mastitis
P39.1	Neonatal conjunctivitis and dacryocystitis
P39.2	Intra-amniotic infection of fetus, not elsewhere classified
P39.3	Neonatal urinary tract infection
P39.4	Neonatal skin infection
P39.8	Other specified infections specific to the perinatal period
P39.9	Infection specific to the perinatal period, unspecified
R57.2	Septic shock
R65.0	Systemic Inflammatory Response Syndrome of infectious origin without organ failure
R65.1	Systemic Inflammatory Response Syndrome of infectious origin with organ failure
Z21	Asymptomatic human immunodeficiency virus [HIV] infection status

The list above comprises almost all codes under Chapter I (Certain infectious and parasitic diseases) of ICD-10 and additionally infectious disease codes from other Chapters of ICD-10. The few excluded codes include sequelae of infectious and parasitic diseases when the infection is not anymore present [B90-B94], prion diseases and other very rare central nervous system conditions [A81.0, A81.8, A81.9], rabies [A82], smallbox [B03], common cold [J00], acute upper respiratory infections of multiple and unspecified sites [J06], complications of surgical and medical care, not elsewhere classified [T80-T88], and codes for special purposes [U00-U85]. UK Biobank replication analysis comprised all codes under Chapter I (Certain infectious and parasitic diseases) of ICD-10 and the same codes from other Chapters of ICD-10 as the primary analysis. * indicates inclusion of underlying 4- and 5-digit codes.

All bacterial infections

A00*	Cholera
A01*	Typhoid and paratyphoid fevers
A02	Other salmonella infections
A02.0	Salmonella enteritis
A02.1	Salmonella sepsis
A02.2	Localized salmonella infections
A02.8	Other specified salmonella infections
A02.9	Salmonella infection, unspecified
A03	Shigellosis
A03.0	Shigellosis due to Shigella dysenteriae
A03.1	Shigellosis due to Shigella flexneri
A03.2	Shigellosis due to Shigella boydii
A03.3	Shigellosis due to Shigella sonnei
A03.8	Other shigellosis

A03.9	Shigellosis, unspecified
A04	Other bacterial intestinal infections
A04.0	Enteropathogenic Escherichia coli infection
A04.1	Enterotoxigenic Escherichia coli infection
A04.2	Enteroinvasive Escherichia coli infection
A04.3	Enterohaemorrhagic Escherichia coli infection
A04.4	Other intestinal Escherichia coli infections
A04.5	Campylobacter enteritis
A04.6	Enteritis due to Yersinia enterocolitica
A04.7	Enterocolitis due to Clostridium difficile
A04.8	Other specified bacterial intestinal infections
A04.9	Bacterial intestinal infection, unspecified
A05	Other bacterial foodborne intoxications, not elsewhere classified
A05.0	Foodborne staphylococcal intoxication
A05.1	Botulism
A05.2	Foodborne Clostridium perfringens [Clostridium welchii] intoxication
A05.3	Foodborne Vibrio parahaemolyticus intoxication
A05.4	Foodborne Bacillus cereus intoxication
A05.8	Other specified bacterial foodborne intoxications
A05.9	Bacterial foodborne intoxication, unspecified
A09*	Other gastroenteritis and colitis of infectious and unspecified origin
A15*	Respiratory tuberculosis, bacteriologically and histologically confirmed
A16*	Respiratory tuberculosis, not confirmed bacteriologically or histologically
A17	Tuberculosis of nervous system
A17.0	Tuberculous meningitis
A17.1	Meningeal tuberculoma
A17.8	Other tuberculosis of nervous system
A17.9	Tuberculosis of nervous system, unspecified
A18*	Tuberculosis of other organs
A19*	Miliary tuberculosis
A20	Plague
A20.0	Bubonic plague
A20.1	Cellulocutaneous plague
A20.2	Pneumonic plague
A20.3	Plague meningitis
A20.7	Septicaemic plague
A20.8	Other forms of plague
A20.9	Plague, unspecified
A21	Tularaemia
A21.0	Ulceroglandular tularaemia
A21.1	Oculoglandular tularaemia
A21.2	Pulmonary tularaemia
A21.3	Gastrointestinal tularaemia
A21.7	Generalized tularaemia
A21.8	Other forms of tularaemia
A21.9	Tularaemia, unspecified
A22	Anthrax
A22.0	Cutaneous anthrax

A22.1	Pulmonary anthrax
A22.2	Gastrointestinal anthrax
A22.7	Anthrax sepsis
A22.8	Other forms of anthrax
A22.9	Anthrax, unspecified
A23	Brucellosis
A23.0	Brucellosis due to Brucella melitensis
A23.1	Brucellosis due to Brucella abortus
A23.2	Brucellosis due to Brucella suis
A23.3	Brucellosis due to Brucella canis
A23.8	Other brucellosis
A23.9	Brucellosis, unspecified
A24	Glanders and melioidosis
A24.0	Glanders
A24.1	Acute and fulminating melioidosis
A24.2	Subacute and chronic melioidosis
A24.3	Other melioidosis
A25	Rat-bite fevers
A25.0	Spirillosis
A25.1	Streptobacillosis
A25.9	Rat-bite fever, unspecified
A26	Erysipeloid
A26.0	Cutaneous erysipeloid
A26.7	Erysipelothrix sepsis
A26.8	Other forms of erysipeloid
A26.9	Erysipeloid, unspecified
A27*	Leptospirosis
A28	Other zoonotic bacterial diseases, not elsewhere classified
A28.0	Pasteurellosis
A28.1	Cat-scratch disease
A28.2	Extraintestinal yersiniosis
A28.8	Other specified zoonotic bacterial diseases, not elsewhere classified
A28.9	Zoonotic bacterial disease, unspecified
A30*	Leprosy [Hansen disease]
A31*	Infection due to other mycobacteria
A32	Listeriosis
A32.0	Cutaneous listeriosis
A32.1	Listerial meningitis and meningoencephalitis
A32.7	Listerial sepsis
A32.8	Other forms of listeriosis
A32.9	Listeriosis, unspecified
A33*	Tetanus neonatorum
A34*	Obstetrical tetanus
A35*	Other tetanus
A36*	Diphtheria
A37*	Whooping cough
A38*	Scarlet fever
A39	Meningococcal infection

A39.0	Meningococcal meningitis
A39.1	Waterhouse-Friderichsen syndrome
A39.2	Acute meningococcaemia
A39.3	Chronic meningococcaemia
A39.4	Meningococcaemia, unspecified
A39.5	Meningococcal heart disease
A39.8	Other meningococcal infections
A39.9	Meningococcal infection, unspecified
A40	Streptococcal sepsis
A40.0	Sepsis due to streptococcus, group A
A40.1	Sepsis due to streptococcus, group B
A40.2	Sepsis due to streptococcus, group D
A40.3	Sepsis due to Streptococcus pneumoniae
A40.8	Other streptococcal sepsis
A40.9	Streptococcal sepsis, unspecified
A41	Other sepsis
A41.0	Sepsis due to Staphylococcus aureus
A41.1	Sepsis due to other specified staphylococcus
A41.2	Sepsis due to unspecified staphylococcus
A41.3	Sepsis due to Haemophilus influenzae
A41.4	Sepsis due to anaerobes
A41.5	Sepsis due to other Gram-negative organisms
A41.8	Other specified sepsis
A41.9	Sepsis, unspecified
A42	Actinomycosis
A42.0	Pulmonary actinomycosis
A42.1	Abdominal actinomycosis
A42.2	Cervicofacial actinomycosis
A42.7	Actinomycotic sepsis
A42.8	Other forms of actinomycosis
A42.9	Actinomycosis, unspecified
A43	Nocardiosis
A43.0	Pulmonary nocardiosis
A43.1	Cutaneous nocardiosis
A43.8	Other forms of nocardiosis
A44	Bartonellosis
A44.0	Systemic bartonellosis
A44.1	Cutaneous and mucocutaneous bartonellosis
A44.8	Other forms of bartonellosis
A44.9	Bartonellosis, unspecified
A46*	Erysipelas
A48.0	Gas gangrene
A48.1	Legionnaires disease
A48.2	Nonpneumonic Legionnaires disease [Pontiac fever]
A48.3	Toxic shock syndrome
A48.4	Brazilian purpuric fever
A48.8	Other specified bacterial diseases
A49	Bacterial infection of unspecified site

A49.0	Staphylococcal infection, unspecified site
A49.1	Streptococcal infection, unspecified site
A49.2	Haemophilus influenzae infection, unspecified site
A49.3	Mycoplasma infection, unspecified site
A49.8	Other bacterial infections of unspecified site
A49.9	Bacterial infection, unspecified
A50	Congenital syphilis
A50.0	Early congenital syphilis, symptomatic
A50.1	Early congenital syphilis, latent
A50.2	Early congenital syphilis, unspecified
A50.3	Late congenital syphilitic oculopathy
A50.4	Late congenital neurosyphilis [juvenile neurosyphilis]
A50.5	Other late congenital syphilis, symptomatic
A50.6	Late congenital syphilis, latent
A50.7	Late congenital syphilis, unspecified
A50.9	Congenital syphilis, unspecified
A51	Early syphilis
A51.0	Primary genital syphilis
A51.1	Primary anal syphilis
A51.2	Primary syphilis of other sites
A51.3	Secondary syphilis of skin and mucous membranes
A51.4	Other secondary syphilis
A51.5	Early syphilis, latent
A51.9	Early syphilis, unspecified
A52	Late syphilis
A52.0	Cardiovascular syphilis
A52.1	Symptomatic neurosyphilis
A52.2	Asymptomatic neurosyphilis
A52.3	Neurosyphilis, unspecified
A52.7	Other symptomatic late syphilis
A52.8	Late syphilis, latent
A52.9	Late syphilis, unspecified
A53*	Other and unspecified syphilis
A54	Gonococcal infection
A54.0	Gonococcal infection of lower genitourinary tract without periurethral or accessory gland abscess
A54.1	Gonococcal infection of lower genitourinary tract with periurethral and accessory gland abscess
A54.2	Gonococcal pelviperitonitis and other gonococcal genitourinary infections
A54.3	Gonococcal infection of eye
A54.4	Gonococcal infection of musculoskeletal system
A54.5	Gonococcal pharyngitis
A54.6	Gonococcal infection of anus and rectum
A54.8	Other gonococcal infections
A54.9	Gonococcal infection, unspecified
A55*	Chlamydial lymphogranuloma (venereum)
A56.0	Chlamydial infection of lower genitourinary tract
A56.1	Chlamydial infection of pelviperitoneum and other genitourinary organs
A56.2	Chlamydial infection of genitourinary tract, unspecified
A56.3	Chlamydial infection of anus and rectum

A56.4	Chlamydial infection of pharynx
A56.8	Sexually transmitted chlamydial infection of other sites
A57*	Chancroid
A58*	Granuloma inguinale
A64*	Unspecified sexually transmitted disease
A65*	Nonvenereal syphilis
A66*	Yaws
A67*	Pinta [carate]
A68*	Relapsing fevers
A69	Other spirochaetal infections
A69.0	Necrotizing ulcerative stomatitis
A69.1	Other Vincent infections
A69.2	Lyme disease
A69.8	Other specified spirochaetal infections
A69.9	Spirochaetal infection, unspecified
A70*	Chlamydia psittaci infection
A71*	Trachoma
A74.0	Chlamydial conjunctivitis
A74.8	Other chlamydial diseases
A74.9	Chlamydial infection, unspecified
A75	Typhus fever
A75.0	Epidemic louse-borne typhus fever due to Rickettsia prowazekii
A75.1	Recrudescent typhus [Brill disease]
A75.2	Typhus fever due to Rickettsia typhi
A75.3	Typhus fever due to Rickettsia tsutsugamushi
A75.9	Typhus fever, unspecified
A77	Spotted fever [tick-borne rickettsioses]
A77.0	Spotted fever due to Rickettsia rickettsii
A77.1	Spotted fever due to Rickettsia conorii
A77.2	Spotted fever due to Rickettsia sibirica
A77.3	Spotted fever due to Rickettsia australis
A77.8	Other spotted fevers
A77.9	Spotted fever, unspecified
A78*	Q fever
A79	Other rickettsioses
A79.0	Trench fever
A79.1	Rickettsialpox due to Rickettsia akari
A79.8	Other specified rickettsioses
A79.9	Rickettsiosis, unspecified
B47.1	Actinomycetoma
B95*	Streptococcus and staphylococcus as the cause of diseases classified to other chapters
B96.0	Mycoplasma pneumoniae [M. pneumoniae] as the cause of diseases classified to other chapters
B96.1	Klebsiella pneumoniae [K. pneumoniae] as the cause of diseases classified to other chapters
B96.2	Escherichia coli [E. coli] as the cause of diseases classified to other chapters
B96.3	Haemophilus influenzae [H. influenzae] as the cause of diseases classified to other chapters
B96.4	Proteus (mirabilis)(morganii) as the cause of diseases classified to other chapters
B96.5	Pseudomonas (aeruginosa) as the cause of diseases classified to other chapters
B96.6	Bacillus fragilis [B. fragilis] as the cause of diseases classified to other chapters

B96.7	Clostridium perfringens [C. perfringens] as the cause of diseases classified to other chapters
B96.8	Other specified bacterial agents as the cause of diseases classified to other chapters
B98.0	Helicobacter pylori [H.pylori] as the cause of diseases classified to other chapters
B98.1	Vibrio vulnificus as the cause of diseases classified to other chapters
D73.3	Abscess of spleen
E32.1	Abscess of thymus
G00	Bacterial meningitis, not elsewhere classified
G00.0	Haemophilus meningitis
G00.1	Pneumococcal meningitis
G00.2	Streptococcal meningitis
G00.3	Staphylococcal meningitis
G00.8	Other bacterial meningitis
G00.9	Bacterial meningitis, unspecified
G01*	Meningitis in bacterial diseases classified elsewhere
G04.2	Bacterial meningoencephalitis and meningomyelitis, not elsewhere classified
G05.0	Encephalitis, myelitis and encephalomyelitis in bacterial diseases classified elsewhere
G06*	Intracranial and intraspinal abscess and granuloma
H00*	Hordeolum and chalazion
H01.0	Blepharitis
H05.0	Acute inflammation of orbit
H10.0	Mucopurulent conjunctivitis
H10.5	Blepharoconjunctivitis
H44.0	Purulent endophthalmitis
H60.0	Abscess of external ear
H60.1	Cellulitis of external ear
H60.2	Malignant otitis externa
H60.3	Other infective otitis externa
H62.0	Otitis externa in bacterial diseases classified elsewhere
H66.0	Acute suppurative otitis media
H67.0	Otitis media in bacterial diseases classified elsewhere
H70.0	Acute mastoiditis
I32.0	Pericarditis in bacterial diseases classified elsewhere
I33.0	Acute and subacute infective endocarditis
I41.0	Myocarditis in bacterial diseases classified elsewhere
I52.0	Other heart disorders in bacterial diseases classified elsewhere
J01.0	Acute maxillary sinusitis
J02	Acute pharyngitis
J02.0	Streptococcal pharyngitis
J02.8	Acute pharyngitis due to other specified organisms
J02.9	Acute pharyngitis, unspecified
J03	Acute tonsillitis
J03.0	Streptococcal tonsillitis
J03.8	Acute tonsillitis due to other specified organisms
J03.9	Acute tonsillitis, unspecified
J04*	Acute laryngitis and tracheitis
J05.1	Acute epiglottitis
J13*	Pneumonia due to Streptococcus pneumoniae
J14*	Pneumonia due to Haemophilus influenzae

J15	Bacterial pneumonia, not elsewhere classified
J15.0	Pneumonia due to Klebsiella pneumoniae
J15.1	Pneumonia due to Pseudomonas
J15.2	Pneumonia due to staphylococcus
J15.3	Pneumonia due to streptococcus, group B
J15.4	Pneumonia due to other streptococci
J15.5	Pneumonia due to Escherichia coli
J15.6	Pneumonia due to other aerobic Gram-negative bacteria
J15.7	Pneumonia due to Mycoplasma pneumoniae
J15.8	Other bacterial pneumonia
J15.9	Bacterial pneumonia, unspecified
J16	Pneumonia due to other infectious organisms, not elsewhere classified
J16.0	Chlamydial pneumonia
J16.8	Pneumonia due to other specified infectious organisms
J17.0	Pneumonia in bacterial diseases classified elsewhere
J17.8	Pneumonia in other diseases classified elsewhere
J18*	Pneumonia, organism unspecified
J20.0	Acute bronchitis due to Mycoplasma pneumoniae
J20.1	Acute bronchitis due to Haemophilus influenzae
J20.2	Acute bronchitis due to streptococcus
J22*	Unspecified acute lower respiratory infection
J36*	Peritonsillar abscess
J39.0	Retropharyngeal and parapharyngeal abscess
J39.1	Other abscess of pharynx
J85.1	Abscess of lung with pneumonia
J85.2	Abscess of lung without pneumonia
J85.3	Abscess of mediastinum
J86*	Pyothorax
K02*	Dental caries
K04.4	Acute apical periodontitis of pulpal origin
K04.5	Chronic apical periodontitis
K04.6	Periapical abscess with sinus
K04.7	Periapical abscess without sinus
K05.0	Acute gingivitis
K05.2	Acute periodontitis
K05.3	Chronic periodontitis
K05.4	Periodontosis
K11.3	Abscess of salivary gland
K12.2	Cellulitis and abscess of mouth
K23.0	Tuberculous oesophagitis
K35*	Acute appendicitis
K57.0	Diverticular disease of small intestine with perforation and abscess
K57.2	Diverticular disease of large intestine with perforation and abscess
K57.4	Diverticular disease of both small and large intestine with perforation and abscess
K57.8	Diverticular disease of intestine, part unspecified, with perforation and abscess
K61*	Abscess of anal and rectal regions
K63.0	Abscess of intestine
K65.0	Acute peritonitis

K67.0	Chlamydial peritonitis
K67.1	Gonococcal peritonitis
K67.2	Syphilitic peritonitis
K67.3	Tuberculous peritonitis
K75.0	Abscess of liver
L00*	Staphylococcal scalded skin syndrome
L01*	Impetigo
L02*	Cutaneous abscess, furuncle and carbuncle
L03*	Cellulitis
L04*	Acute lymphadenitis
L05*	Pilonidal cyst
L08*	Other local infections of skin and subcutaneous tissue
L70.1	Acne conglobata
M00	Pyogenic arthritis
M00.0	Staphylococcal arthritis and polyarthritis
M00.1	Pneumococcal arthritis and polyarthritis
M00.2	Other streptococcal arthritis and polyarthritis
M00.8	Arthritis and polyarthritis due to other specified bacterial agents
M00.9	Pyogenic arthritis, unspecified
M01.0	Meningococcal arthritis
M01.1	Tuberculous arthritis
M01.2	Arthritis in Lyme disease
M01.3	Arthritis in other bacterial diseases classified elsewhere
M46.2	Osteomyelitis of vertebra
M46.3	Infection of intervertebral disc (pyogenic)
M46.5	Other infective spondylopathies
M49.0	Tuberculosis of spine
M49.1	Brucella spondylitis
M49.2	Enterobacterial spondylitis
M63.0	Myositis in bacterial diseases classified elsewhere
M65.0	Abscess of tendon sheath
M65.1	Other infective (teno)synovitis
M71.0	Abscess of bursa
M71.1	Other infective bursitis
M72.6	Necrotizing fasciitis
M73.0	Gonococcal bursitis
M73.1	Syphilitic bursitis
M86*	Osteomyelitis
M86.0	Acute haematogenous osteomyelitis
M86.1	Other acute osteomyelitis
M86.2	Subacute osteomyelitis
M86.3	Chronic multifocal osteomyelitis
M86.4	Chronic osteomyelitis with draining sinus
M86.5	Other chronic haematogenous osteomyelitis
M86.6	Other chronic osteomyelitis
M86.8	Other osteomyelitis
M86.9	Osteomyelitis, unspecified
N10*	Acute tubulo-interstitial nephritis

N13.6	Pyonephrosis
N15.1	Renal and perinephric abscess
N29.0	Late syphilis of kidney
N30.0	Acute cystitis
N34.0	Urethral abscess
N39.0	Urinary tract infection, site not specified
N41.0	Acute prostatitis
N41.2	Abscess of prostate
N45*	Orchitis and epididymitis
N61*	Inflammatory disorders of breast
N70.0	Acute salpingitis and oophoritis
N71.0	Acute inflammatory disease of uterus
N73.0	Acute parametritis and pelvic cellulitis
N73.3	Female acute pelvic peritonitis
N74.0	Tuberculous infection of cervix uteri
N74.1	Female tuberculous pelvic inflammatory disease
N74.2	Female syphilitic pelvic inflammatory disease
N74.3	Female gonococcal pelvic inflammatory disease
N74.4	Female chlamydial pelvic inflammatory disease
N75.1	Abscess of Bartholin gland
N76.0	Acute vaginitis
N76.4	Abscess of vulva
O03.0	Spontaneous abortion: Incomplete, complicated by genital tract and pelvic infection
O03.5	Spontaneous abortion: Complete or unspecified, complicated by genital tract and pelvic infection
O04.0	Medical abortion: Incomplete, complicated by genital tract and pelvic infection
O04.5	Medical abortion: Complete or unspecified, complicated by genital tract and pelvic infection
O05.0	Other abortion: Incomplete, complicated by genital tract and pelvic infection
O05.5	Other abortion: Complete or unspecified, complicated by genital tract and pelvic infection
O06.0	Unspecified abortion: Incomplete, complicated by genital tract and pelvic infection
O06.5	Unspecified abortion: Complete or unspecified, complicated by genital tract and pelvic infection
O07.0	Failed medical abortion, complicated by genital tract and pelvic infection
O07.5	Other and unspecified failed attempted abortion, complicated by genital tract and pelvic infection
O08.0	Genital tract and pelvic infection following abortion and ectopic and molar pregnancy
O23.0	Infections of kidney in pregnancy
O23.1	Infections of bladder in pregnancy
O23.2	Infections of urethra in pregnancy
O23.3	Infections of other parts of urinary tract in pregnancy
O23.4	Unspecified infection of urinary tract in pregnancy
O23.5	Infections of the genital tract in pregnancy
O23.9	Other and unspecified genitourinary tract infection in pregnancy
O75.3	Other infection during labour
O85*	Puerperal sepsis
O86*	Other puerperal infections
O91.0	Infection of nipple associated with childbirth
O91.1	Abscess of breast associated with childbirth
O98.0	Tuberculosis complicating pregnancy, childbirth and the puerperium
O98.1	Syphilis complicating pregnancy, childbirth and the puerperium
O98.2	Gonorrhoea complicating pregnancy, childbirth and the puerperium

O98.3	Other infections with a predominantly sexual mode of transmission complicating pregnancy, childbirth and the puerperium
O98.8	Other maternal infectious and parasitic diseases complicating pregnancy, childbirth and the puerperium
O98.9	Unspecified maternal infectious or parasitic disease complicating pregnancy, childbirth and the puerperium
P23	Congenital pneumonia
P23.1	Congenital pneumonia due to Chlamydia
P23.2	Congenital pneumonia due to staphylococcus
P23.3	Congenital pneumonia due to streptococcus, group B
P23.4	Congenital pneumonia due to Escherichia coli
P23.5	Congenital pneumonia due to Pseudomonas
P23.6	Congenital pneumonia due to other bacterial agents
P23.9	Congenital pneumonia, unspecified
P36	Bacterial sepsis of newborn
P36.0	Sepsis of newborn due to streptococcus, group B
P36.1	Sepsis of newborn due to other and unspecified streptococci
P36.2	Sepsis of newborn due to Staphylococcus aureus
P36.3	Sepsis of newborn due to other and unspecified staphylococci
P36.4	Sepsis of newborn due to Escherichia coli
P36.5	Sepsis of newborn due to anaerobes
P36.8	Other bacterial sepsis of newborn
P36.9	Bacterial sepsis of newborn, unspecified
P37.0	Congenital tuberculosis
P37.2	Neonatal (disseminated) listeriosis
P38*	Omphalitis of newborn with or without mild haemorrhage
P39.0	Neonatal infective mastitis
P39.1	Neonatal conjunctivitis and dacryocystitis
P39.2	Intra-amniotic infection of fetus, not elsewhere classified
P39.3	Neonatal urinary tract infection
P39.4	Neonatal skin infection

Invasive bacterial infections

A01*	Typhoid and paratyphoid fevers
A02	Other salmonella infections
A02.0	Salmonella enteritis
A02.1	Salmonella sepsis
A02.2	Localized salmonella infections
A02.8	Other specified salmonella infections
A02.9	Salmonella infection, unspecified
A03	Shigellosis
A03.0	Shigellosis due to Shigella dysenteriae
A03.1	Shigellosis due to Shigella flexneri
A03.2	Shigellosis due to Shigella boydii
A03.3	Shigellosis due to Shigella sonnei
A03.8	Other shigellosis
A03.9	Shigellosis, unspecified
A15*	Respiratory tuberculosis, bacteriologically and histologically confirmed

A16*	Respiratory tuberculosis, not confirmed bacteriologically or histologically
A17	Tuberculosis of nervous system
A17.0	Tuberculous meningitis
A17.1	Meningeal tuberculoma
A17.8	Other tuberculosis of nervous system
A17.9	Tuberculosis of nervous system, unspecified
A18*	Tuberculosis of other organs
A19*	Miliary tuberculosis
A20	Plague
A20.0	Bubonic plague
A20.1	Cellulocutaneous plague
A20.2	Pneumonic plague
A20.3	Plague meningitis
A20.7	Septicaemic plague
A20.8	Other forms of plague
A20.9	Plague, unspecified
A21	Tularaemia
A21.0	Ulceroglandular tularaemia
A21.1	Oculoglandular tularaemia
A21.2	Pulmonary tularaemia
A21.3	Gastrointestinal tularaemia
A21.7	Generalized tularaemia
A21.8	Other forms of tularaemia
A21.9	Tularaemia, unspecified
A22	Anthrax
A22.0	Cutaneous anthrax
A22.1	Pulmonary anthrax
A22.2	Gastrointestinal anthrax
A22.7	Anthrax sepsis
A22.8	Other forms of anthrax
A22.9	Anthrax, unspecified
A23	Brucellosis
A23.0	Brucellosis due to Brucella melitensis
A23.1	Brucellosis due to Brucella abortus
A23.2	Brucellosis due to Brucella suis
A23.3	Brucellosis due to Brucella canis
A23.8	Other brucellosis
A23.9	Brucellosis, unspecified
A24	Glanders and melioidosis
A24.0	Glanders
A24.1	Acute and fulminating melioidosis
A24.2	Subacute and chronic melioidosis
A24.3	Other melioidosis
A25	Rat-bite fevers
A25.0	Spirillosis
A25.1	Streptobacillosis
A25.9	Rat-bite fever, unspecified
A26.7	Erysipelothrix sepsis

A26.8	Other forms of erysipeloid
A27*	Leptospirosis
A28	Other zoonotic bacterial diseases, not elsewhere classified
A28.0	Pasteurellosis
A28.1	Cat-scratch disease
A28.2	Extraintestinal yersiniosis
A28.8	Other specified zoonotic bacterial diseases, not elsewhere classified
A28.9	Zoonotic bacterial disease, unspecified
A30*	Leprosy [Hansen disease]
A31*	Infection due to other mycobacteria
A32	Listeriosis
A32.0	Cutaneous listeriosis
A32.1	Listerial meningitis and meningoencephalitis
A32.7	Listerial sepsis
A32.8	Other forms of listeriosis
A32.9	Listeriosis, unspecified
A33*	Tetanus neonatorum
A34*	Obstetrical tetanus
A35*	Other tetanus
A36*	Diphtheria
A38*	Scarlet fever
A39	Meningococcal infection
A39.0	Meningococcal meningitis
A39.1	Waterhouse-Friderichsen syndrome
A39.2	Acute meningococcaemia
A39.3	Chronic meningococcaemia
A39.4	Meningococcaemia, unspecified
A39.5	Meningococcal heart disease
A39.8	Other meningococcal infections
A39.9	Meningococcal infection, unspecified
A40	Streptococcal sepsis
A40.0	Sepsis due to streptococcus, group A
A40.1	Sepsis due to streptococcus, group B
A40.2	Sepsis due to streptococcus, group D
A40.3	Sepsis due to Streptococcus pneumoniae
A40.8	Other streptococcal sepsis
A40.9	Streptococcal sepsis, unspecified
A41	Other sepsis
A41.0	Sepsis due to Staphylococcus aureus
A41.1	Sepsis due to other specified staphylococcus
A41.2	Sepsis due to unspecified staphylococcus
A41.3	Sepsis due to Haemophilus influenzae
A41.4	Sepsis due to anaerobes
A41.5	Sepsis due to other Gram-negative organisms
A41.8	Other specified sepsis
A41.9	Sepsis, unspecified
A42	Actinomycosis
A42.0	Pulmonary actinomycosis

A42.1	Abdominal actinomycosis
A42.2	Cervicofacial actinomycosis
A42.7	Actinomycotic sepsis
A42.8	Other forms of actinomycosis
A42.9	Actinomycosis, unspecified
A43	Nocardiosis
A43.0	Pulmonary nocardiosis
A43.1	Cutaneous nocardiosis
A43.8	Other forms of nocardiosis
A44	Bartonellosis
A44.0	Systemic bartonellosis
A44.1	Cutaneous and mucocutaneous bartonellosis
A44.8	Other forms of bartonellosis
A44.9	Bartonellosis, unspecified
A46*	Erysipelas
A48.0	Gas gangrene
A48.1	Legionnaires disease
A48.3	Toxic shock syndrome
A48.4	Brazilian purpuric fever
A48.8	Other specified bacterial diseases
A49.0	Staphylococcal infection, unspecified site
A49.1	Streptococcal infection, unspecified site
A49.2	Haemophilus influenzae infection, unspecified site
A49.8	Other bacterial infections of unspecified site
A49.9	Bacterial infection, unspecified
A50	Congenital syphilis
A50.0	Early congenital syphilis, symptomatic
A50.1	Early congenital syphilis, latent
A50.2	Early congenital syphilis, unspecified
A50.3	Late congenital syphilitic oculopathy
A50.4	Late congenital neurosyphilis [juvenile neurosyphilis]
A50.5	Other late congenital syphilis, symptomatic
A50.6	Late congenital syphilis, latent
A50.7	Late congenital syphilis, unspecified
A50.9	Congenital syphilis, unspecified
A51	Early syphilis
A51.0	Primary genital syphilis
A51.1	Primary anal syphilis
A51.2	Primary syphilis of other sites
A51.3	Secondary syphilis of skin and mucous membranes
A51.4	Other secondary syphilis
A51.5	Early syphilis, latent
A51.9	Early syphilis, unspecified
A52	Late syphilis
A52.0	Cardiovascular syphilis
A52.1	Symptomatic neurosyphilis
A52.2	Asymptomatic neurosyphilis
A52.3	Neurosyphilis, unspecified

A52.7	Other symptomatic late syphilis
A52.8	Late syphilis, latent
A52.9	Late syphilis, unspecified
A53*	Other and unspecified syphilis
A54.1	Gonococcal infection of lower genitourinary tract with periurethral and accessory gland abscess
A54.2	Gonococcal pelviperitonitis and other gonococcal genitourinary infections
A54.4	Gonococcal infection of musculoskeletal system
A54.8	Other gonococcal infections
A55*	Chlamydial lymphogranuloma (venereum)
A56.1	Chlamydial infection of pelviperitoneum and other genitourinary organs
A65*	Nonvenereal syphilis
A66*	Yaws
A67*	Pinta [carate]
A68*	Relapsing fevers
A69	Other spirochaetal infections
A69.0	Necrotizing ulcerative stomatitis
A69.1	Other Vincent infections
A69.2	Lyme disease
A69.8	Other specified spirochaetal infections
A69.9	Spirochaetal infection, unspecified
A70*	Chlamydia psittaci infection
A75	Typhus fever
A75.0	Epidemic louse-borne typhus fever due to Rickettsia prowazekii
A75.1	Recrudescent typhus [Brill disease]
A75.2	Typhus fever due to Rickettsia typhi
A75.3	Typhus fever due to Rickettsia tsutsugamushi
A75.9	Typhus fever, unspecified
A77	Spotted fever [tick-borne rickettsioses]
A77.0	Spotted fever due to Rickettsia rickettsii
A77.1	Spotted fever due to Rickettsia conorii
A77.2	Spotted fever due to Rickettsia sibirica
A77.3	Spotted fever due to Rickettsia australis
A77.8	Other spotted fevers
A77.9	Spotted fever, unspecified
A78*	Q fever
A79	Other rickettsioses
A79.0	Trench fever
A79.1	Rickettsialpox due to Rickettsia akari
A79.8	Other specified rickettsioses
A79.9	Rickettsiosis, unspecified
B47.1	Actinomycetoma
B95*	Streptococcus and staphylococcus as the cause of diseases classified to other chapters
B96.1	Klebsiella pneumoniae [K. pneumoniae] as the cause of diseases classified to other chapters
B96.2	Escherichia coli [E. coli] as the cause of diseases classified to other chapters
B96.3	Haemophilus influenzae [H. influenzae] as the cause of diseases classified to other chapters
B96.4	Proteus (mirabilis)(morganii) as the cause of diseases classified to other chapters
B96.5	Pseudomonas (aeruginosa) as the cause of diseases classified to other chapters
B96.6	Bacillus fragilis [B. fragilis] as the cause of diseases classified to other chapters

B96.7	Clostridium perfringens [C. perfringens] as the cause of diseases classified to other chapters
B96.8	Other specified bacterial agents as the cause of diseases classified to other chapters
B98.1	Vibrio vulnificus as the cause of diseases classified to other chapters
D73.3	Abscess of spleen
E32.1	Abscess of thymus
G00	Bacterial meningitis, not elsewhere classified
G00.0	Haemophilus meningitis
G00.1	Pneumococcal meningitis
G00.2	Streptococcal meningitis
G00.3	Staphylococcal meningitis
G00.8	Other bacterial meningitis
G00.9	Bacterial meningitis, unspecified
G01*	Meningitis in bacterial diseases classified elsewhere
G04.2	Bacterial meningoencephalitis and meningomyelitis, not elsewhere classified
G05.0	Encephalitis, myelitis and encephalomyelitis in bacterial diseases classified elsewhere
G06*	Intracranial and intraspinal abscess and granuloma
H05.0	Acute inflammation of orbit
H44.0	Purulent endophthalmitis
H60.0	Abscess of external ear
H60.1	Cellulitis of external ear
H60.2	Malignant otitis externa
H70.0	Acute mastoiditis
I32.0	Pericarditis in bacterial diseases classified elsewhere
I33.0	Acute and subacute infective endocarditis
I41.0	Myocarditis in bacterial diseases classified elsewhere
I52.0	Other heart disorders in bacterial diseases classified elsewhere
J05.1	Acute epiglottitis
J13*	Pneumonia due to Streptococcus pneumoniae
J14*	Pneumonia due to Haemophilus influenzae
J15	Bacterial pneumonia, not elsewhere classified
J15.0	Pneumonia due to Klebsiella pneumoniae
J15.1	Pneumonia due to Pseudomonas
J15.2	Pneumonia due to staphylococcus
J15.3	Pneumonia due to streptococcus, group B
J15.4	Pneumonia due to other streptococci
J15.5	Pneumonia due to Escherichia coli
J15.6	Pneumonia due to other aerobic Gram-negative bacteria
J15.7	Pneumonia due to Mycoplasma pneumoniae
J15.8	Other bacterial pneumonia
J15.9	Bacterial pneumonia, unspecified
J16	Pneumonia due to other infectious organisms, not elsewhere classified
J16.0	Chlamydial pneumonia
J16.8	Pneumonia due to other specified infectious organisms
J17.0	Pneumonia in bacterial diseases classified elsewhere
J17.8	Pneumonia in other diseases classified elsewhere
J18*	Pneumonia, organism unspecified
J36*	Peritonsillar abscess
J39.0	Retropharyngeal and parapharyngeal abscess

J39.1	Other abscess of pharynx
J85.1	Abscess of lung with pneumonia
J85.2	Abscess of lung without pneumonia
J85.3	Abscess of mediastinum
J86*	Pyothorax
K12.2	Cellulitis and abscess of mouth
K23.0	Tuberculous oesophagitis
K35*	Acute appendicitis
K57.0	Diverticular disease of small intestine with perforation and abscess
K57.2	Diverticular disease of large intestine with perforation and abscess
K57.4	Diverticular disease of both small and large intestine with perforation and abscess
K57.8	Diverticular disease of intestine, part unspecified, with perforation and abscess
K61*	Abscess of anal and rectal regions
K63.0	Abscess of intestine
K65.0	Acute peritonitis
K67.0	Chlamydial peritonitis
K67.1	Gonococcal peritonitis
K67.2	Syphilitic peritonitis
K67.3	Tuberculous peritonitis
K75.0	Abscess of liver
M00	Pyogenic arthritis
M00.0	Staphylococcal arthritis and polyarthritis
M00.1	Pneumococcal arthritis and polyarthritis
M00.2	Other streptococcal arthritis and polyarthritis
M00.8	Arthritis and polyarthritis due to other specified bacterial agents
M00.9	Pyogenic arthritis, unspecified
M01.0	Meningococcal arthritis
M01.1	Tuberculous arthritis
M01.2	Arthritis in Lyme disease
M01.3	Arthritis in other bacterial diseases classified elsewhere
M46.2	Osteomyelitis of vertebra
M46.3	Infection of intervertebral disc (pyogenic)
M46.5	Other infective spondylopathies
M49.0	Tuberculosis of spine
M49.1	Brucella spondylitis
M49.2	Enterobacterial spondylitis
M63.0	Myositis in bacterial diseases classified elsewhere
M65.0	Abscess of tendon sheath
M65.1	Other infective (teno)synovitis
M71.0	Abscess of bursa
M71.1	Other infective bursitis
M72.6	Necrotizing fasciitis
M73.0	Gonococcal bursitis
M73.1	Syphilitic bursitis
M86*	Osteomyelitis
M86.0	Acute haematogenous osteomyelitis
M86.1	Other acute osteomyelitis
M86.2	Subacute osteomyelitis

M86.3	Chronic multifocal osteomyelitis
M86.4	Chronic osteomyelitis with draining sinus
M86.5	Other chronic haematogenous osteomyelitis
M86.6	Other chronic osteomyelitis
M86.8	Other osteomyelitis
M86.9	Osteomyelitis, unspecified
N10*	Acute tubulo-interstitial nephritis
N13.6	Pyonephrosis
N15.1	Renal and perinephric abscess
N29.0	Late syphilis of kidney
N34.0	Urethral abscess
N41.2	Abscess of prostate
N45*	Orchitis and epididymitis
N61*	Inflammatory disorders of breast
N70.0	Acute salpingitis and oophoritis
N71.0	Acute inflammatory disease of uterus
N73.0	Acute parametritis and pelvic cellulitis
N73.3	Female acute pelvic peritonitis
N74.0	Tuberculous infection of cervix uteri
N74.1	Female tuberculous pelvic inflammatory disease
N74.2	Female syphilitic pelvic inflammatory disease
N74.3	Female gonococcal pelvic inflammatory disease
N74.4	Female chlamydial pelvic inflammatory disease
N75.1	Abscess of Bartholin gland
N76.4	Abscess of vulva
O03.0	Spontaneous abortion: Incomplete, complicated by genital tract and pelvic infection
O03.5	Spontaneous abortion: Complete or unspecified, complicated by genital tract and pelvic infection
O04.0	Medical abortion: Incomplete, complicated by genital tract and pelvic infection
O04.5	Medical abortion: Complete or unspecified, complicated by genital tract and pelvic infection
O05.0	Other abortion: Incomplete, complicated by genital tract and pelvic infection
O05.5	Other abortion: Complete or unspecified, complicated by genital tract and pelvic infection
O06.0	Unspecified abortion: Incomplete, complicated by genital tract and pelvic infection
O06.5	Unspecified abortion: Complete or unspecified, complicated by genital tract and pelvic infection
O07.0	Failed medical abortion, complicated by genital tract and pelvic infection
O07.5	Other and unspecified failed attempted abortion, complicated by genital tract and pelvic infection
O08.0	Genital tract and pelvic infection following abortion and ectopic and molar pregnancy
O23.0	Infections of kidney in pregnancy
O75.3	Other infection during labour
O85*	Puerperal sepsis
O91.1	Abscess of breast associated with childbirth
O98.0	Tuberculosis complicating pregnancy, childbirth and the puerperium
O98.1	Syphilis complicating pregnancy, childbirth and the puerperium
P23	Congenital pneumonia
P23.1	Congenital pneumonia due to Chlamydia
P23.2	Congenital pneumonia due to staphylococcus
P23.3	Congenital pneumonia due to streptococcus, group B
P23.4	Congenital pneumonia due to Escherichia coli
P23.5	Congenital pneumonia due to Pseudomonas

P23.6	Congenital pneumonia due to other bacterial agents
P23.9	Congenital pneumonia, unspecified
P36	Bacterial sepsis of newborn
P36.0	Sepsis of newborn due to streptococcus, group B
P36.1	Sepsis of newborn due to other and unspecified streptococci
P36.2	Sepsis of newborn due to Staphylococcus aureus
P36.3	Sepsis of newborn due to other and unspecified staphylococci
P36.4	Sepsis of newborn due to Escherichia coli
P36.5	Sepsis of newborn due to anaerobes
P36.8	Other bacterial sepsis of newborn
P36.9	Bacterial sepsis of newborn, unspecified
P37.0	Congenital tuberculosis
P37.2	Neonatal (disseminated) listeriosis
P39.0	Neonatal infective mastitis
P39.2	Intra-amniotic infection of fetus, not elsewhere classified
P39.3	Neonatal urinary tract infection

Localised bacterial infections

A00*	Cholera
A04	Other bacterial intestinal infections
A04.0	Enteropathogenic Escherichia coli infection
A04.1	Enterotoxigenic Escherichia coli infection
A04.2	Enteroinvasive Escherichia coli infection
A04.3	Enterohaemorrhagic Escherichia coli infection
A04.4	Other intestinal Escherichia coli infections
A04.5	Campylobacter enteritis
A04.6	Enteritis due to Yersinia enterocolitica
A04.7	Enterocolitis due to Clostridium difficile
A04.8	Other specified bacterial intestinal infections
A04.9	Bacterial intestinal infection, unspecified
A05	Other bacterial foodborne intoxications, not elsewhere classified
A05.0	Foodborne staphylococcal intoxication
A05.1	Botulism
A05.2	Foodborne Clostridium perfringens [Clostridium welchii] intoxication
A05.3	Foodborne Vibrio parahaemolyticus intoxication
A05.4	Foodborne Bacillus cereus intoxication
A05.8	Other specified bacterial foodborne intoxications
A05.9	Bacterial foodborne intoxication, unspecified
A09*	Other gastroenteritis and colitis of infectious and unspecified origin
A26	Erysipeloid
A26.0	Cutaneous erysipeloid
A26.9	Erysipeloid, unspecified
A37*	Whooping cough
A48.2	Nonpneumonic Legionnaires disease [Pontiac fever]
A54.0	Gonococcal infection of lower genitourinary tract without periurethral or accessory gland abscess

A54.3	Gonococcal infection of eye
A54.5	Gonococcal pharyngitis
A54.6	Gonococcal infection of anus and rectum
A56.0	Chlamydial infection of lower genitourinary tract
A56.3	Chlamydial infection of anus and rectum
A56.4	Chlamydial infection of pharynx
A57*	Chancroid
A58*	Granuloma inguinale
A64*	Unspecified sexually transmitted disease
A71*	Trachoma
A74.0	Chlamydial conjunctivitis
B98.0	Helicobacter pylori [H.pylori] as the cause of diseases classified to other chapters
H00*	Hordeolum and chalazion
H01.0	Blepharitis
H10.0	Mucopurulent conjunctivitis
H10.5	Blepharoconjunctivitis
H60.3	Other infective otitis externa
H66.0	Acute suppurative otitis media
H67.0	Otitis media in bacterial diseases classified elsewhere
J01.0	Acute maxillary sinusitis
J02	Acute pharyngitis
J02.0	Streptococcal pharyngitis
J02.8	Acute pharyngitis due to other specified organisms
J02.9	Acute pharyngitis, unspecified
J03	Acute tonsillitis
J03.0	Streptococcal tonsillitis
J03.8	Acute tonsillitis due to other specified organisms
J03.9	Acute tonsillitis, unspecified
J04*	Acute laryngitis and tracheitis
J20.0	Acute bronchitis due to Mycoplasma pneumoniae
J20.1	Acute bronchitis due to Haemophilus influenzae
J20.2	Acute bronchitis due to streptococcus
J22*	Unspecified acute lower respiratory infection
K02*	Dental caries
K04.4	Acute apical periodontitis of pulpal origin
K04.5	Chronic apical periodontitis
K04.6	Periapical abscess with sinus
K04.7	Periapical abscess without sinus
K05.0	Acute gingivitis
K05.2	Acute periodontitis
K05.3	Chronic periodontitis
K05.4	Periodontosis
K11.3	Abscess of salivary gland
L00*	Staphylococcal scalded skin syndrome
L01*	Impetigo
L02*	Cutaneous abscess, furuncle and carbuncle
L03*	Cellulitis
L04*	Acute lymphadenitis

L05*	Pilonidal cyst
L08*	Other local infections of skin and subcutaneous tissue
L70.1	Acne conglobata
N30.0	Acute cystitis
N39.0	Urinary tract infection, site not specified
N41.0	Acute prostatitis
N76.0	Acute vaginitis
O23.1	Infections of bladder in pregnancy
O23.2	Infections of urethra in pregnancy
O23.3	Infections of other parts of urinary tract in pregnancy
O23.4	Unspecified infection of urinary tract in pregnancy
O23.5	Infections of the genital tract in pregnancy
O23.9	Other and unspecified genitourinary tract infection in pregnancy
O86*	Other puerperal infections
O91.0	Infection of nipple associated with childbirth
O98.2	Gonorrhoea complicating pregnancy, childbirth and the puerperium
O98.3	Other infections with a predominantly sexual mode of transmission complicating pregnancy, childbirth and the puerperium
O98.8	Other maternal infectious and parasitic diseases complicating pregnancy, childbirth and the puerperium
O98.9	Unspecified maternal infectious or parasitic disease complicating pregnancy, childbirth and the puerperium
P38*	Omphalitis of newborn with or without mild haemorrhage
P39.1	Neonatal conjunctivitis and dacryocystitis
P39.4	Neonatal skin infection

Bacterial infections with sepsis

A02.1	Salmonella sepsis
A20.7	Septicaemic plague
A21.7	Generalized tularaemia
A22.7	Anthrax sepsis
A32.7	Listerial sepsis
A39.1	Waterhouse-Friderichsen syndrome
A39.2	Acute meningococcaemia
A39.3	Chronic meningococcaemia
A39.4	Meningococcaemia, unspecified
A40	Streptococcal sepsis
A40.0	Sepsis due to streptococcus, group A
A40.1	Sepsis due to streptococcus, group B
A40.2	Sepsis due to streptococcus, group D
A40.3	Sepsis due to Streptococcus pneumoniae
A40.8	Other streptococcal sepsis
A40.9	Streptococcal sepsis, unspecified
A41	Other sepsis
A41.0	Sepsis due to Staphylococcus aureus
A41.1	Sepsis due to other specified staphylococcus
A41.2	Sepsis due to unspecified staphylococcus
A41.3	Sepsis due to Haemophilus influenzae

A41.4	Sepsis due to anaerobes
A41.5	Sepsis due to other Gram-negative organisms
A41.8	Other specified sepsis
A41.9	Sepsis, unspecified
A42.7	Actinomycotic sepsis
A44.0	Systemic bartonellosis
A48.3	Toxic shock syndrome
A48.4	Brazilian purpuric fever
P36	Bacterial sepsis of newborn
P36.0	Sepsis of newborn due to streptococcus, group B
P36.1	Sepsis of newborn due to other and unspecified streptococci
P36.2	Sepsis of newborn due to Staphylococcus aureus
P36.3	Sepsis of newborn due to other and unspecified staphylococci
P36.4	Sepsis of newborn due to Escherichia coli
P36.5	Sepsis of newborn due to anaerobes
P36.8	Other bacterial sepsis of newborn
P36.9	Bacterial sepsis of newborn, unspecified
P37.2	Neonatal (disseminated) listeriosis

Bacterial infections without sepsis

A00*	Cholera
A01*	Typhoid and paratyphoid fevers
A02	Other salmonella infections
A02.0	Salmonella enteritis
A02.2	Localized salmonella infections
A02.8	Other specified salmonella infections
A02.9	Salmonella infection, unspecified
A03	Shigellosis
A03.0	Shigellosis due to Shigella dysenteriae
A03.1	Shigellosis due to Shigella flexneri
A03.2	Shigellosis due to Shigella boydii
A03.3	Shigellosis due to Shigella sonnei
A03.8	Other shigellosis
A03.9	Shigellosis, unspecified
A04	Other bacterial intestinal infections
A04.0	Enteropathogenic Escherichia coli infection
A04.1	Enterotoxigenic Escherichia coli infection
A04.2	Enteroinvasive Escherichia coli infection
A04.3	Enterohaemorrhagic Escherichia coli infection
A04.4	Other intestinal Escherichia coli infections
A04.5	Campylobacter enteritis
A04.6	Enteritis due to Yersinia enterocolitica
A04.7	Enterocolitis due to Clostridium difficile
A04.8	Other specified bacterial intestinal infections
A04.9	Bacterial intestinal infection, unspecified

A05	Other bacterial foodborne intoxications, not elsewhere classified
A05.0	Foodborne staphylococcal intoxication
A05.1	Botulism
A05.2	Foodborne Clostridium perfringens [Clostridium welchii] intoxication
A05.3	Foodborne Vibrio parahaemolyticus intoxication
A05.4	Foodborne Bacillus cereus intoxication
A05.8	Other specified bacterial foodborne intoxications
A05.9	Bacterial foodborne intoxication, unspecified
A09*	Other gastroenteritis and colitis of infectious and unspecified origin
A15*	Respiratory tuberculosis, bacteriologically and histologically confirmed
A16*	Respiratory tuberculosis, not confirmed bacteriologically or histologically
A17	Tuberculosis of nervous system
A17.0	Tuberculous meningitis
A17.1	Meningeal tuberculoma
A17.8	Other tuberculosis of nervous system
A17.9	Tuberculosis of nervous system, unspecified
A18*	Tuberculosis of other organs
A19*	Miliary tuberculosis
A20	Plague
A20.0	Bubonic plague
A20.1	Cellulocutaneous plague
A20.2	Pneumonic plague
A20.3	Plague meningitis
A20.8	Other forms of plague
A20.9	Plague, unspecified
A21	Tularaemia
A21.0	Ulceroglandular tularaemia
A21.1	Oculoglandular tularaemia
A21.2	Pulmonary tularaemia
A21.3	Gastrointestinal tularaemia
A21.8	Other forms of tularaemia
A21.9	Tularaemia, unspecified
A22	Anthrax
A22.0	Cutaneous anthrax
A22.1	Pulmonary anthrax
A22.2	Gastrointestinal anthrax
A22.8	Other forms of anthrax
A22.9	Anthrax, unspecified
A23	Brucellosis
A23.0	Brucellosis due to Brucella melitensis
A23.1	Brucellosis due to Brucella abortus
A23.2	Brucellosis due to Brucella suis
A23.3	Brucellosis due to Brucella canis
A23.8	Other brucellosis
A23.9	Brucellosis, unspecified
A24	Glanders and melioidosis
A24.0	Glanders
A24.1	Acute and fulminating melioidosis

A24.2	Subacute and chronic melioidosis
A24.3	Other melioidosis
A25	Rat-bite fevers
A25.0	Spirillosis
A25.1	Streptobacillosis
A25.9	Rat-bite fever, unspecified
A26	Erysipeloid
A26.0	Cutaneous erysipeloid
A26.7	Erysipelothrix sepsis
A26.8	Other forms of erysipeloid
A26.9	Erysipeloid, unspecified
A27*	Leptospirosis
A28	Other zoonotic bacterial diseases, not elsewhere classified
A28.0	Pasteurellosis
A28.1	Cat-scratch disease
A28.2	Extraintestinal yersiniosis
A28.8	Other specified zoonotic bacterial diseases, not elsewhere classified
A28.9	Zoonotic bacterial disease, unspecified
A30*	Leprosy [Hansen disease]
A31*	Infection due to other mycobacteria
A32	Listeriosis
A32.0	Cutaneous listeriosis
A32.1	Listerial meningitis and meningoencephalitis
A32.8	Other forms of listeriosis
A32.9	Listeriosis, unspecified
A33*	Tetanus neonatorum
A34*	Obstetrical tetanus
A35*	Other tetanus
A36*	Diphtheria
A37*	Whooping cough
A38*	Scarlet fever
A39	Meningococcal infection
A39.0	Meningococcal meningitis
A39.5	Meningococcal heart disease
A39.8	Other meningococcal infections
A39.9	Meningococcal infection, unspecified
A42	Actinomycosis
A42.0	Pulmonary actinomycosis
A42.1	Abdominal actinomycosis
A42.2	Cervicofacial actinomycosis
A42.8	Other forms of actinomycosis
A42.9	Actinomycosis, unspecified
A43	Nocardiosis
A43.0	Pulmonary nocardiosis
A43.1	Cutaneous nocardiosis
A43.8	Other forms of nocardiosis
A44	Bartonellosis
A44.1	Cutaneous and mucocutaneous bartonellosis

A44.8	Other forms of bartonellosis
A44.9	Bartonellosis, unspecified
A46*	Erysipelas
A48.0	Gas gangrene
A48.1	Legionnaires disease
A48.2	Nonpneumonic Legionnaires disease [Pontiac fever]
A48.8	Other specified bacterial diseases
A49	Bacterial infection of unspecified site
A49.0	Staphylococcal infection, unspecified site
A49.1	Streptococcal infection, unspecified site
A49.2	Haemophilus influenzae infection, unspecified site
A49.3	Mycoplasma infection, unspecified site
A49.8	Other bacterial infections of unspecified site
A49.9	Bacterial infection, unspecified
A50	Congenital syphilis
A50.0	Early congenital syphilis, symptomatic
A50.1	Early congenital syphilis, latent
A50.2	Early congenital syphilis, unspecified
A50.3	Late congenital syphilitic oculopathy
A50.4	Late congenital neurosyphilis [juvenile neurosyphilis]
A50.5	Other late congenital syphilis, symptomatic
A50.6	Late congenital syphilis, latent
A50.7	Late congenital syphilis, unspecified
A50.9	Congenital syphilis, unspecified
A51	Early syphilis
A51.0	Primary genital syphilis
A51.1	Primary anal syphilis
A51.2	Primary syphilis of other sites
A51.3	Secondary syphilis of skin and mucous membranes
A51.4	Other secondary syphilis
A51.5	Early syphilis, latent
A51.9	Early syphilis, unspecified
A52	Late syphilis
A52.0	Cardiovascular syphilis
A52.1	Symptomatic neurosyphilis
A52.2	Asymptomatic neurosyphilis
A52.3	Neurosyphilis, unspecified
A52.7	Other symptomatic late syphilis
A52.8	Late syphilis, latent
A52.9	Late syphilis, unspecified
A53*	Other and unspecified syphilis
A54	Gonococcal infection
A54.0	Gonococcal infection of lower genitourinary tract without periurethral or accessory gland abscess
A54.1	Gonococcal infection of lower genitourinary tract with periurethral and accessory gland abscess
A54.2	Gonococcal pelviperitonitis and other gonococcal genitourinary infections
A54.3	Gonococcal infection of eye
A54.4	Gonococcal infection of musculoskeletal system
A54.5	Gonococcal pharyngitis

A54.6	Gonococcal infection of anus and rectum
A54.8	Other gonococcal infections
A54.9	Gonococcal infection, unspecified
A55*	Chlamydial lymphogranuloma (venereum)
A56.0	Chlamydial infection of lower genitourinary tract
A56.1	Chlamydial infection of pelviperitoneum and other genitourinary organs
A56.2	Chlamydial infection of genitourinary tract, unspecified
A56.3	Chlamydial infection of anus and rectum
A56.4	Chlamydial infection of pharynx
A56.8	Sexually transmitted chlamydial infection of other sites
A57*	Chancroid
A58*	Granuloma inguinale
A64*	Unspecified sexually transmitted disease
A65*	Nonvenereal syphilis
A66*	Yaws
A67*	Pinta [carate]
A68*	Relapsing fevers
A69	Other spirochaetal infections
A69.0	Necrotizing ulcerative stomatitis
A69.1	Other Vincent infections
A69.2	Lyme disease
A69.8	Other specified spirochaetal infections
A69.9	Spirochaetal infection, unspecified
A70*	Chlamydia psittaci infection
A71*	Trachoma
A74.0	Chlamydial conjunctivitis
A74.8	Other chlamydial diseases
A74.9	Chlamydial infection, unspecified
A75	Typhus fever
A75.0	Epidemic louse-borne typhus fever due to Rickettsia prowazekii
A75.1	Recrudescent typhus [Brill disease]
A75.2	Typhus fever due to Rickettsia typhi
A75.3	Typhus fever due to Rickettsia tsutsugamushi
A75.9	Typhus fever, unspecified
A77	Spotted fever [tick-borne rickettsioses]
A77.0	Spotted fever due to Rickettsia rickettsii
A77.1	Spotted fever due to Rickettsia conorii
A77.2	Spotted fever due to Rickettsia sibirica
A77.3	Spotted fever due to Rickettsia australis
A77.8	Other spotted fevers
A77.9	Spotted fever, unspecified
A78*	Q fever
A79	Other rickettsioses
A79.0	Trench fever
A79.1	Rickettsialpox due to Rickettsia akari
A79.8	Other specified rickettsioses
A79.9	Rickettsiosis, unspecified
B47.1	Actinomycetoma

B95*	Streptococcus and staphylococcus as the cause of diseases classified to other chapters
B96.0	Mycoplasma pneumoniae [M. pneumoniae] as the cause of diseases classified to other chapters
B96.1	Klebsiella pneumoniae [K. pneumoniae] as the cause of diseases classified to other chapters
B96.2	Escherichia coli [E. coli] as the cause of diseases classified to other chapters
B96.3	Haemophilus influenzae [H. influenzae] as the cause of diseases classified to other chapters
B96.4	Proteus (mirabilis)(morganii) as the cause of diseases classified to other chapters
B96.5	Pseudomonas (aeruginosa) as the cause of diseases classified to other chapters
B96.6	Bacillus fragilis [B. fragilis] as the cause of diseases classified to other chapters
B96.7	Clostridium perfringens [C. perfringens] as the cause of diseases classified to other chapters
B96.8	Other specified bacterial agents as the cause of diseases classified to other chapters
B98.0	Helicobacter pylori [H.pylori] as the cause of diseases classified to other chapters
B98.1	Vibrio vulnificus as the cause of diseases classified to other chapters
D73.3	Abscess of spleen
E32.1	Abscess of thymus
G00	Bacterial meningitis, not elsewhere classified
G00.0	Haemophilus meningitis
G00.1	Pneumococcal meningitis
G00.2	Streptococcal meningitis
G00.3	Staphylococcal meningitis
G00.8	Other bacterial meningitis
G00.9	Bacterial meningitis, unspecified
G01*	Meningitis in bacterial diseases classified elsewhere
G04.2	Bacterial meningoencephalitis and meningomyelitis, not elsewhere classified
G05.0	Encephalitis, myelitis and encephalomyelitis in bacterial diseases classified elsewhere
G06*	Intracranial and intraspinal abscess and granuloma
H00*	Hordeolum and chalazion
H01.0	Blepharitis
H05.0	Acute inflammation of orbit
H10.0	Mucopurulent conjunctivitis
H10.5	Blepharoconjunctivitis
H44.0	Purulent endophthalmitis
H60.0	Abscess of external ear
H60.1	Cellulitis of external ear
H60.2	Malignant otitis externa
H60.3	Other infective otitis externa
H62.0	Otitis externa in bacterial diseases classified elsewhere
H66.0	Acute suppurative otitis media
H67.0	Otitis media in bacterial diseases classified elsewhere
H70.0	Acute mastoiditis
I32.0	Pericarditis in bacterial diseases classified elsewhere
I33.0	Acute and subacute infective endocarditis
I41.0	Myocarditis in bacterial diseases classified elsewhere
I52.0	Other heart disorders in bacterial diseases classified elsewhere
J01.0	Acute maxillary sinusitis
J02	Acute pharyngitis
J02.0	Streptococcal pharyngitis
J02.8	Acute pharyngitis due to other specified organisms
J02.9	Acute pharyngitis, unspecified

J03	Acute tonsillitis
J03.0	Streptococcal tonsillitis
J03.8	Acute tonsillitis due to other specified organisms
J03.9	Acute tonsillitis, unspecified
J04*	Acute laryngitis and tracheitis
J05.1	Acute epiglottitis
J13*	Pneumonia due to Streptococcus pneumoniae
J14*	Pneumonia due to Haemophilus influenzae
J15	Bacterial pneumonia, not elsewhere classified
J15.0	Pneumonia due to Klebsiella pneumoniae
J15.1	Pneumonia due to Pseudomonas
J15.2	Pneumonia due to staphylococcus
J15.3	Pneumonia due to streptococcus, group B
J15.4	Pneumonia due to other streptococci
J15.5	Pneumonia due to Escherichia coli
J15.6	Pneumonia due to other aerobic Gram-negative bacteria
J15.7	Pneumonia due to Mycoplasma pneumoniae
J15.8	Other bacterial pneumonia
J15.9	Bacterial pneumonia, unspecified
J16	Pneumonia due to other infectious organisms, not elsewhere classified
J16.0	Chlamydial pneumonia
J16.8	Pneumonia due to other specified infectious organisms
J17.0	Pneumonia in bacterial diseases classified elsewhere
J17.8	Pneumonia in other diseases classified elsewhere
J18*	Pneumonia, organism unspecified
J20.0	Acute bronchitis due to Mycoplasma pneumoniae
J20.1	Acute bronchitis due to Haemophilus influenzae
J20.2	Acute bronchitis due to streptococcus
J22*	Unspecified acute lower respiratory infection
J36*	Peritonsillar abscess
J39.0	Retropharyngeal and parapharyngeal abscess
J39.1	Other abscess of pharynx
J85.1	Abscess of lung with pneumonia
J85.2	Abscess of lung without pneumonia
J85.3	Abscess of mediastinum
J86*	Pyothorax
K02*	Dental caries
K04.4	Acute apical periodontitis of pulpal origin
K04.5	Chronic apical periodontitis
K04.6	Periapical abscess with sinus
K04.7	Periapical abscess without sinus
K05.0	Acute gingivitis
K05.2	Acute periodontitis
K05.3	Chronic periodontitis
K05.4	Periodontosis
K11.3	Abscess of salivary gland
K12.2	Cellulitis and abscess of mouth
K23.0	Tuberculous oesophagitis

K35*	Acute appendicitis
K57.0	Diverticular disease of small intestine with perforation and abscess
K57.2	Diverticular disease of large intestine with perforation and abscess
K57.4	Diverticular disease of both small and large intestine with perforation and abscess
K57.8	Diverticular disease of intestine, part unspecified, with perforation and abscess
K61*	Abscess of anal and rectal regions
K63.0	Abscess of intestine
K65.0	Acute peritonitis
K67.0	Chlamydial peritonitis
K67.1	Gonococcal peritonitis
K67.2	Syphilitic peritonitis
K67.3	Tuberculous peritonitis
K75.0	Abscess of liver
L00*	Staphylococcal scalded skin syndrome
L01*	Impetigo
L02*	Cutaneous abscess, furuncle and carbuncle
L03*	Cellulitis
L04*	Acute lymphadenitis
L05*	Pilonidal cyst
L08*	Other local infections of skin and subcutaneous tissue
L70.1	Acne conglobata
M00	Pyogenic arthritis
M00.0	Staphylococcal arthritis and polyarthritis
M00.1	Pneumococcal arthritis and polyarthritis
M00.2	Other streptococcal arthritis and polyarthritis
M00.8	Arthritis and polyarthritis due to other specified bacterial agents
M00.9	Pyogenic arthritis, unspecified
M01.0	Meningococcal arthritis
M01.1	Tuberculous arthritis
M01.2	Arthritis in Lyme disease
M01.3	Arthritis in other bacterial diseases classified elsewhere
M46.2	Osteomyelitis of vertebra
M46.3	Infection of intervertebral disc (pyogenic)
M46.5	Other infective spondylopathies
M49.0	Tuberculosis of spine
M49.1	Brucella spondylitis
M49.2	Enterobacterial spondylitis
M63.0	Myositis in bacterial diseases classified elsewhere
M65.0	Abscess of tendon sheath
M65.1	Other infective (teno)synovitis
M71.0	Abscess of bursa
M71.1	Other infective bursitis
M72.6	Necrotizing fasciitis
M73.0	Gonococcal bursitis
M73.1	Syphilitic bursitis
M86*	Osteomyelitis
M86.0	Acute haematogenous osteomyelitis
M86.1	Other acute osteomyelitis

M86.2	Subacute osteomyelitis
M86.3	Chronic multifocal osteomyelitis
M86.4	Chronic osteomyelitis with draining sinus
M86.5	Other chronic haematogenous osteomyelitis
M86.6	Other chronic osteomyelitis
M86.8	Other osteomyelitis
M86.9	Osteomyelitis, unspecified
N10*	Acute tubulo-interstitial nephritis
N13.6	Pyonephrosis
N15.1	Renal and perinephric abscess
N29.0	Late syphilis of kidney
N30.0	Acute cystitis
N34.0	Urethral abscess
N39.0	Urinary tract infection, site not specified
N41.0	Acute prostatitis
N41.2	Abscess of prostate
N45*	Orchitis and epididymitis
N61*	Inflammatory disorders of breast
N70.0	Acute salpingitis and oophoritis
N71.0	Acute inflammatory disease of uterus
N73.0	Acute parametritis and pelvic cellulitis
N73.3	Female acute pelvic peritonitis
N74.0	Tuberculous infection of cervix uteri
N74.1	Female tuberculous pelvic inflammatory disease
N74.2	Female syphilitic pelvic inflammatory disease
N74.3	Female gonococcal pelvic inflammatory disease
N74.4	Female chlamydial pelvic inflammatory disease
N75.1	Abscess of Bartholin gland
N76.0	Acute vaginitis
N76.4	Abscess of vulva
O03.0	Spontaneous abortion: Incomplete, complicated by genital tract and pelvic infection
O03.5	Spontaneous abortion: Complete or unspecified, complicated by genital tract and pelvic infection
O04.0	Medical abortion: Incomplete, complicated by genital tract and pelvic infection
O04.5	Medical abortion: Complete or unspecified, complicated by genital tract and pelvic infection
O05.0	Other abortion: Incomplete, complicated by genital tract and pelvic infection
O05.5	Other abortion: Complete or unspecified, complicated by genital tract and pelvic infection
O06.0	Unspecified abortion: Incomplete, complicated by genital tract and pelvic infection
O06.5	Unspecified abortion: Complete or unspecified, complicated by genital tract and pelvic infection
O07.0	Failed medical abortion, complicated by genital tract and pelvic infection
O07.5	Other and unspecified failed attempted abortion, complicated by genital tract and pelvic infection
O08.0	Genital tract and pelvic infection following abortion and ectopic and molar pregnancy
O23.0	Infections of kidney in pregnancy
O23.1	Infections of bladder in pregnancy
O23.2	Infections of urethra in pregnancy
O23.3	Infections of other parts of urinary tract in pregnancy
O23.4	Unspecified infection of urinary tract in pregnancy
O23.5	Infections of the genital tract in pregnancy
O23.9	Other and unspecified genitourinary tract infection in pregnancy

O75.3	Other infection during labour
O85*	Puerperal sepsis
O86*	Other puerperal infections
O91.0	Infection of nipple associated with childbirth
O91.1	Abscess of breast associated with childbirth
O98.0	Tuberculosis complicating pregnancy, childbirth and the puerperium
O98.1	Syphilis complicating pregnancy, childbirth and the puerperium
O98.2	Gonorrhoea complicating pregnancy, childbirth and the puerperium
O98.3	Other infections with a predominantly sexual mode of transmission complicating pregnancy, childbirth and the puerperium
O98.8	Other maternal infectious and parasitic diseases complicating pregnancy, childbirth and the puerperium
O98.9	Unspecified maternal infectious or parasitic disease complicating pregnancy, childbirth and the puerperium
P23	Congenital pneumonia
P23.1	Congenital pneumonia due to Chlamydia
P23.2	Congenital pneumonia due to staphylococcus
P23.3	Congenital pneumonia due to streptococcus, group B
P23.4	Congenital pneumonia due to Escherichia coli
P23.5	Congenital pneumonia due to Pseudomonas
P23.6	Congenital pneumonia due to other bacterial agents
P23.9	Congenital pneumonia, unspecified
P37.0	Congenital tuberculosis
P38*	Omphalitis of newborn with or without mild haemorrhage
P39.0	Neonatal infective mastitis
P39.1	Neonatal conjunctivitis and dacryocystitis
P39.2	Intra-amniotic infection of fetus, not elsewhere classified
P39.3	Neonatal urinary tract infection
P39.4	Neonatal skin infection

Intracellular bacterial infections

A01*	Typhoid and paratyphoid fevers
A02	Other salmonella infections
A02.0	Salmonella enteritis
A02.1	Salmonella sepsis
A02.2	Localized salmonella infections
A02.8	Other specified salmonella infections
A02.9	Salmonella infection, unspecified
A03	Shigellosis
A03.0	Shigellosis due to Shigella dysenteriae
A03.1	Shigellosis due to Shigella flexneri
A03.2	Shigellosis due to Shigella boydii
A03.3	Shigellosis due to Shigella sonnei
A03.8	Other shigellosis
A03.9	Shigellosis, unspecified
A04.2	Enteroinvasive Escherichia coli infection
A04.5	Campylobacter enteritis
A04.6	Enteritis due to Yersinia enterocolitica

A15*	Respiratory tuberculosis, bacteriologically and histologically confirmed
A16*	Respiratory tuberculosis, not confirmed bacteriologically or histologically
A17	Tuberculosis of nervous system
A17.0	Tuberculous meningitis
A17.1	Meningeal tuberculoma
A17.8	Other tuberculosis of nervous system
A17.9	Tuberculosis of nervous system, unspecified
A18*	Tuberculosis of other organs
A19*	Miliary tuberculosis
A20	Plague
A20.0	Bubonic plague
A20.1	Cellulocutaneous plague
A20.2	Pneumonic plague
A20.3	Plague meningitis
A20.7	Septicaemic plague
A20.8	Other forms of plague
A20.9	Plague, unspecified
A21	Tularaemia
A21.0	Ulceroglandular tularaemia
A21.1	Oculoglandular tularaemia
A21.2	Pulmonary tularaemia
A21.3	Gastrointestinal tularaemia
A21.7	Generalized tularaemia
A21.8	Other forms of tularaemia
A21.9	Tularaemia, unspecified
A23	Brucellosis
A23.0	Brucellosis due to Brucella melitensis
A23.1	Brucellosis due to Brucella abortus
A23.2	Brucellosis due to Brucella suis
A23.3	Brucellosis due to Brucella canis
A23.8	Other brucellosis
A23.9	Brucellosis, unspecified
A24	Glanders and melioidosis
A24.0	Glanders
A24.1	Acute and fulminating melioidosis
A24.2	Subacute and chronic melioidosis
A24.3	Other melioidosis
A26	Erysipeloid
A26.0	Cutaneous erysipeloid
A26.7	Erysipelothrix sepsis
A26.8	Other forms of erysipeloid
A26.9	Erysipeloid, unspecified
A28.1	Cat-scratch disease
A28.2	Extraintestinal yersiniosis
A30*	Leprosy [Hansen disease]
A31*	Infection due to other mycobacteria
A32	Listeriosis
A32.0	Cutaneous listeriosis

A32.1	Listerial meningitis and meningoencephalitis
A32.7	Listerial sepsis
A32.8	Other forms of listeriosis
A32.9	Listeriosis, unspecified
A43	Nocardiosis
A43.0	Pulmonary nocardiosis
A43.1	Cutaneous nocardiosis
A43.8	Other forms of nocardiosis
A44	Bartonellosis
A44.0	Systemic bartonellosis
A44.1	Cutaneous and mucocutaneous bartonellosis
A44.8	Other forms of bartonellosis
A44.9	Bartonellosis, unspecified
A48.1	Legionnaires disease
A48.2	Nonpneumonic Legionnaires disease [Pontiac fever]
A49.3	Mycoplasma infection, unspecified site
A55*	Chlamydial lymphogranuloma (venereum)
A56.0	Chlamydial infection of lower genitourinary tract
A56.1	Chlamydial infection of pelviperitoneum and other genitourinary organs
A56.2	Chlamydial infection of genitourinary tract, unspecified
A56.3	Chlamydial infection of anus and rectum
A56.4	Chlamydial infection of pharynx
A56.8	Sexually transmitted chlamydial infection of other sites
A58*	Granuloma inguinale
A70*	Chlamydia psittaci infection
A71*	Trachoma
A74.0	Chlamydial conjunctivitis
A74.8	Other chlamydial diseases
A74.9	Chlamydial infection, unspecified
A75	Typhus fever
A75.0	Epidemic louse-borne typhus fever due to Rickettsia prowazekii
A75.1	Recrudescent typhus [Brill disease]
A75.2	Typhus fever due to Rickettsia typhi
A75.3	Typhus fever due to Rickettsia tsutsugamushi
A75.9	Typhus fever, unspecified
A77	Spotted fever [tick-borne rickettsioses]
A77.0	Spotted fever due to Rickettsia rickettsii
A77.1	Spotted fever due to Rickettsia conorii
A77.2	Spotted fever due to Rickettsia sibirica
A77.3	Spotted fever due to Rickettsia australis
A77.8	Other spotted fevers
A77.9	Spotted fever, unspecified
A78*	Q fever
A79	Other rickettsioses
A79.0	Trench fever
A79.1	Rickettsialpox due to Rickettsia akari
A79.8	Other specified rickettsioses
A79.9	Rickettsiosis, unspecified

B96.0	Mycoplasma pneumoniae [M. pneumoniae] as the cause of diseases classified to other chapters
J15.7	Pneumonia due to Mycoplasma pneumoniae
J16.0	Chlamydial pneumonia
J20.0	Acute bronchitis due to Mycoplasma pneumoniae
K23.0	Tuberculous oesophagitis
K67.0	Chlamydial peritonitis
K67.3	Tuberculous peritonitis
M01.1	Tuberculous arthritis
M49.0	Tuberculosis of spine
M49.1	Brucella spondylitis
N74.0	Tuberculous infection of cervix uteri
N74.1	Female tuberculous pelvic inflammatory disease
N74.4	Female chlamydial pelvic inflammatory disease
O98.0	Tuberculosis complicating pregnancy, childbirth and the puerperium
P23.1	Congenital pneumonia due to Chlamydia
P37.0	Congenital tuberculosis
P37.2	Neonatal (disseminated) listeriosis

Extracellular bacterial infections

A00*	Cholera
A04.0	Enteropathogenic Escherichia coli infection
A04.1	Enterotoxigenic Escherichia coli infection
A04.3	Enterohaemorrhagic Escherichia coli infection
A04.4	Other intestinal Escherichia coli infections
A04.7	Enterocolitis due to Clostridium difficile
A05.0	Foodborne staphylococcal intoxication
A05.1	Botulism
A05.2	Foodborne Clostridium perfringens [Clostridium welchii] intoxication
A05.3	Foodborne Vibrio parahaemolyticus intoxication
A05.4	Foodborne Bacillus cereus intoxication
A22	Anthrax
A22.0	Cutaneous anthrax
A22.1	Pulmonary anthrax
A22.2	Gastrointestinal anthrax
A22.7	Anthrax sepsis
A22.8	Other forms of anthrax
A22.9	Anthrax, unspecified
A25	Rat-bite fevers
A25.0	Spirillosis
A25.1	Streptobacillosis
A25.9	Rat-bite fever, unspecified
A27*	Leptospirosis
A28.0	Pasteurellosis
A33*	Tetanus neonatorum
A34*	Obstetrical tetanus

A35*	Other tetanus
A36*	Diphtheria
A37*	Whooping cough
A38*	Scarlet fever
A39	Meningococcal infection
A39.0	Meningococcal meningitis
A39.1	Waterhouse-Friderichsen syndrome
A39.2	Acute meningococcaemia
A39.3	Chronic meningococcaemia
A39.4	Meningococcaemia, unspecified
A39.5	Meningococcal heart disease
A39.8	Other meningococcal infections
A39.9	Meningococcal infection, unspecified
A40	Streptococcal sepsis
A40.0	Sepsis due to streptococcus, group A
A40.1	Sepsis due to streptococcus, group B
A40.2	Sepsis due to streptococcus, group D
A40.3	Sepsis due to Streptococcus pneumoniae
A40.8	Other streptococcal sepsis
A40.9	Streptococcal sepsis, unspecified
A41.0	Sepsis due to Staphylococcus aureus
A41.1	Sepsis due to other specified staphylococcus
A41.2	Sepsis due to unspecified staphylococcus
A41.3	Sepsis due to Haemophilus influenzae
A48.0	Gas gangrene
A48.3	Toxic shock syndrome
A48.4	Brazilian purpuric fever
A49.0	Staphylococcal infection, unspecified site
A49.2	Haemophilus influenzae infection, unspecified site
A50	Congenital syphilis
A50.0	Early congenital syphilis, symptomatic
A50.1	Early congenital syphilis, latent
A50.2	Early congenital syphilis, unspecified
A50.3	Late congenital syphilitic oculopathy
A50.4	Late congenital neurosyphilis [juvenile neurosyphilis]
A50.5	Other late congenital syphilis, symptomatic
A50.6	Late congenital syphilis, latent
A50.7	Late congenital syphilis, unspecified
A50.9	Congenital syphilis, unspecified
A51	Early syphilis
A51.0	Primary genital syphilis
A51.1	Primary anal syphilis
A51.2	Primary syphilis of other sites
A51.3	Secondary syphilis of skin and mucous membranes
A51.4	Other secondary syphilis
A51.5	Early syphilis, latent
A51.9	Early syphilis, unspecified
A52	Late syphilis

A52.0	Cardiovascular syphilis
A52.1	Symptomatic neurosyphilis
A52.2	Asymptomatic neurosyphilis
A52.3	Neurosyphilis, unspecified
A52.7	Other symptomatic late syphilis
A52.8	Late syphilis, latent
A52.9	Late syphilis, unspecified
A53*	Other and unspecified syphilis
A54	Gonococcal infection
A54.0	Gonococcal infection of lower genitourinary tract without periurethral or accessory gland abscess
A54.1	Gonococcal infection of lower genitourinary tract with periurethral and accessory gland abscess
A54.2	Gonococcal pelviperitonitis and other gonococcal genitourinary infections
A54.3	Gonococcal infection of eye
A54.4	Gonococcal infection of musculoskeletal system
A54.5	Gonococcal pharyngitis
A54.6	Gonococcal infection of anus and rectum
A54.8	Other gonococcal infections
A54.9	Gonococcal infection, unspecified
A57*	Chancroid
A65*	Nonvenereal syphilis
A66*	Yaws
A67*	Pinta [carate]
A68*	Relapsing fevers
A69.2	Lyme disease
A69.8	Other specified spirochaetal infections
A69.9	Spirochaetal infection, unspecified
B95*	Streptococcus and staphylococcus as the cause of diseases classified to other chapters
B96.1	Klebsiella pneumoniae [K. pneumoniae] as the cause of diseases classified to other chapters
B96.2	Escherichia coli [E. coli] as the cause of diseases classified to other chapters
B96.3	Haemophilus influenzae [H. influenzae] as the cause of diseases classified to other chapters
B96.4	Proteus (mirabilis)(morganii) as the cause of diseases classified to other chapters
B96.5	Pseudomonas (aeruginosa) as the cause of diseases classified to other chapters
B96.6	Bacillus fragilis [B. fragilis] as the cause of diseases classified to other chapters
B96.7	Clostridium perfringens [C. perfringens] as the cause of diseases classified to other chapters
B98.0	Helicobacter pylori [H.pylori] as the cause of diseases classified to other chapters
B98.1	Vibrio vulnificus as the cause of diseases classified to other chapters
G00.0	Haemophilus meningitis
G00.1	Pneumococcal meningitis
G00.2	Streptococcal meningitis
G00.3	Staphylococcal meningitis
J02.0	Streptococcal pharyngitis
J03.0	Streptococcal tonsillitis
J13*	Pneumonia due to Streptococcus pneumoniae
J14*	Pneumonia due to Haemophilus influenzae
J15.0	Pneumonia due to Klebsiella pneumoniae
J15.1	Pneumonia due to Pseudomonas
J15.2	Pneumonia due to staphylococcus
J15.3	Pneumonia due to streptococcus, group B

J15.4	Pneumonia due to other streptococci
J15.5	Pneumonia due to Escherichia coli
J20.1	Acute bronchitis due to Haemophilus influenzae
J20.2	Acute bronchitis due to streptococcus
K67.1	Gonococcal peritonitis
K67.2	Syphilitic peritonitis
L00*	Staphylococcal scalded skin syndrome
L01*	Impetigo
M00.0	Staphylococcal arthritis and polyarthritis
M00.1	Pneumococcal arthritis and polyarthritis
M00.2	Other streptococcal arthritis and polyarthritis
M01.0	Meningococcal arthritis
M01.2	Arthritis in Lyme disease
M73.0	Gonococcal bursitis
M73.1	Syphilitic bursitis
N74.2	Female syphilitic pelvic inflammatory disease
N74.3	Female gonococcal pelvic inflammatory disease
O98.1	Syphilis complicating pregnancy, childbirth and the puerperium
O98.2	Gonorrhoea complicating pregnancy, childbirth and the puerperium
P23.2	Congenital pneumonia due to staphylococcus
P23.3	Congenital pneumonia due to streptococcus, group B
P23.4	Congenital pneumonia due to Escherichia coli
P23.5	Congenital pneumonia due to Pseudomonas
P36.0	Sepsis of newborn due to streptococcus, group B
P36.1	Sepsis of newborn due to other and unspecified streptococci
P36.2	Sepsis of newborn due to Staphylococcus aureus
P36.3	Sepsis of newborn due to other and unspecified staphylococci
P36.4	Sepsis of newborn due to Escherichia coli

Gram-positive bacterial infections

A04.7	Enterocolitis due to Clostridium difficile
A05.0	Foodborne staphylococcal intoxication
A05.1	Botulism
A05.2	Foodborne Clostridium perfringens [Clostridium welchii] intoxication
A05.4	Foodborne Bacillus cereus intoxication
A22	Anthrax
A22.0	Cutaneous anthrax
A22.1	Pulmonary anthrax
A22.2	Gastrointestinal anthrax
A22.7	Anthrax sepsis
A22.8	Other forms of anthrax
A22.9	Anthrax, unspecified
A26	Erysipeloid
A26.0	Cutaneous erysipeloid
A26.7	Erysipelothrix sepsis

A26.8	Other forms of erysipeloid
A26.9	Erysipeloid, unspecified
A32	Listeriosis
A32.0	Cutaneous listeriosis
A32.1	Listerial meningitis and meningoencephalitis
A32.7	Listerial sepsis
A32.8	Other forms of listeriosis
A32.9	Listeriosis, unspecified
A33*	Tetanus neonatorum
A34*	Obstetrical tetanus
A35*	Other tetanus
A36*	Diphtheria
A38*	Scarlet fever
A40	Streptococcal sepsis
A40.0	Sepsis due to streptococcus, group A
A40.1	Sepsis due to streptococcus, group B
A40.2	Sepsis due to streptococcus, group D
A40.3	Sepsis due to Streptococcus pneumoniae
A40.8	Other streptococcal sepsis
A40.9	Streptococcal sepsis, unspecified
A41.0	Sepsis due to Staphylococcus aureus
A41.1	Sepsis due to other specified staphylococcus
A41.2	Sepsis due to unspecified staphylococcus
A42	Actinomycosis
A42.0	Pulmonary actinomycosis
A42.1	Abdominal actinomycosis
A42.2	Cervicofacial actinomycosis
A42.7	Actinomycotic sepsis
A42.8	Other forms of actinomycosis
A42.9	Actinomycosis, unspecified
A43	Nocardiosis
A43.0	Pulmonary nocardiosis
A43.1	Cutaneous nocardiosis
A43.8	Other forms of nocardiosis
A48.0	Gas gangrene
A48.3	Toxic shock syndrome
A49.0	Staphylococcal infection, unspecified site
A49.1	Streptococcal infection, unspecified site
B47.1	Actinomycetoma
B95*	Streptococcus and staphylococcus as the cause of diseases classified to other chapters
B96.7	Clostridium perfringens [C. perfringens] as the cause of diseases classified to other chapters
G00.1	Pneumococcal meningitis
G00.2	Streptococcal meningitis
G00.3	Staphylococcal meningitis
J02.0	Streptococcal pharyngitis
J03.0	Streptococcal tonsillitis
J13*	Pneumonia due to Streptococcus pneumoniae
J15.2	Pneumonia due to staphylococcus

J15.3	Pneumonia due to streptococcus, group B
J15.4	Pneumonia due to other streptococci
J20.2	Acute bronchitis due to streptococcus
L00*	Staphylococcal scalded skin syndrome
L01*	Impetigo
M00.0	Staphylococcal arthritis and polyarthritis
M00.1	Pneumococcal arthritis and polyarthritis
M00.2	Other streptococcal arthritis and polyarthritis
P23.2	Congenital pneumonia due to staphylococcus
P23.3	Congenital pneumonia due to streptococcus, group B
P36.0	Sepsis of newborn due to streptococcus, group B
P36.1	Sepsis of newborn due to other and unspecified streptococci
P36.2	Sepsis of newborn due to Staphylococcus aureus
P36.3	Sepsis of newborn due to other and unspecified staphylococci
P37.2	Neonatal (disseminated) listeriosis

Gram-negative bacterial infections

A00*	Cholera
A01*	Typhoid and paratyphoid fevers
A02	Other salmonella infections
A02.0	Salmonella enteritis
A02.1	Salmonella sepsis
A02.2	Localized salmonella infections
A02.8	Other specified salmonella infections
A02.9	Salmonella infection, unspecified
A03	Shigellosis
A03.0	Shigellosis due to Shigella dysenteriae
A03.1	Shigellosis due to Shigella flexneri
A03.2	Shigellosis due to Shigella boydii
A03.3	Shigellosis due to Shigella sonnei
A03.8	Other shigellosis
A03.9	Shigellosis, unspecified
A04.0	Enteropathogenic Escherichia coli infection
A04.1	Enterotoxigenic Escherichia coli infection
A04.2	Enteroinvasive Escherichia coli infection
A04.3	Enterohaemorrhagic Escherichia coli infection
A04.4	Other intestinal Escherichia coli infections
A04.5	Campylobacter enteritis
A04.6	Enteritis due to Yersinia enterocolitica
A05.3	Foodborne Vibrio parahaemolyticus intoxication
A20	Plague
A20.0	Bubonic plague
A20.1	Cellulocutaneous plague
A20.2	Pneumonic plague
A20.3	Plague meningitis

A20.7	Septicaemic plague
A20.8	Other forms of plague
A20.9	Plague, unspecified
A21	Tularaemia
A21.0	Ulceroglandular tularaemia
A21.1	Oculoglandular tularaemia
A21.2	Pulmonary tularaemia
A21.3	Gastrointestinal tularaemia
A21.7	Generalized tularaemia
A21.8	Other forms of tularaemia
A21.9	Tularaemia, unspecified
A23	Brucellosis
A23.0	Brucellosis due to Brucella melitensis
A23.1	Brucellosis due to Brucella abortus
A23.2	Brucellosis due to Brucella suis
A23.3	Brucellosis due to Brucella canis
A23.8	Other brucellosis
A23.9	Brucellosis, unspecified
A24	Glanders and melioidosis
A24.0	Glanders
A24.1	Acute and fulminating melioidosis
A24.2	Subacute and chronic melioidosis
A24.3	Other melioidosis
A25	Rat-bite fevers
A25.0	Spirillosis
A25.1	Streptobacillosis
A25.9	Rat-bite fever, unspecified
A27*	Leptospirosis
A28.0	Pasteurellosis
A28.1	Cat-scratch disease
A28.2	Extraintestinal yersiniosis
A37*	Whooping cough
A39	Meningococcal infection
A39.0	Meningococcal meningitis
A39.1	Waterhouse-Friderichsen syndrome
A39.2	Acute meningococcaemia
A39.3	Chronic meningococcaemia
A39.4	Meningococcaemia, unspecified
A39.5	Meningococcal heart disease
A39.8	Other meningococcal infections
A39.9	Meningococcal infection, unspecified
A41.3	Sepsis due to Haemophilus influenzae
A41.5	Sepsis due to other Gram-negative organisms
A44	Bartonellosis
A44.0	Systemic bartonellosis
A44.1	Cutaneous and mucocutaneous bartonellosis
A44.8	Other forms of bartonellosis
A44.9	Bartonellosis, unspecified

A48.1	Legionnaires disease
A48.2	Nonpneumonic Legionnaires disease [Pontiac fever]
A48.4	Brazilian purpuric fever
A49.2	Haemophilus influenzae infection, unspecified site
A50	Congenital syphilis
A50.0	Early congenital syphilis, symptomatic
A50.1	Early congenital syphilis, latent
A50.2	Early congenital syphilis, unspecified
A50.3	Late congenital syphilitic oculopathy
A50.4	Late congenital neurosyphilis [juvenile neurosyphilis]
A50.5	Other late congenital syphilis, symptomatic
A50.6	Late congenital syphilis, latent
A50.7	Late congenital syphilis, unspecified
A50.9	Congenital syphilis, unspecified
A51	Early syphilis
A51.0	Primary genital syphilis
A51.1	Primary anal syphilis
A51.2	Primary syphilis of other sites
A51.3	Secondary syphilis of skin and mucous membranes
A51.4	Other secondary syphilis
A51.5	Early syphilis, latent
A51.9	Early syphilis, unspecified
A52	Late syphilis
A52.0	Cardiovascular syphilis
A52.1	Symptomatic neurosyphilis
A52.2	Asymptomatic neurosyphilis
A52.3	Neurosyphilis, unspecified
A52.7	Other symptomatic late syphilis
A52.8	Late syphilis, latent
A52.9	Late syphilis, unspecified
A53*	Other and unspecified syphilis
A54	Gonococcal infection
A54.0	Gonococcal infection of lower genitourinary tract without periurethral or accessory gland abscess
A54.1	Gonococcal infection of lower genitourinary tract with periurethral and accessory gland abscess
A54.2	Gonococcal pelviperitonitis and other gonococcal genitourinary infections
A54.3	Gonococcal infection of eye
A54.4	Gonococcal infection of musculoskeletal system
A54.5	Gonococcal pharyngitis
A54.6	Gonococcal infection of anus and rectum
A54.8	Other gonococcal infections
A54.9	Gonococcal infection, unspecified
A55*	Chlamydial lymphogranuloma (venereum)
A56.0	Chlamydial infection of lower genitourinary tract
A56.1	Chlamydial infection of pelviperitoneum and other genitourinary organs
A56.2	Chlamydial infection of genitourinary tract, unspecified
A56.3	Chlamydial infection of anus and rectum
A56.4	Chlamydial infection of pharynx
A56.8	Sexually transmitted chlamydial infection of other sites

A57*	Chancroid
A58*	Granuloma inguinale
A65*	Nonvenereal syphilis
A66*	Yaws
A67*	Pinta [carate]
A68*	Relapsing fevers
A69.2	Lyme disease
A69.8	Other specified spirochaetal infections
A69.9	Spirochaetal infection, unspecified
A70*	Chlamydia psittaci infection
A71*	Trachoma
A74.0	Chlamydial conjunctivitis
A74.8	Other chlamydial diseases
A74.9	Chlamydial infection, unspecified
A75	Typhus fever
A75.0	Epidemic louse-borne typhus fever due to Rickettsia prowazekii
A75.1	Recrudescent typhus [Brill disease]
A75.2	Typhus fever due to Rickettsia typhi
A75.3	Typhus fever due to Rickettsia tsutsugamushi
A75.9	Typhus fever, unspecified
A77	Spotted fever [tick-borne rickettsioses]
A77.0	Spotted fever due to Rickettsia rickettsii
A77.1	Spotted fever due to Rickettsia conorii
A77.2	Spotted fever due to Rickettsia sibirica
A77.3	Spotted fever due to Rickettsia australis
A77.8	Other spotted fevers
A77.9	Spotted fever, unspecified
A78*	Q fever
A79	Other rickettsioses
A79.0	Trench fever
A79.1	Rickettsialpox due to Rickettsia akari
A79.8	Other specified rickettsioses
A79.9	Rickettsiosis, unspecified
B96.1	Klebsiella pneumoniae [K. pneumoniae] as the cause of diseases classified to other chapters
B96.2	Escherichia coli [E. coli] as the cause of diseases classified to other chapters
B96.3	Haemophilus influenzae [H. influenzae] as the cause of diseases classified to other chapters
B96.4	Proteus (mirabilis)(morganii) as the cause of diseases classified to other chapters
B96.5	Pseudomonas (aeruginosa) as the cause of diseases classified to other chapters
B96.6	Bacillus fragilis [B. fragilis] as the cause of diseases classified to other chapters
B98.0	Helicobacter pylori [H.pylori] as the cause of diseases classified to other chapters
B98.1	Vibrio vulnificus as the cause of diseases classified to other chapters
G00.0	Haemophilus meningitis
J14*	Pneumonia due to Haemophilus influenzae
J15.0	Pneumonia due to Klebsiella pneumoniae
J15.1	Pneumonia due to Pseudomonas
J15.5	Pneumonia due to Escherichia coli
J15.6	Pneumonia due to other aerobic Gram-negative bacteria
J16.0	Chlamydial pneumonia

J20.1	Acute bronchitis due to Haemophilus influenzae
K67.0	Chlamydial peritonitis
K67.1	Gonococcal peritonitis
K67.2	Syphilitic peritonitis
M01.0	Meningococcal arthritis
M01.2	Arthritis in Lyme disease
M49.1	Brucella spondylitis
M49.2	Enterobacterial spondylitis
M73.0	Gonococcal bursitis
M73.1	Syphilitic bursitis
N29.0	Late syphilis of kidney
N74.2	Female syphilitic pelvic inflammatory disease
N74.3	Female gonococcal pelvic inflammatory disease
N74.4	Female chlamydial pelvic inflammatory disease
O98.1	Syphilis complicating pregnancy, childbirth and the puerperium
O98.2	Gonorrhoea complicating pregnancy, childbirth and the puerperium
P23.1	Congenital pneumonia due to Chlamydia
P23.4	Congenital pneumonia due to Escherichia coli
P23.5	Congenital pneumonia due to Pseudomonas
P36.4	Sepsis of newborn due to Escherichia coli

Mycobacterial infections

A15*	Respiratory tuberculosis, bacteriologically and histologically confirmed
A16*	Respiratory tuberculosis, not confirmed bacteriologically or histologically
A17	Tuberculosis of nervous system
A17.0	Tuberculous meningitis
A17.1	Meningeal tuberculoma
A17.8	Other tuberculosis of nervous system
A17.9	Tuberculosis of nervous system, unspecified
A18*	Tuberculosis of other organs
A19*	Miliary tuberculosis
A30*	Leprosy [Hansen disease]
A31*	Infection due to other mycobacteria
K23.0	Tuberculous oesophagitis
K67.3	Tuberculous peritonitis
M01.1	Tuberculous arthritis
M49.0	Tuberculosis of spine
N74.0	Tuberculous infection of cervix uteri
N74.1	Female tuberculous pelvic inflammatory disease
O98.0	Tuberculosis complicating pregnancy, childbirth and the puerperium
P37.0	Congenital tuberculosis

Mycoplasma infections

A49.3	Mycoplasma infection, unspecified site
B96.0	Mycoplasma pneumoniae [M. pneumoniae] as the cause of diseases classified to other chapters
J15.7	Pneumonia due to Mycoplasma pneumoniae
J20.0	Acute bronchitis due to Mycoplasma pneumoniae

Central nervous system infections

A06.6	Amoebic brain abscess
A17.0	Tuberculous meningitis
A17.1	Meningeal tuberculoma
A20.3	Plague meningitis
A32.1	Listerial meningitis and meningoencephalitis
A39.0	Meningococcal meningitis
A50.4	Late congenital neurosyphilis [juvenile neurosyphilis]
A52.1	Symptomatic neurosyphilis
A52.2	Asymptomatic neurosyphilis
A52.3	Neurosyphilis, unspecified
A80*	Acute poliomyelitis
A81.1	Subacute sclerosing panencephalitis
A81.2	Progressive multifocal leukoencephalopathy
A83*	Mosquito-borne viral encephalitis
A84*	Tick-borne viral encephalitis
A85*	Other viral encephalitis, not elsewhere classified
A86*	Unspecified viral encephalitis
A87*	Viral meningitis
A88*	Other viral infections of central nervous system, not elsewhere classified
A89*	Unspecified viral infection of central nervous system
B00.3	Herpesviral meningitis
B00.4	Herpesviral encephalitis
B01.0	Varicella meningitis
B01.1	Varicella encephalitis
B02.0	Zoster encephalitis
B02.1	Zoster meningitis
B05.0	Measles complicated by encephalitis
B05.1	Measles complicated by meningitis
B06.0	Rubella with neurological complications
B15.0	Hepatitis A with hepatic coma
B16.0	Acute hepatitis B with delta-agent (coinfection) with hepatic coma
B16.2	Acute hepatitis B without delta-agent with hepatic coma
B19.0	Unspecified viral hepatitis with hepatic coma
B22.0	HIV disease resulting in encephalopathy
B26.1	Mumps meningitis

B26.2	Mumps encephalitis
B37.5	Candidal meningitis
B38.4	Coccidioidomycosis meningitis
B43.1	Phaeomycotic brain abscess
B45.1	Cerebral cryptococcosis
B46.1	Rhinocerebral mucormycosis
B50.0	Plasmodium falciparum malaria with cerebral complications
B58.2	Toxoplasma meningoencephalitis
B60.2	Naegleriasis
B69.0	Cysticercosis of central nervous system
G00	Bacterial meningitis, not elsewhere classified
G00.0	Haemophilus meningitis
G00.1	Pneumococcal meningitis
G00.2	Streptococcal meningitis
G00.3	Staphylococcal meningitis
G00.8	Other bacterial meningitis
G00.9	Bacterial meningitis, unspecified
G01*	Meningitis in bacterial diseases classified elsewhere
G02.0	Meningitis in viral diseases classified elsewhere
G02.1	Meningitis in mycoses
G02.8	Meningitis in other specified infectious and parasitic diseases classified elsewhere
G03*	Meningitis due to other and unspecified causes
G04.1	Tropical spastic paraplegia
G04.2	Bacterial meningoencephalitis and meningomyelitis, not elsewhere classified
G05.0	Encephalitis, myelitis and encephalomyelitis in bacterial diseases classified elsewhere
G05.1	Encephalitis, myelitis and encephalomyelitis in viral diseases classified elsewhere
G05.2	Encephalitis, myelitis and encephalomyelitis in other infectious and parasitic diseases classified elsewhere
G06*	Intracranial and intraspinal abscess and granuloma
G07*	Intracranial and intraspinal abscess and granuloma in diseases classified elsewhere
I68.1	Cerebral arteritis in infectious and parasitic diseases classified elsewhere
P35.0	Congenital rubella syndrome
P35.1	Congenital cytomegalovirus infection

Extra-central nervous system infections

A00*	Cholera
A01*	Typhoid and paratyphoid fevers
A02	Other salmonella infections
A02.0	Salmonella enteritis
A02.1	Salmonella sepsis
A02.2	Localized salmonella infections
A02.8	Other specified salmonella infections
A02.9	Salmonella infection, unspecified
A03	Shigellosis
A03.0	Shigellosis due to Shigella dysenteriae
A03.1	Shigellosis due to Shigella flexneri

A03.2	Shigellosis due to Shigella boydii
A03.3	Shigellosis due to Shigella sonnei
A03.8	Other shigellosis
A03.9	Shigellosis, unspecified
A04	Other bacterial intestinal infections
A04.0	Enteropathogenic Escherichia coli infection
A04.1	Enterotoxigenic Escherichia coli infection
A04.2	Enteroinvasive Escherichia coli infection
A04.3	Enterohaemorrhagic Escherichia coli infection
A04.4	Other intestinal Escherichia coli infections
A04.5	Campylobacter enteritis
A04.6	Enteritis due to Yersinia enterocolitica
A04.7	Enterocolitis due to Clostridium difficile
A04.8	Other specified bacterial intestinal infections
A04.9	Bacterial intestinal infection, unspecified
A05	Other bacterial foodborne intoxications, not elsewhere classified
A05.0	Foodborne staphylococcal intoxication
A05.1	Botulism
A05.2	Foodborne Clostridium perfringens [Clostridium welchii] intoxication
A05.3	Foodborne Vibrio parahaemolyticus intoxication
A05.4	Foodborne Bacillus cereus intoxication
A05.8	Other specified bacterial foodborne intoxications
A05.9	Bacterial foodborne intoxication, unspecified
A06	Amoebiasis
A06.0	Acute amoebic dysentery
A06.1	Chronic intestinal amoebiasis
A06.2	Amoebic nondysenteric colitis
A06.3	Amoeboma of intestine
A06.4	Amoebic liver abscess
A06.5	Amoebic lung abscess
A06.7	Cutaneous amoebiasis
A06.8	Amoebic infection of other sites
A06.9	Amoebiasis, unspecified
A07*	Other protozoal intestinal diseases
A08.0	Rotaviral enteritis
A08.1	Acute gastroenteropathy due to Norwalk agent
A08.2	Adenoviral enteritis
A08.3	Other viral enteritis
A08.4	Viral intestinal infection, unspecified
A08.5	Other specified intestinal infections
A09*	Other gastroenteritis and colitis of infectious and unspecified origin
A15*	Respiratory tuberculosis, bacteriologically and histologically confirmed
A16*	Respiratory tuberculosis, not confirmed bacteriologically or histologically
A17	Tuberculosis of nervous system
A17.8	Other tuberculosis of nervous system
A17.9	Tuberculosis of nervous system, unspecified
A18*	Tuberculosis of other organs
A19*	Miliary tuberculosis

A20	Plague
A20.0	Bubonic plague
A20.1	Cellulocutaneous plague
A20.2	Pneumonic plague
A20.7	Septicaemic plague
A20.8	Other forms of plague
A20.9	Plague, unspecified
A21	Tularaemia
A21.0	Ulceroglandular tularaemia
A21.1	Oculoglandular tularaemia
A21.2	Pulmonary tularaemia
A21.3	Gastrointestinal tularaemia
A21.7	Generalized tularaemia
A21.8	Other forms of tularaemia
A21.9	Tularaemia, unspecified
A22	Anthrax
A22.0	Cutaneous anthrax
A22.1	Pulmonary anthrax
A22.2	Gastrointestinal anthrax
A22.7	Anthrax sepsis
A22.8	Other forms of anthrax
A22.9	Anthrax, unspecified
A23	Brucellosis
A23.0	Brucellosis due to Brucella melitensis
A23.1	Brucellosis due to Brucella abortus
A23.2	Brucellosis due to Brucella suis
A23.3	Brucellosis due to Brucella canis
A23.8	Other brucellosis
A23.9	Brucellosis, unspecified
A24	Glanders and melioidosis
A24.0	Glanders
A24.1	Acute and fulminating melioidosis
A24.2	Subacute and chronic melioidosis
A24.3	Other melioidosis
A25	Rat-bite fevers
A25.0	Spirillosis
A25.1	Streptobacillosis
A25.9	Rat-bite fever, unspecified
A26	Erysipeloid
A26.0	Cutaneous erysipeloid
A26.7	Erysipelothrix sepsis
A26.8	Other forms of erysipeloid
A26.9	Erysipeloid, unspecified
A27*	Leptospirosis
A28	Other zoonotic bacterial diseases, not elsewhere classified
A28.0	Pasteurellosis
A28.1	Cat-scratch disease
A28.2	Extraintestinal yersiniosis

A28.8	Other specified zoonotic bacterial diseases, not elsewhere classified
A28.9	Zoonotic bacterial disease, unspecified
A30*	Leprosy [Hansen disease]
A31*	Infection due to other mycobacteria
A32	Listeriosis
A32.0	Cutaneous listeriosis
A32.7	Listerial sepsis
A32.8	Other forms of listeriosis
A32.9	Listeriosis, unspecified
A33*	Tetanus neonatorum
A34*	Obstetrical tetanus
A35*	Other tetanus
A36*	Diphtheria
A37*	Whooping cough
A38*	Scarlet fever
A39	Meningococcal infection
A39.1	Waterhouse-Friderichsen syndrome
A39.2	Acute meningococcaemia
A39.3	Chronic meningococcaemia
A39.4	Meningococcaemia, unspecified
A39.5	Meningococcal heart disease
A39.8	Other meningococcal infections
A39.9	Meningococcal infection, unspecified
A40	Streptococcal sepsis
A40.0	Sepsis due to streptococcus, group A
A40.1	Sepsis due to streptococcus, group B
A40.2	Sepsis due to streptococcus, group D
A40.3	Sepsis due to Streptococcus pneumoniae
A40.8	Other streptococcal sepsis
A40.9	Streptococcal sepsis, unspecified
A41	Other sepsis
A41.0	Sepsis due to Staphylococcus aureus
A41.1	Sepsis due to other specified staphylococcus
A41.2	Sepsis due to unspecified staphylococcus
A41.3	Sepsis due to Haemophilus influenzae
A41.4	Sepsis due to anaerobes
A41.5	Sepsis due to other Gram-negative organisms
A41.8	Other specified sepsis
A41.9	Sepsis, unspecified
A42	Actinomycosis
A42.0	Pulmonary actinomycosis
A42.1	Abdominal actinomycosis
A42.2	Cervicofacial actinomycosis
A42.7	Actinomycotic sepsis
A42.8	Other forms of actinomycosis
A42.9	Actinomycosis, unspecified
A43	Nocardiosis
A43.0	Pulmonary nocardiosis

A43.1	Cutaneous nocardiosis
A43.8	Other forms of nocardiosis
A44	Bartonellosis
A44.0	Systemic bartonellosis
A44.1	Cutaneous and mucocutaneous bartonellosis
A44.8	Other forms of bartonellosis
A44.9	Bartonellosis, unspecified
A46*	Erysipelas
A48.0	Gas gangrene
A48.1	Legionnaires disease
A48.2	Nonpneumonic Legionnaires disease [Pontiac fever]
A48.3	Toxic shock syndrome
A48.4	Brazilian purpuric fever
A48.8	Other specified bacterial diseases
A49	Bacterial infection of unspecified site
A49.0	Staphylococcal infection, unspecified site
A49.1	Streptococcal infection, unspecified site
A49.2	Haemophilus influenzae infection, unspecified site
A49.3	Mycoplasma infection, unspecified site
A49.8	Other bacterial infections of unspecified site
A49.9	Bacterial infection, unspecified
A50	Congenital syphilis
A50.0	Early congenital syphilis, symptomatic
A50.1	Early congenital syphilis, latent
A50.2	Early congenital syphilis, unspecified
A50.3	Late congenital syphilitic oculopathy
A50.5	Other late congenital syphilis, symptomatic
A50.6	Late congenital syphilis, latent
A50.7	Late congenital syphilis, unspecified
A50.9	Congenital syphilis, unspecified
A51	Early syphilis
A51.0	Primary genital syphilis
A51.1	Primary anal syphilis
A51.2	Primary syphilis of other sites
A51.3	Secondary syphilis of skin and mucous membranes
A51.4	Other secondary syphilis
A51.5	Early syphilis, latent
A51.9	Early syphilis, unspecified
A52	Late syphilis
A52.0	Cardiovascular syphilis
A52.7	Other symptomatic late syphilis
A52.8	Late syphilis, latent
A52.9	Late syphilis, unspecified
A53*	Other and unspecified syphilis
A54	Gonococcal infection
A54.0	Gonococcal infection of lower genitourinary tract without periurethral or accessory gland abscess
A54.1	Gonococcal infection of lower genitourinary tract with periurethral and accessory gland abscess
A54.2	Gonococcal pelviperitonitis and other gonococcal genitourinary infections

A54.3	Gonococcal infection of eye
A54.4	Gonococcal infection of musculoskeletal system
A54.5	Gonococcal pharyngitis
A54.6	Gonococcal infection of anus and rectum
A54.8	Other gonococcal infections
A54.9	Gonococcal infection, unspecified
A55*	Chlamydial lymphogranuloma (venereum)
A56.0	Chlamydial infection of lower genitourinary tract
A56.1	Chlamydial infection of pelviperitoneum and other genitourinary organs
A56.2	Chlamydial infection of genitourinary tract, unspecified
A56.3	Chlamydial infection of anus and rectum
A56.4	Chlamydial infection of pharynx
A56.8	Sexually transmitted chlamydial infection of other sites
A57*	Chancroid
A58*	Granuloma inguinale
A59*	Trichomoniasis
A60*	Anogenital herpesviral [herpes simplex] infection
A63.0	Anogenital (venereal) warts
A63.8	Other specified predominantly sexually transmitted diseases
A64*	Unspecified sexually transmitted disease
A65*	Nonvenereal syphilis
A66*	Yaws
A67*	Pinta [carate]
A68*	Relapsing fevers
A69	Other spirochaetal infections
A69.0	Necrotizing ulcerative stomatitis
A69.1	Other Vincent infections
A69.2	Lyme disease
A69.8	Other specified spirochaetal infections
A69.9	Spirochaetal infection, unspecified
A70*	Chlamydia psittaci infection
A71*	Trachoma
A74.0	Chlamydial conjunctivitis
A74.8	Other chlamydial diseases
A74.9	Chlamydial infection, unspecified
A75	Typhus fever
A75.0	Epidemic louse-borne typhus fever due to Rickettsia prowazekii
A75.1	Recrudescent typhus [Brill disease]
A75.2	Typhus fever due to Rickettsia typhi
A75.3	Typhus fever due to Rickettsia tsutsugamushi
A75.9	Typhus fever, unspecified
A77	Spotted fever [tick-borne rickettsioses]
A77.0	Spotted fever due to Rickettsia rickettsii
A77.1	Spotted fever due to Rickettsia conorii
A77.2	Spotted fever due to Rickettsia sibirica
A77.3	Spotted fever due to Rickettsia australis
A77.8	Other spotted fevers
A77.9	Spotted fever, unspecified

A78*	Q fever
A79	Other rickettsioses
A79.0	Trench fever
A79.1	Rickettsialpox due to Rickettsia akari
A79.8	Other specified rickettsioses
A79.9	Rickettsiosis, unspecified
A90*	Dengue fever [classical dengue]
A91*	Dengue haemorrhagic fever
A92	Other mosquito-borne viral fevers
A92.0	Chikungunya virus disease
A92.1	O'nyong-nyong fever
A92.2	Venezuelan equine fever
A92.3	West Nile virus infection
A92.4	Rift Valley fever
A92.8	Other specified mosquito-borne viral fevers
A92.9	Mosquito-borne viral fever, unspecified
A93	Other arthropod-borne viral fevers, not elsewhere classified
A93.0	Oropouche virus disease
A93.1	Sandfly fever
A93.2	Colorado tick fever
A93.8	Other specified arthropod-borne viral fevers
A94*	Unspecified arthropod-borne viral fever
A95*	Yellow fever
A96*	Arenaviral haemorrhagic fever
A97*	Dengue
A98	Other viral haemorrhagic fevers, not elsewhere classified
A98.0	Crimean-Congo haemorrhagic fever
A98.1	Omsk haemorrhagic fever
A98.2	Kyasanur Forest disease
A98.3	Marburg virus disease
A98.4	Ebola virus disease
A98.5	Haemorrhagic fever with renal syndrome
A98.8	Other specified viral haemorrhagic fevers
A99*	Unspecified viral haemorrhagic fever
B00	Herpesviral [herpes simplex] infections
B00.0	Eczema herpeticum
B00.1	Herpesviral vesicular dermatitis
B00.2	Herpesviral gingivostomatitis and pharyngotonsillitis
B00.5	Herpesviral ocular disease
B00.7	Disseminated herpesviral disease
B00.8	Other forms of herpesviral infection
B00.9	Herpesviral infection, unspecified
B01	Varicella [chickenpox]
B01.2	Varicella pneumonia
B01.8	Varicella with other complications
B01.9	Varicella without complication
B02	Zoster [herpes zoster]
B02.2	Zoster with other nervous system involvement

B02.3	Zoster ocular disease
B02.7	Disseminated zoster
B02.8	Zoster with other complications
B02.9	Zoster without complication
B04*	Monkeypox
B05	Measles
B05.2	Measles complicated by pneumonia
B05.3	Measles complicated by otitis media
B05.4	Measles with intestinal complications
B05.8	Measles with other complications
B05.9	Measles without complication
B06	Rubella [German measles]
B06.8	Rubella with other complications
B06.9	Rubella without complication
B07*	Viral warts
B08.0	Other orthopoxvirus infections
B08.1	Molluscum contagiosum
B08.2	Exanthema subitum [sixth disease]
B08.3	Erythema infectiosum [fifth disease]
B08.4	Enteroviral vesicular stomatitis with exanthem
B08.5	Enteroviral vesicular pharyngitis
B08.8	Other specified viral infections characterized by skin and mucous membrane lesions
B09*	Unspecified viral infection characterized by skin and mucous membrane lesions
B15	Acute hepatitis A
B15.9	Hepatitis A without hepatic coma
B16	Acute hepatitis B
B16.1	Acute hepatitis B with delta-agent (coinfection) without hepatic coma
B16.9	Acute hepatitis B without delta-agent and without hepatic coma
B17*	Other acute viral hepatitis
B18*	Chronic viral hepatitis
B19	Unspecified viral hepatitis
B19.9	Unspecified viral hepatitis without hepatic coma
B20*	Human immunodeficiency virus [HIV] disease resulting in infectious and parasitic diseases
B21*	Human immunodeficiency virus [HIV] disease resulting in malignant neoplasms
B21.0	HIV disease resulting in Kaposi sarcoma
B22	Human immunodeficiency virus [HIV] disease resulting in other specified diseases
B22.1	HIV disease resulting in lymphoid interstitial pneumonitis
B22.2	HIV disease resulting in wasting syndrome
B22.7	HIV disease resulting in multiple diseases classified elsewhere
B23*	Human immunodeficiency virus [HIV] disease resulting in other conditions
B24*	Unspecified human immunodeficiency virus [HIV] disease
B25*	Cytomegaloviral disease
B26	Mumps
B26.0	Mumps orchitis
B26.3	Mumps pancreatitis
B26.8	Mumps with other complications
B26.9	Mumps without complication
B27	Infectious mononucleosis

B27.0	Gammaherpesviral mononucleosis
B27.1	Cytomegaloviral mononucleosis
B27.8	Other infectious mononucleosis
B27.9	Infectious mononucleosis, unspecified
B30*	Viral conjunctivitis
B33	Other viral diseases, not elsewhere classified
B33.0	Epidemic myalgia
B33.1	Ross River disease
B33.2	Viral carditis
B33.3	Retrovirus infections, not elsewhere classified
B33.4	Hantavirus (cardio-)pulmonary syndrome [HPS] [HCPS]
B33.8	Other specified viral diseases
B34	Viral infection of unspecified site
B34.0	Adenovirus infection, unspecified site
B34.1	Enterovirus infection, unspecified site
B34.2	Coronavirus infection, unspecified site
B34.3	Parvovirus infection, unspecified site
B34.4	Papovavirus infection, unspecified site
B34.8	Other viral infections of unspecified site
B34.9	Viral infection, unspecified
B35*	Dermatophytosis
B36*	Other superficial mycoses
B37	Candidiasis
B37.0	Candidal stomatitis
B37.1	Pulmonary candidiasis
B37.2	Candidiasis of skin and nail
B37.3	Candidiasis of vulva and vagina
B37.4	Candidiasis of other urogenital sites
B37.6	Candidal endocarditis
B37.7	Candidal sepsis
B37.8	Candidiasis of other sites
B37.9	Candidiasis, unspecified
B38	Coccidioidomycosis
B38.0	Acute pulmonary coccidioidomycosis
B38.1	Chronic pulmonary coccidioidomycosis
B38.2	Pulmonary coccidioidomycosis, unspecified
B38.3	Cutaneous coccidioidomycosis
B38.7	Disseminated coccidioidomycosis
B38.8	Other forms of coccidioidomycosis
B38.9	Coccidioidomycosis, unspecified
B39	Histoplasmosis
B39.0	Acute pulmonary histoplasmosis capsulati
B39.1	Chronic pulmonary histoplasmosis capsulati
B39.2	Pulmonary histoplasmosis capsulati, unspecified
B39.3	Disseminated histoplasmosis capsulati
B39.4	Histoplasmosis capsulati, unspecified
B39.5	Histoplasmosis duboisii
B39.9	Histoplasmosis, unspecified

B40	Blastomycosis
B40.0	Acute pulmonary blastomycosis
B40.1	Chronic pulmonary blastomycosis
B40.2	Pulmonary blastomycosis, unspecified
B40.3	Cutaneous blastomycosis
B40.7	Disseminated blastomycosis
B40.8	Other forms of blastomycosis
B40.9	Blastomycosis, unspecified
B41	Paracoccidioidomycosis
B41.0	Pulmonary paracoccidioidomycosis
B41.7	Disseminated paracoccidioidomycosis
B41.8	Other forms of paracoccidioidomycosis
B41.9	Paracoccidioidomycosis, unspecified
B42	Sporotrichosis
B42.0	Pulmonary sporotrichosis
B42.1	Lymphocutaneous sporotrichosis
B42.7	Disseminated sporotrichosis
B42.8	Other forms of sporotrichosis
B42.9	Sporotrichosis, unspecified
B43	Chromomycosis and phaeomycotic abscess
B43.0	Cutaneous chromomycosis
B43.2	Subcutaneous phaeomycotic abscess and cyst
B43.8	Other forms of chromomycosis
B43.9	Chromomycosis, unspecified
B44*	Aspergillosis
B45	Cryptococcosis
B45.0	Pulmonary cryptococcosis
B45.2	Cutaneous cryptococcosis
B45.3	Osseous cryptococcosis
B45.7	Disseminated cryptococcosis
B45.8	Other forms of cryptococcosis
B45.9	Cryptococcosis, unspecified
B46	Zygomycosis
B46.0	Pulmonary mucormycosis
B46.2	Gastrointestinal mucormycosis
B46.3	Cutaneous mucormycosis
B46.4	Disseminated mucormycosis
B46.5	Mucormycosis, unspecified
B46.8	Other zygomycoses
B46.9	Zygomycosis, unspecified
B47	Mycetoma
B47.0	Eumycetoma
B47.1	Actinomycetoma
B47.9	Mycetoma, unspecified
B48	Other mycoses, not elsewhere classified
B48.0	Lobomycosis
B48.1	Rhinosporidiosis
B48.2	Allescheriasis

B48.3	Geotrichosis
B48.4	Penicillosis
B48.7	Opportunistic mycoses
B48.8	Other specified mycoses
B49*	Unspecified mycosis
B50*	Plasmodium falciparum malaria
B50.8	Other severe and complicated Plasmodium falciparum malaria
B50.9	Plasmodium falciparum malaria, unspecified
B51*	Plasmodium vivax malaria
B52*	Plasmodium malariae malaria
B53	Other parasitologically confirmed malaria
B53.0	Plasmodium ovale malaria
B53.1	Malaria due to simian plasmodia
B53.8	Other parasitologically confirmed malaria, not elsewhere classified
B54*	Unspecified malaria
B55.0	Visceral leishmaniasis
B55.1	Cutaneous leishmaniasis
B55.2	Mucocutaneous leishmaniasis
B55.9	Leishmaniasis, unspecified
B56*	African trypanosomiasis
B57*	Chagas disease
B58	Toxoplasmosis
B58.0	Toxoplasma oculopathy
B58.1	Toxoplasma hepatitis
B58.3	Pulmonary toxoplasmosis
B58.8	Toxoplasmosis with other organ involvement
B58.9	Toxoplasmosis, unspecified
B59*	Pneumocystosis
B60.0	Babesiosis
B60.1	Acanthamoebiasis
B60.8	Other specified protozoal diseases
B64*	Unspecified protozoal disease
B65	Schistosomiasis [bilharziasis]
B65.0	Schistosomiasis due to Schistosoma haematobium [urinary schistosomiasis]
B65.1	Schistosomiasis due to Schistosoma mansoni [intestinal schistosomiasis]
B65.2	Schistosomiasis due to Schistosoma japonicum
B65.3	Cercarial dermatitis
B65.8	Other schistosomiases
B65.9	Schistosomiasis, unspecified
B66	Other fluke infections
B66.0	Opisthorchiasis
B66.1	Clonorchiasis
B66.2	Dicrocoeliasis
B66.3	Fascioliasis
B66.4	Paragonimiasis
B66.5	Fasciolopsiasis
B66.8	Other specified fluke infections
B66.9	Fluke infection, unspecified

B67*	Echinococcosis
B68	Taeniasis
B68.0	Taenia solium taeniasis
B68.1	Taenia saginata taeniasis
B68.9	Taeniasis, unspecified
B69	Cysticercosis
B69.1	Cysticercosis of eye
B69.8	Cysticercosis of other sites
B69.9	Cysticercosis, unspecified
B70	Diphyllobothriasis and sparganosis
B70.0	Diphyllobothriasis
B70.1	Sparganosis
B71	Other cestode infections
B71.0	Hymenolepiasis
B71.1	Dipylidiasis
B71.8	Other specified cestode infections
B71.9	Cestode infection, unspecified
B72*	Dracunculiasis
B73*	Onchocerciasis
B74*	Filariasis
B75*	Trichinellosis
B76*	Hookworm diseases
B77*	Ascariasis
B78*	Strongyloidiasis
B79*	Trichuriasis
B80*	Enterobiasis
B81	Other intestinal helminthiases, not elsewhere classified
B81.0	Anisakiasis
B81.1	Intestinal capillariasis
B81.2	Trichostrongyliasis
B81.3	Intestinal angiostrongyliasis
B81.4	Mixed intestinal helminthiases
B81.8	Other specified intestinal helminthiases
B82	Unspecified intestinal parasitism
B82.0	Intestinal helminthiasis, unspecified
B82.9	Intestinal parasitism, unspecified
B83	Other helminthiases
B83.0	Visceral larva migrans
B83.1	Gnathostomiasis
B83.2	Angiostrongyliasis due to Parastrongylus cantonensis
B83.3	Syngamiasis
B83.4	Internal hirudiniasis
B83.8	Other specified helminthiases
B83.9	Helminthiasis, unspecified
B85*	Pediculosis and phthiriasis
B86*	Scabies
B87*	Myiasis
B88	Other infestations

B88.0	Other acariasis
B88.1	Tungiasis [sandflea infestation]
B88.2	Other arthropod infestations
B88.3	External hirudiniasis
B88.8	Other specified infestations
B88.9	Infestation, unspecified
B89*	Unspecified parasitic disease
B95*	Streptococcus and staphylococcus as the cause of diseases classified to other chapters
B96.0	Mycoplasma pneumoniae [M. pneumoniae] as the cause of diseases classified to other chapters
B96.1	Klebsiella pneumoniae [K. pneumoniae] as the cause of diseases classified to other chapters
B96.2	Escherichia coli [E. coli] as the cause of diseases classified to other chapters
B96.3	Haemophilus influenzae [H. influenzae] as the cause of diseases classified to other chapters
B96.4	Proteus (mirabilis)(morganii) as the cause of diseases classified to other chapters
B96.5	Pseudomonas (aeruginosa) as the cause of diseases classified to other chapters
B96.6	Bacillus fragilis [B. fragilis] as the cause of diseases classified to other chapters
B96.7	Clostridium perfringens [C. perfringens] as the cause of diseases classified to other chapters
B96.8	Other specified bacterial agents as the cause of diseases classified to other chapters
B97	Viral agents as the cause of diseases classified to other chapters
B97.0	Adenovirus as the cause of diseases classified to other chapters
B97.1	Enterovirus as the cause of diseases classified to other chapters
B97.2	Coronavirus as the cause of diseases classified to other chapters
B97.3	Retrovirus as the cause of diseases classified to other chapters
B97.4	Respiratory syncytial virus as the cause of diseases classified to other chapters
B97.5	Reovirus as the cause of diseases classified to other chapters
B97.6	Parvovirus as the cause of diseases classified to other chapters
B97.7	Papillomavirus as the cause of diseases classified to other chapters
B97.8	Other viral agents as the cause of diseases classified to other chapters
B98.0	Helicobacter pylori [H.pylori] as the cause of diseases classified to other chapters
B98.1	Vibrio vulnificus as the cause of diseases classified to other chapters
B99*	Other and unspecified infectious diseases
C46	Kaposi sarcoma
D73.3	Abscess of spleen
E32.1	Abscess of thymus
H00*	Hordeolum and chalazion
H01.0	Blepharitis
H05.0	Acute inflammation of orbit
H06.1	Parasitic infestation of orbit in diseases classified elsewhere
H10.0	Mucopurulent conjunctivitis
H10.5	Blepharoconjunctivitis
H13.0	Filarial infection of conjunctiva
H19.0	Scleritis and episcleritis in diseases classified elsewhere
H19.1	Herpesviral keratitis and keratoconjunctivitis
H19.2	Keratitis and keratoconjunctivitis in other infectious and parasitic diseases classified elsewhere
H22.0	Iridocyclitis in infectious and parasitic diseases classified elsewhere
H32.0	Chorioretinal inflammation in infectious and parasitic diseases classified elsewhere
H44.0	Purulent endophthalmitis
H60.0	Abscess of external ear
H60.1	Cellulitis of external ear

H60.2	Malignant otitis externa
H60.3	Other infective otitis externa
H62.0	Otitis externa in bacterial diseases classified elsewhere
H62.1	Otitis externa in viral diseases classified elsewhere
H62.2	Otitis externa in mycoses
H62.3	Otitis externa in other infectious and parasitic diseases classified elsewhere
H66.0	Acute suppurative otitis media
H67.0	Otitis media in bacterial diseases classified elsewhere
H67.1	Otitis media in viral diseases classified elsewhere
H70.0	Acute mastoiditis
H75.0	Mastoiditis in infectious and parasitic diseases classified elsewhere
I30.1	Infective pericarditis
I32.0	Pericarditis in bacterial diseases classified elsewhere
I32.1	Pericarditis in other infectious and parasitic diseases classified elsewhere
I33.0	Acute and subacute infective endocarditis
I40.0	Infective myocarditis
I41.0	Myocarditis in bacterial diseases classified elsewhere
I41.1	Myocarditis in viral diseases classified elsewhere
I41.2	Myocarditis in other infectious and parasitic diseases classified elsewhere
I43.0	Cardiomyopathy in infectious and parasitic diseases classified elsewhere
I52.0	Other heart disorders in bacterial diseases classified elsewhere
I52.1	Other heart disorders in other infectious and parasitic diseases classified elsewhere
J01.0	Acute maxillary sinusitis
J02	Acute pharyngitis
J02.0	Streptococcal pharyngitis
J02.8	Acute pharyngitis due to other specified organisms
J02.9	Acute pharyngitis, unspecified
J03	Acute tonsillitis
J03.0	Streptococcal tonsillitis
J03.8	Acute tonsillitis due to other specified organisms
J03.9	Acute tonsillitis, unspecified
J04*	Acute laryngitis and tracheitis
J05.1	Acute epiglottitis
J09*	Influenza due to certain identified influenza virus
J10*	Influenza due to other identified influenza virus
J11*	Influenza, virus not identified
J12*	Viral pneumonia, not elsewhere classified
J13*	Pneumonia due to Streptococcus pneumoniae
J14*	Pneumonia due to Haemophilus influenzae
J15	Bacterial pneumonia, not elsewhere classified
J15.0	Pneumonia due to Klebsiella pneumoniae
J15.1	Pneumonia due to Pseudomonas
J15.2	Pneumonia due to staphylococcus
J15.3	Pneumonia due to streptococcus, group B
J15.4	Pneumonia due to other streptococci
J15.5	Pneumonia due to Escherichia coli
J15.6	Pneumonia due to other aerobic Gram-negative bacteria
J15.7	Pneumonia due to Mycoplasma pneumoniae

J15.8	Other bacterial pneumonia
J15.9	Bacterial pneumonia, unspecified
J16	Pneumonia due to other infectious organisms, not elsewhere classified
J16.0	Chlamydial pneumonia
J16.8	Pneumonia due to other specified infectious organisms
J17.0	Pneumonia in bacterial diseases classified elsewhere
J17.1	Pneumonia in viral diseases classified elsewhere
J17.2	Pneumonia in mycoses
J17.3	Pneumonia in parasitic diseases
J17.8	Pneumonia in other diseases classified elsewhere
J18*	Pneumonia, organism unspecified
J20	Acute bronchitis
J20.0	Acute bronchitis due to Mycoplasma pneumoniae
J20.1	Acute bronchitis due to Haemophilus influenzae
J20.2	Acute bronchitis due to streptococcus
J20.3	Acute bronchitis due to coxsackievirus
J20.4	Acute bronchitis due to parainfluenza virus
J20.5	Acute bronchitis due to respiratory syncytial virus
J20.6	Acute bronchitis due to rhinovirus
J20.7	Acute bronchitis due to echovirus
J20.8	Acute bronchitis due to other specified organisms
J20.9	Acute bronchitis, unspecified
J21*	Acute bronchiolitis
J21.0	Acute bronchiolitis due to respiratory syncytial virus
J21.1	Acute bronchiolitis due to human metapneumovirus
J21.8	Acute bronchiolitis due to other specified organisms
J21.9	Acute bronchiolitis, unspecified
J22*	Unspecified acute lower respiratory infection
J36*	Peritonsillar abscess
J39.0	Retropharyngeal and parapharyngeal abscess
J39.1	Other abscess of pharynx
J85.1	Abscess of lung with pneumonia
J85.2	Abscess of lung without pneumonia
J85.3	Abscess of mediastinum
J86*	Pyothorax
K02*	Dental caries
K04.4	Acute apical periodontitis of pulpal origin
K04.5	Chronic apical periodontitis
K04.6	Periapical abscess with sinus
K04.7	Periapical abscess without sinus
K05.0	Acute gingivitis
K05.2	Acute periodontitis
K05.3	Chronic periodontitis
K05.4	Periodontosis
K11.3	Abscess of salivary gland
K12.2	Cellulitis and abscess of mouth
K23.0	Tuberculous oesophagitis
K23.1	Megaoesophagus in Chagas disease

K35*	Acute appendicitis
K57.0	Diverticular disease of small intestine with perforation and abscess
K57.2	Diverticular disease of large intestine with perforation and abscess
K57.4	Diverticular disease of both small and large intestine with perforation and abscess
K57.8	Diverticular disease of intestine, part unspecified, with perforation and abscess
K61*	Abscess of anal and rectal regions
K63.0	Abscess of intestine
K65.0	Acute peritonitis
K67.0	Chlamydial peritonitis
K67.1	Gonococcal peritonitis
K67.2	Syphilitic peritonitis
K67.3	Tuberculous peritonitis
K67.8	Other disorders of peritoneum in infectious diseases classified elsewhere
K75.0	Abscess of liver
K77.0	Liver disorders in infectious and parasitic diseases classified elsewhere
L00*	Staphylococcal scalded skin syndrome
L01*	Impetigo
L02*	Cutaneous abscess, furuncle and carbuncle
L03*	Cellulitis
L04*	Acute lymphadenitis
L05*	Pilonidal cyst
L08*	Other local infections of skin and subcutaneous tissue
L70.1	Acne conglobata
M00	Pyogenic arthritis
M00.0	Staphylococcal arthritis and polyarthritis
M00.1	Pneumococcal arthritis and polyarthritis
M00.2	Other streptococcal arthritis and polyarthritis
M00.8	Arthritis and polyarthritis due to other specified bacterial agents
M00.9	Pyogenic arthritis, unspecified
M01.0	Meningococcal arthritis
M01.1	Tuberculous arthritis
M01.2	Arthritis in Lyme disease
M01.3	Arthritis in other bacterial diseases classified elsewhere
M01.4	Rubella arthritis
M01.5	Arthritis in other viral diseases classified elsewhere
M01.6	Arthritis in mycoses
M01.8	Arthritis in other infectious and parasitic diseases classified elsewhere
M46.2	Osteomyelitis of vertebra
M46.3	Infection of intervertebral disc (pyogenic)
M46.5	Other infective spondylopathies
M49.0	Tuberculosis of spine
M49.1	Brucella spondylitis
M49.2	Enterobacterial spondylitis
M49.3	Spondylopathy in other infectious and parasitic diseases classified elsewhere
M60.0	Infective myositis
M63.0	Myositis in bacterial diseases classified elsewhere
M63.1	Myositis in protozoal and parasitic infections classified elsewhere
M63.2	Myositis in other infectious diseases classified elsewhere

M65.0	Abscess of tendon sheath
M65.1	Other infective (teno)synovitis
M71.0	Abscess of bursa
M71.1	Other infective bursitis
M72.6	Necrotizing fasciitis
M73.0	Gonococcal bursitis
M73.1	Syphilitic bursitis
M86*	Osteomyelitis
M86.0	Acute haematogenous osteomyelitis
M86.1	Other acute osteomyelitis
M86.2	Subacute osteomyelitis
M86.3	Chronic multifocal osteomyelitis
M86.4	Chronic osteomyelitis with draining sinus
M86.5	Other chronic haematogenous osteomyelitis
M86.6	Other chronic osteomyelitis
M86.8	Other osteomyelitis
M86.9	Osteomyelitis, unspecified
N08.0	Glomerular disorders in infectious and parasitic diseases classified elsewhere
N10*	Acute tubulo-interstitial nephritis
N13.6	Pyonephrosis
N15.1	Renal and perinephric abscess
N16.0	Renal tubulo-interstitial disorders in infectious and parasitic diseases classified elsewhere
N29.0	Late syphilis of kidney
N29.1	Other disorders of kidney and ureter in infectious and parasitic diseases classified elsewhere
N30.0	Acute cystitis
N34.0	Urethral abscess
N39.0	Urinary tract infection, site not specified
N41.0	Acute prostatitis
N41.2	Abscess of prostate
N45*	Orchitis and epididymitis
N61*	Inflammatory disorders of breast
N70.0	Acute salpingitis and oophoritis
N71.0	Acute inflammatory disease of uterus
N73.0	Acute parametritis and pelvic cellulitis
N73.3	Female acute pelvic peritonitis
N74	Female pelvic inflammatory disorders in diseases classified elsewhere
N74.0	Tuberculous infection of cervix uteri
N74.1	Female tuberculous pelvic inflammatory disease
N74.2	Female syphilitic pelvic inflammatory disease
N74.3	Female gonococcal pelvic inflammatory disease
N74.4	Female chlamydial pelvic inflammatory disease
N74.8	Female pelvic inflammatory disorders in other diseases classified elsewhere
N75.1	Abscess of Bartholin gland
N76.0	Acute vaginitis
N76.4	Abscess of vulva
N77.0	Ulceration of vulva in infectious and parasitic diseases classified elsewhere
N77.1	Vaginitis, vulvitis and vulvovaginitis in infectious and parasitic diseases classified elsewhere
O03.0	Spontaneous abortion: Incomplete, complicated by genital tract and pelvic infection

O03.5	Spontaneous abortion: Complete or unspecified, complicated by genital tract and pelvic infection
O04.0	Medical abortion: Incomplete, complicated by genital tract and pelvic infection
O04.5	Medical abortion: Complete or unspecified, complicated by genital tract and pelvic infection
O05.0	Other abortion: Incomplete, complicated by genital tract and pelvic infection
O05.5	Other abortion: Complete or unspecified, complicated by genital tract and pelvic infection
O06.0	Unspecified abortion: Incomplete, complicated by genital tract and pelvic infection
O06.5	Unspecified abortion: Complete or unspecified, complicated by genital tract and pelvic infection
O07.0	Failed medical abortion, complicated by genital tract and pelvic infection
O07.5	Other and unspecified failed attempted abortion, complicated by genital tract and pelvic infection
O08.0	Genital tract and pelvic infection following abortion and ectopic and molar pregnancy
O23.0	Infections of kidney in pregnancy
O23.1	Infections of bladder in pregnancy
O23.2	Infections of urethra in pregnancy
O23.3	Infections of other parts of urinary tract in pregnancy
O23.4	Unspecified infection of urinary tract in pregnancy
O23.5	Infections of the genital tract in pregnancy
O23.9	Other and unspecified genitourinary tract infection in pregnancy
O75.3	Other infection during labour
O85*	Puerperal sepsis
O86*	Other puerperal infections
O91.0	Infection of nipple associated with childbirth
O91.1	Abscess of breast associated with childbirth
O98	Maternal infectious and parasitic diseases classifiable elsewhere but complicating pregnancy, childbirth and the puerperium
O98.0	Tuberculosis complicating pregnancy, childbirth and the puerperium
O98.1	Syphilis complicating pregnancy, childbirth and the puerperium
O98.2	Gonorrhoea complicating pregnancy, childbirth and the puerperium
O98.3	Other infections with a predominantly sexual mode of transmission complicating pregnancy, childbirth and the puerperium
O98.4	Viral hepatitis complicating pregnancy, childbirth and the puerperium
O98.5	Other viral diseases complicating pregnancy, childbirth and the puerperium
O98.6	Protozoal diseases complicating pregnancy, childbirth and the puerperium
O98.7	Human immunodeficiency virus [HIV] disease complicating pregnancy, childbirth and the puerperium
O98.8	Other maternal infectious and parasitic diseases complicating pregnancy, childbirth and the puerperium
O98.9	Unspecified maternal infectious or parasitic disease complicating pregnancy, childbirth and the puerperium
P23	Congenital pneumonia
P23.0	Congenital pneumonia due to viral agent
P23.1	Congenital pneumonia due to Chlamydia
P23.2	Congenital pneumonia due to staphylococcus
P23.3	Congenital pneumonia due to streptococcus, group B
P23.4	Congenital pneumonia due to Escherichia coli
P23.5	Congenital pneumonia due to Pseudomonas
P23.6	Congenital pneumonia due to other bacterial agents
P23.8	Congenital pneumonia due to other organisms
P23.9	Congenital pneumonia, unspecified
P35.2	Congenital herpesviral [herpes simplex] infection
P35.3	Congenital viral hepatitis
P36	Bacterial sepsis of newborn
P36.0	Sepsis of newborn due to streptococcus, group B

P36.1	Sepsis of newborn due to other and unspecified streptococci
P36.2	Sepsis of newborn due to Staphylococcus aureus
P36.3	Sepsis of newborn due to other and unspecified staphylococci
P36.4	Sepsis of newborn due to Escherichia coli
P36.5	Sepsis of newborn due to anaerobes
P36.8	Other bacterial sepsis of newborn
P36.9	Bacterial sepsis of newborn, unspecified
P37	Other congenital infectious and parasitic diseases
P37.0	Congenital tuberculosis
P37.1	Congenital toxoplasmosis
P37.2	Neonatal (disseminated) listeriosis
P37.3	Congenital falciparum malaria
P37.4	Other congenital malaria
P37.5	Neonatal candidiasis
P37.8	Other specified congenital infectious and parasitic diseases
P37.9	Congenital infectious and parasitic disease, unspecified
P38*	Omphalitis of newborn with or without mild haemorrhage
P39	Other infections specific to the perinatal period
P39.0	Neonatal infective mastitis
P39.1	Neonatal conjunctivitis and dacryocystitis
P39.2	Intra-amniotic infection of fetus, not elsewhere classified
P39.3	Neonatal urinary tract infection
P39.4	Neonatal skin infection
P39.8	Other specified infections specific to the perinatal period
P39.9	Infection specific to the perinatal period, unspecified
R57.2	Septic shock
R65.0	Systemic Inflammatory Response Syndrome of infectious origin without organ failure
R65.1	Systemic Inflammatory Response Syndrome of infectious origin with organ failure
Z21	Asymptomatic human immunodeficiency virus [HIV] infection status

Acute infections

A00*	Cholera
A01*	Typhoid and paratyphoid fevers
A02	Other salmonella infections
A02.0	Salmonella enteritis
A02.1	Salmonella sepsis
A02.2	Localized salmonella infections
A02.8	Other specified salmonella infections
A02.9	Salmonella infection, unspecified
A03	Shigellosis
A03.0	Shigellosis due to Shigella dysenteriae
A03.1	Shigellosis due to Shigella flexneri
A03.2	Shigellosis due to Shigella boydii
A03.3	Shigellosis due to Shigella sonnei
A03.8	Other shigellosis

A03.9	Shigellosis, unspecified
A04	Other bacterial intestinal infections
A04.0	Enteropathogenic Escherichia coli infection
A04.1	Enterotoxigenic Escherichia coli infection
A04.2	Enteroinvasive Escherichia coli infection
A04.3	Enterohaemorrhagic Escherichia coli infection
A04.4	Other intestinal Escherichia coli infections
A04.5	Campylobacter enteritis
A04.6	Enteritis due to Yersinia enterocolitica
A04.7	Enterocolitis due to Clostridium difficile
A04.8	Other specified bacterial intestinal infections
A04.9	Bacterial intestinal infection, unspecified
A05	Other bacterial foodborne intoxications, not elsewhere classified
A05.0	Foodborne staphylococcal intoxication
A05.1	Botulism
A05.2	Foodborne Clostridium perfringens [Clostridium welchii] intoxication
A05.3	Foodborne Vibrio parahaemolyticus intoxication
A05.4	Foodborne Bacillus cereus intoxication
A05.8	Other specified bacterial foodborne intoxications
A05.9	Bacterial foodborne intoxication, unspecified
A06	Amoebiasis
A06.0	Acute amoebic dysentery
A06.2	Amoebic nondysenteric colitis
A06.4	Amoebic liver abscess
A06.5	Amoebic lung abscess
A06.6	Amoebic brain abscess
A06.7	Cutaneous amoebiasis
A06.8	Amoebic infection of other sites
A06.9	Amoebiasis, unspecified
A07*	Other protozoal intestinal diseases
A08.0	Rotaviral enteritis
A08.1	Acute gastroenteropathy due to Norwalk agent
A08.2	Adenoviral enteritis
A08.3	Other viral enteritis
A08.4	Viral intestinal infection, unspecified
A08.5	Other specified intestinal infections
A09*	Other gastroenteritis and colitis of infectious and unspecified origin
A20	Plague
A20.0	Bubonic plague
A20.1	Cellulocutaneous plague
A20.2	Pneumonic plague
A20.3	Plague meningitis
A20.7	Septicaemic plague
A20.8	Other forms of plague
A20.9	Plague, unspecified
A21	Tularaemia
A21.0	Ulceroglandular tularaemia
A21.1	Oculoglandular tularaemia

A21.2	Pulmonary tularaemia
A21.3	Gastrointestinal tularaemia
A21.7	Generalized tularaemia
A21.8	Other forms of tularaemia
A21.9	Tularaemia, unspecified
A22	Anthrax
A22.0	Cutaneous anthrax
A22.1	Pulmonary anthrax
A22.2	Gastrointestinal anthrax
A22.7	Anthrax sepsis
A22.8	Other forms of anthrax
A22.9	Anthrax, unspecified
A23	Brucellosis
A23.0	Brucellosis due to Brucella melitensis
A23.1	Brucellosis due to Brucella abortus
A23.2	Brucellosis due to Brucella suis
A23.3	Brucellosis due to Brucella canis
A23.8	Other brucellosis
A23.9	Brucellosis, unspecified
A24	Glanders and melioidosis
A24.0	Glanders
A24.1	Acute and fulminating melioidosis
A24.2	Subacute and chronic melioidosis
A24.3	Other melioidosis
A25	Rat-bite fevers
A25.0	Spirillosis
A25.1	Streptobacillosis
A25.9	Rat-bite fever, unspecified
A26	Erysipeloid
A26.0	Cutaneous erysipeloid
A26.7	Erysipelothrix sepsis
A26.8	Other forms of erysipeloid
A26.9	Erysipeloid, unspecified
A27*	Leptospirosis
A28	Other zoonotic bacterial diseases, not elsewhere classified
A28.0	Pasteurellosis
A28.1	Cat-scratch disease
A28.2	Extraintestinal yersiniosis
A28.8	Other specified zoonotic bacterial diseases, not elsewhere classified
A28.9	Zoonotic bacterial disease, unspecified
A32	Listeriosis
A32.0	Cutaneous listeriosis
A32.1	Listerial meningitis and meningoencephalitis
A32.7	Listerial sepsis
A32.8	Other forms of listeriosis
A32.9	Listeriosis, unspecified
A33*	Tetanus neonatorum
A34*	Obstetrical tetanus

A35*	Other tetanus
A36*	Diphtheria
A37*	Whooping cough
A38*	Scarlet fever
A39	Meningococcal infection
A39.0	Meningococcal meningitis
A39.1	Waterhouse-Friderichsen syndrome
A39.2	Acute meningococcaemia
A39.4	Meningococcaemia, unspecified
A39.5	Meningococcal heart disease
A39.8	Other meningococcal infections
A39.9	Meningococcal infection, unspecified
A40	Streptococcal sepsis
A40.0	Sepsis due to streptococcus, group A
A40.1	Sepsis due to streptococcus, group B
A40.2	Sepsis due to streptococcus, group D
A40.3	Sepsis due to Streptococcus pneumoniae
A40.8	Other streptococcal sepsis
A40.9	Streptococcal sepsis, unspecified
A41	Other sepsis
A41.0	Sepsis due to Staphylococcus aureus
A41.1	Sepsis due to other specified staphylococcus
A41.2	Sepsis due to unspecified staphylococcus
A41.3	Sepsis due to Haemophilus influenzae
A41.4	Sepsis due to anaerobes
A41.5	Sepsis due to other Gram-negative organisms
A41.8	Other specified sepsis
A41.9	Sepsis, unspecified
A43	Nocardiosis
A43.0	Pulmonary nocardiosis
A43.1	Cutaneous nocardiosis
A43.8	Other forms of nocardiosis
A44	Bartonellosis
A44.0	Systemic bartonellosis
A44.8	Other forms of bartonellosis
A44.9	Bartonellosis, unspecified
A46*	Erysipelas
A48.0	Gas gangrene
A48.1	Legionnaires disease
A48.2	Nonpneumonic Legionnaires disease [Pontiac fever]
A48.3	Toxic shock syndrome
A48.4	Brazilian purpuric fever
A48.8	Other specified bacterial diseases
A49	Bacterial infection of unspecified site
A49.0	Staphylococcal infection, unspecified site
A49.1	Streptococcal infection, unspecified site
A49.2	Haemophilus influenzae infection, unspecified site
A49.3	Mycoplasma infection, unspecified site

A49.8	Other bacterial infections of unspecified site
A49.9	Bacterial infection, unspecified
A54	Gonococcal infection
A54.0	Gonococcal infection of lower genitourinary tract without periurethral or accessory gland abscess
A54.1	Gonococcal infection of lower genitourinary tract with periurethral and accessory gland abscess
A54.2	Gonococcal pelviperitonitis and other gonococcal genitourinary infections
A54.3	Gonococcal infection of eye
A54.4	Gonococcal infection of musculoskeletal system
A54.5	Gonococcal pharyngitis
A54.6	Gonococcal infection of anus and rectum
A54.8	Other gonococcal infections
A54.9	Gonococcal infection, unspecified
A55*	Chlamydial lymphogranuloma (venereum)
A56.0	Chlamydial infection of lower genitourinary tract
A56.1	Chlamydial infection of pelviperitoneum and other genitourinary organs
A56.2	Chlamydial infection of genitourinary tract, unspecified
A56.3	Chlamydial infection of anus and rectum
A56.4	Chlamydial infection of pharynx
A56.8	Sexually transmitted chlamydial infection of other sites
A57*	Chancroid
A59*	Trichomoniasis
A63.8	Other specified predominantly sexually transmitted diseases
A64*	Unspecified sexually transmitted disease
A68*	Relapsing fevers
A69	Other spirochaetal infections
A69.0	Necrotizing ulcerative stomatitis
A69.1	Other Vincent infections
A69.2	Lyme disease
A69.8	Other specified spirochaetal infections
A69.9	Spirochaetal infection, unspecified
A70*	Chlamydia psittaci infection
A71*	Trachoma
A74.0	Chlamydial conjunctivitis
A74.8	Other chlamydial diseases
A74.9	Chlamydial infection, unspecified
A75	Typhus fever
A75.0	Epidemic louse-borne typhus fever due to Rickettsia prowazekii
A75.2	Typhus fever due to Rickettsia typhi
A75.3	Typhus fever due to Rickettsia tsutsugamushi
A75.9	Typhus fever, unspecified
A77	Spotted fever [tick-borne rickettsioses]
A77.0	Spotted fever due to Rickettsia rickettsii
A77.1	Spotted fever due to Rickettsia conorii
A77.2	Spotted fever due to Rickettsia sibirica
A77.3	Spotted fever due to Rickettsia australis
A77.8	Other spotted fevers
A77.9	Spotted fever, unspecified
A78*	Q fever

A79	Other rickettsioses
A79.0	Trench fever
A79.1	Rickettsialpox due to Rickettsia akari
A79.8	Other specified rickettsioses
A79.9	Rickettsiosis, unspecified
A80*	Acute poliomyelitis
A83*	Mosquito-borne viral encephalitis
A84*	Tick-borne viral encephalitis
A85*	Other viral encephalitis, not elsewhere classified
A86*	Unspecified viral encephalitis
A87*	Viral meningitis
A88*	Other viral infections of central nervous system, not elsewhere classified
A89*	Unspecified viral infection of central nervous system
A90*	Dengue fever [classical dengue]
A91*	Dengue haemorrhagic fever
A92	Other mosquito-borne viral fevers
A92.0	Chikungunya virus disease
A92.1	O'nyong-nyong fever
A92.2	Venezuelan equine fever
A92.3	West Nile virus infection
A92.4	Rift Valley fever
A92.8	Other specified mosquito-borne viral fevers
A92.9	Mosquito-borne viral fever, unspecified
A93	Other arthropod-borne viral fevers, not elsewhere classified
A93.0	Oropouche virus disease
A93.1	Sandfly fever
A93.2	Colorado tick fever
A93.8	Other specified arthropod-borne viral fevers
A94*	Unspecified arthropod-borne viral fever
A95*	Yellow fever
A96*	Arenaviral haemorrhagic fever
A97*	Dengue
A98	Other viral haemorrhagic fevers, not elsewhere classified
A98.0	Crimean-Congo haemorrhagic fever
A98.1	Omsk haemorrhagic fever
A98.2	Kyasanur Forest disease
A98.3	Marburg virus disease
A98.4	Ebola virus disease
A98.5	Haemorrhagic fever with renal syndrome
A98.8	Other specified viral haemorrhagic fevers
A99*	Unspecified viral haemorrhagic fever
B04*	Monkeypox
B05	Measles
B05.0	Measles complicated by encephalitis
B05.1	Measles complicated by meningitis
B05.2	Measles complicated by pneumonia
B05.3	Measles complicated by otitis media
B05.4	Measles with intestinal complications

B05.8	Measles with other complications
B05.9	Measles without complication
B06	Rubella [German measles]
B06.0	Rubella with neurological complications
B06.8	Rubella with other complications
B06.9	Rubella without complication
B07*	Viral warts
B08.0	Other orthopoxvirus infections
B08.1	Molluscum contagiosum
B08.3	Erythema infectiosum [fifth disease]
B08.4	Enteroviral vesicular stomatitis with exanthem
B08.5	Enteroviral vesicular pharyngitis
B08.8	Other specified viral infections characterized by skin and mucous membrane lesions
B09*	Unspecified viral infection characterized by skin and mucous membrane lesions
B15	Acute hepatitis A
B15.0	Hepatitis A with hepatic coma
B15.9	Hepatitis A without hepatic coma
B16	Acute hepatitis B
B16.0	Acute hepatitis B with delta-agent (coinfection) with hepatic coma
B16.1	Acute hepatitis B with delta-agent (coinfection) without hepatic coma
B16.2	Acute hepatitis B without delta-agent with hepatic coma
B16.9	Acute hepatitis B without delta-agent and without hepatic coma
B17*	Other acute viral hepatitis
B19	Unspecified viral hepatitis
B19.0	Unspecified viral hepatitis with hepatic coma
B19.9	Unspecified viral hepatitis without hepatic coma
B26	Mumps
B26.0	Mumps orchitis
B26.1	Mumps meningitis
B26.2	Mumps encephalitis
B26.3	Mumps pancreatitis
B26.8	Mumps with other complications
B26.9	Mumps without complication
B27.8	Other infectious mononucleosis
B30*	Viral conjunctivitis
B33	Other viral diseases, not elsewhere classified
B33.0	Epidemic myalgia
B33.1	Ross River disease
B33.2	Viral carditis
B33.4	Hantavirus (cardio-)pulmonary syndrome [HPS] [HCPS]
B33.8	Other specified viral diseases
B34	Viral infection of unspecified site
B34.0	Adenovirus infection, unspecified site
B34.1	Enterovirus infection, unspecified site
B34.2	Coronavirus infection, unspecified site
B34.3	Parvovirus infection, unspecified site
B34.8	Other viral infections of unspecified site
B34.9	Viral infection, unspecified

B50*	Plasmodium falciparum malaria
B50.0	Plasmodium falciparum malaria with cerebral complications
B50.8	Other severe and complicated Plasmodium falciparum malaria
B50.9	Plasmodium falciparum malaria, unspecified
B52*	Plasmodium malariae malaria
B53	Other parasitologically confirmed malaria
B53.1	Malaria due to simian plasmodia
B53.8	Other parasitologically confirmed malaria, not elsewhere classified
B54*	Unspecified malaria
B55.1	Cutaneous leishmaniasis
B55.9	Leishmaniasis, unspecified
B60.0	Babesiosis
B60.1	Acanthamoebiasis
B60.2	Naegleriasis
B60.8	Other specified protozoal diseases
B64*	Unspecified protozoal disease
B66	Other fluke infections
B66.5	Fasciolopsiasis
B66.8	Other specified fluke infections
B66.9	Fluke infection, unspecified
B71.8	Other specified cestode infections
B81	Other intestinal helminthiases, not elsewhere classified
B81.0	Anisakiasis
B81.3	Intestinal angiostrongyliasis
B81.4	Mixed intestinal helminthiases
B81.8	Other specified intestinal helminthiases
B82	Unspecified intestinal parasitism
B82.0	Intestinal helminthiasis, unspecified
B82.9	Intestinal parasitism, unspecified
B83	Other helminthiases
B83.0	Visceral larva migrans
B83.2	Angiostrongyliasis due to Parastrongylus cantonensis
B83.4	Internal hirudiniasis
B83.8	Other specified helminthiases
B83.9	Helminthiasis, unspecified
B87*	Myiasis
B88	Other infestations
B88.0	Other acariasis
B88.1	Tungiasis [sandflea infestation]
B88.2	Other arthropod infestations
B88.3	External hirudiniasis
B88.8	Other specified infestations
B88.9	Infestation, unspecified
B89*	Unspecified parasitic disease
B95*	Streptococcus and staphylococcus as the cause of diseases classified to other chapters
B96.0	Mycoplasma pneumoniae [M. pneumoniae] as the cause of diseases classified to other chapters
B96.1	Klebsiella pneumoniae [K. pneumoniae] as the cause of diseases classified to other chapters
B96.2	Escherichia coli [E. coli] as the cause of diseases classified to other chapters

B96.3	Haemophilus influenzae [H. influenzae] as the cause of diseases classified to other chapters
B96.4	Proteus (mirabilis)(morganii) as the cause of diseases classified to other chapters
B96.5	Pseudomonas (aeruginosa) as the cause of diseases classified to other chapters
B96.6	Bacillus fragilis [B. fragilis] as the cause of diseases classified to other chapters
B96.7	Clostridium perfringens [C. perfringens] as the cause of diseases classified to other chapters
B96.8	Other specified bacterial agents as the cause of diseases classified to other chapters
B97	Viral agents as the cause of diseases classified to other chapters
B97.0	Adenovirus as the cause of diseases classified to other chapters
B97.1	Enterovirus as the cause of diseases classified to other chapters
B97.2	Coronavirus as the cause of diseases classified to other chapters
B97.4	Respiratory syncytial virus as the cause of diseases classified to other chapters
B97.5	Reovirus as the cause of diseases classified to other chapters
B97.6	Parvovirus as the cause of diseases classified to other chapters
B97.8	Other viral agents as the cause of diseases classified to other chapters
B98.1	Vibrio vulnificus as the cause of diseases classified to other chapters
B99*	Other and unspecified infectious diseases
D73.3	Abscess of spleen
E32.1	Abscess of thymus
G00	Bacterial meningitis, not elsewhere classified
G00.0	Haemophilus meningitis
G00.1	Pneumococcal meningitis
G00.2	Streptococcal meningitis
G00.3	Staphylococcal meningitis
G00.8	Other bacterial meningitis
G00.9	Bacterial meningitis, unspecified
G01*	Meningitis in bacterial diseases classified elsewhere
G02.0	Meningitis in viral diseases classified elsewhere
G02.8	Meningitis in other specified infectious and parasitic diseases classified elsewhere
G03*	Meningitis due to other and unspecified causes
G04.2	Bacterial meningoencephalitis and meningomyelitis, not elsewhere classified
G05.0	Encephalitis, myelitis and encephalomyelitis in bacterial diseases classified elsewhere
G05.1	Encephalitis, myelitis and encephalomyelitis in viral diseases classified elsewhere
G05.2	Encephalitis, myelitis and encephalomyelitis in other infectious and parasitic diseases classified elsewhere
G06*	Intracranial and intraspinal abscess and granuloma
G07*	Intracranial and intraspinal abscess and granuloma in diseases classified elsewhere
H00*	Hordeolum and chalazion
H01.0	Blepharitis
H05.0	Acute inflammation of orbit
H06.1	Parasitic infestation of orbit in diseases classified elsewhere
H10.0	Mucopurulent conjunctivitis
H10.5	Blepharoconjunctivitis
H19.0	Scleritis and episcleritis in diseases classified elsewhere
H19.2	Keratitis and keratoconjunctivitis in other infectious and parasitic diseases classified elsewhere
H22.0	Iridocyclitis in infectious and parasitic diseases classified elsewhere
H32.0	Chorioretinal inflammation in infectious and parasitic diseases classified elsewhere
H44.0	Purulent endophthalmitis
H60.0	Abscess of external ear
H60.1	Cellulitis of external ear

H60.2	Malignant otitis externa
H60.3	Other infective otitis externa
H62.0	Otitis externa in bacterial diseases classified elsewhere
H62.1	Otitis externa in viral diseases classified elsewhere
H62.3	Otitis externa in other infectious and parasitic diseases classified elsewhere
H66.0	Acute suppurative otitis media
H67.0	Otitis media in bacterial diseases classified elsewhere
H67.1	Otitis media in viral diseases classified elsewhere
H70.0	Acute mastoiditis
H75.0	Mastoiditis in infectious and parasitic diseases classified elsewhere
I30.1	Infective pericarditis
I32.0	Pericarditis in bacterial diseases classified elsewhere
I32.1	Pericarditis in other infectious and parasitic diseases classified elsewhere
I33.0	Acute and subacute infective endocarditis
I40.0	Infective myocarditis
I41.0	Myocarditis in bacterial diseases classified elsewhere
I41.1	Myocarditis in viral diseases classified elsewhere
I41.2	Myocarditis in other infectious and parasitic diseases classified elsewhere
I43.0	Cardiomyopathy in infectious and parasitic diseases classified elsewhere
I52.0	Other heart disorders in bacterial diseases classified elsewhere
I52.1	Other heart disorders in other infectious and parasitic diseases classified elsewhere
I68.1	Cerebral arteritis in infectious and parasitic diseases classified elsewhere
J01.0	Acute maxillary sinusitis
J02	Acute pharyngitis
J02.0	Streptococcal pharyngitis
J02.8	Acute pharyngitis due to other specified organisms
J02.9	Acute pharyngitis, unspecified
J03	Acute tonsillitis
J03.0	Streptococcal tonsillitis
J03.8	Acute tonsillitis due to other specified organisms
J03.9	Acute tonsillitis, unspecified
J04*	Acute laryngitis and tracheitis
J05.1	Acute epiglottitis
J09*	Influenza due to certain identified influenza virus
J10*	Influenza due to other identified influenza virus
J11*	Influenza, virus not identified
J12*	Viral pneumonia, not elsewhere classified
J13*	Pneumonia due to Streptococcus pneumoniae
J14*	Pneumonia due to Haemophilus influenzae
J15	Bacterial pneumonia, not elsewhere classified
J15.0	Pneumonia due to Klebsiella pneumoniae
J15.1	Pneumonia due to Pseudomonas
J15.2	Pneumonia due to staphylococcus
J15.3	Pneumonia due to streptococcus, group B
J15.4	Pneumonia due to other streptococci
J15.5	Pneumonia due to Escherichia coli
J15.6	Pneumonia due to other aerobic Gram-negative bacteria
J15.7	Pneumonia due to Mycoplasma pneumoniae

J15.8	Other bacterial pneumonia
J15.9	Bacterial pneumonia, unspecified
J16	Pneumonia due to other infectious organisms, not elsewhere classified
J16.0	Chlamydial pneumonia
J16.8	Pneumonia due to other specified infectious organisms
J17.0	Pneumonia in bacterial diseases classified elsewhere
J17.1	Pneumonia in viral diseases classified elsewhere
J17.8	Pneumonia in other diseases classified elsewhere
J18*	Pneumonia, organism unspecified
J20	Acute bronchitis
J20.0	Acute bronchitis due to Mycoplasma pneumoniae
J20.1	Acute bronchitis due to Haemophilus influenzae
J20.2	Acute bronchitis due to streptococcus
J20.3	Acute bronchitis due to coxsackievirus
J20.4	Acute bronchitis due to parainfluenza virus
J20.5	Acute bronchitis due to respiratory syncytial virus
J20.6	Acute bronchitis due to rhinovirus
J20.7	Acute bronchitis due to echovirus
J20.8	Acute bronchitis due to other specified organisms
J20.9	Acute bronchitis, unspecified
J21*	Acute bronchiolitis
J21.0	Acute bronchiolitis due to respiratory syncytial virus
J21.1	Acute bronchiolitis due to human metapneumovirus
J21.8	Acute bronchiolitis due to other specified organisms
J21.9	Acute bronchiolitis, unspecified
J22*	Unspecified acute lower respiratory infection
J36*	Peritonsillar abscess
J39.0	Retropharyngeal and parapharyngeal abscess
J39.1	Other abscess of pharynx
J85.1	Abscess of lung with pneumonia
J85.2	Abscess of lung without pneumonia
J85.3	Abscess of mediastinum
J86*	Pyothorax
K04.4	Acute apical periodontitis of pulpal origin
K04.7	Periapical abscess without sinus
K05.0	Acute gingivitis
K05.2	Acute periodontitis
K11.3	Abscess of salivary gland
K12.2	Cellulitis and abscess of mouth
K35*	Acute appendicitis
K57.0	Diverticular disease of small intestine with perforation and abscess
K57.2	Diverticular disease of large intestine with perforation and abscess
K57.4	Diverticular disease of both small and large intestine with perforation and abscess
K57.8	Diverticular disease of intestine, part unspecified, with perforation and abscess
K61*	Abscess of anal and rectal regions
K63.0	Abscess of intestine
K65.0	Acute peritonitis
K67.0	Chlamydial peritonitis

K67.1	Gonococcal peritonitis
K67.8	Other disorders of peritoneum in infectious diseases classified elsewhere
K75.0	Abscess of liver
K77.0	Liver disorders in infectious and parasitic diseases classified elsewhere
L00*	Staphylococcal scalded skin syndrome
L01*	Impetigo
L02*	Cutaneous abscess, furuncle and carbuncle
L03*	Cellulitis
L04*	Acute lymphadenitis
L05*	Pilonidal cyst
L08*	Other local infections of skin and subcutaneous tissue
M00	Pyogenic arthritis
M00.0	Staphylococcal arthritis and polyarthritis
M00.1	Pneumococcal arthritis and polyarthritis
M00.2	Other streptococcal arthritis and polyarthritis
M00.8	Arthritis and polyarthritis due to other specified bacterial agents
M00.9	Pyogenic arthritis, unspecified
M01.0	Meningococcal arthritis
M01.2	Arthritis in Lyme disease
M01.3	Arthritis in other bacterial diseases classified elsewhere
M01.4	Rubella arthritis
M01.5	Arthritis in other viral diseases classified elsewhere
M01.8	Arthritis in other infectious and parasitic diseases classified elsewhere
M46.2	Osteomyelitis of vertebra
M46.3	Infection of intervertebral disc (pyogenic)
M46.5	Other infective spondylopathies
M49.1	Brucella spondylitis
M49.2	Enterobacterial spondylitis
M49.3	Spondylopathy in other infectious and parasitic diseases classified elsewhere
M60.0	Infective myositis
M63.0	Myositis in bacterial diseases classified elsewhere
M63.2	Myositis in other infectious diseases classified elsewhere
M65.0	Abscess of tendon sheath
M65.1	Other infective (teno)synovitis
M71.0	Abscess of bursa
M71.1	Other infective bursitis
M72.6	Necrotizing fasciitis
M73.0	Gonococcal bursitis
M86*	Osteomyelitis
M86.0	Acute haematogenous osteomyelitis
M86.1	Other acute osteomyelitis
M86.9	Osteomyelitis, unspecified
N08.0	Glomerular disorders in infectious and parasitic diseases classified elsewhere
N10*	Acute tubulo-interstitial nephritis
N13.6	Pyonephrosis
N15.1	Renal and perinephric abscess
N16.0	Renal tubulo-interstitial disorders in infectious and parasitic diseases classified elsewhere
N29.1	Other disorders of kidney and ureter in infectious and parasitic diseases classified elsewhere

N30.0	Acute cystitis
N34.0	Urethral abscess
N39.0	Urinary tract infection, site not specified
N41.0	Acute prostatitis
N41.2	Abscess of prostate
N45*	Orchitis and epididymitis
N61*	Inflammatory disorders of breast
N70.0	Acute salpingitis and oophoritis
N71.0	Acute inflammatory disease of uterus
N73.0	Acute parametritis and pelvic cellulitis
N73.3	Female acute pelvic peritonitis
N74	Female pelvic inflammatory disorders in diseases classified elsewhere
N74.3	Female gonococcal pelvic inflammatory disease
N74.4	Female chlamydial pelvic inflammatory disease
N74.8	Female pelvic inflammatory disorders in other diseases classified elsewhere
N75.1	Abscess of Bartholin gland
N76.0	Acute vaginitis
N76.4	Abscess of vulva
N77.0	Ulceration of vulva in infectious and parasitic diseases classified elsewhere
N77.1	Vaginitis, vulvitis and vulvovaginitis in infectious and parasitic diseases classified elsewhere
O03.0	Spontaneous abortion: Incomplete, complicated by genital tract and pelvic infection
O03.5	Spontaneous abortion: Complete or unspecified, complicated by genital tract and pelvic infection
O04.0	Medical abortion: Incomplete, complicated by genital tract and pelvic infection
O04.5	Medical abortion: Complete or unspecified, complicated by genital tract and pelvic infection
O05.0	Other abortion: Incomplete, complicated by genital tract and pelvic infection
O05.5	Other abortion: Complete or unspecified, complicated by genital tract and pelvic infection
O06.0	Unspecified abortion: Incomplete, complicated by genital tract and pelvic infection
O06.5	Unspecified abortion: Complete or unspecified, complicated by genital tract and pelvic infection
O07.0	Failed medical abortion, complicated by genital tract and pelvic infection
O07.5	Other and unspecified failed attempted abortion, complicated by genital tract and pelvic infection
O08.0	Genital tract and pelvic infection following abortion and ectopic and molar pregnancy
O23.0	Infections of kidney in pregnancy
O23.1	Infections of bladder in pregnancy
O23.2	Infections of urethra in pregnancy
O23.3	Infections of other parts of urinary tract in pregnancy
O23.4	Unspecified infection of urinary tract in pregnancy
O23.5	Infections of the genital tract in pregnancy
O23.9	Other and unspecified genitourinary tract infection in pregnancy
O75.3	Other infection during labour
O85*	Puerperal sepsis
O86*	Other puerperal infections
O91.0	Infection of nipple associated with childbirth
O91.1	Abscess of breast associated with childbirth
O98	Maternal infectious and parasitic diseases classifiable elsewhere but complicating pregnancy, childbirth and the puerperium
O98.2	Gonorrhoea complicating pregnancy, childbirth and the puerperium
O98.3	Other infections with a predominantly sexual mode of transmission complicating pregnancy, childbirth and the puerperium
O98.4	Viral hepatitis complicating pregnancy, childbirth and the puerperium
O98.5	Other viral diseases complicating pregnancy, childbirth and the puerperium

O98.6	Protozoal diseases complicating pregnancy, childbirth and the puerperium
O98.8	Other maternal infectious and parasitic diseases complicating pregnancy, childbirth and the puerperium
O98.9	Unspecified maternal infectious or parasitic disease complicating pregnancy, childbirth and the puerperium
P23	Congenital pneumonia
P23.0	Congenital pneumonia due to viral agent
P23.1	Congenital pneumonia due to Chlamydia
P23.2	Congenital pneumonia due to staphylococcus
P23.3	Congenital pneumonia due to streptococcus, group B
P23.4	Congenital pneumonia due to Escherichia coli
P23.5	Congenital pneumonia due to Pseudomonas
P23.6	Congenital pneumonia due to other bacterial agents
P23.8	Congenital pneumonia due to other organisms
P23.9	Congenital pneumonia, unspecified
P35.0	Congenital rubella syndrome
P36	Bacterial sepsis of newborn
P36.0	Sepsis of newborn due to streptococcus, group B
P36.1	Sepsis of newborn due to other and unspecified streptococci
P36.2	Sepsis of newborn due to Staphylococcus aureus
P36.3	Sepsis of newborn due to other and unspecified staphylococci
P36.4	Sepsis of newborn due to Escherichia coli
P36.5	Sepsis of newborn due to anaerobes
P36.8	Other bacterial sepsis of newborn
P36.9	Bacterial sepsis of newborn, unspecified
P37	Other congenital infectious and parasitic diseases
P37.2	Neonatal (disseminated) listeriosis
P37.3	Congenital falciparum malaria
P37.4	Other congenital malaria
P37.8	Other specified congenital infectious and parasitic diseases
P37.9	Congenital infectious and parasitic disease, unspecified
P38*	Omphalitis of newborn with or without mild haemorrhage
P39	Other infections specific to the perinatal period
P39.0	Neonatal infective mastitis
P39.1	Neonatal conjunctivitis and dacryocystitis
P39.2	Intra-amniotic infection of fetus, not elsewhere classified
P39.3	Neonatal urinary tract infection
P39.4	Neonatal skin infection
P39.8	Other specified infections specific to the perinatal period
P39.9	Infection specific to the perinatal period, unspecified
R57.2	Septic shock
R65.0	Systemic Inflammatory Response Syndrome of infectious origin without organ failure
R65.1	Systemic Inflammatory Response Syndrome of infectious origin with organ failure

Chronic infections

A06.1	Chronic intestinal amoebiasis
A06.3	Amoeboma of intestine

A15*	Respiratory tuberculosis, bacteriologically and histologically confirmed
A16*	Respiratory tuberculosis, not confirmed bacteriologically or histologically
A17	Tuberculosis of nervous system
A17.0	Tuberculous meningitis
A17.1	Meningeal tuberculoma
A17.8	Other tuberculosis of nervous system
A17.9	Tuberculosis of nervous system, unspecified
A18*	Tuberculosis of other organs
A19*	Miliary tuberculosis
A24.2	Subacute and chronic melioidosis
A30*	Leprosy [Hansen disease]
A31*	Infection due to other mycobacteria
A39.3	Chronic meningococcaemia
A42	Actinomycosis
A42.0	Pulmonary actinomycosis
A42.1	Abdominal actinomycosis
A42.2	Cervicofacial actinomycosis
A42.7	Actinomycotic sepsis
A42.8	Other forms of actinomycosis
A42.9	Actinomycosis, unspecified
A44.1	Cutaneous and mucocutaneous bartonellosis
A50	Congenital syphilis
A50.0	Early congenital syphilis, symptomatic
A50.1	Early congenital syphilis, latent
A50.2	Early congenital syphilis, unspecified
A50.3	Late congenital syphilitic oculopathy
A50.4	Late congenital neurosyphilis [juvenile neurosyphilis]
A50.5	Other late congenital syphilis, symptomatic
A50.6	Late congenital syphilis, latent
A50.7	Late congenital syphilis, unspecified
A50.9	Congenital syphilis, unspecified
A51	Early syphilis
A51.0	Primary genital syphilis
A51.1	Primary anal syphilis
A51.2	Primary syphilis of other sites
A51.3	Secondary syphilis of skin and mucous membranes
A51.4	Other secondary syphilis
A51.5	Early syphilis, latent
A51.9	Early syphilis, unspecified
A52	Late syphilis
A52.0	Cardiovascular syphilis
A52.1	Symptomatic neurosyphilis
A52.2	Asymptomatic neurosyphilis
A52.3	Neurosyphilis, unspecified
A52.7	Other symptomatic late syphilis
A52.8	Late syphilis, latent
A52.9	Late syphilis, unspecified
A53*	Other and unspecified syphilis

A58*	Granuloma inguinale
A60*	Anogenital herpesviral [herpes simplex] infection
A63.0	Anogenital (venereal) warts
A65*	Nonvenereal syphilis
A66*	Yaws
A67*	Pinta [carate]
A75.1	Recrudescent typhus [Brill disease]
A81.1	Subacute sclerosing panencephalitis
A81.2	Progressive multifocal leukoencephalopathy
B00	Herpesviral [herpes simplex] infections
B00.0	Eczema herpeticum
B00.1	Herpesviral vesicular dermatitis
B00.2	Herpesviral gingivostomatitis and pharyngotonsillitis
B00.3	Herpesviral meningitis
B00.4	Herpesviral encephalitis
B00.5	Herpesviral ocular disease
B00.7	Disseminated herpesviral disease
B00.8	Other forms of herpesviral infection
B00.9	Herpesviral infection, unspecified
B01	Varicella [chickenpox]
B01.0	Varicella meningitis
B01.1	Varicella encephalitis
B01.2	Varicella pneumonia
B01.8	Varicella with other complications
B01.9	Varicella without complication
B02	Zoster [herpes zoster]
B02.0	Zoster encephalitis
B02.1	Zoster meningitis
B02.2	Zoster with other nervous system involvement
B02.3	Zoster ocular disease
B02.7	Disseminated zoster
B02.8	Zoster with other complications
B02.9	Zoster without complication
B08.2	Exanthema subitum [sixth disease]
B18*	Chronic viral hepatitis
B20*	Human immunodeficiency virus [HIV] disease resulting in infectious and parasitic diseases
B21*	Human immunodeficiency virus [HIV] disease resulting in malignant neoplasms
B21.0	HIV disease resulting in Kaposi sarcoma
B22	Human immunodeficiency virus [HIV] disease resulting in other specified diseases
B22.0	HIV disease resulting in encephalopathy
B22.1	HIV disease resulting in lymphoid interstitial pneumonitis
B22.2	HIV disease resulting in wasting syndrome
B22.7	HIV disease resulting in multiple diseases classified elsewhere
B23*	Human immunodeficiency virus [HIV] disease resulting in other conditions
B24*	Unspecified human immunodeficiency virus [HIV] disease
B25*	Cytomegaloviral disease
B27	Infectious mononucleosis
B27.0	Gammaherpesviral mononucleosis

B27.1	Cytomegaloviral mononucleosis
B27.9	Infectious mononucleosis, unspecified
B33.3	Retrovirus infections, not elsewhere classified
B34.4	Papovavirus infection, unspecified site
B35*	Dermatophytosis
B36*	Other superficial mycoses
B37	Candidiasis
B37.0	Candidal stomatitis
B37.1	Pulmonary candidiasis
B37.2	Candidiasis of skin and nail
B37.3	Candidiasis of vulva and vagina
B37.4	Candidiasis of other urogenital sites
B37.5	Candidal meningitis
B37.6	Candidal endocarditis
B37.7	Candidal sepsis
B37.8	Candidiasis of other sites
B37.9	Candidiasis, unspecified
B38.1	Chronic pulmonary coccidioidomycosis
B38.3	Cutaneous coccidioidomycosis
B38.4	Coccidioidomycosis meningitis
B38.7	Disseminated coccidioidomycosis
B38.8	Other forms of coccidioidomycosis
B39.1	Chronic pulmonary histoplasmosis capsulati
B39.3	Disseminated histoplasmosis capsulati
B39.5	Histoplasmosis duboisii
B40.1	Chronic pulmonary blastomycosis
B40.3	Cutaneous blastomycosis
B40.7	Disseminated blastomycosis
B40.8	Other forms of blastomycosis
B41	Paracoccidioidomycosis
B41.0	Pulmonary paracoccidioidomycosis
B41.7	Disseminated paracoccidioidomycosis
B41.8	Other forms of paracoccidioidomycosis
B41.9	Paracoccidioidomycosis, unspecified
B42	Sporotrichosis
B42.0	Pulmonary sporotrichosis
B42.1	Lymphocutaneous sporotrichosis
B42.7	Disseminated sporotrichosis
B42.8	Other forms of sporotrichosis
B42.9	Sporotrichosis, unspecified
B43	Chromomycosis and phaeomycotic abscess
B43.0	Cutaneous chromomycosis
B43.1	Phaeomycotic brain abscess
B43.2	Subcutaneous phaeomycotic abscess and cyst
B43.8	Other forms of chromomycosis
B43.9	Chromomycosis, unspecified
B44*	Aspergillosis
B45.1	Cerebral cryptococcosis

B45.2	Cutaneous cryptococcosis
B45.3	Osseous cryptococcosis
B45.7	Disseminated cryptococcosis
B45.8	Other forms of cryptococcosis
B47	Mycetoma
B47.0	Eumycetoma
B47.1	Actinomycetoma
B47.9	Mycetoma, unspecified
B48.0	Lobomycosis
B48.1	Rhinosporidiosis
B48.2	Allescheriasis
B48.3	Geotrichosis
B48.4	Penicillosis
B48.7	Opportunistic mycoses
B51*	Plasmodium vivax malaria
B53.0	Plasmodium ovale malaria
B55.0	Visceral leishmaniasis
B55.2	Mucocutaneous leishmaniasis
B56*	African trypanosomiasis
B57*	Chagas disease
B58	Toxoplasmosis
B58.0	Toxoplasma oculopathy
B58.1	Toxoplasma hepatitis
B58.2	Toxoplasma meningoencephalitis
B58.3	Pulmonary toxoplasmosis
B58.8	Toxoplasmosis with other organ involvement
B58.9	Toxoplasmosis, unspecified
B65	Schistosomiasis [bilharziasis]
B65.0	Schistosomiasis due to Schistosoma haematobium [urinary schistosomiasis]
B65.1	Schistosomiasis due to Schistosoma mansoni [intestinal schistosomiasis]
B65.2	Schistosomiasis due to Schistosoma japonicum
B65.3	Cercarial dermatitis
B65.8	Other schistosomiases
B65.9	Schistosomiasis, unspecified
B66.0	Opisthorchiasis
B66.1	Clonorchiasis
B66.2	Dicrocoeliasis
B66.3	Fascioliasis
B66.4	Paragonimiasis
B67*	Echinococcosis
B68	Taeniasis
B68.0	Taenia solium taeniasis
B68.1	Taenia saginata taeniasis
B68.9	Taeniasis, unspecified
B69	Cysticercosis
B69.0	Cysticercosis of central nervous system
B69.1	Cysticercosis of eye
B69.8	Cysticercosis of other sites

B69.9	Cysticercosis, unspecified
B70	Diphyllobothriasis and sparganosis
B70.0	Diphyllobothriasis
B70.1	Sparganosis
B71	Other cestode infections
B71.0	Hymenolepiasis
B71.1	Dipylidiasis
B71.9	Cestode infection, unspecified
B72*	Dracunculiasis
B73*	Onchocerciasis
B74*	Filariasis
B75*	Trichinellosis
B76*	Hookworm diseases
B77*	Ascariasis
B78*	Strongyloidiasis
B79*	Trichuriasis
B80*	Enterobiasis
B81.1	Intestinal capillariasis
B81.2	Trichostrongyliasis
B83.1	Gnathostomiasis
B83.3	Syngamiasis
B85*	Pediculosis and phthiriasis
B86*	Scabies
B97.3	Retrovirus as the cause of diseases classified to other chapters
B97.7	Papillomavirus as the cause of diseases classified to other chapters
B98.0	Helicobacter pylori [H.pylori] as the cause of diseases classified to other chapters
C46	Kaposi sarcoma
G02.1	Meningitis in mycoses
G04.1	Tropical spastic paraplegia
H13.0	Filarial infection of conjunctiva
H19.1	Herpesviral keratitis and keratoconjunctivitis
H62.2	Otitis externa in mycoses
J17.3	Pneumonia in parasitic diseases
K02*	Dental caries
K04.5	Chronic apical periodontitis
K04.6	Periapical abscess with sinus
K05.3	Chronic periodontitis
K05.4	Periodontosis
K23.0	Tuberculous oesophagitis
K23.1	Megaoesophagus in Chagas disease
K67.2	Syphilitic peritonitis
K67.3	Tuberculous peritonitis
L70.1	Acne conglobata
M01.1	Tuberculous arthritis
M49.0	Tuberculosis of spine
M63.1	Myositis in protozoal and parasitic infections classified elsewhere
M73.1	Syphilitic bursitis
M86.2	Subacute osteomyelitis

M86.3	Chronic multifocal osteomyelitis
M86.4	Chronic osteomyelitis with draining sinus
M86.5	Other chronic haematogenous osteomyelitis
M86.6	Other chronic osteomyelitis
M86.8	Other osteomyelitis
N29.0	Late syphilis of kidney
N74.0	Tuberculous infection of cervix uteri
N74.1	Female tuberculous pelvic inflammatory disease
N74.2	Female syphilitic pelvic inflammatory disease
O98.0	Tuberculosis complicating pregnancy, childbirth and the puerperium
O98.1	Syphilis complicating pregnancy, childbirth and the puerperium
O98.7	Human immunodeficiency virus [HIV] disease complicating pregnancy, childbirth and the puerperium
P35.1	Congenital cytomegalovirus infection
P35.2	Congenital herpesviral [herpes simplex] infection
P35.3	Congenital viral hepatitis
P37.0	Congenital tuberculosis
P37.1	Congenital toxoplasmosis
P37.5	Neonatal candidiasis
Z21	Asymptomatic human immunodeficiency virus [HIV] infection status

Infections predisposed towards entering the central nervous system

A02.2	Localized salmonella infections
A06	Amoebiasis
A06.0	Acute amoebic dysentery
A06.1	Chronic intestinal amoebiasis
A06.2	Amoebic nondysenteric colitis
A06.3	Amoeboma of intestine
A06.4	Amoebic liver abscess
A06.5	Amoebic lung abscess
A06.6	Amoebic brain abscess
A06.7	Cutaneous amoebiasis
A06.8	Amoebic infection of other sites
A06.9	Amoebiasis, unspecified
A08.2	Adenoviral enteritis
A15*	Respiratory tuberculosis, bacteriologically and histologically confirmed
A16*	Respiratory tuberculosis, not confirmed bacteriologically or histologically
A17	Tuberculosis of nervous system
A17.0	Tuberculous meningitis
A17.1	Meningeal tuberculoma
A17.8	Other tuberculosis of nervous system
A17.9	Tuberculosis of nervous system, unspecified
A18*	Tuberculosis of other organs
A19*	Miliary tuberculosis
A20	Plague
A20.0	Bubonic plague

A20.1	Cellulocutaneous plague
A20.2	Pneumonic plague
A20.3	Plague meningitis
A20.7	Septicaemic plague
A20.8	Other forms of plague
A20.9	Plague, unspecified
A22	Anthrax
A22.0	Cutaneous anthrax
A22.1	Pulmonary anthrax
A22.2	Gastrointestinal anthrax
A22.7	Anthrax sepsis
A22.8	Other forms of anthrax
A22.9	Anthrax, unspecified
A23	Brucellosis
A23.0	Brucellosis due to Brucella melitensis
A23.1	Brucellosis due to Brucella abortus
A23.2	Brucellosis due to Brucella suis
A23.3	Brucellosis due to Brucella canis
A23.8	Other brucellosis
A23.9	Brucellosis, unspecified
A24	Glanders and melioidosis
A24.1	Acute and fulminating melioidosis
A24.2	Subacute and chronic melioidosis
A24.3	Other melioidosis
A25	Rat-bite fevers
A25.1	Streptobacillosis
A25.9	Rat-bite fever, unspecified
A27*	Leptospirosis
A28.0	Pasteurellosis
A28.1	Cat-scratch disease
A32	Listeriosis
A32.0	Cutaneous listeriosis
A32.1	Listerial meningitis and meningoencephalitis
A32.7	Listerial sepsis
A32.8	Other forms of listeriosis
A32.9	Listeriosis, unspecified
A39	Meningococcal infection
A39.0	Meningococcal meningitis
A39.1	Waterhouse-Friderichsen syndrome
A39.2	Acute meningococcaemia
A39.3	Chronic meningococcaemia
A39.4	Meningococcaemia, unspecified
A39.5	Meningococcal heart disease
A39.8	Other meningococcal infections
A39.9	Meningococcal infection, unspecified
A40.3	Sepsis due to Streptococcus pneumoniae
A41.3	Sepsis due to Haemophilus influenzae
A42	Actinomycosis

A42.0	Pulmonary actinomycosis
A42.1	Abdominal actinomycosis
A42.2	Cervicofacial actinomycosis
A42.7	Actinomycotic sepsis
A42.8	Other forms of actinomycosis
A42.9	Actinomycosis, unspecified
A43	Nocardiosis
A43.0	Pulmonary nocardiosis
A43.1	Cutaneous nocardiosis
A43.8	Other forms of nocardiosis
A44	Bartonellosis
A44.0	Systemic bartonellosis
A44.1	Cutaneous and mucocutaneous bartonellosis
A44.8	Other forms of bartonellosis
A44.9	Bartonellosis, unspecified
A49.2	Haemophilus influenzae infection, unspecified site
A50	Congenital syphilis
A50.0	Early congenital syphilis, symptomatic
A50.1	Early congenital syphilis, latent
A50.2	Early congenital syphilis, unspecified
A50.3	Late congenital syphilitic oculopathy
A50.4	Late congenital neurosyphilis [juvenile neurosyphilis]
A50.5	Other late congenital syphilis, symptomatic
A50.6	Late congenital syphilis, latent
A50.7	Late congenital syphilis, unspecified
A50.9	Congenital syphilis, unspecified
A51	Early syphilis
A51.0	Primary genital syphilis
A51.1	Primary anal syphilis
A51.2	Primary syphilis of other sites
A51.3	Secondary syphilis of skin and mucous membranes
A51.4	Other secondary syphilis
A51.5	Early syphilis, latent
A51.9	Early syphilis, unspecified
A52	Late syphilis
A52.0	Cardiovascular syphilis
A52.1	Symptomatic neurosyphilis
A52.2	Asymptomatic neurosyphilis
A52.3	Neurosyphilis, unspecified
A52.7	Other symptomatic late syphilis
A52.8	Late syphilis, latent
A52.9	Late syphilis, unspecified
A53*	Other and unspecified syphilis
A60*	Anogenital herpesviral [herpes simplex] infection
A68*	Relapsing fevers
A69	Other spirochaetal infections
A69.2	Lyme disease
A69.8	Other specified spirochaetal infections

A69.9	Spirochaetal infection, unspecified
A70*	Chlamydia psittaci infection
A75	Typhus fever
A75.0	Epidemic louse-borne typhus fever due to Rickettsia prowazekii
A75.1	Recrudescent typhus [Brill disease]
A75.2	Typhus fever due to Rickettsia typhi
A75.3	Typhus fever due to Rickettsia tsutsugamushi
A75.9	Typhus fever, unspecified
A77	Spotted fever [tick-borne rickettsioses]
A77.0	Spotted fever due to Rickettsia rickettsii
A77.1	Spotted fever due to Rickettsia conorii
A77.2	Spotted fever due to Rickettsia sibirica
A77.3	Spotted fever due to Rickettsia australis
A77.8	Other spotted fevers
A77.9	Spotted fever, unspecified
A78*	Q fever
A79	Other rickettsioses
A79.1	Rickettsialpox due to Rickettsia akari
A79.8	Other specified rickettsioses
A79.9	Rickettsiosis, unspecified
A80*	Acute poliomyelitis
A81.1	Subacute sclerosing panencephalitis
A81.2	Progressive multifocal leukoencephalopathy
A83*	Mosquito-borne viral encephalitis
A84*	Tick-borne viral encephalitis
A85*	Other viral encephalitis, not elsewhere classified
A86*	Unspecified viral encephalitis
A87*	Viral meningitis
A88*	Other viral infections of central nervous system, not elsewhere classified
A89*	Unspecified viral infection of central nervous system
A90*	Dengue fever [classical dengue]
A91*	Dengue haemorrhagic fever
A92.0	Chikungunya virus disease
A92.2	Venezuelan equine fever
A92.3	West Nile virus infection
A92.4	Rift Valley fever
A93.1	Sandfly fever
A93.2	Colorado tick fever
A94*	Unspecified arthropod-borne viral fever
A96*	Arenaviral haemorrhagic fever
A97*	Dengue
A98.1	Omsk haemorrhagic fever
A98.2	Kyasanur Forest disease
A98.3	Marburg virus disease
A98.4	Ebola virus disease
B00	Herpesviral [herpes simplex] infections
B00.0	Eczema herpeticum
B00.1	Herpesviral vesicular dermatitis

B00.2	Herpesviral gingivostomatitis and pharyngotonsillitis
B00.3	Herpesviral meningitis
B00.4	Herpesviral encephalitis
B00.5	Herpesviral ocular disease
B00.7	Disseminated herpesviral disease
B00.8	Other forms of herpesviral infection
B00.9	Herpesviral infection, unspecified
B01	Varicella [chickenpox]
B01.0	Varicella meningitis
B01.1	Varicella encephalitis
B01.2	Varicella pneumonia
B01.8	Varicella with other complications
B01.9	Varicella without complication
B02	Zoster [herpes zoster]
B02.0	Zoster encephalitis
B02.1	Zoster meningitis
B02.2	Zoster with other nervous system involvement
B02.3	Zoster ocular disease
B02.7	Disseminated zoster
B02.8	Zoster with other complications
B02.9	Zoster without complication
B04*	Monkeypox
B05	Measles
B05.0	Measles complicated by encephalitis
B05.1	Measles complicated by meningitis
B05.2	Measles complicated by pneumonia
B05.3	Measles complicated by otitis media
B05.4	Measles with intestinal complications
B05.8	Measles with other complications
B05.9	Measles without complication
B06	Rubella [German measles]
B06.0	Rubella with neurological complications
B06.8	Rubella with other complications
B06.9	Rubella without complication
B08.2	Exanthema subitum [sixth disease]
B08.4	Enteroviral vesicular stomatitis with exanthem
B08.5	Enteroviral vesicular pharyngitis
B15	Acute hepatitis A
B15.0	Hepatitis A with hepatic coma
B15.9	Hepatitis A without hepatic coma
B16	Acute hepatitis B
B16.0	Acute hepatitis B with delta-agent (coinfection) with hepatic coma
B16.1	Acute hepatitis B with delta-agent (coinfection) without hepatic coma
B16.2	Acute hepatitis B without delta-agent with hepatic coma
B16.9	Acute hepatitis B without delta-agent and without hepatic coma
B17*	Other acute viral hepatitis
B18*	Chronic viral hepatitis
B19	Unspecified viral hepatitis

B19.0	Unspecified viral hepatitis with hepatic coma
B19.9	Unspecified viral hepatitis without hepatic coma
B20*	Human immunodeficiency virus [HIV] disease resulting in infectious and parasitic diseases
B21*	Human immunodeficiency virus [HIV] disease resulting in malignant neoplasms
B21.0	HIV disease resulting in Kaposi sarcoma
B22	Human immunodeficiency virus [HIV] disease resulting in other specified diseases
B22.0	HIV disease resulting in encephalopathy
B22.1	HIV disease resulting in lymphoid interstitial pneumonitis
B22.2	HIV disease resulting in wasting syndrome
B22.7	HIV disease resulting in multiple diseases classified elsewhere
B23*	Human immunodeficiency virus [HIV] disease resulting in other conditions
B24*	Unspecified human immunodeficiency virus [HIV] disease
B25*	Cytomegaloviral disease
B26	Mumps
B26.0	Mumps orchitis
B26.1	Mumps meningitis
B26.2	Mumps encephalitis
B26.3	Mumps pancreatitis
B26.8	Mumps with other complications
B26.9	Mumps without complication
B27	Infectious mononucleosis
B27.0	Gammaherpesviral mononucleosis
B27.1	Cytomegaloviral mononucleosis
B27.9	Infectious mononucleosis, unspecified
B33.3	Retrovirus infections, not elsewhere classified
B34.0	Adenovirus infection, unspecified site
B34.1	Enterovirus infection, unspecified site
B37	Candidiasis
B37.0	Candidal stomatitis
B37.1	Pulmonary candidiasis
B37.2	Candidiasis of skin and nail
B37.3	Candidiasis of vulva and vagina
B37.4	Candidiasis of other urogenital sites
B37.5	Candidal meningitis
B37.6	Candidal endocarditis
B37.7	Candidal sepsis
B37.8	Candidiasis of other sites
B37.9	Candidiasis, unspecified
B38	Coccidioidomycosis
B38.0	Acute pulmonary coccidioidomycosis
B38.1	Chronic pulmonary coccidioidomycosis
B38.2	Pulmonary coccidioidomycosis, unspecified
B38.3	Cutaneous coccidioidomycosis
B38.4	Coccidioidomycosis meningitis
B38.7	Disseminated coccidioidomycosis
B38.8	Other forms of coccidioidomycosis
B38.9	Coccidioidomycosis, unspecified
B39	Histoplasmosis

B39.0	Acute pulmonary histoplasmosis capsulati
B39.1	Chronic pulmonary histoplasmosis capsulati
B39.2	Pulmonary histoplasmosis capsulati, unspecified
B39.3	Disseminated histoplasmosis capsulati
B39.4	Histoplasmosis capsulati, unspecified
B39.5	Histoplasmosis duboisii
B39.9	Histoplasmosis, unspecified
B40	Blastomycosis
B40.0	Acute pulmonary blastomycosis
B40.1	Chronic pulmonary blastomycosis
B40.2	Pulmonary blastomycosis, unspecified
B40.3	Cutaneous blastomycosis
B40.7	Disseminated blastomycosis
B40.8	Other forms of blastomycosis
B40.9	Blastomycosis, unspecified
B42	Sporotrichosis
B42.0	Pulmonary sporotrichosis
B42.1	Lymphocutaneous sporotrichosis
B42.7	Disseminated sporotrichosis
B42.8	Other forms of sporotrichosis
B42.9	Sporotrichosis, unspecified
B43	Chromomycosis and phaeomycotic abscess
B43.0	Cutaneous chromomycosis
B43.1	Phaeomycotic brain abscess
B43.2	Subcutaneous phaeomycotic abscess and cyst
B43.8	Other forms of chromomycosis
B43.9	Chromomycosis, unspecified
B45	Cryptococcosis
B45.0	Pulmonary cryptococcosis
B45.1	Cerebral cryptococcosis
B45.2	Cutaneous cryptococcosis
B45.3	Osseous cryptococcosis
B45.7	Disseminated cryptococcosis
B45.8	Other forms of cryptococcosis
B45.9	Cryptococcosis, unspecified
B46	Zygomycosis
B46.0	Pulmonary mucormycosis
B46.1	Rhinocerebral mucormycosis
B46.2	Gastrointestinal mucormycosis
B46.3	Cutaneous mucormycosis
B46.4	Disseminated mucormycosis
B46.5	Mucormycosis, unspecified
B46.8	Other zygomycoses
B46.9	Zygomycosis, unspecified
B47.1	Actinomycetoma
B48.2	Allescheriasis
B49*	Unspecified mycosis
B50*	Plasmodium falciparum malaria

B50.0	Plasmodium falciparum malaria with cerebral complications
B50.8	Other severe and complicated Plasmodium falciparum malaria
B50.9	Plasmodium falciparum malaria, unspecified
B53.1	Malaria due to simian plasmodia
B54*	Unspecified malaria
B56*	African trypanosomiasis
B57*	Chagas disease
B58	Toxoplasmosis
B58.0	Toxoplasma oculopathy
B58.1	Toxoplasma hepatitis
B58.2	Toxoplasma meningoencephalitis
B58.3	Pulmonary toxoplasmosis
B58.8	Toxoplasmosis with other organ involvement
B58.9	Toxoplasmosis, unspecified
B60.1	Acanthamoebiasis
B60.2	Naegleriasis
B65	Schistosomiasis [bilharziasis]
B65.2	Schistosomiasis due to Schistosoma japonicum
B65.9	Schistosomiasis, unspecified
B66.4	Paragonimiasis
B67*	Echinococcosis
B68	Taeniasis
B68.0	Taenia solium taeniasis
B68.9	Taeniasis, unspecified
B69	Cysticercosis
B69.0	Cysticercosis of central nervous system
B69.1	Cysticercosis of eye
B69.8	Cysticercosis of other sites
B69.9	Cysticercosis, unspecified
B70	Diphyllobothriasis and sparganosis
B70.0	Diphyllobothriasis
B70.1	Sparganosis
B78*	Strongyloidiasis
B81.3	Intestinal angiostrongyliasis
B81.4	Mixed intestinal helminthiases
B83.0	Visceral larva migrans
B83.1	Gnathostomiasis
B83.2	Angiostrongyliasis due to Parastrongylus cantonensis
B87*	Myiasis
B96.3	Haemophilus influenzae [H. influenzae] as the cause of diseases classified to other chapters
B97.0	Adenovirus as the cause of diseases classified to other chapters
B97.1	Enterovirus as the cause of diseases classified to other chapters
B97.3	Retrovirus as the cause of diseases classified to other chapters
G00	Bacterial meningitis, not elsewhere classified
G00.0	Haemophilus meningitis
G00.1	Pneumococcal meningitis
G00.2	Streptococcal meningitis
G00.3	Staphylococcal meningitis

G00.8	Other bacterial meningitis
G00.9	Bacterial meningitis, unspecified
G01*	Meningitis in bacterial diseases classified elsewhere
G02.0	Meningitis in viral diseases classified elsewhere
G02.1	Meningitis in mycoses
G02.8	Meningitis in other specified infectious and parasitic diseases classified elsewhere
G03*	Meningitis due to other and unspecified causes
G04.1	Tropical spastic paraplegia
G04.2	Bacterial meningoencephalitis and meningomyelitis, not elsewhere classified
G05.0	Encephalitis, myelitis and encephalomyelitis in bacterial diseases classified elsewhere
G05.1	Encephalitis, myelitis and encephalomyelitis in viral diseases classified elsewhere
G05.2	Encephalitis, myelitis and encephalomyelitis in other infectious and parasitic diseases classified elsewhere
G06*	Intracranial and intraspinal abscess and granuloma
G07*	Intracranial and intraspinal abscess and granuloma in diseases classified elsewhere
H05.0	Acute inflammation of orbit
H19.0	Scleritis and episcleritis in diseases classified elsewhere
H19.1	Herpesviral keratitis and keratoconjunctivitis
H19.2	Keratitis and keratoconjunctivitis in other infectious and parasitic diseases classified elsewhere
H22.0	Iridocyclitis in infectious and parasitic diseases classified elsewhere
H32.0	Chorioretinal inflammation in infectious and parasitic diseases classified elsewhere
H60.2	Malignant otitis externa
H62.1	Otitis externa in viral diseases classified elsewhere
H62.2	Otitis externa in mycoses
H70.0	Acute mastoiditis
H75.0	Mastoiditis in infectious and parasitic diseases classified elsewhere
I33.0	Acute and subacute infective endocarditis
I68.1	Cerebral arteritis in infectious and parasitic diseases classified elsewhere
J13*	Pneumonia due to Streptococcus pneumoniae
J14*	Pneumonia due to Haemophilus influenzae
J17.2	Pneumonia in mycoses
J20.1	Acute bronchitis due to Haemophilus influenzae
K12.2	Cellulitis and abscess of mouth
K23.0	Tuberculous oesophagitis
K67.2	Syphilitic peritonitis
K67.3	Tuberculous peritonitis
M00.1	Pneumococcal arthritis and polyarthritis
M01.0	Meningococcal arthritis
M01.1	Tuberculous arthritis
M01.2	Arthritis in Lyme disease
M01.4	Rubella arthritis
M01.5	Arthritis in other viral diseases classified elsewhere
M49.0	Tuberculosis of spine
M49.1	Brucella spondylitis
M73.1	Syphilitic bursitis
N29.0	Late syphilis of kidney
N74.0	Tuberculous infection of cervix uteri
N74.1	Female tuberculous pelvic inflammatory disease
N74.2	Female syphilitic pelvic inflammatory disease

O98.0	Tuberculosis complicating pregnancy, childbirth and the puerperium
O98.1	Syphilis complicating pregnancy, childbirth and the puerperium
O98.4	Viral hepatitis complicating pregnancy, childbirth and the puerperium
O98.7	Human immunodeficiency virus [HIV] disease complicating pregnancy, childbirth and the puerperium
P23.3	Congenital pneumonia due to streptococcus, group B
P23.4	Congenital pneumonia due to Escherichia coli
P35.0	Congenital rubella syndrome
P35.1	Congenital cytomegalovirus infection
P35.2	Congenital herpesviral [herpes simplex] infection
P35.3	Congenital viral hepatitis
P36	Bacterial sepsis of newborn
P36.0	Sepsis of newborn due to streptococcus, group B
P36.4	Sepsis of newborn due to Escherichia coli
P37.0	Congenital tuberculosis
P37.1	Congenital toxoplasmosis
P37.2	Neonatal (disseminated) listeriosis
P37.3	Congenital falciparum malaria
P37.5	Neonatal candidiasis
P39.2	Intra-amniotic infection of fetus, not elsewhere classified
Z21	Asymptomatic human immunodeficiency virus [HIV] infection status

Infections not predisposed towards entering the central nervous system

A00*	Cholera
A01*	Typhoid and paratyphoid fevers
A02	Other salmonella infections
A02.0	Salmonella enteritis
A02.1	Salmonella sepsis
A02.8	Other specified salmonella infections
A02.9	Salmonella infection, unspecified
A03	Shigellosis
A03.0	Shigellosis due to Shigella dysenteriae
A03.1	Shigellosis due to Shigella flexneri
A03.2	Shigellosis due to Shigella boydii
A03.3	Shigellosis due to Shigella sonnei
A03.8	Other shigellosis
A03.9	Shigellosis, unspecified
A04	Other bacterial intestinal infections
A04.0	Enteropathogenic Escherichia coli infection
A04.1	Enterotoxigenic Escherichia coli infection
A04.2	Enteroinvasive Escherichia coli infection
A04.3	Enterohaemorrhagic Escherichia coli infection
A04.4	Other intestinal Escherichia coli infections
A04.5	Campylobacter enteritis
A04.6	Enteritis due to Yersinia enterocolitica
A04.7	Enterocolitis due to Clostridium difficile

A04.8	Other specified bacterial intestinal infections
A04.9	Bacterial intestinal infection, unspecified
A05	Other bacterial foodborne intoxications, not elsewhere classified
A05.0	Foodborne staphylococcal intoxication
A05.1	Botulism
A05.2	Foodborne Clostridium perfringens [Clostridium welchii] intoxication
A05.3	Foodborne Vibrio parahaemolyticus intoxication
A05.4	Foodborne Bacillus cereus intoxication
A05.8	Other specified bacterial foodborne intoxications
A05.9	Bacterial foodborne intoxication, unspecified
A07*	Other protozoal intestinal diseases
A08.0	Rotaviral enteritis
A08.1	Acute gastroenteropathy due to Norwalk agent
A08.3	Other viral enteritis
A08.4	Viral intestinal infection, unspecified
A08.5	Other specified intestinal infections
A09*	Other gastroenteritis and colitis of infectious and unspecified origin
A21	Tularaemia
A21.0	Ulceroglandular tularaemia
A21.1	Oculoglandular tularaemia
A21.2	Pulmonary tularaemia
A21.3	Gastrointestinal tularaemia
A21.7	Generalized tularaemia
A21.8	Other forms of tularaemia
A21.9	Tularaemia, unspecified
A24.0	Glanders
A25.0	Spirillosis
A26	Erysipeloid
A26.0	Cutaneous erysipeloid
A26.7	Erysipelothrix sepsis
A26.8	Other forms of erysipeloid
A26.9	Erysipeloid, unspecified
A28.2	Extraintestinal yersiniosis
A30*	Leprosy [Hansen disease]
A31*	Infection due to other mycobacteria
A33*	Tetanus neonatorum
A34*	Obstetrical tetanus
A35*	Other tetanus
A36*	Diphtheria
A37*	Whooping cough
A38*	Scarlet fever
A40	Streptococcal sepsis
A40.0	Sepsis due to streptococcus, group A
A40.1	Sepsis due to streptococcus, group B
A40.2	Sepsis due to streptococcus, group D
A40.8	Other streptococcal sepsis
A40.9	Streptococcal sepsis, unspecified
A41	Other sepsis

A41.0	Sepsis due to Staphylococcus aureus
A41.1	Sepsis due to other specified staphylococcus
A41.2	Sepsis due to unspecified staphylococcus
A41.4	Sepsis due to anaerobes
A41.5	Sepsis due to other Gram-negative organisms
A41.8	Other specified sepsis
A41.9	Sepsis, unspecified
A46*	Erysipelas
A48.0	Gas gangrene
A48.1	Legionnaires disease
A48.2	Nonpneumonic Legionnaires disease [Pontiac fever]
A48.3	Toxic shock syndrome
A48.4	Brazilian purpuric fever
A49	Bacterial infection of unspecified site
A49.3	Mycoplasma infection, unspecified site
A54	Gonococcal infection
A54.0	Gonococcal infection of lower genitourinary tract without periurethral or accessory gland abscess
A54.1	Gonococcal infection of lower genitourinary tract with periurethral and accessory gland abscess
A54.2	Gonococcal pelviperitonitis and other gonococcal genitourinary infections
A54.3	Gonococcal infection of eye
A54.4	Gonococcal infection of musculoskeletal system
A54.5	Gonococcal pharyngitis
A54.6	Gonococcal infection of anus and rectum
A54.8	Other gonococcal infections
A54.9	Gonococcal infection, unspecified
A55*	Chlamydial lymphogranuloma (venereum)
A56.0	Chlamydial infection of lower genitourinary tract
A56.1	Chlamydial infection of pelviperitoneum and other genitourinary organs
A56.2	Chlamydial infection of genitourinary tract, unspecified
A56.3	Chlamydial infection of anus and rectum
A56.4	Chlamydial infection of pharynx
A56.8	Sexually transmitted chlamydial infection of other sites
A57*	Chancroid
A58*	Granuloma inguinale
A59*	Trichomoniasis
A63.0	Anogenital (venereal) warts
A63.8	Other specified predominantly sexually transmitted diseases
A64*	Unspecified sexually transmitted disease
A65*	Nonvenereal syphilis
A66*	Yaws
A67*	Pinta [carate]
A69.0	Necrotizing ulcerative stomatitis
A69.1	Other Vincent infections
A71*	Trachoma
A74.0	Chlamydial conjunctivitis
A74.8	Other chlamydial diseases
A74.9	Chlamydial infection, unspecified
A79.0	Trench fever

A92.1	O'nyong-nyong fever
A93.0	Oropouche virus disease
A93.8	Other specified arthropod-borne viral fevers
A95*	Yellow fever
A98.0	Crimean-Congo haemorrhagic fever
A98.5	Haemorrhagic fever with renal syndrome
B07*	Viral warts
B08.0	Other orthopoxvirus infections
B08.1	Molluscum contagiosum
B08.3	Erythema infectiosum [fifth disease]
B08.8	Other specified viral infections characterized by skin and mucous membrane lesions
B09*	Unspecified viral infection characterized by skin and mucous membrane lesions
B30*	Viral conjunctivitis
B33.0	Epidemic myalgia
B33.1	Ross River disease
B33.2	Viral carditis
B33.4	Hantavirus (cardio-)pulmonary syndrome [HPS] [HCPS]
B34.4	Papovavirus infection, unspecified site
B35*	Dermatophytosis
B36*	Other superficial mycoses
B41	Paracoccidioidomycosis
B41.0	Pulmonary paracoccidioidomycosis
B41.7	Disseminated paracoccidioidomycosis
B41.8	Other forms of paracoccidioidomycosis
B41.9	Paracoccidioidomycosis, unspecified
B44*	Aspergillosis
B47	Mycetoma
B47.0	Eumycetoma
B47.9	Mycetoma, unspecified
B48.0	Lobomycosis
B48.1	Rhinosporidiosis
B48.3	Geotrichosis
B48.4	Penicillosis
B48.7	Opportunistic mycoses
B48.8	Other specified mycoses
B51*	Plasmodium vivax malaria
B52*	Plasmodium malariae malaria
B53	Other parasitologically confirmed malaria
B53.0	Plasmodium ovale malaria
B53.8	Other parasitologically confirmed malaria, not elsewhere classified
B55.0	Visceral leishmaniasis
B55.1	Cutaneous leishmaniasis
B55.2	Mucocutaneous leishmaniasis
B55.9	Leishmaniasis, unspecified
B59*	Pneumocystosis
B60.0	Babesiosis
B60.8	Other specified protozoal diseases
B65.0	Schistosomiasis due to Schistosoma haematobium [urinary schistosomiasis]

B65.1	Schistosomiasis due to Schistosoma mansoni [intestinal schistosomiasis]
B65.3	Cercarial dermatitis
B65.8	Other schistosomiases
B66.0	Opisthorchiasis
B66.1	Clonorchiasis
B66.2	Dicrocoeliasis
B66.3	Fascioliasis
B66.5	Fasciolopsiasis
B68.1	Taenia saginata taeniasis
B71	Other cestode infections
B71.0	Hymenolepiasis
B71.1	Dipylidiasis
B71.9	Cestode infection, unspecified
B72*	Dracunculiasis
B73*	Onchocerciasis
B74*	Filariasis
B75*	Trichinellosis
B76*	Hookworm diseases
B77*	Ascariasis
B79*	Trichuriasis
B80*	Enterobiasis
B81.0	Anisakiasis
B81.1	Intestinal capillariasis
B81.2	Trichostrongyliasis
B82	Unspecified intestinal parasitism
B82.0	Intestinal helminthiasis, unspecified
B82.9	Intestinal parasitism, unspecified
B83	Other helminthiases
B83.3	Syngamiasis
B83.4	Internal hirudiniasis
B83.8	Other specified helminthiases
B83.9	Helminthiasis, unspecified
B85*	Pediculosis and phthiriasis
B86*	Scabies
B88.0	Other acariasis
B88.1	Tungiasis [sandflea infestation]
B88.2	Other arthropod infestations
B88.3	External hirudiniasis
B88.8	Other specified infestations
B88.9	Infestation, unspecified
B89*	Unspecified parasitic disease
B95*	Streptococcus and staphylococcus as the cause of diseases classified to other chapters
B96.0	Mycoplasma pneumoniae [M. pneumoniae] as the cause of diseases classified to other chapters
B96.1	Klebsiella pneumoniae [K. pneumoniae] as the cause of diseases classified to other chapters
B96.2	Escherichia coli [E. coli] as the cause of diseases classified to other chapters
B96.4	Proteus (mirabilis)(morganii) as the cause of diseases classified to other chapters
B96.5	Pseudomonas (aeruginosa) as the cause of diseases classified to other chapters
B96.6	Bacillus fragilis [B. fragilis] as the cause of diseases classified to other chapters

B96.7	Clostridium perfringens [C. perfringens] as the cause of diseases classified to other chapters
B97.7	Papillomavirus as the cause of diseases classified to other chapters
B98.0	Helicobacter pylori [H.pylori] as the cause of diseases classified to other chapters
B98.1	Vibrio vulnificus as the cause of diseases classified to other chapters
B99*	Other and unspecified infectious diseases
C46	Kaposi sarcoma
D73.3	Abscess of spleen
E32.1	Abscess of thymus
H00*	Hordeolum and chalazion
H01.0	Blepharitis
H06.1	Parasitic infestation of orbit in diseases classified elsewhere
H10.0	Mucopurulent conjunctivitis
H10.5	Blepharoconjunctivitis
H13.0	Filarial infection of conjunctiva
H44.0	Purulent endophthalmitis
H60.0	Abscess of external ear
H60.1	Cellulitis of external ear
H60.3	Other infective otitis externa
H62.0	Otitis externa in bacterial diseases classified elsewhere
H62.3	Otitis externa in other infectious and parasitic diseases classified elsewhere
H66.0	Acute suppurative otitis media
H67.0	Otitis media in bacterial diseases classified elsewhere
H67.1	Otitis media in viral diseases classified elsewhere
I30.1	Infective pericarditis
I32.0	Pericarditis in bacterial diseases classified elsewhere
I32.1	Pericarditis in other infectious and parasitic diseases classified elsewhere
I40.0	Infective myocarditis
I41.0	Myocarditis in bacterial diseases classified elsewhere
I41.1	Myocarditis in viral diseases classified elsewhere
I41.2	Myocarditis in other infectious and parasitic diseases classified elsewhere
I43.0	Cardiomyopathy in infectious and parasitic diseases classified elsewhere
I52.0	Other heart disorders in bacterial diseases classified elsewhere
I52.1	Other heart disorders in other infectious and parasitic diseases classified elsewhere
J01.0	Acute maxillary sinusitis
J02	Acute pharyngitis
J02.0	Streptococcal pharyngitis
J02.8	Acute pharyngitis due to other specified organisms
J02.9	Acute pharyngitis, unspecified
J03	Acute tonsillitis
J03.0	Streptococcal tonsillitis
J03.8	Acute tonsillitis due to other specified organisms
J03.9	Acute tonsillitis, unspecified
J04*	Acute laryngitis and tracheitis
J05.1	Acute epiglottitis
J09*	Influenza due to certain identified influenza virus
J10*	Influenza due to other identified influenza virus
J11*	Influenza, virus not identified
J12*	Viral pneumonia, not elsewhere classified

J15	Bacterial pneumonia, not elsewhere classified
J15.0	Pneumonia due to Klebsiella pneumoniae
J15.1	Pneumonia due to Pseudomonas
J15.2	Pneumonia due to staphylococcus
J15.3	Pneumonia due to streptococcus, group B
J15.4	Pneumonia due to other streptococci
J15.5	Pneumonia due to Escherichia coli
J15.6	Pneumonia due to other aerobic Gram-negative bacteria
J15.7	Pneumonia due to Mycoplasma pneumoniae
J15.8	Other bacterial pneumonia
J15.9	Bacterial pneumonia, unspecified
J16	Pneumonia due to other infectious organisms, not elsewhere classified
J16.0	Chlamydial pneumonia
J16.8	Pneumonia due to other specified infectious organisms
J17.0	Pneumonia in bacterial diseases classified elsewhere
J17.1	Pneumonia in viral diseases classified elsewhere
J17.3	Pneumonia in parasitic diseases
J18*	Pneumonia, organism unspecified
J20	Acute bronchitis
J20.0	Acute bronchitis due to Mycoplasma pneumoniae
J20.2	Acute bronchitis due to streptococcus
J20.3	Acute bronchitis due to coxsackievirus
J20.4	Acute bronchitis due to parainfluenza virus
J20.5	Acute bronchitis due to respiratory syncytial virus
J20.6	Acute bronchitis due to rhinovirus
J20.7	Acute bronchitis due to echovirus
J20.8	Acute bronchitis due to other specified organisms
J20.9	Acute bronchitis, unspecified
J21*	Acute bronchiolitis
J21.0	Acute bronchiolitis due to respiratory syncytial virus
J21.1	Acute bronchiolitis due to human metapneumovirus
J21.8	Acute bronchiolitis due to other specified organisms
J21.9	Acute bronchiolitis, unspecified
J22*	Unspecified acute lower respiratory infection
J36*	Peritonsillar abscess
J39.0	Retropharyngeal and parapharyngeal abscess
J39.1	Other abscess of pharynx
J85.1	Abscess of lung with pneumonia
J85.2	Abscess of lung without pneumonia
J85.3	Abscess of mediastinum
J86*	Pyothorax
K02*	Dental caries
K04.0	Pulpitis
K04.4	Acute apical periodontitis of pulpal origin
K04.5	Chronic apical periodontitis
K04.6	Periapical abscess with sinus
K04.7	Periapical abscess without sinus
K05.0	Acute gingivitis

K05.2	Acute periodontitis
K05.3	Chronic periodontitis
K05.4	Periodontosis
K11.3	Abscess of salivary gland
K23.1	Megaoesophagus in Chagas disease
K35*	Acute appendicitis
K57.0	Diverticular disease of small intestine with perforation and abscess
K57.2	Diverticular disease of large intestine with perforation and abscess
K57.4	Diverticular disease of both small and large intestine with perforation and abscess
K57.8	Diverticular disease of intestine, part unspecified, with perforation and abscess
K61*	Abscess of anal and rectal regions
K63.0	Abscess of intestine
K67.0	Chlamydial peritonitis
K67.1	Gonococcal peritonitis
K67.8	Other disorders of peritoneum in infectious diseases classified elsewhere
K75.0	Abscess of liver
K75.1	Phlebitis of portal vein
K77.0	Liver disorders in infectious and parasitic diseases classified elsewhere
L00*	Staphylococcal scalded skin syndrome
L01*	Impetigo
L02*	Cutaneous abscess, furuncle and carbuncle
L03*	Cellulitis
L04*	Acute lymphadenitis
L05.0	Pilonidal cyst with abscess
L08*	Other local infections of skin and subcutaneous tissue
L70.1	Acne conglobata
M00	Pyogenic arthritis
M00.0	Staphylococcal arthritis and polyarthritis
M00.2	Other streptococcal arthritis and polyarthritis
M00.8	Arthritis and polyarthritis due to other specified bacterial agents
M00.9	Pyogenic arthritis, unspecified
M01.3	Arthritis in other bacterial diseases classified elsewhere
M01.6	Arthritis in mycoses
M01.8	Arthritis in other infectious and parasitic diseases classified elsewhere
M46.2	Osteomyelitis of vertebra
M46.3	Infection of intervertebral disc (pyogenic)
M46.5	Other infective spondylopathies
M49.2	Enterobacterial spondylitis
M49.3	Spondylopathy in other infectious and parasitic diseases classified elsewhere
M60.0	Infective myositis
M63.0	Myositis in bacterial diseases classified elsewhere
M63.1	Myositis in protozoal and parasitic infections classified elsewhere
M63.2	Myositis in other infectious diseases classified elsewhere
M65.0	Abscess of tendon sheath
M65.1	Other infective (teno)synovitis
M71.0	Abscess of bursa
M71.1	Other infective bursitis
M72.6	Necrotizing fasciitis

M73.0	Gonococcal bursitis
M86*	Osteomyelitis
M86.0	Acute haematogenous osteomyelitis
M86.1	Other acute osteomyelitis
M86.2	Subacute osteomyelitis
M86.3	Chronic multifocal osteomyelitis
M86.4	Chronic osteomyelitis with draining sinus
M86.5	Other chronic haematogenous osteomyelitis
M86.6	Other chronic osteomyelitis
M86.8	Other osteomyelitis
M86.9	Osteomyelitis, unspecified
N08.0	Glomerular disorders in infectious and parasitic diseases classified elsewhere
N10*	Acute tubulo-interstitial nephritis
N13.6	Pyonephrosis
N15.1	Renal and perinephric abscess
N16.0	Renal tubulo-interstitial disorders in infectious and parasitic diseases classified elsewhere
N29.1	Other disorders of kidney and ureter in infectious and parasitic diseases classified elsewhere
N30.0	Acute cystitis
N34.0	Urethral abscess
N39.0	Urinary tract infection, site not specified
N41.0	Acute prostatitis
N41.2	Abscess of prostate
N45*	Orchitis and epididymitis
N61*	Inflammatory disorders of breast
N70.0	Acute salpingitis and oophoritis
N71.0	Acute inflammatory disease of uterus
N73.0	Acute parametritis and pelvic cellulitis
N73.3	Female acute pelvic peritonitis
N74	Female pelvic inflammatory disorders in diseases classified elsewhere
N74.3	Female gonococcal pelvic inflammatory disease
N74.4	Female chlamydial pelvic inflammatory disease
N74.8	Female pelvic inflammatory disorders in other diseases classified elsewhere
N75.1	Abscess of Bartholin gland
N76.0	Acute vaginitis
N76.4	Abscess of vulva
N77.0	Ulceration of vulva in infectious and parasitic diseases classified elsewhere
N77.1	Vaginitis, vulvitis and vulvovaginitis in infectious and parasitic diseases classified elsewhere
O03.0	Spontaneous abortion: Incomplete, complicated by genital tract and pelvic infection
O03.5	Spontaneous abortion: Complete or unspecified, complicated by genital tract and pelvic infection
O04.0	Medical abortion: Incomplete, complicated by genital tract and pelvic infection
O04.5	Medical abortion: Complete or unspecified, complicated by genital tract and pelvic infection
O05.0	Other abortion: Incomplete, complicated by genital tract and pelvic infection
O05.5	Other abortion: Complete or unspecified, complicated by genital tract and pelvic infection
O06.0	Unspecified abortion: Incomplete, complicated by genital tract and pelvic infection
O06.5	Unspecified abortion: Complete or unspecified, complicated by genital tract and pelvic infection
O07.0	Failed medical abortion, complicated by genital tract and pelvic infection
O07.5	Other and unspecified failed attempted abortion, complicated by genital tract and pelvic infection
O08.0	Genital tract and pelvic infection following abortion and ectopic and molar pregnancy

O23.0	Infections of kidney in pregnancy
O23.1	Infections of bladder in pregnancy
O23.2	Infections of urethra in pregnancy
O23.3	Infections of other parts of urinary tract in pregnancy
O23.4	Unspecified infection of urinary tract in pregnancy
O23.5	Infections of the genital tract in pregnancy
O23.9	Other and unspecified genitourinary tract infection in pregnancy
O75.3	Other infection during labour
O85*	Puerperal sepsis
O86*	Other puerperal infections
O91.0	Infection of nipple associated with childbirth
O91.1	Abscess of breast associated with childbirth
O98	Maternal infectious and parasitic diseases classifiable elsewhere but complicating pregnancy, childbirth and the puerperium
O98.2	Gonorrhoea complicating pregnancy, childbirth and the puerperium
O98.3	Other infections with a predominantly sexual mode of transmission complicating pregnancy, childbirth and the puerperium
O98.6	Protozoal diseases complicating pregnancy, childbirth and the puerperium
O98.8	Other maternal infectious and parasitic diseases complicating pregnancy, childbirth and the puerperium
O98.9	Unspecified maternal infectious or parasitic disease complicating pregnancy, childbirth and the puerperium
P23	Congenital pneumonia
P23.0	Congenital pneumonia due to viral agent
P23.1	Congenital pneumonia due to Chlamydia
P23.2	Congenital pneumonia due to staphylococcus
P23.5	Congenital pneumonia due to Pseudomonas
P23.6	Congenital pneumonia due to other bacterial agents
P23.8	Congenital pneumonia due to other organisms
P23.9	Congenital pneumonia, unspecified
P36.1	Sepsis of newborn due to other and unspecified streptococci
P36.2	Sepsis of newborn due to Staphylococcus aureus
P36.3	Sepsis of newborn due to other and unspecified staphylococci
P36.5	Sepsis of newborn due to anaerobes
P36.8	Other bacterial sepsis of newborn
P36.9	Bacterial sepsis of newborn, unspecified
P37	Other congenital infectious and parasitic diseases
P37.4	Other congenital malaria
P37.8	Other specified congenital infectious and parasitic diseases
P37.9	Congenital infectious and parasitic disease, unspecified
P38*	Omphalitis of newborn with or without mild haemorrhage
P39	Other infections specific to the perinatal period
P39.0	Neonatal infective mastitis
P39.1	Neonatal conjunctivitis and dacryocystitis
P39.3	Neonatal urinary tract infection
P39.4	Neonatal skin infection
P39.8	Other specified infections specific to the perinatal period
P39.9	Infection specific to the perinatal period, unspecified
R57.2	Septic shock
R65.0	Systemic Inflammatory Response Syndrome of infectious origin without organ failure
R65.1	Systemic Inflammatory Response Syndrome of infectious origin with organ failure

All viral infections

A08.0	Rotaviral enteritis
A08.1	Acute gastroenteropathy due to Norwalk agent
A08.2	Adenoviral enteritis
A08.3	Other viral enteritis
A08.4	Viral intestinal infection, unspecified
A60*	Anogenital herpesviral [herpes simplex] infection
A63.0	Anogenital (venereal) warts
A80*	Acute poliomyelitis
A81.1	Subacute sclerosing panencephalitis
A81.2	Progressive multifocal leukoencephalopathy
A83*	Mosquito-borne viral encephalitis
A84*	Tick-borne viral encephalitis
A85*	Other viral encephalitis, not elsewhere classified
A86*	Unspecified viral encephalitis
A87*	Viral meningitis
A88*	Other viral infections of central nervous system, not elsewhere classified
A89*	Unspecified viral infection of central nervous system
A90*	Dengue fever [classical dengue]
A91*	Dengue haemorrhagic fever
A92	Other mosquito-borne viral fevers
A92.0	Chikungunya virus disease
A92.1	O'nyong-nyong fever
A92.2	Venezuelan equine fever
A92.3	West Nile virus infection
A92.4	Rift Valley fever
A92.8	Other specified mosquito-borne viral fevers
A92.9	Mosquito-borne viral fever, unspecified
A93	Other arthropod-borne viral fevers, not elsewhere classified
A93.0	Oropouche virus disease
A93.1	Sandfly fever
A93.2	Colorado tick fever
A93.8	Other specified arthropod-borne viral fevers
A94*	Unspecified arthropod-borne viral fever
A95*	Yellow fever
A96*	Arenaviral haemorrhagic fever
A97*	Dengue
A98	Other viral haemorrhagic fevers, not elsewhere classified
A98.0	Crimean-Congo haemorrhagic fever
A98.1	Omsk haemorrhagic fever
A98.2	Kyasanur Forest disease
A98.3	Marburg virus disease
A98.4	Ebola virus disease
A98.5	Haemorrhagic fever with renal syndrome
A98.8	Other specified viral haemorrhagic fevers

A99*	Unspecified viral haemorrhagic fever
B00	Herpesviral [herpes simplex] infections
B00.0	Eczema herpeticum
B00.1	Herpesviral vesicular dermatitis
B00.2	Herpesviral gingivostomatitis and pharyngotonsillitis
B00.3	Herpesviral meningitis
B00.4	Herpesviral encephalitis
B00.5	Herpesviral ocular disease
B00.7	Disseminated herpesviral disease
B00.8	Other forms of herpesviral infection
B00.9	Herpesviral infection, unspecified
B01	Varicella [chickenpox]
B01.0	Varicella meningitis
B01.1	Varicella encephalitis
B01.2	Varicella pneumonia
B01.8	Varicella with other complications
B01.9	Varicella without complication
B02	Zoster [herpes zoster]
B02.0	Zoster encephalitis
B02.1	Zoster meningitis
B02.2	Zoster with other nervous system involvement
B02.3	Zoster ocular disease
B02.7	Disseminated zoster
B02.8	Zoster with other complications
B02.9	Zoster without complication
B04*	Monkeypox
B05	Measles
B05.0	Measles complicated by encephalitis
B05.1	Measles complicated by meningitis
B05.2	Measles complicated by pneumonia
B05.3	Measles complicated by otitis media
B05.4	Measles with intestinal complications
B05.8	Measles with other complications
B05.9	Measles without complication
B06	Rubella [German measles]
B06.0	Rubella with neurological complications
B06.8	Rubella with other complications
B06.9	Rubella without complication
B07*	Viral warts
B08.0	Other orthopoxvirus infections
B08.1	Molluscum contagiosum
B08.2	Exanthema subitum [sixth disease]
B08.3	Erythema infectiosum [fifth disease]
B08.4	Enteroviral vesicular stomatitis with exanthem
B08.5	Enteroviral vesicular pharyngitis
B08.8	Other specified viral infections characterized by skin and mucous membrane lesions
B09*	Unspecified viral infection characterized by skin and mucous membrane lesions
B15	Acute hepatitis A

B15.0	Hepatitis A with hepatic coma
B15.9	Hepatitis A without hepatic coma
B16	Acute hepatitis B
B16.0	Acute hepatitis B with delta-agent (coinfection) with hepatic coma
B16.1	Acute hepatitis B with delta-agent (coinfection) without hepatic coma
B16.2	Acute hepatitis B without delta-agent with hepatic coma
B16.9	Acute hepatitis B without delta-agent and without hepatic coma
B17*	Other acute viral hepatitis
B18*	Chronic viral hepatitis
B19	Unspecified viral hepatitis
B19.0	Unspecified viral hepatitis with hepatic coma
B19.9	Unspecified viral hepatitis without hepatic coma
B20*	Human immunodeficiency virus [HIV] disease resulting in infectious and parasitic diseases
B21*	Human immunodeficiency virus [HIV] disease resulting in malignant neoplasms
B21.0	HIV disease resulting in Kaposi sarcoma
B22	Human immunodeficiency virus [HIV] disease resulting in other specified diseases
B22.0	HIV disease resulting in encephalopathy
B22.1	HIV disease resulting in lymphoid interstitial pneumonitis
B22.2	HIV disease resulting in wasting syndrome
B22.7	HIV disease resulting in multiple diseases classified elsewhere
B23*	Human immunodeficiency virus [HIV] disease resulting in other conditions
B24*	Unspecified human immunodeficiency virus [HIV] disease
B25*	Cytomegaloviral disease
B26	Mumps
B26.0	Mumps orchitis
B26.1	Mumps meningitis
B26.2	Mumps encephalitis
B26.3	Mumps pancreatitis
B26.8	Mumps with other complications
B26.9	Mumps without complication
B27	Infectious mononucleosis
B27.0	Gammaherpesviral mononucleosis
B27.1	Cytomegaloviral mononucleosis
B27.9	Infectious mononucleosis, unspecified
B30*	Viral conjunctivitis
B33	Other viral diseases, not elsewhere classified
B33.0	Epidemic myalgia
B33.1	Ross River disease
B33.2	Viral carditis
B33.3	Retrovirus infections, not elsewhere classified
B33.4	Hantavirus (cardio-)pulmonary syndrome [HPS] [HCPS]
B33.8	Other specified viral diseases
B34	Viral infection of unspecified site
B34.0	Adenovirus infection, unspecified site
B34.1	Enterovirus infection, unspecified site
B34.2	Coronavirus infection, unspecified site
B34.3	Parvovirus infection, unspecified site
B34.4	Papovavirus infection, unspecified site

B34.8	Other viral infections of unspecified site
B34.9	Viral infection, unspecified
B97	Viral agents as the cause of diseases classified to other chapters
B97.0	Adenovirus as the cause of diseases classified to other chapters
B97.1	Enterovirus as the cause of diseases classified to other chapters
B97.2	Coronavirus as the cause of diseases classified to other chapters
B97.3	Retrovirus as the cause of diseases classified to other chapters
B97.4	Respiratory syncytial virus as the cause of diseases classified to other chapters
B97.5	Reovirus as the cause of diseases classified to other chapters
B97.6	Parvovirus as the cause of diseases classified to other chapters
B97.7	Papillomavirus as the cause of diseases classified to other chapters
B97.8	Other viral agents as the cause of diseases classified to other chapters
C46	Kaposi sarcoma
G02.0	Meningitis in viral diseases classified elsewhere
G04.1	Tropical spastic paraplegia
G05.1	Encephalitis, myelitis and encephalomyelitis in viral diseases classified elsewhere
H19.1	Herpesviral keratitis and keratoconjunctivitis
H62.1	Otitis externa in viral diseases classified elsewhere
H67.1	Otitis media in viral diseases classified elsewhere
I41.1	Myocarditis in viral diseases classified elsewhere
J09*	Influenza due to certain identified influenza virus
J10*	Influenza due to other identified influenza virus
J11*	Influenza, virus not identified
J12*	Viral pneumonia, not elsewhere classified
J17.1	Pneumonia in viral diseases classified elsewhere
J20.3	Acute bronchitis due to coxsackievirus
J20.4	Acute bronchitis due to parainfluenza virus
J20.5	Acute bronchitis due to respiratory syncytial virus
J20.6	Acute bronchitis due to rhinovirus
J20.7	Acute bronchitis due to echovirus
J21*	Acute bronchiolitis
J21.0	Acute bronchiolitis due to respiratory syncytial virus
J21.1	Acute bronchiolitis due to human metapneumovirus
J21.8	Acute bronchiolitis due to other specified organisms
J21.9	Acute bronchiolitis, unspecified
M01.4	Rubella arthritis
M01.5	Arthritis in other viral diseases classified elsewhere
O98.4	Viral hepatitis complicating pregnancy, childbirth and the puerperium
O98.5	Other viral diseases complicating pregnancy, childbirth and the puerperium
O98.7	Human immunodeficiency virus [HIV] disease complicating pregnancy, childbirth and the puerperium
P23.0	Congenital pneumonia due to viral agent
P35.0	Congenital rubella syndrome
P35.1	Congenital cytomegalovirus infection
P35.2	Congenital herpesviral [herpes simplex] infection
P35.3	Congenital viral hepatitis
Z21	Asymptomatic human immunodeficiency virus [HIV] infection status

Acute viral infections

A08.0	Rotaviral enteritis
A08.1	Acute gastroenteropathy due to Norwalk agent
A08.2	Adenoviral enteritis
A08.3	Other viral enteritis
A08.4	Viral intestinal infection, unspecified
A80*	Acute poliomyelitis
A83*	Mosquito-borne viral encephalitis
A84*	Tick-borne viral encephalitis
A85*	Other viral encephalitis, not elsewhere classified
A86*	Unspecified viral encephalitis
A87*	Viral meningitis
A88*	Other viral infections of central nervous system, not elsewhere classified
A89*	Unspecified viral infection of central nervous system
A90*	Dengue fever [classical dengue]
A91*	Dengue haemorrhagic fever
A92	Other mosquito-borne viral fevers
A92.0	Chikungunya virus disease
A92.1	O'nyong-nyong fever
A92.2	Venezuelan equine fever
A92.3	West Nile virus infection
A92.4	Rift Valley fever
A92.8	Other specified mosquito-borne viral fevers
A92.9	Mosquito-borne viral fever, unspecified
A93	Other arthropod-borne viral fevers, not elsewhere classified
A93.0	Oropouche virus disease
A93.1	Sandfly fever
A93.2	Colorado tick fever
A93.8	Other specified arthropod-borne viral fevers
A94*	Unspecified arthropod-borne viral fever
A95*	Yellow fever
A96*	Arenaviral haemorrhagic fever
A97*	Dengue
A98	Other viral haemorrhagic fevers, not elsewhere classified
A98.0	Crimean-Congo haemorrhagic fever
A98.1	Omsk haemorrhagic fever
A98.2	Kyasanur Forest disease
A98.3	Marburg virus disease
A98.4	Ebola virus disease
A98.5	Haemorrhagic fever with renal syndrome
A98.8	Other specified viral haemorrhagic fevers
A99*	Unspecified viral haemorrhagic fever
B04*	Monkeypox
B05	Measles
B05.0	Measles complicated by encephalitis

B05.1	Measles complicated by meningitis
B05.2	Measles complicated by pneumonia
B05.3	Measles complicated by otitis media
B05.4	Measles with intestinal complications
B05.8	Measles with other complications
B05.9	Measles without complication
B06	Rubella [German measles]
B06.0	Rubella with neurological complications
B06.8	Rubella with other complications
B06.9	Rubella without complication
B07*	Viral warts
B08.0	Other orthopoxvirus infections
B08.1	Molluscum contagiosum
B08.3	Erythema infectiosum [fifth disease]
B08.4	Enteroviral vesicular stomatitis with exanthem
B08.5	Enteroviral vesicular pharyngitis
B08.8	Other specified viral infections characterized by skin and mucous membrane lesions
B09*	Unspecified viral infection characterized by skin and mucous membrane lesions
B15	Acute hepatitis A
B15.0	Hepatitis A with hepatic coma
B15.9	Hepatitis A without hepatic coma
B16	Acute hepatitis B
B16.0	Acute hepatitis B with delta-agent (coinfection) with hepatic coma
B16.1	Acute hepatitis B with delta-agent (coinfection) without hepatic coma
B16.2	Acute hepatitis B without delta-agent with hepatic coma
B16.9	Acute hepatitis B without delta-agent and without hepatic coma
B17*	Other acute viral hepatitis
B19	Unspecified viral hepatitis
B19.0	Unspecified viral hepatitis with hepatic coma
B19.9	Unspecified viral hepatitis without hepatic coma
B26	Mumps
B26.0	Mumps orchitis
B26.1	Mumps meningitis
B26.2	Mumps encephalitis
B26.3	Mumps pancreatitis
B26.8	Mumps with other complications
B26.9	Mumps without complication
B30*	Viral conjunctivitis
B33	Other viral diseases, not elsewhere classified
B33.0	Epidemic myalgia
B33.1	Ross River disease
B33.2	Viral carditis
B33.4	Hantavirus (cardio-)pulmonary syndrome [HPS] [HCPS]
B33.8	Other specified viral diseases
B34	Viral infection of unspecified site
B34.0	Adenovirus infection, unspecified site
B34.1	Enterovirus infection, unspecified site
B34.2	Coronavirus infection, unspecified site

B34.3	Parvovirus infection, unspecified site
B34.8	Other viral infections of unspecified site
B34.9	Viral infection, unspecified
B97	Viral agents as the cause of diseases classified to other chapters
B97.0	Adenovirus as the cause of diseases classified to other chapters
B97.1	Enterovirus as the cause of diseases classified to other chapters
B97.2	Coronavirus as the cause of diseases classified to other chapters
B97.4	Respiratory syncytial virus as the cause of diseases classified to other chapters
B97.5	Reovirus as the cause of diseases classified to other chapters
B97.6	Parvovirus as the cause of diseases classified to other chapters
B97.8	Other viral agents as the cause of diseases classified to other chapters
H67.1	Otitis media in viral diseases classified elsewhere
I41.1	Myocarditis in viral diseases classified elsewhere
J09*	Influenza due to certain identified influenza virus
J10*	Influenza due to other identified influenza virus
J11*	Influenza, virus not identified
J12*	Viral pneumonia, not elsewhere classified
J17.1	Pneumonia in viral diseases classified elsewhere
J20.3	Acute bronchitis due to coxsackievirus
J20.4	Acute bronchitis due to parainfluenza virus
J20.5	Acute bronchitis due to respiratory syncytial virus
J20.6	Acute bronchitis due to rhinovirus
J20.7	Acute bronchitis due to echovirus
J21*	Acute bronchiolitis
J21.0	Acute bronchiolitis due to respiratory syncytial virus
J21.1	Acute bronchiolitis due to human metapneumovirus
J21.8	Acute bronchiolitis due to other specified organisms
J21.9	Acute bronchiolitis, unspecified
M01.4	Rubella arthritis
M01.5	Arthritis in other viral diseases classified elsewhere
O98.5	Other viral diseases complicating pregnancy, childbirth and the puerperium
P23.0	Congenital pneumonia due to viral agent
P35.0	Congenital rubella syndrome

Herpesvirus infections

A60*	Anogenital herpesviral [herpes simplex] infection
B00	Herpesviral [herpes simplex] infections
B00.0	Eczema herpeticum
B00.1	Herpesviral vesicular dermatitis
B00.2	Herpesviral gingivostomatitis and pharyngotonsillitis
B00.3	Herpesviral meningitis
B00.4	Herpesviral encephalitis
B00.5	Herpesviral ocular disease
B00.7	Disseminated herpesviral disease
B00.8	Other forms of herpesviral infection

B00.9	Herpesviral infection, unspecified
B01	Varicella [chickenpox]
B01.0	Varicella meningitis
B01.1	Varicella encephalitis
B01.2	Varicella pneumonia
B01.8	Varicella with other complications
B01.9	Varicella without complication
B02	Zoster [herpes zoster]
B02.0	Zoster encephalitis
B02.1	Zoster meningitis
B02.2	Zoster with other nervous system involvement
B02.3	Zoster ocular disease
B02.7	Disseminated zoster
B02.8	Zoster with other complications
B02.9	Zoster without complication
B08.2	Exanthema subitum [sixth disease]
B21.0	HIV disease resulting in Kaposi sarcoma
B25*	Cytomegaloviral disease
B27	Infectious mononucleosis
B27.0	Gammaherpesviral mononucleosis
B27.1	Cytomegaloviral mononucleosis
B27.9	Infectious mononucleosis, unspecified
C46	Kaposi sarcoma
H19.1	Herpesviral keratitis and keratoconjunctivitis
P35.1	Congenital cytomegalovirus infection
P35.2	Congenital herpesviral [herpes simplex] infection

Other potentially persistent viral infections

A63.0	Anogenital (venereal) warts
A81.1	Subacute sclerosing panencephalitis
A81.2	Progressive multifocal leukoencephalopathy
B18*	Chronic viral hepatitis
B20*	Human immunodeficiency virus [HIV] disease resulting in infectious and parasitic diseases
B21*	Human immunodeficiency virus [HIV] disease resulting in malignant neoplasms
B22	Human immunodeficiency virus [HIV] disease resulting in other specified diseases
B22.0	HIV disease resulting in encephalopathy
B22.1	HIV disease resulting in lymphoid interstitial pneumonitis
B22.2	HIV disease resulting in wasting syndrome
B22.7	HIV disease resulting in multiple diseases classified elsewhere
B23*	Human immunodeficiency virus [HIV] disease resulting in other conditions
B24*	Unspecified human immunodeficiency virus [HIV] disease
B33.3	Retrovirus infections, not elsewhere classified
B34.4	Papovavirus infection, unspecified site
B97.3	Retrovirus as the cause of diseases classified to other chapters
B97.7	Papillomavirus as the cause of diseases classified to other chapters

G04.1	Tropical spastic paraplegia
O98.4	Viral hepatitis complicating pregnancy, childbirth and the puerperium
O98.7	Human immunodeficiency virus [HIV] disease complicating pregnancy, childbirth and the puerperium
P35.3	Congenital viral hepatitis
Z21	Asymptomatic human immunodeficiency virus [HIV] infection status

Parasitic infections

A06	Amoebiasis
A06.0	Acute amoebic dysentery
A06.1	Chronic intestinal amoebiasis
A06.2	Amoebic nondysenteric colitis
A06.3	Amoeboma of intestine
A06.4	Amoebic liver abscess
A06.5	Amoebic lung abscess
A06.6	Amoebic brain abscess
A06.7	Cutaneous amoebiasis
A06.8	Amoebic infection of other sites
A06.9	Amoebiasis, unspecified
A07*	Other protozoal intestinal diseases
A59*	Trichomoniasis
B50*	Plasmodium falciparum malaria
B50.0	Plasmodium falciparum malaria with cerebral complications
B50.8	Other severe and complicated Plasmodium falciparum malaria
B50.9	Plasmodium falciparum malaria, unspecified
B51*	Plasmodium vivax malaria
B52*	Plasmodium malariae malaria
B53	Other parasitologically confirmed malaria
B53.0	Plasmodium ovale malaria
B53.1	Malaria due to simian plasmodia
B53.8	Other parasitologically confirmed malaria, not elsewhere classified
B54*	Unspecified malaria
B55.0	Visceral leishmaniasis
B55.1	Cutaneous leishmaniasis
B55.2	Mucocutaneous leishmaniasis
B55.9	Leishmaniasis, unspecified
B56*	African trypanosomiasis
B57*	Chagas disease
B58	Toxoplasmosis
B58.0	Toxoplasma oculopathy
B58.1	Toxoplasma hepatitis
B58.2	Toxoplasma meningoencephalitis
B58.3	Pulmonary toxoplasmosis
B58.8	Toxoplasmosis with other organ involvement
B58.9	Toxoplasmosis, unspecified
B60.0	Babesiosis

B60.1	Acanthamoebiasis
B60.2	Naegleriasis
B60.8	Other specified protozoal diseases
B64*	Unspecified protozoal disease
B65	Schistosomiasis [bilharziasis]
B65.0	Schistosomiasis due to Schistosoma haematobium [urinary schistosomiasis]
B65.1	Schistosomiasis due to Schistosoma mansoni [intestinal schistosomiasis]
B65.2	Schistosomiasis due to Schistosoma japonicum
B65.3	Cercarial dermatitis
B65.8	Other schistosomiases
B65.9	Schistosomiasis, unspecified
B66	Other fluke infections
B66.0	Opisthorchiasis
B66.1	Clonorchiasis
B66.2	Dicrocoeliasis
B66.3	Fascioliasis
B66.4	Paragonimiasis
B66.5	Fasciolopsiasis
B66.8	Other specified fluke infections
B66.9	Fluke infection, unspecified
B67*	Echinococcosis
B68	Taeniasis
B68.0	Taenia solium taeniasis
B68.1	Taenia saginata taeniasis
B68.9	Taeniasis, unspecified
B69	Cysticercosis
B69.0	Cysticercosis of central nervous system
B69.1	Cysticercosis of eye
B69.8	Cysticercosis of other sites
B69.9	Cysticercosis, unspecified
B70	Diphyllobothriasis and sparganosis
B70.0	Diphyllobothriasis
B70.1	Sparganosis
B71	Other cestode infections
B71.0	Hymenolepiasis
B71.1	Dipylidiasis
B71.8	Other specified cestode infections
B71.9	Cestode infection, unspecified
B72*	Dracunculiasis
B73*	Onchocerciasis
B74*	Filariasis
B75*	Trichinellosis
B76*	Hookworm diseases
B77*	Ascariasis
B78*	Strongyloidiasis
B79*	Trichuriasis
B80*	Enterobiasis
B81	Other intestinal helminthiases, not elsewhere classified

B81.0	Anisakiasis
B81.1	Intestinal capillariasis
B81.2	Trichostrongyliasis
B81.3	Intestinal angiostrongyliasis
B81.4	Mixed intestinal helminthiases
B81.8	Other specified intestinal helminthiases
B82	Unspecified intestinal parasitism
B82.0	Intestinal helminthiasis, unspecified
B82.9	Intestinal parasitism, unspecified
B83	Other helminthiases
B83.0	Visceral larva migrans
B83.1	Gnathostomiasis
B83.2	Angiostrongyliasis due to Parastrongylus cantonensis
B83.3	Syngamiasis
B83.4	Internal hirudiniasis
B83.8	Other specified helminthiases
B83.9	Helminthiasis, unspecified
B85*	Pediculosis and phthiriasis
B86*	Scabies
B87*	Myiasis
B88	Other infestations
B88.0	Other acariasis
B88.1	Tungiasis [sandflea infestation]
B88.2	Other arthropod infestations
B88.3	External hirudiniasis
B88.8	Other specified infestations
B88.9	Infestation, unspecified
B89*	Unspecified parasitic disease
H06.1	Parasitic infestation of orbit in diseases classified elsewhere
H13.0	Filarial infection of conjunctiva
J17.3	Pneumonia in parasitic diseases
K23.1	Megaoesophagus in Chagas disease
M63.1	Myositis in protozoal and parasitic infections classified elsewhere
O98.6	Protozoal diseases complicating pregnancy, childbirth and the puerperium
P37.1	Congenital toxoplasmosis
P37.3	Congenital falciparum malaria
P37.4	Other congenital malaria

Fungal infections (mycoses)

B35*	Dermatophytosis
B36*	Other superficial mycoses
B37	Candidiasis
B37.0	Candidal stomatitis
B37.1	Pulmonary candidiasis
B37.2	Candidiasis of skin and nail

B37.3	Candidiasis of vulva and vagina
B37.4	Candidiasis of other urogenital sites
B37.5	Candidal meningitis
B37.6	Candidal endocarditis
B37.7	Candidal sepsis
B37.8	Candidiasis of other sites
B37.9	Candidiasis, unspecified
B38	Coccidioidomycosis
B38.0	Acute pulmonary coccidioidomycosis
B38.1	Chronic pulmonary coccidioidomycosis
B38.2	Pulmonary coccidioidomycosis, unspecified
B38.3	Cutaneous coccidioidomycosis
B38.4	Coccidioidomycosis meningitis
B38.7	Disseminated coccidioidomycosis
B38.8	Other forms of coccidioidomycosis
B38.9	Coccidioidomycosis, unspecified
B39	Histoplasmosis
B39.0	Acute pulmonary histoplasmosis capsulati
B39.1	Chronic pulmonary histoplasmosis capsulati
B39.2	Pulmonary histoplasmosis capsulati, unspecified
B39.3	Disseminated histoplasmosis capsulati
B39.4	Histoplasmosis capsulati, unspecified
B39.5	Histoplasmosis duboisii
B39.9	Histoplasmosis, unspecified
B40	Blastomycosis
B40.0	Acute pulmonary blastomycosis
B40.1	Chronic pulmonary blastomycosis
B40.2	Pulmonary blastomycosis, unspecified
B40.3	Cutaneous blastomycosis
B40.7	Disseminated blastomycosis
B40.8	Other forms of blastomycosis
B40.9	Blastomycosis, unspecified
B41	Paracoccidioidomycosis
B41.0	Pulmonary paracoccidioidomycosis
B41.7	Disseminated paracoccidioidomycosis
B41.8	Other forms of paracoccidioidomycosis
B41.9	Paracoccidioidomycosis, unspecified
B42	Sporotrichosis
B42.0	Pulmonary sporotrichosis
B42.1	Lymphocutaneous sporotrichosis
B42.7	Disseminated sporotrichosis
B42.8	Other forms of sporotrichosis
B42.9	Sporotrichosis, unspecified
B43	Chromomycosis and phaeomycotic abscess
B43.0	Cutaneous chromomycosis
B43.1	Phaeomycotic brain abscess
B43.2	Subcutaneous phaeomycotic abscess and cyst
B43.8	Other forms of chromomycosis

B43.9	Chromomycosis, unspecified
B44*	Aspergillosis
B45	Cryptococcosis
B45.0	Pulmonary cryptococcosis
B45.1	Cerebral cryptococcosis
B45.2	Cutaneous cryptococcosis
B45.3	Osseous cryptococcosis
B45.7	Disseminated cryptococcosis
B45.8	Other forms of cryptococcosis
B45.9	Cryptococcosis, unspecified
B46	Zygomycosis
B46.0	Pulmonary mucormycosis
B46.1	Rhinocerebral mucormycosis
B46.2	Gastrointestinal mucormycosis
B46.3	Cutaneous mucormycosis
B46.4	Disseminated mucormycosis
B46.5	Mucormycosis, unspecified
B46.8	Other zygomycoses
B46.9	Zygomycosis, unspecified
B47.0	Eumycetoma
B48	Other mycoses, not elsewhere classified
B48.0	Lobomycosis
B48.1	Rhinosporidiosis
B48.2	Allescheriasis
B48.3	Geotrichosis
B48.4	Penicillosis
B48.7	Opportunistic mycoses
B48.8	Other specified mycoses
B49*	Unspecified mycosis
B59*	Pneumocystosis
G02.1	Meningitis in mycoses
H62.2	Otitis externa in mycoses
J17.2	Pneumonia in mycoses
M01.6	Arthritis in mycoses
P37.5	Neonatal candidiasis

* Includes the underlying 4-digit codes

Abbreviations: ICD-10, International Classification of Diseases, 10th Revision.
ICD-10 disease names are from: World Health Organization. International Statistical Classification of Diseases and Related Health Problems 10th Revision [Internet]. ICD-10 Version:2016. [cited 2 Oct 2018]. Available from: https://icd.who.int/browse10/2016/en

eTable 6. ICD-10 codes for infectious diseases with the corresponding ICD-8 and ICD-9 codes

ICD-10 code	ICD-10 decription	ICD-9 code	ICD-8 code
A00	Cholera	0010A	00001
A00	Cholera	0011A	00010
A00	Cholera	0019X	00097
A00	Cholera		00099
A01*	Typhoid and paratyphoid fevers		
A01.0	Typhoid fever	0020A	00197
A01.0	Typhoid fever		00199
A01.1	Paratyphoid fever A	0021A	00200
A01.2	Paratyphoid fever B	0022A	00210
A01.3	Paratyphoid fever C	0023A	00220
A01.4	Paratyphoid fever, unspecified	0029X	0029
A02	Other salmonella infections	003	003
A02.0	Salmonella enteritis	0030A	
A02.1	Salmonella sepsis	0031A	
G01*A02.2	Localized salmonella infections	0032A	
A02.2	Localized salmonella infections	0032B	
A02.2	Localized salmonella infections	0032C	
A02.2	Localized salmonella infections	0032D	
A02.8	Other specified salmonella infections	0038X	
A02.9	Salmonella infection, unspecified	0039X	
A03	Shigellosis	004	004
A03.0	Shigellosis due to Shigella dysenteriae	0040A	00400
A03.0	Shigellosis due to Shigella dysenteriae		00440
A03.1	Shigellosis due to Shigella flexneri	0041A	00410
A03.2	Shigellosis due to Shigella boydii	0042A	00420
A03.3	Shigellosis due to Shigella sonnei	0043A	00430
A03.8	Other shigellosis	0048X	00480
A03.9	Shigellosis, unspecified	0049X	00499
A04	Other bacterial intestinal infections		
A04.0	Enteropathogenic Escherichia coli infection		
A04.1	Enterotoxigenic Escherichia coli infection	0080A	
A04.2	Enteroinvasive Escherichia coli infection	0080B	
A04.3	Enterohaemorrhagic Escherichia coli infection		
A04.4	Other intestinal Escherichia coli infections		00800
A04.5	Campylobacter enteritis	0084E	
A04.6	Enteritis due to Yersinia enterocolitica	0084C	
A04.6	Enteritis due to Yersinia enterocolitica	0084D	
A04.7	Enterocolitis due to Clostridium difficile	0084F	
A04.8	Other specified bacterial intestinal infections	0081A	00810
A04.8	Other specified bacterial intestinal infections	0082A	00820
A04.8	Other specified bacterial intestinal infections	0083A	
A04.8	Other specified bacterial intestinal infections	0084A	
A04.8	Other specified bacterial intestinal infections	0084B	
A04.9	Bacterial intestinal infection, unspecified	0085X	00830
A05	Other bacterial foodborne intoxications, not elsewhere classified		

A05.0	Foodborne staphylococcal intoxication	0050A	00500
A05.1	Botulism	0051A	00510
A05.2	Foodborne Clostridium perfringens [Clostridium welchii] intoxication	0052A	
A05.3	Foodborne Vibrio parahaemolyticus intoxication	0054A	
A05.4	Foodborne Bacillus cereus intoxication		
A05.8	Other specified bacterial foodborne intoxications	0058X	00580
A05.9	Bacterial foodborne intoxication, unspecified	0053A	00520
A05.9	Bacterial foodborne intoxication, unspecified	0059X	00599
A05.9	Bacterial foodborne intoxication, unspecified		00597
A06	Amoebiasis	006	
A06.0	Acute amoebic dysentery	0060A	
A06.1	Chronic intestinal amoebiasis	0061A	
A06.2	Amoebic nondysenteric colitis	0062A	
A06.3	Amoeboma of intestine		
A06.4	Amoebic liver abscess	0063A	00600
A06.5	Amoebic lung abscess		
A06.6	Amoebic brain abscess		
A06.7	Cutaneous amoebiasis		
A06.8	Amoebic infection of other sites	0068X	
A06.9	Amoebiasis, unspecified	0069X	00690
A06.9	Amoebiasis, unspecified		00697
A06.9	Amoebiasis, unspecified		00699
A07	Other protozoal intestinal diseases	007	007
A07.8	Other specified protozoal intestinal diseases	1365A	
A08.0	Rotaviral enteritis	0086A	
A08.1	Acute gastroenteropathy due to Norwalk agent	0086B	
A08.2	Adenoviral enteritis	0086C	
A08.3	Other viral enteritis		00880
A08.4	Viral intestinal infection, unspecified	0086X	00898
A08.5	Other specified intestinal infections	0088X	
A09	Other gastroenteritis and colitis of infectious and unspecified origin	009	009
A15*	Respiratory tuberculosis, bacteriologically and histologically confirmed		
A15.0	Tuberculosis of lung, confirmed by sputum microscopy with or without culture	0110A	
A15.0	Tuberculosis of lung, confirmed by sputum microscopy with or without culture	0112A	
A15.0	Tuberculosis of lung, confirmed by sputum microscopy with or without culture	0113A	
A15.0	Tuberculosis of lung, confirmed by sputum microscopy with or without culture	0116A	
A15.0	Tuberculosis of lung, confirmed by sputum microscopy with or without culture	0118A	
A15.0	Tuberculosis of lung, confirmed by sputum microscopy with or without culture	0119A	
A15.2	Tuberculosis of lung, confirmed histologically	0110B	
A15.2	Tuberculosis of lung, confirmed histologically	0112B	
A15.2	Tuberculosis of lung, confirmed histologically	0113B	
A15.2	Tuberculosis of lung, confirmed histologically	0116B	
A15.2	Tuberculosis of lung, confirmed histologically	0118B	
A15.2	Tuberculosis of lung, confirmed histologically	0119B	
A15.4	Tuberculosis of intrathoracic lymph nodes, confirmed bacteriologically and histologically	0121A	
A15.4	Tuberculosis of intrathoracic lymph nodes, confirmed bacteriologically and histologically	0121B	
A15.5	Tuberculosis of larynx, trachea and bronchus, confirmed bacteriologically and histologically	0122A	
A15.5	Tuberculosis of larynx, trachea and bronchus, confirmed bacteriologically and histologically	0122B	
A15.5	Tuberculosis of larynx, trachea and bronchus, confirmed bacteriologically and histologically	0123A	
A15.5	Tuberculosis of larynx, trachea and bronchus, confirmed bacteriologically and histologically	0123B	

A15.6	Tuberculous pleurisy, confirmed bacteriologically and histologically	0120A	
A15.6	Tuberculous pleurisy, confirmed bacteriologically and histologically	0120B	
A15.7	Primary respiratory tuberculosis, confirmed bacteriologically and histologically	0101A	
A15.7	Primary respiratory tuberculosis, confirmed bacteriologically and histologically	0101B	
A15.8	Other respiratory tuberculosis, confirmed bacteriologically and histologically	0128A	
A15.8	Other respiratory tuberculosis, confirmed bacteriologically and histologically	0128B	
A16*	Respiratory tuberculosis, not confirmed bacteriologically or histologically		
A16.2	Tuberculosis of lung, without mention of bacteriological or histological confirmation	0110X	01099
A16.2	Tuberculosis of lung, without mention of bacteriological or histological confirmation	0112X	01101
A16.2	Tuberculosis of lung, without mention of bacteriological or histological confirmation	0113X	01102
A16.2	Tuberculosis of lung, without mention of bacteriological or histological confirmation	0116X	01103
A16.2	Tuberculosis of lung, without mention of bacteriological or histological confirmation	0118X	01104
A16.2	Tuberculosis of lung, without mention of bacteriological or histological confirmation	0119X	01105
A16.2	Tuberculosis of lung, without mention of bacteriological or histological confirmation		01106
A16.2	Tuberculosis of lung, without mention of bacteriological or histological confirmation		01107
A16.3	Tuberculosis of intrathoracic lymph nodes, without mention of bacteriological or histological confirmation	0121X	01292
A16.4	Tuberculosis of larynx, trachea and bronchus, without mention of bacteriological or histological confirmation	0122X	01230
A16.4	Tuberculosis of larynx, trachea and bronchus, without mention of bacteriological or histological confirmation	0123X	01290
A16.5	Tuberculous pleurisy, without mention of bacteriological or histological confirmation	0120X	01210
A16.5	Tuberculous pleurisy, without mention of bacteriological or histological confirmation		01211
A16.7	Primary respiratory tuberculosis without mention of bacteriological or histological confirmation	0100	01200
A16.7	Primary respiratory tuberculosis without mention of bacteriological or histological confirmation	0101X	
A16.8	Other respiratory tuberculosis, without mention of bacteriological or histological confirmation	0128X	01291
A16.8	Other respiratory tuberculosis, without mention of bacteriological or histological confirmation		01296
A16.9	Respiratory tuberculosis unspecified, without mention of bacteriological or histological confirmation	0108A	
A16.9	Respiratory tuberculosis unspecified, without mention of bacteriological or histological confirmation	0108B	
A16.9	Respiratory tuberculosis unspecified, without mention of bacteriological or histological confirmation	0108X	01212
A16.9	Respiratory tuberculosis unspecified, without mention of bacteriological or histological confirmation	0109A	
A16.9	Respiratory tuberculosis unspecified, without mention of bacteriological or histological confirmation	0109B	
A16.9	Respiratory tuberculosis unspecified, without mention of bacteriological or histological confirmation	0109X	01297
A16.9	Respiratory tuberculosis unspecified, without mention of bacteriological or histological confirmation		01109
A16.9	Respiratory tuberculosis unspecified, without mention of bacteriological or histological confirmation		01797
A17	Tuberculosis of nervous system		
A17.0	Tuberculous meningitis	0130	01300
A17.1	Meningeal tuberculoma	0131	
A17.8	Other tuberculosis of nervous system	0132	01390
A17.8	Other tuberculosis of nervous system	0133	
A17.8	Other tuberculosis of nervous system	0138	
A17.9	Tuberculosis of nervous system, unspecified	0139	01397
A17.9	Tuberculosis of nervous system, unspecified		01399
A18*	Tuberculosis of other organs		
A18.0	Tuberculosis of bones and joints	015	015
A18.1	Tuberculosis of genitourinary system	016	016
A18.2	Tuberculous peripheral lymphadenopathy	0172A	01710
A18.2	Tuberculous peripheral lymphadenopathy		01718
A18.2	Tuberculous peripheral lymphadenopathy		01719
A18.3	Tuberculosis of intestines, peritoneum and mesenteric glands	014	014
A18.4	Tuberculosis of skin and subcutaneous tissue	0170	0170
A18.4	Tuberculosis of skin and subcutaneous tissue	0171A	

A18.4	Tuberculosis of skin and subcutaneous tissue	0171B	
A18.4	Tuberculosis of skin and subcutaneous tissue	0171C	
A18.4	Tuberculosis of skin and subcutaneous tissue	0171D	
A18.5	Tuberculosis of eye	0173A	01720
A18.5	Tuberculosis of eye		01721
A18.5	Tuberculosis of eye		01722
A18.5	Tuberculosis of eye		01723
A18.5	Tuberculosis of eye		01729
A18.6	Tuberculosis of ear	0174A	01732
A18.6	Tuberculosis of ear		01739
A18.7	Tuberculosis of adrenal glands	0176A	
A18.8	Tuberculosis of other specified organs	0175A	01791
A18.8	Tuberculosis of other specified organs	0177A	01793
A18.8	Tuberculosis of other specified organs	0178A	01794
A18.8	Tuberculosis of other specified organs	0178X	01795
A18.8	Tuberculosis of other specified organs		01796
A19*	Miliary tuberculosis		
A19.2	Acute miliary tuberculosis, unspecified	0180	01800
A19.2	Acute miliary tuberculosis, unspecified		01810
A19.9	Miliary tuberculosis, unspecified	0189	01898
A20	Plague	020	020
A20.0	Bubonic plague	0200A	02000
A20.1	Cellulocutaneous plague	0201A	
A20.2	Pneumonic plague	0203A	02010
A20.2	Pneumonic plague	0204A	
A20.2	Pneumonic plague	0205A	
A20.3	Plague meningitis		
A20.7	Septicaemic plague	0202A	
A20.8	Other forms of plague	0208X	
A20.9	Plague, unspecified	0209X	02099
A21	Tularaemia	021	021
A21.0	Ulceroglandular tularaemia	0210A	02100
A21.1	Oculoglandular tularaemia	0213A	02101
A21.2	Pulmonary tularaemia	0212A	
A21.3	Gastrointestinal tularaemia	0211A	
A21.7	Generalized tularaemia		02102
A21.8	Other forms of tularaemia	0218X	02106
A21.8	Other forms of tularaemia		02107
A21.9	Tularaemia, unspecified	0219X	02109
A22	Anthrax	022	022
A22.0	Cutaneous anthrax	0220A	
A22.1	Pulmonary anthrax	0221A	
A22.2	Gastrointestinal anthrax	0222A	
A22.7	Anthrax sepsis	0223A	
A22.8	Other forms of anthrax	0228X	
A22.9	Anthrax, unspecified	0229X	
A23	Brucellosis	023	023
A23.0	Brucellosis due to Brucella melitensis	0230A	02300
A23.1	Brucellosis due to Brucella abortus	0231A	02310
A23.2	Brucellosis due to Brucella suis	0232A	02320

A23.3	Brucellosis due to Brucella canis	0233A	
A23.8	Other brucellosis	0238X	
A23.9	Brucellosis, unspecified	0239X	02397
A23.9	Brucellosis, unspecified		02399
A24	Glanders and melioidosis		
A24.0	Glanders	024	024
A24.1	Acute and fulminating melioidosis		
A24.2	Subacute and chronic melioidosis		
A24.3	Other melioidosis		
A24.4	Melioidosis, unspecified	025	025
A25	Rat-bite fevers		
A25.0	Spirillosis		02600
A25.1	Streptobacillosis		02610
A25.9	Rat-bite fever, unspecified	026	02601
A25.9	Rat-bite fever, unspecified		02699
A26	Erysipeloid	0271A	02710
A26.0	Cutaneous erysipeloid		
A26.7	Erysipelothrix sepsis		
A26.8	Other forms of erysipeloid		
A26.9	Erysipeloid, unspecified		
A27	Leptospirosis	100	100
A28	Other zoonotic bacterial diseases, not elsewhere classified		
A28.0	Pasteurellosis	0272A	
A28.1	Cat-scratch disease	0783A	07930
A28.2	Extraintestinal yersiniosis		0391
A28.8	Other specified zoonotic bacterial diseases, not elsewhere classified	0278X	
A28.9	Zoonotic bacterial disease, unspecified	0279X	02798
A30	Leprosy [Hansen disease]	030	030
A31	Infection due to other mycobacteria	031	031
A32	Listeriosis		
A32.0	Cutaneous listeriosis		
A32.1	Listerial meningitis and meningoencephalitis	0270A	02701
A32.7	Listerial sepsis		
A32.8	Other forms of listeriosis	0270X	02708
A32.9	Listeriosis, unspecified		02709
A33	Tetanus neonatorum	7713A	
A34*	Obstetrical tetanus		
A35	Other tetanus	037	037
A36	Diphtheria	032	032
A37	Whooping cough	033	033
A38	Scarlet fever	0341	0341
A39	Meningococcal infection	036	036
A39.0	Meningococcal meningitis	0360A	03600
A39.1	Waterhouse-Friderichsen syndrome	0363A	03611
A39.2	Acute meningococcaemia		
A39.3	Chronic meningococcaemia		03612
A39.4	Meningococcaemia, unspecified	0362A	03610
A39.5	Meningococcal heart disease	0364A	
A39.8	Other meningococcal infections	0361A	03680
A39.8	Other meningococcal infections	0361B	

A39.8	Other meningococcal infections	0368A	
A39.8	Other meningococcal infections	0368X	
A39.9	Meningococcal infection, unspecified	0369X	03697
A40	Streptococcal sepsis		
A40.0	Sepsis due to streptococcus, group A		
A40.1	Sepsis due to streptococcus, group B		
A40.2	Sepsis due to streptococcus, group D		
A40.3	Sepsis due to Streptococcus pneumoniae	0382A	03820
A40.8	Other streptococcal sepsis	0380B	
A40.8	Other streptococcal sepsis	0380X	
A40.9	Streptococcal sepsis, unspecified	0380A	03800
A41	Other sepsis		
A41.0	Sepsis due to Staphylococcus aureus	0381A	
A41.1	Sepsis due to other specified staphylococcus	0381B	
A41.2	Sepsis due to unspecified staphylococcus		03810
A41.3	Sepsis due to Haemophilus influenzae	0384A	
A41.4	Sepsis due to anaerobes	0383A	
A41.5	Sepsis due to other Gram-negative organisms	0384B	03880
A41.5	Sepsis due to other Gram-negative organisms	0384C	
A41.5	Sepsis due to other Gram-negative organisms	0384X	
A41.8	Other specified sepsis	0380C	03888
A41.8	Other specified sepsis	0388X	
A41.9	Sepsis, unspecified		0389
A42	Actinomycosis	039	
A42	Actinomycosis	0402A	
A42.0	Pulmonary actinomycosis	0391A	
A42.1	Abdominal actinomycosis	0392A	
A42.2	Cervicofacial actinomycosis	0393A	
A42.7	Actinomycotic sepsis		
A42.8	Other forms of actinomycosis	0390A	
A42.9	Actinomycosis, unspecified		11399
A43	Nocardiosis		
A43.0	Pulmonary nocardiosis		
A43.1	Cutaneous nocardiosis		
A43.8	Other forms of nocardiosis		
A44	Bartonellosis		
A44.0	Systemic bartonellosis		
A44.1	Cutaneous and mucocutaneous bartonellosis		
A44.8	Other forms of bartonellosis		
A44.9	Bartonellosis, unspecified	0880A	08900
A46	Erysipelas	035	035
A48.0	Gas gangrene	0400A	0390A
A48.1	Legionnaires disease		
A48.2	Nonpneumonic Legionnaires disease [Pontiac fever]		
A48.3	Toxic shock syndrome		
A48.4	Brazilian purpuric fever		
A48.8	Other specified bacterial diseases	0401A	03910
A48.8	Other specified bacterial diseases	0403A	
A48.8	Other specified bacterial diseases	0408X	
A49	Bacterial infection of unspecified site		

A49.0	Staphylococcal infection, unspecified site		03990
A49.1	Streptococcal infection, unspecified site		
A49.2	Haemophilus influenzae infection, unspecified site		
A49.3	Mycoplasma infection, unspecified site		
A49.8	Other bacterial infections of unspecified site	0418X	03991
A49.8	Other bacterial infections of unspecified site		03992
A49.8	Other bacterial infections of unspecified site		03993
A49.8	Other bacterial infections of unspecified site		03998
A49.9	Bacterial infection, unspecified	0419X	
A50	Congenital syphilis	090	
A50.0	Early congenital syphilis, symptomatic	0900A	09000
A50.1	Early congenital syphilis, latent	0901A	09010
A50.2	Early congenital syphilis, unspecified	0902A	09020
A50.3	Late congenital syphilitic oculopathy	0903A	09030
A50.4	Late congenital neurosyphilis [juvenile neurosyphilis]	0904A	09040
A50.5	Other late congenital syphilis, symptomatic	0905A	09050
A50.6	Late congenital syphilis, latent	0906A	09060
A50.7	Late congenital syphilis, unspecified	0907A	09070
A50.9	Congenital syphilis, unspecified	0909X	09097
A50.9	Congenital syphilis, unspecified		09099
A51	Early syphilis		
A51.0	Primary genital syphilis	0910A	09100
A51.1	Primary anal syphilis	0911A	
A51.2	Primary syphilis of other sites	0912A	
A51.3	Secondary syphilis of skin and mucous membranes	0913A	09120
A51.4	Other secondary syphilis	0914A	09188
A51.4	Other secondary syphilis	0915A	
A51.5	Early syphilis, latent	092	092
A51.9	Early syphilis, unspecified	0917A	09130
A51.9	Early syphilis, unspecified	0918X	09197
A51.9	Early syphilis, unspecified	0919X	09199
A52	Late syphilis		
A52.0	Cardiovascular syphilis	093	093
A52.1	Symptomatic neurosyphilis	0940A	09400
A52.1	Symptomatic neurosyphilis	0941A	09401
A52.1	Symptomatic neurosyphilis	0942A	09402
A52.1	Symptomatic neurosyphilis	0948X	09408
A52.1	Symptomatic neurosyphilis		09409
A52.1	Symptomatic neurosyphilis		09410
A52.1	Symptomatic neurosyphilis		09490
A52.1	Symptomatic neurosyphilis		09498
A52.2	Asymptomatic neurosyphilis	0943A	
A52.3	Neurosyphilis, unspecified	0949X	09491
A52.3	Neurosyphilis, unspecified		09499
A52.7	Other symptomatic late syphilis	095	095
A52.8	Late syphilis, latent	096	096
A52.9	Late syphilis, unspecified	0970A	
A53	Other and unspecified syphilis	097	097
A53.0	Latent syphilis, unspecified as early or late	0971A	
A53.9	Syphilis, unspecified	0979X	

A54	Gonococcal infection	098	098
A54.0	Gonococcal infection of lower genitourinary tract without periurethral or accessory gland abscess		09800
A54.1	Gonococcal infection of lower genitourinary tract with periurethral and accessory gland abscess		09802
A54.1	Gonococcal infection of lower genitourinary tract with periurethral and accessory gland abscess		09812
A54.2	Gonococcal pelviperitonitis and other gonococcal genitourinary infections	0981A	09801
A54.2	Gonococcal pelviperitonitis and other gonococcal genitourinary infections	0983A	09803
A54.2	Gonococcal pelviperitonitis and other gonococcal genitourinary infections		09804
A54.2	Gonococcal pelviperitonitis and other gonococcal genitourinary infections		09805
A54.2	Gonococcal pelviperitonitis and other gonococcal genitourinary infections		09811
A54.2	Gonococcal pelviperitonitis and other gonococcal genitourinary infections		09813
A54.2	Gonococcal pelviperitonitis and other gonococcal genitourinary infections		09814
A54.2	Gonococcal pelviperitonitis and other gonococcal genitourinary infections		09815
A54.3	Gonococcal infection of eye	0984A	
A54.3	Gonococcal infection of eye		09820
A54.3	Gonococcal infection of eye		09821
A54.3	Gonococcal infection of eye		09828
A54.4	Gonococcal infection of musculoskeletal system	0985A	
A54.4	Gonococcal infection of musculoskeletal system		09839
A54.5	Gonococcal pharyngitis	0986A	
A54.6	Gonococcal infection of anus and rectum	0987A	
A54.8	Other gonococcal infections	0988A	
A54.8	Other gonococcal infections	0988B	
A54.8	Other gonococcal infections	0988X	
A54.9	Gonococcal infection, unspecified	0980A	09806
A54.9	Gonococcal infection, unspecified	0982A	09808
A54.9	Gonococcal infection, unspecified		09809
A54.9	Gonococcal infection, unspecified		09810
A54.9	Gonococcal infection, unspecified		09816
A54.9	Gonococcal infection, unspecified		09818
A54.9	Gonococcal infection, unspecified		09888
A54.9	Gonococcal infection, unspecified		09899
A55	Chlamydial lymphogranuloma (venereum)	0991A	09919
A56.0	Chlamydial infection of lower genitourinary tract	0769A	
A56.1	Chlamydial infection of pelviperitoneum and other genitourinary organs	0769C	
A56.2	Chlamydial infection of genitourinary tract, unspecified		
A56.3	Chlamydial infection of anus and rectum	0769B	
A56.4	Chlamydial infection of pharynx	0769E	
A56.8	Sexually transmitted chlamydial infection of other sites		
A57	Chancroid	0990A	09909
A58	Granuloma inguinale	0992A	09929
A59*	Trichomoniasis		
A59.0	Urogenital trichomoniasis	1310A	13197
A59.0	Urogenital trichomoniasis		13199
A59.8	Trichomoniasis of other sites	1318X	
A59.9	Trichomoniasis, unspecified	1319X	
A60*	Anogenital herpesviral [herpes simplex] infection		
A60.0	Herpesviral infection of genitalia and urogenital tract	0541	05402
A63.0	Anogenital (venereal) warts	0781C	
A63.0	Anogenital (venereal) warts		09992
A63.8	Other specified predominantly sexually transmitted diseases	0998X	09996

A64	Unspecified sexually transmitted disease	0994A	09990
A64	Unspecified sexually transmitted disease	0999X	09991
A64	Unspecified sexually transmitted disease		09997
A64	Unspecified sexually transmitted disease		09999
A65	Nonvenereal syphilis	1040A	10400
A66	Yaws	102	10299
A67	Pinta [carate]	103	10399
A68	Relapsing fevers	0870A	08899
A68	Relapsing fevers	0871A	
A68	Relapsing fevers	0879X	
A69	Other spirochaetal infections		
A69.0	Necrotizing ulcerative stomatitis		
A69.1	Other Vincent infections	101	10199
A69.1	Other Vincent infections	1010A	
A69.2	Lyme disease	1048A	
A69.2	Lyme disease	1048B	
A69.8	Other specified spirochaetal infections	1048X	10498
A69.9	Spirochaetal infection, unspecified	1049X	
A70	Chlamydia psittaci infection	073	073
A71	Trachoma	0760A	07699
A74.0	Chlamydial conjunctivitis		
A74.8	Other chlamydial diseases	0769D	
A74.9	Chlamydial infection, unspecified	0769X	
A75	Typhus fever		
A75.0	Epidemic louse-borne typhus fever due to Rickettsia prowazekii	080	080
A75.1	Recrudescent typhus [Brill disease]	0811A	08110
A75.2	Typhus fever due to Rickettsia typhi	0810A	08100
A75.3	Typhus fever due to Rickettsia tsutsugamushi	0812A	
A75.9	Typhus fever, unspecified	0819X	08197
A75.9	Typhus fever, unspecified		08199
A77	Spotted fever [tick-borne rickettsioses]		
A77.0	Spotted fever due to Rickettsia rickettsii	0820A	08200
A77.1	Spotted fever due to Rickettsia conorii	0821A	08210
A77.2	Spotted fever due to Rickettsia sibirica		
A77.3	Spotted fever due to Rickettsia australis		
A77.8	Other spotted fevers	0828X	
A77.9	Spotted fever, unspecified	0829X	08229
A77.9	Spotted fever, unspecified		08220
A78	Q fever	0830A	08310
A79	Other rickettsioses		
A79.0	Trench fever	0831A	08320
A79.1	Rickettsialpox due to Rickettsia akari	0832A	08300
A79.8	Other specified rickettsioses	0838X	08388
A79.9	Rickettsiosis, unspecified	0839X	08120
A79.9	Rickettsiosis, unspecified		08397
A79.9	Rickettsiosis, unspecified		08399
A80	Acute poliomyelitis	0450A	04199
A80	Acute poliomyelitis	0450B	
A80	Acute poliomyelitis	0450C	
A80	Acute poliomyelitis	0450X	04001

A80	Acute poliomyelitis	0451A	
A80	Acute poliomyelitis	0451B	
A80	Acute poliomyelitis	0451C	
A80	Acute poliomyelitis	0451X	04000
A80	Acute poliomyelitis	0452A	
A80	Acute poliomyelitis	0452B	
A80	Acute poliomyelitis	0452C	
A80	Acute poliomyelitis	0452X	04299
A80	Acute poliomyelitis	0459A	04397
A80	Acute poliomyelitis	0459B	04399
A80	Acute poliomyelitis	0459C	
A80	Acute poliomyelitis	0459X	04007
A81.0	Creutzfeldt-Jakob disease	0461A	
A81.1	Subacute sclerosing panencephalitis	0462A	
A81.1	Subacute sclerosing panencephalitis		32301
A81.2	Progressive multifocal leukoencephalopathy	0463A	
A81.8	Other atypical virus infections of central nervous system	0460A	
A81.8	Other atypical virus infections of central nervous system	0468X	
A81.9	Atypical virus infection of central nervous system, unspecified	0469X	
A83*	Mosquito-borne viral encephalitis		
A83.0	Japanese encephalitis	0620A	06200
A83.1	Western equine encephalitis	0621A	06210
A83.2	Eastern equine encephalitis	0622A	06220
A83.3	St Louis encephalitis	0623A	06230
A83.4	Australian encephalitis	0624A	06240
A83.5	California encephalitis	0625A	
A83.8	Other mosquito-borne viral encephalitis	0625B	06298
A83.8	Other mosquito-borne viral encephalitis	0628X	
A83.9	Mosquito-borne viral encephalitis, unspecified	0629X	06297
A83.9	Mosquito-borne viral encephalitis, unspecified		06299
A84	Tick-borne viral encephalitis	063	063
A85*	Other viral encephalitis, not elsewhere classified		
A85.0	Enteroviral encephalitis	0480A	04699
A85.1	Adenoviral encephalitis	0491A	
A85.2	Arthropod-borne viral encephalitis, unspecified	0640A	06499
A85.8	Other specified viral encephalitis		06590
A86	Unspecified viral encephalitis		06599
A87*	Viral meningitis		
A87.0	Enteroviral meningitis	047	04500
A87.0	Enteroviral meningitis		04510
A87.0	Enteroviral meningitis		04597
A87.0	Enteroviral meningitis		04599
A87.2	Lymphocytic choriomeningitis	0490A	
A87.2	Lymphocytic choriomeningitis		07920
A88*	Other viral infections of central nervous system, not elsewhere classified		
A88.8	Other specified viral infections of central nervous system	0498X	
A89	Unspecified viral infection of central nervous system	0499X	
A90	Dengue fever [classical dengue]	061	061
A91*	Dengue haemorrhagic fever		
A92	Other mosquito-borne viral fevers		

A92.0	Chikungunya virus disease		
A92.1	O'nyong-nyong fever		
A92.2	Venezuelan equine fever	0662A	
A92.3	West Nile virus infection		
A92.4	Rift Valley fever		
A92.8	Other specified mosquito-borne viral fevers	0663A	06820
A92.8	Other specified mosquito-borne viral fevers	0663B	
A92.9	Mosquito-borne viral fever, unspecified		
A93	Other arthropod-borne viral fevers, not elsewhere classified		
A93.0	Oropouche virus disease		
A93.1	Sandfly fever		06800
A93.2	Colorado tick fever		
A93.8	Other specified arthropod-borne viral fevers	0661A	06810
A93.8	Other specified arthropod-borne viral fevers	0668X	06898
A94	Unspecified arthropod-borne viral fever	0669X	
A95	Yellow fever	060	060
A96*	Arenaviral haemorrhagic fever		
A96.9	Arenaviral haemorrhagic fever, unspecified	0787A	
A97*	Dengue		
A98	Other viral haemorrhagic fevers, not elsewhere classified		
A98.0	Crimean-Congo haemorrhagic fever	0650A	06700
A98.1	Omsk haemorrhagic fever	0651A	06710
A98.2	Kyasanur Forest disease	0652A	06720
A98.3	Marburg virus disease		
A98.4	Ebola virus disease		
A98.5	Haemorrhagic fever with renal syndrome	0786A	06750
A98.8	Other specified viral haemorrhagic fevers	0653A	06738
A98.8	Other specified viral haemorrhagic fevers	0654A	06740
A98.8	Other specified viral haemorrhagic fevers	0658X	
A99	Unspecified viral haemorrhagic fever	0659X	06790
A99	Unspecified viral haemorrhagic fever	0787X	
B00	Herpesviral [herpes simplex] infections		
B00.0	Eczema herpeticum	0540A	05403
B00.1	Herpesviral vesicular dermatitis	0547A	
B00.1	Herpesviral vesicular dermatitis	0549X	05400
B00.2	Herpesviral gingivostomatitis and pharyngotonsillitis	0542A	05401
B00.3	Herpesviral meningitis	0543A	
B00.4	Herpesviral encephalitis	0543B	05404
B00.5	Herpesviral ocular disease	0544	05405
B00.7	Disseminated herpesviral disease	0545A	
B00.8	Other forms of herpesviral infection	0542B	05406
B00.8	Other forms of herpesviral infection	0546A	
B00.8	Other forms of herpesviral infection	0548X	
B00.8	Other forms of herpesviral infection	0574X	
B00.9	Herpesviral infection, unspecified		05407
B00.9	Herpesviral infection, unspecified		05409
B01	Varicella [chickenpox]	052	052
B01.0	Varicella meningitis		
B01.1	Varicella encephalitis	0520A	05200
B01.2	Varicella pneumonia	0520B	

B01.8	Varicella with other complications	0527A	05201
B01.9	Varicella without complication	0529X	05207
B01.9	Varicella without complication		05209
B02	Zoster [herpes zoster]	053	053
B02.0	Zoster encephalitis		
B02.1	Zoster meningitis	0530A	
B02.2	Zoster with other nervous system involvement	0531A	
B02.3	Zoster ocular disease	0532A	05300
B02.3	Zoster ocular disease	0532B	
B02.3	Zoster ocular disease	0532C	
B02.3	Zoster ocular disease	0532X	
B02.7	Disseminated zoster	0538A	05302
B02.8	Zoster with other complications	0537A	05301
B02.8	Zoster with other complications	0538X	05306
B02.9	Zoster without complication	0539X	05307
B02.9	Zoster without complication		05309
B04*	Monkeypox		
B05	Measles	055	055
B05.0	Measles complicated by encephalitis	0550A	
B05.1	Measles complicated by meningitis		
B05.2	Measles complicated by pneumonia	0551A	
B05.3	Measles complicated by otitis media	0552A	
B05.4	Measles with intestinal complications		
B05.8	Measles with other complications	0557X	
B05.8	Measles with other complications	0558X	
B05.9	Measles without complication	0559X	05597
B05.9	Measles without complication		05599
B06	Rubella [German measles]	056	056
B06.0	Rubella with neurological complications	0560A	
B06.8	Rubella with other complications	0567A	
B06.8	Rubella with other complications	0567X	
B06.8	Rubella with other complications	0568X	
B06.9	Rubella without complication	0569A	05697
B06.9	Rubella without complication	0569X	05699
B07	Viral warts	0781A	
B07	Viral warts	0781B	07910
B08.0	Other orthopoxvirus infections	051	051
B08.1	Molluscum contagiosum	0780A	07900
B08.2	Exanthema subitum [sixth disease]	0578A	05710
B08.3	Erythema infectiosum [fifth disease]	0570A	05700
B08.4	Enteroviral vesicular stomatitis with exanthem	0743A	07940
B08.5	Enteroviral vesicular pharyngitis	0740A	07400
B08.8	Other specified viral infections characterized by skin and mucous membrane lesions	0578X	05788
B09	Unspecified viral infection characterized by skin and mucous membrane lesions	0579X	05797
B09	Unspecified viral infection characterized by skin and mucous membrane lesions		05799
B15	Acute hepatitis A		
B15.0	Hepatitis A with hepatic coma	0700A	
B15.9	Hepatitis A without hepatic coma	0701A	
B16	Acute hepatitis B		
B16.0	Acute hepatitis B with delta-agent (coinfection) with hepatic coma		

B16.1	Acute hepatitis B with delta-agent (coinfection) without hepatic coma		
B16.2	Acute hepatitis B without delta-agent with hepatic coma		
B16.9	Acute hepatitis B without delta-agent and without hepatic coma		
B17*	Other acute viral hepatitis		
B18*	Chronic viral hepatitis		
B18.1	Chronic viral hepatitis B without delta-agent	V026A	
B18.1	Chronic viral hepatitis B without delta-agent	V026B	
B19	Unspecified viral hepatitis		
B19.0	Unspecified viral hepatitis with hepatic coma	0702A	
B19.0	Unspecified viral hepatitis with hepatic coma	0704A	
B19.0	Unspecified viral hepatitis with hepatic coma	0706X	
B19.9	Unspecified viral hepatitis without hepatic coma	0703A	
B19.9	Unspecified viral hepatitis without hepatic coma	0705A	
B19.9	Unspecified viral hepatitis without hepatic coma	0709X	
B19.9	Unspecified viral hepatitis without hepatic coma		07001
B19.9	Unspecified viral hepatitis without hepatic coma		07002
B19.9	Unspecified viral hepatitis without hepatic coma		07003
B19.9	Unspecified viral hepatitis without hepatic coma		07004
B19.9	Unspecified viral hepatitis without hepatic coma		07007
B19.9	Unspecified viral hepatitis without hepatic coma		07009
B20*	Human immunodeficiency virus [HIV] disease resulting in infectious and parasitic diseases		
B21*	Human immunodeficiency virus [HIV] disease resulting in malignant neoplasms		
B21.0	HIV disease resulting in Kaposi sarcoma		
B22	Human immunodeficiency virus [HIV] disease resulting in other specified diseases		
B22.0	HIV disease resulting in encephalopathy		
B22.1	HIV disease resulting in lymphoid interstitial pneumonitis		
B22.2	HIV disease resulting in wasting syndrome		
B22.7	HIV disease resulting in multiple diseases classified elsewhere		
B23*	Human immunodeficiency virus [HIV] disease resulting in other conditions		
B24	Unspecified human immunodeficiency virus [HIV] disease	0788A	
B24	Unspecified human immunodeficiency virus [HIV] disease	0788B	
B24	Unspecified human immunodeficiency virus [HIV] disease	0788C	
B25*	Cytomegaloviral disease		
B25.0	Cytomegaloviral pneumonitis	0785C	
B25.1	Cytomegaloviral hepatitis	0785B	
B25.8	Other cytomegaloviral diseases	0785X	
B25.9	Cytomegaloviral disease, unspecified		07951
B25.9	Cytomegaloviral disease, unspecified		07959
B26	Mumps	072	072
B26.0	Mumps orchitis	0720A	07202
B26.1	Mumps meningitis	0721A	
B26.2	Mumps encephalitis	0722A	07201
B26.3	Mumps pancreatitis	0723A	07200
B26.8	Mumps with other complications	0727X	07203
B26.8	Mumps with other complications	0728X	07204
B26.8	Mumps with other complications		07206
B26.9	Mumps without complication	0729X	07207
B26.9	Mumps without complication		07209
B27	Infectious mononucleosis		
B27.0	Gammaherpesviral mononucleosis	0750A	

B27.0	Gammaherpesviral mononucleosis	0750X	
B27.1	Cytomegaloviral mononucleosis	0785A	
B27.8	Other infectious mononucleosis		
B27.9	Infectious mononucleosis, unspecified		07501
G05.1*B27.9	Infectious mononucleosis, unspecified		07502
B27.9	Infectious mononucleosis, unspecified		07507
B27.9	Infectious mononucleosis, unspecified		07508
B27.9	Infectious mononucleosis, unspecified		07509
B30	Viral conjunctivitis	0770A	07800
B30	Viral conjunctivitis	0771A	07810
B30	Viral conjunctivitis	0772A	07820
B30	Viral conjunctivitis	0773A	07880
B30	Viral conjunctivitis	0774A	07899
B30	Viral conjunctivitis	0778X	
B30	Viral conjunctivitis	0779X	
B33	Other viral diseases, not elsewhere classified		
B33.0	Epidemic myalgia	0741A	07410
B33.1	Ross River disease		
B33.2	Viral carditis		
B33.3	Retrovirus infections, not elsewhere classified	0788D	
B33.4	Hantavirus (cardio-)pulmonary syndrome [HPS] [HCPS]		
B33.8	Other specified viral diseases	0788X	
B34	Viral infection of unspecified site		
B34.0	Adenovirus infection, unspecified site		07981
B34.1	Enterovirus infection, unspecified site		07499
B34.1	Enterovirus infection, unspecified site		07980
B34.2	Coronavirus infection, unspecified site		
B34.3	Parvovirus infection, unspecified site		
B34.4	Papovavirus infection, unspecified site		
B34.8	Other viral infections of unspecified site		07988
B34.9	Viral infection, unspecified		07997
B34.9	Viral infection, unspecified		07999
B35	Dermatophytosis	110	110
B36	Other superficial mycoses	111	111
B37	Candidiasis	112	112
B37.0	Candidal stomatitis	1120A	
B37.1	Pulmonary candidiasis	1124A	
B37.2	Candidiasis of skin and nail	1123A	
B37.3	Candidiasis of vulva and vagina	1121A	
B37.4	Candidiasis of other urogenital sites	1122A	
B37.4	Candidiasis of other urogenital sites	1122X	
B37.5	Candidal meningitis	1128C	
B37.6	Candidal endocarditis	1128A	
B37.7	Candidal sepsis	1125A	
B37.8	Candidiasis of other sites	1120B	
B37.8	Candidiasis of other sites	1128B	
B37.8	Candidiasis of other sites	1128X	
B37.9	Candidiasis, unspecified	1129X	
B38	Coccidioidomycosis	114	114
B38.0	Acute pulmonary coccidioidomycosis		

B38.1	Chronic pulmonary coccidioidomycosis		
B38.2	Pulmonary coccidioidomycosis, unspecified		
B38.3	Cutaneous coccidioidomycosis		
B38.4	Coccidioidomycosis meningitis		
B38.7	Disseminated coccidioidomycosis		
B38.8	Other forms of coccidioidomycosis		
B38.9	Coccidioidomycosis, unspecified		
B39	Histoplasmosis	115	115
B39.0	Acute pulmonary histoplasmosis capsulati		
B39.1	Chronic pulmonary histoplasmosis capsulati		
B39.2	Pulmonary histoplasmosis capsulati, unspecified		
B39.3	Disseminated histoplasmosis capsulati		
B39.4	Histoplasmosis capsulati, unspecified		
B39.5	Histoplasmosis duboisii		
B39.9	Histoplasmosis, unspecified		
B40	Blastomycosis	116	116
B40.0	Acute pulmonary blastomycosis		
B40.1	Chronic pulmonary blastomycosis		
B40.2	Pulmonary blastomycosis, unspecified		
B40.3	Cutaneous blastomycosis		
B40.7	Disseminated blastomycosis		
B40.8	Other forms of blastomycosis		
B40.9	Blastomycosis, unspecified		
B41	Paracoccidioidomycosis		
B41.0	Pulmonary paracoccidioidomycosis		
B41.7	Disseminated paracoccidioidomycosis		
B41.8	Other forms of paracoccidioidomycosis		
B41.9	Paracoccidioidomycosis, unspecified		
B42	Sporotrichosis		
B42.0	Pulmonary sporotrichosis		
B42.1	Lymphocutaneous sporotrichosis		
B42.7	Disseminated sporotrichosis		
B42.8	Other forms of sporotrichosis		
B42.9	Sporotrichosis, unspecified	1171A	11710
B43	Chromomycosis and phaeomycotic abscess		
B43.0	Cutaneous chromomycosis		
B43.1	Phaeomycotic brain abscess		
B43.2	Subcutaneous phaeomycotic abscess and cyst		
B43.8	Other forms of chromomycosis		
B43.9	Chromomycosis, unspecified	1172A	11720
B44*	Aspergillosis		
B44.9	Aspergillosis, unspecified	1173A	11730
B45	Cryptococcosis		
B45.0	Pulmonary cryptococcosis		
B45.1	Cerebral cryptococcosis		
B45.2	Cutaneous cryptococcosis		
B45.3	Osseous cryptococcosis		
B45.7	Disseminated cryptococcosis		
B45.8	Other forms of cryptococcosis		
B45.9	Cryptococcosis, unspecified	1175A	

B46	Zygomycosis		
B46.0	Pulmonary mucormycosis		
B46.1	Rhinocerebral mucormycosis		
B46.2	Gastrointestinal mucormycosis		
B46.3	Cutaneous mucormycosis		
B46.4	Disseminated mucormycosis		
B46.5	Mucormycosis, unspecified	1177A	
B46.8	Other zygomycoses		
B46.9	Zygomycosis, unspecified		
B47	Mycetoma		
B47.0	Eumycetoma	1174A	11740
B47.1	Actinomycetoma	0394A	
B47.9	Mycetoma, unspecified		
B48	Other mycoses, not elsewhere classified		
B48.0	Lobomycosis		
B48.1	Rhinosporidiosis	1170A	11700
B48.2	Allescheriasis	1176A	
B48.3	Geotrichosis		
B48.4	Penicillosis		
B48.7	Opportunistic mycoses	118	
B48.8	Other specified mycoses	1178A	11788
B48.8	Other specified mycoses	1178B	
B49	Unspecified mycosis	117	
B49	Unspecified mycosis	1179X	11799
B50*	Plasmodium falciparum malaria		
B50.0	Plasmodium falciparum malaria with cerebral complications		
B50.8	Other severe and complicated Plasmodium falciparum malaria	0848A	
B50.9	Plasmodium falciparum malaria, unspecified	0840A	08400
B51	Plasmodium vivax malaria	0841A	08410
B52	Plasmodium malariae malaria	0842A	08420
B53	Other parasitologically confirmed malaria		
B53.0	Plasmodium ovale malaria	0843A	08430
B53.1	Malaria due to simian plasmodia		
B53.8	Other parasitologically confirmed malaria, not elsewhere classified	0844X	08488
B54	Unspecified malaria	0845A	08440
B54	Unspecified malaria	0846X	08450
B54	Unspecified malaria	0847A	08497
B54	Unspecified malaria	0849X	08499
B55.0	Visceral leishmaniasis	0850A	08500
B55.1	Cutaneous leishmaniasis	0851A	08510
B55.2	Mucocutaneous leishmaniasis	0855A	08520
B55.9	Leishmaniasis, unspecified	085	08599
B55.9	Leishmaniasis, unspecified	0859X	08597
B56*	African trypanosomiasis		
B56.0	Gambiense trypanosomiasis	0863A	
B56.1	Rhodesiense trypanosomiasis	0864A	
B56.9	African trypanosomiasis, unspecified	0865A	
B57*	Chagas disease		
B57.0	Acute Chagas disease with heart involvement	0860A	
B57.2	Chagas disease (chronic) with heart involvement	0861A	

B57.2	Chagas disease (chronic) with heart involvement	0862A	08699
B58	Toxoplasmosis		
B58.0	Toxoplasma oculopathy	1302A	
B58.1	Toxoplasma hepatitis	1305A	
B58.2	Toxoplasma meningoencephalitis	1300A	
B58.3	Pulmonary toxoplasmosis	1304A	
B58.8	Toxoplasmosis with other organ involvement	1303A	
B58.8	Toxoplasmosis with other organ involvement	1307X	
B58.8	Toxoplasmosis with other organ involvement	1308A	
B58.9	Toxoplasmosis, unspecified	1308X	13000
B58.9	Toxoplasmosis, unspecified	1309X	13099
B59	Pneumocystosis	1363A	13601
B60.0	Babesiosis		
B60.1	Acanthamoebiasis		
B60.2	Naegleriasis	1362A	
B60.8	Other specified protozoal diseases		08799
B64	Unspecified protozoal disease	0869X	
B64	Unspecified protozoal disease		13602
B65	Schistosomiasis [bilharziasis]	120	
B65.0	Schistosomiasis due to Schistosoma haematobium [urinary schistosomiasis]	1200A	12000
B65.1	Schistosomiasis due to Schistosoma mansoni [intestinal schistosomiasis]	1201A	12010
B65.2	Schistosomiasis due to Schistosoma japonicum	1202A	12020
B65.3	Cercarial dermatitis	1203A	12030
B65.8	Other schistosomiases	1208X	12088
B65.9	Schistosomiasis, unspecified	1209X	12097
B65.9	Schistosomiasis, unspecified		12099
B66	Other fluke infections	121	
B66.0	Opisthorchiasis	1210A	
B66.1	Clonorchiasis	1211A	
B66.2	Dicrocoeliasis		
B66.3	Fascioliasis	1213A	
B66.4	Paragonimiasis	1212A	
B66.5	Fasciolopsiasis		
B66.8	Other specified fluke infections	1218X	
B66.9	Fluke infection, unspecified	1219X	12199
B67	Echinococcosis	122	122
B68	Taeniasis		
B68.0	Taenia solium taeniasis	1230A	12300
B68.1	Taenia saginata taeniasis	1232A	12320
B68.9	Taeniasis, unspecified	1233A	12330
B69	Cysticercosis	1231A	12311
B69.0	Cysticercosis of central nervous system		
B69.1	Cysticercosis of eye		
B69.8	Cysticercosis of other sites		
B69.9	Cysticercosis, unspecified		
B70	Diphyllobothriasis and sparganosis		
B70.0	Diphyllobothriasis	1234A	12340
B70.1	Sparganosis	1235A	12350
B71	Other cestode infections		
B71.0	Hymenolepiasis	1236A	12360

B71.1	Dipylidiasis		
B71.8	Other specified cestode infections	1238X	12398
B71.9	Cestode infection, unspecified	123	
B71.9	Cestode infection, unspecified	1239X	12399
B72*	Dracunculiasis		
B73*	Onchocerciasis		
B74	Filariasis	125	125
B75	Trichinellosis	124	124
B76	Hookworm diseases	126	126
B77*	Ascariasis		
B77.9	Ascariasis, unspecified	1270A	12700
B78*	Strongyloidiasis		
B78.9	Strongyloidiasis, unspecified	1272A	12710
B79	Trichuriasis	1273A	12720
B80	Enterobiasis	1274A	12730
B81	Other intestinal helminthiases, not elsewhere classified		
B81.0	Anisakiasis	1271A	
B81.1	Intestinal capillariasis	1275A	
B81.2	Trichostrongyliasis	1276A	
B81.3	Intestinal angiostrongyliasis		
B81.4	Mixed intestinal helminthiases	1278X	12750
B81.8	Other specified intestinal helminthiases	1277X	12740
B82	Unspecified intestinal parasitism		
B82.0	Intestinal helminthiasis, unspecified	1279X	12799
B82.9	Intestinal parasitism, unspecified	1290A	12999
B82.9	Intestinal parasitism, unspecified	1299X	
B83	Other helminthiases		
B83.0	Visceral larva migrans	1280A	
B83.1	Gnathostomiasis	1281A	
B83.2	Angiostrongyliasis due to Parastrongylus cantonensis		
B83.3	Syngamiasis		
B83.4	Internal hirudiniasis		
B83.8	Other specified helminthiases	1288X	
B83.9	Helminthiasis, unspecified	1289X	12899
B85	Pediculosis and phthiriasis	132	132
B85	Pediculosis and phthiriasis		13410
B86	Scabies	1330A	13300
B87*	Myiasis		
B87.9	Myiasis, unspecified	1340A	13400
B88	Other infestations		
B88.0	Other acariasis	1338X	13390
B88.0	Other acariasis		13398
B88.1	Tungiasis [sandflea infestation]		
B88.2	Other arthropod infestations	1341A	13420
B88.3	External hirudiniasis		
B88.8	Other specified infestations	1348X	13498
B88.9	Infestation, unspecified	1339X	13399
B88.9	Infestation, unspecified	1342A	13430
B89	Unspecified parasitic disease	1349X	13499
B95*	Streptococcus and staphylococcus as the cause of diseases classified to other chapters		

B95.3	Streptococcus pneumoniae as the cause of diseases classified to other chapters	0412A	
B95.5	Unspecified streptococcus as the cause of diseases classified to other chapters	0410A	
B95.8	Unspecified staphylococcus as the cause of diseases classified to other chapters	0411A	
B96.0	Mycoplasma pneumoniae [M. pneumoniae] as the cause of diseases classified to other chapters		
B96.1	Klebsiella pneumoniae [K. pneumoniae] as the cause of diseases classified to other chapters	0413A	
B96.2	Escherichia coli [E. coli] as the cause of diseases classified to other chapters	0414A	
B96.3	Haemophilus influenzae [H. influenzae] as the cause of diseases classified to other chapters	0415A	
B96.4	Proteus (mirabilis)(morganii) as the cause of diseases classified to other chapters	0416A	
B96.5	Pseudomonas (aeruginosa) as the cause of diseases classified to other chapters	0417A	
B96.6	Bacillus fragilis [B. fragilis] as the cause of diseases classified to other chapters		
B96.7	Clostridium perfringens [C. perfringens] as the cause of diseases classified to other chapters		
B96.8	Other specified bacterial agents as the cause of diseases classified to other chapters		
B97	Viral agents as the cause of diseases classified to other chapters		
B97.0	Adenovirus as the cause of diseases classified to other chapters	0790A	
B97.1	Enterovirus as the cause of diseases classified to other chapters	0742A	07401
B97.1	Enterovirus as the cause of diseases classified to other chapters	0748X	07420
B97.1	Enterovirus as the cause of diseases classified to other chapters	0791A	
B97.1	Enterovirus as the cause of diseases classified to other chapters	0793A	
B97.1	Enterovirus as the cause of diseases classified to other chapters		07421
B97.1	Enterovirus as the cause of diseases classified to other chapters		07498
B97.2	Coronavirus as the cause of diseases classified to other chapters		
B97.3	Retrovirus as the cause of diseases classified to other chapters		
B97.4	Respiratory syncytial virus as the cause of diseases classified to other chapters		
B97.5	Reovirus as the cause of diseases classified to other chapters		
B97.6	Parvovirus as the cause of diseases classified to other chapters		
B97.7	Papillomavirus as the cause of diseases classified to other chapters		
B97.8	Other viral agents as the cause of diseases classified to other chapters	0798X	
B97.8	Other viral agents as the cause of diseases classified to other chapters	0799X	
B98.0	Helicobacter pylori [H.pylori] as the cause of diseases classified to other chapters		
B98.1	Vibrio vulnificus as the cause of diseases classified to other chapters		
B99	Other and unspecified infectious diseases	0888X	
B99	Other and unspecified infectious diseases	0889X	
B99	Other and unspecified infectious diseases	1364A	
B99	Other and unspecified infectious diseases	1368X	
B99	Other and unspecified infectious diseases	1369X	
B99	Other and unspecified infectious diseases		08990
B99	Other and unspecified infectious diseases		13608
B99	Other and unspecified infectious diseases		13609
C46	Kaposi sarcoma		
D73.3	Abscess of spleen		
E32.1	Abscess of thymus	2541A	
G00	Bacterial meningitis, not elsewhere classified		
G00.0	Haemophilus meningitis	3200A	32000
G00.1	Pneumococcal meningitis	3201A	32010
G00.2	Streptococcal meningitis	3202A	32080
G00.3	Staphylococcal meningitis	3203A	
G00.8	Other bacterial meningitis	3208X	32088
G00.9	Bacterial meningitis, unspecified	3209X	32099
G01*	Meningitis in bacterial diseases classified elsewhere		
G02.0	Meningitis in viral diseases classified elsewhere		

G02.1	Meningitis in mycoses		
G02.8	Meningitis in other specified infectious and parasitic diseases classified elsewhere		
G03*	Meningitis due to other and unspecified causes		
G03.9	Meningitis, unspecified	3229X	
G04.0	Acute disseminated encephalitis	3235A	
G04.1	Tropical spastic paraplegia		
G04.2	Bacterial meningoencephalitis and meningomyelitis, not elsewhere classified	3238A	
G04.8	Other encephalitis, myelitis and encephalomyelitis	3238B	
G04.8	Other encephalitis, myelitis and encephalomyelitis	3238X	32300
G04.9	Encephalitis, myelitis and encephalomyelitis, unspecified	3239X	
G04.9	Encephalitis, myelitis and encephalomyelitis, unspecified		32308
G04.9	Encephalitis, myelitis and encephalomyelitis, unspecified		32309
G05.0	Encephalitis, myelitis and encephalomyelitis in bacterial diseases classified elsewhere		
G05.1	Encephalitis, myelitis and encephalomyelitis in viral diseases classified elsewhere		
G05.2	Encephalitis, myelitis and encephalomyelitis in other infectious and parasitic diseases classified elsewhere		
G06	Intracranial and intraspinal abscess and granuloma	3249X	32201
G06	Intracranial and intraspinal abscess and granuloma		32202
G06	Intracranial and intraspinal abscess and granuloma		32203
G06.0	Intracranial abscess and granuloma	3240A	32200
G06.1	Intraspinal abscess and granuloma	3241A	
G07*	Intracranial and intraspinal abscess and granuloma in diseases classified elsewhere		
H00*	Hordeolum and chalazion		
H00.0	Hordeolum and other deep inflammation of eyelid	3731A	36200
H00.0	Hordeolum and other deep inflammation of eyelid		36201
H00.0	Hordeolum and other deep inflammation of eyelid		36202
H00.0	Hordeolum and other deep inflammation of eyelid		36209
H00.1	Chalazion	3732A	37800
H01.0	Blepharitis		36101
H01.8	Other specified inflammation of eyelid	3738X	
H01.9	Inflammation of eyelid, unspecified	3739X	36109
H05.0	Acute inflammation of orbit	3760A	36900
H05.0	Acute inflammation of orbit		36901
H05.0	Acute inflammation of orbit		36902
H06.1	Parasitic infestation of orbit in diseases classified elsewhere		
H10.0	Mucopurulent conjunctivitis	3720A	36000
H10.5	Blepharoconjunctivitis	3730A	
H13.0	Filarial infection of conjunctiva		
H19.0	Scleritis and episcleritis in diseases classified elsewhere		
H19.1	Herpesviral keratitis and keratoconjunctivitis		
H19.2	Keratitis and keratoconjunctivitis in other infectious and parasitic diseases classified elsewhere		
H22.0	Iridocyclitis in infectious and parasitic diseases classified elsewhere		
H32.0	Chorioretinal inflammation in infectious and parasitic diseases classified elsewhere		
H44.0	Purulent endophthalmitis	3600A	36600
H44.0	Purulent endophthalmitis		36601
H60.0	Abscess of external ear		38000
H60.0	Abscess of external ear		38001
H60.1	Cellulitis of external ear	3800A	
H60.2	Malignant otitis externa	3801C	
H60.3	Other infective otitis externa	3801A	
H60.3	Other infective otitis externa	3801B	

H62.0	Otitis externa in bacterial diseases classified elsewhere		
H62.1	Otitis externa in viral diseases classified elsewhere		
H62.2	Otitis externa in mycoses	3801E	
H62.3	Otitis externa in other infectious and parasitic diseases classified elsewhere		
H66.0	Acute suppurative otitis media	3820A	38200
H67.0	Otitis media in bacterial diseases classified elsewhere		
H67.1	Otitis media in viral diseases classified elsewhere		
H70.0	Acute mastoiditis	3830A	38300
H75.0	Mastoiditis in infectious and parasitic diseases classified elsewhere		
I30.1	Infective pericarditis	4209A	
I32.0	Pericarditis in bacterial diseases classified elsewhere		
I32.1	Pericarditis in other infectious and parasitic diseases classified elsewhere		
I33.0	Acute and subacute infective endocarditis	4210A	42100
I33.0	Acute and subacute infective endocarditis	4219X	
I40.0	Infective myocarditis	4220A	
I41.0	Myocarditis in bacterial diseases classified elsewhere		
I41.1	Myocarditis in viral diseases classified elsewhere		
I41.2	Myocarditis in other infectious and parasitic diseases classified elsewhere		
I43.0	Cardiomyopathy in infectious and parasitic diseases classified elsewhere		
I52.0	Other heart disorders in bacterial diseases classified elsewhere		
I52.1	Other heart disorders in other infectious and parasitic diseases classified elsewhere		
I68.1	Cerebral arteritis in infectious and parasitic diseases classified elsewhere		
J01	Acute sinusitis	461	461
J01.0	Acute maxillary sinusitis		
J02	Acute pharyngitis		
J02.0	Streptococcal pharyngitis		
J02.8	Acute pharyngitis due to other specified organisms		
J02.9	Acute pharyngitis, unspecified	462	462
J03	Acute tonsillitis		
J03.0	Streptococcal tonsillitis	0340	0340
J03.8	Acute tonsillitis due to other specified organisms		
J03.9	Acute tonsillitis, unspecified	463	463
J04*	Acute laryngitis and tracheitis		
J04.0	Acute laryngitis	4640A	46401
J04.0	Acute laryngitis	4644A	
J04.1	Acute tracheitis	4641A	
J04.2	Acute laryngotracheitis	4642A	46403
J05.1	Acute epiglottitis	4643A	46404
J09*	Influenza due to certain identified influenza virus		
J10*	Influenza due to other identified influenza virus		
J11*	Influenza, virus not identified		
J11.0	Influenza with pneumonia, virus not identified	4870A	47101
J11.0	Influenza with pneumonia, virus not identified		47109
J11.1	Influenza with other respiratory manifestations, virus not identified	4871A	47201
J11.1	Influenza with other respiratory manifestations, virus not identified		47202
J11.1	Influenza with other respiratory manifestations, virus not identified		47209
J11.8	Influenza with other manifestations, virus not identified	4878X	47099
J11.8	Influenza with other manifestations, virus not identified		47399
J11.8	Influenza with other manifestations, virus not identified		47499
J12	Viral pneumonia, not elsewhere classified	480	480

J13	Pneumonia due to Streptococcus pneumoniae	481	481
J14	Pneumonia due to Haemophilus influenzae	4822A	48210
J15	Bacterial pneumonia, not elsewhere classified		
J15.0	Pneumonia due to Klebsiella pneumoniae	4820A	48201
J15.1	Pneumonia due to Pseudomonas	4821A	
J15.2	Pneumonia due to staphylococcus	4824A	48230
J15.3	Pneumonia due to streptococcus, group B		
J15.4	Pneumonia due to other streptococci		
J15.5	Pneumonia due to Escherichia coli		
J15.6	Pneumonia due to other aerobic Gram-negative bacteria		
J15.7	Pneumonia due to Mycoplasma pneumoniae	4830A	
J15.8	Other bacterial pneumonia	4828X	48298
J15.9	Bacterial pneumonia, unspecified	4823A	48220
J15.9	Bacterial pneumonia, unspecified	4829X	
J16	Pneumonia due to other infectious organisms, not elsewhere classified		
J16.0	Chlamydial pneumonia		
J16.8	Pneumonia due to other specified infectious organisms	4830X	48399
J17.0	Pneumonia in bacterial diseases classified elsewhere		
J17.1	Pneumonia in viral diseases classified elsewhere		
J17.2	Pneumonia in mycoses		
J17.3	Pneumonia in parasitic diseases		
J17.8	Pneumonia in other diseases classified elsewhere		
J18*	Pneumonia, organism unspecified		
J18.0	Bronchopneumonia, unspecified		48501
J18.0	Bronchopneumonia, unspecified		48502
J18.0	Bronchopneumonia, unspecified		48509
J18.9	Pneumonia, unspecified	4859X	486
J20	Acute bronchitis		
J20.0	Acute bronchitis due to Mycoplasma pneumoniae		
J20.1	Acute bronchitis due to Haemophilus influenzae		
J20.2	Acute bronchitis due to streptococcus		
J20.3	Acute bronchitis due to coxsackievirus		
J20.4	Acute bronchitis due to parainfluenza virus		
J20.5	Acute bronchitis due to respiratory syncytial virus		
J20.6	Acute bronchitis due to rhinovirus		
J20.7	Acute bronchitis due to echovirus		
J20.8	Acute bronchitis due to other specified organisms		
J20.9	Acute bronchitis, unspecified	4660A	
J21*	Acute bronchiolitis		
J21.0	Acute bronchiolitis due to respiratory syncytial virus		
J21.1	Acute bronchiolitis due to human metapneumovirus		
J21.8	Acute bronchiolitis due to other specified organisms		
J21.9	Acute bronchiolitis, unspecified	4661A	
J22	Unspecified acute lower respiratory infection		46699
J36	Peritonsillar abscess	475	50199
J39.0	Retropharyngeal and parapharyngeal abscess	4782A	
J39.0	Retropharyngeal and parapharyngeal abscess	4782B	
J39.1	Other abscess of pharynx		
J85.1	Abscess of lung with pneumonia		
J85.2	Abscess of lung without pneumonia	5130A	51399

J85.3	Abscess of mediastinum	5131A	51992
J86	Pyothorax	510	510
K02	Dental caries	5210A	52100
K04.0	Pulpitis	5220A	52200
K04.4	Acute apical periodontitis of pulpal origin	5224A	52240
K04.5	Chronic apical periodontitis	5226A	52260
K04.6	Periapical abscess with sinus		
K04.7	Periapical abscess without sinus	5225A	52250
K05.0	Acute gingivitis	5230A	52300
K05.2	Acute periodontitis	5233A	52330
K05.3	Chronic periodontitis	5234A	52340
K05.3	Chronic periodontitis		52350
K05.4	Periodontosis	5235A	
K11.3	Abscess of salivary gland	5273A	52730
K12.2	Cellulitis and abscess of mouth	5283A	52830
K23.0	Tuberculous oesophagitis		
K23.1	Megaoesophagus in Chagas disease		
K35	Acute appendicitis	540	540
K57.0	Diverticular disease of small intestine with perforation and abscess	5620B	56202
K57.0	Diverticular disease of small intestine with perforation and abscess	5620D	
K57.2	Diverticular disease of large intestine with perforation and abscess	5621B	
K57.2	Diverticular disease of large intestine with perforation and abscess	5621C	
K57.2	Diverticular disease of large intestine with perforation and abscess	5621D	56211
K57.4	Diverticular disease of both small and large intestine with perforation and abscess		
K57.8	Diverticular disease of intestine, part unspecified, with perforation and abscess		
K61	Abscess of anal and rectal regions	566	566
K63.0	Abscess of intestine	5695A	56900
K65.0	Acute peritonitis	5672A	56700
K65.0	Acute peritonitis	5672B	56701
K65.0	Acute peritonitis		56702
K65.0	Acute peritonitis		56704
K67.0	Chlamydial peritonitis		
K67.1	Gonococcal peritonitis		
K67.2	Syphilitic peritonitis		
K67.3	Tuberculous peritonitis		
K67.8	Other disorders of peritoneum in infectious diseases classified elsewhere		
K75.0	Abscess of liver	5720A	57299
K75.1	Phlebitis of portal vein	5721A	
K77.0	Liver disorders in infectious and parasitic diseases classified elsewhere		
L00*	Staphylococcal scalded skin syndrome		
L01	Impetigo	6868C	68690
L01	Impetigo		68691
L02	Cutaneous abscess, furuncle and carbuncle	680	680
L03*	Cellulitis		
L03.0	Cellulitis of finger and toe	681	681
L03.1	Cellulitis of other parts of limb	6823A	68220
L03.1	Cellulitis of other parts of limb	6824A	68230
L03.1	Cellulitis of other parts of limb	6826A	
L03.1	Cellulitis of other parts of limb	6827A	68250
L03.2	Cellulitis of face	6820A	

L03.3	Cellulitis of trunk	6822A	68210
L03.8	Cellulitis of other sites	6821A	68200
L03.8	Cellulitis of other sites	6825A	68240
L03.8	Cellulitis of other sites	6828A	
L03.8	Cellulitis of other sites	6828X	68298
L03.9	Cellulitis, unspecified	682	
L03.9	Cellulitis, unspecified	6829X	68299
L04	Acute lymphadenitis	683	683
L05*	Pilonidal cyst		
L05.0	Pilonidal cyst with abscess	6850A	
L08*	Other local infections of skin and subcutaneous tissue		
L08.0	Pyoderma	6860A	
L08.8	Other specified local infections of skin and subcutaneous tissue	6868A	
L08.8	Other specified local infections of skin and subcutaneous tissue	6868X	68698
L08.9	Local infection of skin and subcutaneous tissue, unspecified	684	684
L08.9	Local infection of skin and subcutaneous tissue, unspecified	6869X	
L70.1	Acne conglobata	7061E	
M00	Pyogenic arthritis		
M00.0	Staphylococcal arthritis and polyarthritis		
M00.1	Pneumococcal arthritis and polyarthritis		
M00.2	Other streptococcal arthritis and polyarthritis		
M00.8	Arthritis and polyarthritis due to other specified bacterial agents		
M00.9	Pyogenic arthritis, unspecified	7110A	71000
M00.9	Pyogenic arthritis, unspecified	7110B	71001
M00.9	Pyogenic arthritis, unspecified	7110C	71002
M00.9	Pyogenic arthritis, unspecified	7110D	71003
M00.9	Pyogenic arthritis, unspecified	7110E	71004
M00.9	Pyogenic arthritis, unspecified	7110F	71005
M00.9	Pyogenic arthritis, unspecified	7110G	71006
M00.9	Pyogenic arthritis, unspecified	7110H	71007
M00.9	Pyogenic arthritis, unspecified	7110K	71008
M00.9	Pyogenic arthritis, unspecified	7110L	71009
M00.9	Pyogenic arthritis, unspecified	7110X	
M01.0	Meningococcal arthritis		
M01.1	Tuberculous arthritis		
M01.2	Arthritis in Lyme disease		
M01.3	Arthritis in other bacterial diseases classified elsewhere		
M01.4	Rubella arthritis		
M01.5	Arthritis in other viral diseases classified elsewhere		
M01.6	Arthritis in mycoses		
M01.8	Arthritis in other infectious and parasitic diseases classified elsewhere		
M46.1	Sacroiliitis, not elsewhere classified	7202A	
M46.2	Osteomyelitis of vertebra	7208A	
M46.3	Infection of intervertebral disc (pyogenic)		
M46.5	Other infective spondylopathies		
M46.9	Inflammatory spondylopathy, unspecified	7209X	
M49.0	Tuberculosis of spine		
M49.1	Brucella spondylitis		
M49.2	Enterobacterial spondylitis		
M49.3	Spondylopathy in other infectious and parasitic diseases classified elsewhere		

M60.0	Infective myositis	7280A	
M63.0	Myositis in bacterial diseases classified elsewhere		
M63.1	Myositis in protozoal and parasitic infections classified elsewhere		
M63.2	Myositis in other infectious diseases classified elsewhere		
M65.0	Abscess of tendon sheath		
M65.1	Other infective (teno)synovitis		
M71.0	Abscess of bursa		
M71.1	Other infective bursitis		
M72.6	Necrotizing fasciitis		
M73.0	Gonococcal bursitis		
M73.1	Syphilitic bursitis		
M86*	Osteomyelitis		
M86.0	Acute haematogenous osteomyelitis		
M86.1	Other acute osteomyelitis	7300	72000
M86.2	Subacute osteomyelitis		
M86.3	Chronic multifocal osteomyelitis		
M86.4	Chronic osteomyelitis with draining sinus		
M86.5	Other chronic haematogenous osteomyelitis		
M86.6	Other chronic osteomyelitis	7301	72010
M86.8	Other osteomyelitis		
M86.9	Osteomyelitis, unspecified	7302	72029
M86.9	Osteomyelitis, unspecified	7303	72030
M86.9	Osteomyelitis, unspecified	7309X	72031
M86.9	Osteomyelitis, unspecified		72039
N08.0	Glomerular disorders in infectious and parasitic diseases classified elsewhere	5838C	
N10	Acute tubulo-interstitial nephritis	5901A	59010
N10	Acute tubulo-interstitial nephritis		59011
N10	Acute tubulo-interstitial nephritis		59012
N13.6	Pyonephrosis		59013
N13.6	Pyonephrosis		59014
N15.1	Renal and perinephric abscess	5902A	59020
N16.0	Renal tubulo-interstitial disorders in infectious and parasitic diseases classified elsewhere		
N29.0	Late syphilis of kidney		
N29.1	Other disorders of kidney and ureter in infectious and parasitic diseases classified elsewhere		
N30.0	Acute cystitis	5950A	59500
N34.0	Urethral abscess	5970A	
N39.0	Urinary tract infection, site not specified	5990B	59902
N41.0	Acute prostatitis	6010A	60100
N41.2	Abscess of prostate	6012A	
N45*	Orchitis and epididymitis		
N45.0	Orchitis, epididymitis and epididymo-orchitis with abscess	6040A	
N45.9	Orchitis, epididymitis and epididymo-orchitis without abscess	6049A	60400
N45.9	Orchitis, epididymitis and epididymo-orchitis without abscess	6049X	60401
N45.9	Orchitis, epididymitis and epididymo-orchitis without abscess		60402
N45.9	Orchitis, epididymitis and epididymo-orchitis without abscess		60403
N61	Inflammatory disorders of breast	6110A	61100
N61	Inflammatory disorders of breast		61101
N70.0	Acute salpingitis and oophoritis	6140	61201
N70.0	Acute salpingitis and oophoritis		61202
N70.0	Acute salpingitis and oophoritis		61207

N70.0	Acute salpingitis and oophoritis		61208
N70.0	Acute salpingitis and oophoritis		61209
N71.0	Acute inflammatory disease of uterus	6150A	62200
N71.0	Acute inflammatory disease of uterus	6150B	
N73.0	Acute parametritis and pelvic cellulitis	6143A	61600
N73.0	Acute parametritis and pelvic cellulitis		61602
N73.3	Female acute pelvic peritonitis	6145A	
N74	Female pelvic inflammatory disorders in diseases classified elsewhere		
N74.0	Tuberculous infection of cervix uteri		
N74.1	Female tuberculous pelvic inflammatory disease		
N74.2	Female syphilitic pelvic inflammatory disease		
N74.3	Female gonococcal pelvic inflammatory disease		
N74.4	Female chlamydial pelvic inflammatory disease		
N74.8	Female pelvic inflammatory disorders in other diseases classified elsewhere		
N75.1	Abscess of Bartholin gland	6163A	62212
N76.0	Acute vaginitis		
N76.4	Abscess of vulva	6164A	
N77.0	Ulceration of vulva in infectious and parasitic diseases classified elsewhere		
N77.1	Vaginitis, vulvitis and vulvovaginitis in infectious and parasitic diseases classified elsewhere		
O03.0	Spontaneous abortion: Incomplete, complicated by genital tract and pelvic infection		
O03.5	Spontaneous abortion: Complete or unspecified, complicated by genital tract and pelvic infection	6342A	64300
O03.5	Spontaneous abortion: Complete or unspecified, complicated by genital tract and pelvic infection		64320
O04.0	Medical abortion: Incomplete, complicated by genital tract and pelvic infection		
O04.5	Medical abortion: Complete or unspecified, complicated by genital tract and pelvic infection	6351A	
O04.5	Medical abortion: Complete or unspecified, complicated by genital tract and pelvic infection	6351B	
O04.5	Medical abortion: Complete or unspecified, complicated by genital tract and pelvic infection	6351C	
O04.5	Medical abortion: Complete or unspecified, complicated by genital tract and pelvic infection	6351D	
O04.5	Medical abortion: Complete or unspecified, complicated by genital tract and pelvic infection	6351X	
O05.0	Other abortion: Incomplete, complicated by genital tract and pelvic infection		
O05.5	Other abortion: Complete or unspecified, complicated by genital tract and pelvic infection	6361A	
O06.0	Unspecified abortion: Incomplete, complicated by genital tract and pelvic infection		
O06.5	Unspecified abortion: Complete or unspecified, complicated by genital tract and pelvic infection		64200
O06.5	Unspecified abortion: Complete or unspecified, complicated by genital tract and pelvic infection		64220
O06.5	Unspecified abortion: Complete or unspecified, complicated by genital tract and pelvic infection		64299
O06.5	Unspecified abortion: Complete or unspecified, complicated by genital tract and pelvic infection		64400
O06.5	Unspecified abortion: Complete or unspecified, complicated by genital tract and pelvic infection		64420
O07.0	Failed medical abortion, complicated by genital tract and pelvic infection		
O07.5	Other and unspecified failed attempted abortion, complicated by genital tract and pelvic infection	6381A	
O08.0	Genital tract and pelvic infection following abortion and ectopic and molar pregnancy	6391A	64590
O08.0	Genital tract and pelvic infection following abortion and ectopic and molar pregnancy		63100
O08.0	Genital tract and pelvic infection following abortion and ectopic and molar pregnancy		63101
O08.0	Genital tract and pelvic infection following abortion and ectopic and molar pregnancy		63110
O08.0	Genital tract and pelvic infection following abortion and ectopic and molar pregnancy		63120
O08.0	Genital tract and pelvic infection following abortion and ectopic and molar pregnancy		63130
O08.0	Genital tract and pelvic infection following abortion and ectopic and molar pregnancy		64591
O23.0	Infections of kidney in pregnancy		63500
O23.0	Infections of kidney in pregnancy		63501
O23.1	Infections of bladder in pregnancy		63590
O23.2	Infections of urethra in pregnancy		
O23.3	Infections of other parts of urinary tract in pregnancy		

O23.4	Unspecified infection of urinary tract in pregnancy		63599
O23.5	Infections of the genital tract in pregnancy		63000
O23.5	Infections of the genital tract in pregnancy		63001
O23.5	Infections of the genital tract in pregnancy		63002
O23.5	Infections of the genital tract in pregnancy		63003
O23.5	Infections of the genital tract in pregnancy		63008
O23.5	Infections of the genital tract in pregnancy		63009
O23.9	Other and unspecified genitourinary tract infection in pregnancy	6466A	
O75.3	Other infection during labour		
O85	Puerperal sepsis	670	670
O86*	Other puerperal infections		
O91.0	Infection of nipple associated with childbirth	6750A	
O91.1	Abscess of breast associated with childbirth	6751A	67802
O98	Maternal infectious and parasitic diseases classifiable elsewhere but complicating pregnancy, childbirth and the puerperium		
O98.0	Tuberculosis complicating pregnancy, childbirth and the puerperium	6473A	
O98.1	Syphilis complicating pregnancy, childbirth and the puerperium	6470A	
O98.2	Gonorrhoea complicating pregnancy, childbirth and the puerperium	6471A	
O98.3	Other infections with a predominantly sexual mode of transmission complicating pregnancy, childbirth and the puerperium	6472X	
O98.4	Viral hepatitis complicating pregnancy, childbirth and the puerperium		
O98.5	Other viral diseases complicating pregnancy, childbirth and the puerperium	6475A	
O98.5	Other viral diseases complicating pregnancy, childbirth and the puerperium	6476A	
O98.6	Protozoal diseases complicating pregnancy, childbirth and the puerperium		
O98.7	Human immunodeficiency virus [HIV] disease complicating pregnancy, childbirth and the puerperium		
O98.8	Other maternal infectious and parasitic diseases complicating pregnancy, childbirth and the puerperium	6474A	
O98.8	Other maternal infectious and parasitic diseases complicating pregnancy, childbirth and the puerperium	6478X	
O98.9	Unspecified maternal infectious or parasitic disease complicating pregnancy, childbirth and the puerperium	6479X	
P23	Congenital pneumonia	7700A	
P23.0	Congenital pneumonia due to viral agent		
P23.1	Congenital pneumonia due to Chlamydia		
P23.2	Congenital pneumonia due to staphylococcus		
P23.3	Congenital pneumonia due to streptococcus, group B		
P23.4	Congenital pneumonia due to Escherichia coli		
P23.5	Congenital pneumonia due to Pseudomonas		
P23.6	Congenital pneumonia due to other bacterial agents		
P23.8	Congenital pneumonia due to other organisms		
P23.9	Congenital pneumonia, unspecified		
P35.0	Congenital rubella syndrome	7710A	
P35.1	Congenital cytomegalovirus infection	7711A	07950
P35.2	Congenital herpesviral [herpes simplex] infection	7712A	
P35.3	Congenital viral hepatitis		
P36	Bacterial sepsis of newborn		
P36.0	Sepsis of newborn due to streptococcus, group B		
P36.1	Sepsis of newborn due to other and unspecified streptococci		
P36.2	Sepsis of newborn due to Staphylococcus aureus		
P36.3	Sepsis of newborn due to other and unspecified staphylococci		
P36.4	Sepsis of newborn due to Escherichia coli		
P36.5	Sepsis of newborn due to anaerobes		
P36.8	Other bacterial sepsis of newborn		

P36.9	Bacterial sepsis of newborn, unspecified	7718A	
P37	Other congenital infectious and parasitic diseases		
P37.0	Congenital tuberculosis		
P37.1	Congenital toxoplasmosis	7712C	13010
P37.1	Congenital toxoplasmosis		13020
P37.2	Neonatal (disseminated) listeriosis	7712B	02700
P37.3	Congenital falciparum malaria		
P37.4	Other congenital malaria		
P37.5	Neonatal candidiasis	7717A	
P37.8	Other specified congenital infectious and parasitic diseases		
P37.9	Congenital infectious and parasitic disease, unspecified	7712X	
P38	Omphalitis of newborn with or without mild haemorrhage	7714A	
P39	Other infections specific to the perinatal period		
P39.0	Neonatal infective mastitis	7715A	77890
P39.1	Neonatal conjunctivitis and dacryocystitis	7716A	
P39.2	Intra-amniotic infection of fetus, not elsewhere classified		
P39.3	Neonatal urinary tract infection	7718B	
P39.4	Neonatal skin infection		
P39.8	Other specified infections specific to the perinatal period	7718C	
P39.9	Infection specific to the perinatal period, unspecified	7718X	
R57.2	Septic shock	7855B	
R65.0	Systemic Inflammatory Response Syndrome of infectious origin without organ failure		
R65.1	Systemic Inflammatory Response Syndrome of infectious origin with organ failure		
Z21	Asymptomatic human immunodeficiency virus [HIV] infection status		

The diagnosis codes are from the Finnish national editions of the International Classification of Diseases, 8th, 9th, and 10th Revisions (ICD-8, ICD-9, and ICD-10)[2–4] and from the UK Biobank showcase (https://biobank.ndph.ox.ac.uk/showcase/field.cgi?id=41271).
ICD-10 disease names are from: World Health Organization. International Statistical Classification of Diseases and Related Health Problems 10th Revision [Internet]. ICD-10 Version:2016. [cited 2 Oct 2018]. Available from: https://icd.who.int/browse10/2016/en

eTable 7. Distribution of all infections in primary analysis (Finnish multicohort sample)

ICD-10 code and description	N	%
K35 Acute appendicitis	11 865	15.39
J18.9 Pneumonia, unspecified	6062	7.86
A09 Other gastroenteritis and colitis..	5608	7.27
N10 Acute tubulo-interstitial nephritis	3599	4.67
J03.9 Acute tonsillitis, unspecified	3084	4.00
A46 Erysipelas	3082	4.00
J36 Peritonsillar abscess	3035	3.94
J15.9 Bacterial pneumonia, unspecified	2228	2.89
N70.0 Acute salpingitis and oophoritis	1957	2.54
O85 Puerperal sepsis	1731	2.24
J20.9 Acute bronchitis, unspecified	1149	1.49
N39.0 Urinary tract infection, site n..	1055	1.37
J01.0 Acute maxillary sinusitis	1038	1.35
A49.9 Bacterial infection, unspecified	1026	1.33
J03.0 Streptococcal tonsillitis	919	1.19
A63.0 Anogenital (venereal) warts	843	1.09
A08.4 Viral intestinal infection, uns..	817	1.06
J22 Unspecified acute lower respirato..	777	1.01
N71.0 Acute inflammatory disease of u..	719	0.93
J02.9 Acute pharyngitis, unspecified	695	0.90
J04.0 Acute laryngitis	677	0.88
B34.9 Viral infection, unspecified	619	0.80
A98.5 Haemorrhagic fever with renal s..	577	0.75
A08.5 Other specified intestinal infe..	487	0.63
K57.2 Diverticular disease of large i..	456	0.59
J18.0 Bronchopneumonia, unspecified	441	0.57
N30.0 Acute cystitis	440	0.57
B27.0 Gammaherpesviral mononucleosis	415	0.54
A41.5 Sepsis due to other Gram-negati..	409	0.53
L05.9 Pilonidal cyst without abscess	389	0.50
O08.0 Genital tract and pelvic infect..	375	0.49
O03.5 Spontaneous abortion: Complete ..	365	0.47
B27.9 Infectious mononucleosis, unspe..	362	0.47
K61.0 Anal abscess	330	0.43
K05.3 Chronic periodontitis	327	0.42
A04.7 Enterocolitis due to Clostridiu..	306	0.40
N75.1 Abscess of Bartholin gland	297	0.39
H66.0 Acute suppurative otitis media	290	0.38
J11.1 Influenza with other respirator..	286	0.37
A41.9 Sepsis, unspecified	278	0.36
B06.9 Rubella without complication	277	0.36
J13 Pneumonia due to Streptococcus pn..	273	0.35
K04.7 Periapical abscess without sinus	264	0.34
B01.9 Varicella without complication	255	0.33
J11.8 Influenza with other manifestat..	255	0.33

N61 Inflammatory disorders of breast	253	0.33
A69.2 Lyme disease	229	0.30
L04 Acute lymphadenitis	225	0.29
M00.9 Pyogenic arthritis, unspecified	213	0.28
N45.9 Orchitis, epididymitis and epid..	209	0.27
J10.1 Influenza with other respirator..	206	0.27
B99 Other and unspecified infectious ..	199	0.26
L03.0 Cellulitis of finger and toe	196	0.25
A04.5 Campylobacter enteritis	194	0.25
K04.5 Chronic apical periodontitis	192	0.25
A87.9 Viral meningitis, unspecified	181	0.23
K65.0 Acute peritonitis	180	0.23
A60.0 Herpesviral infection of genita..	177	0.23
K61 Abscess of anal and rectal regions	175	0.23
O23.0 Infections of kidney in pregnancy	175	0.23
O23.5 Infections of the genital tract..	175	0.23
K12.2 Cellulitis and abscess of mouth	174	0.23
B18.2 Chronic viral hepatitis C	173	0.22
J05.1 Acute epiglottitis	169	0.22
O03.0 Spontaneous abortion: Incomplet..	168	0.22
A41.0 Sepsis due to Staphylococcus au..	165	0.21
L02 Cutaneous abscess, furuncle and c..	165	0.21
K02.1 Caries of dentine	163	0.21
O86.0 Infection of obstetric surgical..	162	0.21
L08.9 Local infection of skin and sub..	158	0.20
O23.9 Other and unspecified genitouri..	158	0.20
A02.0 Salmonella enteritis	153	0.20
J15.8 Other bacterial pneumonia	147	0.19
L02.4 Cutaneous abscess, furuncle and..	146	0.19
B19.9 Unspecified viral hepatitis wit..	142	0.18
A41.8 Other specified sepsis	140	0.18
J12 Viral pneumonia, not elsewhere cl..	140	0.18
A16.2 Tuberculosis of lung, without m..	135	0.18
A89 Unspecified viral infection of ce..	134	0.17
B07 Viral warts	134	0.17
O86.1 Other infection of genital trac..	130	0.17
A87.0 Enteroviral meningitis	129	0.17
B86 Scabies	125	0.16
G00.9 Bacterial meningitis, unspecified	124	0.16
L05.0 Pilonidal cyst with abscess	123	0.16
O86.4 Pyrexia of unknown origin follo..	120	0.16
K05.2 Acute periodontitis	115	0.15
J18.1 Lobar pneumonia, unspecified	112	0.15
A40.3 Sepsis due to Streptococcus pne..	111	0.14
A02 Other salmonella infections	109	0.14
K02 Dental caries	104	0.13
A86 Unspecified viral encephalitis	103	0.13
B35 Dermatophytosis	101	0.13

B02.9 Zoster without complication	100	0.13
B97.8 Other viral agents as the cause..	100	0.13
L04.0 Acute lymphadenitis of face, he..	100	0.13
A56.1 Chlamydial infection of pelvipe..	99	0.13
L03.1 Cellulitis of other parts of limb	97	0.13
J15.7 Pneumonia due to Mycoplasma pne..	92	0.12
O23.4 Unspecified infection of urinar..	92	0.12
O04.0 Medical abortion: Incomplete, c..	89	0.12
L02.2 Cutaneous abscess, furuncle and..	88	0.11
B05.9 Measles without complication	86	0.11
A49.8 Other bacterial infections of u..	85	0.11
O23.1 Infections of bladder in pregna..	84	0.11
A04.9 Bacterial intestinal infection,..	81	0.11
H00.1 Chalazion	79	0.10
L02.3 Cutaneous abscess, furuncle and..	79	0.10
B00.1 Herpesviral vesicular dermatitis	78	0.10
B08.2 Exanthema subitum [sixth disease]	78	0.10
H10.0 Mucopurulent conjunctivitis	78	0.10
J86.9 Pyothorax without fistula	78	0.10
A15.0 Tuberculosis of lung, confirmed..	77	0.10
A95 Yellow fever	76	0.10
B34.8 Other viral infections of unspe..	76	0.10
J16.8 Pneumonia due to other specifie..	76	0.10
M71.1 Other infective bursitis	75	0.10
O86.2 Urinary tract infection followi..	74	0.10
A54.9 Gonococcal infection, unspecified	73	0.09
L03.9 Cellulitis, unspecified	73	0.09
I33.0 Acute and subacute infective en..	71	0.09
N41.0 Acute prostatitis	71	0.09
A08.3 Other viral enteritis	70	0.09
H60.3 Other infective otitis externa	70	0.09
L08.8 Other specified local infection..	70	0.09
M86.9 Osteomyelitis, unspecified	68	0.09
N76.4 Abscess of vulva	68	0.09
B26.2 Mumps encephalitis	67	0.09
G03.9 Meningitis, unspecified	67	0.09
J39.0 Retropharyngeal and parapharyng..	66	0.09
K57.0 Diverticular disease of small i..	65	0.08
O04.5 Medical abortion: Complete or u..	65	0.08
K04.6 Periapical abscess with sinus	63	0.08
J09 Influenza due to certain identifi..	62	0.08
M86.1 Other acute osteomyelitis	62	0.08
B00.2 Herpesviral gingivostomatitis a..	61	0.08
B37 Candidiasis	61	0.08
O75.3 Other infection during labour	61	0.08
O86.8 Other specified puerperal infec..	60	0.08
O98.8 Other maternal infectious and p..	60	0.08
A04.8 Other specified bacterial intes..	58	0.08

J10.0 Influenza with pneumonia, other..	58	0.08
N77.1 Vaginitis, vulvitis and vulvova..	57	0.07
H62.0 Otitis externa in bacterial dis..	56	0.07
A54.2 Gonococcal pelviperitonitis and..	55	0.07
A74.9 Chlamydial infection, unspecified	54	0.07
B09 Unspecified viral infection chara..	54	0.07
L02.1 Cutaneous abscess, furuncle and..	54	0.07
B00.0 Eczema herpeticum	53	0.07
L02.9 Cutaneous abscess, furuncle and..	52	0.07
O91.1 Abscess of breast associated wi..	50	0.06
A38 Scarlet fever	48	0.06
N73.3 Female acute pelvic peritonitis	47	0.06
H00.0 Hordeolum and other deep inflam..	46	0.06
A40.8 Other streptococcal sepsis	45	0.06
H05.0 Acute inflammation of orbit	44	0.06
O98.5 Other viral diseases complicati..	43	0.06
A56.0 Chlamydial infection of lower g..	42	0.05
B08.1 Molluscum contagiosum	41	0.05
A18.2 Tuberculous peripheral lymphade..	40	0.05
G00.8 Other bacterial meningitis	40	0.05
J11.0 Influenza with pneumonia, virus..	40	0.05
J20.0 Acute bronchitis due to Mycopla..	40	0.05
A40.9 Streptococcal sepsis, unspecified	39	0.05
B15.9 Hepatitis A without hepatic coma	39	0.05
A40.0 Sepsis due to streptococcus, gr..	38	0.05
I40.0 Infective myocarditis	38	0.05
J02.0 Streptococcal pharyngitis	38	0.05
L02.0 Cutaneous abscess, furuncle and..	38	0.05
A08.0 Rotaviral enteritis	37	0.05
J18.8 Other pneumonia, organism unspe..	37	0.05
K61.1 Rectal abscess	37	0.05
L03.2 Cellulitis of face	37	0.05
O98.3 Other infections with a predomi..	37	0.05
A41.1 Sepsis due to other specified s..	36	0.05
J12.9 Viral pneumonia, unspecified	36	0.05
L08.0 Pyoderma	36	0.05
A49.0 Staphylococcal infection, unspe..	35	0.05
B02.8 Zoster with other complications	35	0.05
B85 Pediculosis and phthiriasis	35	0.05
J04.2 Acute laryngotracheitis	35	0.05
L02.8 Cutaneous abscess, furuncle and..	35	0.05
B00.5 Herpesviral ocular disease	34	0.04
B97.7 Papillomavirus as the cause of ..	34	0.04
K02.9 Dental caries, unspecified	34	0.04
B01.1 Varicella encephalitis	33	0.04
B33.8 Other specified viral diseases	33	0.04
M71.0 Abscess of bursa	33	0.04
A04.6 Enteritis due to Yersinia enter..	32	0.04

B01.8 Varicella with other complicati..	31	0.04
H70.0 Acute mastoiditis	31	0.04
M86.6 Other chronic osteomyelitis	31	0.04
A02.1 Salmonella sepsis	30	0.04
A16.5 Tuberculous pleurisy, without m..	30	0.04
H60.1 Cellulitis of external ear	30	0.04
A39.0 Meningococcal meningitis	29	0.04
A59.0 Urogenital trichomoniasis	29	0.04
G00.0 Haemophilus meningitis	29	0.04
G05.1 Encephalitis, myelitis and ence..	29	0.04
H60.0 Abscess of external ear	28	0.04
N73.0 Acute parametritis and pelvic c..	28	0.04
J85.2 Abscess of lung without pneumonia	27	0.04
A37 Whooping cough	26	0.03
B16.9 Acute hepatitis B without delta..	26	0.03
B18.1 Chronic viral hepatitis B witho..	26	0.03
J10.8 Influenza with other manifestat..	26	0.03
A05.9 Bacterial foodborne intoxicatio..	25	0.03
A41.2 Sepsis due to unspecified staph..	25	0.03
J86.0 Pyothorax with fistula	24	0.03
K63.0 Abscess of intestine	24	0.03
K75.0 Abscess of liver	23	0.03
M00.0 Staphylococcal arthritis and po..	23	0.03
H44.0 Purulent endophthalmitis	22	0.03
I30.1 Infective pericarditis	22	0.03
J21.9 Acute bronchiolitis, unspecified	22	0.03
O86.3 Other genitourinary tract infec..	22	0.03
O98.9 Unspecified maternal infectious..	22	0.03
B02.3 Zoster ocular disease	21	0.03
K11.3 Abscess of salivary gland	21	0.03
M65.0 Abscess of tendon sheath	21	0.03
A18.0 Tuberculosis of bones and joints	20	0.03
A21.9 Tularaemia, unspecified	20	0.03
A54.1 Gonococcal infection of lower g..	20	0.03
B34.0 Adenovirus infection, unspecifi..	20	0.03
G02.0 Meningitis in viral diseases cl..	20	0.03
G06.0 Intracranial abscess and granul..	20	0.03
K61.2 Anorectal abscess	20	0.03
L03.3 Cellulitis of trunk	20	0.03
A21.0 Ulceroglandular tularaemia	19	0.02
B50.9 Plasmodium falciparum malaria, ..	19	0.02
G00.1 Pneumococcal meningitis	19	0.02
N76.0 Acute vaginitis	19	0.02
A80 Acute poliomyelitis	18	0.02
J04.1 Acute tracheitis	18	0.02
J17.3 Pneumonia in parasitic diseases	18	0.02
K04.4 Acute apical periodontitis of p..	18	0.02
B01 Varicella [chickenpox]	17	0.02

B25.8 Other cytomegaloviral diseases	17	0.02
B25.9 Cytomegaloviral disease, unspec..	17	0.02
B26.9 Mumps without complication	17	0.02
G01 Meningitis in bacterial diseases ..	17	0.02
I42.1 Obstructive hypertrophic cardio..	17	0.02
N15.1 Renal and perinephric abscess	17	0.02
N74.4 Female chlamydial pelvic inflam..	17	0.02
A07 Other protozoal intestinal diseases	16	0.02
A41.4 Sepsis due to anaerobes	16	0.02
A48.8 Other specified bacterial disea..	16	0.02
A88.8 Other specified viral infection..	16	0.02
B17.1 Acute hepatitis C	16	0.02
B23.1 HIV disease resulting in (persi..	16	0.02
B37.3 Candidiasis of vulva and vagina	16	0.02
J86 Pyothorax	16	0.02
O98.4 Viral hepatitis complicating pr..	16	0.02
A15.1 Tuberculosis of lung, confirmed..	15	0.02
A40.1 Sepsis due to streptococcus, gr..	15	0.02
B27.1 Cytomegaloviral mononucleosis	15	0.02
B37.7 Candidal sepsis	15	0.02
B58.9 Toxoplasmosis, unspecified	15	0.02
L01.0 Impetigo [any organism] [any si..	15	0.02
M46.3 Infection of intervertebral dis..	15	0.02
N45.0 Orchitis, epididymitis and epid..	15	0.02
A51.0 Primary genital syphilis	14	0.02
B37.8 Candidiasis of other sites	14	0.02
K57.4 Diverticular disease of both sm..	14	0.02
O91.0 Infection of nipple associated ..	14	0.02
A02.9 Salmonella infection, unspecified	13	0.02
A03.3 Shigellosis due to Shigella son..	13	0.02
A51.3 Secondary syphilis of skin and ..	13	0.02
B35.1 Tinea unguium	13	0.02
B59 Pneumocystosis	13	0.02
B97.0 Adenovirus as the cause of dise..	13	0.02
J03.8 Acute tonsillitis due to other ..	13	0.02
L03.8 Cellulitis of other sites	13	0.02
N34.0 Urethral abscess	13	0.02
B00.8 Other forms of herpesviral infe..	12	0.02
B00.9 Herpesviral infection, unspecif..	12	0.02
B24 Unspecified human immunodeficienc..	12	0.02
B30 Viral conjunctivitis	12	0.02
B37.0 Candidal stomatitis	12	0.02
B70.0 Diphyllobothriasis	12	0.02
J85.1 Abscess of lung with pneumonia	12	0.02
L70.1 Acne conglobata	12	0.02
M60.0 Infective myositis	12	0.02
O06.5 Unspecified abortion: Complete ..	12	0.02
A01.4 Paratyphoid fever, unspecified	11	0.01

A64 Unspecified sexually transmitted ..	11	0.01
B06 Rubella [German measles]	11	0.01
B26.0 Mumps orchitis	11	0.01
B34.1 Enterovirus infection, unspecif..	11	0.01
G06.1 Intraspinal abscess and granuloma	11	0.01
L01 Impetigo	11	0.01
L04.9 Acute lymphadenitis, unspecified	11	0.01
A01.2 Paratyphoid fever B	10	0.01
A03.1 Shigellosis due to Shigella fle..	10	0.01
A39.2 Acute meningococcaemia	10	0.01
B54 Unspecified malaria	10	0.01
B58.0 Toxoplasma oculopathy	10	0.01
J14 Pneumonia due to Haemophilus infl..	10	0.01
J16.0 Chlamydial pneumonia	10	0.01
J20.5 Acute bronchitis due to respira..	10	0.01
K61.3 Ischiorectal abscess	10	0.01
L01.1 Impetiginization of other derma..	10	0.01
M46.2 Osteomyelitis of vertebra	10	0.01
N13.6 Pyonephrosis	10	0.01
A06.9 Amoebiasis, unspecified	9	0.01
A63.8 Other specified predominantly s..	9	0.01
A90 Dengue fever [classical dengue]	9	0.01
B00.7 Disseminated herpesviral disease	9	0.01
B08.8 Other specified viral infection..	9	0.01
B33.2 Viral carditis	9	0.01
K77.0 Liver disorders in infectious a..	9	0.01
M00.2 Other streptococcal arthritis a..	9	0.01
A02.2 Localized salmonella infections	8	0.01
A02.8 Other specified salmonella infe..	8	0.01
A15.6 Tuberculous pleurisy, confirmed..	8	0.01
A16.0 Tuberculosis of lung, bacteriol..	8	0.01
A16.9 Respiratory tuberculosis unspec..	8	0.01
A18.1 Tuberculosis of genitourinary s..	8	0.01
A32.8 Other forms of listeriosis	8	0.01
A39.4 Meningococcaemia, unspecified	8	0.01
A49.1 Streptococcal infection, unspec..	8	0.01
A87.8 Other viral meningitis	8	0.01
B00.4 Herpesviral encephalitis	8	0.01
B05.8 Measles with other complications	8	0.01
B26.3 Mumps pancreatitis	8	0.01
B53.0 Plasmodium ovale malaria	8	0.01
J20.8 Acute bronchitis due to other s..	8	0.01
J39.1 Other abscess of pharynx	8	0.01
M01.2 Arthritis in Lyme disease	8	0.01
O07.0 Failed medical abortion, compli..	8	0.01
O07.5 Other and unspecified failed at..	8	0.01
A01.0 Typhoid fever	7	0.01
A31.0 Pulmonary mycobacterial infection	7	0.01

A84.8 Other tick-borne viral encephal..	7	0.01
B02.0 Zoster encephalitis	7	0.01
B02.2 Zoster with other nervous syste..	7	0.01
B36 Other superficial mycoses	7	0.01
B80 Enterobiasis	7	0.01
J20.1 Acute bronchitis due to Haemoph..	7	0.01
K02.8 Other dental caries	7	0.01
N08.0 Glomerular disorders in infecti..	7	0.01
O05.0 Other abortion: Incomplete, com..	7	0.01
O05.5 Other abortion: Complete or uns..	7	0.01
Z21 Asymptomatic human immunodeficien..	7	0.01
A04.3 Enterohaemorrhagic Escherichia ..	6	0.01
A08.1 Acute gastroenteropathy due to ..	6	0.01
A08.2 Adenoviral enteritis	6	0.01
A18.3 Tuberculosis of intestines, per..	6	0.01
A48.0 Gas gangrene	6	0.01
A54.0 Gonococcal infection of lower g..	6	0.01
A74.8 Other chlamydial diseases	6	0.01
B02.1 Zoster meningitis	6	0.01
B08.3 Erythema infectiosum [fifth dis..	6	0.01
B18.9 Chronic viral hepatitis, unspec..	6	0.01
B25.1 Cytomegaloviral hepatitis	6	0.01
B35.3 Tinea pedis	6	0.01
B51.9 Plasmodium vivax malaria withou..	6	0.01
B67 Echinococcosis	6	0.01
H10.5 Blepharoconjunctivitis	6	0.01
J21.0 Acute bronchiolitis due to resp..	6	0.01
J85.3 Abscess of mediastinum	6	0.01
K05.0 Acute gingivitis	6	0.01
M65.1 Other infective (teno)synovitis	6	0.01
N77.0 Ulceration of vulva in infectio..	6	0.01
O06.0 Unspecified abortion: Incomplet..	6	0.01
A03.9 Shigellosis, unspecified	5	0.01
A06.0 Acute amoebic dysentery	5	0.01
A15.2 Tuberculosis of lung, confirmed..	5	0.01
A31 Infection due to other mycobacteria	5	0.01
A31.9 Mycobacterial infection, unspec..	5	0.01
A32.7 Listerial sepsis	5	0.01
A52.1 Symptomatic neurosyphilis	5	0.01
A60.9 Anogenital herpesviral infectio..	5	0.01
A84.9 Tick-borne viral encephalitis, ..	5	0.01
B01.2 Varicella pneumonia	5	0.01
B02.7 Disseminated zoster	5	0.01
B08.5 Enteroviral vesicular pharyngitis	5	0.01
B19.0 Unspecified viral hepatitis wit..	5	0.01
B37.4 Candidiasis of other urogenital..	5	0.01
B74 Filariasis	5	0.01
B77.9 Ascariasis, unspecified	5	0.01

B95.8 Unspecified staphylococcus as t..	5	0.01
B97.1 Enterovirus as the cause of dis..	5	0.01
G03.8 Meningitis due to other specifi..	5	0.01
H19.1 Herpesviral keratitis and kerat..	5	0.01
H19.2 Keratitis and keratoconjunctivi..	5	0.01
H62.1 Otitis externa in viral disease..	5	0.01
J12.1 Respiratory syncytial virus pne..	5	0.01
J15.2 Pneumonia due to staphylococcus	5	0.01
K57.8 Diverticular disease of intesti..	5	0.01
L04.2 Acute lymphadenitis of upper limb	5	0.01
L04.8 Acute lymphadenitis of other si..	5	0.01
M86.0 Acute haematogenous osteomyelitis	5	0.01
M86.2 Subacute osteomyelitis	5	0.01
A00.9 Cholera, unspecified	<5	<0.01
A01.1 Paratyphoid fever A	<5	<0.01
A01.3 Paratyphoid fever C	<5	<0.01
A03 Shigellosis	<5	<0.01
A03.0 Shigellosis due to Shigella dys..	<5	<0.01
A03.2 Shigellosis due to Shigella boy..	<5	<0.01
A04.0 Enteropathogenic Escherichia co..	<5	<0.01
A04.2 Enteroinvasive Escherichia coli..	<5	<0.01
A04.4 Other intestinal Escherichia co..	<5	<0.01
A05.0 Foodborne staphylococcal intoxi..	<5	<0.01
A05.1 Botulism	<5	<0.01
A05.2 Foodborne Clostridium perfringe..	<5	<0.01
A05.8 Other specified bacterial foodb..	<5	<0.01
A06.2 Amoebic nondysenteric colitis	<5	<0.01
A06.4 Amoebic liver abscess	<5	<0.01
A06.5 Amoebic lung abscess	<5	<0.01
A06.8 Amoebic infection of other sites	<5	<0.01
A07.0 Balantidiasis	<5	<0.01
A07.1 Giardiasis [lambliasis]	<5	<0.01
A07.2 Cryptosporidiosis	<5	<0.01
A07.3 Isosporiasis	<5	<0.01
A07.9 Protozoal intestinal disease, u..	<5	<0.01
A15.3 Tuberculosis of lung, confirmed..	<5	<0.01
A15.4 Tuberculosis of intrathoracic l..	<5	<0.01
A15.5 Tuberculosis of larynx, trachea..	<5	<0.01
A15.7 Primary respiratory tuberculosi..	<5	<0.01
A15.8 Other respiratory tuberculosis,..	<5	<0.01
A15.9 Respiratory tuberculosis unspec..	<5	<0.01
A16.1 Tuberculosis of lung, bacteriol..	<5	<0.01
A16.3 Tuberculosis of intrathoracic l..	<5	<0.01
A16.4 Tuberculosis of larynx, trachea..	<5	<0.01
A16.7 Primary respiratory tuberculosi..	<5	<0.01
A16.8 Other respiratory tuberculosis,..	<5	<0.01
A17.0 Tuberculous meningitis	<5	<0.01
A17.9 Tuberculosis of nervous system,..	<5	<0.01

A18.4 Tuberculosis of skin and subcut..	<5	<0.01
A18.8 Tuberculosis of other specified..	<5	<0.01
A19.0 Acute miliary tuberculosis of a..	<5	<0.01
A19.2 Acute miliary tuberculosis, uns..	<5	<0.01
A19.9 Miliary tuberculosis, unspecified	<5	<0.01
A20.9 Plague, unspecified	<5	<0.01
A21.1 Oculoglandular tularaemia	<5	<0.01
A21.2 Pulmonary tularaemia	<5	<0.01
A21.7 Generalized tularaemia	<5	<0.01
A21.8 Other forms of tularaemia	<5	<0.01
A22.0 Cutaneous anthrax	<5	<0.01
A22.2 Gastrointestinal anthrax	<5	<0.01
A23.9 Brucellosis, unspecified	<5	<0.01
A24.0 Glanders	<5	<0.01
A26.0 Cutaneous erysipeloid	<5	<0.01
A26.9 Erysipeloid, unspecified	<5	<0.01
A27 Leptospirosis	<5	<0.01
A27.0 Leptospirosis icterohaemorrhagica	<5	<0.01
A27.8 Other forms of leptospirosis	<5	<0.01
A28.2 Extraintestinal yersiniosis	<5	<0.01
A28.8 Other specified zoonotic bacter..	<5	<0.01
A28.9 Zoonotic bacterial disease, uns..	<5	<0.01
A30 Leprosy [Hansen disease]	<5	<0.01
A31.1 Cutaneous mycobacterial infection	<5	<0.01
A31.8 Other mycobacterial infections	<5	<0.01
A32.9 Listeriosis, unspecified	<5	<0.01
A35 Other tetanus	<5	<0.01
A36 Diphtheria	<5	<0.01
A37.0 Whooping cough due to Bordetell..	<5	<0.01
A37.9 Whooping cough, unspecified	<5	<0.01
A39 Meningococcal infection	<5	<0.01
A39.8 Other meningococcal infections	<5	<0.01
A39.9 Meningococcal infection, unspec..	<5	<0.01
A40 Streptococcal sepsis	<5	<0.01
A40.2 Sepsis due to streptococcus, gr..	<5	<0.01
A41 Other sepsis	<5	<0.01
A41.3 Sepsis due to Haemophilus influ..	<5	<0.01
A42 Actinomycosis	<5	<0.01
A42.0 Pulmonary actinomycosis	<5	<0.01
A42.1 Abdominal actinomycosis	<5	<0.01
A42.2 Cervicofacial actinomycosis	<5	<0.01
A42.7 Actinomycotic sepsis	<5	<0.01
A42.8 Other forms of actinomycosis	<5	<0.01
A42.9 Actinomycosis, unspecified	<5	<0.01
A44.1 Cutaneous and mucocutaneous bar..	<5	<0.01
A49.2 Haemophilus influenzae infectio..	<5	<0.01
A49.3 Mycoplasma infection, unspecifi..	<5	<0.01
A50 Congenital syphilis	<5	<0.01

A50.5 Other late congenital syphilis,..	<5	<0.01
A50.9 Congenital syphilis, unspecified	<5	<0.01
A51.2 Primary syphilis of other sites	<5	<0.01
A51.4 Other secondary syphilis	<5	<0.01
A51.5 Early syphilis, latent	<5	<0.01
A51.9 Early syphilis, unspecified	<5	<0.01
A52.0 Cardiovascular syphilis	<5	<0.01
A52.7 Other symptomatic late syphilis	<5	<0.01
A52.8 Late syphilis, latent	<5	<0.01
A53 Other and unspecified syphilis	<5	<0.01
A53.9 Syphilis, unspecified	<5	<0.01
A54 Gonococcal infection	<5	<0.01
A54.3 Gonococcal infection of eye	<5	<0.01
A54.4 Gonococcal infection of musculo..	<5	<0.01
A54.8 Other gonococcal infections	<5	<0.01
A55 Chlamydial lymphogranuloma (vener..	<5	<0.01
A56.2 Chlamydial infection of genitou..	<5	<0.01
A56.3 Chlamydial infection of anus an..	<5	<0.01
A56.8 Sexually transmitted chlamydial..	<5	<0.01
A58 Granuloma inguinale	<5	<0.01
A60.1 Herpesviral infection of perian..	<5	<0.01
A66.1 Multiple papillomata and wet cr..	<5	<0.01
A66.3 Hyperkeratosis of yaws	<5	<0.01
A67 Pinta [carate]	<5	<0.01
A67.1 Intermediate lesions of pinta	<5	<0.01
A68 Relapsing fevers	<5	<0.01
A69.0 Necrotizing ulcerative stomatitis	<5	<0.01
A69.1 Other Vincent infections	<5	<0.01
A69.9 Spirochaetal infection, unspeci..	<5	<0.01
A71 Trachoma	<5	<0.01
A75.0 Epidemic louse-borne typhus fev..	<5	<0.01
A75.9 Typhus fever, unspecified	<5	<0.01
A77.1 Spotted fever due to Rickettsia..	<5	<0.01
A78 Q fever	<5	<0.01
A79.9 Rickettsiosis, unspecified	<5	<0.01
A80.1 Acute paralytic poliomyelitis, ..	<5	<0.01
A80.3 Acute paralytic poliomyelitis, ..	<5	<0.01
A81.2 Progressive multifocal leukoenc..	<5	<0.01
A83.0 Japanese encephalitis	<5	<0.01
A83.6 Rocio virus disease	<5	<0.01
A83.8 Other mosquito-borne viral ence..	<5	<0.01
A84 Tick-borne viral encephalitis	<5	<0.01
A84.1 Central European tick-borne enc..	<5	<0.01
A85.0 Enteroviral encephalitis	<5	<0.01
A85.1 Adenoviral encephalitis	<5	<0.01
A85.2 Arthropod-borne viral encephali..	<5	<0.01
A85.8 Other specified viral encephali..	<5	<0.01
A87.1 Adenoviral meningitis	<5	<0.01

A87.2 Lymphocytic choriomeningitis	<5	<0.01
A88.1 Epidemic vertigo	<5	<0.01
A92.8 Other specified mosquito-borne ..	<5	<0.01
A96.0 Junin haemorrhagic fever	<5	<0.01
A98.0 Crimean-Congo haemorrhagic fever	<5	<0.01
A98.1 Omsk haemorrhagic fever	<5	<0.01
A98.3 Marburg virus disease	<5	<0.01
A98.4 Ebola virus disease	<5	<0.01
A99 Unspecified viral haemorrhagic fe..	<5	<0.01
B00.3 Herpesviral meningitis	<5	<0.01
B02 Zoster [herpes zoster]	<5	<0.01
B05 Measles	<5	<0.01
B05.3 Measles complicated by otitis m..	<5	<0.01
B06.8 Rubella with other complications	<5	<0.01
B08.0 Other orthopoxvirus infections	<5	<0.01
B08.4 Enteroviral vesicular stomatiti..	<5	<0.01
B15.0 Hepatitis A with hepatic coma	<5	<0.01
B16.1 Acute hepatitis B with delta-ag..	<5	<0.01
B17.2 Acute hepatitis E	<5	<0.01
B17.8 Other specified acute viral hep..	<5	<0.01
B18.0 Chronic viral hepatitis B with ..	<5	<0.01
B20.0 HIV disease resulting in mycoba..	<5	<0.01
B20.6 HIV disease resulting in Pneumo..	<5	<0.01
B21.2 HIV disease resulting in other ..	<5	<0.01
B23.8 HIV disease resulting in other ..	<5	<0.01
B25.0 Cytomegaloviral pneumonitis	<5	<0.01
B26 Mumps	<5	<0.01
B26.1 Mumps meningitis	<5	<0.01
B26.8 Mumps with other complications	<5	<0.01
B27.8 Other infectious mononucleosis	<5	<0.01
B30.8 Other viral conjunctivitis	<5	<0.01
B33.0 Epidemic myalgia	<5	<0.01
B34.3 Parvovirus infection, unspecifi..	<5	<0.01
B35.0 Tinea barbae and tinea capitis	<5	<0.01
B35.2 Tinea manuum	<5	<0.01
B35.4 Tinea corporis	<5	<0.01
B35.6 Tinea cruris	<5	<0.01
B35.9 Dermatophytosis, unspecified	<5	<0.01
B36.0 Pityriasis versicolor	<5	<0.01
B36.9 Superficial mycosis, unspecified	<5	<0.01
B37.2 Candidiasis of skin and nail	<5	<0.01
B37.9 Candidiasis, unspecified	<5	<0.01
B38 Coccidioidomycosis	<5	<0.01
B38.0 Acute pulmonary coccidioidomyco..	<5	<0.01
B39.3 Disseminated histoplasmosis cap..	<5	<0.01
B39.4 Histoplasmosis capsulati, unspe..	<5	<0.01
B41.0 Pulmonary paracoccidioidomycosis	<5	<0.01
B43.9 Chromomycosis, unspecified	<5	<0.01

B44.9 Aspergillosis, unspecified	<5	<0.01
B47.9 Mycetoma, unspecified	<5	<0.01
B48.1 Rhinosporidiosis	<5	<0.01
B49 Unspecified mycosis	<5	<0.01
B50.0 Plasmodium falciparum malaria w..	<5	<0.01
B50.8 Other severe and complicated Pl..	<5	<0.01
B51 Plasmodium vivax malaria	<5	<0.01
B51.0 Plasmodium vivax malaria with r..	<5	<0.01
B52.0 Plasmodium malariae malaria wit..	<5	<0.01
B52.9 Plasmodium malariae malaria wit..	<5	<0.01
B55.2 Mucocutaneous leishmaniasis	<5	<0.01
B58.8 Toxoplasmosis with other organ ..	<5	<0.01
B60.8 Other specified protozoal disea..	<5	<0.01
B65.0 Schistosomiasis due to Schistos..	<5	<0.01
B65.1 Schistosomiasis due to Schistos..	<5	<0.01
B65.2 Schistosomiasis due to Schistos..	<5	<0.01
B65.9 Schistosomiasis, unspecified	<5	<0.01
B66 Other fluke infections	<5	<0.01
B67.3 Echinococcus granulosus infecti..	<5	<0.01
B67.8 Echinococcosis, unspecified, of..	<5	<0.01
B67.9 Echinococcosis, other and unspe..	<5	<0.01
B68.0 Taenia solium taeniasis	<5	<0.01
B68.1 Taenia saginata taeniasis	<5	<0.01
B68.9 Taeniasis, unspecified	<5	<0.01
B69.0 Cysticercosis of central nervou..	<5	<0.01
B71.0 Hymenolepiasis	<5	<0.01
B75 Trichinellosis	<5	<0.01
B76 Hookworm diseases	<5	<0.01
B78.0 Intestinal strongyloidiasis	<5	<0.01
B79 Trichuriasis	<5	<0.01
B81.0 Anisakiasis	<5	<0.01
B81.4 Mixed intestinal helminthiases	<5	<0.01
B82.9 Intestinal parasitism, unspecif..	<5	<0.01
B83.0 Visceral larva migrans	<5	<0.01
B83.9 Helminthiasis, unspecified	<5	<0.01
B85.1 Pediculosis due to Pediculus hu..	<5	<0.01
B87.1 Wound myiasis	<5	<0.01
B87.9 Myiasis, unspecified	<5	<0.01
B95.0 Streptococcus, group A, as the ..	<5	<0.01
B95.1 Streptococcus, group B, as the ..	<5	<0.01
B95.3 Streptococcus pneumoniae as the..	<5	<0.01
B95.4 Other streptococcus as the caus..	<5	<0.01
B95.5 Unspecified streptococcus as th..	<5	<0.01
B95.6 Staphylococcus aureus as the ca..	<5	<0.01
B95.7 Other staphylococcus as the cau..	<5	<0.01
B96.1 Klebsiella pneumoniae [K. pneum..	<5	<0.01
B96.2 Escherichia coli [E. coli] as t..	<5	<0.01
B96.3 Haemophilus influenzae [H. infl..	<5	<0.01

B96.5 Pseudomonas (aeruginosa) as the..	<5	<0.01
B97.4 Respiratory syncytial virus as ..	<5	<0.01
D73.3 Abscess of spleen	<5	<0.01
G00.2 Streptococcal meningitis	<5	<0.01
G00.3 Staphylococcal meningitis	<5	<0.01
G03.0 Nonpyogenic meningitis	<5	<0.01
G03.1 Chronic meningitis	<5	<0.01
G04.1 Tropical spastic paraplegia	<5	<0.01
G04.2 Bacterial meningoencephalitis a..	<5	<0.01
G05.0 Encephalitis, myelitis and ence..	<5	<0.01
G05.2 Encephalitis, myelitis and ence..	<5	<0.01
G06 Intracranial and intraspinal absc..	<5	<0.01
G06.2 Extradural and subdural abscess..	<5	<0.01
H01.0 Blepharitis	<5	<0.01
H19.0 Scleritis and episcleritis in d..	<5	<0.01
H22.0 Iridocyclitis in infectious and..	<5	<0.01
H32.0 Chorioretinal inflammation in i..	<5	<0.01
H60.2 Malignant otitis externa	<5	<0.01
I32.0 Pericarditis in bacterial disea..	<5	<0.01
I41.1 Myocarditis in viral diseases c..	<5	<0.01
J02.8 Acute pharyngitis due to other ..	<5	<0.01
J03 Acute tonsillitis	<5	<0.01
J10 Influenza due to other identified..	<5	<0.01
J11 Influenza, virus not identified	<5	<0.01
J12.0 Adenoviral pneumonia	<5	<0.01
J12.2 Parainfluenza virus pneumonia	<5	<0.01
J12.8 Other viral pneumonia	<5	<0.01
J15.0 Pneumonia due to Klebsiella pne..	<5	<0.01
J15.1 Pneumonia due to Pseudomonas	<5	<0.01
J15.3 Pneumonia due to streptococcus,..	<5	<0.01
J15.4 Pneumonia due to other streptoc..	<5	<0.01
J15.5 Pneumonia due to Escherichia coli	<5	<0.01
J15.6 Pneumonia due to other aerobic ..	<5	<0.01
J17.0 Pneumonia in bacterial diseases..	<5	<0.01
J17.1 Pneumonia in viral diseases cla..	<5	<0.01
J17.2 Pneumonia in mycoses	<5	<0.01
J18.2 Hypostatic pneumonia, unspecified	<5	<0.01
J20 Acute bronchitis	<5	<0.01
J20.2 Acute bronchitis due to strepto..	<5	<0.01
J20.4 Acute bronchitis due to parainf..	<5	<0.01
J20.6 Acute bronchitis due to rhinovi..	<5	<0.01
J21.8 Acute bronchiolitis due to othe..	<5	<0.01
K02.0 Caries limited to enamel	<5	<0.01
K02.2 Caries of cementum	<5	<0.01
K02.3 Arrested dental caries	<5	<0.01
K23.0 Tuberculous oesophagitis	<5	<0.01
K61.4 Intrasphincteric abscess	<5	<0.01
K67.3 Tuberculous peritonitis	<5	<0.01

K67.8 Other disorders of peritoneum i..	<5	<0.01
L00 Staphylococcal scalded skin syndr..	<5	<0.01
L04.1 Acute lymphadenitis of trunk	<5	<0.01
L04.3 Acute lymphadenitis of lower limb	<5	<0.01
L08.1 Erythrasma	<5	<0.01
M00.8 Arthritis and polyarthritis due..	<5	<0.01
M01.0 Meningococcal arthritis	<5	<0.01
M01.1 Tuberculous arthritis	<5	<0.01
M01.3 Arthritis in other bacterial di..	<5	<0.01
M46.5 Other infective spondylopathies	<5	<0.01
M49.0 Tuberculosis of spine	<5	<0.01
M49.1 Brucella spondylitis	<5	<0.01
M49.3 Spondylopathy in other infectio..	<5	<0.01
M63.0 Myositis in bacterial diseases ..	<5	<0.01
M72.6 Necrotizing fasciitis	<5	<0.01
M86.3 Chronic multifocal osteomyelitis	<5	<0.01
M86.4 Chronic osteomyelitis with drai..	<5	<0.01
M86.8 Other osteomyelitis	<5	<0.01
N16.0 Renal tubulo-interstitial disor..	<5	<0.01
O23.2 Infections of urethra in pregna..	<5	<0.01
O23.3 Infections of other parts of ur..	<5	<0.01
O98.0 Tuberculosis complicating pregn..	<5	<0.01
O98.1 Syphilis complicating pregnancy..	<5	<0.01
O98.6 Protozoal diseases complicating..	<5	<0.01
P36.9 Bacterial sepsis of newborn, un..	<5	<0.01
P37.1 Congenital toxoplasmosis	<5	<0.01
P37.9 Congenital infectious and paras..	<5	<0.01
P39.0 Neonatal infective mastitis	<5	<0.01
P39.1 Neonatal conjunctivitis and dac..	<5	<0.01
P39.9 Infection specific to the perin..	<5	<0.01
R57.2 Septic shock	<5	<0.01
**Total**	**77 108**	**100**

eTable 8. Distribution of bacterial infections in primary analysis (Finnish multicohort sample)

ICD-10 code and description	N	%
K35 Acute appendicitis	12 201	17.69
J18.9 Pneumonia, unspecified	6447	9.35
A09 Other gastroenteritis and colitis..	5917	8.58
N10 Acute tubulo-interstitial nephritis	3728	5.40
J03.9 Acute tonsillitis, unspecified	3306	4.79
A46 Erysipelas	3222	4.67
J36 Peritonsillar abscess	3144	4.56
J15.9 Bacterial pneumonia, unspecified	2373	3.44
N70.0 Acute salpingitis and oophoritis	1991	2.89
O85 Puerperal sepsis	1763	2.56
J01.0 Acute maxillary sinusitis	1136	1.65
N39.0 Urinary tract infection, site n..	1114	1.61

A49.9 Bacterial infection, unspecified	1091	1.58
J03.0 Streptococcal tonsillitis	1005	1.46
J22 Unspecified acute lower respirato..	800	1.16
N71.0 Acute inflammatory disease of u..	737	1.07
J02.9 Acute pharyngitis, unspecified	732	1.06
J04.0 Acute laryngitis	704	1.02
K57.2 Diverticular disease of large i..	465	0.67
J18.0 Bronchopneumonia, unspecified	458	0.66
N30.0 Acute cystitis	455	0.66
A41.5 Sepsis due to other Gram-negati..	439	0.64
L05.9 Pilonidal cyst without abscess	416	0.60
O08.0 Genital tract and pelvic infect..	379	0.55
O03.5 Spontaneous abortion: Complete ..	370	0.54
K61.0 Anal abscess	348	0.50
K05.3 Chronic periodontitis	347	0.50
A04.7 Enterocolitis due to Clostridiu..	325	0.47
H66.0 Acute suppurative otitis media	325	0.47
N75.1 Abscess of Bartholin gland	308	0.45
A41.9 Sepsis, unspecified	304	0.44
J13 Pneumonia due to Streptococcus pn..	282	0.41
K04.7 Periapical abscess without sinus	274	0.40
N61 Inflammatory disorders of breast	266	0.39
A69.2 Lyme disease	249	0.36
L04 Acute lymphadenitis	236	0.34
N45.9 Orchitis, epididymitis and epid..	217	0.31
M00.9 Pyogenic arthritis, unspecified	215	0.31
L03.0 Cellulitis of finger and toe	211	0.31
K04.5 Chronic apical periodontitis	210	0.30
A04.5 Campylobacter enteritis	205	0.30
K65.0 Acute peritonitis	190	0.28
O23.0 Infections of kidney in pregnancy	182	0.26
K12.2 Cellulitis and abscess of mouth	181	0.26
O23.5 Infections of the genital tract..	180	0.26
K61 Abscess of anal and rectal regions	179	0.26
O03.0 Spontaneous abortion: Incomplet..	178	0.26
K02.1 Caries of dentine	176	0.26
J05.1 Acute epiglottitis	175	0.25
L08.9 Local infection of skin and sub..	175	0.25
A41.0 Sepsis due to Staphylococcus au..	174	0.25
O86.0 Infection of obstetric surgical..	171	0.25
L02 Cutaneous abscess, furuncle and c..	169	0.25
A02.0 Salmonella enteritis	165	0.24
O23.9 Other and unspecified genitouri..	162	0.23
L02.4 Cutaneous abscess, furuncle and..	155	0.22
A41.8 Other specified sepsis	151	0.22
J15.8 Other bacterial pneumonia	150	0.22
O86.1 Other infection of genital trac..	138	0.20
A16.2 Tuberculosis of lung, without m..	136	0.20

L05.0 Pilonidal cyst with abscess	130	0.19
G00.9 Bacterial meningitis, unspecified	128	0.19
O86.4 Pyrexia of unknown origin follo..	126	0.18
K05.2 Acute periodontitis	125	0.18
J18.1 Lobar pneumonia, unspecified	117	0.17
A40.3 Sepsis due to Streptococcus pne..	113	0.16
A02 Other salmonella infections	111	0.16
K02 Dental caries	107	0.16
L04.0 Acute lymphadenitis of face, he..	103	0.15
L03.1 Cellulitis of other parts of limb	101	0.15
A56.1 Chlamydial infection of pelvipe..	100	0.14
L02.2 Cutaneous abscess, furuncle and..	97	0.14
J15.7 Pneumonia due to Mycoplasma pne..	96	0.14
O04.0 Medical abortion: Incomplete, c..	94	0.14
O23.4 Unspecified infection of urinar..	93	0.13
A49.8 Other bacterial infections of u..	91	0.13
A04.9 Bacterial intestinal infection,..	90	0.13
H10.0 Mucopurulent conjunctivitis	88	0.13
J86.9 Pyothorax without fistula	86	0.12
O23.1 Infections of bladder in pregna..	85	0.12
H00.1 Chalazion	84	0.12
L02.3 Cutaneous abscess, furuncle and..	84	0.12
A15.0 Tuberculosis of lung, confirmed..	81	0.12
M71.1 Other infective bursitis	81	0.12
H60.3 Other infective otitis externa	79	0.11
J16.8 Pneumonia due to other specifie..	78	0.11
O86.2 Urinary tract infection followi..	78	0.11
L03.9 Cellulitis, unspecified	76	0.11
A54.9 Gonococcal infection, unspecified	75	0.11
L08.8 Other specified local infection..	74	0.11
N41.0 Acute prostatitis	73	0.11
I33.0 Acute and subacute infective en..	71	0.10
N76.4 Abscess of vulva	71	0.10
J39.0 Retropharyngeal and parapharyng..	70	0.10
M86.9 Osteomyelitis, unspecified	69	0.10
O04.5 Medical abortion: Complete or u..	68	0.10
K04.6 Periapical abscess with sinus	67	0.10
K57.0 Diverticular disease of small i..	66	0.10
M86.1 Other acute osteomyelitis	63	0.09
A04.8 Other specified bacterial intes..	62	0.09
O75.3 Other infection during labour	62	0.09
O86.8 Other specified puerperal infec..	62	0.09
O98.8 Other maternal infectious and p..	62	0.09
A54.2 Gonococcal pelviperitonitis and..	58	0.08
H62.0 Otitis externa in bacterial dis..	58	0.08
L02.1 Cutaneous abscess, furuncle and..	56	0.08
L02.9 Cutaneous abscess, furuncle and..	56	0.08
A74.9 Chlamydial infection, unspecified	55	0.08

N73.3 Female acute pelvic peritonitis	53	0.08
A38 Scarlet fever	51	0.07
O91.1 Abscess of breast associated wi..	51	0.07
A40.8 Other streptococcal sepsis	47	0.07
H00.0 Hordeolum and other deep inflam..	47	0.07
A56.0 Chlamydial infection of lower g..	45	0.07
H05.0 Acute inflammation of orbit	45	0.07
J20.0 Acute bronchitis due to Mycopla..	44	0.06
O98.3 Other infections with a predomi..	43	0.06
J02.0 Streptococcal pharyngitis	42	0.06
L03.2 Cellulitis of face	42	0.06
A40.9 Streptococcal sepsis, unspecified	41	0.06
G00.8 Other bacterial meningitis	41	0.06
J18.8 Other pneumonia, organism unspe..	41	0.06
A18.2 Tuberculous peripheral lymphade..	40	0.06
A40.0 Sepsis due to streptococcus, gr..	40	0.06
A41.1 Sepsis due to other specified s..	40	0.06
K61.1 Rectal abscess	40	0.06
K02.9 Dental caries, unspecified	39	0.06
L02.0 Cutaneous abscess, furuncle and..	39	0.06
L08.0 Pyoderma	38	0.06
M71.0 Abscess of bursa	38	0.06
J04.2 Acute laryngotracheitis	37	0.05
A49.0 Staphylococcal infection, unspe..	36	0.05
A04.6 Enteritis due to Yersinia enter..	35	0.05
L02.8 Cutaneous abscess, furuncle and..	35	0.05
H60.1 Cellulitis of external ear	33	0.05
H70.0 Acute mastoiditis	33	0.05
A02.1 Salmonella sepsis	32	0.05
A39.0 Meningococcal meningitis	32	0.05
M86.6 Other chronic osteomyelitis	32	0.05
A16.5 Tuberculous pleurisy, without m..	30	0.04
H60.0 Abscess of external ear	30	0.04
G00.0 Haemophilus meningitis	29	0.04
N73.0 Acute parametritis and pelvic c..	29	0.04
A37 Whooping cough	28	0.04
J85.2 Abscess of lung without pneumonia	28	0.04
A41.2 Sepsis due to unspecified staph..	26	0.04
M00.0 Staphylococcal arthritis and po..	26	0.04
A05.9 Bacterial foodborne intoxicatio..	25	0.04
H44.0 Purulent endophthalmitis	25	0.04
J86.0 Pyothorax with fistula	25	0.04
K63.0 Abscess of intestine	25	0.04
K75.0 Abscess of liver	24	0.03
G00.1 Pneumococcal meningitis	23	0.03
O86.3 Other genitourinary tract infec..	23	0.03
O98.9 Unspecified maternal infectious..	23	0.03
A21.9 Tularaemia, unspecified	22	0.03

K11.3 Abscess of salivary gland	22	0.03
L03.3 Cellulitis of trunk	22	0.03
M65.0 Abscess of tendon sheath	22	0.03
A18.0 Tuberculosis of bones and joints	21	0.03
A21.0 Ulceroglandular tularaemia	21	0.03
G06.0 Intracranial abscess and granul..	21	0.03
K61.2 Anorectal abscess	21	0.03
N76.0 Acute vaginitis	21	0.03
A54.1 Gonococcal infection of lower g..	20	0.03
G01 Meningitis in bacterial diseases ..	20	0.03
K04.4 Acute apical periodontitis of p..	20	0.03
A48.8 Other specified bacterial disea..	19	0.03
J04.1 Acute tracheitis	18	0.03
N74.4 Female chlamydial pelvic inflam..	18	0.03
A02.9 Salmonella infection, unspecified	17	0.02
A41.4 Sepsis due to anaerobes	17	0.02
J86 Pyothorax	17	0.02
N15.1 Renal and perinephric abscess	17	0.02
A15.1 Tuberculosis of lung, confirmed..	16	0.02
L01.0 Impetigo [any organism] [any si..	16	0.02
A40.1 Sepsis due to streptococcus, gr..	15	0.02
A51.0 Primary genital syphilis	15	0.02
J03.8 Acute tonsillitis due to other ..	15	0.02
L03.8 Cellulitis of other sites	15	0.02
M46.3 Infection of intervertebral dis..	15	0.02
N45.0 Orchitis, epididymitis and epid..	15	0.02
O91.0 Infection of nipple associated ..	15	0.02
A51.3 Secondary syphilis of skin and ..	14	0.02
K57.4 Diverticular disease of both sm..	14	0.02
A01.4 Paratyphoid fever, unspecified	13	0.02
A03.3 Shigellosis due to Shigella son..	13	0.02
J85.1 Abscess of lung with pneumonia	13	0.02
N34.0 Urethral abscess	13	0.02
A64 Unspecified sexually transmitted ..	12	0.02
G06.1 Intraspinal abscess and granuloma	12	0.02
L01 Impetigo	12	0.02
L70.1 Acne conglobata	12	0.02
O06.5 Unspecified abortion: Complete ..	12	0.02
J14 Pneumonia due to Haemophilus infl..	11	0.02
L04.9 Acute lymphadenitis, unspecified	11	0.02
M00.2 Other streptococcal arthritis a..	11	0.02
A01.2 Paratyphoid fever B	10	0.01
A03.1 Shigellosis due to Shigella fle..	10	0.01
A39.2 Acute meningococcaemia	10	0.01
J16.0 Chlamydial pneumonia	10	0.01
K61.3 Ischiorectal abscess	10	0.01
L01.1 Impetiginization of other derma..	10	0.01
M01.2 Arthritis in Lyme disease	10	0.01

M46.2 Osteomyelitis of vertebra	10	0.01
N13.6 Pyonephrosis	10	0.01
A01.0 Typhoid fever	8	0.01
A02.2 Localized salmonella infections	8	0.01
A02.8 Other specified salmonella infe..	8	0.01
A15.6 Tuberculous pleurisy, confirmed..	8	0.01
A16.0 Tuberculosis of lung, bacteriol..	8	0.01
A16.9 Respiratory tuberculosis unspec..	8	0.01
A18.1 Tuberculosis of genitourinary s..	8	0.01
A31.0 Pulmonary mycobacterial infection	8	0.01
A32.8 Other forms of listeriosis	8	0.01
A39.4 Meningococcaemia, unspecified	8	0.01
A49.1 Streptococcal infection, unspec..	8	0.01
J39.1 Other abscess of pharynx	8	0.01
K02.8 Other dental caries	8	0.01
O07.0 Failed medical abortion, compli..	8	0.01
O07.5 Other and unspecified failed at..	8	0.01
A74.8 Other chlamydial diseases	7	0.01
J20.1 Acute bronchitis due to Haemoph..	7	0.01
K05.0 Acute gingivitis	7	0.01
O05.0 Other abortion: Incomplete, com..	7	0.01
O05.5 Other abortion: Complete or uns..	7	0.01
A04.3 Enterohaemorrhagic Escherichia ..	6	0.01
A18.3 Tuberculosis of intestines, per..	6	0.01
A31 Infection due to other mycobacteria	6	0.01
A48.0 Gas gangrene	6	0.01
A52.1 Symptomatic neurosyphilis	6	0.01
A54.0 Gonococcal infection of lower g..	6	0.01
H10.5 Blepharoconjunctivitis	6	0.01
J15.0 Pneumonia due to Klebsiella pne..	6	0.01
J85.3 Abscess of mediastinum	6	0.01
M65.1 Other infective (teno)synovitis	6	0.01
O06.0 Unspecified abortion: Incomplet..	6	0.01
A03.9 Shigellosis, unspecified	5	0.01
A15.2 Tuberculosis of lung, confirmed..	5	0.01
A18.4 Tuberculosis of skin and subcut..	5	0.01
A31.9 Mycobacterial infection, unspec..	5	0.01
A32.7 Listerial sepsis	5	0.01
B95.8 Unspecified staphylococcus as t..	5	0.01
G05.0 Encephalitis, myelitis and ence..	5	0.01
J15.2 Pneumonia due to staphylococcus	5	0.01
K57.8 Diverticular disease of intesti..	5	0.01
L04.2 Acute lymphadenitis of upper limb	5	0.01
L04.8 Acute lymphadenitis of other si..	5	0.01
M86.0 Acute haematogenous osteomyelitis	5	0.01
M86.2 Subacute osteomyelitis	5	0.01
A00.9 Cholera, unspecified	<5	<0.01
A01.1 Paratyphoid fever A	<5	<0.01

A01.3 Paratyphoid fever C	<5	<0.01
A03 Shigellosis	<5	<0.01
A03.0 Shigellosis due to Shigella dys..	<5	<0.01
A03.2 Shigellosis due to Shigella boy..	<5	<0.01
A04 Other bacterial intestinal infect..	<5	<0.01
A04.0 Enteropathogenic Escherichia co..	<5	<0.01
A04.1 Enterotoxigenic Escherichia col..	<5	<0.01
A04.2 Enteroinvasive Escherichia coli..	<5	<0.01
A04.4 Other intestinal Escherichia co..	<5	<0.01
A05.0 Foodborne staphylococcal intoxi..	<5	<0.01
A05.1 Botulism	<5	<0.01
A05.2 Foodborne Clostridium perfringe..	<5	<0.01
A05.8 Other specified bacterial foodb..	<5	<0.01
A15.3 Tuberculosis of lung, confirmed..	<5	<0.01
A15.4 Tuberculosis of intrathoracic l..	<5	<0.01
A15.5 Tuberculosis of larynx, trachea..	<5	<0.01
A15.7 Primary respiratory tuberculosi..	<5	<0.01
A15.8 Other respiratory tuberculosis,..	<5	<0.01
A15.9 Respiratory tuberculosis unspec..	<5	<0.01
A16.1 Tuberculosis of lung, bacteriol..	<5	<0.01
A16.3 Tuberculosis of intrathoracic l..	<5	<0.01
A16.4 Tuberculosis of larynx, trachea..	<5	<0.01
A16.7 Primary respiratory tuberculosi..	<5	<0.01
A16.8 Other respiratory tuberculosis,..	<5	<0.01
A17.0 Tuberculous meningitis	<5	<0.01
A17.9 Tuberculosis of nervous system,..	<5	<0.01
A18.8 Tuberculosis of other specified..	<5	<0.01
A19.0 Acute miliary tuberculosis of a..	<5	<0.01
A19.2 Acute miliary tuberculosis, uns..	<5	<0.01
A19.9 Miliary tuberculosis, unspecified	<5	<0.01
A20.9 Plague, unspecified	<5	<0.01
A21.1 Oculoglandular tularaemia	<5	<0.01
A21.2 Pulmonary tularaemia	<5	<0.01
A21.7 Generalized tularaemia	<5	<0.01
A21.8 Other forms of tularaemia	<5	<0.01
A22.0 Cutaneous anthrax	<5	<0.01
A22.2 Gastrointestinal anthrax	<5	<0.01
A23.9 Brucellosis, unspecified	<5	<0.01
A24.0 Glanders	<5	<0.01
A26.0 Cutaneous erysipeloid	<5	<0.01
A26.9 Erysipeloid, unspecified	<5	<0.01
A27 Leptospirosis	<5	<0.01
A27.0 Leptospirosis icterohaemorrhagica	<5	<0.01
A27.8 Other forms of leptospirosis	<5	<0.01
A27.9 Leptospirosis, unspecified	<5	<0.01
A28.2 Extraintestinal yersiniosis	<5	<0.01
A28.8 Other specified zoonotic bacter..	<5	<0.01
A28.9 Zoonotic bacterial disease, uns..	<5	<0.01

A30 Leprosy [Hansen disease]	<5	<0.01
A31.1 Cutaneous mycobacterial infection	<5	<0.01
A31.8 Other mycobacterial infections	<5	<0.01
A32.9 Listeriosis, unspecified	<5	<0.01
A35 Other tetanus	<5	<0.01
A36 Diphtheria	<5	<0.01
A37.0 Whooping cough due to Bordetell..	<5	<0.01
A37.9 Whooping cough, unspecified	<5	<0.01
A39 Meningococcal infection	<5	<0.01
A39.8 Other meningococcal infections	<5	<0.01
A39.9 Meningococcal infection, unspec..	<5	<0.01
A40 Streptococcal sepsis	<5	<0.01
A40.2 Sepsis due to streptococcus, gr..	<5	<0.01
A41 Other sepsis	<5	<0.01
A41.3 Sepsis due to Haemophilus influ..	<5	<0.01
A42 Actinomycosis	<5	<0.01
A42.0 Pulmonary actinomycosis	<5	<0.01
A42.1 Abdominal actinomycosis	<5	<0.01
A42.2 Cervicofacial actinomycosis	<5	<0.01
A42.7 Actinomycotic sepsis	<5	<0.01
A42.8 Other forms of actinomycosis	<5	<0.01
A42.9 Actinomycosis, unspecified	<5	<0.01
A44.1 Cutaneous and mucocutaneous bar..	<5	<0.01
A49.2 Haemophilus influenzae infectio..	<5	<0.01
A49.3 Mycoplasma infection, unspecifi..	<5	<0.01
A50 Congenital syphilis	<5	<0.01
A50.5 Other late congenital syphilis,..	<5	<0.01
A50.9 Congenital syphilis, unspecified	<5	<0.01
A51.2 Primary syphilis of other sites	<5	<0.01
A51.4 Other secondary syphilis	<5	<0.01
A51.5 Early syphilis, latent	<5	<0.01
A51.9 Early syphilis, unspecified	<5	<0.01
A52.0 Cardiovascular syphilis	<5	<0.01
A52.7 Other symptomatic late syphilis	<5	<0.01
A52.8 Late syphilis, latent	<5	<0.01
A53 Other and unspecified syphilis	<5	<0.01
A53.9 Syphilis, unspecified	<5	<0.01
A54 Gonococcal infection	<5	<0.01
A54.3 Gonococcal infection of eye	<5	<0.01
A54.4 Gonococcal infection of musculo..	<5	<0.01
A54.8 Other gonococcal infections	<5	<0.01
A55 Chlamydial lymphogranuloma (vener..	<5	<0.01
A56.2 Chlamydial infection of genitou..	<5	<0.01
A56.3 Chlamydial infection of anus an..	<5	<0.01
A56.8 Sexually transmitted chlamydial..	<5	<0.01
A58 Granuloma inguinale	<5	<0.01
A66.1 Multiple papillomata and wet cr..	<5	<0.01
A66.3 Hyperkeratosis of yaws	<5	<0.01

A67 Pinta [carate]	<5	<0.01
A67.1 Intermediate lesions of pinta	<5	<0.01
A68 Relapsing fevers	<5	<0.01
A69.0 Necrotizing ulcerative stomatitis	<5	<0.01
A69.1 Other Vincent infections	<5	<0.01
A69.9 Spirochaetal infection, unspeci..	<5	<0.01
A70 Chlamydia psittaci infection	<5	<0.01
A71 Trachoma	<5	<0.01
A75.0 Epidemic louse-borne typhus fev..	<5	<0.01
A75.9 Typhus fever, unspecified	<5	<0.01
A77.1 Spotted fever due to Rickettsia..	<5	<0.01
A77.8 Other spotted fevers	<5	<0.01
A78 Q fever	<5	<0.01
A79.9 Rickettsiosis, unspecified	<5	<0.01
B95.0 Streptococcus, group A, as the ..	<5	<0.01
B95.1 Streptococcus, group B, as the ..	<5	<0.01
B95.3 Streptococcus pneumoniae as the..	<5	<0.01
B95.4 Other streptococcus as the caus..	<5	<0.01
B95.5 Unspecified streptococcus as th..	<5	<0.01
B95.6 Staphylococcus aureus as the ca..	<5	<0.01
B95.7 Other staphylococcus as the cau..	<5	<0.01
B96.1 Klebsiella pneumoniae [K. pneum..	<5	<0.01
B96.2 Escherichia coli [E. coli] as t..	<5	<0.01
B96.3 Haemophilus influenzae [H. infl..	<5	<0.01
B96.5 Pseudomonas (aeruginosa) as the..	<5	<0.01
B96.8 Other specified bacterial agent..	<5	<0.01
D73.3 Abscess of spleen	<5	<0.01
G00.2 Streptococcal meningitis	<5	<0.01
G00.3 Staphylococcal meningitis	<5	<0.01
G04.2 Bacterial meningoencephalitis a..	<5	<0.01
G06 Intracranial and intraspinal absc..	<5	<0.01
G06.2 Extradural and subdural abscess..	<5	<0.01
H01.0 Blepharitis	<5	<0.01
H60.2 Malignant otitis externa	<5	<0.01
I32.0 Pericarditis in bacterial disea..	<5	<0.01
I41.0 Myocarditis in bacterial diseas..	<5	<0.01
J02.8 Acute pharyngitis due to other ..	<5	<0.01
J03 Acute tonsillitis	<5	<0.01
J15.1 Pneumonia due to Pseudomonas	<5	<0.01
J15.3 Pneumonia due to streptococcus,..	<5	<0.01
J15.4 Pneumonia due to other streptoc..	<5	<0.01
J15.5 Pneumonia due to Escherichia coli	<5	<0.01
J15.6 Pneumonia due to other aerobic ..	<5	<0.01
J17.0 Pneumonia in bacterial diseases..	<5	<0.01
J18.2 Hypostatic pneumonia, unspecified	<5	<0.01
J20.2 Acute bronchitis due to strepto..	<5	<0.01
K02.0 Caries limited to enamel	<5	<0.01
K02.2 Caries of cementum	<5	<0.01

K02.3 Arrested dental caries	<5	<0.01
K23.0 Tuberculous oesophagitis	<5	<0.01
K61.4 Intrasphincteric abscess	<5	<0.01
K67.3 Tuberculous peritonitis	<5	<0.01
L00 Staphylococcal scalded skin syndr..	<5	<0.01
L04.1 Acute lymphadenitis of trunk	<5	<0.01
L04.3 Acute lymphadenitis of lower limb	<5	<0.01
L08.1 Erythrasma	<5	<0.01
M00.8 Arthritis and polyarthritis due..	<5	<0.01
M01.0 Meningococcal arthritis	<5	<0.01
M01.1 Tuberculous arthritis	<5	<0.01
M01.3 Arthritis in other bacterial di..	<5	<0.01
M46.5 Other infective spondylopathies	<5	<0.01
M49.0 Tuberculosis of spine	<5	<0.01
M49.1 Brucella spondylitis	<5	<0.01
M63.0 Myositis in bacterial diseases ..	<5	<0.01
M72.6 Necrotizing fasciitis	<5	<0.01
M86.3 Chronic multifocal osteomyelitis	<5	<0.01
M86.4 Chronic osteomyelitis with drai..	<5	<0.01
M86.8 Other osteomyelitis	<5	<0.01
O23.2 Infections of urethra in pregna..	<5	<0.01
O23.3 Infections of other parts of ur..	<5	<0.01
O98.0 Tuberculosis complicating pregn..	<5	<0.01
O98.1 Syphilis complicating pregnancy..	<5	<0.01
P36.9 Bacterial sepsis of newborn, un..	<5	<0.01
P39.0 Neonatal infective mastitis	<5	<0.01
P39.1 Neonatal conjunctivitis and dac..	<5	<0.01
**Total**	**68 979**	**100**

eTable 9. Distribution of invasive bacterial infections in primary analysis (Finnish multicohort sample)

ICD-10 code and description	N	%
K35 Acute appendicitis	13 003	25.53
J18.9 Pneumonia, unspecified	7258	14.25
N10 Acute tubulo-interstitial nephritis	4076	8.00
A46 Erysipelas	3608	7.08
J36 Peritonsillar abscess	3380	6.64
J15.9 Bacterial pneumonia, unspecified	2656	5.21
N70.0 Acute salpingitis and oophoritis	2054	4.03
O85 Puerperal sepsis	1822	3.58
A49.9 Bacterial infection, unspecified	1228	2.41
N71.0 Acute inflammatory disease of u..	772	1.52
J18.0 Bronchopneumonia, unspecified	514	1.01
K57.2 Diverticular disease of large i..	496	0.97
A41.5 Sepsis due to other Gram-negati..	489	0.96
K61.0 Anal abscess	416	0.82
O08.0 Genital tract and pelvic infect..	382	0.75

O03.5 Spontaneous abortion: Complete ..	380	0.75
A41.9 Sepsis, unspecified	360	0.71
N75.1 Abscess of Bartholin gland	319	0.63
J13 Pneumonia due to Streptococcus pn..	310	0.61
N61 Inflammatory disorders of breast	278	0.55
A69.2 Lyme disease	271	0.53
N45.9 Orchitis, epididymitis and epid..	248	0.49
M00.9 Pyogenic arthritis, unspecified	233	0.46
K12.2 Cellulitis and abscess of mouth	218	0.43
K65.0 Acute peritonitis	211	0.41
A41.0 Sepsis due to Staphylococcus au..	208	0.41
J05.1 Acute epiglottitis	202	0.40
O23.0 Infections of kidney in pregnancy	196	0.38
A02.0 Salmonella enteritis	195	0.38
K61 Abscess of anal and rectal regions	188	0.37
O03.0 Spontaneous abortion: Incomplet..	188	0.37
J15.8 Other bacterial pneumonia	166	0.33
A41.8 Other specified sepsis	165	0.32
A16.2 Tuberculosis of lung, without m..	138	0.27
G00.9 Bacterial meningitis, unspecified	132	0.26
J18.1 Lobar pneumonia, unspecified	132	0.26
A40.3 Sepsis due to Streptococcus pne..	126	0.25
A02 Other salmonella infections	118	0.23
J15.7 Pneumonia due to Mycoplasma pne..	109	0.21
A56.1 Chlamydial infection of pelvipe..	106	0.21
O04.0 Medical abortion: Incomplete, c..	106	0.21
A49.8 Other bacterial infections of u..	96	0.19
J86.9 Pyothorax without fistula	90	0.18
A15.0 Tuberculosis of lung, confirmed..	86	0.17
J16.8 Pneumonia due to other specifie..	85	0.17
M71.1 Other infective bursitis	85	0.17
M86.9 Osteomyelitis, unspecified	83	0.16
J39.0 Retropharyngeal and parapharyng..	81	0.16
I33.0 Acute and subacute infective en..	76	0.15
N76.4 Abscess of vulva	74	0.15
M86.1 Other acute osteomyelitis	73	0.14
O04.5 Medical abortion: Complete or u..	73	0.14
K57.0 Diverticular disease of small i..	68	0.13
O75.3 Other infection during labour	68	0.13
A54.2 Gonococcal pelviperitonitis and..	62	0.12
A40.8 Other streptococcal sepsis	56	0.11
O91.1 Abscess of breast associated wi..	56	0.11
A38 Scarlet fever	55	0.11
N73.3 Female acute pelvic peritonitis	55	0.11
A41.1 Sepsis due to other specified s..	53	0.10
A40.0 Sepsis due to streptococcus, gr..	51	0.10
H05.0 Acute inflammation of orbit	50	0.10
J18.8 Other pneumonia, organism unspe..	48	0.09

A18.2 Tuberculous peripheral lymphade..	45	0.09
A40.9 Streptococcal sepsis, unspecified	45	0.09
K61.1 Rectal abscess	44	0.09
M71.0 Abscess of bursa	44	0.09
A49.0 Staphylococcal infection, unspe..	43	0.08
G00.8 Other bacterial meningitis	42	0.08
H60.1 Cellulitis of external ear	38	0.07
H70.0 Acute mastoiditis	38	0.07
H60.0 Abscess of external ear	35	0.07
A02.1 Salmonella sepsis	34	0.07
A39.0 Meningococcal meningitis	34	0.07
M86.6 Other chronic osteomyelitis	33	0.06
N73.0 Acute parametritis and pelvic c..	32	0.06
A16.5 Tuberculous pleurisy, without m..	31	0.06
G00.0 Haemophilus meningitis	30	0.06
J85.2 Abscess of lung without pneumonia	30	0.06
K75.0 Abscess of liver	29	0.06
A41.2 Sepsis due to unspecified staph..	28	0.05
H44.0 Purulent endophthalmitis	28	0.05
J86.0 Pyothorax with fistula	27	0.05
K63.0 Abscess of intestine	27	0.05
M00.0 Staphylococcal arthritis and po..	26	0.05
A21.9 Tularaemia, unspecified	24	0.05
G00.1 Pneumococcal meningitis	24	0.05
M65.0 Abscess of tendon sheath	24	0.05
A21.0 Ulceroglandular tularaemia	23	0.05
G06.0 Intracranial abscess and granul..	23	0.05
K61.2 Anorectal abscess	23	0.05
A18.0 Tuberculosis of bones and joints	21	0.04
A40.1 Sepsis due to streptococcus, gr..	21	0.04
A48.8 Other specified bacterial disea..	21	0.04
G01 Meningitis in bacterial diseases ..	21	0.04
A54.1 Gonococcal infection of lower g..	20	0.04
A41.4 Sepsis due to anaerobes	19	0.04
J86 Pyothorax	19	0.04
N15.1 Renal and perinephric abscess	19	0.04
A02.9 Salmonella infection, unspecified	18	0.04
M46.3 Infection of intervertebral dis..	18	0.04
N74.4 Female chlamydial pelvic inflam..	18	0.04
A15.1 Tuberculosis of lung, confirmed..	17	0.03
A51.0 Primary genital syphilis	17	0.03
N45.0 Orchitis, epididymitis and epid..	17	0.03
A51.3 Secondary syphilis of skin and ..	15	0.03
K57.4 Diverticular disease of both sm..	15	0.03
A03.3 Shigellosis due to Shigella son..	14	0.03
G06.1 Intraspinal abscess and granuloma	14	0.03
J85.1 Abscess of lung with pneumonia	14	0.03
A01.4 Paratyphoid fever, unspecified	13	0.03

N34.0 Urethral abscess	13	0.03
O06.5 Unspecified abortion: Complete ..	13	0.03
A39.2 Acute meningococcaemia	12	0.02
J14 Pneumonia due to Haemophilus infl..	12	0.02
A03.1 Shigellosis due to Shigella fle..	11	0.02
J16.0 Chlamydial pneumonia	11	0.02
M00.2 Other streptococcal arthritis a..	11	0.02
M46.2 Osteomyelitis of vertebra	11	0.02
O07.0 Failed medical abortion, compli..	11	0.02
A01.2 Paratyphoid fever B	10	0.02
A02.8 Other specified salmonella infe..	10	0.02
A31.0 Pulmonary mycobacterial infection	10	0.02
B95.8 Unspecified staphylococcus as t..	10	0.02
K61.3 Ischiorectal abscess	10	0.02
M01.2 Arthritis in Lyme disease	10	0.02
N13.6 Pyonephrosis	10	0.02
O07.5 Other and unspecified failed at..	10	0.02
A18.1 Tuberculosis of genitourinary s..	9	0.02
A49.1 Streptococcal infection, unspec..	9	0.02
A01.0 Typhoid fever	8	0.02
A02.2 Localized salmonella infections	8	0.02
A15.6 Tuberculous pleurisy, confirmed..	8	0.02
A16.0 Tuberculosis of lung, bacteriol..	8	0.02
A16.9 Respiratory tuberculosis unspec..	8	0.02
A32.8 Other forms of listeriosis	8	0.02
A39.4 Meningococcaemia, unspecified	8	0.02
J39.1 Other abscess of pharynx	8	0.02
A03.9 Shigellosis, unspecified	7	0.01
A32.7 Listerial sepsis	7	0.01
A48.0 Gas gangrene	7	0.01
A52.1 Symptomatic neurosyphilis	7	0.01
B95.5 Unspecified streptococcus as th..	7	0.01
J15.0 Pneumonia due to Klebsiella pne..	7	0.01
M86.0 Acute haematogenous osteomyelitis	7	0.01
M86.8 Other osteomyelitis	7	0.01
O05.0 Other abortion: Incomplete, com..	7	0.01
O05.5 Other abortion: Complete or uns..	7	0.01
O06.0 Unspecified abortion: Incomplet..	7	0.01
A15.2 Tuberculosis of lung, confirmed..	6	0.01
A18.3 Tuberculosis of intestines, per..	6	0.01
A31 Infection due to other mycobacteria	6	0.01
B96.2 Escherichia coli [E. coli] as t..	6	0.01
G05.0 Encephalitis, myelitis and ence..	6	0.01
J85.3 Abscess of mediastinum	6	0.01
M65.1 Other infective (teno)synovitis	6	0.01
A18.4 Tuberculosis of skin and subcut..	5	0.01
A31.9 Mycobacterial infection, unspec..	5	0.01
B95.0 Streptococcus, group A, as the ..	5	0.01

B95.6 Staphylococcus aureus as the ca..	5	0.01
B96.8 Other specified bacterial agent..	5	0.01
G00.2 Streptococcal meningitis	5	0.01
J15.2 Pneumonia due to staphylococcus	5	0.01
K57.8 Diverticular disease of intesti..	5	0.01
M01.3 Arthritis in other bacterial di..	5	0.01
M86.2 Subacute osteomyelitis	5	0.01
O98.0 Tuberculosis complicating pregn..	5	0.01
A01.1 Paratyphoid fever A	<5	<0.01
A01.3 Paratyphoid fever C	<5	<0.01
A03 Shigellosis	<5	<0.01
A03.0 Shigellosis due to Shigella dys..	<5	<0.01
A03.2 Shigellosis due to Shigella boy..	<5	<0.01
A15.3 Tuberculosis of lung, confirmed..	<5	<0.01
A15.4 Tuberculosis of intrathoracic l..	<5	<0.01
A15.5 Tuberculosis of larynx, trachea..	<5	<0.01
A15.7 Primary respiratory tuberculosi..	<5	<0.01
A15.8 Other respiratory tuberculosis,..	<5	<0.01
A15.9 Respiratory tuberculosis unspec..	<5	<0.01
A16.1 Tuberculosis of lung, bacteriol..	<5	<0.01
A16.3 Tuberculosis of intrathoracic l..	<5	<0.01
A16.4 Tuberculosis of larynx, trachea..	<5	<0.01
A16.7 Primary respiratory tuberculosi..	<5	<0.01
A16.8 Other respiratory tuberculosis,..	<5	<0.01
A17.0 Tuberculous meningitis	<5	<0.01
A17.8 Other tuberculosis of nervous s..	<5	<0.01
A17.9 Tuberculosis of nervous system,..	<5	<0.01
A18.8 Tuberculosis of other specified..	<5	<0.01
A19.0 Acute miliary tuberculosis of a..	<5	<0.01
A19.2 Acute miliary tuberculosis, uns..	<5	<0.01
A19.9 Miliary tuberculosis, unspecified	<5	<0.01
A20.9 Plague, unspecified	<5	<0.01
A21.1 Oculoglandular tularaemia	<5	<0.01
A21.2 Pulmonary tularaemia	<5	<0.01
A21.7 Generalized tularaemia	<5	<0.01
A21.8 Other forms of tularaemia	<5	<0.01
A22.0 Cutaneous anthrax	<5	<0.01
A22.2 Gastrointestinal anthrax	<5	<0.01
A23.9 Brucellosis, unspecified	<5	<0.01
A24.0 Glanders	<5	<0.01
A25.9 Rat-bite fever, unspecified	<5	<0.01
A27 Leptospirosis	<5	<0.01
A27.0 Leptospirosis icterohaemorrhagica	<5	<0.01
A27.8 Other forms of leptospirosis	<5	<0.01
A27.9 Leptospirosis, unspecified	<5	<0.01
A28.2 Extraintestinal yersiniosis	<5	<0.01
A28.8 Other specified zoonotic bacter..	<5	<0.01
A28.9 Zoonotic bacterial disease, uns..	<5	<0.01

A30 Leprosy [Hansen disease]	<5	<0.01
A31.1 Cutaneous mycobacterial infection	<5	<0.01
A31.8 Other mycobacterial infections	<5	<0.01
A32.9 Listeriosis, unspecified	<5	<0.01
A35 Other tetanus	<5	<0.01
A36 Diphtheria	<5	<0.01
A39 Meningococcal infection	<5	<0.01
A39.8 Other meningococcal infections	<5	<0.01
A39.9 Meningococcal infection, unspec..	<5	<0.01
A40 Streptococcal sepsis	<5	<0.01
A40.2 Sepsis due to streptococcus, gr..	<5	<0.01
A41 Other sepsis	<5	<0.01
A41.3 Sepsis due to Haemophilus influ..	<5	<0.01
A42 Actinomycosis	<5	<0.01
A42.0 Pulmonary actinomycosis	<5	<0.01
A42.1 Abdominal actinomycosis	<5	<0.01
A42.2 Cervicofacial actinomycosis	<5	<0.01
A42.7 Actinomycotic sepsis	<5	<0.01
A42.8 Other forms of actinomycosis	<5	<0.01
A42.9 Actinomycosis, unspecified	<5	<0.01
A44.1 Cutaneous and mucocutaneous bar..	<5	<0.01
A49.2 Haemophilus influenzae infectio..	<5	<0.01
A50 Congenital syphilis	<5	<0.01
A50.5 Other late congenital syphilis,..	<5	<0.01
A50.9 Congenital syphilis, unspecified	<5	<0.01
A51.2 Primary syphilis of other sites	<5	<0.01
A51.4 Other secondary syphilis	<5	<0.01
A51.5 Early syphilis, latent	<5	<0.01
A51.9 Early syphilis, unspecified	<5	<0.01
A52.0 Cardiovascular syphilis	<5	<0.01
A52.7 Other symptomatic late syphilis	<5	<0.01
A52.8 Late syphilis, latent	<5	<0.01
A53 Other and unspecified syphilis	<5	<0.01
A53.9 Syphilis, unspecified	<5	<0.01
A54.4 Gonococcal infection of musculo..	<5	<0.01
A54.8 Other gonococcal infections	<5	<0.01
A55 Chlamydial lymphogranuloma (vener..	<5	<0.01
A66.1 Multiple papillomata and wet cr..	<5	<0.01
A66.3 Hyperkeratosis of yaws	<5	<0.01
A67 Pinta [carate]	<5	<0.01
A67.1 Intermediate lesions of pinta	<5	<0.01
A68 Relapsing fevers	<5	<0.01
A69.0 Necrotizing ulcerative stomatitis	<5	<0.01
A69.1 Other Vincent infections	<5	<0.01
A69.9 Spirochaetal infection, unspeci..	<5	<0.01
A70 Chlamydia psittaci infection	<5	<0.01
A75.0 Epidemic louse-borne typhus fev..	<5	<0.01
A75.9 Typhus fever, unspecified	<5	<0.01

A77.1 Spotted fever due to Rickettsia..	<5	<0.01
A77.8 Other spotted fevers	<5	<0.01
A78 Q fever	<5	<0.01
A79.9 Rickettsiosis, unspecified	<5	<0.01
B95.1 Streptococcus, group B, as the ..	<5	<0.01
B95.3 Streptococcus pneumoniae as the..	<5	<0.01
B95.4 Other streptococcus as the caus..	<5	<0.01
B95.7 Other staphylococcus as the cau..	<5	<0.01
B96.1 Klebsiella pneumoniae [K. pneum..	<5	<0.01
B96.3 Haemophilus influenzae [H. infl..	<5	<0.01
B96.5 Pseudomonas (aeruginosa) as the..	<5	<0.01
D73.3 Abscess of spleen	<5	<0.01
G00.3 Staphylococcal meningitis	<5	<0.01
G04.2 Bacterial meningoencephalitis a..	<5	<0.01
G06 Intracranial and intraspinal absc..	<5	<0.01
G06.2 Extradural and subdural abscess..	<5	<0.01
H60.2 Malignant otitis externa	<5	<0.01
I32.0 Pericarditis in bacterial disea..	<5	<0.01
I41.0 Myocarditis in bacterial diseas..	<5	<0.01
I52.0 Other heart disorders in bacter..	<5	<0.01
J15.1 Pneumonia due to Pseudomonas	<5	<0.01
J15.3 Pneumonia due to streptococcus,..	<5	<0.01
J15.4 Pneumonia due to other streptoc..	<5	<0.01
J15.5 Pneumonia due to Escherichia coli	<5	<0.01
J15.6 Pneumonia due to other aerobic ..	<5	<0.01
J17.0 Pneumonia in bacterial diseases..	<5	<0.01
J18.2 Hypostatic pneumonia, unspecified	<5	<0.01
K23.0 Tuberculous oesophagitis	<5	<0.01
K61.4 Intrasphincteric abscess	<5	<0.01
K67.3 Tuberculous peritonitis	<5	<0.01
M00.8 Arthritis and polyarthritis due..	<5	<0.01
M01.0 Meningococcal arthritis	<5	<0.01
M01.1 Tuberculous arthritis	<5	<0.01
M46.5 Other infective spondylopathies	<5	<0.01
M49.0 Tuberculosis of spine	<5	<0.01
M49.1 Brucella spondylitis	<5	<0.01
M63.0 Myositis in bacterial diseases ..	<5	<0.01
M72.6 Necrotizing fasciitis	<5	<0.01
M86.3 Chronic multifocal osteomyelitis	<5	<0.01
M86.4 Chronic osteomyelitis with drai..	<5	<0.01
O98.1 Syphilis complicating pregnancy..	<5	<0.01
P36.9 Bacterial sepsis of newborn, un..	<5	<0.01
P39.0 Neonatal infective mastitis	<5	<0.01
**Total**	**50 938**	**100**

eTable 10. Distribution of localised bacterial infections in primary analysis (Finnish multicohort sample)

ICD-10 code and description	N	%
A09 Other gastroenteritis and colitis..	6929	26.05
J03.9 Acute tonsillitis, unspecified	3647	13.71
J01.0 Acute maxillary sinusitis	1530	5.75
N39.0 Urinary tract infection, site n..	1518	5.71
J03.0 Streptococcal tonsillitis	1147	4.31
J22 Unspecified acute lower respirato..	931	3.50
J02.9 Acute pharyngitis, unspecified	804	3.02
J04.0 Acute laryngitis	742	2.79
N30.0 Acute cystitis	645	2.42
A04.7 Enterocolitis due to Clostridiu..	555	2.09
L05.9 Pilonidal cyst without abscess	479	1.80
K05.3 Chronic periodontitis	413	1.55
H66.0 Acute suppurative otitis media	370	1.39
K04.7 Periapical abscess without sinus	333	1.25
K04.5 Chronic apical periodontitis	273	1.03
L04 Acute lymphadenitis	262	0.98
A04.5 Campylobacter enteritis	253	0.95
L03.0 Cellulitis of finger and toe	244	0.92
L02.4 Cutaneous abscess, furuncle and..	243	0.91
K02.1 Caries of dentine	240	0.90
O23.5 Infections of the genital tract..	206	0.77
O86.0 Infection of obstetric surgical..	195	0.73
L08.9 Local infection of skin and sub..	194	0.73
O23.9 Other and unspecified genitouri..	187	0.70
L02 Cutaneous abscess, furuncle and c..	186	0.70
O86.1 Other infection of genital trac..	163	0.61
L03.1 Cellulitis of other parts of limb	157	0.59
L05.0 Pilonidal cyst with abscess	150	0.56
L02.2 Cutaneous abscess, furuncle and..	144	0.54
O86.4 Pyrexia of unknown origin follo..	141	0.53
K05.2 Acute periodontitis	140	0.53
L04.0 Acute lymphadenitis of face, he..	127	0.48
L02.3 Cutaneous abscess, furuncle and..	119	0.45
L03.9 Cellulitis, unspecified	116	0.44
A04.9 Bacterial intestinal infection,..	113	0.42
K02 Dental caries	113	0.42
O23.4 Unspecified infection of urinar..	104	0.39
H10.0 Mucopurulent conjunctivitis	102	0.38
O23.1 Infections of bladder in pregna..	101	0.38
H60.3 Other infective otitis externa	96	0.36
O86.2 Urinary tract infection followi..	95	0.36
L08.8 Other specified local infection..	92	0.35
N41.0 Acute prostatitis	91	0.34
A54.9 Gonococcal infection, unspecified	89	0.33

H00.1 Chalazion	87	0.33
A54.2 Gonococcal pelviperitonitis and..	86	0.32
L02.9 Cutaneous abscess, furuncle and..	82	0.31
O98.8 Other maternal infectious and p..	80	0.30
A04.8 Other specified bacterial intes..	77	0.29
O86.8 Other specified puerperal infec..	77	0.29
K04.6 Periapical abscess with sinus	74	0.28
L02.1 Cutaneous abscess, furuncle and..	69	0.26
J20.0 Acute bronchitis due to Mycopla..	64	0.24
O98.3 Other infections with a predomi..	53	0.20
H00.0 Hordeolum and other deep inflam..	52	0.20
L02.0 Cutaneous abscess, furuncle and..	52	0.20
A56.0 Chlamydial infection of lower g..	51	0.19
J02.0 Streptococcal pharyngitis	51	0.19
K02.9 Dental caries, unspecified	49	0.18
L03.2 Cellulitis of face	48	0.18
L08.0 Pyoderma	48	0.18
L02.8 Cutaneous abscess, furuncle and..	47	0.18
A04.6 Enteritis due to Yersinia enter..	44	0.17
J04.2 Acute laryngotracheitis	43	0.16
L03.3 Cellulitis of trunk	39	0.15
A37 Whooping cough	32	0.12
A05.9 Bacterial foodborne intoxicatio..	31	0.12
N76.0 Acute vaginitis	31	0.12
O98.9 Unspecified maternal infectious..	30	0.11
L01.0 Impetigo [any organism] [any si..	26	0.10
K11.3 Abscess of salivary gland	25	0.09
O86.3 Other genitourinary tract infec..	25	0.09
K04.4 Acute apical periodontitis of p..	24	0.09
L03.8 Cellulitis of other sites	23	0.09
A54.1 Gonococcal infection of lower g..	22	0.08
J04.1 Acute tracheitis	20	0.08
O91.0 Infection of nipple associated ..	18	0.07
J03.8 Acute tonsillitis due to other ..	17	0.06
A64 Unspecified sexually transmitted ..	14	0.05
L04.9 Acute lymphadenitis, unspecified	14	0.05
L01 Impetigo	12	0.05
L70.1 Acne conglobata	12	0.05
K02.8 Other dental caries	11	0.04
K05.0 Acute gingivitis	11	0.04
A54.0 Gonococcal infection of lower g..	10	0.04
L01.1 Impetiginization of other derma..	10	0.04
A04.3 Enterohaemorrhagic Escherichia ..	9	0.03
J20.1 Acute bronchitis due to Haemoph..	9	0.03
H10.5 Blepharoconjunctivitis	7	0.03
L04.3 Acute lymphadenitis of lower limb	7	0.03
L04.8 Acute lymphadenitis of other si..	6	0.02
O23.3 Infections of other parts of ur..	6	0.02

A04.0 Enteropathogenic Escherichia co..	5	0.02
A05.0 Foodborne staphylococcal intoxi..	5	0.02
A05.8 Other specified bacterial foodb..	5	0.02
H01.0 Blepharitis	5	0.02
L04.2 Acute lymphadenitis of upper limb	5	0.02
A00.9 Cholera, unspecified	<5	<0.02
A04 Other bacterial intestinal infect..	<5	<0.02
A04.1 Enterotoxigenic Escherichia col..	<5	<0.02
A04.2 Enteroinvasive Escherichia coli..	<5	<0.02
A04.4 Other intestinal Escherichia co..	<5	<0.02
A05.1 Botulism	<5	<0.02
A05.2 Foodborne Clostridium perfringe..	<5	<0.02
A26.0 Cutaneous erysipeloid	<5	<0.02
A26.9 Erysipeloid, unspecified	<5	<0.02
A37.0 Whooping cough due to Bordetell..	<5	<0.02
A37.9 Whooping cough, unspecified	<5	<0.02
A54 Gonococcal infection	<5	<0.02
A54.3 Gonococcal infection of eye	<5	<0.02
A54.4 Gonococcal infection of musculo..	<5	<0.02
A54.8 Other gonococcal infections	<5	<0.02
A56.3 Chlamydial infection of anus an..	<5	<0.02
A58 Granuloma inguinale	<5	<0.02
A71 Trachoma	<5	<0.02
J02.8 Acute pharyngitis due to other ..	<5	<0.02
J03 Acute tonsillitis	<5	<0.02
J20.2 Acute bronchitis due to strepto..	<5	<0.02
K02.0 Caries limited to enamel	<5	<0.02
K02.2 Caries of cementum	<5	<0.02
K02.3 Arrested dental caries	<5	<0.02
L00 Staphylococcal scalded skin syndr..	<5	<0.02
L04.1 Acute lymphadenitis of trunk	<5	<0.02
L08.1 Erythrasma	<5	<0.02
O23.2 Infections of urethra in pregna..	<5	<0.02
P39.1 Neonatal conjunctivitis and dac..	<5	<0.02
**Total**	**26 601**	**100**

eTable 11. Distribution of bacterial infections with sepsis in primary analysis (Finnish multicohort sample)

ICD-10 code and description	N	%
A41.5 Sepsis due to other Gram-negati..	850	29.11
A41.9 Sepsis, unspecified	619	21.20
A41.0 Sepsis due to Staphylococcus au..	381	13.05
A41.8 Other specified sepsis	269	9.21
A40.3 Sepsis due to Streptococcus pne..	256	8.77
A40.8 Other streptococcal sepsis	112	3.84
A40.0 Sepsis due to streptococcus, gr..	85	2.91
A40.9 Streptococcal sepsis, unspecified	74	2.53

A41.1 Sepsis due to other specified s..	72	2.47
A02.1 Salmonella sepsis	41	1.40
A40.1 Sepsis due to streptococcus, gr..	40	1.37
A41.2 Sepsis due to unspecified staph..	37	1.27
A41.4 Sepsis due to anaerobes	26	0.89
A39.2 Acute meningococcaemia	14	0.48
A32.7 Listerial sepsis	13	0.45
A39.4 Meningococcaemia, unspecified	10	0.34
A41.3 Sepsis due to Haemophilus influ..	7	0.24
A21.7 Generalized tularaemia	<5	<0.17
A39.1 Waterhouse-Friderichsen syndrome	<5	<0.17
A40 Streptococcal sepsis	<5	<0.17
A40.2 Sepsis due to streptococcus, gr..	<5	<0.17
A41 Other sepsis	<5	<0.17
A42.7 Actinomycotic sepsis	<5	<0.17
A48.3 Toxic shock syndrome	<5	<0.17
P36.9 Bacterial sepsis of newborn, un..	<5	<0.17
**Total**	**2 920**	**100**

eTable 12. Distribution of bacterial infections without sepsis in primary analysis (Finnish multicohort sample)

ICD-10 code and description	N	%
K35 Acute appendicitis	12 215	17.92
J18.9 Pneumonia, unspecified	6530	9.58
A09 Other gastroenteritis and colitis..	5950	8.73
N10 Acute tubulo-interstitial nephritis	3844	5.64
J03.9 Acute tonsillitis, unspecified	3310	4.86
A46 Erysipelas	3274	4.80
J36 Peritonsillar abscess	3151	4.62
J15.9 Bacterial pneumonia, unspecified	2402	3.52
N70.0 Acute salpingitis and oophoritis	1992	2.92
O85 Puerperal sepsis	1763	2.59
J01.0 Acute maxillary sinusitis	1146	1.68
N39.0 Urinary tract infection, site n..	1131	1.66
A49.9 Bacterial infection, unspecified	1124	1.65
J03.0 Streptococcal tonsillitis	1011	1.48
J22 Unspecified acute lower respirato..	804	1.18
N71.0 Acute inflammatory disease of u..	739	1.08
J02.9 Acute pharyngitis, unspecified	733	1.08
J04.0 Acute laryngitis	704	1.03
K57.2 Diverticular disease of large i..	467	0.69
N30.0 Acute cystitis	462	0.68
J18.0 Bronchopneumonia, unspecified	458	0.67
L05.9 Pilonidal cyst without abscess	418	0.61
O08.0 Genital tract and pelvic infect..	379	0.56
O03.5 Spontaneous abortion: Complete ..	370	0.54
K05.3 Chronic periodontitis	349	0.51

K61.0 Anal abscess	349	0.51
A04.7 Enterocolitis due to Clostridiu..	338	0.50
H66.0 Acute suppurative otitis media	326	0.48
J13 Pneumonia due to Streptococcus pn..	313	0.46
N75.1 Abscess of Bartholin gland	309	0.45
K04.7 Periapical abscess without sinus	275	0.40
N61 Inflammatory disorders of breast	267	0.39
A69.2 Lyme disease	249	0.37
L04 Acute lymphadenitis	236	0.35
M00.9 Pyogenic arthritis, unspecified	223	0.33
N45.9 Orchitis, epididymitis and epid..	218	0.32
L03.0 Cellulitis of finger and toe	214	0.31
K04.5 Chronic apical periodontitis	213	0.31
A04.5 Campylobacter enteritis	208	0.31
K65.0 Acute peritonitis	193	0.28
O23.0 Infections of kidney in pregnancy	184	0.27
K12.2 Cellulitis and abscess of mouth	181	0.27
K02.1 Caries of dentine	180	0.26
K61 Abscess of anal and rectal regions	180	0.26
O23.5 Infections of the genital tract..	180	0.26
O03.0 Spontaneous abortion: Incomplet..	179	0.26
J05.1 Acute epiglottitis	176	0.26
L08.9 Local infection of skin and sub..	176	0.26
L02 Cutaneous abscess, furuncle and c..	171	0.25
O86.0 Infection of obstetric surgical..	171	0.25
A02.0 Salmonella enteritis	168	0.25
O23.9 Other and unspecified genitouri..	162	0.24
L02.4 Cutaneous abscess, furuncle and..	156	0.23
J15.8 Other bacterial pneumonia	153	0.22
O86.1 Other infection of genital trac..	139	0.20
A16.2 Tuberculosis of lung, without m..	136	0.20
G00.9 Bacterial meningitis, unspecified	130	0.19
L05.0 Pilonidal cyst with abscess	130	0.19
O86.4 Pyrexia of unknown origin follo..	126	0.18
K05.2 Acute periodontitis	125	0.18
J18.1 Lobar pneumonia, unspecified	119	0.17
A02 Other salmonella infections	112	0.16
K02 Dental caries	108	0.16
L04.0 Acute lymphadenitis of face, he..	103	0.15
L03.1 Cellulitis of other parts of limb	102	0.15
A56.1 Chlamydial infection of pelvipe..	100	0.15
L02.2 Cutaneous abscess, furuncle and..	98	0.14
J15.7 Pneumonia due to Mycoplasma pne..	96	0.14
O04.0 Medical abortion: Incomplete, c..	94	0.14
O23.4 Unspecified infection of urinar..	93	0.14
A49.8 Other bacterial infections of u..	92	0.13
J86.9 Pyothorax without fistula	92	0.13
A04.9 Bacterial intestinal infection,..	91	0.13

I33.0 Acute and subacute infective en..	91	0.13
H10.0 Mucopurulent conjunctivitis	88	0.13
O23.1 Infections of bladder in pregna..	85	0.12
H00.1 Chalazion	84	0.12
L02.3 Cutaneous abscess, furuncle and..	84	0.12
A15.0 Tuberculosis of lung, confirmed..	81	0.12
M71.1 Other infective bursitis	81	0.12
H60.3 Other infective otitis externa	79	0.12
J16.8 Pneumonia due to other specifie..	79	0.12
O86.2 Urinary tract infection followi..	78	0.11
L03.9 Cellulitis, unspecified	76	0.11
A54.9 Gonococcal infection, unspecified	75	0.11
L08.8 Other specified local infection..	74	0.11
N41.0 Acute prostatitis	74	0.11
M86.9 Osteomyelitis, unspecified	72	0.11
N76.4 Abscess of vulva	71	0.10
J39.0 Retropharyngeal and parapharyng..	70	0.10
O04.5 Medical abortion: Complete or u..	68	0.10
K04.6 Periapical abscess with sinus	67	0.10
K57.0 Diverticular disease of small i..	67	0.10
M86.1 Other acute osteomyelitis	64	0.09
O86.8 Other specified puerperal infec..	63	0.09
A04.8 Other specified bacterial intes..	62	0.09
O75.3 Other infection during labour	62	0.09
O98.8 Other maternal infectious and p..	62	0.09
A54.2 Gonococcal pelviperitonitis and..	58	0.09
H62.0 Otitis externa in bacterial dis..	58	0.09
L02.1 Cutaneous abscess, furuncle and..	57	0.08
L02.9 Cutaneous abscess, furuncle and..	56	0.08
A74.9 Chlamydial infection, unspecified	55	0.08
N73.3 Female acute pelvic peritonitis	53	0.08
A38 Scarlet fever	51	0.07
O91.1 Abscess of breast associated wi..	51	0.07
H00.0 Hordeolum and other deep inflam..	47	0.07
H05.0 Acute inflammation of orbit	46	0.07
A56.0 Chlamydial infection of lower g..	45	0.07
J20.0 Acute bronchitis due to Mycopla..	44	0.06
O98.3 Other infections with a predomi..	43	0.06
J02.0 Streptococcal pharyngitis	42	0.06
J18.8 Other pneumonia, organism unspe..	42	0.06
L03.2 Cellulitis of face	42	0.06
A18.2 Tuberculous peripheral lymphade..	41	0.06
G00.8 Other bacterial meningitis	41	0.06
L02.0 Cutaneous abscess, furuncle and..	41	0.06
K02.9 Dental caries, unspecified	40	0.06
K61.1 Rectal abscess	40	0.06
M71.0 Abscess of bursa	39	0.06
L08.0 Pyoderma	38	0.06

A49.0 Staphylococcal infection, unspe..	37	0.05
J04.2 Acute laryngotracheitis	37	0.05
A04.6 Enteritis due to Yersinia enter..	35	0.05
L02.8 Cutaneous abscess, furuncle and..	35	0.05
H60.1 Cellulitis of external ear	33	0.05
H70.0 Acute mastoiditis	33	0.05
M86.6 Other chronic osteomyelitis	33	0.05
A39.0 Meningococcal meningitis	32	0.05
M00.0 Staphylococcal arthritis and po..	31	0.05
A16.5 Tuberculous pleurisy, without m..	30	0.04
G00.0 Haemophilus meningitis	30	0.04
G00.1 Pneumococcal meningitis	30	0.04
H60.0 Abscess of external ear	30	0.04
K75.0 Abscess of liver	29	0.04
N73.0 Acute parametritis and pelvic c..	29	0.04
A37 Whooping cough	28	0.04
J85.2 Abscess of lung without pneumonia	28	0.04
K63.0 Abscess of intestine	27	0.04
H44.0 Purulent endophthalmitis	26	0.04
A05.9 Bacterial foodborne intoxicatio..	25	0.04
J86.0 Pyothorax with fistula	25	0.04
L03.3 Cellulitis of trunk	24	0.04
O86.3 Other genitourinary tract infec..	23	0.03
O98.9 Unspecified maternal infectious..	23	0.03
A21.9 Tularaemia, unspecified	22	0.03
G06.0 Intracranial abscess and granul..	22	0.03
K11.3 Abscess of salivary gland	22	0.03
M65.0 Abscess of tendon sheath	22	0.03
A18.0 Tuberculosis of bones and joints	21	0.03
A21.0 Ulceroglandular tularaemia	21	0.03
G01 Meningitis in bacterial diseases ..	21	0.03
K61.2 Anorectal abscess	21	0.03
N76.0 Acute vaginitis	21	0.03
A54.1 Gonococcal infection of lower g..	20	0.03
K04.4 Acute apical periodontitis of p..	20	0.03
M46.3 Infection of intervertebral dis..	20	0.03
A48.8 Other specified bacterial disea..	19	0.03
N15.1 Renal and perinephric abscess	19	0.03
G06.1 Intraspinal abscess and granuloma	18	0.03
J04.1 Acute tracheitis	18	0.03
J86 Pyothorax	18	0.03
N74.4 Female chlamydial pelvic inflam..	18	0.03
A02.9 Salmonella infection, unspecified	17	0.02
A15.1 Tuberculosis of lung, confirmed..	16	0.02
L01.0 Impetigo [any organism] [any si..	16	0.02
N45.0 Orchitis, epididymitis and epid..	16	0.02
A51.0 Primary genital syphilis	15	0.02
J03.8 Acute tonsillitis due to other ..	15	0.02

L03.8 Cellulitis of other sites	15	0.02
O91.0 Infection of nipple associated ..	15	0.02
A01.4 Paratyphoid fever, unspecified	14	0.02
A03.3 Shigellosis due to Shigella son..	14	0.02
A51.3 Secondary syphilis of skin and ..	14	0.02
K57.4 Diverticular disease of both sm..	14	0.02
N34.0 Urethral abscess	14	0.02
J85.1 Abscess of lung with pneumonia	13	0.02
A64 Unspecified sexually transmitted ..	12	0.02
L01 Impetigo	12	0.02
L70.1 Acne conglobata	12	0.02
O06.5 Unspecified abortion: Complete ..	12	0.02
A01.2 Paratyphoid fever B	11	0.02
J14 Pneumonia due to Haemophilus infl..	11	0.02
L04.9 Acute lymphadenitis, unspecified	11	0.02
M00.2 Other streptococcal arthritis a..	11	0.02
M46.2 Osteomyelitis of vertebra	11	0.02
A03.1 Shigellosis due to Shigella fle..	10	0.01
A49.1 Streptococcal infection, unspec..	10	0.01
J16.0 Chlamydial pneumonia	10	0.01
K61.3 Ischiorectal abscess	10	0.01
L01.1 Impetiginization of other derma..	10	0.01
M01.2 Arthritis in Lyme disease	10	0.01
N13.6 Pyonephrosis	10	0.01
A02.8 Other specified salmonella infe..	9	0.01
J39.1 Other abscess of pharynx	9	0.01
A01.0 Typhoid fever	8	0.01
A02.2 Localized salmonella infections	8	0.01
A15.6 Tuberculous pleurisy, confirmed..	8	0.01
A16.0 Tuberculosis of lung, bacteriol..	8	0.01
A16.9 Respiratory tuberculosis unspec..	8	0.01
A18.1 Tuberculosis of genitourinary s..	8	0.01
A31.0 Pulmonary mycobacterial infection	8	0.01
A32.8 Other forms of listeriosis	8	0.01
J15.2 Pneumonia due to staphylococcus	8	0.01
K02.8 Other dental caries	8	0.01
O07.0 Failed medical abortion, compli..	8	0.01
O07.5 Other and unspecified failed at..	8	0.01
A04.3 Enterohaemorrhagic Escherichia ..	7	0.01
A18.3 Tuberculosis of intestines, per..	7	0.01
A74.8 Other chlamydial diseases	7	0.01
J20.1 Acute bronchitis due to Haemoph..	7	0.01
K05.0 Acute gingivitis	7	0.01
M65.1 Other infective (teno)synovitis	7	0.01
O05.0 Other abortion: Incomplete, com..	7	0.01
O05.5 Other abortion: Complete or uns..	7	0.01
O06.0 Unspecified abortion: Incomplet..	7	0.01
A31 Infection due to other mycobacteria	6	0.01

A48.0 Gas gangrene	6	0.01
A52.1 Symptomatic neurosyphilis	6	0.01
A54.0 Gonococcal infection of lower g..	6	0.01
B95.6 Staphylococcus aureus as the ca..	6	0.01
B96.2 Escherichia coli [E. coli] as t..	6	0.01
H10.5 Blepharoconjunctivitis	6	0.01
J15.0 Pneumonia due to Klebsiella pne..	6	0.01
J85.3 Abscess of mediastinum	6	0.01
A03.9 Shigellosis, unspecified	5	0.01
A15.2 Tuberculosis of lung, confirmed..	5	0.01
A18.4 Tuberculosis of skin and subcut..	5	0.01
A31.9 Mycobacterial infection, unspec..	5	0.01
B95.8 Unspecified staphylococcus as t..	5	0.01
G05.0 Encephalitis, myelitis and ence..	5	0.01
J15.4 Pneumonia due to other streptoc..	5	0.01
K57.8 Diverticular disease of intesti..	5	0.01
L04.2 Acute lymphadenitis of upper limb	5	0.01
L04.8 Acute lymphadenitis of other si..	5	0.01
M01.3 Arthritis in other bacterial di..	5	0.01
M86.0 Acute haematogenous osteomyelitis	5	0.01
M86.2 Subacute osteomyelitis	5	0.01
A00.9 Cholera, unspecified	<5	<0.01
A01.1 Paratyphoid fever A	<5	<0.01
A01.3 Paratyphoid fever C	<5	<0.01
A03 Shigellosis	<5	<0.01
A03.0 Shigellosis due to Shigella dys..	<5	<0.01
A03.2 Shigellosis due to Shigella boy..	<5	<0.01
A04 Other bacterial intestinal infect..	<5	<0.01
A04.0 Enteropathogenic Escherichia co..	<5	<0.01
A04.1 Enterotoxigenic Escherichia col..	<5	<0.01
A04.2 Enteroinvasive Escherichia coli..	<5	<0.01
A04.4 Other intestinal Escherichia co..	<5	<0.01
A05.0 Foodborne staphylococcal intoxi..	<5	<0.01
A05.1 Botulism	<5	<0.01
A05.2 Foodborne Clostridium perfringe..	<5	<0.01
A05.8 Other specified bacterial foodb..	<5	<0.01
A15.3 Tuberculosis of lung, confirmed..	<5	<0.01
A15.4 Tuberculosis of intrathoracic l..	<5	<0.01
A15.5 Tuberculosis of larynx, trachea..	<5	<0.01
A15.7 Primary respiratory tuberculosi..	<5	<0.01
A15.8 Other respiratory tuberculosis,..	<5	<0.01
A15.9 Respiratory tuberculosis unspec..	<5	<0.01
A16.1 Tuberculosis of lung, bacteriol..	<5	<0.01
A16.3 Tuberculosis of intrathoracic l..	<5	<0.01
A16.4 Tuberculosis of larynx, trachea..	<5	<0.01
A16.7 Primary respiratory tuberculosi..	<5	<0.01
A16.8 Other respiratory tuberculosis,..	<5	<0.01
A17.0 Tuberculous meningitis	<5	<0.01

A17.9 Tuberculosis of nervous system,..	<5	<0.01
A18.8 Tuberculosis of other specified..	<5	<0.01
A19.0 Acute miliary tuberculosis of a..	<5	<0.01
A19.2 Acute miliary tuberculosis, uns..	<5	<0.01
A19.9 Miliary tuberculosis, unspecified	<5	<0.01
A20.9 Plague, unspecified	<5	<0.01
A21.1 Oculoglandular tularaemia	<5	<0.01
A21.2 Pulmonary tularaemia	<5	<0.01
A21.8 Other forms of tularaemia	<5	<0.01
A22.0 Cutaneous anthrax	<5	<0.01
A22.2 Gastrointestinal anthrax	<5	<0.01
A23.9 Brucellosis, unspecified	<5	<0.01
A24.0 Glanders	<5	<0.01
A26.0 Cutaneous erysipeloid	<5	<0.01
A26.9 Erysipeloid, unspecified	<5	<0.01
A27 Leptospirosis	<5	<0.01
A27.0 Leptospirosis icterohaemorrhagica	<5	<0.01
A27.8 Other forms of leptospirosis	<5	<0.01
A27.9 Leptospirosis, unspecified	<5	<0.01
A28.2 Extraintestinal yersiniosis	<5	<0.01
A28.8 Other specified zoonotic bacter..	<5	<0.01
A28.9 Zoonotic bacterial disease, uns..	<5	<0.01
A30 Leprosy [Hansen disease]	<5	<0.01
A31.1 Cutaneous mycobacterial infection	<5	<0.01
A31.8 Other mycobacterial infections	<5	<0.01
A32.9 Listeriosis, unspecified	<5	<0.01
A35 Other tetanus	<5	<0.01
A36 Diphtheria	<5	<0.01
A37.0 Whooping cough due to Bordetell..	<5	<0.01
A37.9 Whooping cough, unspecified	<5	<0.01
A39 Meningococcal infection	<5	<0.01
A39.8 Other meningococcal infections	<5	<0.01
A39.9 Meningococcal infection, unspec..	<5	<0.01
A42 Actinomycosis	<5	<0.01
A42.0 Pulmonary actinomycosis	<5	<0.01
A42.1 Abdominal actinomycosis	<5	<0.01
A42.2 Cervicofacial actinomycosis	<5	<0.01
A42.8 Other forms of actinomycosis	<5	<0.01
A42.9 Actinomycosis, unspecified	<5	<0.01
A44.1 Cutaneous and mucocutaneous bar..	<5	<0.01
A49 Bacterial infection of unspecifie..	<5	<0.01
A49.2 Haemophilus influenzae infectio..	<5	<0.01
A49.3 Mycoplasma infection, unspecifi..	<5	<0.01
A50 Congenital syphilis	<5	<0.01
A50.5 Other late congenital syphilis,..	<5	<0.01
A50.9 Congenital syphilis, unspecified	<5	<0.01
A51.2 Primary syphilis of other sites	<5	<0.01
A51.4 Other secondary syphilis	<5	<0.01

A51.5 Early syphilis, latent	<5	<0.01
A51.9 Early syphilis, unspecified	<5	<0.01
A52.0 Cardiovascular syphilis	<5	<0.01
A52.7 Other symptomatic late syphilis	<5	<0.01
A52.8 Late syphilis, latent	<5	<0.01
A53 Other and unspecified syphilis	<5	<0.01
A53.9 Syphilis, unspecified	<5	<0.01
A54 Gonococcal infection	<5	<0.01
A54.3 Gonococcal infection of eye	<5	<0.01
A54.4 Gonococcal infection of musculo..	<5	<0.01
A54.8 Other gonococcal infections	<5	<0.01
A55 Chlamydial lymphogranuloma (vener..	<5	<0.01
A56.2 Chlamydial infection of genitou..	<5	<0.01
A56.3 Chlamydial infection of anus an..	<5	<0.01
A56.8 Sexually transmitted chlamydial..	<5	<0.01
A58 Granuloma inguinale	<5	<0.01
A66.1 Multiple papillomata and wet cr..	<5	<0.01
A66.3 Hyperkeratosis of yaws	<5	<0.01
A67 Pinta [carate]	<5	<0.01
A67.1 Intermediate lesions of pinta	<5	<0.01
A68 Relapsing fevers	<5	<0.01
A69.0 Necrotizing ulcerative stomatitis	<5	<0.01
A69.1 Other Vincent infections	<5	<0.01
A69.9 Spirochaetal infection, unspeci..	<5	<0.01
A70 Chlamydia psittaci infection	<5	<0.01
A71 Trachoma	<5	<0.01
A75.0 Epidemic louse-borne typhus fev..	<5	<0.01
A75.9 Typhus fever, unspecified	<5	<0.01
A77.1 Spotted fever due to Rickettsia..	<5	<0.01
A77.8 Other spotted fevers	<5	<0.01
A78 Q fever	<5	<0.01
A79.9 Rickettsiosis, unspecified	<5	<0.01
B95.0 Streptococcus, group A, as the ..	<5	<0.01
B95.1 Streptococcus, group B, as the ..	<5	<0.01
B95.3 Streptococcus pneumoniae as the..	<5	<0.01
B95.4 Other streptococcus as the caus..	<5	<0.01
B95.5 Unspecified streptococcus as th..	<5	<0.01
B95.7 Other staphylococcus as the cau..	<5	<0.01
B96.1 Klebsiella pneumoniae [K. pneum..	<5	<0.01
B96.3 Haemophilus influenzae [H. infl..	<5	<0.01
B96.5 Pseudomonas (aeruginosa) as the..	<5	<0.01
B96.7 Clostridium perfringens [C. per..	<5	<0.01
B96.8 Other specified bacterial agent..	<5	<0.01
D73.3 Abscess of spleen	<5	<0.01
G00.2 Streptococcal meningitis	<5	<0.01
G00.3 Staphylococcal meningitis	<5	<0.01
G04.2 Bacterial meningoencephalitis a..	<5	<0.01
G06 Intracranial and intraspinal absc..	<5	<0.01

G06.2 Extradural and subdural abscess..	<5	<0.01
H01.0 Blepharitis	<5	<0.01
H60.2 Malignant otitis externa	<5	<0.01
I32.0 Pericarditis in bacterial disea..	<5	<0.01
I41.0 Myocarditis in bacterial diseas..	<5	<0.01
J02.8 Acute pharyngitis due to other ..	<5	<0.01
J03 Acute tonsillitis	<5	<0.01
J15.1 Pneumonia due to Pseudomonas	<5	<0.01
J15.3 Pneumonia due to streptococcus,..	<5	<0.01
J15.5 Pneumonia due to Escherichia coli	<5	<0.01
J15.6 Pneumonia due to other aerobic ..	<5	<0.01
J17.0 Pneumonia in bacterial diseases..	<5	<0.01
J17.8 Pneumonia in other diseases cla..	<5	<0.01
J18.2 Hypostatic pneumonia, unspecified	<5	<0.01
J20.2 Acute bronchitis due to strepto..	<5	<0.01
K02.0 Caries limited to enamel	<5	<0.01
K02.2 Caries of cementum	<5	<0.01
K02.3 Arrested dental caries	<5	<0.01
K23.0 Tuberculous oesophagitis	<5	<0.01
K61.4 Intrasphincteric abscess	<5	<0.01
K67.3 Tuberculous peritonitis	<5	<0.01
L00 Staphylococcal scalded skin syndr..	<5	<0.01
L04.1 Acute lymphadenitis of trunk	<5	<0.01
L04.3 Acute lymphadenitis of lower limb	<5	<0.01
L08.1 Erythrasma	<5	<0.01
M00.8 Arthritis and polyarthritis due..	<5	<0.01
M01.0 Meningococcal arthritis	<5	<0.01
M01.1 Tuberculous arthritis	<5	<0.01
M46.5 Other infective spondylopathies	<5	<0.01
M49.0 Tuberculosis of spine	<5	<0.01
M49.1 Brucella spondylitis	<5	<0.01
M63.0 Myositis in bacterial diseases ..	<5	<0.01
M72.6 Necrotizing fasciitis	<5	<0.01
M86.3 Chronic multifocal osteomyelitis	<5	<0.01
M86.4 Chronic osteomyelitis with drai..	<5	<0.01
M86.8 Other osteomyelitis	<5	<0.01
O23.2 Infections of urethra in pregna..	<5	<0.01
O23.3 Infections of other parts of ur..	<5	<0.01
O98.0 Tuberculosis complicating pregn..	<5	<0.01
O98.1 Syphilis complicating pregnancy..	<5	<0.01
P39.0 Neonatal infective mastitis	<5	<0.01
P39.1 Neonatal conjunctivitis and dac..	<5	<0.01
**Total**	**68 152**	**100**

eTable 13. Distribution of extracellular bacterial infections in primary analysis (Finnish multicohort sample)

ICD-10 code and description	N	%

J03.0 Streptococcal tonsillitis	1336	27.52
A04.7 Enterocolitis due to Clostridiu..	666	13.72
J13 Pneumonia due to Streptococcus pn..	411	8.47
A41.0 Sepsis due to Staphylococcus au..	378	7.79
A69.2 Lyme disease	343	7.06
A40.3 Sepsis due to Streptococcus pne..	200	4.12
A40.8 Other streptococcal sepsis	124	2.55
A54.9 Gonococcal infection, unspecified	91	1.87
A54.2 Gonococcal pelviperitonitis and..	88	1.81
A40.0 Sepsis due to streptococcus, gr..	82	1.69
A41.1 Sepsis due to other specified s..	79	1.63
A40.9 Streptococcal sepsis, unspecified	73	1.50
J02.0 Streptococcal pharyngitis	62	1.28
A49.0 Staphylococcal infection, unspe..	58	1.19
A38 Scarlet fever	56	1.15
A39.0 Meningococcal meningitis	47	0.97
M00.0 Staphylococcal arthritis and po..	42	0.87
A37 Whooping cough	41	0.84
A40.1 Sepsis due to streptococcus, gr..	39	0.80
A41.2 Sepsis due to unspecified staph..	38	0.78
B96.2 Escherichia coli [E. coli] as t..	36	0.74
G00.0 Haemophilus meningitis	33	0.68
L01.0 Impetigo [any organism] [any si..	30	0.62
G00.1 Pneumococcal meningitis	25	0.51
A54.1 Gonococcal infection of lower g..	22	0.45
J14 Pneumonia due to Haemophilus infl..	18	0.37
A51.0 Primary genital syphilis	17	0.35
A52.1 Symptomatic neurosyphilis	17	0.35
B95.6 Staphylococcus aureus as the ca..	17	0.35
A51.3 Secondary syphilis of skin and ..	16	0.33
A48.0 Gas gangrene	14	0.29
B95.8 Unspecified staphylococcus as t..	14	0.29
A39.2 Acute meningococcaemia	13	0.27
J15.0 Pneumonia due to Klebsiella pne..	13	0.27
L01 Impetigo	13	0.27
M00.2 Other streptococcal arthritis a..	13	0.27
J20.1 Acute bronchitis due to Haemoph..	12	0.25
L01.1 Impetiginization of other derma..	12	0.25
A04.3 Enterohaemorrhagic Escherichia ..	11	0.23
M01.2 Arthritis in Lyme disease	11	0.23
A54.0 Gonococcal infection of lower g..	10	0.21
B96.5 Pseudomonas (aeruginosa) as the..	10	0.21
A39.4 Meningococcaemia, unspecified	9	0.19
B95.5 Unspecified streptococcus as th..	9	0.19
B95.4 Other streptococcus as the caus..	8	0.16
J15.2 Pneumonia due to staphylococcus	8	0.16
J15.4 Pneumonia due to other streptoc..	8	0.16
A04.0 Enteropathogenic Escherichia co..	7	0.14

J15.1 Pneumonia due to Pseudomonas	7	0.14
A05.0 Foodborne staphylococcal intoxi..	6	0.12
B95.3 Streptococcus pneumoniae as the..	6	0.12
G00.3 Staphylococcal meningitis	6	0.12
J15.3 Pneumonia due to streptococcus,..	6	0.12
A04.4 Other intestinal Escherichia co..	5	0.10
A37.9 Whooping cough, unspecified	5	0.10
A51.5 Early syphilis, latent	5	0.10
A51.9 Early syphilis, unspecified	5	0.10
A53.9 Syphilis, unspecified	5	0.10
B95.0 Streptococcus, group A, as the ..	5	0.10
G00.2 Streptococcal meningitis	5	0.10
J15.5 Pneumonia due to Escherichia coli	5	0.10
A00.9 Cholera, unspecified	<5	<0.10
A04.1 Enterotoxigenic Escherichia col..	<5	<0.10
A05.1 Botulism	<5	<0.10
A05.2 Foodborne Clostridium perfringe..	<5	<0.10
A22.0 Cutaneous anthrax	<5	<0.10
A22.2 Gastrointestinal anthrax	<5	<0.10
A25.9 Rat-bite fever, unspecified	<5	<0.10
A27 Leptospirosis	<5	<0.10
A27.0 Leptospirosis icterohaemorrhagica	<5	<0.10
A27.8 Other forms of leptospirosis	<5	<0.10
A27.9 Leptospirosis, unspecified	<5	<0.10
A28.0 Pasteurellosis	<5	<0.10
A35 Other tetanus	<5	<0.10
A36 Diphtheria	<5	<0.10
A37.0 Whooping cough due to Bordetell..	<5	<0.10
A39 Meningococcal infection	<5	<0.10
A39.1 Waterhouse-Friderichsen syndrome	<5	<0.10
A39.5 Meningococcal heart disease	<5	<0.10
A39.8 Other meningococcal infections	<5	<0.10
A39.9 Meningococcal infection, unspec..	<5	<0.10
A40 Streptococcal sepsis	<5	<0.10
A40.2 Sepsis due to streptococcus, gr..	<5	<0.10
A41.3 Sepsis due to Haemophilus influ..	<5	<0.10
A48.3 Toxic shock syndrome	<5	<0.10
A49.2 Haemophilus influenzae infectio..	<5	<0.10
A50 Congenital syphilis	<5	<0.10
A50.5 Other late congenital syphilis,..	<5	<0.10
A50.9 Congenital syphilis, unspecified	<5	<0.10
A51.1 Primary anal syphilis	<5	<0.10
A51.2 Primary syphilis of other sites	<5	<0.10
A51.4 Other secondary syphilis	<5	<0.10
A52.0 Cardiovascular syphilis	<5	<0.10
A52.7 Other symptomatic late syphilis	<5	<0.10
A52.8 Late syphilis, latent	<5	<0.10
A53 Other and unspecified syphilis	<5	<0.10

A54 Gonococcal infection	<5	<0.10
A54.3 Gonococcal infection of eye	<5	<0.10
A54.4 Gonococcal infection of musculo..	<5	<0.10
A54.6 Gonococcal infection of anus an..	<5	<0.10
A54.8 Other gonococcal infections	<5	<0.10
A66.1 Multiple papillomata and wet cr..	<5	<0.10
A66.3 Hyperkeratosis of yaws	<5	<0.10
A67 Pinta [carate]	<5	<0.10
A67.1 Intermediate lesions of pinta	<5	<0.10
A68 Relapsing fevers	<5	<0.10
A68.9 Relapsing fever, unspecified	<5	<0.10
A69.9 Spirochaetal infection, unspeci..	<5	<0.10
B95.1 Streptococcus, group B, as the ..	<5	<0.10
B95.7 Other staphylococcus as the cau..	<5	<0.10
B96.1 Klebsiella pneumoniae [K. pneum..	<5	<0.10
B96.3 Haemophilus influenzae [H. infl..	<5	<0.10
B96.7 Clostridium perfringens [C. per..	<5	<0.10
J20.2 Acute bronchitis due to strepto..	<5	<0.10
L00 Staphylococcal scalded skin syndr..	<5	<0.10
M00.1 Pneumococcal arthritis and poly..	<5	<0.10
M01.0 Meningococcal arthritis	<5	<0.10
M73.0 Gonococcal bursitis	<5	<0.10
N74.3 Female gonococcal pelvic inflam..	<5	<0.10
O98.1 Syphilis complicating pregnancy..	<5	<0.10
**Total**	**4 855**	**100**

eTable 14. Distribution of intracellular bacterial infections in primary analysis (Finnish multicohort sample)

ICD-10 code and description	N	%
A04.5 Campylobacter enteritis	289	13.47
A02.0 Salmonella enteritis	230	10.72
A16.2 Tuberculosis of lung, without m..	152	7.08
J15.7 Pneumonia due to Mycoplasma pne..	144	6.71
A02 Other salmonella infections	130	6.06
A56.1 Chlamydial infection of pelvipe..	128	5.96
A15.0 Tuberculosis of lung, confirmed..	106	4.94
J20.0 Acute bronchitis due to Mycopla..	79	3.68
A74.9 Chlamydial infection, unspecified	73	3.40
A56.0 Chlamydial infection of lower g..	59	2.75
A18.2 Tuberculous peripheral lymphade..	52	2.42
A04.6 Enteritis due to Yersinia enter..	50	2.33
A02.1 Salmonella sepsis	41	1.91
A16.5 Tuberculous pleurisy, without m..	35	1.63
A21.9 Tularaemia, unspecified	32	1.49
A21.0 Ulceroglandular tularaemia	29	1.35
A15.1 Tuberculosis of lung, confirmed..	26	1.21
A18.0 Tuberculosis of bones and joints	23	1.07

A31.0 Pulmonary mycobacterial infection	23	1.07
N74.4 Female chlamydial pelvic inflam..	22	1.03
A02.9 Salmonella infection, unspecified	18	0.84
A01.4 Paratyphoid fever, unspecified	16	0.75
A03.3 Shigellosis due to Shigella son..	16	0.75
J16.0 Chlamydial pneumonia	15	0.70
A02.8 Other specified salmonella infe..	13	0.61
A18.1 Tuberculosis of genitourinary s..	13	0.61
A02.2 Localized salmonella infections	12	0.56
A03.1 Shigellosis due to Shigella fle..	12	0.56
A15.6 Tuberculous pleurisy, confirmed..	12	0.56
A01.2 Paratyphoid fever B	11	0.51
A16.0 Tuberculosis of lung, bacteriol..	11	0.51
A32.7 Listerial sepsis	11	0.51
A74.8 Other chlamydial diseases	10	0.47
A01.0 Typhoid fever	9	0.42
A16.9 Respiratory tuberculosis unspec..	9	0.42
A03.9 Shigellosis, unspecified	8	0.37
A15.2 Tuberculosis of lung, confirmed..	8	0.37
A18.3 Tuberculosis of intestines, per..	8	0.37
A32.8 Other forms of listeriosis	8	0.37
A49.3 Mycoplasma infection, unspecifi..	8	0.37
A18.8 Tuberculosis of other specified..	7	0.33
A31.9 Mycobacterial infection, unspec..	7	0.33
A56.2 Chlamydial infection of genitou..	7	0.33
A21.2 Pulmonary tularaemia	6	0.28
A31 Infection due to other mycobacteria	6	0.28
A15.4 Tuberculosis of intrathoracic l..	5	0.23
A15.7 Primary respiratory tuberculosi..	5	0.23
A15.9 Respiratory tuberculosis unspec..	5	0.23
A16.1 Tuberculosis of lung, bacteriol..	5	0.23
A18.4 Tuberculosis of skin and subcut..	5	0.23
A26.0 Cutaneous erysipeloid	5	0.23
A30 Leprosy [Hansen disease]	5	0.23
A32.1 Listerial meningitis and mening..	5	0.23
O98.0 Tuberculosis complicating pregn..	5	0.23
A01.3 Paratyphoid fever C	<5	<0.23
A15.8 Other respiratory tuberculosis,..	<5	<0.23
A16.7 Primary respiratory tuberculosi..	<5	<0.23
A21.7 Generalized tularaemia	<5	<0.23
A78 Q fever	<5	<0.23
M49.0 Tuberculosis of spine	<5	<0.23
A01.1 Paratyphoid fever A	<5	<0.23
A03.0 Shigellosis due to Shigella dys..	<5	<0.23
A15.3 Tuberculosis of lung, confirmed..	<5	<0.23
A16.3 Tuberculosis of intrathoracic l..	<5	<0.23
A17.0 Tuberculous meningitis	<5	<0.23
A17.8 Other tuberculosis of nervous s..	<5	<0.23

A21.8 Other forms of tularaemia	<5	<0.23
A28.2 Extraintestinal yersiniosis	<5	<0.23
A31.1 Cutaneous mycobacterial infection	<5	<0.23
A48.1 Legionnaires disease	<5	<0.23
A75.0 Epidemic louse-borne typhus fev..	<5	<0.23
A75.9 Typhus fever, unspecified	<5	<0.23
B96.0 Mycoplasma pneumoniae [M. pneum..	<5	<0.23
A03 Shigellosis	<5	<0.23
A03.2 Shigellosis due to Shigella boy..	<5	<0.23
A15.5 Tuberculosis of larynx, trachea..	<5	<0.23
A16.8 Other respiratory tuberculosis,..	<5	<0.23
A19.2 Acute miliary tuberculosis, uns..	<5	<0.23
A19.9 Miliary tuberculosis, unspecified	<5	<0.23
A23.9 Brucellosis, unspecified	<5	<0.23
A24.0 Glanders	<5	<0.23
A31.8 Other mycobacterial infections	<5	<0.23
A43.0 Pulmonary nocardiosis	<5	<0.23
A44.9 Bartonellosis, unspecified	<5	<0.23
A70 Chlamydia psittaci infection	<5	<0.23
A77.1 Spotted fever due to Rickettsia..	<5	<0.23
K67.3 Tuberculous peritonitis	<5	<0.23
M49.1 Brucella spondylitis	<5	<0.23
A03.8 Other shigellosis	<5	<0.23
A04.2 Enteroinvasive Escherichia coli..	<5	<0.23
A16.4 Tuberculosis of larynx, trachea..	<5	<0.23
A17.9 Tuberculosis of nervous system,..	<5	<0.23
A18.5 Tuberculosis of eye	<5	<0.23
A19.0 Acute miliary tuberculosis of a..	<5	<0.23
A20.3 Plague meningitis	<5	<0.23
A20.9 Plague, unspecified	<5	<0.23
A21.1 Oculoglandular tularaemia	<5	<0.23
A24.1 Acute and fulminating melioidosis	<5	<0.23
A26.9 Erysipeloid, unspecified	<5	<0.23
A32.9 Listeriosis, unspecified	<5	<0.23
A44.1 Cutaneous and mucocutaneous bar..	<5	<0.23
A44.8 Other forms of bartonellosis	<5	<0.23
A55 Chlamydial lymphogranuloma (vener..	<5	<0.23
A56.3 Chlamydial infection of anus an..	<5	<0.23
A56.8 Sexually transmitted chlamydial..	<5	<0.23
A58 Granuloma inguinale	<5	<0.23
A71 Trachoma	<5	<0.23
A77.8 Other spotted fevers	<5	<0.23
A79 Other rickettsioses	<5	<0.23
A79.9 Rickettsiosis, unspecified	<5	<0.23
K23.0 Tuberculous oesophagitis	<5	<0.23
M01.1 Tuberculous arthritis	<5	<0.23
**Total**	**2 146**	**100**

eTable 15. Distribution of Gram-positive bacterial infections in primary analysis (Finnish multicohort sample)

ICD-10 code and description	N	%
J03.0 Streptococcal tonsillitis	1336	33.73
A04.7 Enterocolitis due to Clostridiu..	670	16.91
J13 Pneumonia due to Streptococcus pn..	412	10.40
A41.0 Sepsis due to Staphylococcus au..	380	9.59
A40.3 Sepsis due to Streptococcus pne..	200	5.05
A40.8 Other streptococcal sepsis	123	3.11
A40.0 Sepsis due to streptococcus, gr..	83	2.10
A41.1 Sepsis due to other specified s..	79	1.99
A40.9 Streptococcal sepsis, unspecified	73	1.84
J02.0 Streptococcal pharyngitis	63	1.59
A49.0 Staphylococcal infection, unspe..	58	1.46
A38 Scarlet fever	56	1.41
M00.0 Staphylococcal arthritis and po..	42	1.06
A40.1 Sepsis due to streptococcus, gr..	39	0.98
A41.2 Sepsis due to unspecified staph..	38	0.96
L01.0 Impetigo [any organism] [any si..	30	0.76
G00.1 Pneumococcal meningitis	26	0.66
B95.6 Staphylococcus aureus as the ca..	18	0.45
A49.1 Streptococcal infection, unspec..	17	0.43
A48.0 Gas gangrene	14	0.35
B95.8 Unspecified staphylococcus as t..	14	0.35
L01 Impetigo	13	0.33
M00.2 Other streptococcal arthritis a..	13	0.33
L01.1 Impetiginization of other derma..	12	0.30
A32.7 Listerial sepsis	10	0.25
B95.5 Unspecified streptococcus as th..	10	0.25
A32.8 Other forms of listeriosis	9	0.23
A42.9 Actinomycosis, unspecified	8	0.20
B95.4 Other streptococcus as the caus..	8	0.20
J15.2 Pneumonia due to staphylococcus	8	0.20
J15.4 Pneumonia due to other streptoc..	8	0.20
A05.0 Foodborne staphylococcal intoxi..	6	0.15
B95.3 Streptococcus pneumoniae as the..	6	0.15
G00.3 Staphylococcal meningitis	6	0.15
J15.3 Pneumonia due to streptococcus,..	6	0.15
A26.0 Cutaneous erysipeloid	5	0.13
A32.1 Listerial meningitis and mening..	5	0.13
B95.0 Streptococcus, group A, as the ..	5	0.13
B95.7 Other staphylococcus as the cau..	5	0.13
G00.2 Streptococcal meningitis	5	0.13
A05.1 Botulism	<5	<0.13
A05.2 Foodborne Clostridium perfringe..	<5	<0.13
A22.0 Cutaneous anthrax	<5	<0.13
A22.2 Gastrointestinal anthrax	<5	<0.13

A26.9 Erysipeloid, unspecified	<5	<0.13
A32.9 Listeriosis, unspecified	<5	<0.13
A35 Other tetanus	<5	<0.13
A36 Diphtheria	<5	<0.13
A40 Streptococcal sepsis	<5	<0.13
A40.2 Sepsis due to streptococcus, gr..	<5	<0.13
A42 Actinomycosis	<5	<0.13
A42.0 Pulmonary actinomycosis	<5	<0.13
A42.1 Abdominal actinomycosis	<5	<0.13
A42.2 Cervicofacial actinomycosis	<5	<0.13
A42.7 Actinomycotic sepsis	<5	<0.13
A42.8 Other forms of actinomycosis	<5	<0.13
A43.0 Pulmonary nocardiosis	<5	<0.13
A48.3 Toxic shock syndrome	<5	<0.13
B95.1 Streptococcus, group B, as the ..	<5	<0.13
B96.7 Clostridium perfringens [C. per..	<5	<0.13
J20.2 Acute bronchitis due to strepto..	<5	<0.13
L00 Staphylococcal scalded skin syndr..	<5	<0.13
M00.1 Pneumococcal arthritis and poly..	<5	<0.13
**Total**	**3 961**	**100**

eTable 16. Distribution of Gram-negative bacterial infections in primary analysis (Finnish multicohort sample)

ICD-10 code and description	N	%
A41.5 Sepsis due to other Gram-negati..	907	28.54
A69.2 Lyme disease	345	10.86
A04.5 Campylobacter enteritis	285	8.97
A02.0 Salmonella enteritis	231	7.27
A02 Other salmonella infections	130	4.09
A56.1 Chlamydial infection of pelvipe..	122	3.84
A54.9 Gonococcal infection, unspecified	90	2.83
A54.2 Gonococcal pelviperitonitis and..	85	2.67
A74.9 Chlamydial infection, unspecified	71	2.23
A56.0 Chlamydial infection of lower g..	59	1.86
A04.6 Enteritis due to Yersinia enter..	51	1.60
A39.0 Meningococcal meningitis	47	1.48
A37 Whooping cough	42	1.32
A02.1 Salmonella sepsis	40	1.26
B96.2 Escherichia coli [E. coli] as t..	35	1.10
G00.0 Haemophilus meningitis	33	1.04
A21.9 Tularaemia, unspecified	32	1.01
A21.0 Ulceroglandular tularaemia	29	0.91
A54.1 Gonococcal infection of lower g..	22	0.69
N74.4 Female chlamydial pelvic inflam..	22	0.69
A52.1 Symptomatic neurosyphilis	19	0.60
J14 Pneumonia due to Haemophilus infl..	19	0.60
A02.9 Salmonella infection, unspecified	18	0.57

A51.0 Primary genital syphilis	17	0.53
A01.4 Paratyphoid fever, unspecified	16	0.50
A03.3 Shigellosis due to Shigella son..	16	0.50
A51.3 Secondary syphilis of skin and ..	16	0.50
J16.0 Chlamydial pneumonia	15	0.47
A39.2 Acute meningococcaemia	13	0.41
A02.2 Localized salmonella infections	12	0.38
A02.8 Other specified salmonella infe..	12	0.38
A03.1 Shigellosis due to Shigella fle..	12	0.38
A04.3 Enterohaemorrhagic Escherichia ..	12	0.38
J15.0 Pneumonia due to Klebsiella pne..	12	0.38
J20.1 Acute bronchitis due to Haemoph..	11	0.35
M01.2 Arthritis in Lyme disease	11	0.35
A01.2 Paratyphoid fever B	10	0.31
A54.0 Gonococcal infection of lower g..	10	0.31
A74.8 Other chlamydial diseases	10	0.31
A01.0 Typhoid fever	9	0.28
A39.4 Meningococcaemia, unspecified	9	0.28
J15.1 Pneumonia due to Pseudomonas	9	0.28
A03.9 Shigellosis, unspecified	8	0.25
B96.5 Pseudomonas (aeruginosa) as the..	8	0.25
A56.2 Chlamydial infection of genitou..	7	0.22
A04.0 Enteropathogenic Escherichia co..	6	0.19
A21.2 Pulmonary tularaemia	6	0.19
J15.5 Pneumonia due to Escherichia coli	6	0.19
J15.6 Pneumonia due to other aerobic ..	6	0.19
A04.4 Other intestinal Escherichia co..	5	0.16
A37.9 Whooping cough, unspecified	5	0.16
A51.5 Early syphilis, latent	5	0.16
A53.9 Syphilis, unspecified	5	0.16
A01.3 Paratyphoid fever C	<5	<0.16
A21.7 Generalized tularaemia	<5	<0.16
A41.3 Sepsis due to Haemophilus influ..	<5	<0.16
A51.9 Early syphilis, unspecified	<5	<0.16
A69.9 Spirochaetal infection, unspeci..	<5	<0.16
A78 Q fever	<5	<0.16
A00.9 Cholera, unspecified	<5	<0.16
A01.1 Paratyphoid fever A	<5	<0.16
A03.0 Shigellosis due to Shigella dys..	<5	<0.16
A21.8 Other forms of tularaemia	<5	<0.16
A28.2 Extraintestinal yersiniosis	<5	<0.16
A37.0 Whooping cough due to Bordetell..	<5	<0.16
A39.8 Other meningococcal infections	<5	<0.16
A48.1 Legionnaires disease	<5	<0.16
A51.4 Other secondary syphilis	<5	<0.16
A53 Other and unspecified syphilis	<5	<0.16
A54.4 Gonococcal infection of musculo..	<5	<0.16
A54.8 Other gonococcal infections	<5	<0.16

A68 Relapsing fevers	<5	<0.16
A75.0 Epidemic louse-borne typhus fev..	<5	<0.16
A75.9 Typhus fever, unspecified	<5	<0.16
B96.1 Klebsiella pneumoniae [K. pneum..	<5	<0.16
A03 Shigellosis	<5	<0.16
A03.2 Shigellosis due to Shigella boy..	<5	<0.16
A24.0 Glanders	<5	<0.16
A27 Leptospirosis	<5	<0.16
A44.9 Bartonellosis, unspecified	<5	<0.16
A50.5 Other late congenital syphilis,..	<5	<0.16
A50.9 Congenital syphilis, unspecified	<5	<0.16
A51.2 Primary syphilis of other sites	<5	<0.16
A67 Pinta [carate]	<5	<0.16
A67.1 Intermediate lesions of pinta	<5	<0.16
A68.9 Relapsing fever, unspecified	<5	<0.16
A77.1 Spotted fever due to Rickettsia..	<5	<0.16
B96.3 Haemophilus influenzae [H. infl..	<5	<0.16
M49.1 Brucella spondylitis	<5	<0.16
N74.3 Female gonococcal pelvic inflam..	<5	<0.16
A03.8 Other shigellosis	<5	<0.16
A04.1 Enterotoxigenic Escherichia col..	<5	<0.16
A04.2 Enteroinvasive Escherichia coli..	<5	<0.16
A20.3 Plague meningitis	<5	<0.16
A20.9 Plague, unspecified	<5	<0.16
A21.1 Oculoglandular tularaemia	<5	<0.16
A23.9 Brucellosis, unspecified	<5	<0.16
A24.1 Acute and fulminating melioidosis	<5	<0.16
A25.9 Rat-bite fever, unspecified	<5	<0.16
A27.0 Leptospirosis icterohaemorrhagica	<5	<0.16
A27.8 Other forms of leptospirosis	<5	<0.16
A27.9 Leptospirosis, unspecified	<5	<0.16
A28.0 Pasteurellosis	<5	<0.16
A39 Meningococcal infection	<5	<0.16
A39.1 Waterhouse-Friderichsen syndrome	<5	<0.16
A39.5 Meningococcal heart disease	<5	<0.16
A39.9 Meningococcal infection, unspec..	<5	<0.16
A44.1 Cutaneous and mucocutaneous bar..	<5	<0.16
A44.8 Other forms of bartonellosis	<5	<0.16
A49.2 Haemophilus influenzae infectio..	<5	<0.16
A50 Congenital syphilis	<5	<0.16
A51.1 Primary anal syphilis	<5	<0.16
A52.0 Cardiovascular syphilis	<5	<0.16
A52.7 Other symptomatic late syphilis	<5	<0.16
A52.8 Late syphilis, latent	<5	<0.16
A54 Gonococcal infection	<5	<0.16
A54.3 Gonococcal infection of eye	<5	<0.16
A54.6 Gonococcal infection of anus an..	<5	<0.16
A55 Chlamydial lymphogranuloma (vener..	<5	<0.16

A56.3 Chlamydial infection of anus an..	<5	<0.16
A56.8 Sexually transmitted chlamydial..	<5	<0.16
A58 Granuloma inguinale	<5	<0.16
A66.1 Multiple papillomata and wet cr..	<5	<0.16
A66.3 Hyperkeratosis of yaws	<5	<0.16
A70 Chlamydia psittaci infection	<5	<0.16
A71 Trachoma	<5	<0.16
A77.8 Other spotted fevers	<5	<0.16
A79 Other rickettsioses	<5	<0.16
A79.9 Rickettsiosis, unspecified	<5	<0.16
M01.0 Meningococcal arthritis	<5	<0.16
M49.2 Enterobacterial spondylitis	<5	<0.16
M73.0 Gonococcal bursitis	<5	<0.16
O98.1 Syphilis complicating pregnancy..	<5	<0.16
**Total**	**3 178**	**100**

eTable 17. Distribution of viral infections in primary analysis (Finnish multicohort sample)

ICD-10 code and description	N	%
A08.4 Viral intestinal infection, uns..	1131	9.98
A63.0 Anogenital (venereal) warts	1050	9.26
B34.9 Viral infection, unspecified	775	6.84
A98.5 Haemorrhagic fever with renal s..	757	6.68
B27.0 Gammaherpesviral mononucleosis	529	4.67
B27.9 Infectious mononucleosis, unspe..	413	3.64
J11.1 Influenza with other respirator..	389	3.43
B01.9 Varicella without complication	338	2.98
B06.9 Rubella without complication	335	2.96
J10.1 Influenza with other respirator..	316	2.79
J11.8 Influenza with other manifestat..	312	2.75
B18.2 Chronic viral hepatitis C	294	2.59
A60.0 Herpesviral infection of genita..	277	2.44
A87.9 Viral meningitis, unspecified	257	2.27
B02.9 Zoster without complication	184	1.62
B19.9 Unspecified viral hepatitis wit..	178	1.57
J12 Viral pneumonia, not elsewhere cl..	177	1.56
B07 Viral warts	165	1.46
A89 Unspecified viral infection of ce..	163	1.44
J09 Influenza due to certain identifi..	154	1.36
A86 Unspecified viral encephalitis	145	1.28
A87.0 Enteroviral meningitis	145	1.28
J10.0 Influenza with pneumonia, other..	132	1.16
B97.8 Other viral agents as the cause..	121	1.07
B00.1 Herpesviral vesicular dermatitis	112	0.99
A95 Yellow fever	109	0.96
B05.9 Measles without complication	99	0.87
A08.3 Other viral enteritis	96	0.85
B34.8 Other viral infections of unspe..	86	0.76

B08.2 Exanthema subitum [sixth disease]	85	0.75
B00.2 Herpesviral gingivostomatitis a..	83	0.73
B26.2 Mumps encephalitis	70	0.62
B09 Unspecified viral infection chara..	68	0.60
B00.0 Eczema herpeticum	62	0.55
J11.0 Influenza with pneumonia, virus..	62	0.55
B02.8 Zoster with other complications	59	0.52
O98.5 Other viral diseases complicati..	58	0.51
B97.7 Papillomavirus as the cause of ..	56	0.49
A08.0 Rotaviral enteritis	53	0.47
J12.9 Viral pneumonia, unspecified	52	0.46
B15.9 Hepatitis A without hepatic coma	45	0.40
B33.8 Other specified viral diseases	45	0.40
B08.1 Molluscum contagiosum	44	0.39
B25.8 Other cytomegaloviral diseases	42	0.37
J21.9 Acute bronchiolitis, unspecified	42	0.37
B01.8 Varicella with other complicati..	41	0.36
B16.9 Acute hepatitis B without delta..	41	0.36
J10.8 Influenza with other manifestat..	41	0.36
B01.1 Varicella encephalitis	40	0.35
G05.1 Encephalitis, myelitis and ence..	38	0.34
B00.5 Herpesviral ocular disease	37	0.33
B18.1 Chronic viral hepatitis B witho..	36	0.32
B25.9 Cytomegaloviral disease, unspec..	35	0.31
B02.3 Zoster ocular disease	34	0.30
B23.1 HIV disease resulting in (persi..	29	0.26
G02.0 Meningitis in viral diseases cl..	28	0.25
B34.0 Adenovirus infection, unspecifi..	26	0.23
B17.1 Acute hepatitis C	25	0.22
B26.9 Mumps without complication	24	0.21
B01 Varicella [chickenpox]	23	0.20
B24 Unspecified human immunodeficienc..	23	0.20
O98.4 Viral hepatitis complicating pr..	23	0.20
B00.8 Other forms of herpesviral infe..	22	0.19
A80 Acute poliomyelitis	21	0.19
A88.8 Other specified viral infection..	21	0.19
B27.1 Cytomegaloviral mononucleosis	20	0.18
B97.0 Adenovirus as the cause of dise..	19	0.17
J20.5 Acute bronchitis due to respira..	17	0.15
B30 Viral conjunctivitis	16	0.14
B00.9 Herpesviral infection, unspecif..	15	0.13
A87.8 Other viral meningitis	14	0.12
B06 Rubella [German measles]	14	0.12
B25.1 Cytomegaloviral hepatitis	14	0.12
B33.2 Viral carditis	14	0.12
B34.1 Enterovirus infection, unspecif..	13	0.11
A08.1 Acute gastroenteropathy due to ..	12	0.11
A90 Dengue fever [classical dengue]	12	0.11

B00.4 Herpesviral encephalitis	12	0.11
B00.7 Disseminated herpesviral disease	12	0.11
B26.0 Mumps orchitis	12	0.11
B02.0 Zoster encephalitis	11	0.10
J21.0 Acute bronchiolitis due to resp..	11	0.10
A08.2 Adenoviral enteritis	10	0.09
B08.8 Other specified viral infection..	10	0.09
A84.8 Other tick-borne viral encephal..	9	0.08
B05.8 Measles with other complications	9	0.08
Z21 Asymptomatic human immunodeficien..	9	0.08
A60.9 Anogenital herpesviral infectio..	8	0.07
B01.2 Varicella pneumonia	8	0.07
B26.3 Mumps pancreatitis	8	0.07
H62.1 Otitis externa in viral disease..	8	0.07
J12.1 Respiratory syncytial virus pne..	8	0.07
J12.8 Other viral pneumonia	8	0.07
J17.1 Pneumonia in viral diseases cla..	8	0.07
B02.1 Zoster meningitis	7	0.06
B02.2 Zoster with other nervous syste..	7	0.06
B18.9 Chronic viral hepatitis, unspec..	7	0.06
B19.0 Unspecified viral hepatitis wit..	7	0.06
H19.1 Herpesviral keratitis and kerat..	7	0.06
J20.4 Acute bronchitis due to parainf..	7	0.06
A84.9 Tick-borne viral encephalitis, ..	6	0.05
A92.8 Other specified mosquito-borne ..	6	0.05
B02.7 Disseminated zoster	6	0.05
B08.3 Erythema infectiosum [fifth dis..	6	0.05
B08.5 Enteroviral vesicular pharyngitis	6	0.05
B15.0 Hepatitis A with hepatic coma	6	0.05
B97.1 Enterovirus as the cause of dis..	6	0.05
I41.1 Myocarditis in viral diseases c..	6	0.05
A87.2 Lymphocytic choriomeningitis	5	0.04
B17.8 Other specified acute viral hep..	5	0.04
B26.8 Mumps with other complications	5	0.04
B97.4 Respiratory syncytial virus as ..	5	0.04
A60.1 Herpesviral infection of perian..	<5	<0.04
A80.1 Acute paralytic poliomyelitis, ..	<5	<0.04
A80.3 Acute paralytic poliomyelitis, ..	<5	<0.04
A81.2 Progressive multifocal leukoenc..	<5	<0.04
A83.0 Japanese encephalitis	<5	<0.04
A83.2 Eastern equine encephalitis	<5	<0.04
A83.6 Rocio virus disease	<5	<0.04
A83.8 Other mosquito-borne viral ence..	<5	<0.04
A84 Tick-borne viral encephalitis	<5	<0.04
A84.1 Central European tick-borne enc..	<5	<0.04
A85.0 Enteroviral encephalitis	<5	<0.04
A85.1 Adenoviral encephalitis	<5	<0.04
A85.2 Arthropod-borne viral encephali..	<5	<0.04

A85.8 Other specified viral encephali..	<5	<0.04
A87.1 Adenoviral meningitis	<5	<0.04
A88.1 Epidemic vertigo	<5	<0.04
A96.0 Junin haemorrhagic fever	<5	<0.04
A98.0 Crimean-Congo haemorrhagic fever	<5	<0.04
A98.1 Omsk haemorrhagic fever	<5	<0.04
A98.3 Marburg virus disease	<5	<0.04
A98.4 Ebola virus disease	<5	<0.04
A99 Unspecified viral haemorrhagic fe..	<5	<0.04
B00.3 Herpesviral meningitis	<5	<0.04
B01.0 Varicella meningitis	<5	<0.04
B02 Zoster [herpes zoster]	<5	<0.04
B05 Measles	<5	<0.04
B05.3 Measles complicated by otitis m..	<5	<0.04
B06.8 Rubella with other complications	<5	<0.04
B08.0 Other orthopoxvirus infections	<5	<0.04
B08.4 Enteroviral vesicular stomatiti..	<5	<0.04
B16.0 Acute hepatitis B with delta-ag..	<5	<0.04
B16.1 Acute hepatitis B with delta-ag..	<5	<0.04
B17.0 Acute delta-(super)infection of..	<5	<0.04
B17.2 Acute hepatitis E	<5	<0.04
B18.0 Chronic viral hepatitis B with ..	<5	<0.04
B18.8 Other chronic viral hepatitis	<5	<0.04
B20.0 HIV disease resulting in mycoba..	<5	<0.04
B20.6 HIV disease resulting in Pneumo..	<5	<0.04
B21.2 HIV disease resulting in other ..	<5	<0.04
B21.9 HIV disease resulting in unspec..	<5	<0.04
B23.8 HIV disease resulting in other ..	<5	<0.04
B25.0 Cytomegaloviral pneumonitis	<5	<0.04
B26 Mumps	<5	<0.04
B26.1 Mumps meningitis	<5	<0.04
B30.0 Keratoconjunctivitis due to ade..	<5	<0.04
B30.8 Other viral conjunctivitis	<5	<0.04
B33.0 Epidemic myalgia	<5	<0.04
B34.2 Coronavirus infection, unspecif..	<5	<0.04
B34.3 Parvovirus infection, unspecifi..	<5	<0.04
B97.2 Coronavirus as the cause of dis..	<5	<0.04
G04.1 Tropical spastic paraplegia	<5	<0.04
J10 Influenza due to other identified..	<5	<0.04
J11 Influenza, virus not identified	<5	<0.04
J12.0 Adenoviral pneumonia	<5	<0.04
J12.2 Parainfluenza virus pneumonia	<5	<0.04
J20.6 Acute bronchitis due to rhinovi..	<5	<0.04
J21.8 Acute bronchiolitis due to othe..	<5	<0.04
**Total**	**11 335**	**100**

eTable 18. Distribution of herpesvirus infections in primary analysis (Finnish multicohort sample)

ICD-10 code and description	N	%
B27.0 Gammaherpesviral mononucleosis	550	19.88
B27.9 Infectious mononucleosis, unspe..	433	15.65
B01.9 Varicella without complication	353	12.76
A60.0 Herpesviral infection of genita..	295	10.66
B02.9 Zoster without complication	199	7.19
B00.1 Herpesviral vesicular dermatitis	123	4.45
B00.2 Herpesviral gingivostomatitis a..	91	3.29
B08.2 Exanthema subitum [sixth disease]	86	3.11
B02.8 Zoster with other complications	63	2.28
B00.0 Eczema herpeticum	62	2.24
B25.8 Other cytomegaloviral diseases	50	1.81
B01.1 Varicella encephalitis	48	1.73
B01.8 Varicella with other complicati..	41	1.48
B00.4 Herpesviral encephalitis	40	1.45
B00.5 Herpesviral ocular disease	40	1.45
B25.9 Cytomegaloviral disease, unspec..	40	1.45
B02.3 Zoster ocular disease	35	1.26
B00.8 Other forms of herpesviral infe..	24	0.87
B01 Varicella [chickenpox]	24	0.87
B27.1 Cytomegaloviral mononucleosis	21	0.76
B00.3 Herpesviral meningitis	20	0.72
B00.9 Herpesviral infection, unspecif..	15	0.54
B01.2 Varicella pneumonia	14	0.51
B25.1 Cytomegaloviral hepatitis	14	0.51
B00.7 Disseminated herpesviral disease	13	0.47
B02.0 Zoster encephalitis	11	0.4
B02.1 Zoster meningitis	10	0.36
A60.9 Anogenital herpesviral infectio..	9	0.33
B02.2 Zoster with other nervous syste..	8	0.29
B25.0 Cytomegaloviral pneumonitis	8	0.29
B01.0 Varicella meningitis	7	0.25
H19.1 Herpesviral keratitis and kerat..	7	0.25
B02.7 Disseminated zoster	6	0.22
A60.1 Herpesviral infection of perian..	<5	<0.18
B02 Zoster [herpes zoster]	<5	<0.18
B21.0 HIV disease resulting in Kaposi..	<5	<0.18
**Total**	**2 767**	**100**

eTable 19. Distribution of other potentially persistent viral infections in primary analysis (Finnish multicohort sample)

ICD-10 code and description	N	%
A63.0 Anogenital (venereal) warts	1 085	65.24
B18.2 Chronic viral hepatitis C	352	21.17
B97.7 Papillomavirus as the cause of ..	57	3.43

B18.1 Chronic viral hepatitis B witho..	40	2.41
B23.1 HIV disease resulting in (persi..	30	1.8
B24 Unspecified human immunodeficienc..	26	1.56
O98.4 Viral hepatitis complicating pr..	24	1.44
Z21 Asymptomatic human immunodeficien..	10	0.6
B18.9 Chronic viral hepatitis, unspec..	9	0.54
A81.2 Progressive multifocal leukoenc..	5	0.3
B23.8 HIV disease resulting in other ..	<5	<0.3
B18.0 Chronic viral hepatitis B with ..	<5	<0.3
B18.8 Other chronic viral hepatitis	<5	<0.3
B20.0 HIV disease resulting in mycoba..	<5	<0.3
B20.6 HIV disease resulting in Pneumo..	<5	<0.3
B21.2 HIV disease resulting in other ..	<5	<0.3
B20.9 HIV disease resulting in unspec..	<5	<0.3
B21.0 HIV disease resulting in Kaposi..	<5	<0.3
B21.9 HIV disease resulting in unspec..	<5	<0.3
B22.0 HIV disease resulting in enceph..	<5	<0.3
B23.0 Acute HIV infection syndrome	<5	<0.3
B34.4 Papovavirus infection, unspecif..	<5	<0.3
G04.1 Tropical spastic paraplegia	<5	<0.3
**Total**	**1 663**	**100**

eTable 20. Distribution of acute viral infections in primary analysis (Finnish multicohort sample)

ICD-10 code and description	N	%
A08.4 Viral intestinal infection, uns..	1 156	15.77
B34.9 Viral infection, unspecified	785	10.71
A98.5 Haemorrhagic fever with renal s..	769	10.49
J11.1 Influenza with other respirator..	395	5.39
B06.9 Rubella without complication	341	4.65
J10.1 Influenza with other respirator..	325	4.43
J11.8 Influenza with other manifestat..	315	4.3
A87.9 Viral meningitis, unspecified	268	3.66
B19.9 Unspecified viral hepatitis wit..	182	2.48
J12 Viral pneumonia, not elsewhere cl..	180	2.46
B07 Viral warts	168	2.29
A89 Unspecified viral infection of ce..	166	2.26
J09 Influenza due to certain identifi..	157	2.14
A86 Unspecified viral encephalitis	150	2.05
A87.0 Enteroviral meningitis	147	2.01
J10.0 Influenza with pneumonia, other..	137	1.87
B97.8 Other viral agents as the cause..	126	1.72
A95 Yellow fever	111	1.51
B05.9 Measles without complication	102	1.39
A08.3 Other viral enteritis	99	1.35
B34.8 Other viral infections of unspe..	87	1.19
B26.2 Mumps encephalitis	70	0.96
B09 Unspecified viral infection chara..	68	0.93

O98.5 Other viral diseases complicati..	66	0.9
J11.0 Influenza with pneumonia, virus..	65	0.89
A08.0 Rotaviral enteritis	55	0.75
J12.9 Viral pneumonia, unspecified	53	0.72
B15.9 Hepatitis A without hepatic coma	46	0.63
B33.8 Other specified viral diseases	45	0.61
B08.1 Molluscum contagiosum	44	0.6
J21.9 Acute bronchiolitis, unspecified	44	0.6
J10.8 Influenza with other manifestat..	43	0.59
B16.9 Acute hepatitis B without delta..	42	0.57
B17.1 Acute hepatitis C	32	0.44
B34.0 Adenovirus infection, unspecifi..	27	0.37
B26.9 Mumps without complication	24	0.33
A88.8 Other specified viral infection..	22	0.3
A80 Acute poliomyelitis	21	0.29
B97.0 Adenovirus as the cause of dise..	20	0.27
J20.5 Acute bronchitis due to respira..	19	0.26
A87.8 Other viral meningitis	16	0.22
B30 Viral conjunctivitis	16	0.22
B06 Rubella [German measles]	14	0.19
B33.2 Viral carditis	14	0.19
B34.1 Enterovirus infection, unspecif..	13	0.18
J21.0 Acute bronchiolitis due to resp..	13	0.18
A08.1 Acute gastroenteropathy due to ..	12	0.16
A90 Dengue fever [classical dengue]	12	0.16
B26.0 Mumps orchitis	12	0.16
A08.2 Adenoviral enteritis	10	0.14
A84.8 Other tick-borne viral encephal..	10	0.14
B08.8 Other specified viral infection..	10	0.14
J17.1 Pneumonia in viral diseases cla..	10	0.14
B05.8 Measles with other complications	9	0.12
J12.1 Respiratory syncytial virus pne..	9	0.12
B19.0 Unspecified viral hepatitis wit..	8	0.11
B26.3 Mumps pancreatitis	8	0.11
J12.8 Other viral pneumonia	8	0.11
A92.8 Other specified mosquito-borne ..	7	0.1
B15.0 Hepatitis A with hepatic coma	7	0.1
B17.8 Other specified acute viral hep..	7	0.1
B97.1 Enterovirus as the cause of dis..	7	0.1
J20.4 Acute bronchitis due to parainf..	7	0.1
A84.9 Tick-borne viral encephalitis, ..	6	0.08
B08.3 Erythema infectiosum [fifth dis..	6	0.08
B08.5 Enteroviral vesicular pharyngitis	6	0.08
B97.4 Respiratory syncytial virus as ..	6	0.08
I41.1 Myocarditis in viral diseases c..	6	0.08
A87.2 Lymphocytic choriomeningitis	5	0.07
B26.8 Mumps with other complications	5	0.07
A80.1 Acute paralytic poliomyelitis, ..	<5	<0.07

A80.3 Acute paralytic poliomyelitis, ..	<5	<0.07
A83.0 Japanese encephalitis	<5	<0.07
A83.2 Eastern equine encephalitis	<5	<0.07
A83.6 Rocio virus disease	<5	<0.07
A83.8 Other mosquito-borne viral ence..	<5	<0.07
A84 Tick-borne viral encephalitis	<5	<0.07
A84.1 Central European tick-borne enc..	<5	<0.07
A85.0 Enteroviral encephalitis	<5	<0.07
A85.1 Adenoviral encephalitis	<5	<0.07
A85.2 Arthropod-borne viral encephali..	<5	<0.07
A85.8 Other specified viral encephali..	<5	<0.07
A87.1 Adenoviral meningitis	<5	<0.07
A88.1 Epidemic vertigo	<5	<0.07
A96.0 Junin haemorrhagic fever	<5	<0.07
A98.0 Crimean-Congo haemorrhagic fever	<5	<0.07
A98.1 Omsk haemorrhagic fever	<5	<0.07
A98.3 Marburg virus disease	<5	<0.07
A98.4 Ebola virus disease	<5	<0.07
A99 Unspecified viral haemorrhagic fe..	<5	<0.07
B05 Measles	<5	<0.07
B05.3 Measles complicated by otitis m..	<5	<0.07
B06.8 Rubella with other complications	<5	<0.07
B08.0 Other orthopoxvirus infections	<5	<0.07
B08.4 Enteroviral vesicular stomatiti..	<5	<0.07
B16.0 Acute hepatitis B with delta-ag..	<5	<0.07
B16.1 Acute hepatitis B with delta-ag..	<5	<0.07
B17.0 Acute delta-(super)infection of..	<5	<0.07
B17.2 Acute hepatitis E	<5	<0.07
B26 Mumps	<5	<0.07
B26.1 Mumps meningitis	<5	<0.07
B30.0 Keratoconjunctivitis due to ade..	<5	<0.07
B30.8 Other viral conjunctivitis	<5	<0.07
B33.0 Epidemic myalgia	<5	<0.07
B34.2 Coronavirus infection, unspecif..	<5	<0.07
B34.3 Parvovirus infection, unspecifi..	<5	<0.07
B97.2 Coronavirus as the cause of dis..	<5	<0.07
J10 Influenza due to other identified..	<5	<0.07
J11 Influenza, virus not identified	<5	<0.07
J12.0 Adenoviral pneumonia	<5	<0.07
J12.2 Parainfluenza virus pneumonia	<5	<0.07
J20.6 Acute bronchitis due to rhinovi..	<5	<0.07
J21.8 Acute bronchiolitis due to othe..	<5	<0.07
**Total**	**7 329**	**100**

eTable 21. Distribution of cenral nervous system infections in primary analysis (Finnish multicohort sample)

ICD-10 code and description	N	%

A87.9 Viral meningitis, unspecified	280	16.91
A89 Unspecified viral infection of ce..	167	10.08
A86 Unspecified viral encephalitis	152	9.18
G00.9 Bacterial meningitis, unspecified	147	8.88
A87.0 Enteroviral meningitis	145	8.76
G03.9 Meningitis, unspecified	102	6.16
B26.2 Mumps encephalitis	71	4.29
G00.8 Other bacterial meningitis	46	2.78
B01.1 Varicella encephalitis	42	2.54
G05.1 Encephalitis, myelitis and ence..	40	2.42
G00.1 Pneumococcal meningitis	37	2.23
G06.1 Intraspinal abscess and granuloma	35	2.11
A39.0 Meningococcal meningitis	34	2.05
G00.0 Haemophilus meningitis	33	1.99
G06.0 Intracranial abscess and granul..	33	1.99
G01 Meningitis in bacterial diseases ..	29	1.75
G02.0 Meningitis in viral diseases cl..	28	1.69
A88.8 Other specified viral infection..	22	1.33
A80 Acute poliomyelitis	21	1.27
A52.1 Symptomatic neurosyphilis	18	1.09
A87.8 Other viral meningitis	16	0.97
B00.4 Herpesviral encephalitis	12	0.72
B02.0 Zoster encephalitis	11	0.66
A84.8 Other tick-borne viral encephal..	10	0.6
B19.0 Unspecified viral hepatitis wit..	9	0.54
G03.8 Meningitis due to other specifi..	9	0.54
B02.1 Zoster meningitis	8	0.48
B15.0 Hepatitis A with hepatic coma	8	0.48
A84.9 Tick-borne viral encephalitis, ..	6	0.36
G03.0 Nonpyogenic meningitis	6	0.36
G05.0 Encephalitis, myelitis and ence..	6	0.36
A81.2 Progressive multifocal leukoenc..	5	0.3
G00.2 Streptococcal meningitis	5	0.3
G00.3 Staphylococcal meningitis	5	0.3
A17.0 Tuberculous meningitis	<5	<0.3
A20.3 Plague meningitis	<5	<0.3
A32.1 Listerial meningitis and mening..	<5	<0.3
A80.1 Acute paralytic poliomyelitis, ..	<5	<0.3
A80.3 Acute paralytic poliomyelitis, ..	<5	<0.3
A83.0 Japanese encephalitis	<5	<0.3
A83.2 Eastern equine encephalitis	<5	<0.3
A83.6 Rocio virus disease	<5	<0.3
A83.8 Other mosquito-borne viral ence..	<5	<0.3
A84 Tick-borne viral encephalitis	<5	<0.3
A84.1 Central European tick-borne enc..	<5	<0.3
A85.0 Enteroviral encephalitis	<5	<0.3
A85.1 Adenoviral encephalitis	<5	<0.3
A85.2 Arthropod-borne viral encephali..	<5	<0.3

A85.8 Other specified viral encephali..	<5	<0.3
A87.1 Adenoviral meningitis	<5	<0.3
A87.2 Lymphocytic choriomeningitis	<5	<0.3
A88.1 Epidemic vertigo	<5	<0.3
B00.3 Herpesviral meningitis	<5	<0.3
B01.0 Varicella meningitis	<5	<0.3
B16.0 Acute hepatitis B with delta-ag..	<5	<0.3
B22.0 HIV disease resulting in enceph..	<5	<0.3
B26.1 Mumps meningitis	<5	<0.3
B50.0 Plasmodium falciparum malaria w..	<5	<0.3
B69.0 Cysticercosis of central nervou..	<5	<0.3
G03.1 Chronic meningitis	<5	<0.3
G04.1 Tropical spastic paraplegia	<5	<0.3
G04.2 Bacterial meningoencephalitis a..	<5	<0.3
G05.2 Encephalitis, myelitis and ence..	<5	<0.3
G06 Intracranial and intraspinal absc..	<5	<0.3
G06.2 Extradural and subdural abscess..	<5	<0.3
G07 Intracranial and intraspinal absc..	<5	<0.3
I68.1 Cerebral arteritis in infectiou..	<5	<0.3
**Total**	**1 656**	**100**

eTable 22. Distribution of extra-central nervoys system infections in primary analysis (Finnish multicohort sample)

ICD-10 code and description	N	%
K35 Acute appendicitis	11 903	15.60
J18.9 Pneumonia, unspecified	6088	7.98
A09 Other gastroenteritis and colitis..	5639	7.39
N10 Acute tubulo-interstitial nephritis	3614	4.74
J03.9 Acute tonsillitis, unspecified	3098	4.06
A46 Erysipelas	3097	4.06
J36 Peritonsillar abscess	3041	3.99
J15.9 Bacterial pneumonia, unspecified	2243	2.94
N70.0 Acute salpingitis and oophoritis	1959	2.57
O85 Puerperal sepsis	1735	2.27
J20.9 Acute bronchitis, unspecified	1153	1.51
N39.0 Urinary tract infection, site n..	1060	1.39
J01.0 Acute maxillary sinusitis	1047	1.37
A49.9 Bacterial infection, unspecified	1030	1.35
J03.0 Streptococcal tonsillitis	927	1.21
A63.0 Anogenital (venereal) warts	847	1.11
A08.4 Viral intestinal infection, uns..	819	1.07
J22 Unspecified acute lower respirato..	778	1.02
N71.0 Acute inflammatory disease of u..	726	0.95
J02.9 Acute pharyngitis, unspecified	696	0.91
J04.0 Acute laryngitis	678	0.89
B34.9 Viral infection, unspecified	620	0.81
A98.5 Haemorrhagic fever with renal s..	580	0.76

A08.5 Other specified intestinal infe..	490	0.64
K57.2 Diverticular disease of large i..	457	0.60
N30.0 Acute cystitis	445	0.58
J18.0 Bronchopneumonia, unspecified	441	0.58
B27.0 Gammaherpesviral mononucleosis	417	0.55
A41.5 Sepsis due to other Gram-negati..	413	0.54
L05.9 Pilonidal cyst without abscess	392	0.51
O08.0 Genital tract and pelvic infect..	375	0.49
O03.5 Spontaneous abortion: Complete ..	366	0.48
B27.9 Infectious mononucleosis, unspe..	365	0.48
K61.0 Anal abscess	330	0.43
K05.3 Chronic periodontitis	328	0.43
A04.7 Enterocolitis due to Clostridiu..	310	0.41
N75.1 Abscess of Bartholin gland	297	0.39
H66.0 Acute suppurative otitis media	290	0.38
J11.1 Influenza with other respirator..	287	0.38
A41.9 Sepsis, unspecified	281	0.37
B06.9 Rubella without complication	278	0.36
J13 Pneumonia due to Streptococcus pn..	276	0.36
K04.7 Periapical abscess without sinus	264	0.35
B01.9 Varicella without complication	261	0.34
J11.8 Influenza with other manifestat..	257	0.34
N61 Inflammatory disorders of breast	255	0.33
A69.2 Lyme disease	246	0.32
L04 Acute lymphadenitis	227	0.30
M00.9 Pyogenic arthritis, unspecified	214	0.28
N45.9 Orchitis, epididymitis and epid..	210	0.28
J10.1 Influenza with other respirator..	209	0.27
B99 Other and unspecified infectious ..	199	0.26
L03.0 Cellulitis of finger and toe	197	0.26
A04.5 Campylobacter enteritis	194	0.25
K04.5 Chronic apical periodontitis	194	0.25
A60.0 Herpesviral infection of genita..	181	0.24
K65.0 Acute peritonitis	180	0.24
O23.0 Infections of kidney in pregnancy	176	0.23
K61 Abscess of anal and rectal regions	175	0.23
O23.5 Infections of the genital tract..	175	0.23
K12.2 Cellulitis and abscess of mouth	174	0.23
B18.2 Chronic viral hepatitis C	173	0.23
A41.0 Sepsis due to Staphylococcus au..	169	0.22
J05.1 Acute epiglottitis	169	0.22
O03.0 Spontaneous abortion: Incomplet..	169	0.22
L02 Cutaneous abscess, furuncle and c..	166	0.22
K02.1 Caries of dentine	163	0.21
O86.0 Infection of obstetric surgical..	163	0.21
L08.9 Local infection of skin and sub..	158	0.21
O23.9 Other and unspecified genitouri..	158	0.21
A02.0 Salmonella enteritis	153	0.20

J15.8 Other bacterial pneumonia	148	0.19
L02.4 Cutaneous abscess, furuncle and..	146	0.19
B19.9 Unspecified viral hepatitis wit..	143	0.19
J12 Viral pneumonia, not elsewhere cl..	141	0.18
A41.8 Other specified sepsis	140	0.18
A16.2 Tuberculosis of lung, without m..	135	0.18
B07 Viral warts	135	0.18
O86.1 Other infection of genital trac..	131	0.17
B86 Scabies	127	0.17
L05.0 Pilonidal cyst with abscess	123	0.16
O86.4 Pyrexia of unknown origin follo..	120	0.16
A40.3 Sepsis due to Streptococcus pne..	116	0.15
K05.2 Acute periodontitis	115	0.15
J18.1 Lobar pneumonia, unspecified	112	0.15
A02 Other salmonella infections	110	0.14
K02 Dental caries	105	0.14
B02.9 Zoster without complication	102	0.13
B97.8 Other viral agents as the cause..	102	0.13
B35 Dermatophytosis	101	0.13
L04.0 Acute lymphadenitis of face, he..	100	0.13
A56.1 Chlamydial infection of pelvipe..	99	0.13
L03.1 Cellulitis of other parts of limb	97	0.13
J15.7 Pneumonia due to Mycoplasma pne..	92	0.12
O23.4 Unspecified infection of urinar..	92	0.12
L02.2 Cutaneous abscess, furuncle and..	91	0.12
O04.0 Medical abortion: Incomplete, c..	90	0.12
B05.9 Measles without complication	87	0.11
A49.8 Other bacterial infections of u..	86	0.11
O23.1 Infections of bladder in pregna..	84	0.11
A04.9 Bacterial intestinal infection,..	81	0.11
H00.1 Chalazion	81	0.11
J86.9 Pyothorax without fistula	80	0.10
B00.1 Herpesviral vesicular dermatitis	79	0.10
L02.3 Cutaneous abscess, furuncle and..	79	0.10
A15.0 Tuberculosis of lung, confirmed..	78	0.10
B08.2 Exanthema subitum [sixth disease]	78	0.10
H10.0 Mucopurulent conjunctivitis	78	0.10
A95 Yellow fever	76	0.10
B34.8 Other viral infections of unspe..	76	0.10
J16.8 Pneumonia due to other specifie..	76	0.10
M71.1 Other infective bursitis	76	0.10
O86.2 Urinary tract infection followi..	74	0.10
A54.9 Gonococcal infection, unspecified	73	0.10
L03.9 Cellulitis, unspecified	73	0.10
A08.3 Other viral enteritis	72	0.09
I33.0 Acute and subacute infective en..	71	0.09
N41.0 Acute prostatitis	71	0.09
H60.3 Other infective otitis externa	70	0.09

L08.8 Other specified local infection..	70	0.09
M86.9 Osteomyelitis, unspecified	69	0.09
N76.4 Abscess of vulva	69	0.09
O04.5 Medical abortion: Complete or u..	67	0.09
J39.0 Retropharyngeal and parapharyng..	66	0.09
K57.0 Diverticular disease of small i..	65	0.09
M86.1 Other acute osteomyelitis	64	0.08
B00.2 Herpesviral gingivostomatitis a..	63	0.08
J09 Influenza due to certain identifi..	63	0.08
K04.6 Periapical abscess with sinus	63	0.08
B37 Candidiasis	62	0.08
O75.3 Other infection during labour	61	0.08
J10.0 Influenza with pneumonia, other..	60	0.08
O86.8 Other specified puerperal infec..	60	0.08
O98.8 Other maternal infectious and p..	60	0.08
A04.8 Other specified bacterial intes..	58	0.08
N77.1 Vaginitis, vulvitis and vulvova..	57	0.07
H62.0 Otitis externa in bacterial dis..	56	0.07
A54.2 Gonococcal pelviperitonitis and..	55	0.07
A74.9 Chlamydial infection, unspecified	55	0.07
B00.0 Eczema herpeticum	54	0.07
B09 Unspecified viral infection chara..	54	0.07
L02.1 Cutaneous abscess, furuncle and..	54	0.07
L02.9 Cutaneous abscess, furuncle and..	52	0.07
O91.1 Abscess of breast associated wi..	50	0.07
A38 Scarlet fever	48	0.06
N73.3 Female acute pelvic peritonitis	47	0.06
H00.0 Hordeolum and other deep inflam..	46	0.06
A40.8 Other streptococcal sepsis	45	0.06
H05.0 Acute inflammation of orbit	44	0.06
O98.5 Other viral diseases complicati..	44	0.06
A56.0 Chlamydial infection of lower g..	42	0.06
B08.1 Molluscum contagiosum	41	0.05
J11.0 Influenza with pneumonia, virus..	41	0.05
J20.0 Acute bronchitis due to Mycopla..	41	0.05
A18.2 Tuberculous peripheral lymphade..	40	0.05
A40.9 Streptococcal sepsis, unspecified	39	0.05
B15.9 Hepatitis A without hepatic coma	39	0.05
I40.0 Infective myocarditis	39	0.05
J02.0 Streptococcal pharyngitis	39	0.05
A40.0 Sepsis due to streptococcus, gr..	38	0.05
K61.1 Rectal abscess	38	0.05
L02.0 Cutaneous abscess, furuncle and..	38	0.05
L03.2 Cellulitis of face	38	0.05
A08.0 Rotaviral enteritis	37	0.05
J18.8 Other pneumonia, organism unspe..	37	0.05
O98.3 Other infections with a predomi..	37	0.05
A41.1 Sepsis due to other specified s..	36	0.05

B02.8 Zoster with other complications	36	0.05
J12.9 Viral pneumonia, unspecified	36	0.05
L08.0 Pyoderma	36	0.05
A49.0 Staphylococcal infection, unspe..	35	0.05
B00.5 Herpesviral ocular disease	35	0.05
B85 Pediculosis and phthiriasis	35	0.05
J04.2 Acute laryngotracheitis	35	0.05
L02.8 Cutaneous abscess, furuncle and..	35	0.05
B97.7 Papillomavirus as the cause of ..	34	0.04
K02.9 Dental caries, unspecified	34	0.04
B33.8 Other specified viral diseases	33	0.04
M71.0 Abscess of bursa	33	0.04
A04.6 Enteritis due to Yersinia enter..	32	0.04
B01.8 Varicella with other complicati..	31	0.04
H70.0 Acute mastoiditis	31	0.04
M86.6 Other chronic osteomyelitis	31	0.04
A02.1 Salmonella sepsis	30	0.04
A16.5 Tuberculous pleurisy, without m..	30	0.04
H60.1 Cellulitis of external ear	30	0.04
A59.0 Urogenital trichomoniasis	29	0.04
H60.0 Abscess of external ear	28	0.04
J85.2 Abscess of lung without pneumonia	28	0.04
N73.0 Acute parametritis and pelvic c..	28	0.04
A05.9 Bacterial foodborne intoxicatio..	26	0.03
A37 Whooping cough	26	0.03
B16.9 Acute hepatitis B without delta..	26	0.03
B18.1 Chronic viral hepatitis B witho..	26	0.03
J10.8 Influenza with other manifestat..	26	0.03
A41.2 Sepsis due to unspecified staph..	25	0.03
J86.0 Pyothorax with fistula	24	0.03
K63.0 Abscess of intestine	24	0.03
K75.0 Abscess of liver	24	0.03
B02.3 Zoster ocular disease	23	0.03
M00.0 Staphylococcal arthritis and po..	23	0.03
O98.9 Unspecified maternal infectious..	23	0.03
H44.0 Purulent endophthalmitis	22	0.03
I30.1 Infective pericarditis	22	0.03
J21.9 Acute bronchiolitis, unspecified	22	0.03
O86.3 Other genitourinary tract infec..	22	0.03
K11.3 Abscess of salivary gland	21	0.03
M65.0 Abscess of tendon sheath	21	0.03
A18.0 Tuberculosis of bones and joints	20	0.03
A21.0 Ulceroglandular tularaemia	20	0.03
A21.9 Tularaemia, unspecified	20	0.03
A54.1 Gonococcal infection of lower g..	20	0.03
B34.0 Adenovirus infection, unspecifi..	20	0.03
K61.2 Anorectal abscess	20	0.03
L03.3 Cellulitis of trunk	20	0.03

B50.9 Plasmodium falciparum malaria, ..	19	0.02
N76.0 Acute vaginitis	19	0.02
B26.9 Mumps without complication	18	0.02
J04.1 Acute tracheitis	18	0.02
J17.3 Pneumonia in parasitic diseases	18	0.02
K04.4 Acute apical periodontitis of p..	18	0.02
A48.8 Other specified bacterial disea..	17	0.02
B01 Varicella [chickenpox]	17	0.02
B25.8 Other cytomegaloviral diseases	17	0.02
B25.9 Cytomegaloviral disease, unspec..	17	0.02
I42.1 Obstructive hypertrophic cardio..	17	0.02
N15.1 Renal and perinephric abscess	17	0.02
N74.4 Female chlamydial pelvic inflam..	17	0.02
A07 Other protozoal intestinal diseases	16	0.02
A41.4 Sepsis due to anaerobes	16	0.02
B17.1 Acute hepatitis C	16	0.02
B23.1 HIV disease resulting in (persi..	16	0.02
B37.3 Candidiasis of vulva and vagina	16	0.02
B37.7 Candidal sepsis	16	0.02
J86 Pyothorax	16	0.02
O98.4 Viral hepatitis complicating pr..	16	0.02
A15.1 Tuberculosis of lung, confirmed..	15	0.02
A40.1 Sepsis due to streptococcus, gr..	15	0.02
B27.1 Cytomegaloviral mononucleosis	15	0.02
B58.9 Toxoplasmosis, unspecified	15	0.02
L01.0 Impetigo [any organism] [any si..	15	0.02
M46.3 Infection of intervertebral dis..	15	0.02
N45.0 Orchitis, epididymitis and epid..	15	0.02
A02.9 Salmonella infection, unspecified	14	0.02
A51.0 Primary genital syphilis	14	0.02
A51.3 Secondary syphilis of skin and ..	14	0.02
B37.8 Candidiasis of other sites	14	0.02
K57.4 Diverticular disease of both sm..	14	0.02
O91.0 Infection of nipple associated ..	14	0.02
A03.3 Shigellosis due to Shigella son..	13	0.02
B00.8 Other forms of herpesviral infe..	13	0.02
B00.9 Herpesviral infection, unspecif..	13	0.02
B35.1 Tinea unguium	13	0.02
B59 Pneumocystosis	13	0.02
B97.0 Adenovirus as the cause of dise..	13	0.02
J03.8 Acute tonsillitis due to other ..	13	0.02
L03.8 Cellulitis of other sites	13	0.02
N34.0 Urethral abscess	13	0.02
B24 Unspecified human immunodeficienc..	12	0.02
B30 Viral conjunctivitis	12	0.02
B37.0 Candidal stomatitis	12	0.02
B70.0 Diphyllobothriasis	12	0.02
J85.1 Abscess of lung with pneumonia	12	0.02

L70.1 Acne conglobata	12	0.02
M60.0 Infective myositis	12	0.02
O06.5 Unspecified abortion: Complete ..	12	0.02
A01.4 Paratyphoid fever, unspecified	11	0.01
A39.2 Acute meningococcaemia	11	0.01
A64 Unspecified sexually transmitted ..	11	0.01
B06 Rubella [German measles]	11	0.01
B26.0 Mumps orchitis	11	0.01
B26.3 Mumps pancreatitis	11	0.01
B34.1 Enterovirus infection, unspecif..	11	0.01
L01 Impetigo	11	0.01
L04.9 Acute lymphadenitis, unspecified	11	0.01
M46.2 Osteomyelitis of vertebra	11	0.01
A01.2 Paratyphoid fever B	10	0.01
A03.1 Shigellosis due to Shigella fle..	10	0.01
B33.2 Viral carditis	10	0.01
B54 Unspecified malaria	10	0.01
B58.0 Toxoplasma oculopathy	10	0.01
J14 Pneumonia due to Haemophilus infl..	10	0.01
J16.0 Chlamydial pneumonia	10	0.01
J20.5 Acute bronchitis due to respira..	10	0.01
K61.3 Ischiorectal abscess	10	0.01
L01.1 Impetiginization of other derma..	10	0.01
N13.6 Pyonephrosis	10	0.01
A06.9 Amoebiasis, unspecified	9	0.01
A39.4 Meningococcaemia, unspecified	9	0.01
A63.8 Other specified predominantly s..	9	0.01
A90 Dengue fever [classical dengue]	9	0.01
B00.7 Disseminated herpesviral disease	9	0.01
B08.8 Other specified viral infection..	9	0.01
K77.0 Liver disorders in infectious a..	9	0.01
M00.2 Other streptococcal arthritis a..	9	0.01
A02.2 Localized salmonella infections	8	0.01
A02.8 Other specified salmonella infe..	8	0.01
A15.6 Tuberculous pleurisy, confirmed..	8	0.01
A16.0 Tuberculosis of lung, bacteriol..	8	0.01
A16.9 Respiratory tuberculosis unspec..	8	0.01
A18.1 Tuberculosis of genitourinary s..	8	0.01
A32.8 Other forms of listeriosis	8	0.01
A49.1 Streptococcal infection, unspec..	8	0.01
B02.2 Zoster with other nervous syste..	8	0.01
B05.8 Measles with other complications	8	0.01
B53.0 Plasmodium ovale malaria	8	0.01
J20.8 Acute bronchitis due to other s..	8	0.01
J39.1 Other abscess of pharynx	8	0.01
M01.2 Arthritis in Lyme disease	8	0.01
N08.0 Glomerular disorders in infecti..	8	0.01
O07.0 Failed medical abortion, compli..	8	0.01

O07.5 Other and unspecified failed at..	8	0.01
A01.0 Typhoid fever	7	0.01
A31.0 Pulmonary mycobacterial infection	7	0.01
A74.8 Other chlamydial diseases	7	0.01
B36 Other superficial mycoses	7	0.01
B80 Enterobiasis	7	0.01
J20.1 Acute bronchitis due to Haemoph..	7	0.01
K02.8 Other dental caries	7	0.01
O05.0 Other abortion: Incomplete, com..	7	0.01
O05.5 Other abortion: Complete or uns..	7	0.01
Z21 Asymptomatic human immunodeficien..	7	0.01
A04.3 Enterohaemorrhagic Escherichia ..	6	0.01
A08.1 Acute gastroenteropathy due to ..	6	0.01
A08.2 Adenoviral enteritis	6	0.01
A18.3 Tuberculosis of intestines, per..	6	0.01
A32.7 Listerial sepsis	6	0.01
A48.0 Gas gangrene	6	0.01
A54.0 Gonococcal infection of lower g..	6	0.01
B08.3 Erythema infectiosum [fifth dis..	6	0.01
B18.9 Chronic viral hepatitis, unspec..	6	0.01
B25.1 Cytomegaloviral hepatitis	6	0.01
B35.3 Tinea pedis	6	0.01
B51.9 Plasmodium vivax malaria withou..	6	0.01
B67 Echinococcosis	6	0.01
H10.5 Blepharoconjunctivitis	6	0.01
H62.1 Otitis externa in viral disease..	6	0.01
J21.0 Acute bronchiolitis due to resp..	6	0.01
J85.3 Abscess of mediastinum	6	0.01
K05.0 Acute gingivitis	6	0.01
M65.1 Other infective (teno)synovitis	6	0.01
N77.0 Ulceration of vulva in infectio..	6	0.01
O06.0 Unspecified abortion: Incomplet..	6	0.01
A03.9 Shigellosis, unspecified	5	0.01
A06.0 Acute amoebic dysentery	5	0.01
A15.2 Tuberculosis of lung, confirmed..	5	0.01
A31 Infection due to other mycobacteria	5	0.01
A31.9 Mycobacterial infection, unspec..	5	0.01
A60.9 Anogenital herpesviral infectio..	5	0.01
B01.2 Varicella pneumonia	5	0.01
B02.7 Disseminated zoster	5	0.01
B08.5 Enteroviral vesicular pharyngitis	5	0.01
B37.4 Candidiasis of other urogenital..	5	0.01
B74 Filariasis	5	0.01
B77.9 Ascariasis, unspecified	5	0.01
B95.8 Unspecified staphylococcus as t..	5	0.01
B97.1 Enterovirus as the cause of dis..	5	0.01
H19.1 Herpesviral keratitis and kerat..	5	0.01
H19.2 Keratitis and keratoconjunctivi..	5	0.01

J12.1 Respiratory syncytial virus pne..	5	0.01
J15.2 Pneumonia due to staphylococcus	5	0.01
K57.8 Diverticular disease of intesti..	5	0.01
L04.2 Acute lymphadenitis of upper limb	5	0.01
L04.8 Acute lymphadenitis of other si..	5	0.01
M86.0 Acute haematogenous osteomyelitis	5	0.01
M86.2 Subacute osteomyelitis	5	0.01
A00.9 Cholera, unspecified	<5	<0.01
A01.1 Paratyphoid fever A	<5	<0.01
A01.3 Paratyphoid fever C	<5	<0.01
A03 Shigellosis	<5	<0.01
A03.0 Shigellosis due to Shigella dys..	<5	<0.01
A03.2 Shigellosis due to Shigella boy..	<5	<0.01
A04.0 Enteropathogenic Escherichia co..	<5	<0.01
A04.2 Enteroinvasive Escherichia coli..	<5	<0.01
A04.4 Other intestinal Escherichia co..	<5	<0.01
A05.0 Foodborne staphylococcal intoxi..	<5	<0.01
A05.1 Botulism	<5	<0.01
A05.2 Foodborne Clostridium perfringe..	<5	<0.01
A05.8 Other specified bacterial foodb..	<5	<0.01
A06.2 Amoebic nondysenteric colitis	<5	<0.01
A06.4 Amoebic liver abscess	<5	<0.01
A06.5 Amoebic lung abscess	<5	<0.01
A06.8 Amoebic infection of other sites	<5	<0.01
A07.0 Balantidiasis	<5	<0.01
A07.1 Giardiasis [lambliasis]	<5	<0.01
A07.2 Cryptosporidiosis	<5	<0.01
A07.3 Isosporiasis	<5	<0.01
A07.9 Protozoal intestinal disease, u..	<5	<0.01
A15.3 Tuberculosis of lung, confirmed..	<5	<0.01
A15.4 Tuberculosis of intrathoracic l..	<5	<0.01
A15.5 Tuberculosis of larynx, trachea..	<5	<0.01
A15.7 Primary respiratory tuberculosi..	<5	<0.01
A15.8 Other respiratory tuberculosis,..	<5	<0.01
A15.9 Respiratory tuberculosis unspec..	<5	<0.01
A16.1 Tuberculosis of lung, bacteriol..	<5	<0.01
A16.3 Tuberculosis of intrathoracic l..	<5	<0.01
A16.4 Tuberculosis of larynx, trachea..	<5	<0.01
A16.7 Primary respiratory tuberculosi..	<5	<0.01
A16.8 Other respiratory tuberculosis,..	<5	<0.01
A17.8 Other tuberculosis of nervous s..	<5	<0.01
A17.9 Tuberculosis of nervous system,..	<5	<0.01
A18.4 Tuberculosis of skin and subcut..	<5	<0.01
A18.8 Tuberculosis of other specified..	<5	<0.01
A19.0 Acute miliary tuberculosis of a..	<5	<0.01
A19.2 Acute miliary tuberculosis, uns..	<5	<0.01
A19.9 Miliary tuberculosis, unspecified	<5	<0.01
A20.9 Plague, unspecified	<5	<0.01

A21.1 Oculoglandular tularaemia	<5	<0.01
A21.2 Pulmonary tularaemia	<5	<0.01
A21.7 Generalized tularaemia	<5	<0.01
A21.8 Other forms of tularaemia	<5	<0.01
A22.0 Cutaneous anthrax	<5	<0.01
A22.2 Gastrointestinal anthrax	<5	<0.01
A23.9 Brucellosis, unspecified	<5	<0.01
A24.0 Glanders	<5	<0.01
A26.0 Cutaneous erysipeloid	<5	<0.01
A26.9 Erysipeloid, unspecified	<5	<0.01
A27 Leptospirosis	<5	<0.01
A27.0 Leptospirosis icterohaemorrhagica	<5	<0.01
A27.8 Other forms of leptospirosis	<5	<0.01
A28.2 Extraintestinal yersiniosis	<5	<0.01
A28.8 Other specified zoonotic bacter..	<5	<0.01
A28.9 Zoonotic bacterial disease, uns..	<5	<0.01
A30 Leprosy [Hansen disease]	<5	<0.01
A31.1 Cutaneous mycobacterial infection	<5	<0.01
A31.8 Other mycobacterial infections	<5	<0.01
A32.9 Listeriosis, unspecified	<5	<0.01
A35 Other tetanus	<5	<0.01
A36 Diphtheria	<5	<0.01
A37.0 Whooping cough due to Bordetell..	<5	<0.01
A37.9 Whooping cough, unspecified	<5	<0.01
A39 Meningococcal infection	<5	<0.01
A39.8 Other meningococcal infections	<5	<0.01
A39.9 Meningococcal infection, unspec..	<5	<0.01
A40 Streptococcal sepsis	<5	<0.01
A40.2 Sepsis due to streptococcus, gr..	<5	<0.01
A41 Other sepsis	<5	<0.01
A41.3 Sepsis due to Haemophilus influ..	<5	<0.01
A42 Actinomycosis	<5	<0.01
A42.0 Pulmonary actinomycosis	<5	<0.01
A42.1 Abdominal actinomycosis	<5	<0.01
A42.2 Cervicofacial actinomycosis	<5	<0.01
A42.7 Actinomycotic sepsis	<5	<0.01
A42.8 Other forms of actinomycosis	<5	<0.01
A42.9 Actinomycosis, unspecified	<5	<0.01
A44.1 Cutaneous and mucocutaneous bar..	<5	<0.01
A49.2 Haemophilus influenzae infectio..	<5	<0.01
A49.3 Mycoplasma infection, unspecifi..	<5	<0.01
A50 Congenital syphilis	<5	<0.01
A50.5 Other late congenital syphilis,..	<5	<0.01
A50.9 Congenital syphilis, unspecified	<5	<0.01
A51.2 Primary syphilis of other sites	<5	<0.01
A51.4 Other secondary syphilis	<5	<0.01
A51.5 Early syphilis, latent	<5	<0.01
A51.9 Early syphilis, unspecified	<5	<0.01

A52.0 Cardiovascular syphilis	<5	<0.01
A52.7 Other symptomatic late syphilis	<5	<0.01
A52.8 Late syphilis, latent	<5	<0.01
A53 Other and unspecified syphilis	<5	<0.01
A53.9 Syphilis, unspecified	<5	<0.01
A54 Gonococcal infection	<5	<0.01
A54.3 Gonococcal infection of eye	<5	<0.01
A54.4 Gonococcal infection of musculo..	<5	<0.01
A54.8 Other gonococcal infections	<5	<0.01
A55 Chlamydial lymphogranuloma (vener..	<5	<0.01
A56.2 Chlamydial infection of genitou..	<5	<0.01
A56.3 Chlamydial infection of anus an..	<5	<0.01
A56.8 Sexually transmitted chlamydial..	<5	<0.01
A58 Granuloma inguinale	<5	<0.01
A60.1 Herpesviral infection of perian..	<5	<0.01
A66.1 Multiple papillomata and wet cr..	<5	<0.01
A66.3 Hyperkeratosis of yaws	<5	<0.01
A67 Pinta [carate]	<5	<0.01
A67.1 Intermediate lesions of pinta	<5	<0.01
A68 Relapsing fevers	<5	<0.01
A69.0 Necrotizing ulcerative stomatitis	<5	<0.01
A69.1 Other Vincent infections	<5	<0.01
A69.9 Spirochaetal infection, unspeci..	<5	<0.01
A71 Trachoma	<5	<0.01
A75.0 Epidemic louse-borne typhus fev..	<5	<0.01
A75.9 Typhus fever, unspecified	<5	<0.01
A77.1 Spotted fever due to Rickettsia..	<5	<0.01
A78 Q fever	<5	<0.01
A79.9 Rickettsiosis, unspecified	<5	<0.01
A92.8 Other specified mosquito-borne ..	<5	<0.01
A96.0 Junin haemorrhagic fever	<5	<0.01
A98.0 Crimean-Congo haemorrhagic fever	<5	<0.01
A98.1 Omsk haemorrhagic fever	<5	<0.01
A98.3 Marburg virus disease	<5	<0.01
A98.4 Ebola virus disease	<5	<0.01
A99 Unspecified viral haemorrhagic fe..	<5	<0.01
B02 Zoster [herpes zoster]	<5	<0.01
B05 Measles	<5	<0.01
B05.3 Measles complicated by otitis m..	<5	<0.01
B06.8 Rubella with other complications	<5	<0.01
B08.0 Other orthopoxvirus infections	<5	<0.01
B08.4 Enteroviral vesicular stomatiti..	<5	<0.01
B16.1 Acute hepatitis B with delta-ag..	<5	<0.01
B17.2 Acute hepatitis E	<5	<0.01
B17.8 Other specified acute viral hep..	<5	<0.01
B18.0 Chronic viral hepatitis B with ..	<5	<0.01
B20.0 HIV disease resulting in mycoba..	<5	<0.01
B20.6 HIV disease resulting in Pneumo..	<5	<0.01

B21.2 HIV disease resulting in other ..	<5	<0.01
B23.8 HIV disease resulting in other ..	<5	<0.01
B25.0 Cytomegaloviral pneumonitis	<5	<0.01
B26 Mumps	<5	<0.01
B26.8 Mumps with other complications	<5	<0.01
B27.8 Other infectious mononucleosis	<5	<0.01
B30.8 Other viral conjunctivitis	<5	<0.01
B33.0 Epidemic myalgia	<5	<0.01
B34.3 Parvovirus infection, unspecifi..	<5	<0.01
B35.0 Tinea barbae and tinea capitis	<5	<0.01
B35.2 Tinea manuum	<5	<0.01
B35.4 Tinea corporis	<5	<0.01
B35.6 Tinea cruris	<5	<0.01
B35.9 Dermatophytosis, unspecified	<5	<0.01
B36.0 Pityriasis versicolor	<5	<0.01
B36.9 Superficial mycosis, unspecified	<5	<0.01
B37.2 Candidiasis of skin and nail	<5	<0.01
B37.9 Candidiasis, unspecified	<5	<0.01
B38 Coccidioidomycosis	<5	<0.01
B38.0 Acute pulmonary coccidioidomyco..	<5	<0.01
B39.3 Disseminated histoplasmosis cap..	<5	<0.01
B39.4 Histoplasmosis capsulati, unspe..	<5	<0.01
B41.0 Pulmonary paracoccidioidomycosis	<5	<0.01
B43.9 Chromomycosis, unspecified	<5	<0.01
B44.9 Aspergillosis, unspecified	<5	<0.01
B47.9 Mycetoma, unspecified	<5	<0.01
B48.1 Rhinosporidiosis	<5	<0.01
B49 Unspecified mycosis	<5	<0.01
B50.0 Plasmodium falciparum malaria w..	<5	<0.01
B50.8 Other severe and complicated Pl..	<5	<0.01
B51 Plasmodium vivax malaria	<5	<0.01
B51.0 Plasmodium vivax malaria with r..	<5	<0.01
B52.0 Plasmodium malariae malaria wit..	<5	<0.01
B52.9 Plasmodium malariae malaria wit..	<5	<0.01
B55.2 Mucocutaneous leishmaniasis	<5	<0.01
B58.8 Toxoplasmosis with other organ ..	<5	<0.01
B60.8 Other specified protozoal disea..	<5	<0.01
B65.0 Schistosomiasis due to Schistos..	<5	<0.01
B65.1 Schistosomiasis due to Schistos..	<5	<0.01
B65.2 Schistosomiasis due to Schistos..	<5	<0.01
B65.9 Schistosomiasis, unspecified	<5	<0.01
B66 Other fluke infections	<5	<0.01
B67.3 Echinococcus granulosus infecti..	<5	<0.01
B67.8 Echinococcosis, unspecified, of..	<5	<0.01
B67.9 Echinococcosis, other and unspe..	<5	<0.01
B68.0 Taenia solium taeniasis	<5	<0.01
B68.1 Taenia saginata taeniasis	<5	<0.01
B68.9 Taeniasis, unspecified	<5	<0.01

B71.0 Hymenolepiasis	<5	<0.01
B75 Trichinellosis	<5	<0.01
B76 Hookworm diseases	<5	<0.01
B78.0 Intestinal strongyloidiasis	<5	<0.01
B79 Trichuriasis	<5	<0.01
B81.0 Anisakiasis	<5	<0.01
B81.4 Mixed intestinal helminthiases	<5	<0.01
B82.9 Intestinal parasitism, unspecif..	<5	<0.01
B83.0 Visceral larva migrans	<5	<0.01
B83.9 Helminthiasis, unspecified	<5	<0.01
B85.1 Pediculosis due to Pediculus hu..	<5	<0.01
B87.1 Wound myiasis	<5	<0.01
B87.9 Myiasis, unspecified	<5	<0.01
B95.0 Streptococcus, group A, as the ..	<5	<0.01
B95.1 Streptococcus, group B, as the ..	<5	<0.01
B95.3 Streptococcus pneumoniae as the..	<5	<0.01
B95.4 Other streptococcus as the caus..	<5	<0.01
B95.5 Unspecified streptococcus as th..	<5	<0.01
B95.6 Staphylococcus aureus as the ca..	<5	<0.01
B95.7 Other staphylococcus as the cau..	<5	<0.01
B96.1 Klebsiella pneumoniae [K. pneum..	<5	<0.01
B96.2 Escherichia coli [E. coli] as t..	<5	<0.01
B96.3 Haemophilus influenzae [H. infl..	<5	<0.01
B96.5 Pseudomonas (aeruginosa) as the..	<5	<0.01
B96.8 Other specified bacterial agent..	<5	<0.01
B97.4 Respiratory syncytial virus as ..	<5	<0.01
D73.3 Abscess of spleen	<5	<0.01
H01.0 Blepharitis	<5	<0.01
H19.0 Scleritis and episcleritis in d..	<5	<0.01
H22.0 Iridocyclitis in infectious and..	<5	<0.01
H32.0 Chorioretinal inflammation in i..	<5	<0.01
H60.2 Malignant otitis externa	<5	<0.01
I32.0 Pericarditis in bacterial disea..	<5	<0.01
I41.1 Myocarditis in viral diseases c..	<5	<0.01
J02.8 Acute pharyngitis due to other ..	<5	<0.01
J03 Acute tonsillitis	<5	<0.01
J10 Influenza due to other identified..	<5	<0.01
J11 Influenza, virus not identified	<5	<0.01
J12.0 Adenoviral pneumonia	<5	<0.01
J12.2 Parainfluenza virus pneumonia	<5	<0.01
J12.8 Other viral pneumonia	<5	<0.01
J15.0 Pneumonia due to Klebsiella pne..	<5	<0.01
J15.1 Pneumonia due to Pseudomonas	<5	<0.01
J15.3 Pneumonia due to streptococcus,..	<5	<0.01
J15.4 Pneumonia due to other streptoc..	<5	<0.01
J15.5 Pneumonia due to Escherichia coli	<5	<0.01
J15.6 Pneumonia due to other aerobic ..	<5	<0.01
J17.0 Pneumonia in bacterial diseases..	<5	<0.01

J17.1 Pneumonia in viral diseases cla..	<5	<0.01
J17.2 Pneumonia in mycoses	<5	<0.01
J18.2 Hypostatic pneumonia, unspecified	<5	<0.01
J20 Acute bronchitis	<5	<0.01
J20.2 Acute bronchitis due to strepto..	<5	<0.01
J20.4 Acute bronchitis due to parainf..	<5	<0.01
J20.6 Acute bronchitis due to rhinovi..	<5	<0.01
J21.8 Acute bronchiolitis due to othe..	<5	<0.01
K02.0 Caries limited to enamel	<5	<0.01
K02.2 Caries of cementum	<5	<0.01
K02.3 Arrested dental caries	<5	<0.01
K23.0 Tuberculous oesophagitis	<5	<0.01
K61.4 Intrasphincteric abscess	<5	<0.01
K67.3 Tuberculous peritonitis	<5	<0.01
K67.8 Other disorders of peritoneum i..	<5	<0.01
L00 Staphylococcal scalded skin syndr..	<5	<0.01
L04.1 Acute lymphadenitis of trunk	<5	<0.01
L04.3 Acute lymphadenitis of lower limb	<5	<0.01
L08.1 Erythrasma	<5	<0.01
M00.8 Arthritis and polyarthritis due..	<5	<0.01
M01.0 Meningococcal arthritis	<5	<0.01
M01.1 Tuberculous arthritis	<5	<0.01
M01.3 Arthritis in other bacterial di..	<5	<0.01
M46.5 Other infective spondylopathies	<5	<0.01
M49.0 Tuberculosis of spine	<5	<0.01
M49.1 Brucella spondylitis	<5	<0.01
M49.3 Spondylopathy in other infectio..	<5	<0.01
M63.0 Myositis in bacterial diseases ..	<5	<0.01
M72.6 Necrotizing fasciitis	<5	<0.01
M86.3 Chronic multifocal osteomyelitis	<5	<0.01
M86.4 Chronic osteomyelitis with drai..	<5	<0.01
M86.8 Other osteomyelitis	<5	<0.01
N16.0 Renal tubulo-interstitial disor..	<5	<0.01
O23.2 Infections of urethra in pregna..	<5	<0.01
O23.3 Infections of other parts of ur..	<5	<0.01
O98.0 Tuberculosis complicating pregn..	<5	<0.01
O98.1 Syphilis complicating pregnancy..	<5	<0.01
O98.6 Protozoal diseases complicating..	<5	<0.01
P36.9 Bacterial sepsis of newborn, un..	<5	<0.01
P37.1 Congenital toxoplasmosis	<5	<0.01
P37.9 Congenital infectious and paras..	<5	<0.01
P39.0 Neonatal infective mastitis	<5	<0.01
P39.1 Neonatal conjunctivitis and dac..	<5	<0.01
P39.9 Infection specific to the perin..	<5	<0.01
R57.2 Septic shock	<5	<0.01
**Total**	**76 301**	**100**

eTable 23. Distribution of acute infections in primary analysis (Finnish multicohort sample)

ICD-10 code and description	N	%
K35 Acute appendicitis	12 007	16.32
J18.9 Pneumonia, unspecified	6204	8.43
A09 Other gastroenteritis and colitis..	5703	7.75
N10 Acute tubulo-interstitial nephritis	3650	4.96
A46 Erysipelas	3167	4.31
J03.9 Acute tonsillitis, unspecified	3138	4.27
J36 Peritonsillar abscess	3090	4.20
J15.9 Bacterial pneumonia, unspecified	2284	3.10
N70.0 Acute salpingitis and oophoritis	1981	2.69
O85 Puerperal sepsis	1749	2.38
J20.9 Acute bronchitis, unspecified	1185	1.61
N39.0 Urinary tract infection, site n..	1081	1.47
J01.0 Acute maxillary sinusitis	1063	1.45
A49.9 Bacterial infection, unspecified	1060	1.44
J03.0 Streptococcal tonsillitis	949	1.29
A08.4 Viral intestinal infection, uns..	835	1.14
J22 Unspecified acute lower respirato..	784	1.07
N71.0 Acute inflammatory disease of u..	730	0.99
J02.9 Acute pharyngitis, unspecified	704	0.96
J04.0 Acute laryngitis	684	0.93
B34.9 Viral infection, unspecified	623	0.85
A98.5 Haemorrhagic fever with renal s..	587	0.80
A08.5 Other specified intestinal infe..	493	0.67
K57.2 Diverticular disease of large i..	459	0.62
N30.0 Acute cystitis	449	0.61
J18.0 Bronchopneumonia, unspecified	448	0.61
A41.5 Sepsis due to other Gram-negati..	421	0.57
L05.9 Pilonidal cyst without abscess	398	0.54
O08.0 Genital tract and pelvic infect..	377	0.51
O03.5 Spontaneous abortion: Complete ..	368	0.50
K61.0 Anal abscess	339	0.46
A04.7 Enterocolitis due to Clostridiu..	311	0.42
H66.0 Acute suppurative otitis media	303	0.41
N75.1 Abscess of Bartholin gland	301	0.41
J11.1 Influenza with other respirator..	287	0.39
A41.9 Sepsis, unspecified	284	0.39
K04.7 Periapical abscess without sinus	281	0.38
B06.9 Rubella without complication	280	0.38
J13 Pneumonia due to Streptococcus pn..	277	0.38
N61 Inflammatory disorders of breast	258	0.35
J11.8 Influenza with other manifestat..	257	0.35
A69.2 Lyme disease	239	0.32
L04 Acute lymphadenitis	227	0.31
M00.9 Pyogenic arthritis, unspecified	214	0.29
N45.9 Orchitis, epididymitis and epid..	212	0.29

J10.1 Influenza with other respirator..	210	0.29
B99 Other and unspecified infectious ..	201	0.27
L03.0 Cellulitis of finger and toe	201	0.27
A04.5 Campylobacter enteritis	196	0.27
A87.9 Viral meningitis, unspecified	185	0.25
K12.2 Cellulitis and abscess of mouth	184	0.25
K65.0 Acute peritonitis	182	0.25
O23.5 Infections of the genital tract..	179	0.24
O23.0 Infections of kidney in pregnancy	177	0.24
K61 Abscess of anal and rectal regions	176	0.24
O03.0 Spontaneous abortion: Incomplet..	175	0.24
J05.1 Acute epiglottitis	174	0.24
L08.9 Local infection of skin and sub..	173	0.24
A41.0 Sepsis due to Staphylococcus au..	171	0.23
O86.0 Infection of obstetric surgical..	168	0.23
L02 Cutaneous abscess, furuncle and c..	165	0.22
O23.9 Other and unspecified genitouri..	162	0.22
A02.0 Salmonella enteritis	156	0.21
L02.4 Cutaneous abscess, furuncle and..	153	0.21
A41.8 Other specified sepsis	151	0.21
J15.8 Other bacterial pneumonia	148	0.20
B19.9 Unspecified viral hepatitis wit..	145	0.20
J12 Viral pneumonia, not elsewhere cl..	144	0.20
A89 Unspecified viral infection of ce..	137	0.19
B07 Viral warts	136	0.18
O86.1 Other infection of genital trac..	132	0.18
A87.0 Enteroviral meningitis	131	0.18
L05.0 Pilonidal cyst with abscess	126	0.17
G00.9 Bacterial meningitis, unspecified	125	0.17
O86.4 Pyrexia of unknown origin follo..	122	0.17
J18.1 Lobar pneumonia, unspecified	117	0.16
K05.2 Acute periodontitis	116	0.16
A40.3 Sepsis due to Streptococcus pne..	113	0.15
A02 Other salmonella infections	110	0.15
A86 Unspecified viral encephalitis	106	0.14
B97.8 Other viral agents as the cause..	102	0.14
L04.0 Acute lymphadenitis of face, he..	102	0.14
A56.1 Chlamydial infection of pelvipe..	100	0.14
L03.1 Cellulitis of other parts of limb	98	0.13
J15.7 Pneumonia due to Mycoplasma pne..	93	0.13
O23.4 Unspecified infection of urinar..	93	0.13
L02.2 Cutaneous abscess, furuncle and..	92	0.13
O04.0 Medical abortion: Incomplete, c..	90	0.12
B05.9 Measles without complication	89	0.12
A49.8 Other bacterial infections of u..	87	0.12
A04.9 Bacterial intestinal infection,..	86	0.12
O23.1 Infections of bladder in pregna..	85	0.12
L02.3 Cutaneous abscess, furuncle and..	82	0.11

H10.0 Mucopurulent conjunctivitis	81	0.11
J86.9 Pyothorax without fistula	80	0.11
A95 Yellow fever	79	0.11
H00.1 Chalazion	79	0.11
J16.8 Pneumonia due to other specifie..	77	0.10
B34.8 Other viral infections of unspe..	76	0.10
M71.1 Other infective bursitis	76	0.10
L03.9 Cellulitis, unspecified	75	0.10
O86.2 Urinary tract infection followi..	75	0.10
H60.3 Other infective otitis externa	74	0.10
A54.9 Gonococcal infection, unspecified	73	0.10
N41.0 Acute prostatitis	73	0.10
A08.3 Other viral enteritis	72	0.10
M86.9 Osteomyelitis, unspecified	72	0.10
I33.0 Acute and subacute infective en..	71	0.10
L08.8 Other specified local infection..	71	0.10
G03.9 Meningitis, unspecified	70	0.10
N76.4 Abscess of vulva	69	0.09
J39.0 Retropharyngeal and parapharyng..	68	0.09
B26.2 Mumps encephalitis	67	0.09
O04.5 Medical abortion: Complete or u..	67	0.09
K57.0 Diverticular disease of small i..	66	0.09
J09 Influenza due to certain identifi..	63	0.09
M86.1 Other acute osteomyelitis	63	0.09
O75.3 Other infection during labour	61	0.08
O86.8 Other specified puerperal infec..	61	0.08
A04.8 Other specified bacterial intes..	60	0.08
O98.8 Other maternal infectious and p..	60	0.08
J10.0 Influenza with pneumonia, other..	59	0.08
A54.2 Gonococcal pelviperitonitis and..	58	0.08
N77.1 Vaginitis, vulvitis and vulvova..	57	0.08
H62.0 Otitis externa in bacterial dis..	56	0.08
L02.1 Cutaneous abscess, furuncle and..	55	0.07
A74.9 Chlamydial infection, unspecified	54	0.07
B09 Unspecified viral infection chara..	54	0.07
L02.9 Cutaneous abscess, furuncle and..	54	0.07
O91.1 Abscess of breast associated wi..	52	0.07
N73.3 Female acute pelvic peritonitis	51	0.07
O98.5 Other viral diseases complicati..	51	0.07
A38 Scarlet fever	49	0.07
A40.8 Other streptococcal sepsis	47	0.06
H00.0 Hordeolum and other deep inflam..	46	0.06
A56.0 Chlamydial infection of lower g..	44	0.06
H05.0 Acute inflammation of orbit	44	0.06
G00.8 Other bacterial meningitis	42	0.06
A40.9 Streptococcal sepsis, unspecified	41	0.06
B08.1 Molluscum contagiosum	41	0.06
J20.0 Acute bronchitis due to Mycopla..	41	0.06

B15.9 Hepatitis A without hepatic coma	40	0.05
J11.0 Influenza with pneumonia, virus..	40	0.05
L03.2 Cellulitis of face	40	0.05
A40.0 Sepsis due to streptococcus, gr..	39	0.05
I40.0 Infective myocarditis	39	0.05
L02.0 Cutaneous abscess, furuncle and..	39	0.05
A08.0 Rotaviral enteritis	38	0.05
A41.1 Sepsis due to other specified s..	38	0.05
J02.0 Streptococcal pharyngitis	38	0.05
K61.1 Rectal abscess	38	0.05
L08.0 Pyoderma	38	0.05
O98.3 Other infections with a predomi..	38	0.05
J12.9 Viral pneumonia, unspecified	37	0.05
J18.8 Other pneumonia, organism unspe..	37	0.05
A49.0 Staphylococcal infection, unspe..	35	0.05
J04.2 Acute laryngotracheitis	35	0.05
L02.8 Cutaneous abscess, furuncle and..	35	0.05
M71.0 Abscess of bursa	35	0.05
B33.8 Other specified viral diseases	34	0.05
B59 Pneumocystosis	33	0.04
H60.1 Cellulitis of external ear	33	0.04
M86.6 Other chronic osteomyelitis	33	0.04
A04.6 Enteritis due to Yersinia enter..	32	0.04
H70.0 Acute mastoiditis	32	0.04
G05.1 Encephalitis, myelitis and ence..	31	0.04
A02.1 Salmonella sepsis	30	0.04
A39.0 Meningococcal meningitis	30	0.04
A59.0 Urogenital trichomoniasis	29	0.04
B16.9 Acute hepatitis B without delta..	29	0.04
G00.0 Haemophilus meningitis	29	0.04
H60.0 Abscess of external ear	29	0.04
J85.2 Abscess of lung without pneumonia	29	0.04
N73.0 Acute parametritis and pelvic c..	28	0.04
A37 Whooping cough	26	0.04
J10.8 Influenza with other manifestat..	26	0.04
J86.0 Pyothorax with fistula	26	0.04
M00.0 Staphylococcal arthritis and po..	26	0.04
A05.9 Bacterial foodborne intoxicatio..	25	0.03
A41.2 Sepsis due to unspecified staph..	25	0.03
H44.0 Purulent endophthalmitis	24	0.03
I30.1 Infective pericarditis	24	0.03
K63.0 Abscess of intestine	24	0.03
J21.9 Acute bronchiolitis, unspecified	23	0.03
K04.4 Acute apical periodontitis of p..	23	0.03
K75.0 Abscess of liver	23	0.03
O98.4 Viral hepatitis complicating pr..	23	0.03
G00.1 Pneumococcal meningitis	22	0.03
M65.0 Abscess of tendon sheath	22	0.03

O86.3 Other genitourinary tract infec..	22	0.03
O98.9 Unspecified maternal infectious..	22	0.03
B34.0 Adenovirus infection, unspecifi..	21	0.03
K11.3 Abscess of salivary gland	21	0.03
K61.2 Anorectal abscess	21	0.03
L03.3 Cellulitis of trunk	21	0.03
A21.9 Tularaemia, unspecified	20	0.03
A54.1 Gonococcal infection of lower g..	20	0.03
B17.1 Acute hepatitis C	20	0.03
B50.9 Plasmodium falciparum malaria, ..	20	0.03
G02.0 Meningitis in viral diseases cl..	20	0.03
G06.0 Intracranial abscess and granul..	20	0.03
N76.0 Acute vaginitis	20	0.03
A21.0 Ulceroglandular tularaemia	19	0.03
A48.8 Other specified bacterial disea..	18	0.02
A80 Acute poliomyelitis	18	0.02
G01 Meningitis in bacterial diseases ..	18	0.02
I42.1 Obstructive hypertrophic cardio..	18	0.02
J04.1 Acute tracheitis	18	0.02
N74.4 Female chlamydial pelvic inflam..	18	0.02
A88.8 Other specified viral infection..	17	0.02
B26.9 Mumps without complication	17	0.02
J86 Pyothorax	17	0.02
N15.1 Renal and perinephric abscess	17	0.02
A07 Other protozoal intestinal diseases	16	0.02
A40.1 Sepsis due to streptococcus, gr..	16	0.02
A41.4 Sepsis due to anaerobes	16	0.02
L01.0 Impetigo [any organism] [any si..	16	0.02
L03.8 Cellulitis of other sites	15	0.02
M46.3 Infection of intervertebral dis..	15	0.02
N45.0 Orchitis, epididymitis and epid..	15	0.02
O91.0 Infection of nipple associated ..	15	0.02
A02.9 Salmonella infection, unspecified	14	0.02
K57.4 Diverticular disease of both sm..	14	0.02
A03.3 Shigellosis due to Shigella son..	13	0.02
B97.0 Adenovirus as the cause of dise..	13	0.02
J03.8 Acute tonsillitis due to other ..	13	0.02
N34.0 Urethral abscess	13	0.02
A01.4 Paratyphoid fever, unspecified	12	0.02
A64 Unspecified sexually transmitted ..	12	0.02
B30 Viral conjunctivitis	12	0.02
G06.1 Intraspinal abscess and granuloma	12	0.02
J14 Pneumonia due to Haemophilus infl..	12	0.02
J85.1 Abscess of lung with pneumonia	12	0.02
L01 Impetigo	12	0.02
M60.0 Infective myositis	12	0.02
O06.5 Unspecified abortion: Complete ..	12	0.02
B06 Rubella [German measles]	11	0.01

B26.0 Mumps orchitis	11	0.01
B34.1 Enterovirus infection, unspecif..	11	0.01
L04.9 Acute lymphadenitis, unspecified	11	0.01
M46.2 Osteomyelitis of vertebra	11	0.01
A01.2 Paratyphoid fever B	10	0.01
A03.1 Shigellosis due to Shigella fle..	10	0.01
A39.2 Acute meningococcaemia	10	0.01
A63.8 Other specified predominantly s..	10	0.01
A87.8 Other viral meningitis	10	0.01
B54 Unspecified malaria	10	0.01
J16.0 Chlamydial pneumonia	10	0.01
J20.5 Acute bronchitis due to respira..	10	0.01
K61.3 Ischiorectal abscess	10	0.01
L01.1 Impetiginization of other derma..	10	0.01
N13.6 Pyonephrosis	10	0.01
A06.9 Amoebiasis, unspecified	9	0.01
A90 Dengue fever [classical dengue]	9	0.01
B08.8 Other specified viral infection..	9	0.01
B33.2 Viral carditis	9	0.01
K77.0 Liver disorders in infectious a..	9	0.01
M00.2 Other streptococcal arthritis a..	9	0.01
M01.2 Arthritis in Lyme disease	9	0.01
A02.2 Localized salmonella infections	8	0.01
A02.8 Other specified salmonella infe..	8	0.01
A32.8 Other forms of listeriosis	8	0.01
A39.4 Meningococcaemia, unspecified	8	0.01
A49.1 Streptococcal infection, unspec..	8	0.01
A54.0 Gonococcal infection of lower g..	8	0.01
B05.8 Measles with other complications	8	0.01
B26.3 Mumps pancreatitis	8	0.01
J20.8 Acute bronchitis due to other s..	8	0.01
J39.1 Other abscess of pharynx	8	0.01
N77.0 Ulceration of vulva in infectio..	8	0.01
O07.0 Failed medical abortion, compli..	8	0.01
O07.5 Other and unspecified failed at..	8	0.01
A01.0 Typhoid fever	7	0.01
A84.8 Other tick-borne viral encephal..	7	0.01
J20.1 Acute bronchitis due to Haemoph..	7	0.01
K05.0 Acute gingivitis	7	0.01
N08.0 Glomerular disorders in infecti..	7	0.01
O05.0 Other abortion: Incomplete, com..	7	0.01
O05.5 Other abortion: Complete or uns..	7	0.01
A04.3 Enterohaemorrhagic Escherichia ..	6	0.01
A06.0 Acute amoebic dysentery	6	0.01
A08.1 Acute gastroenteropathy due to ..	6	0.01
A08.2 Adenoviral enteritis	6	0.01
A32.7 Listerial sepsis	6	0.01
A48.0 Gas gangrene	6	0.01

A74.8 Other chlamydial diseases	6	0.01
B08.3 Erythema infectiosum [fifth dis..	6	0.01
B19.0 Unspecified viral hepatitis wit..	6	0.01
H10.5 Blepharoconjunctivitis	6	0.01
H62.1 Otitis externa in viral disease..	6	0.01
J21.0 Acute bronchiolitis due to resp..	6	0.01
J85.3 Abscess of mediastinum	6	0.01
M65.1 Other infective (teno)synovitis	6	0.01
O06.0 Unspecified abortion: Incomplet..	6	0.01
A03.9 Shigellosis, unspecified	5	0.01
A84.9 Tick-borne viral encephalitis, ..	5	0.01
B08.5 Enteroviral vesicular pharyngitis	5	0.01
B95.8 Unspecified staphylococcus as t..	5	0.01
B97.1 Enterovirus as the cause of dis..	5	0.01
G03.8 Meningitis due to other specifi..	5	0.01
H19.2 Keratitis and keratoconjunctivi..	5	0.01
J12.1 Respiratory syncytial virus pne..	5	0.01
J15.2 Pneumonia due to staphylococcus	5	0.01
K57.8 Diverticular disease of intesti..	5	0.01
L04.2 Acute lymphadenitis of upper limb	5	0.01
L04.8 Acute lymphadenitis of other si..	5	0.01
M86.0 Acute haematogenous osteomyelitis	5	0.01
M86.2 Subacute osteomyelitis	5	0.01
A00.9 Cholera, unspecified	<5	<0.01
A01.1 Paratyphoid fever A	<5	<0.01
A01.3 Paratyphoid fever C	<5	<0.01
A03 Shigellosis	<5	<0.01
A03.0 Shigellosis due to Shigella dys..	<5	<0.01
A03.2 Shigellosis due to Shigella boy..	<5	<0.01
A04.0 Enteropathogenic Escherichia co..	<5	<0.01
A04.2 Enteroinvasive Escherichia coli..	<5	<0.01
A04.4 Other intestinal Escherichia co..	<5	<0.01
A05.0 Foodborne staphylococcal intoxi..	<5	<0.01
A05.1 Botulism	<5	<0.01
A05.2 Foodborne Clostridium perfringe..	<5	<0.01
A05.8 Other specified bacterial foodb..	<5	<0.01
A06.2 Amoebic nondysenteric colitis	<5	<0.01
A06.4 Amoebic liver abscess	<5	<0.01
A06.5 Amoebic lung abscess	<5	<0.01
A06.8 Amoebic infection of other sites	<5	<0.01
A07.0 Balantidiasis	<5	<0.01
A07.1 Giardiasis [lambliasis]	<5	<0.01
A07.2 Cryptosporidiosis	<5	<0.01
A07.3 Isosporiasis	<5	<0.01
A07.9 Protozoal intestinal disease, u..	<5	<0.01
A20.9 Plague, unspecified	<5	<0.01
A21.1 Oculoglandular tularaemia	<5	<0.01
A21.2 Pulmonary tularaemia	<5	<0.01

A21.7 Generalized tularaemia	<5	<0.01
A21.8 Other forms of tularaemia	<5	<0.01
A22.0 Cutaneous anthrax	<5	<0.01
A22.2 Gastrointestinal anthrax	<5	<0.01
A23.9 Brucellosis, unspecified	<5	<0.01
A24.0 Glanders	<5	<0.01
A26.0 Cutaneous erysipeloid	<5	<0.01
A26.9 Erysipeloid, unspecified	<5	<0.01
A27 Leptospirosis	<5	<0.01
A27.0 Leptospirosis icterohaemorrhagica	<5	<0.01
A27.8 Other forms of leptospirosis	<5	<0.01
A27.9 Leptospirosis, unspecified	<5	<0.01
A28.2 Extraintestinal yersiniosis	<5	<0.01
A28.8 Other specified zoonotic bacter..	<5	<0.01
A28.9 Zoonotic bacterial disease, uns..	<5	<0.01
A32.9 Listeriosis, unspecified	<5	<0.01
A35 Other tetanus	<5	<0.01
A36 Diphtheria	<5	<0.01
A37.0 Whooping cough due to Bordetell..	<5	<0.01
A37.9 Whooping cough, unspecified	<5	<0.01
A39 Meningococcal infection	<5	<0.01
A39.8 Other meningococcal infections	<5	<0.01
A39.9 Meningococcal infection, unspec..	<5	<0.01
A40 Streptococcal sepsis	<5	<0.01
A40.2 Sepsis due to streptococcus, gr..	<5	<0.01
A41 Other sepsis	<5	<0.01
A41.3 Sepsis due to Haemophilus influ..	<5	<0.01
A49.2 Haemophilus influenzae infectio..	<5	<0.01
A49.3 Mycoplasma infection, unspecifi..	<5	<0.01
A54 Gonococcal infection	<5	<0.01
A54.3 Gonococcal infection of eye	<5	<0.01
A54.4 Gonococcal infection of musculo..	<5	<0.01
A54.8 Other gonococcal infections	<5	<0.01
A55 Chlamydial lymphogranuloma (vener..	<5	<0.01
A56.2 Chlamydial infection of genitou..	<5	<0.01
A56.3 Chlamydial infection of anus an..	<5	<0.01
A56.8 Sexually transmitted chlamydial..	<5	<0.01
A68 Relapsing fevers	<5	<0.01
A69.0 Necrotizing ulcerative stomatitis	<5	<0.01
A69.1 Other Vincent infections	<5	<0.01
A69.9 Spirochaetal infection, unspeci..	<5	<0.01
A70 Chlamydia psittaci infection	<5	<0.01
A71 Trachoma	<5	<0.01
A75.0 Epidemic louse-borne typhus fev..	<5	<0.01
A75.9 Typhus fever, unspecified	<5	<0.01
A77.1 Spotted fever due to Rickettsia..	<5	<0.01
A78 Q fever	<5	<0.01
A79.9 Rickettsiosis, unspecified	<5	<0.01

A80.1 Acute paralytic poliomyelitis, ..	<5	<0.01
A80.3 Acute paralytic poliomyelitis, ..	<5	<0.01
A83.0 Japanese encephalitis	<5	<0.01
A83.6 Rocio virus disease	<5	<0.01
A83.8 Other mosquito-borne viral ence..	<5	<0.01
A84 Tick-borne viral encephalitis	<5	<0.01
A84.1 Central European tick-borne enc..	<5	<0.01
A85.0 Enteroviral encephalitis	<5	<0.01
A85.1 Adenoviral encephalitis	<5	<0.01
A85.2 Arthropod-borne viral encephali..	<5	<0.01
A85.8 Other specified viral encephali..	<5	<0.01
A87.1 Adenoviral meningitis	<5	<0.01
A87.2 Lymphocytic choriomeningitis	<5	<0.01
A88.1 Epidemic vertigo	<5	<0.01
A92.8 Other specified mosquito-borne ..	<5	<0.01
A96.0 Junin haemorrhagic fever	<5	<0.01
A98.0 Crimean-Congo haemorrhagic fever	<5	<0.01
A98.1 Omsk haemorrhagic fever	<5	<0.01
A98.3 Marburg virus disease	<5	<0.01
A98.4 Ebola virus disease	<5	<0.01
A99 Unspecified viral haemorrhagic fe..	<5	<0.01
B05 Measles	<5	<0.01
B05.3 Measles complicated by otitis m..	<5	<0.01
B06.8 Rubella with other complications	<5	<0.01
B08.0 Other orthopoxvirus infections	<5	<0.01
B08.4 Enteroviral vesicular stomatiti..	<5	<0.01
B15.0 Hepatitis A with hepatic coma	<5	<0.01
B16.1 Acute hepatitis B with delta-ag..	<5	<0.01
B17.2 Acute hepatitis E	<5	<0.01
B17.8 Other specified acute viral hep..	<5	<0.01
B26 Mumps	<5	<0.01
B26.1 Mumps meningitis	<5	<0.01
B26.8 Mumps with other complications	<5	<0.01
B27.8 Other infectious mononucleosis	<5	<0.01
B30.8 Other viral conjunctivitis	<5	<0.01
B33.0 Epidemic myalgia	<5	<0.01
B34.3 Parvovirus infection, unspecifi..	<5	<0.01
B38 Coccidioidomycosis	<5	<0.01
B38.0 Acute pulmonary coccidioidomyco..	<5	<0.01
B39.4 Histoplasmosis capsulati, unspe..	<5	<0.01
B49 Unspecified mycosis	<5	<0.01
B50.0 Plasmodium falciparum malaria w..	<5	<0.01
B50.8 Other severe and complicated Pl..	<5	<0.01
B52.0 Plasmodium malariae malaria wit..	<5	<0.01
B52.9 Plasmodium malariae malaria wit..	<5	<0.01
B60.8 Other specified protozoal disea..	<5	<0.01
B66 Other fluke infections	<5	<0.01
B81.0 Anisakiasis	<5	<0.01

B81.4 Mixed intestinal helminthiases	<5	<0.01
B82.9 Intestinal parasitism, unspecif..	<5	<0.01
B83.0 Visceral larva migrans	<5	<0.01
B83.9 Helminthiasis, unspecified	<5	<0.01
B87.1 Wound myiasis	<5	<0.01
B87.9 Myiasis, unspecified	<5	<0.01
B95.0 Streptococcus, group A, as the ..	<5	<0.01
B95.1 Streptococcus, group B, as the ..	<5	<0.01
B95.3 Streptococcus pneumoniae as the..	<5	<0.01
B95.4 Other streptococcus as the caus..	<5	<0.01
B95.5 Unspecified streptococcus as th..	<5	<0.01
B95.6 Staphylococcus aureus as the ca..	<5	<0.01
B95.7 Other staphylococcus as the cau..	<5	<0.01
B96.1 Klebsiella pneumoniae [K. pneum..	<5	<0.01
B96.2 Escherichia coli [E. coli] as t..	<5	<0.01
B96.3 Haemophilus influenzae [H. infl..	<5	<0.01
B96.5 Pseudomonas (aeruginosa) as the..	<5	<0.01
B97.4 Respiratory syncytial virus as ..	<5	<0.01
D73.3 Abscess of spleen	<5	<0.01
G00.2 Streptococcal meningitis	<5	<0.01
G00.3 Staphylococcal meningitis	<5	<0.01
G03.0 Nonpyogenic meningitis	<5	<0.01
G03.1 Chronic meningitis	<5	<0.01
G04.2 Bacterial meningoencephalitis a..	<5	<0.01
G05.0 Encephalitis, myelitis and ence..	<5	<0.01
G05.2 Encephalitis, myelitis and ence..	<5	<0.01
G06 Intracranial and intraspinal absc..	<5	<0.01
G06.2 Extradural and subdural abscess..	<5	<0.01
H01.0 Blepharitis	<5	<0.01
H19.0 Scleritis and episcleritis in d..	<5	<0.01
H22.0 Iridocyclitis in infectious and..	<5	<0.01
H32.0 Chorioretinal inflammation in i..	<5	<0.01
H60.2 Malignant otitis externa	<5	<0.01
I32.0 Pericarditis in bacterial disea..	<5	<0.01
I41.0 Myocarditis in bacterial diseas..	<5	<0.01
I41.1 Myocarditis in viral diseases c..	<5	<0.01
I52.0 Other heart disorders in bacter..	<5	<0.01
J02.8 Acute pharyngitis due to other ..	<5	<0.01
J03 Acute tonsillitis	<5	<0.01
J10 Influenza due to other identified..	<5	<0.01
J11 Influenza, virus not identified	<5	<0.01
J12.0 Adenoviral pneumonia	<5	<0.01
J12.2 Parainfluenza virus pneumonia	<5	<0.01
J12.8 Other viral pneumonia	<5	<0.01
J15.0 Pneumonia due to Klebsiella pne..	<5	<0.01
J15.1 Pneumonia due to Pseudomonas	<5	<0.01
J15.3 Pneumonia due to streptococcus,..	<5	<0.01
J15.4 Pneumonia due to other streptoc..	<5	<0.01

J15.5 Pneumonia due to Escherichia coli	<5	<0.01
J15.6 Pneumonia due to other aerobic ..	<5	<0.01
J17.0 Pneumonia in bacterial diseases..	<5	<0.01
J17.1 Pneumonia in viral diseases cla..	<5	<0.01
J17.2 Pneumonia in mycoses	<5	<0.01
J18.2 Hypostatic pneumonia, unspecified	<5	<0.01
J20 Acute bronchitis	<5	<0.01
J20.2 Acute bronchitis due to strepto..	<5	<0.01
J20.4 Acute bronchitis due to parainf..	<5	<0.01
J20.6 Acute bronchitis due to rhinovi..	<5	<0.01
J21.8 Acute bronchiolitis due to othe..	<5	<0.01
K61.4 Intrasphincteric abscess	<5	<0.01
K67.8 Other disorders of peritoneum i..	<5	<0.01
L00 Staphylococcal scalded skin syndr..	<5	<0.01
L04.1 Acute lymphadenitis of trunk	<5	<0.01
L04.3 Acute lymphadenitis of lower limb	<5	<0.01
L08.1 Erythrasma	<5	<0.01
M00.8 Arthritis and polyarthritis due..	<5	<0.01
M01.0 Meningococcal arthritis	<5	<0.01
M01.3 Arthritis in other bacterial di..	<5	<0.01
M46.5 Other infective spondylopathies	<5	<0.01
M49.1 Brucella spondylitis	<5	<0.01
M49.3 Spondylopathy in other infectio..	<5	<0.01
M63.0 Myositis in bacterial diseases ..	<5	<0.01
M72.6 Necrotizing fasciitis	<5	<0.01
M86.3 Chronic multifocal osteomyelitis	<5	<0.01
M86.4 Chronic osteomyelitis with drai..	<5	<0.01
M86.8 Other osteomyelitis	<5	<0.01
N16.0 Renal tubulo-interstitial disor..	<5	<0.01
O23.2 Infections of urethra in pregna..	<5	<0.01
O23.3 Infections of other parts of ur..	<5	<0.01
O98.6 Protozoal diseases complicating..	<5	<0.01
P36.9 Bacterial sepsis of newborn, un..	<5	<0.01
P37.9 Congenital infectious and paras..	<5	<0.01
P39.0 Neonatal infective mastitis	<5	<0.01
P39.1 Neonatal conjunctivitis and dac..	<5	<0.01
P39.9 Infection specific to the perin..	<5	<0.01
R57.2 Septic shock	<5	<0.01
**Total**	**73 563**	**100.00**

eTable 24. Distribution of chronic infections in primary analysis (Finnish multicohort sample)

ICD-10 code and description	N	%
A63.0 Anogenital (venereal) warts	1061	14.69
B27.0 Gammaherpesviral mononucleosis	547	7.57
K05.3 Chronic periodontitis	469	6.49
B27.9 Infectious mononucleosis, unspe..	432	5.98
B01.9 Varicella without complication	347	4.80

B18.2 Chronic viral hepatitis C	338	4.68
K04.5 Chronic apical periodontitis	303	4.19
A60.0 Herpesviral infection of genita..	290	4.01
K02.1 Caries of dentine	267	3.70
B02.9 Zoster without complication	191	2.64
B86 Scabies	160	2.21
A16.2 Tuberculosis of lung, without m..	151	2.09
B35 Dermatophytosis	133	1.84
K02 Dental caries	126	1.74
B00.1 Herpesviral vesicular dermatitis	121	1.67
A15.0 Tuberculosis of lung, confirmed..	103	1.43
B00.2 Herpesviral gingivostomatitis a..	87	1.20
B08.2 Exanthema subitum [sixth disease]	86	1.19
K04.6 Periapical abscess with sinus	84	1.16
B37 Candidiasis	74	1.02
B00.0 Eczema herpeticum	62	0.86
B02.8 Zoster with other complications	62	0.86
K02.9 Dental caries, unspecified	57	0.79
B97.7 Papillomavirus as the cause of ..	54	0.75
A18.2 Tuberculous peripheral lymphade..	52	0.72
M86.6 Other chronic osteomyelitis	51	0.71
B01.1 Varicella encephalitis	48	0.66
B85 Pediculosis and phthiriasis	45	0.62
B25.8 Other cytomegaloviral diseases	43	0.60
B00.4 Herpesviral encephalitis	39	0.54
B00.5 Herpesviral ocular disease	39	0.54
B01.8 Varicella with other complicati..	39	0.54
B18.1 Chronic viral hepatitis B witho..	38	0.53
B25.9 Cytomegaloviral disease, unspec..	38	0.53
A16.5 Tuberculous pleurisy, without m..	35	0.48
B02.3 Zoster ocular disease	35	0.48
B37.3 Candidiasis of vulva and vagina	32	0.44
J17.3 Pneumonia in parasitic diseases	31	0.43
B37.0 Candidal stomatitis	28	0.39
B37.7 Candidal sepsis	28	0.39
B37.8 Candidiasis of other sites	28	0.39
B35.3 Tinea pedis	26	0.36
A15.1 Tuberculosis of lung, confirmed..	25	0.35
B00.8 Other forms of herpesviral infe..	24	0.33
B01 Varicella [chickenpox]	24	0.33
B23.1 HIV disease resulting in (persi..	24	0.33
B24 Unspecified human immunodeficienc..	23	0.32
A18.0 Tuberculosis of bones and joints	22	0.30
A31.0 Pulmonary mycobacterial infection	21	0.29
B27.1 Cytomegaloviral mononucleosis	21	0.29
B00.3 Herpesviral meningitis	19	0.26
B58.9 Toxoplasmosis, unspecified	19	0.26
M86.8 Other osteomyelitis	19	0.26

A52.1 Symptomatic neurosyphilis	17	0.24
M86.4 Chronic osteomyelitis with drai..	17	0.24
A51.3 Secondary syphilis of skin and ..	16	0.22
A51.0 Primary genital syphilis	15	0.21
B00.9 Herpesviral infection, unspecif..	15	0.21
B35.1 Tinea unguium	15	0.21
B70.0 Diphyllobothriasis	15	0.21
B01.2 Varicella pneumonia	14	0.19
B00.7 Disseminated herpesviral disease	13	0.18
B25.1 Cytomegaloviral hepatitis	13	0.18
B58.0 Toxoplasma oculopathy	13	0.18
B80 Enterobiasis	13	0.18
K02.8 Other dental caries	13	0.18
A15.6 Tuberculous pleurisy, confirmed..	12	0.17
A18.1 Tuberculosis of genitourinary s..	12	0.17
L70.1 Acne conglobata	12	0.17
A16.0 Tuberculosis of lung, bacteriol..	11	0.15
B02.0 Zoster encephalitis	11	0.15
B02.1 Zoster meningitis	10	0.14
B37.4 Candidiasis of other urogenital..	10	0.14
B53.0 Plasmodium ovale malaria	10	0.14
Z21 Asymptomatic human immunodeficien..	10	0.14
A16.9 Respiratory tuberculosis unspec..	9	0.12
B18.9 Chronic viral hepatitis, unspec..	9	0.12
A18.3 Tuberculosis of intestines, per..	8	0.11
A60.9 Anogenital herpesviral infectio..	8	0.11
B02.2 Zoster with other nervous syste..	8	0.11
B36 Other superficial mycoses	8	0.11
B51.9 Plasmodium vivax malaria withou..	8	0.11
A42.9 Actinomycosis, unspecified	7	0.10
B25.0 Cytomegaloviral pneumonitis	7	0.10
B44.1 Other pulmonary aspergillosis	7	0.10
B67 Echinococcosis	7	0.10
B77.9 Ascariasis, unspecified	7	0.10
H19.1 Herpesviral keratitis and kerat..	7	0.10
M86.2 Subacute osteomyelitis	7	0.10
A15.2 Tuberculosis of lung, confirmed..	6	0.08
A18.8 Tuberculosis of other specified..	6	0.08
A31.9 Mycobacterial infection, unspec..	6	0.08
B01.0 Varicella meningitis	6	0.08
B02.7 Disseminated zoster	6	0.08
B35.6 Tinea cruris	6	0.08
B74 Filariasis	6	0.08
A15.4 Tuberculosis of intrathoracic l..	5	0.07
A15.7 Primary respiratory tuberculosi..	5	0.07
A15.9 Respiratory tuberculosis unspec..	5	0.07
A16.1 Tuberculosis of lung, bacteriol..	5	0.07
A18.4 Tuberculosis of skin and subcut..	5	0.07

A30 Leprosy [Hansen disease]	5	0.07
A31 Infection due to other mycobacteria	5	0.07
A42.8 Other forms of actinomycosis	5	0.07
A51.5 Early syphilis, latent	5	0.07
A51.9 Early syphilis, unspecified	5	0.07
B44.0 Invasive pulmonary aspergillosis	5	0.07
B44.9 Aspergillosis, unspecified	5	0.07
K02.0 Caries limited to enamel	5	0.07
O98.0 Tuberculosis complicating pregn..	5	0.07
A15.3 Tuberculosis of lung, confirmed..	<5	<0.07
A15.5 Tuberculosis of larynx, trachea..	<5	<0.07
A15.8 Other respiratory tuberculosis,..	<5	<0.07
A16.3 Tuberculosis of intrathoracic l..	<5	<0.07
A16.4 Tuberculosis of larynx, trachea..	<5	<0.07
A16.7 Primary respiratory tuberculosi..	<5	<0.07
A16.8 Other respiratory tuberculosis,..	<5	<0.07
A17.0 Tuberculous meningitis	<5	<0.07
A17.8 Other tuberculosis of nervous s..	<5	<0.07
A17.9 Tuberculosis of nervous system,..	<5	<0.07
A19.0 Acute miliary tuberculosis of a..	<5	<0.07
A19.2 Acute miliary tuberculosis, uns..	<5	<0.07
A19.9 Miliary tuberculosis, unspecified	<5	<0.07
A31.1 Cutaneous mycobacterial infection	<5	<0.07
A31.8 Other mycobacterial infections	<5	<0.07
A42 Actinomycosis	<5	<0.07
A42.0 Pulmonary actinomycosis	<5	<0.07
A42.1 Abdominal actinomycosis	<5	<0.07
A42.2 Cervicofacial actinomycosis	<5	<0.07
A42.7 Actinomycotic sepsis	<5	<0.07
A44.1 Cutaneous and mucocutaneous bar..	<5	<0.07
A50 Congenital syphilis	<5	<0.07
A50.5 Other late congenital syphilis,..	<5	<0.07
A50.9 Congenital syphilis, unspecified	<5	<0.07
A51.1 Primary anal syphilis	<5	<0.07
A51.2 Primary syphilis of other sites	<5	<0.07
A51.4 Other secondary syphilis	<5	<0.07
A52.0 Cardiovascular syphilis	<5	<0.07
A52.7 Other symptomatic late syphilis	<5	<0.07
A52.8 Late syphilis, latent	<5	<0.07
A53 Other and unspecified syphilis	<5	<0.07
A53.9 Syphilis, unspecified	<5	<0.07
A58 Granuloma inguinale	<5	<0.07
A60.1 Herpesviral infection of perian..	<5	<0.07
A66.1 Multiple papillomata and wet cr..	<5	<0.07
A66.3 Hyperkeratosis of yaws	<5	<0.07
A67 Pinta [carate]	<5	<0.07
A67.1 Intermediate lesions of pinta	<5	<0.07
A81.2 Progressive multifocal leukoenc..	<5	<0.07

B02 Zoster [herpes zoster]	<5	<0.07
B18.0 Chronic viral hepatitis B with ..	<5	<0.07
B18.8 Other chronic viral hepatitis	<5	<0.07
B20.0 HIV disease resulting in mycoba..	<5	<0.07
B20.6 HIV disease resulting in Pneumo..	<5	<0.07
B21.2 HIV disease resulting in other ..	<5	<0.07
B21.9 HIV disease resulting in unspec..	<5	<0.07
B22.0 HIV disease resulting in enceph..	<5	<0.07
B23.0 Acute HIV infection syndrome	<5	<0.07
B23.8 HIV disease resulting in other ..	<5	<0.07
B35.0 Tinea barbae and tinea capitis	<5	<0.07
B35.2 Tinea manuum	<5	<0.07
B35.4 Tinea corporis	<5	<0.07
B35.9 Dermatophytosis, unspecified	<5	<0.07
B36.0 Pityriasis versicolor	<5	<0.07
B36.8 Other specified superficial myc..	<5	<0.07
B36.9 Superficial mycosis, unspecified	<5	<0.07
B37.1 Pulmonary candidiasis	<5	<0.07
B37.2 Candidiasis of skin and nail	<5	<0.07
B37.9 Candidiasis, unspecified	<5	<0.07
B38.1 Chronic pulmonary coccidioidomy..	<5	<0.07
B39.1 Chronic pulmonary histoplasmosi..	<5	<0.07
B39.3 Disseminated histoplasmosis cap..	<5	<0.07
B41.0 Pulmonary paracoccidioidomycosis	<5	<0.07
B43.9 Chromomycosis, unspecified	<5	<0.07
B44.8 Other forms of aspergillosis	<5	<0.07
B47.9 Mycetoma, unspecified	<5	<0.07
B48.1 Rhinosporidiosis	<5	<0.07
B51 Plasmodium vivax malaria	<5	<0.07
B51.0 Plasmodium vivax malaria with r..	<5	<0.07
B55.2 Mucocutaneous leishmaniasis	<5	<0.07
B58.8 Toxoplasmosis with other organ ..	<5	<0.07
B65.0 Schistosomiasis due to Schistos..	<5	<0.07
B65.1 Schistosomiasis due to Schistos..	<5	<0.07
B65.2 Schistosomiasis due to Schistos..	<5	<0.07
B65.9 Schistosomiasis, unspecified	<5	<0.07
B67.3 Echinococcus granulosus infecti..	<5	<0.07
B67.8 Echinococcosis, unspecified, of..	<5	<0.07
B67.9 Echinococcosis, other and unspe..	<5	<0.07
B68.0 Taenia solium taeniasis	<5	<0.07
B68.1 Taenia saginata taeniasis	<5	<0.07
B68.9 Taeniasis, unspecified	<5	<0.07
B69.0 Cysticercosis of central nervou..	<5	<0.07
B71.0 Hymenolepiasis	<5	<0.07
B74.0 Filariasis due to Wuchereria ba..	<5	<0.07
B75 Trichinellosis	<5	<0.07
B76 Hookworm diseases	<5	<0.07
B76.0 Ancylostomiasis	<5	<0.07

B77.8 Ascariasis with other complicat..	<5	<0.07
B78.0 Intestinal strongyloidiasis	<5	<0.07
B78.9 Strongyloidiasis, unspecified	<5	<0.07
B79 Trichuriasis	<5	<0.07
B81.2 Trichostrongyliasis	<5	<0.07
B85.0 Pediculosis due to Pediculus hu..	<5	<0.07
B85.1 Pediculosis due to Pediculus hu..	<5	<0.07
B85.3 Phthiriasis	<5	<0.07
G04.1 Tropical spastic paraplegia	<5	<0.07
H62.2 Otitis externa in mycoses	<5	<0.07
K02.2 Caries of cementum	<5	<0.07
K02.3 Arrested dental caries	<5	<0.07
K23.0 Tuberculous oesophagitis	<5	<0.07
K67.3 Tuberculous peritonitis	<5	<0.07
M01.1 Tuberculous arthritis	<5	<0.07
M49.0 Tuberculosis of spine	<5	<0.07
M86.3 Chronic multifocal osteomyelitis	<5	<0.07
O98.1 Syphilis complicating pregnancy..	<5	<0.07
P37.1 Congenital toxoplasmosis	<5	<0.07
**Total**	**7 225**	**100.00**

eTable 25. Distribution of infections predisposed towards entering the central nervous system in primary analysis (Finnish multicohort sample)

ICD-10 code and description	N	%
B27.0 Gammaherpesviral mononucleosis	545	6.83
B27.9 Infectious mononucleosis, unspe..	425	5.33
J13 Pneumonia due to Streptococcus pn..	402	5.04
B01.9 Varicella without complication	343	4.3
B06.9 Rubella without complication	339	4.25
A69.2 Lyme disease	312	3.91
B18.2 Chronic viral hepatitis C	287	3.6
A60.0 Herpesviral infection of genita..	281	3.52
A87.9 Viral meningitis, unspecified	272	3.41
K12.2 Cellulitis and abscess of mouth	252	3.16
A40.3 Sepsis due to Streptococcus pne..	195	2.45
B02.9 Zoster without complication	195	2.45
B19.9 Unspecified viral hepatitis wit..	177	2.22
A89 Unspecified viral infection of ce..	165	2.07
A16.2 Tuberculosis of lung, without m..	152	1.91
A86 Unspecified viral encephalitis	145	1.82
G00.9 Bacterial meningitis, unspecified	144	1.81
A87.0 Enteroviral meningitis	143	1.79
I33.0 Acute and subacute infective en..	127	1.59
B00.1 Herpesviral vesicular dermatitis	114	1.43
A15.0 Tuberculosis of lung, confirmed..	102	1.28
B05.9 Measles without complication	98	1.23
G03.9 Meningitis, unspecified	98	1.23

B08.2 Exanthema subitum [sixth disease]	85	1.07
B00.2 Herpesviral gingivostomatitis a..	84	1.05
B37 Candidiasis	73	0.92
B26.2 Mumps encephalitis	69	0.87
B00.0 Eczema herpeticum	61	0.76
B02.8 Zoster with other complications	59	0.74
A18.2 Tuberculous peripheral lymphade..	52	0.65
H05.0 Acute inflammation of orbit	50	0.63
B25.8 Other cytomegaloviral diseases	47	0.59
B15.9 Hepatitis A without hepatic coma	45	0.56
G00.8 Other bacterial meningitis	44	0.55
H70.0 Acute mastoiditis	43	0.54
B01.1 Varicella encephalitis	41	0.51
B16.9 Acute hepatitis B without delta..	41	0.51
B01.8 Varicella with other complicati..	40	0.5
G05.1 Encephalitis, myelitis and ence..	38	0.48
B00.5 Herpesviral ocular disease	37	0.46
B25.9 Cytomegaloviral disease, unspec..	36	0.45
A16.5 Tuberculous pleurisy, without m..	35	0.44
B18.1 Chronic viral hepatitis B witho..	34	0.43
A39.0 Meningococcal meningitis	33	0.41
B37.3 Candidiasis of vulva and vagina	31	0.39
G00.0 Haemophilus meningitis	31	0.39
G06.1 Intraspinal abscess and granuloma	31	0.39
B37.8 Candidiasis of other sites	30	0.38
G06.0 Intracranial abscess and granul..	30	0.38
B02.3 Zoster ocular disease	28	0.35
B37.7 Candidal sepsis	28	0.35
B17.1 Acute hepatitis C	27	0.34
B23.1 HIV disease resulting in (persi..	26	0.33
B34.0 Adenovirus infection, unspecifi..	26	0.33
G02.0 Meningitis in viral diseases cl..	26	0.33
A15.1 Tuberculosis of lung, confirmed..	25	0.31
B37.0 Candidal stomatitis	25	0.31
G01 Meningitis in bacterial diseases ..	25	0.31
B01 Varicella [chickenpox]	24	0.3
B24 Unspecified human immunodeficienc..	24	0.3
B26.9 Mumps without complication	24	0.3
B50.9 Plasmodium falciparum malaria, ..	23	0.29
G00.1 Pneumococcal meningitis	23	0.29
A18.0 Tuberculosis of bones and joints	22	0.28
B00.8 Other forms of herpesviral infe..	22	0.28
A88.8 Other specified viral infection..	21	0.26
B27.1 Cytomegaloviral mononucleosis	21	0.26
O98.4 Viral hepatitis complicating pr..	21	0.26
B97.0 Adenovirus as the cause of dise..	20	0.25
B58.9 Toxoplasmosis, unspecified	19	0.24
J14 Pneumonia due to Haemophilus infl..	18	0.23

A51.0 Primary genital syphilis	16	0.2
A52.1 Symptomatic neurosyphilis	16	0.2
A51.3 Secondary syphilis of skin and ..	15	0.19
B70.0 Diphyllobothriasis	15	0.19
A02.2 Localized salmonella infections	14	0.18
A87.8 Other viral meningitis	14	0.18
B00.9 Herpesviral infection, unspecif..	14	0.18
B01.2 Varicella pneumonia	14	0.18
B06 Rubella [German measles]	14	0.18
A06.9 Amoebiasis, unspecified	13	0.16
A90 Dengue fever [classical dengue]	13	0.16
B25.1 Cytomegaloviral hepatitis	13	0.16
B34.1 Enterovirus infection, unspecif..	13	0.16
J20.1 Acute bronchitis due to Haemoph..	13	0.16
A15.6 Tuberculous pleurisy, confirmed..	12	0.15
A18.1 Tuberculosis of genitourinary s..	12	0.15
A39.2 Acute meningococcaemia	12	0.15
B26.0 Mumps orchitis	12	0.15
B54 Unspecified malaria	12	0.15
A16.0 Tuberculosis of lung, bacteriol..	11	0.14
A32.7 Listerial sepsis	11	0.14
B00.4 Herpesviral encephalitis	11	0.14
B00.7 Disseminated herpesviral disease	11	0.14
B02.0 Zoster encephalitis	11	0.14
B58.0 Toxoplasma oculopathy	11	0.14
A08.2 Adenoviral enteritis	10	0.13
A84.8 Other tick-borne viral encephal..	10	0.13
B37.4 Candidiasis of other urogenital..	10	0.13
Z21 Asymptomatic human immunodeficien..	10	0.13
A06.0 Acute amoebic dysentery	9	0.11
A16.9 Respiratory tuberculosis unspec..	9	0.11
A32.8 Other forms of listeriosis	9	0.11
A39.4 Meningococcaemia, unspecified	9	0.11
B05.8 Measles with other complications	9	0.11
G03.8 Meningitis due to other specifi..	9	0.11
M01.2 Arthritis in Lyme disease	9	0.11
A15.2 Tuberculosis of lung, confirmed..	8	0.1
A18.3 Tuberculosis of intestines, per..	8	0.1
A60.9 Anogenital herpesviral infectio..	8	0.1
B02.2 Zoster with other nervous syste..	8	0.1
B26.3 Mumps pancreatitis	8	0.1
B67 Echinococcosis	8	0.1
H62.1 Otitis externa in viral disease..	8	0.1
A42.9 Actinomycosis, unspecified	7	0.09
B02.1 Zoster meningitis	7	0.09
B18.9 Chronic viral hepatitis, unspec..	7	0.09
B19.0 Unspecified viral hepatitis wit..	7	0.09
B25.0 Cytomegaloviral pneumonitis	7	0.09

H19.1 Herpesviral keratitis and kerat..	7	0.09
H19.2 Keratitis and keratoconjunctivi..	7	0.09
J17.2 Pneumonia in mycoses	7	0.09
A18.8 Tuberculosis of other specified..	6	0.08
A84.9 Tick-borne viral encephalitis, ..	6	0.08
B02.7 Disseminated zoster	6	0.08
B08.5 Enteroviral vesicular pharyngitis	6	0.08
B15.0 Hepatitis A with hepatic coma	6	0.08
B26.8 Mumps with other complications	6	0.08
B97.1 Enterovirus as the cause of dis..	6	0.08
G03.0 Nonpyogenic meningitis	6	0.08
A15.4 Tuberculosis of intrathoracic l..	5	0.06
A15.7 Primary respiratory tuberculosi..	5	0.06
A15.9 Respiratory tuberculosis unspec..	5	0.06
A16.1 Tuberculosis of lung, bacteriol..	5	0.06
A18.4 Tuberculosis of skin and subcut..	5	0.06
A42.8 Other forms of actinomycosis	5	0.06
A51.5 Early syphilis, latent	5	0.06
A51.9 Early syphilis, unspecified	5	0.06
B17.8 Other specified acute viral hep..	5	0.06
G00.2 Streptococcal meningitis	5	0.06
G00.3 Staphylococcal meningitis	5	0.06
G05.0 Encephalitis, myelitis and ence..	5	0.06
H32.0 Chorioretinal inflammation in i..	5	0.06
O98.0 Tuberculosis complicating pregn..	5	0.06
A06.2 Amoebic nondysenteric colitis	<5	<0.06
A06.4 Amoebic liver abscess	<5	<0.06
A06.5 Amoebic lung abscess	<5	<0.06
A06.7 Cutaneous amoebiasis	<5	<0.06
A06.8 Amoebic infection of other sites	<5	<0.06
A15.3 Tuberculosis of lung, confirmed..	<5	<0.06
A15.5 Tuberculosis of larynx, trachea..	<5	<0.06
A15.8 Other respiratory tuberculosis,..	<5	<0.06
A16.3 Tuberculosis of intrathoracic l..	<5	<0.06
A16.4 Tuberculosis of larynx, trachea..	<5	<0.06
A16.7 Primary respiratory tuberculosi..	<5	<0.06
A16.8 Other respiratory tuberculosis,..	<5	<0.06
A17.0 Tuberculous meningitis	<5	<0.06
A17.9 Tuberculosis of nervous system,..	<5	<0.06
A19.0 Acute miliary tuberculosis of a..	<5	<0.06
A19.2 Acute miliary tuberculosis, uns..	<5	<0.06
A19.9 Miliary tuberculosis, unspecified	<5	<0.06
A20.9 Plague, unspecified	<5	<0.06
A22.0 Cutaneous anthrax	<5	<0.06
A22.2 Gastrointestinal anthrax	<5	<0.06
A23.9 Brucellosis, unspecified	<5	<0.06
A24.1 Acute and fulminating melioidosis	<5	<0.06
A25.9 Rat-bite fever, unspecified	<5	<0.06

A27 Leptospirosis	<5	<0.06
A27.0 Leptospirosis icterohaemorrhagica	<5	<0.06
A27.8 Other forms of leptospirosis	<5	<0.06
A28.0 Pasteurellosis	<5	<0.06
A32.1 Listerial meningitis and mening..	<5	<0.06
A32.9 Listeriosis, unspecified	<5	<0.06
A39 Meningococcal infection	<5	<0.06
A39.1 Waterhouse-Friderichsen syndrome	<5	<0.06
A39.5 Meningococcal heart disease	<5	<0.06
A39.8 Other meningococcal infections	<5	<0.06
A39.9 Meningococcal infection, unspec..	<5	<0.06
A41.3 Sepsis due to Haemophilus influ..	<5	<0.06
A42 Actinomycosis	<5	<0.06
A42.0 Pulmonary actinomycosis	<5	<0.06
A42.1 Abdominal actinomycosis	<5	<0.06
A42.2 Cervicofacial actinomycosis	<5	<0.06
A42.7 Actinomycotic sepsis	<5	<0.06
A43.0 Pulmonary nocardiosis	<5	<0.06
A44.1 Cutaneous and mucocutaneous bar..	<5	<0.06
A44.8 Other forms of bartonellosis	<5	<0.06
A44.9 Bartonellosis, unspecified	<5	<0.06
A49.2 Haemophilus influenzae infectio..	<5	<0.06
A50 Congenital syphilis	<5	<0.06
A50.5 Other late congenital syphilis,..	<5	<0.06
A50.9 Congenital syphilis, unspecified	<5	<0.06
A51.1 Primary anal syphilis	<5	<0.06
A51.2 Primary syphilis of other sites	<5	<0.06
A51.4 Other secondary syphilis	<5	<0.06
A52.0 Cardiovascular syphilis	<5	<0.06
A52.7 Other symptomatic late syphilis	<5	<0.06
A52.8 Late syphilis, latent	<5	<0.06
A53 Other and unspecified syphilis	<5	<0.06
A53.9 Syphilis, unspecified	<5	<0.06
A60.1 Herpesviral infection of perian..	<5	<0.06
A68 Relapsing fevers	<5	<0.06
A68.9 Relapsing fever, unspecified	<5	<0.06
A69.9 Spirochaetal infection, unspeci..	<5	<0.06
A70 Chlamydia psittaci infection	<5	<0.06
A75.0 Epidemic louse-borne typhus fev..	<5	<0.06
A75.9 Typhus fever, unspecified	<5	<0.06
A77.1 Spotted fever due to Rickettsia..	<5	<0.06
A77.8 Other spotted fevers	<5	<0.06
A78 Q fever	<5	<0.06
A79 Other rickettsioses	<5	<0.06
A79.9 Rickettsiosis, unspecified	<5	<0.06
A80 Acute poliomyelitis	<5	<0.06
A80.1 Acute paralytic poliomyelitis, ..	<5	<0.06
A80.3 Acute paralytic poliomyelitis, ..	<5	<0.06

A81.2 Progressive multifocal leukoenc..	<5	<0.06
A83.0 Japanese encephalitis	<5	<0.06
A83.2 Eastern equine encephalitis	<5	<0.06
A83.6 Rocio virus disease	<5	<0.06
A83.8 Other mosquito-borne viral ence..	<5	<0.06
A84 Tick-borne viral encephalitis	<5	<0.06
A84.1 Central European tick-borne enc..	<5	<0.06
A85.0 Enteroviral encephalitis	<5	<0.06
A85.1 Adenoviral encephalitis	<5	<0.06
A85.2 Arthropod-borne viral encephali..	<5	<0.06
A85.8 Other specified viral encephali..	<5	<0.06
A87.1 Adenoviral meningitis	<5	<0.06
A87.2 Lymphocytic choriomeningitis	<5	<0.06
A88.1 Epidemic vertigo	<5	<0.06
A96.0 Junin haemorrhagic fever	<5	<0.06
A98.1 Omsk haemorrhagic fever	<5	<0.06
A98.3 Marburg virus disease	<5	<0.06
A98.4 Ebola virus disease	<5	<0.06
B00.3 Herpesviral meningitis	<5	<0.06
B01.0 Varicella meningitis	<5	<0.06
B02 Zoster [herpes zoster]	<5	<0.06
B05 Measles	<5	<0.06
B05.3 Measles complicated by otitis m..	<5	<0.06
B06.8 Rubella with other complications	<5	<0.06
B08.4 Enteroviral vesicular stomatiti..	<5	<0.06
B16.0 Acute hepatitis B with delta-ag..	<5	<0.06
B16.1 Acute hepatitis B with delta-ag..	<5	<0.06
B17.0 Acute delta-(super)infection of..	<5	<0.06
B17.2 Acute hepatitis E	<5	<0.06
B18.0 Chronic viral hepatitis B with ..	<5	<0.06
B18.8 Other chronic viral hepatitis	<5	<0.06
B20.0 HIV disease resulting in mycoba..	<5	<0.06
B20.6 HIV disease resulting in Pneumo..	<5	<0.06
B21.2 HIV disease resulting in other ..	<5	<0.06
B21.9 HIV disease resulting in unspec..	<5	<0.06
B22.0 HIV disease resulting in enceph..	<5	<0.06
B23.8 HIV disease resulting in other ..	<5	<0.06
B26 Mumps	<5	<0.06
B26.1 Mumps meningitis	<5	<0.06
B37.1 Pulmonary candidiasis	<5	<0.06
B37.2 Candidiasis of skin and nail	<5	<0.06
B37.9 Candidiasis, unspecified	<5	<0.06
B38 Coccidioidomycosis	<5	<0.06
B38.0 Acute pulmonary coccidioidomyco..	<5	<0.06
B38.1 Chronic pulmonary coccidioidomy..	<5	<0.06
B39.1 Chronic pulmonary histoplasmosi..	<5	<0.06
B39.3 Disseminated histoplasmosis cap..	<5	<0.06
B39.4 Histoplasmosis capsulati, unspe..	<5	<0.06

B40 Blastomycosis	<5	<0.06
B43.9 Chromomycosis, unspecified	<5	<0.06
B45.0 Pulmonary cryptococcosis	<5	<0.06
B46.0 Pulmonary mucormycosis	<5	<0.06
B46.4 Disseminated mucormycosis	<5	<0.06
B49 Unspecified mycosis	<5	<0.06
B50.0 Plasmodium falciparum malaria w..	<5	<0.06
B50.8 Other severe and complicated Pl..	<5	<0.06
B58.8 Toxoplasmosis with other organ ..	<5	<0.06
B65.2 Schistosomiasis due to Schistos..	<5	<0.06
B65.9 Schistosomiasis, unspecified	<5	<0.06
B67.3 Echinococcus granulosus infecti..	<5	<0.06
B67.8 Echinococcosis, unspecified, of..	<5	<0.06
B67.9 Echinococcosis, other and unspe..	<5	<0.06
B68.0 Taenia solium taeniasis	<5	<0.06
B68.9 Taeniasis, unspecified	<5	<0.06
B69.0 Cysticercosis of central nervou..	<5	<0.06
B78.0 Intestinal strongyloidiasis	<5	<0.06
B81.4 Mixed intestinal helminthiases	<5	<0.06
B83.0 Visceral larva migrans	<5	<0.06
B87.1 Wound myiasis	<5	<0.06
B87.9 Myiasis, unspecified	<5	<0.06
B96.3 Haemophilus influenzae [H. infl..	<5	<0.06
G03.1 Chronic meningitis	<5	<0.06
G04.1 Tropical spastic paraplegia	<5	<0.06
G04.2 Bacterial meningoencephalitis a..	<5	<0.06
G05.2 Encephalitis, myelitis and ence..	<5	<0.06
G06 Intracranial and intraspinal absc..	<5	<0.06
G06.2 Extradural and subdural abscess..	<5	<0.06
G07 Intracranial and intraspinal absc..	<5	<0.06
H19.0 Scleritis and episcleritis in d..	<5	<0.06
H22.0 Iridocyclitis in infectious and..	<5	<0.06
H60.2 Malignant otitis externa	<5	<0.06
H62.2 Otitis externa in mycoses	<5	<0.06
I68.1 Cerebral arteritis in infectiou..	<5	<0.06
K23.0 Tuberculous oesophagitis	<5	<0.06
K67.3 Tuberculous peritonitis	<5	<0.06
M00.1 Pneumococcal arthritis and poly..	<5	<0.06
M01.0 Meningococcal arthritis	<5	<0.06
M01.1 Tuberculous arthritis	<5	<0.06
M49.0 Tuberculosis of spine	<5	<0.06
M49.1 Brucella spondylitis	<5	<0.06
O98.1 Syphilis complicating pregnancy..	<5	<0.06
P37.1 Congenital toxoplasmosis	<5	<0.06
**Total**	**7 974**	**100.00**

eTable 26. Distribution of infections not predisposed towards entering the central nervous system in primary analysis (Finnish multicohort sample)

ICD-10 code and description	N	%
K35 Acute appendicitis	12 102	17.04
J18.9 Pneumonia, unspecified	6364	8.96
A09 Other gastroenteritis and colitis..	5789	8.15
N10 Acute tubulo-interstitial nephritis	3719	5.24
A46 Erysipelas	3215	4.53
J03.9 Acute tonsillitis, unspecified	3191	4.49
J36 Peritonsillar abscess	3106	4.37
J15.9 Bacterial pneumonia, unspecified	2358	3.32
N70.0 Acute salpingitis and oophoritis	1827	2.57
O85 Puerperal sepsis	1760	2.48
J20.9 Acute bronchitis, unspecified	1209	1.7
N39.0 Urinary tract infection, site n..	1104	1.55
J01.0 Acute maxillary sinusitis	1082	1.52
J03.0 Streptococcal tonsillitis	970	1.37
A63.0 Anogenital (venereal) warts	864	1.22
A08.4 Viral intestinal infection, uns..	836	1.18
J22 Unspecified acute lower respirato..	791	1.11
N71.0 Acute inflammatory disease of u..	740	1.04
J02.9 Acute pharyngitis, unspecified	711	1
J04.0 Acute laryngitis	690	0.97
A98.5 Haemorrhagic fever with renal s..	591	0.83
A08.5 Other specified intestinal infe..	498	0.7
K57.2 Diverticular disease of large i..	469	0.66
N30.0 Acute cystitis	459	0.65
J18.0 Bronchopneumonia, unspecified	457	0.64
A41.5 Sepsis due to other Gram-negati..	450	0.63
O08.0 Genital tract and pelvic infect..	376	0.53
O03.5 Spontaneous abortion: Complete ..	369	0.52
K61.0 Anal abscess	340	0.48
K05.3 Chronic periodontitis	336	0.47
A04.7 Enterocolitis due to Clostridiu..	329	0.46
H66.0 Acute suppurative otitis media	307	0.43
A41.9 Sepsis, unspecified	306	0.43
N75.1 Abscess of Bartholin gland	301	0.42
J11.1 Influenza with other respirator..	289	0.41
K04.7 Periapical abscess without sinus	275	0.39
J11.8 Influenza with other manifestat..	262	0.37
N61 Inflammatory disorders of breast	260	0.37
L04 Acute lymphadenitis	233	0.33
M00.9 Pyogenic arthritis, unspecified	217	0.31
J10.1 Influenza with other respirator..	216	0.3
N45.9 Orchitis, epididymitis and epid..	210	0.3
K04.5 Chronic apical periodontitis	209	0.29
L03.0 Cellulitis of finger and toe	201	0.28
A04.5 Campylobacter enteritis	196	0.28

B99 Other and unspecified infectious ..	196	0.28
A41.0 Sepsis due to Staphylococcus au..	188	0.26
O23.0 Infections of kidney in pregnancy	178	0.25
O23.5 Infections of the genital tract..	178	0.25
K61 Abscess of anal and rectal regions	177	0.25
O03.0 Spontaneous abortion: Incomplet..	174	0.24
J05.1 Acute epiglottitis	173	0.24
K02.1 Caries of dentine	171	0.24
L08.9 Local infection of skin and sub..	171	0.24
L05.0 Pilonidal cyst with abscess	169	0.24
O86.0 Infection of obstetric surgical..	166	0.23
L02 Cutaneous abscess, furuncle and c..	165	0.23
O23.9 Other and unspecified genitouri..	161	0.23
A02.0 Salmonella enteritis	158	0.22
A41.8 Other specified sepsis	157	0.22
J15.8 Other bacterial pneumonia	152	0.21
L02.4 Cutaneous abscess, furuncle and..	151	0.21
J12 Viral pneumonia, not elsewhere cl..	148	0.21
B07 Viral warts	138	0.19
O86.1 Other infection of genital trac..	134	0.19
B86 Scabies	129	0.18
O86.4 Pyrexia of unknown origin follo..	124	0.17
L03.1 Cellulitis of other parts of limb	123	0.17
J18.1 Lobar pneumonia, unspecified	115	0.16
K05.2 Acute periodontitis	115	0.16
A02 Other salmonella infections	110	0.15
K02 Dental caries	108	0.15
L04.0 Acute lymphadenitis of face, he..	105	0.15
B35 Dermatophytosis	103	0.15
A56.1 Chlamydial infection of pelvipe..	100	0.14
J15.7 Pneumonia due to Mycoplasma pne..	94	0.13
L02.2 Cutaneous abscess, furuncle and..	93	0.13
O23.4 Unspecified infection of urinar..	93	0.13
O04.0 Medical abortion: Incomplete, c..	92	0.13
J86.9 Pyothorax without fistula	89	0.13
A04.9 Bacterial intestinal infection,..	87	0.12
L03.9 Cellulitis, unspecified	84	0.12
O23.1 Infections of bladder in pregna..	84	0.12
H00.1 Chalazion	82	0.12
H10.0 Mucopurulent conjunctivitis	82	0.12
L02.3 Cutaneous abscess, furuncle and..	81	0.11
A95 Yellow fever	79	0.11
M71.1 Other infective bursitis	78	0.11
J16.8 Pneumonia due to other specifie..	77	0.11
O86.2 Urinary tract infection followi..	77	0.11
A08.3 Other viral enteritis	75	0.11
H60.3 Other infective otitis externa	75	0.11
N41.0 Acute prostatitis	75	0.11

A54.9 Gonococcal infection, unspecified	74	0.1
L08.8 Other specified local infection..	73	0.1
M86.9 Osteomyelitis, unspecified	73	0.1
J09 Influenza due to certain identifi..	68	0.1
N76.4 Abscess of vulva	68	0.1
J39.0 Retropharyngeal and parapharyng..	67	0.09
K57.0 Diverticular disease of small i..	67	0.09
O04.5 Medical abortion: Complete or u..	67	0.09
M86.1 Other acute osteomyelitis	66	0.09
J10.0 Influenza with pneumonia, other..	64	0.09
K04.6 Periapical abscess with sinus	64	0.09
O86.8 Other specified puerperal infec..	63	0.09
O75.3 Other infection during labour	62	0.09
O98.8 Other maternal infectious and p..	61	0.09
A04.8 Other specified bacterial intes..	60	0.08
A54.2 Gonococcal pelviperitonitis and..	58	0.08
H62.0 Otitis externa in bacterial dis..	57	0.08
N77.1 Vaginitis, vulvitis and vulvova..	57	0.08
B09 Unspecified viral infection chara..	56	0.08
A74.9 Chlamydial infection, unspecified	55	0.08
L02.1 Cutaneous abscess, furuncle and..	54	0.08
L02.9 Cutaneous abscess, furuncle and..	54	0.08
A40.8 Other streptococcal sepsis	52	0.07
N73.3 Female acute pelvic peritonitis	52	0.07
O91.1 Abscess of breast associated wi..	52	0.07
L03.2 Cellulitis of face	51	0.07
A38 Scarlet fever	49	0.07
L03.8 Cellulitis of other sites	48	0.07
H00.0 Hordeolum and other deep inflam..	47	0.07
A40.9 Streptococcal sepsis, unspecified	45	0.06
A40.0 Sepsis due to streptococcus, gr..	43	0.06
J20.0 Acute bronchitis due to Mycopla..	43	0.06
A56.0 Chlamydial infection of lower g..	42	0.06
B08.1 Molluscum contagiosum	42	0.06
J11.0 Influenza with pneumonia, virus..	42	0.06
K61.1 Rectal abscess	42	0.06
O98.3 Other infections with a predomi..	42	0.06
I40.0 Infective myocarditis	41	0.06
J02.0 Streptococcal pharyngitis	40	0.06
L02.0 Cutaneous abscess, furuncle and..	40	0.06
A41.1 Sepsis due to other specified s..	39	0.05
L08.0 Pyoderma	39	0.05
A08.0 Rotaviral enteritis	38	0.05
J18.8 Other pneumonia, organism unspe..	38	0.05
J12.9 Viral pneumonia, unspecified	37	0.05
K02.9 Dental caries, unspecified	37	0.05
B85 Pediculosis and phthiriasis	36	0.05
M71.0 Abscess of bursa	36	0.05

B97.7 Papillomavirus as the cause of ..	35	0.05
J04.2 Acute laryngotracheitis	35	0.05
H60.1 Cellulitis of external ear	34	0.05
L02.8 Cutaneous abscess, furuncle and..	34	0.05
A04.6 Enteritis due to Yersinia enter..	32	0.05
A02.1 Salmonella sepsis	31	0.04
M86.6 Other chronic osteomyelitis	31	0.04
A59.0 Urogenital trichomoniasis	30	0.04
J85.2 Abscess of lung without pneumonia	30	0.04
L03.3 Cellulitis of trunk	29	0.04
N73.0 Acute parametritis and pelvic c..	29	0.04
A37 Whooping cough	27	0.04
H60.0 Abscess of external ear	27	0.04
A05.9 Bacterial foodborne intoxicatio..	26	0.04
J10.8 Influenza with other manifestat..	26	0.04
J86.0 Pyothorax with fistula	26	0.04
M00.0 Staphylococcal arthritis and po..	26	0.04
A41.2 Sepsis due to unspecified staph..	25	0.04
K63.0 Abscess of intestine	25	0.04
O98.9 Unspecified maternal infectious..	25	0.04
K75.0 Abscess of liver	24	0.03
H44.0 Purulent endophthalmitis	23	0.03
I30.1 Infective pericarditis	23	0.03
J21.9 Acute bronchiolitis, unspecified	23	0.03
O86.3 Other genitourinary tract infec..	22	0.03
A21.0 Ulceroglandular tularaemia	21	0.03
A21.9 Tularaemia, unspecified	21	0.03
K11.3 Abscess of salivary gland	21	0.03
K61.2 Anorectal abscess	21	0.03
M65.0 Abscess of tendon sheath	21	0.03
N76.0 Acute vaginitis	21	0.03
A54.1 Gonococcal infection of lower g..	20	0.03
J17.3 Pneumonia in parasitic diseases	20	0.03
K04.4 Acute apical periodontitis of p..	19	0.03
J04.1 Acute tracheitis	18	0.03
M46.3 Infection of intervertebral dis..	18	0.03
N74.4 Female chlamydial pelvic inflam..	18	0.03
B59 Pneumocystosis	17	0.02
J86 Pyothorax	17	0.02
N15.1 Renal and perinephric abscess	17	0.02
A07 Other protozoal intestinal diseases	16	0.02
A41.4 Sepsis due to anaerobes	16	0.02
L01.0 Impetigo [any organism] [any si..	16	0.02
A02.9 Salmonella infection, unspecified	15	0.02
A40.1 Sepsis due to streptococcus, gr..	15	0.02
K57.4 Diverticular disease of both sm..	15	0.02
N45.0 Orchitis, epididymitis and epid..	15	0.02
O91.0 Infection of nipple associated ..	15	0.02

J03.8 Acute tonsillitis due to other ..	14	0.02
M46.2 Osteomyelitis of vertebra	14	0.02
M60.0 Infective myositis	14	0.02
A01.4 Paratyphoid fever, unspecified	13	0.02
A03.3 Shigellosis due to Shigella son..	13	0.02
B35.1 Tinea unguium	13	0.02
J85.1 Abscess of lung with pneumonia	13	0.02
N34.0 Urethral abscess	13	0.02
A31.0 Pulmonary mycobacterial infection	12	0.02
B30 Viral conjunctivitis	12	0.02
L70.1 Acne conglobata	12	0.02
O06.5 Unspecified abortion: Complete ..	12	0.02
J20.5 Acute bronchitis due to respira..	11	0.02
K61.3 Ischiorectal abscess	11	0.02
L01 Impetigo	11	0.02
L04.9 Acute lymphadenitis, unspecified	11	0.02
A01.2 Paratyphoid fever B	10	0.01
A03.1 Shigellosis due to Shigella fle..	10	0.01
A64 Unspecified sexually transmitted ..	10	0.01
B33.2 Viral carditis	10	0.01
J16.0 Chlamydial pneumonia	10	0.01
L01.1 Impetiginization of other derma..	10	0.01
M00.2 Other streptococcal arthritis a..	10	0.01
N08.0 Glomerular disorders in infecti..	10	0.01
N13.6 Pyonephrosis	10	0.01
A63.8 Other specified predominantly s..	9	0.01
B08.8 Other specified viral infection..	9	0.01
B53.0 Plasmodium ovale malaria	9	0.01
K02.8 Other dental caries	9	0.01
K04.0 Pulpitis	9	0.01
K77.0 Liver disorders in infectious a..	9	0.01
A01.0 Typhoid fever	8	0.01
A02.8 Other specified salmonella infe..	8	0.01
A04.3 Enterohaemorrhagic Escherichia ..	8	0.01
A54.0 Gonococcal infection of lower g..	8	0.01
B80 Enterobiasis	8	0.01
J20.8 Acute bronchitis due to other s..	8	0.01
J39.1 Other abscess of pharynx	8	0.01
N77.0 Ulceration of vulva in infectio..	8	0.01
O07.0 Failed medical abortion, compli..	8	0.01
O07.5 Other and unspecified failed at..	8	0.01
A74.8 Other chlamydial diseases	7	0.01
B36 Other superficial mycoses	7	0.01
B51.9 Plasmodium vivax malaria withou..	7	0.01
J21.0 Acute bronchiolitis due to resp..	7	0.01
K05.0 Acute gingivitis	7	0.01
O05.0 Other abortion: Incomplete, com..	7	0.01
O05.5 Other abortion: Complete or uns..	7	0.01

A08.1 Acute gastroenteropathy due to ..	6	0.01
A31 Infection due to other mycobacteria	6	0.01
A48.0 Gas gangrene	6	0.01
B08.3 Erythema infectiosum [fifth dis..	6	0.01
B35.3 Tinea pedis	6	0.01
B77.9 Ascariasis, unspecified	6	0.01
B95.6 Staphylococcus aureus as the ca..	6	0.01
B95.8 Unspecified staphylococcus as t..	6	0.01
H10.5 Blepharoconjunctivitis	6	0.01
J15.2 Pneumonia due to staphylococcus	6	0.01
J85.3 Abscess of mediastinum	6	0.01
M65.1 Other infective (teno)synovitis	6	0.01
O06.0 Unspecified abortion: Incomplet..	6	0.01
A03.9 Shigellosis, unspecified	5	0.01
A07.1 Giardiasis [lambliasis]	5	0.01
A31.9 Mycobacterial infection, unspec..	5	0.01
B74 Filariasis	5	0.01
I41.1 Myocarditis in viral diseases c..	5	0.01
J12.1 Respiratory syncytial virus pne..	5	0.01
J15.3 Pneumonia due to streptococcus,..	5	0.01
K57.8 Diverticular disease of intesti..	5	0.01
L04.2 Acute lymphadenitis of upper limb	5	0.01
L04.8 Acute lymphadenitis of other si..	5	0.01
M72.6 Necrotizing fasciitis	5	0.01
M86.0 Acute haematogenous osteomyelitis	5	0.01
M86.2 Subacute osteomyelitis	5	0.01
M86.8 Other osteomyelitis	5	0.01
A00.9 Cholera, unspecified	<5	<0.01
A01.1 Paratyphoid fever A	<5	<0.01
A01.3 Paratyphoid fever C	<5	<0.01
A03 Shigellosis	<5	<0.01
A03.0 Shigellosis due to Shigella dys..	<5	<0.01
A03.2 Shigellosis due to Shigella boy..	<5	<0.01
A04.0 Enteropathogenic Escherichia co..	<5	<0.01
A04.2 Enteroinvasive Escherichia coli..	<5	<0.01
A04.4 Other intestinal Escherichia co..	<5	<0.01
A05.0 Foodborne staphylococcal intoxi..	<5	<0.01
A05.1 Botulism	<5	<0.01
A05.2 Foodborne Clostridium perfringe..	<5	<0.01
A05.8 Other specified bacterial foodb..	<5	<0.01
A07.0 Balantidiasis	<5	<0.01
A07.2 Cryptosporidiosis	<5	<0.01
A07.3 Isosporiasis	<5	<0.01
A07.8 Other specified protozoal intes..	<5	<0.01
A07.9 Protozoal intestinal disease, u..	<5	<0.01
A21.1 Oculoglandular tularaemia	<5	<0.01
A21.2 Pulmonary tularaemia	<5	<0.01
A21.7 Generalized tularaemia	<5	<0.01

A21.8 Other forms of tularaemia	<5	<0.01
A24.0 Glanders	<5	<0.01
A26.0 Cutaneous erysipeloid	<5	<0.01
A26.9 Erysipeloid, unspecified	<5	<0.01
A28.2 Extraintestinal yersiniosis	<5	<0.01
A30 Leprosy [Hansen disease]	<5	<0.01
A31.1 Cutaneous mycobacterial infection	<5	<0.01
A31.8 Other mycobacterial infections	<5	<0.01
A35 Other tetanus	<5	<0.01
A36 Diphtheria	<5	<0.01
A37.0 Whooping cough due to Bordetell..	<5	<0.01
A37.9 Whooping cough, unspecified	<5	<0.01
A40 Streptococcal sepsis	<5	<0.01
A40.2 Sepsis due to streptococcus, gr..	<5	<0.01
A41 Other sepsis	<5	<0.01
A48.3 Toxic shock syndrome	<5	<0.01
A49 Bacterial infection of unspecifie..	<5	<0.01
A49.3 Mycoplasma infection, unspecifi..	<5	<0.01
A54 Gonococcal infection	<5	<0.01
A54.3 Gonococcal infection of eye	<5	<0.01
A54.4 Gonococcal infection of musculo..	<5	<0.01
A54.8 Other gonococcal infections	<5	<0.01
A55 Chlamydial lymphogranuloma (vener..	<5	<0.01
A56.2 Chlamydial infection of genitou..	<5	<0.01
A56.3 Chlamydial infection of anus an..	<5	<0.01
A56.8 Sexually transmitted chlamydial..	<5	<0.01
A58 Granuloma inguinale	<5	<0.01
A66.1 Multiple papillomata and wet cr..	<5	<0.01
A66.3 Hyperkeratosis of yaws	<5	<0.01
A67 Pinta [carate]	<5	<0.01
A67.1 Intermediate lesions of pinta	<5	<0.01
A69.0 Necrotizing ulcerative stomatitis	<5	<0.01
A69.1 Other Vincent infections	<5	<0.01
A71 Trachoma	<5	<0.01
A98.0 Crimean-Congo haemorrhagic fever	<5	<0.01
B08.0 Other orthopoxvirus infections	<5	<0.01
B30.0 Keratoconjunctivitis due to ade..	<5	<0.01
B30.8 Other viral conjunctivitis	<5	<0.01
B33.0 Epidemic myalgia	<5	<0.01
B35.0 Tinea barbae and tinea capitis	<5	<0.01
B35.2 Tinea manuum	<5	<0.01
B35.4 Tinea corporis	<5	<0.01
B35.6 Tinea cruris	<5	<0.01
B35.9 Dermatophytosis, unspecified	<5	<0.01
B36.0 Pityriasis versicolor	<5	<0.01
B36.9 Superficial mycosis, unspecified	<5	<0.01
B41.0 Pulmonary paracoccidioidomycosis	<5	<0.01
B44.0 Invasive pulmonary aspergillosis	<5	<0.01

B44.1 Other pulmonary aspergillosis	<5	<0.01
B44.8 Other forms of aspergillosis	<5	<0.01
B44.9 Aspergillosis, unspecified	<5	<0.01
B47.0 Eumycetoma	<5	<0.01
B47.9 Mycetoma, unspecified	<5	<0.01
B48.1 Rhinosporidiosis	<5	<0.01
B48.7 Opportunistic mycoses	<5	<0.01
B51 Plasmodium vivax malaria	<5	<0.01
B51.0 Plasmodium vivax malaria with r..	<5	<0.01
B52.0 Plasmodium malariae malaria wit..	<5	<0.01
B52.9 Plasmodium malariae malaria wit..	<5	<0.01
B53.8 Other parasitologically confirm..	<5	<0.01
B55.1 Cutaneous leishmaniasis	<5	<0.01
B55.2 Mucocutaneous leishmaniasis	<5	<0.01
B60.8 Other specified protozoal disea..	<5	<0.01
B65.0 Schistosomiasis due to Schistos..	<5	<0.01
B65.1 Schistosomiasis due to Schistos..	<5	<0.01
B68.1 Taenia saginata taeniasis	<5	<0.01
B71.0 Hymenolepiasis	<5	<0.01
B75 Trichinellosis	<5	<0.01
B76 Hookworm diseases	<5	<0.01
B79 Trichuriasis	<5	<0.01
B81.0 Anisakiasis	<5	<0.01
B82.9 Intestinal parasitism, unspecif..	<5	<0.01
B83.9 Helminthiasis, unspecified	<5	<0.01
B85.1 Pediculosis due to Pediculus hu..	<5	<0.01
B95.0 Streptococcus, group A, as the ..	<5	<0.01
B95.1 Streptococcus, group B, as the ..	<5	<0.01
B95.3 Streptococcus pneumoniae as the..	<5	<0.01
B95.4 Other streptococcus as the caus..	<5	<0.01
B95.5 Unspecified streptococcus as th..	<5	<0.01
B95.7 Other staphylococcus as the cau..	<5	<0.01
B96.1 Klebsiella pneumoniae [K. pneum..	<5	<0.01
B96.2 Escherichia coli [E. coli] as t..	<5	<0.01
B96.5 Pseudomonas (aeruginosa) as the..	<5	<0.01
D73.3 Abscess of spleen	<5	<0.01
H01.0 Blepharitis	<5	<0.01
I32.0 Pericarditis in bacterial disea..	<5	<0.01
J02.8 Acute pharyngitis due to other ..	<5	<0.01
J03 Acute tonsillitis	<5	<0.01
J10 Influenza due to other identified..	<5	<0.01
J11 Influenza, virus not identified	<5	<0.01
J12.0 Adenoviral pneumonia	<5	<0.01
J12.2 Parainfluenza virus pneumonia	<5	<0.01
J12.8 Other viral pneumonia	<5	<0.01
J15 Bacterial pneumonia, not elsewher..	<5	<0.01
J15.0 Pneumonia due to Klebsiella pne..	<5	<0.01
J15.1 Pneumonia due to Pseudomonas	<5	<0.01

J15.4 Pneumonia due to other streptoc..	<5	<0.01
J15.5 Pneumonia due to Escherichia coli	<5	<0.01
J15.6 Pneumonia due to other aerobic ..	<5	<0.01
J17.0 Pneumonia in bacterial diseases..	<5	<0.01
J17.1 Pneumonia in viral diseases cla..	<5	<0.01
J18.2 Hypostatic pneumonia, unspecified	<5	<0.01
J20 Acute bronchitis	<5	<0.01
J20.2 Acute bronchitis due to strepto..	<5	<0.01
J20.4 Acute bronchitis due to parainf..	<5	<0.01
J20.6 Acute bronchitis due to rhinovi..	<5	<0.01
J21.8 Acute bronchiolitis due to othe..	<5	<0.01
K02.0 Caries limited to enamel	<5	<0.01
K02.2 Caries of cementum	<5	<0.01
K02.3 Arrested dental caries	<5	<0.01
K61.4 Intrasphincteric abscess	<5	<0.01
K67.8 Other disorders of peritoneum i..	<5	<0.01
K75.1 Phlebitis of portal vein	<5	<0.01
L00 Staphylococcal scalded skin syndr..	<5	<0.01
L04.1 Acute lymphadenitis of trunk	<5	<0.01
L04.3 Acute lymphadenitis of lower limb	<5	<0.01
L08.1 Erythrasma	<5	<0.01
M00.8 Arthritis and polyarthritis due..	<5	<0.01
M01.3 Arthritis in other bacterial di..	<5	<0.01
M46.5 Other infective spondylopathies	<5	<0.01
M49.3 Spondylopathy in other infectio..	<5	<0.01
M63.0 Myositis in bacterial diseases ..	<5	<0.01
M86.3 Chronic multifocal osteomyelitis	<5	<0.01
M86.4 Chronic osteomyelitis with drai..	<5	<0.01
N16.0 Renal tubulo-interstitial disor..	<5	<0.01
O23.2 Infections of urethra in pregna..	<5	<0.01
O23.3 Infections of other parts of ur..	<5	<0.01
O98.6 Protozoal diseases complicating..	<5	<0.01
P36.9 Bacterial sepsis of newborn, un..	<5	<0.01
P37.9 Congenital infectious and paras..	<5	<0.01
P39.0 Neonatal infective mastitis	<5	<0.01
P39.1 Neonatal conjunctivitis and dac..	<5	<0.01
P39.9 Infection specific to the perin..	<5	<0.01
R57.2 Septic shock	<5	<0.01
**Total**	**71 030**	**100.00**

eMethods 3. Stata code for data analysis

```
version 16

**** PRIMARY ANALYSIS (FINNISH MULTICOHORT SAMPLE) ********



capture log close
log using "Jakauma-datan luominen.log", append

*Create the indicator variables for start of follow up for each infectious disease

use "Pooled infections_trimmattu", clear

*Random order
*seed from random.org from range 1 - 1 000 000 000
set rng mt64s
set rngstream 1
set seed 141476928

gen double shuffle1 = runiform()
gen double shuffle2 = runiform()

sort shuffle1 shuffle2

*New id that is in the random order
gen shuffle_id = _n

*Difference between entry and time of infection
gen entry_to_inf = (ensianyinfpvm-entrypvm)/365.25
replace entry_to_inf = 0 if entry_to_inf < 0
bysort cohort supu: sum entry_to_inf if entry_to_inf>0
*hist entry_to_inf
sum entry_to_inf
capture drop entry_to_inf_cut
egen entry_to_inf_cut = cut(entry_to_inf), at(0 0.0009765625 5(5)20 100)
tab entry_to_inf_cut
by cohort supu: tab entry_to_inf_cut
capture drop age_cut
egen age_cut = cut(age), at(18 30(10)60 100)
tab age_cut
bysort cohort supu age_cut: tab entry_to_inf_cut

levelsof cohort, local(cohorts)
levelsof age_cut, local(age_groups)
levelsof supu, local(sexes)
levelsof entry_to_inf_cut, local(entry_lags)
local i 0
foreach cohort of local cohorts {
			foreach age of local age_groups {
						foreach sex of local sexes {
									local i 0
									foreach lag of local entry_lags {
												local ++i
```

```
					quietly: sum entry_to_inf if cohort==`cohort' & age_cut==`age'
& supu==`sex' & entry_to_inf_cut==`lag'

					if `i'==1 & `r(N)' == 0 {
					matrix mean_lag_`cohort'_`age'_`sex' = 0
					}
					else if `i'==1 {
					matrix mean_lag_`cohort'_`age'_`sex' = `r(mean)'
					}
					else if `r(N)' == 0 {
					matrix mean_lag_`cohort'_`age'_`sex' =
mean_lag_`cohort'_`age'_`sex' \ 0
					}
					else {
					matrix mean_lag_`cohort'_`age'_`sex' =
mean_lag_`cohort'_`age'_`sex' \ `r(mean)'
					}
					}
					matrix list mean_lag_`cohort'_`age'_`sex'

					}
					}
					}

*Start of follow up when the infection occurred more than 10 years before dementia
gen entry_to_inf_10y = 10 + (ensianyinfpvm-entrypvm)/365.25
replace entry_to_inf_10y = 0 if entry_to_inf_10y < 0
*hist entry_to_inf_10y
sum entry_to_inf_10y
capture drop entry_to_inf_10y_cut
egen entry_to_inf_10y_cut = cut(entry_to_inf_10y), at(0 0.0009765625 5(5)30 100)
tab entry_to_inf_10y_cut
sum entry_to_inf_10y if entry_to_inf_10y_cut==0

levelsof cohort, local(cohorts)
levelsof age_cut, local(age_groups)
levelsof supu, local(sexes)
levelsof entry_to_inf_10y_cut, local(entry_lags)
local i 0
foreach cohort of local cohorts {
		foreach age of local age_groups {
				foreach sex of local sexes {
						local i 0
						foreach lag of local entry_lags {
								local ++i

								quietly: sum entry_to_inf_10y if cohort==`cohort' &
age_cut==`age' & supu==`sex' & entry_to_inf_10y_cut==`lag'

								if `i'==1 & `r(N)' == 0 {
								matrix mean_lag_10y_`cohort'_`age'_`sex' = 0
								}
								else if `i'==1 {
								matrix mean_lag_10y_`cohort'_`age'_`sex' = `r(mean)'
								}
								else if `r(N)' == 0 {
								matrix mean_lag_10y_`cohort'_`age'_`sex' =
mean_lag_10y_`cohort'_`age'_`sex' \ 0
```

```
}
else {
matrix mean_lag_10y_`cohort'_`age'_`sex' =
mean_lag_10y_`cohort'_`age'_`sex' \ `r(mean)'
}
}
matrix list mean_lag_10y_`cohort'_`age'_`sex'

}
}
}

*Return random order
capture drop sub_order
bysort cohort age_cut supu anyanyinf (shuffle1 shuffle2): gen sub_order = _n

*Similar distribution of delays in start of follow up for those not exposed to infections
capture drop entry_lag
gen entry_lag = .
levelsof cohort, local(cohorts)
levelsof age_cut, local(age_groups)
levelsof supu, local(sexes)
levelsof entry_to_inf_cut, local(entry_lags)
foreach cohort of local cohorts {
		foreach age of local age_groups {
				foreach sex of local sexes {
						local i 0
						local cum = 0
						sum id if cohort==`cohort' & age_cut==`age' & supu==`sex' & anyanyinf==0

						local N_control = `r(N)'
						sum entry_to_inf_cut if cohort==`cohort' & age_cut==`age' & supu==`sex' &
anyanyinf==1
						local N_`cohort'_`age'_`sex' = `r(N)'
						foreach lag of local entry_lags {
								di "cohort `cohort', age_group `age', sex `sex'"
								local ++i
								di `i'
								sum entry_to_inf_cut if cohort==`cohort' & age_cut==`age' &
supu==`sex' & anyanyinf==1 & entry_to_inf_cut==`lag'
								local cum = `cum' + `r(N)'
								di `cum'
								di `N_`cohort'_`age'_`sex''
								di `N_control'
								di mean_lag_`cohort'_`age'_`sex'[`i',1]
								replace entry_lag = mean_lag_`cohort'_`age'_`sex'[`i',1] if
cohort==`cohort' & age_cut==`age' & supu==`sex' & anyanyinf==0 & entry_lag==. & sub_order <= round((`cum' /
`N_`cohort'_`age'_`sex'')*`N_control')
								}
								replace entry_lag = 0 if `N_`cohort'_`age'_`sex''==0 &
entry_lag==. & anyanyinf==0 & cohort==`cohort' & supu==`sex'
								}
								}
								}

*Delays for the 10y exclusion analysis
capture drop entry_lag_10y
gen entry_lag_10y = .
```

```
levelsof cohort, local(cohorts)
levelsof age_cut, local(age_groups)
levelsof supu, local(sexes)
levelsof entry_to_inf_10y_cut, local(entry_lags)
foreach cohort of local cohorts {
		foreach age of local age_groups {
				foreach sex of local sexes {
						local i 0
						local cum = 0
						sum id if cohort==`cohort' & age_cut==`age' & supu==`sex' & anyanyinf==0

						local N_control = `r(N)'
						sum entry_to_inf_10y_cut if cohort==`cohort' & age_cut==`age' & supu==`sex'
& anyanyinf==1
						local N_`cohort'_`age'_`sex' = `r(N)'
						foreach lag of local entry_lags {
								di "cohort `cohort', age_group `age', sex `sex'"
								local ++i
								di `i'
								sum entry_to_inf_10y_cut if cohort==`cohort' & age_cut==`age'
& supu==`sex' & anyanyinf==1 & entry_to_inf_10y_cut==`lag'
								local cum = `cum' + `r(N)'
								di `cum'
								di `N_`cohort'_`age'_`sex''
								di `N_control'
								di mean_lag_10y_`cohort'_`age'_`sex'[`i',1]
								replace entry_lag_10y =
mean_lag_10y_`cohort'_`age'_`sex'[`i',1] if cohort==`cohort' & age_cut==`age' & supu==`sex' & anyanyinf==0 &
entry_lag_10y==. & sub_order <= round((`cum' / `N_`cohort'_`age'_`sex'')*`N_control')
								}
								replace entry_lag_10y = 0 if `N_`cohort'_`age'_`sex''==0 &
entry_lag_10y==. & anyanyinf==0 & cohort==`cohort' & supu==`sex'
								}
								}
								}


* Check  that the distribution are correct:
sum entry_to_inf_10y
sum entry_lag_10y
levelsof cohort, local(cohorts)
levelsof age_cut, local(age_groups)
levelsof supu, local(sexes)
levelsof entry_to_inf_10y_cut, local(entry_lags)
foreach cohort of local cohorts {
		foreach age of local age_groups {
				foreach sex of local sexes {
						sum entry_to_inf if cohort==`cohort' & age_cut==`age' & supu==`sex'
						sum entry_lag if cohort==`cohort' & age_cut==`age' & supu==`sex'
						sum entry_to_inf_10y if cohort==`cohort' & age_cut==`age' & supu==`sex'
						sum entry_lag_10y if cohort==`cohort' & age_cut==`age' & supu==`sex'
								}
								}
								}


save "jakauma.dta", replace
```

```
use "jakauma.dta", clear
*Update the infection variables so that they are coded missing if the participant does not have the index infection but has some other infection
local dg anyinf bactinf sysbact localbact bactsepsis bact_nonsepsis extrac intrac gramplus gramminus viralinf herpes persviral acuteviral
foreach idg of local dg {
				tab any`idg'
				replace any`idg' = . if anyanyinf==1 & any`idg'==0
				tab any`idg'

				egen lag_entry_`idg' = rowmax(entrypvm ensi`idg'pvm) if any`idg'==1
				replace lag_entry_`idg' = entrypvm + round(entry_lag*365.25) if any`idg'==0
				capture drop ensi`idg'pvm_10y
				gen ensi`idg'pvm_10y = ensi`idg'pvm + round(10*365.25)
				egen lag_entry_10y_`idg' = rowmax(entrypvm ensi`idg'pvm_10y) if any`idg'==1
				replace lag_entry_10y_`idg' = entrypvm + round(entry_lag_10y*365.25) if any`idg'==0
				}

save "Inf_dem_lag.dta", replace



****** Descriptive analysis and definition of variables *************
use "Inf_dem_lag.dta", replace

*Katsotaan eri infektioiden määrät
tab anyanyinf

tab anybactinf

tab anysysbact
tab anylocalbact

tab anybactsepsis
tab anybact_nonsepsi

tab anyextrac
tab anyintrac

tab anygramplus
tab anygramminus
tab anymycobact
tab anymycoplasma

tab anyviralinf
tab anyherpes
tab anypersviral
tab anyacuteviral

tab anyparasite
tab anymycose

*Piirretään histogrammi dementian iästä

stset exitpvm, id(id) failure(ensidementia) origin(time syntpvm) enter(time lag_entry_anyinf) scale(365.25)

egen latedementia = cut(_t), at(0,65,100), if _d==1
tab latedementia
```

```
gen dementia_age = int(_t)+0.5 if _d==1
histogram dementia_age, disc scheme(s1color) freq ///
			ysize(7.8) xsize(8.27) ///
			title(" ", size(medsmall) color(black)) ///
			graphregion(color(white)) ///
			xtitle("Age (years)", size(medsmall) color(black)) ///
			ytitle("Dementias, No.", size(medsmall) color(black))
graph export "eFigure4_new.emf", as(emf) replace
graph export "eFigure4_new.pdf", as(pdf) replace


				use "Inf_dem_lag.dta", replace
				merge 1:1 id using "Parkinson.dta"
				keep if _merge==3
				drop _merge


stset exitpvm, id(id) failure(ensidementia) origin(time syntpvm) enter(time lag_entry_anyinf) scale(365.25)
			keep if _st==1
			count
			count if ensidementia==1

				label define supu 1"Men" 2"Women"
				label values supu supu
				label values diabetes yesno
				label values hypertensio yesno
				recode ensidementia .=0
				label values ensidementia yesno

				gen followup = _t-_t0

				drop age
				gen age = (lag_entry_anyinf - syntpvm)/365.25
				egen age_cat = cut(age), at(18,40,50,60,100)
				tab age_cat

				label define alcocl 0 "Non-drinker" 1 "Moderate" 2 "Intermediate" 3 "Heavy"
				label values alcocl alcocl

				foreach cm in hypert diabetes ihd cereb parkinson {
					gen `cm'_entry= `cm'_combpvm<=lag_entry_anyinf
					tab `cm'_entry
					}

				gen age_at_dementia = _t if _d==1

				keep if _st==1
				keep if anyanyinf!=.

				baselinetable ///
				age_cat(novarlabel afterhead("Age at entry, years")) ///
				supu(novarlabel afterhead("Sex")) ///
				ses(novarlabel afterhead("Education/Socioeconomic status")) ///
				hypert_entry(novarlabel afterhead("Hypertension")) ///
				diabetes_entry(novarlabel afterhead("Diabetes mellitus")) ///
				ihd_entry(novarlabel afterhead("Ischaemic heart disease")) ///
				cereb_entry(novarlabel afterhead("Cerebrovascular disease")) ///
```

```
                                         parkinson_entry(novarlabel afterhead("Parkinson's disease")) ///
                                         tupakka(novarlabel afterhead("Smoking status")) ///
                                         alcocl(novarlabel afterhead("Alcohol drinking")) ///
                                         followup(cts novarlabel afterhead("Follow-up, median (IQR), years")) ///
                                         ensidementia (novarlabel afterhead("Dementia")) ///
                                         age_at_dementia(cts novarlabel afterhead("Age at dementia, median (IQR), years")) ///
                                         , by(cohort, total) ctsvartab(p50 (p25-p75)) missing ///
                                         exportexcel(eTable_3, cell(A6) replace)

tab ensidementia    // byte   %8.0g          any dementia diagnosis (yes/no)
tab dementiatyyppi              //
                                          type of dementia
sum anydementiapvm  // int    %9.0g           date of first dementia diagnosis

tab anyanyinf     // byte   %8.0g    yesno    Hospitalisation for any infectious disease

tab anybactinf    // byte   %8.0g    yesno    Hospitalisation for any bacterial infection

tab anysysbact    // byte   %8.0g    yesno    Hospitalisation for any potentially invasive bacterial infections
tab anylocalbact   // byte   %8.0g    yesno    Hospitalisation for any localised bacterial infection

tab anygramplus    // byte   %8.0g    yesno    Hospitalisation for any Gram-positive bacterial infection

tab anygramminus   // byte   %8.0g    yesno    Hospitalisation for any Gram-negative bacterial infection

tab anymycobact    // byte   %8.0g    yesno    Hospitalisation for any mycobacterial infection

tab anymycoplasma  // byte   %8.0g    yesno    Hospitalisation for any mycoplasma infection

tab anyextrac     // byte   %8.0g    yesno    Hospitalisation for any extracellular bacterial infection
tab anyintrac     // byte   %8.0g    yesno    Hospitalisation for any intracellular bacterial infection

tab anyviralinf    // byte   %8.0g    yesno    Hospitalisation for any viral infection

tab anyacuteviral  // byte   %8.0g    yesno    Hospitalisation for any acute viral infection
tab anyherpes     // byte   %8.0g    yesno    Hospitalisation for any herpesvirus infection
tab anypersviral   // byte   %8.0g    yesno    Hospitalisation for any potentially persistent viral infection (excluding herpesvirus
infections)

tab anyparasite    // byte   %8.0g    yesno    Hospitalisation for any parasitic infection

tab anymycose     // byte   %8.0g    yesno    Hospitalisation for any fungal infection (mycosis)

tab anybactsepsis                            // byte   %8.0g    yesno    Hospitalisation for bacterial infection with sepsis
tab anybact_nonsepsis           // byte   %8.0g    yesno    Hospitalisation for bacterial infection without sepsis

*For all categories of infections listed above, there exists a variable for the date of first hospitalisation for an infection in that
category.
*The variable is named "ensi(name_of_disease)pvm" where "name_of_disease" is that of the variable above when the prefix
"any" is removed.

tab anyhiv                                   //
                                             Known HIV (yes/no)

sum entrypvm                                 //
                                         date of entry to cohort
sum exitpvm                                          //
                                             date of end of follow-up
```

```
sum syntpvm		//
			date of birth

tab ses								//
								socioeconomic status (low, intermediate, high)
tab supu						//
							sex (man/woman)
tab alcocl						//
							alcohol drinking (no, light, moderate, heavy)
tab tupakka						//
							smoking (never, ex, current)
tab bmi_who						//
							body mass index (normal weight, overweight, obese)
sum hypert_combpvm		//
				date of hypertension diagnosis
sum diabetes_combpvm //
			date of diabetes diagnosis
sum ihd_combpvm						//
							date of ischaemic heart disease diagnosis
sum cereb_combpvm		//
				date of cerebrovascular disease diagnosis
sum parkinson_combpvm		//
				date of cerebrovascular disease diagnosis



*** PRIMARY ANALYSES USING THREE FINNISH COHORTS ***

*Incidence of infections

capture log close
log using "Incidence of infections and dementia.log", replace

use "Inf_dem_lag.dta", clear
stset exitpvm, id(id) failure(ensidementia) origin(time syntpvm) enter(time lag_entry_anyinf) scale(365.25)
keep if _st==1
gen inf_at_entry = ensianyinfpvm<=entrypvm
tab inf_at_entry
gen byte inf_at_fu = ensianyinfpvm<exitpvm & inf_at_entry==0
tab inf_at_fu
egen infexit = rowmin(ensianyinfpvm exitpvm)
stset infexit, id(id) failure(inf_at_fu) origin(time syntpvm) enter(time entrypvm) scale(365.25)
stptime, per(100000)
*seed from random.org from range 1 - 1 000 000 000
set seed 519750045
preserve
drop if _st!=1
bootstrap incidence = r(rate), reps(1000) strata(cohort): stptime, per(100000)
estat bootstrap, all
*Percentile-based confidence intervals were reported in the manuscript.
restore

capture log close
log using "FIGURE 2 dist.log", replace

*FIGURE 2.

use "Inf_dem_lag.dta", clear
```

```
local dg anyinf bactinf sysbact localbact bactsepsis bact_nonsepsis extrac intrac gramplus gramminus viralinf herpes persviral acuteviral
local row 7 10 13 14 16 17 20 21 24 25 28 31 32 33
local n_dg: word count `dg'
local dg_name `""Any infectious disease vs no infection" "Any bacterial infection vs no infection" "Potentially invasive bacterial infection vs no infection"  "Localised bacterial infection vs no infection" "Bacterial infection with sepsis vs no infection" "Bacterial infection without sepsis vs no infection" "Extracellular bacterial infection vs no infection" "Intracellular bacterial infection vs no infection" "Gram-positive bacterial infection vs no infection" "Gram-negative bacterial infection vs no infection" "Any viral infection vs no infection" "Herpesvirus (persistent) infection vs no infection" "Other persistent viral infection vs no infection" "Acute viral infection vs no infection""'
forvalues i=1/`n_dg' {
		quietly: local idg : word `i' of `dg'
		quietly: local idg_name : word  `i' of `dg_name'
		quietly: local irow : word `i' of `row'
		display ""
		display "`idg' followed by broad dementia, adjusted for sex and education and stratified by cohort, age as time-scale"
		display ""
		display "Pooled IPD-analysis, normal model and 10-year exclusion"
		display ""
		display "$S_TIME  $S_DATE"
		display ""

		preserve

		stset exitpvm, id(id) failure(ensidementia) origin(time syntpvm) enter(time lag_entry_`idg') scale(365.25)
		stdescribe
		stdescribe if any`idg'==0
		stdescribe if any`idg'==1
		quietly: keep id any`idg' supu cohort exitpvm ensidementia syntpvm entrypvm ensi`idg'pvm lag_entry_`idg' lag_entry_10y_`idg' _st _d _origin _t _t0 ses
		keep if _st==1
		tab any`idg'
		tab any`idg' ensidementia

		stcox any`idg' 2.supu#cohort 2.ses#cohort 3.ses#cohort, strata(cohort)
		quietly: matrix m = r(table)
		tempname hr ll ul
		scalar `hr' = m[1,1]
		scalar `ll' = m[5,1]
		scalar `ul' = m[6,1]
		quietly: putexcel set "Pooled results.xlsm", sheet("FIGURE 2 dist") modify
			quietly: putexcel K`irow'=(`e(N_sub)'), nformat(number)
			quietly: putexcel L`irow'=(`e(N_fail)'), nformat(number)
			quietly: putexcel E`irow'=(`hr'), nformat(number_d2)
			quietly: putexcel F`irow'=(`ll'), nformat(number_d2)
			quietly: putexcel G`irow'=(`ul'), nformat(number_d2)
			quietly: putexcel B`irow'=("`idg'")
			quietly: putexcel C`irow'=("pooled")
			quietly: putexcel D`irow'=("`idg_name'")
		tab any`idg', matcell(x), if _st==1
		matrix list x
		quietly: tab any`idg' ensidementia, matcell(y), if _d==1
		matrix list y
		quietly: putexcel set "Pooled results.xlsm", sheet("FIGURE 2 dist") modify
			quietly: putexcel M`irow'=(x[2,1]), nformat(number)
```

```
				quietly: putexcel N`irow'=(y[2,1]), nformat(number)
			local irow = `irow' + 29

			*Dementia occurring from year 10 onwards
			stset exitpvm, id(id) failure(ensidementia) origin(time syntpvm) enter(time lag_entry_10y_`idg')
scale(365.25)
			stdescribe
			stdescribe if any`idg'==0
			stdescribe if any`idg'==1

			stcox any`idg' 2.supu#cohort 2.ses#cohort 3.ses#cohort, strata(cohort)
			quietly: matrix m = r(table)
			scalar `hr' = m[1,1]
			scalar `ll' = m[5,1]
			scalar `ul' = m[6,1]
			quietly: putexcel set "Pooled results.xlsm", sheet("FIGURE 2 dist") modify
				quietly: putexcel K`irow'=(`e(N_sub)'), nformat(number)
				quietly: putexcel L`irow'=(`e(N_fail)'), nformat(number)
				quietly: putexcel E`irow'=(`hr'), nformat(number_d2)
				quietly: putexcel F`irow'=(`ll'), nformat(number_d2)
				quietly: putexcel G`irow'=(`ul'), nformat(number_d2)
				quietly: putexcel B`irow'=("`idg'")
				quietly: putexcel C`irow'=("pooled")
				quietly: putexcel D`irow'=("`idg_name'")
			tab any`idg', matcell(x), if _st==1
			matrix list x
			quietly: tab any`idg' ensidementia, matcell(y), if _d==1
			matrix list y
			quietly: putexcel set "Pooled results.xlsm", sheet("FIGURE 2 dist") modify
				quietly: putexcel M`irow'=(x[2,1]), nformat(number)
				quietly: putexcel N`irow'=(y[2,1]), nformat(number)
			restore
			}
log close

***Age-standardised incidence of dementia

*()Information form FIGURE 2 that goes to appendix)
capture log close
log using "FIGURE 2 append.log", replace

use "Inf_dem_lag.dta", clear

local dg anyinf bactinf sysbact localbact bactsepsis bact_nonsepsis extrac intrac gramplus gramminus viralinf herpes persviral acuteviral
local row 7 10 13 14 16 17 20 21 24 25 28 31 32 33
local n_dg: word count `dg'
local dg_name `""Any infectious disease vs no infection" "Any bacterial infection vs no infection" "Potentially invasive bacterial infection vs no infection"  "Localised bacterial infection vs no infection" "Bacterial infection with sepsis vs no infection" "Bacterial infection without sepsis vs no infection" "Extracellular bacterial infection vs no infection" "Intracellular bacterial infection vs no infection" "Gram-positive bacterial infection vs no infection" "Gram-negative bacterial infection vs no infection" "Any viral infection vs no infection" "Herpesvirus (persistent) infection vs no infection" "Other persistent viral infection vs no infection" "Acute viral infection vs no infection""'
forvalues i=1/`n_dg' {
			quietly: local idg : word `i' of `dg'
			quietly: local idg_name : word  `i' of `dg_name'
			quietly: local irow : word `i' of `row'
			display ""
```

```
				display "`idg' followed by broad dementia, adjusted for sex and education and stratified by
cohort, age as time-scale"
				display ""
				display "Pooled IPD-analysis, normal model and 10-year exclusion"
				display ""
				display "$S_TIME  $S_DATE"
				display ""

				if "`idg'" == "anyinf" {
				stset exitpvm, id(id) failure(ensidementia) origin(time syntpvm) enter(time lag_entry_`idg')
scale(365.25)
				keep if _st==1
				*define scalars
				tempname base_pt_18 base_pt_30 base_pt_35 base_pt_40 base_pt_45 base_pt_50
base_pt_55 base_pt_60 ///
				base_pt_65 base_pt_70 base_pt_75 base_pt_80 base_pt_85
				tempname base_rate_18_10y base_rate_30_10y base_rate_35_10y base_rate_40_10y
base_rate_45_10y ///
				base_rate_50_10y base_rate_55_10y base_rate_60_10y base_rate_65_10y base_rate_70_10y
///
				base_rate_75_10y base_rate_80_10y base_rate_85_10y
				tempname inf18 inf30 inf35 inf40 inf45 inf50 inf55 inf60 inf65 inf70 inf75 inf80 inf85
				tempname ptime_base incid_base stand_incid_base_10y stand_incid
				stptime, per(100000), if anyanyinf==0
				scalar `ptime_base' = r(ptime)
				scalar `incid_base' = r(rate)
				}

				preserve

				if "`idg'" != "anyinf" {
				stset exitpvm, id(id) failure(ensidementia) origin(time syntpvm) enter(time lag_entry_`idg')
scale(365.25)
				keep if _st==1
				}

				tab any`idg', matcell(x), if _st==1
				matrix list x
				quietly: tab any`idg' ensidementia, matcell(y), if _d==1
				matrix list y
				sum _st if _st==1
				local N = `r(N)'
				sum _d if _d==1
				local failures = `r(N)'

				*Store person-times and rates to scalars
				stsplit age_split, at(18 30(5)85)
				tab age_split
				levelsof age_split, local(age_groups)
				di `age_groups'
				foreach age of local age_groups {
						capture noisily stptime, per(100000), if age_split==`age' & anyanyinf==0
						if _rc == 0 { // _rc is the return code of the command following capture. 0 means
that there was no error (e.g., due to no observations)).
						scalar `base_pt_`age'' = r(ptime)
						}
						else {
						scalar `base_pt_`age'' = 0
```

```
			}
			capture noisily stptime, per(100000), if age_split==`age' & anyanyinf==1
			if _rc == 0  {
			scalar `inf`age'' = r(rate)
			}
			else {
			scalar `inf`age'' = 0
			}
		}
		*Compute age-standardised incidence rate (per 100 000 person years)
		scalar `stand_incid' = (`base_pt_18'*`inf18' + `base_pt_30'*`inf30' + `base_pt_35'*`inf35' + 
`base_pt_40'*`inf40' + `base_pt_45'*`inf45' ///
		+ `base_pt_50'*`inf50' + `base_pt_55'*`inf55' + `base_pt_60'*`inf60' + `base_pt_65'*`inf65' + 
`base_pt_70'*`inf70' + `base_pt_75'*`inf75' ///
		+ `base_pt_80'*`inf80' + `base_pt_85'*`inf85') / `ptime_base'
		di "Age-standardised incidence rate is " `stand_incid'

		quietly: putexcel set "Pooled results.xlsm", sheet("FIGURE 2 append") modify
			quietly: putexcel M`irow'=(x[2,1]), nformat(number)
			quietly: putexcel N`irow'=(y[2,1]), nformat(number)
			quietly: putexcel K`irow'=(`N'), nformat(number)
			quietly: putexcel L`irow'=(`failures'), nformat(number)
			quietly: putexcel G`irow'=(`incid_base'), nformat(number_d2)
			quietly: putexcel H`irow'=(`stand_incid'), nformat(number_d2)
			quietly: putexcel B`irow'=("`idg'")
			quietly: putexcel C`irow'=("pooled")
			quietly: putexcel D`irow'=("`idg_name'")

		restore

		*Dementia occurring from year 10 onwards
		local irow = `irow' + 29

		preserve
		stset exitpvm, id(id) failure(ensidementia) origin(time syntpvm) enter(time lag_entry_10y_`idg') 
scale(365.25)
		keep if _st==1

		tab any`idg', matcell(x), if _st==1
		matrix list x
		quietly: tab any`idg' ensidementia, matcell(y), if _d==1
		matrix list y
		sum _st if _st==1
		local N = `r(N)'
		sum _d if _d==1
		local failures = `r(N)'

		*Store person-times and rates to scalars
		stsplit age_split, at(18 30(5)85)
		levelsof age_split, local(age_groups)
		foreach age of local age_groups {
			capture noisily stptime, per(100000), if age_split==`age' & anyanyinf==1
			if _rc == 0  {
			scalar `inf`age'' = r(rate)
			}
			else {
			scalar `inf`age'' = 0
			}
```

```
					}

				if "`idg'" == "anyinf" {
					levelsof age_split, local(age_groups)
					foreach age of local age_groups {
						capture noisily stptime, per(100000), if age_split==`age' &
anyanyinf==0
						if _rc == 0  {
						scalar `base_rate_`age'_10y' = r(rate)
						}
						else {
						scalar `base_rate_`age'_10y' = 0
						}
					}
					}

				if "`idg'" == "anyinf" {
					scalar `stand_incid_base_10y' = (`base_pt_18'*`base_rate_18_10y' +
`base_pt_30'*`base_rate_30_10y' + `base_pt_35'*`base_rate_35_10y' + ///
					`base_pt_40'*`base_rate_40_10y' + `base_pt_45'*`base_rate_45_10y' +
`base_pt_50'*`base_rate_50_10y' ///
					+ `base_pt_55'*`base_rate_55_10y' + `base_pt_60'*`base_rate_60_10y' +
`base_pt_65'*`base_rate_65_10y' + `base_pt_70'*`base_rate_70_10y' + ///
					`base_pt_75'*`base_rate_75_10y' + `base_pt_80'*`base_rate_80_10y' +
`base_pt_85'*`base_rate_85_10y') / `ptime_base'

				di "Age-standardised incidence for comparison group in 10-year lag analysis is "
`stand_incid_base_10y'

				}

				*Compute age-standardised incidence rate (per 100 000 person years)
				scalar `stand_incid' = (`base_pt_18'*`inf18' + `base_pt_30'*`inf30' + `base_pt_35'*`inf35' +
`base_pt_40'*`inf40' + `base_pt_45'*`inf45' ///
				+ `base_pt_50'*`inf50' + `base_pt_55'*`inf55' + `base_pt_60'*`inf60' + `base_pt_65'*`inf65' +
`base_pt_70'*`inf70' + `base_pt_75'*`inf75' ///
				+ `base_pt_80'*`inf80' + `base_pt_85'*`inf85') / `ptime_base'
				di "Age-standardised incidence rate is " `stand_incid' " in 10-year lag analysis"
				quietly: putexcel set "Pooled results.xlsm", sheet("FIGURE 2 append") modify
					quietly: putexcel M`irow'=(x[2,1]), nformat(number)
					quietly: putexcel N`irow'=(y[2,1]), nformat(number)
					quietly: putexcel K`irow'=(`N'), nformat(number)
					quietly: putexcel L`irow'=(`failures'), nformat(number)
					quietly: putexcel G`irow'=(`stand_incid_base_10y'), nformat(number_d2)
					quietly: putexcel H`irow'=(`stand_incid'), nformat(number_d2)
					quietly: putexcel B`irow'=("`idg'")
					quietly: putexcel C`irow'=("pooled")
					quietly: putexcel D`irow'=("`idg_name'")

				restore
					}
log close

*** CUMULATIVE HAZARD *****

use "Inf_dem_lag.dta", clear
stset exitpvm, id(id) failure(ensidementia) origin(time syntpvm) enter(time lag_entry_anyinf) scale(365.25)
```

```
sts graph, na by(anyanyinf) ///
title("Any infectious disease" "vs no infection" " " ///
, size(medsmall) color(black))  xti("Age (years)") yti("Nelson-Aalen cumulative hazard estimate",
height(5)) ///
legend(order(1 "No infection"  2 "Infection")) scheme(s1color) ///
plot1opts(lcolor(blue)) plot2opts(lcolor(red)) xscale(range(18 100)) xlabel(20(20)100) ///
risktable(20 40 60 80 100, size(small) failevents order(1 "No infection" 2 "Infection") ///
title("N at risk (dementias)", justification(left) size(small)) rowtitle(, justification(left)))
graph export "age_NA_anyinf.emf", as(emf) replace




***eFIGURE 8 ****
*(was previously figure 3)

capture log close
log using "FIGURE 3 dist.log", replace

use "Inf_dem_lag.dta", clear

*Use this first listed dementia diagnosis if there are several on the same hospitalisation (primary diagnosis instead of secondary
and 1st secondary instead of 2nd secondary and so on)
drop dementiatyyppi
gen dementiatyyppi = 1 if dementiatyyppi_laaja=="AD"
replace dementiatyyppi = 2 if inlist(dementiatyyppi_laaja,"FTD","PD","VD","other","unspecified")

local dg anyinf bactinf sysbact localbact bactsepsis bact_nonsepsis extrac intrac gramplus gramminus viralinf herpes persviral
acuteviral
local row 7 11 15 18 21 24 28 31 35 38 42 46 49 52
local n_dg: word count `dg'
local dg_name `""Any infectious disease vs not" "Any bacterial infection vs not" "Potentially invasive bacterial infection vs not"
"Localised bacterial infection vs not" "Bacterial infection with sepsis vs not" "Bacterial infection without sepsis vs not"
"Extracellular bacterial infection vs not" "Intracellular bacterial infection vs not" "Gram-positive bacterial infection vs not"
"Gram-negative bacterial infection vs not" "Any viral infection vs not" "Herpesvirus (persistent) infection vs not" "Other
persistent viral infection vs not" "Acute viral infection vs not""'
	forvalues i=1/`n_dg' {
		quietly: local idg : word `i' of `dg'
		quietly: local idg_name : word  `i' of `dg_name'
		quietly: local irow : word `i' of `row'
		display ""
		display "`idg' followed by broad dementia, adjusted for sex and education and stratified by
cohort"
		display ""
		display "Pooled IPD-analysis, normal model and 10-year exclusion"
		display ""
		display "$S_TIME  $S_DATE"
		display ""

		*Alzheimer's disease dementia
		preserve
		drop if ses==.
		quietly: stset exitpvm, id(id) failure(dementiatyyppi == 1) origin(time syntpvm) enter(time
lag_entry_`idg') scale(365.25)
		quietly: keep id any`idg' supu cohort exitpvm ensidementia syntpvm entrypvm ensi`idg'pvm  _st
_d _origin _t _t0 ses dementiatyyppi
		tab any`idg' if _st==1
```

```
stcox any`idg' 2.supu#cohort 2.ses#cohort 3.ses#cohort, strata(cohort)
quietly: matrix m = r(table)
tempname hr ll ul
scalar `hr' = m[1,1]
scalar `ll' = m[5,1]
scalar `ul' = m[6,1]
quietly: putexcel set "Pooled results.xlsm", sheet("FIGURE 3 dist") modify
        quietly: putexcel K`irow'=(`e(N_sub)'), nformat(number)
        quietly: putexcel L`irow'=(`e(N_fail)'), nformat(number)
        quietly: putexcel E`irow'=(`hr'), nformat(number_d2)
        quietly: putexcel F`irow'=(`ll'), nformat(number_d2)
        quietly: putexcel G`irow'=(`ul'), nformat(number_d2)
        quietly: putexcel B`irow'=("`idg'_AD")
        quietly: putexcel C`irow'=("pooled")
        quietly: putexcel D`irow'=("`idg_name'")
tab any`idg', matcell(x), if _st==1
matrix list x
quietly: tab any`idg' ensidementia, matcell(y), if _d==1
matrix list y
quietly: putexcel set "Pooled results.xlsm", sheet("FIGURE 3 dist") modify
        quietly: putexcel M`irow'=(x[2,1]), nformat(number)
        quietly: putexcel N`irow'=(y[2,1]), nformat(number)
local ++irow
restore

*Non-Alzheimer's disease dementia
preserve
quietly: stset exitpvm, id(id) failure(dementiatyyppi == 2 3 4) origin(time syntpvm) enter(time lag_entry_`idg') scale(365.25)
quietly: keep id any`idg' supu cohort exitpvm ensidementia syntpvm entrypvm ensi`idg'pvm  _st _d _origin _t _t0 ses dementiatyyppi
tab any`idg' if _st==1

stcox any`idg' 2.supu#cohort 2.ses#cohort 3.ses#cohort, strata(cohort)
quietly: matrix m = r(table)
tempname hr ll ul
scalar `hr' = m[1,1]
scalar `ll' = m[5,1]
scalar `ul' = m[6,1]
quietly: putexcel set "Pooled results.xlsm", sheet("FIGURE 3 dist") modify
        quietly: putexcel K`irow'=(`e(N_sub)'), nformat(number)
        quietly: putexcel L`irow'=(`e(N_fail)'), nformat(number)
        quietly: putexcel E`irow'=(`hr'), nformat(number_d2)
        quietly: putexcel F`irow'=(`ll'), nformat(number_d2)
        quietly: putexcel G`irow'=(`ul'), nformat(number_d2)
        quietly: putexcel B`irow'=("`idg'_nonAD")
        quietly: putexcel C`irow'=("pooled")
        quietly: putexcel D`irow'=("`idg_name'")
tab any`idg', matcell(x), if _st==1
matrix list x
quietly: tab any`idg' ensidementia, matcell(y), if _d==1
matrix list y
quietly: putexcel set "Pooled results.xlsm", sheet("FIGURE 3 dist") modify
        quietly: putexcel M`irow'=(x[2,1]), nformat(number)
        quietly: putexcel N`irow'=(y[2,1]), nformat(number)
restore
}
```

```
log close


*FIGURE sensitivity, acute and chronic infections
capture log close
log using "Sensitivity, acute, chronic, coinfectionsm, dist.log", replace
local dg nonpers anypers bactviralcoinf
local n_dg: word count `dg'
local dg_name `""Any acute infectious disease" "Any chronic infectious disease""'

				quietly: use "Inf_dem_lag.dta", clear
				merge 1:1 id using "Coinfections_trimmattu"
				drop _merge
				foreach idg of local dg {
						tab any`idg'
						replace any`idg' = . if any`idg'== 0 & anyanyinf == 1
						tab any`idg'
						egen lag_entry_`idg' = rowmax(lag_entry_anyinf ensi`idg'pvm)

						gen ensi`idg'pvm_10y = ensi`idg'pvm + round(10*365.25)
						egen lag_entry_10y_`idg' = rowmax(lag_entry_10y_anyinf ensi`idg'pvm_10y)
				}
				drop if ses==.

quietly: local irow = 7
		forvalues i=1/`n_dg' {
				preserve
				quietly: local idg : word `i' of `dg'
				quietly: local idg_name : word  `i' of `dg_name'
				display ""
				display "`idg' followed by broad dementia, adjusted for sex and education and stratified by
cohort"
				display ""
				display "Pooled IPD-analysis, normal model and 10-year exclusion"
				display ""
				display "$S_TIME  $S_DATE"
				display ""

				quietly: stset exitpvm, id(id) failure(ensidementia) origin(time syntpvm) enter(time
lag_entry_`idg') scale(365.25)
				quietly: keep id any`idg' supu cohort exitpvm ensidementia syntpvm entrypvm ensi`idg'pvm  _st
_d _origin _t _t0 ses
				tab any`idg' if _st==1
				tab any`idg' ensidementia if _st==1

				quietly: stdescribe
				quietly: local failures = `r(N_fail)'
				stcox any`idg' 2.supu#cohort 2.ses#cohort 3.ses#cohort, strata(cohort)
				quietly: matrix m = r(table)
				tempname hr ll ul
				scalar `hr' = m[1,1]
				scalar `ll' = m[5,1]
				scalar `ul' = m[6,1]
				quietly: putexcel set "Pooled results.xlsm", sheet("FIGURE sensitivity dist") modify
						quietly: putexcel K`irow'=(`e(N_sub)'), nformat(number)
						quietly: putexcel L`irow'=(`e(N_fail)'), nformat(number)
						quietly: putexcel E`irow'=(`hr'), nformat(number_d2)
						quietly: putexcel F`irow'=(`ll'), nformat(number_d2)
```

```
quietly: putexcel G`irow'=(`ul'), nformat(number_d2)
quietly: putexcel B`irow'=("`idg'")
quietly: putexcel C`irow'=("pooled")
quietly: putexcel D`irow'=("`idg_name'")
tab any`idg', matcell(x), if _st==1
matrix list x
quietly: tab any`idg' ensidementia, matcell(y), if _d==1
matrix list y
quietly: putexcel set "Pooled results.xlsm", sheet("FIGURE sensitivity dist") modify
quietly: putexcel M`irow'=(x[2,1]), nformat(number)
quietly: putexcel N`irow'=(y[2,1]), nformat(number)
local ++irow
restore

*Dementia occurring from year 10 onwards
preserve
quietly: stset exitpvm, id(id) failure(ensidementia) origin(time syntpvm) enter(time
lag_entry_10y_`idg') scale(365.25)
quietly: keep id any`idg' supu cohort exitpvm ensidementia syntpvm entrypvm ensi`idg'pvm  _st
_d _origin _t _t0 ses
tab any`idg' if _st==1
tab any`idg' ensidementia if _st==1


quietly: stdescribe
quietly: local failures = `r(N_fail)'
stcox any`idg' 2.supu#cohort 2.ses#cohort 3.ses#cohort, strata(cohort)
quietly: matrix m = r(table)
scalar `hr' = m[1,1]
scalar `ll' = m[5,1]
scalar `ul' = m[6,1]
quietly: putexcel set "Pooled results.xlsm", sheet("FIGURE sensitivity dist") modify
quietly: putexcel K`irow'=(`e(N_sub)'), nformat(number)
quietly: putexcel L`irow'=(`e(N_fail)'), nformat(number)
quietly: putexcel E`irow'=(`hr'), nformat(number_d2)
quietly: putexcel F`irow'=(`ll'), nformat(number_d2)
quietly: putexcel G`irow'=(`ul'), nformat(number_d2)
quietly: putexcel B`irow'=("   `idg'_10y_exclusion")
quietly: putexcel C`irow'=("pooled")
quietly: putexcel D`irow'=("   Dementia occurring more than 10 years after the
hospitalisation")
tab any`idg', matcell(x), if _st==1
matrix list x
quietly: tab any`idg' ensidementia, matcell(y), if _d==1
matrix list y
quietly: putexcel set "Pooled results.xlsm", sheet("FIGURE sensitivity dist") modify
quietly: putexcel M`irow'=(x[2,1]), nformat(number)
quietly: putexcel N`irow'=(y[2,1]), nformat(number)
local ++irow
local ++irow
restore
}
log close



***** SINGLE VS MULTIPLE INFECTIONS *******
```

```
capture log close
log using "Single vs multiple infections.log", replace

local dg_name `""Multiple infections""'
local irow 16
local prow = `irow'

				quietly: use "Inf_dem_lag.dta", clear
				merge 1:1 id using "Coinfections_trimmattu"
				drop _merge
				drop if ses==.
				egen lag_entry_multiinf = rowmax(lag_entry_anyinf ensitoinenanyinfpvm ensikolmasanyinfpvm)
				egen multiinfpvm = rowmax(ensianyinfpvm ensitoinenanyinfpvm ensikolmasanyinfpvm)
				gen multiinfpvm_10y = multiinfpvm +round(10*365.25)
				egen lag_entry_10y_multiinf = rowmax(lag_entry_10y_anyinf multiinfpvm_10y)

				preserve
				quietly: stset exitpvm, id(id) failure(ensidementia) origin(time syntpvm) enter(time
lag_entry_multiinf) scale(365.25)
				keep if _st==1
				quietly: keep id anyanyinf supu cohort exitpvm ensidementia syntpvm entrypvm
ensi*anyinfpvm *toinen* *kolmas*  _st _d _origin _t _t0 ses
				sum ensi*anyinfpvm

				gen byte multiinf = 0
				tab multiinf
				replace multiinf = 1 if anyanyinf==1
				tab multiinf
				replace multiinf = 2 if ensitoinenanyinfpvm!=.
				replace multiinf = 3 if ensikolmasanyinfpvm!=.
				tab multiinf

				quietly: stdescribe
				quietly: local failures = `r(N_fail)'
				tempname hr1 ll1 ul1 hr2 ll2 ul2 hr3 ll3 ul3 N1 N2 N3 fail1 fail2 fail3
				forvalues i =1/3 {
				stcox i.multiinf 2.supu#cohort 2.ses#cohort 3.ses#cohort, strata(cohort), if inlist(multiinf,0,`i')
				quietly: matrix m = r(table)
				matrix list m
				scalar `N`i'' = e(N_sub)
				scalar `fail`i'' = e(N_fail)
				scalar `hr`i'' = m[1,2]
				scalar `ll`i'' = m[5,2]
				scalar `ul`i'' = m[6,2]
				}

				forvalues i =1/3 {
				quietly: putexcel set "Pooled results.xlsm", sheet("FIGURE sensitivity dist") modify
					quietly: putexcel K`irow'=(`N`i''), nformat(number)
					quietly: putexcel L`irow'=(`fail`i''), nformat(number)
					quietly: putexcel E`irow'=(`hr`i''), nformat(number_d2)
					quietly: putexcel F`irow'=(`ll`i''), nformat(number_d2)
					quietly: putexcel G`irow'=(`ul`i''), nformat(number_d2)
					quietly: putexcel B`irow'=("Multiinf")
					quietly: putexcel C`irow'=("pooled")
					quietly: putexcel D`irow'=("Single vs multiple infections, infection No. `i'")
				tab multiinf, matcell(x), if inlist(multiinf,0,`i') & _st==1
				matrix list x
```

```
tab multiinf ensidementia, matcell(y), if inlist(multiinf,0,`i') & _d==1
matrix list y
quietly: putexcel set "Pooled results.xlsm", sheet("FIGURE sensitivity dist") modify
        quietly: putexcel M`irow'=(x[2,1]), nformat(number)
        quietly: putexcel N`irow'=(y[2,1]), nformat(number)
local ++irow
local ++irow
}
*P for trend
stcox multiinf 2.supu#cohort 2.ses#cohort 3.ses#cohort, strata(cohort)
quietly: matrix n = r(table)
matrix list n
tempname p
scalar `p' = n[4,1]
quietly: putexcel set "Pooled results.xlsm", sheet("FIGURE sensitivity dist") modify
        quietly: putexcel Q`prow'=(`p'), nformat(number)
restore

*Dementia occurring from year 10 onwards
preserve
quietly: stset exitpvm, id(id) failure(ensidementia) origin(time syntpvm) enter(time
lag_entry_10y_multiinf) scale(365.25)
keep if _st==1
quietly: keep id anyanyinf supu cohort exitpvm ensidementia syntpvm entrypvm
ensi*anyinfpvm *toinen* *kolmas*  _st _d _origin _t _t0 ses
sum ensi*anyinfpvm

gen byte multiinf = 0
tab multiinf
replace multiinf = 1 if anyanyinf==1
tab multiinf
replace multiinf = 2 if ensitoinenanyinfpvm!=.
replace multiinf = 3 if ensikolmasanyinfpvm!=.
tab multiinf

quietly: stdescribe
quietly: local failures = `r(N_fail)'
tempname hr1 ll1 ul1 hr2 ll2 ul2 hr3 ll3 ul3 N1 N2 N3 fail1 fail2 fail3
forvalues i =1/3 {
stcox i.multiinf 2.supu#cohort 2.ses#cohort 3.ses#cohort, strata(cohort), if inlist(multiinf,0,`i')
quietly: matrix m = r(table)
matrix list m
scalar `N`i'' = e(N_sub)
scalar `fail`i'' = e(N_fail)
scalar `hr`i'' = m[1,2]
scalar `ll`i'' = m[5,2]
scalar `ul`i'' = m[6,2]
}

local irow 17
forvalues i =1/3 {
quietly: putexcel set "Pooled results.xlsm", sheet("FIGURE sensitivity dist") modify
        quietly: putexcel K`irow'=(`N`i''), nformat(number)
        quietly: putexcel L`irow'=(`fail`i''), nformat(number)
        quietly: putexcel E`irow'=(`hr`i''), nformat(number_d2)
        quietly: putexcel F`irow'=(`ll`i''), nformat(number_d2)
        quietly: putexcel G`irow'=(`ul`i''), nformat(number_d2)
        quietly: putexcel B`irow'=("Multiinf")
```

```
					quietly: putexcel C`irow'=("pooled")
					quietly: putexcel D`irow'=("10-year lag, single vs multiple infections, infection
No. `i'")
				tab multiinf, matcell(x), if inlist(multiinf,0,`i') & _st==1
				matrix list x
				tab multiinf ensidementia, matcell(y), if inlist(multiinf,0,`i') & _d==1
				matrix list y
				quietly: putexcel set "Pooled results.xlsm", sheet("FIGURE sensitivity dist") modify
					quietly: putexcel M`irow'=(x[2,1]), nformat(number)
					quietly: putexcel N`irow'=(y[2,1]), nformat(number)
				local ++irow
				local ++irow
				}

				*P for trend
				stcox multiinf 2.supu#cohort 2.ses#cohort 3.ses#cohort, strata(cohort)
				quietly: matrix n = r(table)
				matrix list n
				tempname p
				scalar `p' = n[4,1]
				quietly: putexcel set "Pooled results.xlsm", sheet("FIGURE sensitivity dist") modify
					quietly: putexcel Q`prow'=(`p'), nformat(number)
				restore

log close

***** One or several different pathogens *******

capture log close
log using "One vs at least two different pathogens.log", replace

local dg_name `""Multiple organisms""'
local irow 82
local prow = `irow'

				quietly: use "Inf_dem_lag.dta", clear
				drop if ses==.
				egen ensibactviralpvm = rowmin(ensibactinfpvm ensiviralinfpvm)
				gen toinenbactviralpvm = ensibactviralpvm if ensibactinfpvm==ensiviralinfpvm &
ensibactinfpvm!=.
				replace toinenbactviralpvm = ensibactinfpvm if ensibactinfpvm>ensiviralinfpvm &
ensibactinfpvm!=.
				replace toinenbactviralpvm = ensiviralinfpvm if ensiviralinfpvm>ensibactinfpvm &
ensiviralinfpvm!=.
				gen anybactviral = 1 if ensibactviralpvm!=.
				gen toinenbactviral = 1 if toinenbactviralpvm!=.
				egen lag_entry_bactviral = rowmax(lag_entry_anyinf ensibactviralpvm toinenbactviralpvm)
				gen ensibactviralpvm_10y = ensibactviralpvm + round(10*365.25)
				gen toinenbactviralpvm_10y = toinenbactviralpvm + round(10*365.25)
				egen lag_entry_10y_bactviral = rowmax(lag_entry_10y_anyinf ensibactviralpvm_10y
toinenbactviralpvm_10y)

				preserve
				quietly: stset exitpvm, id(id) failure(ensidementia) origin(time syntpvm) enter(time
lag_entry_bactviral) scale(365.25)
				quietly: keep id anybactviral supu cohort exitpvm ensidementia syntpvm entrypvm ensi*pvm
*toinen* _st _d _origin _t _t0 ses anyanyinf
				sum ensibactviralpvm toinenbactviralpvm
```

```
tab anybactviral if _st==1
tab toinenbactviral if _st==1

gen byte multiinf = 0 if anyanyinf==0
tab multiinf
tab multiinf if _st==1
replace multiinf = 1 if anybactviral==1
tab multiinf
tab multiinf if _st==1
replace multiinf = 2 if toinenbactviral==1
tab multiinf
tab multiinf if _st==1
*browse id ensibactviralpvm toinenbactviralpvm multiinf _t0 _t

quietly: stdescribe
stcox i.multiinf 2.supu#cohort 2.ses#cohort 3.ses#cohort, strata(cohort), if multiinf!=2
quietly: matrix m = r(table)
matrix list m
tempname hr1 ll1 ul1 hr2 ll2 ul2 N1 fail1 N2 fail2
scalar `hr1' = m[1,2]
scalar `ll1' = m[5,2]
scalar `ul1' = m[6,2]
scalar `N1' = e(N_sub)
scalar `fail1' = e(N_fail)
stcox i.multiinf 2.supu#cohort 2.ses#cohort 3.ses#cohort, strata(cohort), if multiinf!=1
quietly: matrix m = r(table)
matrix list m
scalar `hr2' = m[1,2]
scalar `ll2' = m[5,2]
scalar `ul2' = m[6,2]
scalar `N2' = e(N_sub)
scalar `fail2' = e(N_fail)

forvalues i =1/2 {
quietly: putexcel set "Pooled results.xlsm", sheet("FIGURE sensitivity dist") modify
		quietly: putexcel K`irow'=(`N`i''), nformat(number)
		quietly: putexcel L`irow'=(`fail`i''), nformat(number)
		quietly: putexcel E`irow'=(`hr`i''), nformat(number_d2)
		quietly: putexcel F`irow'=(`ll`i''), nformat(number_d2)
		quietly: putexcel G`irow'=(`ul`i''), nformat(number_d2)
		quietly: putexcel B`irow'=("`idg_name'")
		quietly: putexcel C`irow'=("pooled")
		quietly: putexcel D`irow'=("Different micro-organisms, infection No. `i'")
tab multiinf, matcell(x), if _st==1 & inlist(multiinf,0,`i')
matrix list x
tab multiinf ensidementia, matcell(y), if _d==1 & inlist(multiinf,0,`i')
matrix list y
quietly: putexcel set "Pooled results.xlsm", sheet("FIGURE sensitivity dist") modify
		local j = `i' + 1
		quietly: putexcel M`irow'=(x[2,1]), nformat(number)
		quietly: putexcel N`irow'=(y[2,1]), nformat(number)
local ++irow
local ++irow
}
*P for trend
stcox multiinf 2.supu#cohort 2.ses#cohort 3.ses#cohort, strata(cohort)
quietly: matrix n = r(table)
matrix list n
```

```
tempname p
scalar `p' = n[4,1]
quietly: putexcel set "Pooled results.xlsm", sheet("FIGURE sensitivity dist") modify
        quietly: putexcel Q`prow'=(`p'), nformat(number)
restore


*Dementia occurring from year 10 onwards
local i 1
preserve
quietly: stset exitpvm, id(id) failure(ensidementia) origin(time syntpvm) enter(time
lag_entry_10y_bactviral) scale(365.25)
quietly: keep id anybactviral supu cohort exitpvm ensidementia syntpvm entrypvm ensi*pvm
*toinen* _st _d _origin _t _t0 ses anyanyinf
sum ensibactviralpvm toinenbactviralpvm
tab anybactviral if _st==1
tab toinenbactviral if _st==1

gen byte multiinf = 0 if anyanyinf==0
tab multiinf
tab multiinf if _st==1
replace multiinf = 1 if anybactviral==1
tab multiinf
tab multiinf if _st==1
replace multiinf = 2 if toinenbactviral==1
tab multiinf
tab multiinf if _st==1
*browse id ensibactviralpvm toinenbactviralpvm multiinf _t0 _t

quietly: stdescribe
quietly: local failures = `r(N_fail)'
stcox i.multiinf 2.supu#cohort 2.ses#cohort 3.ses#cohort, strata(cohort), if multiinf!=2
quietly: matrix m = r(table)
matrix list m
tempname hr1 ll1 ul1 hr2 ll2 ul2 N1 fail1 N2 fail2
scalar `hr1' = m[1,2]
scalar `ll1' = m[5,2]
scalar `ul1' = m[6,2]
scalar `N1' = e(N_sub)
scalar `fail1' = e(N_fail)
stcox i.multiinf 2.supu#cohort 2.ses#cohort 3.ses#cohort, strata(cohort), if multiinf!=1
quietly: matrix m = r(table)
matrix list m
scalar `hr2' = m[1,2]
scalar `ll2' = m[5,2]
scalar `ul2' = m[6,2]
scalar `N2' = e(N_sub)
scalar `fail2' = e(N_fail)

forvalues i =1/2 {
quietly: putexcel set "Pooled results.xlsm", sheet("FIGURE sensitivity dist") modify
        quietly: putexcel K`irow'=(`N`i''), nformat(number)
        quietly: putexcel L`irow'=(`fail`i''), nformat(number)
        quietly: putexcel E`irow'=(`hr`i''), nformat(number_d2)
        quietly: putexcel F`irow'=(`ll`i''), nformat(number_d2)
        quietly: putexcel G`irow'=(`ul`i''), nformat(number_d2)
        quietly: putexcel B`irow'=("`idg_name'")
        quietly: putexcel C`irow'=("pooled")
```

```
                    quietly: putexcel D`irow'=("Different micro-organisms after 10+ years, infection No. `i'")
            tab multiinf, matcell(x), if _st==1 & inlist(multiinf,0,`i')
            matrix list x
            tab multiinf ensidementia, matcell(y), if _d==1 & inlist(multiinf,0,`i')
            matrix list y
            quietly: putexcel set "Pooled results.xlsm", sheet("FIGURE sensitivity dist") modify
                    local j = `i' + 1
                    quietly: putexcel M`irow'=(x[2,1]), nformat(number)
                    quietly: putexcel N`irow'=(y[2,1]), nformat(number)
            local ++irow
            local ++irow
            }
            *P for trend
            stcox multiinf 2.supu#cohort 2.ses#cohort 3.ses#cohort, strata(cohort)
            quietly: matrix n = r(table)
            matrix list n
            tempname p
            scalar `p' = n[4,1]
            quietly: putexcel set "Pooled results.xlsm", sheet("FIGURE sensitivity dist") modify
                    quietly: putexcel Q`prow'=(`p'), nformat(number)
            restore


log close



***** NUMBER OF SIMULTANEOUS INFECTIONS *******

capture log close
log using "Number of infections.log", replace

local dg infcount
local n_dg: word count `dg'
local dg_name `""No. of infections""'

            quietly: use "Inf_dem_lag.dta", clear
            merge 1:1 id using "Infection count.dta"
            tab anyinfcount
            replace anyinfcount=0 if anyanyinf==0
            recode anyinfcount 3=2 4=2
            drop if ses==.
            gen ensiinfcountpvm = ensianyinfpvm

quietly: local irow = 23
quietly: local prow = `irow'
      forvalues i=1/`n_dg' {

            quietly: local idg : word `i' of `dg'
            quietly: local idg_name : word  `i' of `dg_name'
            display ""
            display "`idg' followed by broad dementia, adjusted for sex and education and stratified by cohort"
            display ""
            display "Pooled IPD-analysis, normal model and 10-year exclusion"
            display ""
            display "$S_TIME  $S_DATE"
```

```
display ""

preserve
quietly: stset exitpvm, id(id) failure(ensidementia) origin(time syntpvm) enter(time
lag_entry_anyinf) scale(365.25)
quietly: keep id any`idg' supu cohort exitpvm ensidementia syntpvm entrypvm ensi`idg'pvm  _st
_d _origin _t _t0 ses
tab any`idg' if _st==1

quietly: stdescribe
quietly: local failures = `r(N_fail)'
stcox i.any`idg' 2.supu#cohort 2.ses#cohort 3.ses#cohort, strata(cohort), if any`idg'!=2
quietly: matrix m = r(table)
matrix list m
tempname hr1 ll1 ul1 hr2 ll2 ul2 N1 fail1 N2 fail2
scalar `hr1' = m[1,2]
scalar `ll1' = m[5,2]
scalar `ul1' = m[6,2]
scalar `N1' = e(N_sub)
scalar `fail1' = e(N_fail)
stcox i.any`idg' 2.supu#cohort 2.ses#cohort 3.ses#cohort, strata(cohort), if any`idg'!=1
quietly: matrix m = r(table)
matrix list m
scalar `hr2' = m[1,2]
scalar `ll2' = m[5,2]
scalar `ul2' = m[6,2]
scalar `N2' = e(N_sub)
scalar `fail2' = e(N_fail)

forvalues i =1/2 {
quietly: putexcel set "Pooled results.xlsm", sheet("FIGURE sensitivity dist") modify
        quietly: putexcel K`irow'=(`N`i''), nformat(number)
        quietly: putexcel L`irow'=(`fail`i''), nformat(number)
        quietly: putexcel E`irow'=(`hr`i''), nformat(number_d2)
        quietly: putexcel F`irow'=(`ll`i''), nformat(number_d2)
        quietly: putexcel G`irow'=(`ul`i''), nformat(number_d2)
        quietly: putexcel B`irow'=("Simultaneous infection diagnoses")
        quietly: putexcel C`irow'=("pooled")
        quietly: putexcel D`irow'=("No. of simultaneous infection diagnoses = `i'")
tab any`idg', matcell(x), if _st==1 &  inlist(any`idg',0,`i')
matrix list x
tab any`idg' ensidementia, matcell(y), if _d==1 &  inlist(any`idg',0,`i')
matrix list y
quietly: putexcel set "Pooled results.xlsm", sheet("FIGURE sensitivity dist") modify
        quietly: putexcel M`irow'=(x[2,1]), nformat(number)
        quietly: putexcel N`irow'=(y[2,1]), nformat(number)
local ++irow
local ++irow
}

*P for trend
stcox any`idg' 2.supu#cohort 2.ses#cohort 3.ses#cohort, strata(cohort)
quietly: matrix n = r(table)
matrix list n
tempname p
scalar `p' = n[4,1]
quietly: putexcel set "Pooled results.xlsm", sheet("FIGURE sensitivity dist") modify
        quietly: putexcel Q`prow'=(`p'), nformat(number)
```

```
local irow 24
restore

*Dementia occurring from year 10 onwards
preserve
quietly: stset exitpvm, id(id) failure(ensidementia) origin(time syntpvm) enter(time lag_entry_10y_anyinf) scale(365.25)
quietly: keep id any`idg' supu cohort exitpvm ensidementia syntpvm entrypvm ensi`idg'pvm  _st _d _origin _t _t0 ses
tab any`idg' if _st==1

quietly: stdescribe
quietly: local failures = `r(N_fail)'
stcox i.any`idg' 2.supu#cohort 2.ses#cohort 3.ses#cohort, strata(cohort), if any`idg'!=2
quietly: matrix m = r(table)
matrix list m
tempname hr1 ll1 ul1 hr2 ll2 ul2 N1 fail1 N2 fail2
scalar `hr1' = m[1,2]
scalar `ll1' = m[5,2]
scalar `ul1' = m[6,2]
scalar `N1' = e(N_sub)
scalar `fail1' = e(N_fail)
stcox i.any`idg' 2.supu#cohort 2.ses#cohort 3.ses#cohort, strata(cohort), if any`idg'!=1
quietly: matrix m = r(table)
matrix list m
scalar `hr2' = m[1,2]
scalar `ll2' = m[5,2]
scalar `ul2' = m[6,2]
scalar `N2' = e(N_sub)
scalar `fail2' = e(N_fail)

forvalues i =1/2 {
quietly: putexcel set "Pooled results.xlsm", sheet("FIGURE sensitivity dist") modify
        quietly: putexcel K`irow'=(`N`i''), nformat(number)
        quietly: putexcel L`irow'=(`fail`i''), nformat(number)
        quietly: putexcel E`irow'=(`hr`i''), nformat(number_d2)
        quietly: putexcel F`irow'=(`ll`i''), nformat(number_d2)
        quietly: putexcel G`irow'=(`ul`i''), nformat(number_d2)
        quietly: putexcel B`irow'=("Simultaneous infection diagnoses")
        quietly: putexcel C`irow'=("pooled")
        quietly: putexcel D`irow'=("10-year lag, No. of simultaneous infection diagnoses = `i'")
tab any`idg', matcell(x), if _st==1 &  inlist(any`idg',0,`i')
matrix list x
tab any`idg' ensidementia, matcell(y), if _d==1 &  inlist(any`idg',0,`i')
matrix list y
quietly: putexcel set "Pooled results.xlsm", sheet("FIGURE sensitivity dist") modify
        quietly: putexcel M`irow'=(x[2,1]), nformat(number)
        quietly: putexcel N`irow'=(y[2,1]), nformat(number)
local ++irow
local ++irow
}

*P for trend
stcox any`idg' 2.supu#cohort 2.ses#cohort 3.ses#cohort, strata(cohort)
quietly: matrix n = r(table)
matrix list n
```

```
				tempname p
				scalar `p' = n[4,1]
				quietly: putexcel set "Pooled results.xlsm", sheet("FIGURE sensitivity dist") modify
						quietly: putexcel Q`prow'=(`p'), nformat(number)

				local irow 24
				restore
				}
log close


*FIGURE sensitivity, CNS vs other infections
capture log close
log using "Sensitivity, CNS infection vs other infection.log", replace
local dg anycns noncns
local n_dg: word count `dg'
local dg_name `""Any CNS infection vs not" "Any extra-CNS infection vs not""'

				quietly: use "Inf_dem_lag.dta", clear
				drop if ses==.

foreach idg of local dg {
		tab any`idg'
		replace any`idg' = . if any`idg'!=1 & anyanyinf==1
		tab any`idg'
		egen lag_entry_`idg' = rowmax(lag_entry_anyinf ensi`idg'pvm)
		gen ensi`idg'pvm_10y =  ensi`idg'pvm + round(10*365.25)
		egen lag_entry_10y_`idg' = rowmax(lag_entry_10y_anyinf ensi`idg'pvm_10y)
		}

quietly: local irow = 46
		forvalues i=1/`n_dg' {
				preserve
				quietly: local idg : word `i' of `dg'
				quietly: local idg_name : word  `i' of `dg_name'
				display ""
				display "`idg' followed by broad dementia, adjusted for sex and education and stratified by
cohort"
				display ""
				display "Pooled IPD-analysis, normal model and 10-year exclusion"
				display ""
				display "$S_TIME  $S_DATE"
				display ""

				quietly: stset exitpvm, id(id) failure(ensidementia) origin(time syntpvm) enter(time
lag_entry_`idg') scale(365.25)
				quietly: keep id any`idg' supu cohort exitpvm ensidementia syntpvm entrypvm ensi`idg'pvm  _st
_d _origin _t _t0 ses
				tab any`idg' if _st==1

				quietly: stdescribe
				quietly: local failures = `r(N_fail)'
				stcox any`idg' 2.supu#cohort 2.ses#cohort 3.ses#cohort, strata(cohort)
				quietly: matrix m = r(table)
				tempname hr ll ul
				scalar `hr' = m[1,1]
				scalar `ll' = m[5,1]
				scalar `ul' = m[6,1]
```

```
			quietly: putexcel set "Pooled results.xlsm", sheet("FIGURE sensitivity dist") modify
					quietly: putexcel K`irow'=(`e(N_sub)'), nformat(number)
					quietly: putexcel L`irow'=(`e(N_fail)'), nformat(number)
					quietly: putexcel E`irow'=(`hr'), nformat(number_d2)
					quietly: putexcel F`irow'=(`ll'), nformat(number_d2)
					quietly: putexcel G`irow'=(`ul'), nformat(number_d2)
					quietly: putexcel B`irow'=("`idg'")
					quietly: putexcel C`irow'=("pooled")
					quietly: putexcel D`irow'=("`idg_name'")
			tab any`idg', matcell(x), if _st==1
			matrix list x
			quietly: tab any`idg' ensidementia, matcell(y), if _d==1
			matrix list y
			quietly: putexcel set "Pooled results.xlsm", sheet("FIGURE sensitivity dist") modify
					quietly: putexcel M`irow'=(x[2,1]), nformat(number)
					quietly: putexcel N`irow'=(y[2,1]), nformat(number)
			local ++irow
			restore

			*Dementia occurring from year 10 onwards
			preserve
			quietly: stset exitpvm, id(id) failure(ensidementia) origin(time syntpvm) enter(time
lag_entry_10y_`idg') scale(365.25)
			quietly: stdescribe
			quietly: local failures = `r(N_fail)'
			stcox any`idg' 2.supu#cohort 2.ses#cohort 3.ses#cohort, strata(cohort)
			quietly: matrix m = r(table)
			scalar `hr' = m[1,1]
			scalar `ll' = m[5,1]
			scalar `ul' = m[6,1]
			quietly: putexcel set "Pooled results.xlsm", sheet("FIGURE sensitivity dist") modify
					quietly: putexcel K`irow'=(`e(N_sub)'), nformat(number)
					quietly: putexcel L`irow'=(`e(N_fail)'), nformat(number)
					quietly: putexcel E`irow'=(`hr'), nformat(number_d2)
					quietly: putexcel F`irow'=(`ll'), nformat(number_d2)
					quietly: putexcel G`irow'=(`ul'), nformat(number_d2)
					quietly: putexcel B`irow'=("   `idg'_10y_exclusion")
					quietly: putexcel C`irow'=("pooled")
					quietly: putexcel D`irow'=("   Dementia occurring more than 10 years after the
hospitalisation")
			tab any`idg', matcell(x), if _st==1
			matrix list x
			quietly: tab any`idg' ensidementia, matcell(y), if _d==1
			matrix list y
			quietly: putexcel set "Pooled results.xlsm", sheet("FIGURE sensitivity dist") modify
					quietly: putexcel M`irow'=(x[2,1]), nformat(number)
					quietly: putexcel N`irow'=(y[2,1]), nformat(number)
			local ++irow
			local ++irow
			restore
			}
log close


*FIGURE sensitivity, infections predisposed towards entering the CNS vs other infections
capture log close
log using "Sensitivity, predisposed towards CNS vs others.log", replace
local dg anycnspredisp noncnspredisp
```

```
local n_dg: word count `dg'
local dg_name `""Infection predisposed towards entering the CNS vs no infection" "Infection not predisposed towards entering the CNS vs no infection""'

				quietly: use "Inf_dem_lag.dta", clear
				drop if ses==.

				foreach idg of local dg {
						tab any`idg'
						replace any`idg' = . if any`idg'!=1 & anyanyinf==1
						tab any`idg'
						egen lag_entry_`idg' = rowmax(lag_entry_anyinf ensi`idg'pvm)
						gen ensi`idg'pvm_10y =  ensi`idg'pvm + round(10*365.25)
						egen lag_entry_10y_`idg' = rowmax(lag_entry_10y_anyinf ensi`idg'pvm_10y)
						}

quietly: local irow = 76
		forvalues i=1/`n_dg' {
				preserve
				quietly: local idg : word `i' of `dg'
				quietly: local idg_name : word  `i' of `dg_name'
				display ""
				display "`idg' followed by broad dementia, adjusted for sex and education and stratified by cohort"
				display ""
				display "Pooled IPD-analysis, normal model and 10-year exclusion"
				display ""
				display "$S_TIME  $S_DATE"
				display ""

				quietly: stset exitpvm, id(id) failure(ensidementia) origin(time syntpvm) enter(time lag_entry_`idg') scale(365.25)
				quietly: keep id any`idg' supu cohort exitpvm ensidementia syntpvm entrypvm ensi`idg'pvm  _st _d _origin _t _t0 ses
				tab any`idg' if _st==1

				quietly: stdescribe
				quietly: local failures = `r(N_fail)'
				stcox any`idg' 2.supu#cohort 2.ses#cohort 3.ses#cohort, strata(cohort)
				quietly: matrix m = r(table)
				tempname hr ll ul
				scalar `hr' = m[1,1]
				scalar `ll' = m[5,1]
				scalar `ul' = m[6,1]
				quietly: putexcel set "Pooled results.xlsm", sheet("FIGURE sensitivity dist") modify
						quietly: putexcel K`irow'=(`e(N_sub)'), nformat(number)
						quietly: putexcel L`irow'=(`e(N_fail)'), nformat(number)
						quietly: putexcel E`irow'=(`hr'), nformat(number_d2)
						quietly: putexcel F`irow'=(`ll'), nformat(number_d2)
						quietly: putexcel G`irow'=(`ul'), nformat(number_d2)
						quietly: putexcel B`irow'=("`idg'")
						quietly: putexcel C`irow'=("pooled")
						quietly: putexcel D`irow'=("`idg_name'")
				tab any`idg', matcell(x), if _st==1
				matrix list x
				quietly: tab any`idg' ensidementia, matcell(y), if _d==1
				matrix list y
				quietly: putexcel set "Pooled results.xlsm", sheet("FIGURE sensitivity dist") modify
```

```
					quietly: putexcel M`irow'=(x[2,1]), nformat(number)
					quietly: putexcel N`irow'=(y[2,1]), nformat(number)
				local ++irow
				restore

				*Dementia occurring from year 10 onwards
				preserve
				quietly: stset exitpvm, id(id) failure(ensidementia) origin(time syntpvm) enter(time
lag_entry_10y_`idg') scale(365.25)
				quietly: stdescribe
				quietly: local failures = `r(N_fail)'
				stcox any`idg' 2.supu#cohort 2.ses#cohort 3.ses#cohort, strata(cohort)
				quietly: matrix m = r(table)
				scalar `hr' = m[1,1]
				scalar `ll' = m[5,1]
				scalar `ul' = m[6,1]
				quietly: putexcel set "Pooled results.xlsm", sheet("FIGURE sensitivity dist") modify
					quietly: putexcel K`irow'=(`e(N_sub)'), nformat(number)
					quietly: putexcel L`irow'=(`e(N_fail)'), nformat(number)
					quietly: putexcel E`irow'=(`hr'), nformat(number_d2)
					quietly: putexcel F`irow'=(`ll'), nformat(number_d2)
					quietly: putexcel G`irow'=(`ul'), nformat(number_d2)
					quietly: putexcel B`irow'=("   `idg'_10y_exclusion")
					quietly: putexcel C`irow'=("pooled")
					quietly: putexcel D`irow'=("   Dementia occurring more than 10 years after the
hospitalisation")
				tab any`idg', matcell(x), if _st==1
				matrix list x
				quietly: tab any`idg' ensidementia, matcell(y), if _d==1
				matrix list y
				quietly: putexcel set "Pooled results.xlsm", sheet("FIGURE sensitivity dist") modify
					quietly: putexcel M`irow'=(x[2,1]), nformat(number)
					quietly: putexcel N`irow'=(y[2,1]), nformat(number)
				local ++irow
				local ++irow
				restore
				}
log close


*TYPE OF HERPESVIRUS

capture log close
log using "FIGURE herpestype.log", replace
local dg hsv12 nonhsv mildherpes
local n_dg: word count `dg'
local dg_name `""Herpes simplex virus 1 and 2 infection" "Other herpesvirusinfection" "Mild herpesvirusinfection""'
quietly: local irow = 7

				quietly: use "Inf_dem_lag.dta", clear
				merge 1:1 id using "herpeskoodit.dta"
				drop _merge
				drop if ses==.
				tab anyhsv12
				gen anynonhsv = 0
				replace anynonhsv = 1 if anyherpes==1 & anyhsv12!=1

		forvalues i=1/`n_dg' {
```

```
preserve
quietly: local idg : word `i' of `dg'
quietly: local idg_name : word  `i' of `dg_name'
display ""
display "`idg' followed by broad dementia, adjusted for sex and education and stratified by
cohort"
display ""
display "Pooled IPD-analysis, normal model and 10-year exclusion"
display ""
display "$S_TIME  $S_DATE"
display ""

capture drop ensi`idg'pvm
gen ensi`idg'pvm = ensiherpespvm if any`idg'==1

tab any`idg'
replace any`idg' = 0 if anyanyinf==0
replace any`idg' = . if any`idg'!=1 & anyanyinf==1
tab any`idg'
egen lag_entry_`idg' = rowmax(lag_entry_anyinf ensi`idg'pvm)
gen ensi`idg'pvm_10y =  ensi`idg'pvm + round(10*365.25)
egen lag_entry_10y_`idg' = rowmax(lag_entry_10y_anyinf ensi`idg'pvm_10y)

quietly: stset exitpvm, id(id) failure(ensidementia) origin(time syntpvm) enter(time
lag_entry_`idg') scale(365.25)
quietly: keep id any`idg' supu cohort exitpvm ensidementia syntpvm entrypvm ensi`idg'pvm  _st
_d _origin _t _t0 ses

tab any`idg' if _st==1

quietly: stdescribe
quietly: local failures = `r(N_fail)'
stcox any`idg' 2.supu#cohort 2.ses#cohort 3.ses#cohort, strata(cohort)
quietly: matrix m = r(table)
tempname hr ll ul
scalar `hr' = m[1,1]
scalar `ll' = m[5,1]
scalar `ul' = m[6,1]
quietly: putexcel set "Pooled results.xlsm", sheet("FIGURE herpes sensitivity") modify
        quietly: putexcel K`irow'=(`e(N_sub)'), nformat(number)
        quietly: putexcel L`irow'=(`e(N_fail)'), nformat(number)
        quietly: putexcel E`irow'=(`hr'), nformat(number_d2)
        quietly: putexcel F`irow'=(`ll'), nformat(number_d2)
        quietly: putexcel G`irow'=(`ul'), nformat(number_d2)
        quietly: putexcel B`irow'=("`idg'")
        quietly: putexcel C`irow'=("pooled")
        quietly: putexcel D`irow'=("`idg_name'")
tab any`idg', matcell(x), if _st==1
matrix list x
quietly: tab any`idg' ensidementia, matcell(y), if _d==1
matrix list y
quietly: putexcel set "Pooled results.xlsm", sheet("FIGURE herpes sensitivity") modify
        quietly: putexcel M`irow'=(x[2,1]), nformat(number)
        quietly: putexcel N`irow'=(y[2,1]), nformat(number)
local ++irow

restore
}
```

```
		log close


*MOST COMMON GRAM-POSITIVE INFECTIONS

capture log close
log using "FIGURE gramplus.log", replace
local dg streptoc cdiffic othergramplus
local n_dg: word count `dg'
local dg_name `""Streptococcal infection" "Enterocolitis due to Clostridium difficile" "Other Gram-positive infection""'
quietly: local irow = 7

				quietly: use "Inf_dem_lag.dta", clear
				merge 1:1 id using "grampluskoodit.dta"
				drop _merge
				drop if ses==.

		forvalues i=1/`n_dg' {
				preserve
				quietly: local idg : word `i' of `dg'
				quietly: local idg_name : word  `i' of `dg_name'
				display ""
				display "`idg' followed by broad dementia, adjusted for sex and education and stratified by
cohort"
				display ""
				display "Pooled IPD-analysis, normal model and 10-year exclusion"
				display ""
				display "$S_TIME  $S_DATE"
				display ""

				recode any`idg' 0=.
				recode any`idg' .=0 if anyanyinf==0
				gen ensi`idg'pvm = ensigrampluspvm if any`idg'==1
				egen lag_entry_`idg' = rowmax(lag_entry_anyinf ensi`idg'pvm)

				quietly: stset exitpvm, id(id) failure(ensidementia) origin(time syntpvm) enter(time
lag_entry_`idg') scale(365.25)
				quietly: keep id any`idg' supu cohort exitpvm ensidementia syntpvm entrypvm ensi`idg'pvm  _st
_d _origin _t _t0 ses
				tab any`idg' if _st==1

				quietly: stdescribe
				quietly: local failures = `r(N_fail)'
				stcox any`idg' 2.supu#cohort 2.ses#cohort 3.ses#cohort, strata(cohort)
				quietly: matrix m = r(table)
				tempname hr ll ul
				scalar `hr' = m[1,1]
				scalar `ll' = m[5,1]
				scalar `ul' = m[6,1]
				quietly: putexcel set "Pooled results.xlsm", sheet("FIGURE gramplus") modify
						quietly: putexcel K`irow'=(`e(N_sub)'), nformat(number)
						quietly: putexcel L`irow'=(`e(N_fail)'), nformat(number)
						quietly: putexcel E`irow'=(`hr'), nformat(number_d2)
						quietly: putexcel F`irow'=(`ll'), nformat(number_d2)
						quietly: putexcel G`irow'=(`ul'), nformat(number_d2)
						quietly: putexcel B`irow'=("`idg'")
						quietly: putexcel C`irow'=("pooled")
						quietly: putexcel D`irow'=("`idg_name'")
```

```
tab any`idg', matcell(x), if _st==1
matrix list x
quietly: tab any`idg' ensidementia, matcell(y), if _d==1
matrix list y
quietly: putexcel set "Pooled results.xlsm", sheet("FIGURE gramplus") modify
        quietly: putexcel M`irow'=(x[2,1]), nformat(number)
        quietly: putexcel N`irow'=(y[2,1]), nformat(number)
local ++irow

restore
}

log close

*MOST COMMON GRAM-NEGATIVE INFECTIONS

capture log close
log using "FIGURE gramminus.log", replace
local dg salmonella lyme othergramminus
local n_dg: word count `dg'
local dg_name `""Salmonella infection" "Lyme disease" "Other Gram-negative infection""'
quietly: local irow = 7

quietly: use "Inf_dem_lag.dta", clear
merge 1:1 id using "gramminuskoodit.dta"
tab anygramminus
tab anysalmonella
tab anylyme
tab anyothergramminus
tab anyothergramminussepsis
replace anyothergramminus = 1 if anyothergramminussepsis==1
tab anyothergramminus
drop _merge
drop if ses==.

forvalues i=1/`n_dg' {
preserve
quietly: local idg : word `i' of `dg'
quietly: local idg_name : word  `i' of `dg_name'
display ""
display "`idg' followed by broad dementia, adjusted for sex and education and stratified by
cohort"
display ""
display "Pooled IPD-analysis, normal model and 10-year exclusion"
display ""
display "$S_TIME  $S_DATE"
display ""

recode any`idg' 0=.
recode any`idg' .=0 if anyanyinf==0
gen ensi`idg'pvm = ensigramminuspvm if any`idg'==1
egen lag_entry_`idg' = rowmax(lag_entry_anyinf ensi`idg'pvm)

quietly: stset exitpvm, id(id) failure(ensidementia) origin(time syntpvm) enter(time
lag_entry_`idg') scale(365.25)
quietly: keep id any`idg' supu cohort exitpvm ensidementia syntpvm entrypvm ensi`idg'pvm  _st
_d _origin _t _t0 ses
tab any`idg' if _st==1
```

```
			quietly: stdescribe
			quietly: local failures = `r(N_fail)'
			stcox any`idg' 2.supu#cohort 2.ses#cohort 3.ses#cohort, strata(cohort)
			quietly: matrix m = r(table)
			tempname hr ll ul
			scalar `hr' = m[1,1]
			scalar `ll' = m[5,1]
			scalar `ul' = m[6,1]
			quietly: putexcel set "Pooled results.xlsm", sheet("FIGURE gramminus") modify
					quietly: putexcel K`irow'=(`e(N_sub)'), nformat(number)
					quietly: putexcel L`irow'=(`e(N_fail)'), nformat(number)
					quietly: putexcel E`irow'=(`hr'), nformat(number_d2)
					quietly: putexcel F`irow'=(`ll'), nformat(number_d2)
					quietly: putexcel G`irow'=(`ul'), nformat(number_d2)
					quietly: putexcel B`irow'=("`idg'")
					quietly: putexcel C`irow'=("pooled")
					quietly: putexcel D`irow'=("`idg_name'")
			tab any`idg', matcell(x), if _st==1
			matrix list x
			quietly: tab any`idg' ensidementia, matcell(y), if _d==1
			matrix list y
			quietly: putexcel set "Pooled results.xlsm", sheet("FIGURE gramminus") modify
					quietly: putexcel M`irow'=(x[2,1]), nformat(number)
					quietly: putexcel N`irow'=(y[2,1]), nformat(number)
			local ++irow

			restore
			}

	log close



*** FINE AND GRAY MODEL ***

capture log close
log using "FIGURE fine gray.log", replace
local dg anyinf bactinf viralinf
local n_dg: word count `dg'
local dg_name `""Any infectious disease" "Any bacterial infection" "Any viral infection""'
quietly: local irow = 7

			quietly: use "Inf_dem_lag.dta", clear
			drop if ses==.

			stset exitpvm, id(id) failure(ensidementia) origin(time syntpvm) enter(time lag_entry_anyinf) 
scale(365.25)
			tab ensidementia
			*Coding of detailedfail: 1 early-onset dementia, 2 late-onset dementia, 3 death
			gen byte detailedfail = 1 if _d==1 & _t<65
			replace detailedfail = 2 if _d==1 & _t>=65
			replace detailedfail = 3 if failure==2
			tab ensidementia
			tab failure
			tab detailedfail

	forvalues i=1/`n_dg' {
```

```
				quietly: local idg : word `i' of `dg'
				quietly: local idg_name : word  `i' of `dg_name'
				display ""
				display "`idg' followed by broad dementia, adjusted for sex and education and stratified by
cohort"
				display ""
				display "Pooled IPD-analysis, normal model and 10-year exclusion"
				display ""
				display "$S_TIME  $S_DATE"
				display ""

				forvalues j=1/3 {
				preserve
				if `j'==1 | `j'== 2 {
				stset exitpvm, id(id) failure(ensidementia) origin(time syntpvm) enter(time lag_entry_`idg')
scale(365.25)
				}
				else if `j'== 3 {
				stset exitpvm, id(id) failure(detailedfail==2) origin(time syntpvm) enter(time lag_entry_`idg')
scale(365.25)
				}

				quietly: keep id any`idg' supu cohort exitpvm ensidementia syntpvm entrypvm ensi`idg'pvm  _st
_d _origin _t _t0 ses detailedfail lag_entry_`idg'
				tab any`idg' if _st==1

				quietly: stdescribe
				quietly: local failures = `r(N_fail)'

				if `j'==1 {
				stcox any`idg' 2.supu#cohort 2.ses#cohort 3.ses#cohort, strata(cohort)
				}
				else if `j'== 2 {
				stcrreg any`idg' 2.supu#cohort 2.ses#cohort 3.ses#cohort i.cohort, cl(cohort)
compete(detailedfail==3)
				}
				else if `j'== 3 {
				stcrreg any`idg' 2.supu#cohort 2.ses#cohort 3.ses#cohort i.cohort, cl(cohort)
compete(detailedfail==1 3)
				}

				quietly: matrix m = r(table)
				tempname hr ll ul
				scalar `hr' = m[1,1]
				scalar `ll' = m[5,1]
				scalar `ul' = m[6,1]
				quietly: putexcel set "Pooled results.xlsm", sheet("FIGURE fine gray (appendix)") modify
					quietly: putexcel K`irow'=(`e(N_sub)'), nformat(number)
					quietly: putexcel L`irow'=(`e(N_fail)'), nformat(number)
					quietly: putexcel E`irow'=(`hr'), nformat(number_d2)
					quietly: putexcel F`irow'=(`ll'), nformat(number_d2)
					quietly: putexcel G`irow'=(`ul'), nformat(number_d2)
					quietly: putexcel B`irow'=("`idg'")
					quietly: putexcel C`irow'=("pooled")
					if `j'==1 {
					quietly: putexcel D`irow'=("`idg_name', Cox model")
					}
					else if `j'== 2 {
```

```
					quietly: putexcel D`irow'=("`idg_name', Fine & Gray model, competing outcome
death")
					}
					else if `j'== 3 {
					quietly: putexcel D`irow'=("`idg_name', Fine & Gray model, competing outcome
death + early-onset dementia")
					}
				tab any`idg', matcell(x), if _st==1
				matrix list x
				quietly: tab any`idg' _d, matcell(y), if _d==1
				matrix list y
				quietly: putexcel set "Pooled results.xlsm", sheet("FIGURE fine gray (appendix)") modify
					quietly: putexcel M`irow'=(x[2,1]), nformat(number)
					quietly: putexcel N`irow'=(y[2,1]), nformat(number)
				local ++irow
				restore
				}
				local ++irow
				}
		log close



*** TIME INTERACTION ***

capture log close
log using "Time interaction.log", replace

				quietly: use "Inf_dem_lag.dta", clear
				drop if ses==.

local dg anyinf bactinf viralinf
local n_dg: word count `dg'
local dg_name `""Any infectious disease" "Bacterial infection" "Viral infection""'
quietly: local irow = 7
		forvalues i=1/`n_dg' {
				preserve
				quietly: local idg : word `i' of `dg'
				quietly: local idg_name : word  `i' of `dg_name'
				display ""
				display "`idg' followed by broad dementia, adjusted for sex and education and stratified by
cohort"
				display ""
				display "Pooled IPD-analysis, normal model and 10-year exclusion"
				display ""
				display "$S_TIME  $S_DATE"
				display ""

				quietly: keep id any`idg' supu cohort exitpvm ensidementia syntpvm entrypvm ensi`idg'pvm ses
lag_entry_`idg'
				stset exitpvm, id(id) failure(ensidementia) origin(time lag_entry_`idg') scale(365.25)
				gen age`idg' = (lag_entry_`idg'-syntpvm)/365.25
				gen age`idg'2 = age`idg'^2

				tab any`idg' if _st==1

				quietly: stdescribe
				quietly: local failures = `r(N_fail)'
```

```
				stcox any`idg' 2.supu#cohort 2.ses#cohort 3.ses#cohort c.age`idg'#cohort c.age`idg'2#cohort,
strata(cohort)
				matrix m = r(table)
				matrix list m
				stcox any`idg' 2.supu#cohort 2.ses#cohort 3.ses#cohort c.age`idg'#cohort c.age`idg'2#cohort,
strata(cohort) tvc(any`idg') texp(ln(_t))
				matrix m2 = r(table)
				matrix list m2
				restore
				}
log close

version 16

				use "Inf_dem_lag.dta", replace


*** eTABLE 4 ****
*(was previously TABLE 2) *

capture log close
log using "TABLE2.log", replace

use "Inf_dem_lag.dta", clear
quietly: drop if ses==.
quietly: gen hypertensio_combpvm = hypert_combpvm
merge 1:1 id using "Parkinson.dta"
keep if _merge==3
drop _merge

local dg anyinf
local eks `""no one" "hypertensio" "diabetes" "ihd" "cereb" "parkinson"'
local n_dg: word count `dg'
local n_eks: word count `eks'
local dg_name `""Any infectious disease vs no infection""'
local eks_name `""no one" "hypertension" "diabetes" "ischaemic heart disease" "cerebrovascular disease" "Parkinson´s disease"'
quietly: local irow = 8
		forvalues i=1/`n_dg' {
		forvalues j=1/`n_eks' {
				preserve
				quietly: local idg : word `i' of `dg'
				quietly: local idg_name : word  `i' of `dg_name'
				quietly: local ieks : word `j' of `eks'
				quietly: local ieks_name : word  `j' of `eks_name'
				display ""
				display "`idg' followed by broad dementia, adjusted for sex and education and stratified by
cohort"
				display ""
				display "Pooled IPD-analysis, normal model"
				display ""
				display "$S_TIME  $S_DATE"
				display ""

				if inlist("`ieks'","hypertensio", "diabetes", "ihd", "cereb", "parkinson") {
				drop if `ieks'_combpvm <= lag_entry_anyinf
				}
```

```
				stset exitpvm, id(id) failure(ensidementia) origin(time syntpvm) enter(time lag_entry_anyinf) 
scale(365.25)
				keep if _st==1

				if "`ieks'"=="hypertensio" & "`idg'"=="anyinf" {
				local irow = 25
				}

				quietly: capture keep id any`idg' supu cohort exitpvm ensidementia syntpvm entrypvm 
ensi`idg'pvm  _st _d _origin _t _t0 ses `ieks'_comb
				tab any`idg'

				quietly: stdescribe
				quietly: local failures = `r(N_fail)'
				stcox any`idg' 2.supu#cohort 2.ses#cohort 3.ses#cohort, strata(cohort)
				quietly: matrix m = r(table)
				tempname hr ll ul
				scalar `hr' = m[1,1]
				scalar `ll' = m[5,1]
				scalar `ul' = m[6,1]
				quietly: putexcel set "Pooled results.xlsm", sheet("TABLE2") modify
						quietly: putexcel K`irow'=(`e(N_sub)'), nformat(number)
						quietly: putexcel L`irow'=(`e(N_fail)'), nformat(number)
						quietly: putexcel E`irow'=(`hr'), nformat(number_d2)
						quietly: putexcel F`irow'=(`ll'), nformat(number_d2)
						quietly: putexcel G`irow'=(`ul'), nformat(number_d2)
						quietly: putexcel B`irow'=("`idg'")
						quietly: putexcel C`irow'=("pooled")
						quietly: putexcel D`irow'=("Those with `ieks_name' excluded")
				tab any`idg', matcell(x), if _st==1
				matrix list x
				quietly: tab any`idg' _d, matcell(y), if _d==1
				matrix list y
				quietly: putexcel set "Pooled results.xlsm", sheet("TABLE2") modify
						quietly: putexcel M`irow'=(x[2,1]), nformat(number)
						quietly: putexcel N`irow'=(y[2,1]), nformat(number)
				local ++irow
				restore
}
}

*Early- vs late-onset dementia (Gram-negative)
				use "Inf_dem_lag.dta", clear
				drop if ses==.

				quietly: stset exitpvm, id(id) failure(ensidementia) origin(time syntpvm) enter(time 
lag_entry_anyinf) scale(365.25)
				keep if _st==1
				local idg anyinf
				quietly: capture keep id any`idg' supu cohort exitpvm ensidementia syntpvm entrypvm 
ensi`idg'pvm  _st _d _origin _t _t0 ses
				tab any`idg'

				stsplit age65, at(64.999)
				recode age65 64.999=1

				preserve
				keep if age65==0
```

```
local irow 12
quietly: stdescribe
quietly: local failures = `r(N_fail)'
stcox any`idg' 2.supu#cohort 2.ses#cohort 3.ses#cohort, strata(cohort)
quietly: matrix m = r(table)
tempname hr ll ul
scalar `hr' = m[1,1]
scalar `ll' = m[5,1]
scalar `ul' = m[6,1]
quietly: putexcel set "Pooled results.xlsm", sheet("TABLE2") modify
		quietly: putexcel K`irow'=(`e(N_sub)'), nformat(number)
		quietly: putexcel L`irow'=(`e(N_fail)'), nformat(number)
		quietly: putexcel E`irow'=(`hr'), nformat(number_d2)
		quietly: putexcel F`irow'=(`ll'), nformat(number_d2)
		quietly: putexcel G`irow'=(`ul'), nformat(number_d2)
		quietly: putexcel B`irow'=("`idg'")
		quietly: putexcel C`irow'=("pooled")
		quietly: putexcel D`irow'=("Early-onset dementia (onset before age 65)")
tab any`idg', matcell(x), if _st==1
matrix list x
quietly: tab any`idg' ensidementia, matcell(y), if _d==1
matrix list y
quietly: putexcel set "Pooled results.xlsm", sheet("TABLE2") modify
		quietly: putexcel M`irow'=(x[2,1]), nformat(number)
		quietly: putexcel N`irow'=(y[2,1]), nformat(number)
restore

preserve
keep if age65==1
local irow 13
quietly: stdescribe
quietly: local failures = `r(N_fail)'
stcox any`idg' 2.supu#cohort 2.ses#cohort 3.ses#cohort, strata(cohort)
quietly: matrix m = r(table)
tempname hr ll ul
scalar `hr' = m[1,1]
scalar `ll' = m[5,1]
scalar `ul' = m[6,1]
quietly: putexcel set "Pooled results.xlsm", sheet("TABLE2") modify
		quietly: putexcel K`irow'=(`e(N_sub)'), nformat(number)
		quietly: putexcel L`irow'=(`e(N_fail)'), nformat(number)
		quietly: putexcel E`irow'=(`hr'), nformat(number_d2)
		quietly: putexcel F`irow'=(`ll'), nformat(number_d2)
		quietly: putexcel G`irow'=(`ul'), nformat(number_d2)
		quietly: putexcel B`irow'=("`idg'")
		quietly: putexcel C`irow'=("pooled")
		quietly: putexcel D`irow'=("Late-onset dementia (onset at or after age 65)")
tab any`idg', matcell(x), if _st==1
matrix list x
quietly: tab any`idg' ensidementia, matcell(y), if _d==1
matrix list y
quietly: putexcel set "Pooled results.xlsm", sheet("TABLE2") modify
		quietly: putexcel M`irow'=(x[2,1]), nformat(number)
		quietly: putexcel N`irow'=(y[2,1]), nformat(number)
restore
```

```
*Type of dementia
```

```
use "Inf_dem_lag.dta", clear
tab dementiatyyppi_laaja anyanyinf if ensidementia==1, col chi
local irow 15
local j 1
foreach type in AD FTD PD VD other unspecified  {
preserve
encode dementiatyyppi_laaja, gen(dementiatyyppi_laaja_nro)

display ""
display "`type'"
display ""
local idg gramminus
drop if ses==.

stset exitpvm, id(id) failure(dementiatyyppi_laaja_nro==`j') origin(time syntpvm) enter(time
lag_entry_anyinf) scale(365.25)
keep if _st==1

local idg anyinf
quietly: capture keep id any`idg' supu cohort exitpvm ensidementia syntpvm entrypvm
ensi`idg'pvm  _st _d _origin _t _t0 ses
tab any`idg'

quietly: stdescribe
quietly: local failures = `r(N_fail)'
stcox any`idg' 2.supu#cohort 2.ses#cohort 3.ses#cohort, strata(cohort)
quietly: matrix m = r(table)
tempname hr ll ul
scalar `hr' = m[1,1]
scalar `ll' = m[5,1]
scalar `ul' = m[6,1]
quietly: putexcel set "Pooled results.xlsm", sheet("TABLE2") modify
quietly: putexcel K`irow'=(`e(N_sub)'), nformat(number)
quietly: putexcel L`irow'=(`e(N_fail)'), nformat(number)
quietly: putexcel E`irow'=(`hr'), nformat(number_d2)
quietly: putexcel F`irow'=(`ll'), nformat(number_d2)
quietly: putexcel G`irow'=(`ul'), nformat(number_d2)
quietly: putexcel B`irow'=("`idg'")
quietly: putexcel C`irow'=("pooled")
quietly: putexcel D`irow'=("`type'")
tab any`idg', matcell(x), if _st==1
matrix list x
quietly: tab any`idg' _d, matcell(y), if _d==1
matrix list y
quietly: putexcel set "Pooled results.xlsm", sheet("TABLE2") modify
quietly: putexcel M`irow'=(x[2,1]), nformat(number)
quietly: putexcel N`irow'=(y[2,1]), nformat(number)
local ++irow
local ++j
restore
}

*Adjustments

local dg anyinf
local eks alcocl tupakka
local n_dg: word count `dg'
local n_eks: word count `eks'
```

```
local dg_name `""Any infectious disease""'
local eks_name `""heavy drinking" "smoking""'
quietly: local irow = 31
		forvalues i=1/`n_dg' {
		forvalues j=1/`n_eks' {
				quietly: local idg : word `i' of `dg'
				quietly: local idg_name : word  `i' of `dg_name'
				quietly: local ieks : word `j' of `eks'
				quietly: local ieks_name : word  `j' of `eks_name'
				display ""
				display `"`idg' followed by broad dementia, adjusted for sex and education and `eks_name' and
stratified by cohort"'
				display ""
				display "Pooled IPD-analysis, normal model"
				display ""
				display "$S_TIME  $S_DATE"
				display ""

				use "Inf_dem_lag.dta", clear
				drop if ses==.

				if "`ieks'"=="alcocl" {
				replace lag_entry_anyinf = alkopvm if cohort==1 & alkopvm>=lag_entry_anyinf // 1 = FPS, in
FPS, the questionnaire data was sometimes collected only after study entry. To avoid immortal time bias, the follow up is started
only after the date of the questionnaire in the analyses using information from that questionnaire.
				tab `ieks'
				drop if `ieks' ==.
				}
				if "`ieks'"=="tupakka" {
				replace lag_entry_anyinf = tupakkapvm if cohort==1 & tupakkapvm>=lag_entry_anyinf  // 1 =
FPS, in FPS, the questionnaire data was sometimes collected only after study entry. To avoid immortal time bias, the follow up is
started only after the date of the questionnaire in the analyses using information from that questionnaire.
				tab `ieks'
				drop if `ieks' ==.
				}

				quietly: stset exitpvm, id(id) failure(ensidementia) origin(time syntpvm) enter(time
lag_entry_anyinf) scale(365.25)
				keep if _st==1
				quietly: capture keep id any`idg' supu cohort exitpvm ensidementia syntpvm lag_entry_anyinf
ensi`idg'pvm  _st _d _origin _t _t0 ses `ieks'_comb
				tab any`idg'

				*Those with data on the covariate available but without adjustment
				quietly: stdescribe
				quietly: local failures = `r(N_fail)'
				stcox any`idg' 2.supu#cohort 2.ses#cohort 3.ses#cohort, strata(cohort)
				quietly: matrix m = r(table)
				tempname hr ll ul
				scalar `hr' = m[1,1]
				scalar `ll' = m[5,1]
				scalar `ul' = m[6,1]
				quietly: putexcel set "Pooled results.xlsm", sheet("TABLE2") modify
						quietly: putexcel K`irow'=(`e(N_sub)'), nformat(number)
						quietly: putexcel L`irow'=(`e(N_fail)'), nformat(number)
						quietly: putexcel E`irow'=(`hr'), nformat(number_d2)
						quietly: putexcel F`irow'=(`ll'), nformat(number_d2)
						quietly: putexcel G`irow'=(`ul'), nformat(number_d2)
```

```
					quietly: putexcel B`irow'=("`idg'")
					quietly: putexcel C`irow'=("pooled")
					quietly: putexcel D`irow'=("Data available for `ieks_name'")
			tab any`idg', matcell(x), if _st==1
			matrix list x
			quietly: tab any`idg' ensidementia, matcell(y), if _d==1
			matrix list y
			quietly: putexcel set "Pooled results.xlsm", sheet("TABLE2") modify
					quietly: putexcel M`irow'=(x[2,1]), nformat(number)
					quietly: putexcel N`irow'=(y[2,1]), nformat(number)
			local ++irow

			*Those with data on the covariate available and adjusted
			quietly: stdescribe
			quietly: local failures = `r(N_fail)'
			if "`ieks'" == "alcocl" {
			stcox any`idg' 2.supu#cohort 2.ses#cohort 3.ses#cohort  1.`ieks'#cohort 2.`ieks'#cohort
3.`ieks'#cohort, strata(cohort)
			}
			else if "`ieks'" == "tupakka" {
			stcox any`idg' 2.supu#cohort 2.ses#cohort 3.ses#cohort  2.`ieks'#cohort 3.`ieks'#cohort,
strata(cohort)
			}
			else if "`ieks'" == "bmi_who" {
			stcox any`idg' 2.supu#cohort 2.ses#cohort 3.ses#cohort  25.`ieks'#cohort 30.`ieks'#cohort,
strata(cohort)
			}
			else if "`ieks'" == "bmi" {
			stcox any`idg' 2.supu#cohort 2.ses#cohort 3.ses#cohort  c.`ieks'#cohort c.`ieks'2#cohort,
strata(cohort)
			}
			quietly: matrix m = r(table)
			tempname hr ll ul
			scalar `hr' = m[1,1]
			scalar `ll' = m[5,1]
			scalar `ul' = m[6,1]
			quietly: putexcel set "Pooled results.xlsm", sheet("TABLE2") modify
					quietly: putexcel K`irow'=(`e(N_sub)'), nformat(number)
					quietly: putexcel L`irow'=(`e(N_fail)'), nformat(number)
					quietly: putexcel E`irow'=(`hr'), nformat(number_d2)
					quietly: putexcel F`irow'=(`ll'), nformat(number_d2)
					quietly: putexcel G`irow'=(`ul'), nformat(number_d2)
					quietly: putexcel B`irow'=("`idg'")
					quietly: putexcel C`irow'=("pooled")
					quietly: putexcel D`irow'=("Additionally adjusted for `ieks_name'")
			tab any`idg', matcell(x), if _st==1
			matrix list x
			quietly: tab any`idg' ensidementia, matcell(y), if _d==1
			matrix list y
			quietly: putexcel set "Pooled results.xlsm", sheet("TABLE2") modify
					quietly: putexcel M`irow'=(x[2,1]), nformat(number)
					quietly: putexcel N`irow'=(y[2,1]), nformat(number)
			local ++irow
			local ++irow
}
quietly: local irow = 59
}
```

*Potential period effect

```
local dg anyinf
local n_dg: word count `dg'
local dg_name `""Any infectious disease""'
local irow 37
		forvalues i=1/`n_dg' {
				quietly: local idg : word `i' of `dg'
				quietly: local idg_name : word  `i' of `dg_name'
				display ""
				display "`idg' followed by broad dementia, adjusted for sex and education and stratified by cohort"
				display ""
				display "Pooled IPD-analysis, potential period effect"
				display ""
				display "$S_TIME  $S_DATE"
				display ""

				use "Inf_dem_lag.dta", clear
				drop if ses==.

				quietly: stset exitpvm, id(id) failure(failure ==1) origin(time syntpvm) enter(time lag_entry_anyinf) scale(365.25)
				keep if _st==1
				quietly: keep id any`idg' supu cohort exitpvm ensidementia failure syntpvm entrypvm ensi`idg'pvm  _st _d _origin _t _t0 ses syntvuosi
				tab any`idg'

				quietly: stdescribe
				quietly: local failures = `r(N_fail)'
				stcox any`idg' 2.supu#cohort 2.ses#cohort 3.ses#cohort c.syntvuosi#cohort, strata(cohort)

				quietly: matrix m = r(table)
				tempname hr ll ul
				scalar `hr' = m[1,1]
				scalar `ll' = m[5,1]
				scalar `ul' = m[6,1]
				quietly: putexcel set "Pooled results.xlsm", sheet("TABLE2") modify
						quietly: putexcel K`irow'=(`e(N_sub)'), nformat(number)
						quietly: putexcel L`irow'=(`e(N_fail)'), nformat(number)
						quietly: putexcel E`irow'=(`hr'), nformat(number_d2)
						quietly: putexcel F`irow'=(`ll'), nformat(number_d2)
						quietly: putexcel G`irow'=(`ul'), nformat(number_d2)
						quietly: putexcel B`irow'=("`idg'")
						quietly: putexcel C`irow'=("pooled")
						quietly: putexcel D`irow'=("Adjusted for period effect")
				tab any`idg', matcell(x), if _st==1
				matrix list x
				quietly: tab any`idg' ensidementia, matcell(y), if _d==1
				matrix list y
				quietly: putexcel set "Pooled results.xlsm", sheet("TABLE2") modify
						quietly: putexcel M`irow'=(x[2,1]), nformat(number)
						quietly: putexcel N`irow'=(y[2,1]), nformat(number)
				local irow 65
				}
```

***** TEST FOR DIFFERENCE BETWEEN INFECTIONS *****

```
*Test for difference.

capture log close
log using "Test for difference v7.log", replace


		use "Inf_dem_lag.dta", replace
		drop if ses==.

		*Intra vs extracellular
		egen ensiintranotextracpvm = rowmin(ensiintracpvm ensiextracpvm)
		replace ensiintranotextracpvm = . if ensiintracpvm==ensiextracpvm
		gen byte anyintranotextrac = 1 if ensiintracpvm==ensiintranotextracpvm & ensiintranotextracpvm!=.
		replace anyintranotextrac = 0 if ensiextracpvm==ensiintranotextracpvm & ensiintranotextracpvm!=. & 
anyintranotextrac!=1
		sum ensiintranotextracpvm
		tab anyintranotextrac
		tab anyintrac
		tab anyextrac


		*Gram+ vs gram-
		egen ensigramplusnotminuspvm = rowmin(ensigrampluspvm ensigramminuspvm)
		replace ensigramplusnotminuspvm = . if ensigrampluspvm==ensigramminuspvm
		gen byte anygramplusnotminus = 1 if ensigrampluspvm==ensigramplusnotminuspvm & 
ensigramplusnotminuspvm!=.
		replace anygramplusnotminus = 0 if ensigramminuspvm==ensigramplusnotminuspvm & 
ensigramplusnotminuspvm!=. & anygramplusnotminus!=1
		sum ensigramplusnotminuspvm
		tab anygramplusnotminus
		tab anygramplus
		tab anygramminus


		*Bacterial infection with sepsis vs without sepsis
		gen byte anybactsepsisnotsepsis = 1 if ensibactsepsispvm==ensibactinfpvm & anybactsepsis==1
		replace anybactsepsisnotsepsis = 0 if ensibact_nonsepsispvm==ensibactinfpvm & anybactsepsisnotsepsis!=1 & 
anybact_nonsepsis==1
		gen ensibactsepsisnotsepsispvm = ensibactinfpvm
		sum ensibactsepsisnotsepsispvm
		tab anybactsepsisnotsepsis
		tab anybact_nonsepsis
		tab anybactsepsis

		*CNS infection vs non cns infection
		gen byte anycnsnotcns = 1 if ensianycnspvm==ensianyinfpvm & anyanycns==1
		replace anycnsnotcns = 0 if ensinoncnspvm==ensianyinfpvm & anycnsnotcns!=1 & anynoncns==1
		gen ensicnsnotcnspvm = ensianyinfpvm
		sum ensicnsnotcnspvm
		tab anycnsnotcns
		tab anyanycns
		tab anynoncns

		*CNS predisposed vs non cns predisposed
		gen byte anycnspredispnotpredisp = 1 if ensianycnspredisppvm==ensianyinfpvm & anyanycnspredisp==1
		replace anycnspredispnotpredisp = 0 if ensinoncnspredisppvm==ensianyinfpvm & anycnspredispnotpredisp!=1 
& anynoncnspredisp==1
		gen ensicnspredispnotpredisppvm = ensianyinfpvm
```

```
sum ensicnspredispnotpredisppvm
tab anycnspredispnotpredisp
tab anyanycnspredisp
tab anynoncnspredisp

*Acute vs chronic
gen byte anyacutenotchronic = 0 if ensianyperspvm==ensianyinfpvm & anyanypers==1
replace anyacutenotchronic = 1 if ensinonperspvm==ensianyinfpvm & anyacutenotchronic!=1 & anynonpers==1
gen ensiacutenotchronicpvm = ensianyinfpvm
sum ensiacutenotchronicpvm
tab anyacutenotchronic
tab anyanypers
tab anynonpers

*Herpesvirus vs persistent viral infection
egen ensiherpesnotpersviralpvm = rowmin(ensiherpespvm ensipersviralpvm)
gen byte anyherpesnotpersviral = 1 if ensiherpespvm==ensiherpesnotpersviralpvm & anyherpes==1
replace anyherpesnotpersviral = 0 if ensipersviralpvm==ensiherpesnotpersviralpvm & anyherpesnotpersviral!=1
& anypersviral==1
sum ensiherpesnotpersviralpvm
tab anyherpesnotpersviral
tab anyherpes
tab anypersviral

*Herpesvirus vs acute viral infection
egen ensiherpesnotacuteviralpvm = rowmin(ensiherpespvm ensiacuteviralpvm)
gen byte anyherpesnotacuteviral = 1 if ensiherpespvm==ensiherpesnotacuteviralpvm & anyherpes==1
replace anyherpesnotacuteviral = 0 if ensiacuteviralpvm==ensiherpesnotacuteviralpvm &
anyherpesnotacuteviral!=1 & anyacuteviral==1
sum ensiherpesnotacuteviralpvm
tab anyherpesnotacuteviral
tab anyherpes
tab anyacuteviral

*Non herpes persistent vs acute viral infection
egen ensipersnotacuteviralpvm = rowmin(ensipersviralpvm ensiacuteviralpvm)
gen byte anypersnotacuteviral = 1 if ensipersviralpvm==ensipersnotacuteviralpvm & anypersviral==1
replace anypersnotacuteviral = 0 if ensiacuteviralpvm==ensipersnotacuteviralpvm & anypersnotacuteviral!=1 &
anyacuteviral==1
sum ensipersnotacuteviralpvm
tab anypersnotacuteviral
tab anypersviral
tab anyacuteviral

*Bacteria vs viruses
egen ensibactnotviralpvm = rowmin(ensibactinfpvm ensiviralinfpvm)
gen byte anybactnotviral = 1 if ensibactinfpvm==ensibactnotviralpvm & anybactinf==1
replace anybactnotviral = 0 if ensiviralinfpvm==ensibactnotviralpvm & anybactnotviral!=1 & anyviralinf==1
sum ensibactnotviralpvm
tab anybactnotviral
tab anybactinf
tab anyviralinf

*Type of viral infection
egen ensivirustypepvm = rowmin(ensiherpespvm ensipersviralpvm ensiacuteviralpvm)
gen byte anyvirustype = 1 if ensiherpespvm==ensivirustypepvm & anyviralinf==1 & ensivirustypepvm!=.
replace anyvirustype = 2 if ensipersviralpvm==ensivirustypepvm & anyviralinf==1 & anyvirustype==. &
ensivirustypepvm!=.
```

```
		replace anyvirustype = 3 if ensiacuteviralpvm==ensivirustypepvm & anyviralinf==1 & anyvirustype==. & 
ensivirustypepvm!=.
		tab anyvirustype
		tab anyviralinf
		sum ensivirustypepvm ensiviralinfpvm
		*viisi tapausta, joilla on virusinfektio, mutta jonka tyyppi ei ole tiedossa.

		*Any persistent viral infection vs acute viral infection
		gen ensiacutenotanypersviralpvm = ensivirustypepvm
		recode anyvirustype 3=1 1=0 2=0, gen(anyacutenotanypersviral)



		*Invasive vs localised
		egen ensisysnotlocalpvm = rowmin(ensisysbactpvm ensilocalbactpvm)
		gen byte anysysnotlocal = 1 if ensisysbactpvm==ensisysnotlocalpvm & anysysbact==1
		replace anysysnotlocal = 0 if ensilocalbactpvm==ensisysnotlocalpvm & anysysnotlocal!=1 & anylocalbact==1
		sum ensisysnotlocalpvm
		tab anysysnotlocal
		tab anysysbact
		tab anylocalbact






local dg sysnotlocal intranotextrac gramplusnotminus bactnotviral acutenotanypersviral cnsnotcns acutenotchronic 
bactsepsisnotsepsis cnspredispnotpredisp
local n_dg: word count `dg'
local dg_name `""Invasive vs localised bacterial infection" "Intra vs extracellular bacterial infection" "Gram-positive vs gram-
negative bacterial infection" "Bacterial vs viral infection" "Acute vs any persisten viral infection" "CNS vs extra-CNS infection" 
"Acute vs chronic infection" "Septic vs not septic bacterial infection" "Infection predisposed towards entering the CNS vs not 
predisposed""'
quietly: local irow = 7
		forvalues i=1/`n_dg' {
			preserve
			quietly: local idg : word `i' of `dg'
			quietly: local idg_name : word  `i' of `dg_name'
			display ""
			display "`idg' followed by broad dementia, adjusted for sex and education and stratified by 
cohort"
			display ""
			display "Pooled IPD-analysis"
			display ""
			display "$S_TIME  $S_DATE"
			display ""

			egen le_`idg' = rowmax(lag_entry_anyinf ensi`idg'pvm)
			gen ensi`idg'pvm_10y = ensi`idg'pvm + round(10*365.25)
			egen le_10y_`idg' = rowmax(lag_entry_anyinf ensi`idg'pvm_10y)

			gen byte anyeither = ensi`idg'pvm !=.
			replace anyeither = . if anyeither==0 & anyanyinf==1
			tab anyeither

			quietly: stset exitpvm, id(id) failure(ensidementia) origin(time syntpvm) enter(time le_`idg') 
scale(365.25)
			quietly: keep id any`idg' ensi`idg'pvm supu cohort exitpvm ensidementia syntpvm entrypvm 
ensi`idg'pvm_10y anyeither ///
```

```
				le_`idg' le_10y_`idg' _st _d _origin _t _t0 ses

				keep if _st==1
				gen `idg'_compare =0
				replace `idg'_compare = 1 if anyeither==1
				tab `idg'_compare
				recode `idg'_compare 1=2 if any`idg'==0
				tab `idg'_compare
				tab any`idg'
				tab anyeither
				quietly: stdescribe
				quietly: local failures = `r(N_fail)'

				stcox i.`idg'_compare 2.supu#cohort 2.ses#cohort 3.ses#cohort, strata(cohort)
				test 1.`idg'_compare = 2.`idg'_compare

				quietly: putexcel set "Pooled results.xlsm", sheet("FIGURE compare dist") modify
						quietly: putexcel K`irow'=(`e(N_sub)'), nformat(number)
						quietly: putexcel L`irow'=(`e(N_fail)'), nformat(number)
						quietly: putexcel E`irow'=(`r(p)'), nformat(number_d2)
						quietly: putexcel B`irow'=("`idg'")
						quietly: putexcel C`irow'=("pooled")
						quietly: putexcel D`irow'=("`idg_name'")
				local ++irow

				*Dementia occurring from year 10 onwards
				stset exitpvm, id(id) failure(ensidementia) origin(time syntpvm) enter(time le_10y_`idg')
scale(365.25)
				stdescribe
				quietly: local failures = `r(N_fail)'

				stcox i.`idg'_compare 2.supu#cohort 2.ses#cohort 3.ses#cohort, strata(cohort)
				test 1.`idg'_compare = 2.`idg'_compare

				quietly: putexcel set "Pooled results.xlsm", sheet("FIGURE compare dist") modify
						quietly: putexcel K`irow'=(`e(N_sub)'), nformat(number)
						quietly: putexcel L`irow'=(`e(N_fail)'), nformat(number)
						quietly: putexcel E`irow'=(`r(p)'), nformat(number_d2)
						quietly: putexcel B`irow'=("`idg'")
						quietly: putexcel C`irow'=("pooled")
						quietly: putexcel D`irow'=("`idg_name' from year 10 onwards")
				local ++irow
				local ++irow
				restore
}

		log close

*Comparing viral infections
capture log close
log using "herpes vs other persistent viral v7.log", replace
local dg herpesnotpersviral
local n_dg: word count `dg'
local dg_name `""Herpesvirus infection vs other potentially persistent viral infection""'
quietly: local irow = 40
		forvalues i=1/`n_dg' {
				preserve
				quietly: local idg : word `i' of `dg'
```

```
quietly: local idg_name : word `i' of `dg_name'
display ""
display "`idg' followed by broad dementia, adjusted for sex and education and stratified by
cohort"
display ""
display "Pooled IPD-analysis"
display ""
display "$S_TIME  $S_DATE"
display ""

egen le_`idg' = rowmax(lag_entry_anyinf ensi`idg'pvm)
gen ensi`idg'pvm_10y = ensi`idg'pvm + round(10*365.25)
egen le_10y_`idg' = rowmax(lag_entry_anyinf ensi`idg'pvm_10y)

gen byte anyeither = ensi`idg'pvm !=.
replace anyeither = . if anyeither==0 & anyanyinf==1
tab anyeither

quietly: stset exitpvm, id(id) failure(ensidementia) origin(time syntpvm) enter(time le_`idg')
scale(365.25)
quietly: keep id any`idg' ensi`idg'pvm supu cohort exitpvm ensidementia syntpvm entrypvm
ensi`idg'pvm_10y anyeither ///
le_`idg' le_10y_`idg' _st _d _origin _t _t0 ses

keep if _st==1
gen `idg'_compare =0
replace `idg'_compare = 1 if anyeither==1
tab `idg'_compare
recode `idg'_compare 1=2 if any`idg'==0
tab `idg'_compare
tab any`idg'
tab anyeither

quietly: stdescribe
quietly: local failures = `r(N_fail)'

stcox i.`idg'_compare 2.supu#cohort 2.ses#cohort 3.ses#cohort, strata(cohort)
test 1.`idg'_compare = 2.`idg'_compare

quietly: putexcel set "Pooled results.xlsm", sheet("FIGURE compare dist") modify
        quietly: putexcel K`irow'=(`e(N_sub)'), nformat(number)
        quietly: putexcel L`irow'=(`e(N_fail)'), nformat(number)
        quietly: putexcel E`irow'=(`r(p)'), nformat(number_d2)
        quietly: putexcel B`irow'=("`idg'")
        quietly: putexcel C`irow'=("pooled")
        quietly: putexcel D`irow'=("`idg_name'")
local ++irow

*Dementia occurring from year 10 onwards
stset exitpvm, id(id) failure(ensidementia) origin(time syntpvm) enter(time le_10y_`idg')
scale(365.25)
stdescribe
quietly: local failures = `r(N_fail)'

stcox i.`idg'_compare 2.supu#cohort 2.ses#cohort 3.ses#cohort, strata(cohort)
test 1.`idg'_compare = 2.`idg'_compare

quietly: putexcel set "Pooled results.xlsm", sheet("FIGURE compare dist") modify
```

```
quietly: putexcel K`irow'=(`e(N_sub)'), nformat(number)
quietly: putexcel L`irow'=(`e(N_fail)'), nformat(number)
quietly: putexcel E`irow'=(`r(p)'), nformat(number_d2)
quietly: putexcel B`irow'=("`idg'")
quietly: putexcel C`irow'=("pooled")
quietly: putexcel D`irow'=("`idg_name' from year 10 onwards")
local ++irow
local ++irow
restore
}
log close


capture log close
log using "Types of viral infection v7.log", replace

local dg virustype
local n_dg: word count `dg'
local dg_name `""Types of viral infection""'
quietly: local irow = 34
forvalues i=1/`n_dg' {
preserve
quietly: local idg : word `i' of `dg'
quietly: local idg_name : word  `i' of `dg_name'
display ""
display "`idg' followed by broad dementia, adjusted for sex and education and stratified by
cohort"
display ""
display "Pooled IPD-analysis"
display ""
display "$S_TIME  $S_DATE"
display ""

egen le_`idg' = rowmax(lag_entry_anyinf ensi`idg'pvm)
gen ensi`idg'pvm_10y = ensi`idg'pvm + round(10*365.25)
egen le_10y_`idg' = rowmax(lag_entry_anyinf ensi`idg'pvm_10y)

gen byte anyeither = ensi`idg'pvm !=.
replace anyeither = . if anyeither==0 & anyanyinf==1
tab anyeither

quietly: stset exitpvm, id(id) failure(ensidementia) origin(time syntpvm) enter(time le_`idg')
scale(365.25)
quietly: keep id any`idg' ensi`idg'pvm supu cohort exitpvm ensidementia syntpvm entrypvm
ensi`idg'pvm_10y anyeither ///
le_`idg' le_10y_`idg' _st _d _origin _t _t0 ses anyviralinf anyanyinf

keep if _st==1
gen  `idg'_compare = any`idg' if anyeither==1
replace `idg'_compare = 0 if anyanyinf==0
tab `idg'_compare
tab any`idg'
tab anyeither

quietly: stdescribe
quietly: local failures = `r(N_fail)'
```

```
				if "`idg'"=="virustype" {
				local comp "viralinf"
				replace anyviralinf = . if anyvirustype==.
				}
				tab any`comp'
				stcox i.`idg'_compare 2.supu#cohort 2.ses#cohort 3.ses#cohort, strata(cohort)
				test 1.`idg'_compare = 2.`idg'_compare = 3.`idg'_compare

				quietly: putexcel set "Pooled results.xlsm", sheet("FIGURE compare dist") modify
						quietly: putexcel K`irow'=(`e(N_sub)'), nformat(number)
						quietly: putexcel L`irow'=(`e(N_fail)'), nformat(number)
						quietly: putexcel E`irow'=(`r(p)'), nformat(number_d2)
						quietly: putexcel B`irow'=("`comp'")
						quietly: putexcel C`irow'=("pooled")
						quietly: putexcel D`irow'=("`idg_name'")
				local ++irow

				*Dementia occurring from year 10 onwards
				quietly: stset exitpvm, id(id) failure(ensidementia) origin(time syntpvm) enter(time le_10y_`idg')
scale(365.25)

				stdescribe
				quietly: local failures = `r(N_fail)'

				stcox i.`idg'_compare 2.supu#cohort 2.ses#cohort 3.ses#cohort, strata(cohort)
				test 1.`idg'_compare = 2.`idg'_compare = 3.`idg'_compare

				quietly: putexcel set "Pooled results.xlsm", sheet("FIGURE compare dist") modify
						quietly: putexcel K`irow'=(`e(N_sub)'), nformat(number)
						quietly: putexcel L`irow'=(`e(N_fail)'), nformat(number)
						quietly: putexcel E`irow'=(`r(p)'), nformat(number_d2)
						quietly: putexcel B`irow'=("`idg'")
						quietly: putexcel C`irow'=("pooled")
						quietly: putexcel D`irow'=("`idg_name' from year 10 onwards")
				local ++irow
				local ++irow
				restore
				}

		log close



***** NUMBER OF INFECTIONS *******

capture log close
log using "P for number of simultaneous infections v7.log", replace

local dg infcount
local n_dg: word count `dg'
local dg_name `""No. of infections""'

				use "Inf_dem_lag.dta", replace
				merge 1:1 id using "Infection count.dta"
				tab anyinfcount
				recode anyinfcount 3=2 4=2
				drop if ses==.
				gen ensiinfcountpvm = ensianyinfpvm
```

```
quietly: local irow = 37
			forvalues i=1/`n_dg' {
				preserve
				quietly: local idg : word `i' of `dg'
				quietly: local idg_name : word  `i' of `dg_name'
				display ""
				display "`idg' followed by broad dementia, adjusted for sex and education and stratified by 
cohort"
				display ""
				display "Pooled IPD-analysis, normal model and 10-year exclusion"
				display ""
				display "$S_TIME  $S_DATE"
				display ""

				quietly: stset exitpvm, id(id) failure(ensidementia) origin(time syntpvm) enter(time 
lag_entry_anyinf) scale(365.25)
				quietly: keep id any`idg' supu cohort exitpvm ensidementia syntpvm entrypvm ensi`idg'pvm  _st 
_d _origin _t _t0 ses ///
				lag_entry_anyinf lag_entry_10y_anyinf
				keep if _st==1
				tab any`idg'

				quietly: stdescribe
				quietly: local failures = `r(N_fail)'
				stcox i.any`idg' 2.supu#cohort 2.ses#cohort 3.ses#cohort, strata(cohort)
				test 1.any`idg' = 2.any`idg'

				quietly: putexcel set "Pooled results.xlsm", sheet("FIGURE compare dist") modify
					quietly: putexcel K`irow'=(`e(N_sub)'), nformat(number)
					quietly: putexcel L`irow'=(`e(N_fail)'), nformat(number)
					quietly: putexcel E`irow'=(`r(p)'), nformat(number_d2)
					quietly: putexcel B`irow'=("`idg'")
					quietly: putexcel C`irow'=("pooled")
					quietly: putexcel D`irow'=("`idg_name'")
				local ++irow

				*Dementia occurring from year 10 onwards
				quietly: stset exitpvm, id(id) failure(ensidementia) origin(time syntpvm) enter(time 
lag_entry_10y_anyinf) scale(365.25)

				quietly: stdescribe
				quietly: local failures = `r(N_fail)'
				stcox i.any`idg' 2.supu#cohort 2.ses#cohort 3.ses#cohort, strata(cohort)
				test 1.any`idg' = 2.any`idg'

				quietly: putexcel set "Pooled results.xlsm", sheet("FIGURE compare dist") modify
					quietly: putexcel K`irow'=(`e(N_sub)'), nformat(number)
					quietly: putexcel L`irow'=(`e(N_fail)'), nformat(number)
					quietly: putexcel E`irow'=(`r(p)'), nformat(number_d2)
					quietly: putexcel B`irow'=("`idg'")
					quietly: putexcel C`irow'=("pooled")
					quietly: putexcel D`irow'=("`idg_name' from year 10 onwards")
				local ++irow
				local ++irow
				restore
				}
log close
```

```
capture log close
log using "HSV vs other herpes v7.log", replace
				use "Inf_dem_lag.dta", replace
				merge 1:1 id using "herpeskoodit.dta"
				drop _merge
				drop if ses==.
				tab anyhsv12
				gen anynonhsv = 0 if anyanyinf==0
				replace anynonhsv = 1 if anyherpes==1 & anyhsv12!=1

				gen herpes_compare = 1 if anyhsv12==1
				replace herpes_compare = 2 if anynonhsv==1
				replace herpes_compare = 0 if anyanyinf==0
				tab herpes_compare

				quietly: stset exitpvm, id(id) failure(ensidementia) origin(time syntpvm) enter(time
lag_entry_herpes) scale(365.25)
				quietly: keep id anyherpes anyhsv12 anynonhsv supu cohort exitpvm ensidementia syntpvm
entrypvm ensiherpespvm ///
				_st _d _origin _t _t0 ses herpes_compare
				tab anyherpes

				quietly: stdescribe
				quietly: local failures = `r(N_fail)'
				stcox i.herpes_compare 2.supu#cohort 2.ses#cohort 3.ses#cohort, strata(cohort)
				test 1.herpes_compare=2.herpes_compare
log close

capture log close
log using "Type of Gram-positive and Gram-negative infections v7.log", replace

				quietly: use "Inf_dem_lag.dta", clear
				merge 1:1 id using "grampluskoodit.dta"
				drop _merge
				merge 1:1 id using "gramminuskoodit.dta"
				drop _merge
				drop if ses==.

				anystreptoc anycdiffic anyothergramplus anysalmonella anylyme anyothergramminussepsis

				gen gramplus_compare = 0 if anyanyinf==0
				replace gramplus_compare = 1 if anystreptoc==1
				replace gramplus_compare = 2 if anycdiffic==1
				replace gramplus_compare = 3 if anyothergramplus==1
				tab anygramplus
				tab gramplus_compare

				gen gramminus_compare = 0 if anyanyinf==0
				replace gramminus_compare = 1 if anysalmonella==1
				replace gramminus_compare = 2 if anylyme==1
				replace gramminus_compare = 3 if anyothergramminussepsis==1 | anyothergramminus==1
				tab anygramminus
				tab gramminus_compare

				quietly: stset exitpvm, id(id) failure(ensidementia) origin(time syntpvm) enter(time
lag_entry_gramplus) scale(365.25)
```

```
				quietly: keep id *gram* supu cohort exitpvm ensidementia syntpvm entrypvm  _st _d _origin _t
_t0 ses
				tab anygramplus if _st==1
				tab gramplus_compare if _st==1

				stcox i.gramplus_compare 2.supu#cohort 2.ses#cohort 3.ses#cohort, strata(cohort)
				test 1.gramplus_compare = 2.gramplus_compare
				test 1.gramplus_compare = 3.gramplus_compare
				test 2.gramplus_compare = 3.gramplus_compare
				test 1.gramplus_compare = 2.gramplus_compare = 3.gramplus_compare

				quietly: stset exitpvm, id(id) failure(ensidementia) origin(time syntpvm) enter(time
lag_entry_gramminus) scale(365.25)
				quietly: keep id *gram* supu cohort exitpvm ensidementia syntpvm entrypvm  _st _d _origin _t
_t0 ses
				tab anygramminus if _st==1
				tab gramminus_compare if _st==1

				stcox i.gramminus_compare 2.supu#cohort 2.ses#cohort 3.ses#cohort, strata(cohort)
				test 1.gramminus_compare = 2.gramminus_compare
				test 1.gramminus_compare = 3.gramminus_compare
				test 2.gramminus_compare = 3.gramminus_compare
				test 1.gramminus_compare = 2.gramminus_compare = 3.gramminus_compare

		log close


********* PROPORTIONAL HAZARDS ASSUMPTIONS **********

capture log close
log using "Proportional hazards assumptions v3.log", replace

local dg anyinf bactinf sysbact localbact bactsepsis bact_nonsepsis extrac intrac gramplus gramminus viralinf herpes persviral
acuteviral anycns noncns
local n_dg: word count `dg'
local dg_name `""Any infectious disease" "Any bacterial infection" "Invasive bacterial infection" "Localised bacterial infection"
"Bacterial infection with sepsis" "Bacterial infection without sepsis" "Extracellular bacterial infection" "Intracellular bacterial
infection" "Gram-positive bacterial infection" "Gram-negative bacterial infection" "Any viral infection" "Herpesvirus infection"
"Other potentially persistent viral infection" "Acute viral infection" "CNS infection" "Extra-CNS infection""'

				quietly: use "Inf_dem_lag.dta", clear
				drop if ses==.
				egen lag_entry_anycns = rowmax(lag_entry_anyinf ensianycnspvm)
				egen lag_entry_noncns = rowmax(lag_entry_anyinf ensinoncnspvm)
				replace anyanycns = . if anyanycns==0 & anyanyinf==1
				replace anynoncns = . if anynoncns==0 & anyanyinf==1



quietly: local irow = 7
		forvalues i=1/`n_dg' {
				quietly: local idg : word `i' of `dg'
				quietly: local idg_name : word  `i' of `dg_name'
				display ""
				display "`idg' followed by broad dementia, adjusted for sex and education and stratified by
cohort"
				display ""
				display "Pooled IPD-analysis, normal model"
				display ""
```

```
display "$S_TIME  $S_DATE"
display ""

preserve

quietly: stset exitpvm, id(id) failure(ensidementia) origin(time syntpvm) enter(time lag_entry_`idg') scale(365.25)
keep if _st==1
quietly: keep id any`idg' supu cohort exitpvm ensidementia syntpvm entrypvm ensi`idg'pvm  _st _d _origin _t _t0 ses lag_entry_`idg'
tab any`idg'

quietly: stdescribe
quietly: local failures = `r(N_fail)'
stcox any`idg' 2.supu#cohort 2.ses#cohort 3.ses#cohort, strata(cohort)
matrix r = r(table)
estat phtest, detail
matrix m = r(phtest)
quietly: putexcel set "Pooled results.xlsm", sheet("IPD, PH-assumptions") modify
quietly: putexcel A`irow'="`idg'", nformat(number)
quietly: putexcel B`irow'="`idg_name'", nformat(number)
quietly: putexcel C`irow'=`r(p)', nformat(number_d2)
quietly: putexcel D`irow'=matrix(m[1,4]), nformat(number_d2)
quietly: putexcel E`irow'=matrix(m[2,4]), nformat(number_d2)
quietly: putexcel F`irow'=matrix(m[3,4]), nformat(number_d2)
quietly: putexcel G`irow'=matrix(m[4,4]), nformat(number_d2)
quietly: putexcel H`irow'=matrix(m[5,4]), nformat(number_d2)
quietly: putexcel I`irow'=matrix(m[6,4]), nformat(number_d2)
quietly: putexcel J`irow'=matrix(m[7,4]), nformat(number_d2)
quietly: putexcel K`irow'=matrix(m[8,4]), nformat(number_d2)
quietly: putexcel L`irow'=matrix(m[9,4]), nformat(number_d2)
quietly: putexcel M`irow'=matrix(m[10,4]), nformat(number_d2)
quietly: putexcel N`irow'=matrix(r[1,1]), nformat(number_d2)
local ++irow
restore
}
quietly: local irow = 6
quietly: putexcel set "Pooled results.xlsm", sheet("IPD, PH-assumptions") modify
quietly: putexcel E`irow'="Sex_FPS", nformat(number_d2)
quietly: putexcel F`irow'="Sex_HeSSup", nformat(number_d2)
quietly: putexcel G`irow'="Sex_STW", nformat(number_d2)
quietly: putexcel E`irow'="2.edu_FPS", nformat(number_d2)
quietly: putexcel F`irow'="2.edu_HeSSup", nformat(number_d2)
quietly: putexcel G`irow'="2.edu_STW", nformat(number_d2)
quietly: putexcel E`irow'="3.edu_FPS", nformat(number_d2)
quietly: putexcel F`irow'="3.edu_HeSSup", nformat(number_d2)
quietly: putexcel G`irow'="3.edu_STW", nformat(number_d2)
log close
```

*any infectious disease, invasive bacterial infections, extracellular bacterial infections, intracellular bacterial infections, Gram-negative bacterial infections, any viral infection, herpesvirus infections, other potentially persistent viral infections and non-cns infections had p<0.05 which means evidence for the violation of the proportional hazards assumption. Let's draw figures from them:

*********************Scaled Schoenfeld residuals**********************************

```
capture log close
log using "Scaled Schoenfeld residuals.log", replace


*Scaled schoenfeld residuals -figures:
*The code below that draws the figures has been modified from:
*Royston and Lambert: Flexible Parametric Survival Analysis Using Stata: Beyond the Cox Model. Chapter 7.3 What do we mean by a TD effect?


local dg anyinf sysbact extrac intrac gramminus viralinf herpes persviral noncns
local n_dg: word count `dg'
local dg_name `""Any infectious disease (vs no infection)" "Invasive bacterial infection (vs no infection)" "Extracellular bacterial infection (vs no infection)" "Intracellular bacterial infection (vs no infection)" "Gram-negative bacterial infection (vs no infection)" "Any viral infection (vs no infection)" "Herpesvirus infection (vs no infection)" "Other potentially persistent viral infection (vs no infection)" "Extra-CNS infection (vs no infection)"'
		quietly: use "Inf_dem_lag.dta", clear
		drop if ses==.
		egen lag_entry_anycns = rowmax(lag_entry_anyinf ensianycnspvm)
		egen lag_entry_noncns = rowmax(lag_entry_anyinf ensinoncnspvm)
		replace anyanycns = . if anyanycns==0 & anyanyinf==1
		replace anynoncns = . if anynoncns==0 & anyanyinf==1
		drop if ses==.

forvalues i=1/`n_dg' {
		quietly: local idg : word `i' of `dg'
		quietly: local idg_name : word  `i' of `dg_name'

		preserve

		local j = 1
		foreach cohort in FPS HeSSup STW {
				gen `cohort'supu=0
				replace `cohort'supu=1 if supu==2 & cohort==`j'
				gen `cohort'ses2=0
				replace `cohort'ses2=1 if ses==2 & cohort==`j'
				gen `cohort'ses3=0
				replace `cohort'ses3=1 if ses==3 & cohort==`j'
				local ++j
				}
		tabulate cohort, gen(dummycohort)

		quietly: stset exitpvm, id(id) failure(ensidementia) origin(time syntpvm) enter(time lag_entry_`idg') scale(365.25)
				quietly: keep id any`idg' *supu* cohort exitpvm ensidementia syntpvm entrypvm ensi`idg'pvm  _st _d _origin _t _t0 *ses*
				keep if _st==1
				tab any`idg'

		stcox any`idg' FPSsupu HeSSupsupu STWsupu FPSses2 HeSSupses2 STWses2 FPSses3 HeSSupses3 STWses3, strata(cohort)
		capture drop *sca*
		predict sca*, scaledsch
		running sca1 _t if _d==1, gen(smooth_sca) gense(smooth_sca_se) nodraw
		gen smooth_esca = exp(smooth_sca)
		gen smooth_esca_lci = exp(smooth_sca - 1.96*smooth_sca_se)
		gen smooth_esca_uci = exp(smooth_sca + 1.96*smooth_sca_se)
```

```
				local betaround: di %7.2f exp(_b[any`idg'])
				tokenize "`betaround'"
				di "`1'"
				twoway (rarea smooth_esca_lci smooth_esca_uci _t, pstyle(ci) sort yaxis(1 2)) ///
						(line smooth_esca _t, sort clpattern(solid)) ///
						(function y = 1, lpattern(shortdash) range(_t)) ///
						(function y = `1', lpattern(longdash) range(_t)), ///
						legend(order(2 "Exponentiated scaled Schoenfeld residuals" 1 "95% confidence
interval" 4 "Overall hazard ratio   ") holes(2) size(small)) ///
						title("`idg_name'" "and dementia", size(medsmall)) ///
						ytitle("Exponentiated scaled Schoenfeld residuals", size(small)) ///
						xtitle("Age (years)", size(small)) ///
						yscale(log range(0.1 100)) ///
						scheme(sj) ///
						ylabel(0.1 1 10 100, labsize(medsmall) angle(0)) ///
						ylabel("`1'", angle(0) axis(2))
				graph save sca_`idg'.gph, replace
				graph export sca_`idg'.pdf, as(pdf) replace
				restore
				}


		*Combine the figures:


		*An empty figure that is needed if the number of figures is not divisible by three:
		scatter cohort supu if _n==1, ysize(3.9) xsize(4.135) scheme(s1color) plotregion(style(none)) yscale(off noline)
xscale(off noline) mcolor(%0) graphregion(color(white))
		graph save "sca_.gph", replace


		local dg anyinf sysbact extrac intrac gramminus viralinf herpes persviral noncns
		local n_dg: word count `dg'
		local i=1
		while `i' <= 9 {
				forvalues j=1/3 {
				local k = `i'+`j'-1
				quietly: local dg`j' : word `k' of `dg'
				}
		graph combine sca_`dg1'.gph sca_`dg2'.gph sca_`dg3'.gph, col(1) altshrink  scheme(s1color)
graphregion(color(white))  plotregion(color(gs15)) ysize(8.27) xsize(5.83) // ysize ja xsize A5:n mukaan (tuumina)
		graph export "IPD_sca_`i'.emf", as(emf) replace
		local i = `i'+3
				}

		log close



***** TIME-DEPENDENT ANALYSES ********

****eFIGURE 11 *******

*(time-dependent version of the analyses in FIGURE 2)
capture log close
log using "FIGURE 2.log", replace
local dg anyinf bactinf sysbact localbact bactsepsis bact_nonsepsis extrac intrac gramplus gramminus viralinf herpes persviral
acuteviral
```

```
local row 7 10 13 14 16 17 20 21 24 25 28 31 32 33
local n_dg: word count `dg'
local dg_name `""Any infectious disease vs not" "Any bacterial infection vs not" "Potentially invasive bacterial infection vs not" "Localised bacterial infection vs not" "Bacterial infection with sepsis vs not" "Bacterial infection without sepsis vs not" "Extracellular bacterial infection vs not" "Intracellular bacterial infection vs not" "Gram-positive bacterial infection vs not" "Gram-negative bacterial infection vs not" "Any viral infection vs not" "Herpesvirus (persistent) infection vs not" "Other persistent viral infection vs not" "Acute viral infection vs not""'
		forvalues i=1/`n_dg' {
				quietly: local idg : word `i' of `dg'
				quietly: local idg_name : word  `i' of `dg_name'
				quietly: local irow : word `i' of `row'
				display ""
				display "`idg' followed by broad dementia, adjusted for sex and education and stratified by cohort"
				display ""
				display "Pooled IPD-analysis, normal model and 10-year exclusion"
				display ""
				display "$S_TIME  $S_DATE"
				display ""

				quietly: use "Pooled infections_trimmattu", clear
				drop if ses==.
				quietly: stset exitpvm, id(id) failure(ensidementia) origin(time syntpvm) enter(time entrypvm) scale(365.25)
				quietly: keep id any`idg' supu cohort exitpvm ensidementia syntpvm entrypvm ensi`idg'pvm  _st _d _origin _t _t0 ses
				tab any`idg'
				stsplit `idg'_split, at(0) after(time=ensi`idg'pvm)
				tab `idg'_split
				gen `idg'_timedep =0
				replace `idg'_timedep = 1 if any`idg'==1 & `idg'_split==0
				tab `idg'_timedep
				tab any`idg' if `idg'_split!=-1

				quietly: stdescribe
				quietly: local failures = `r(N_fail)'
				stcox `idg'_timedep 2.supu#cohort 2.ses#cohort 3.ses#cohort, strata(cohort)
				quietly: matrix m = r(table)
				tempname hr ll ul
				scalar `hr' = m[1,1]
				scalar `ll' = m[5,1]
				scalar `ul' = m[6,1]
				quietly: putexcel set "Pooled results.xlsm", sheet("FIGURE 2 revised") modify
					quietly: putexcel K`irow'=(`e(N_sub)'), nformat(number)
					quietly: putexcel L`irow'=(`e(N_fail)'), nformat(number)
					quietly: putexcel E`irow'=(`hr'), nformat(number_d2)
					quietly: putexcel F`irow'=(`ll'), nformat(number_d2)
					quietly: putexcel G`irow'=(`ul'), nformat(number_d2)
					quietly: putexcel B`irow'=("`idg'")
					quietly: putexcel C`irow'=("pooled")
					quietly: putexcel D`irow'=("`idg_name'")
				tab any`idg', matcell(x), if `idg'_split!=-1
				matrix list x
				quietly: tab any`idg' ensidementia, matcell(y), if `idg'_split!=-1 & _d==1
				matrix list y
				quietly: putexcel set "Pooled results.xlsm", sheet("FIGURE 2 revised") modify
					quietly: putexcel M`irow'=(x[2,1]), nformat(number)
					quietly: putexcel N`irow'=(y[2,1]), nformat(number)
```

```
				local irow = `irow' + 29

				*Dementia occurring from year 10 onwards
				gen ensi`idg'pvm10v = ensi`idg'pvm + round(10*365.25) if ensi`idg'pvm<entrypvm
				replace ensi`idg'pvm10v = entrypvm if ensi`idg'pvm10v<entrypvm
				replace _t0 = (ensi`idg'pvm10v-syntpvm)/365.25 if ensi`idg'pvm10v!=.
				replace _t0 = _t0 + 10 if ensi`idg'pvm10v==.
				drop if _t0>=_t
				quietly: stdescribe
				quietly: local failures = `r(N_fail)'
				stcox `idg'_timedep 2.supu#cohort 2.ses#cohort 3.ses#cohort, strata(cohort)
				quietly: matrix m = r(table)
				scalar `hr' = m[1,1]
				scalar `ll' = m[5,1]
				scalar `ul' = m[6,1]
				quietly: putexcel set "Pooled results.xlsm", sheet("FIGURE 2 revised") modify
						quietly: putexcel K`irow'=(`e(N_sub)'), nformat(number)
						quietly: putexcel L`irow'=(`e(N_fail)'), nformat(number)
						quietly: putexcel E`irow'=(`hr'), nformat(number_d2)
						quietly: putexcel F`irow'=(`ll'), nformat(number_d2)
						quietly: putexcel G`irow'=(`ul'), nformat(number_d2)
						quietly: putexcel B`irow'=("`idg'")
						quietly: putexcel C`irow'=("pooled")
						quietly: putexcel D`irow'=("`idg_name'")
				tab any`idg', matcell(x), if `idg'_split!=-1
				matrix list x
				quietly: tab any`idg' ensidementia, matcell(y), if `idg'_split!=-1 & _d==1
				matrix list y
				quietly: putexcel set "Pooled results.xlsm", sheet("FIGURE 2 revised") modify
						quietly: putexcel M`irow'=(x[2,1]), nformat(number)
						quietly: putexcel N`irow'=(y[2,1]), nformat(number)
				}
log close



***** TIME-DEPENDENT ANALYSES: SINGLE VS MULTIPLE INFECTIONS *******

capture log close
log using "Single vs multiple infections.log", replace

local dg_name `""Multiple infections""'
local irow 16
local prow = `irow'

				quietly: use "Pooled infections_trimmattu", clear
				merge 1:1 id using "Coinfections_trimmattu"
				drop _merge
				drop if ses==.

				quietly: stset exitpvm, id(id) failure(ensidementia) origin(time syntpvm) enter(time entrypvm)
scale(365.25)
				quietly: keep id anyanyinf supu cohort exitpvm ensidementia syntpvm entrypvm
ensi*anyinfpvm *toinen* *kolmas*  _st _d _origin _t _t0 ses
				sum ensi*anyinfpvm

				stsplit first_split, at(0) after(time=ensianyinfpvm)
				gen multiinf_timedep =0
```

```
tab multiinf_timedep
replace multiinf_timedep = 1 if anyanyinf==1 & first_split==0
tab multiinf_timedep
stsplit second_split, at(0) after(time=ensitoinenanyinfpvm)
replace multiinf_timedep = 2 if toinenanyinf==1 & second_split==0
tab multiinf_timedep
stsplit third_split, at(0) after(time=ensikolmasanyinfpvm)
replace multiinf_timedep = 3 if kolmasanyinf==1 &third_split==0
tab multiinf_timedep

quietly: stdescribe
quietly: local failures = `r(N_fail)'
stcox i.multiinf_timedep 2.supu#cohort 2.ses#cohort 3.ses#cohort, strata(cohort)
quietly: matrix m = r(table)
matrix list m
tempname hr1 ll1 ul1 hr2 ll2 ul2 hr3 ll3 ul3
scalar `hr1' = m[1,2]
scalar `ll1' = m[5,2]
scalar `ul1' = m[6,2]
scalar `hr2' = m[1,3]
scalar `ll2' = m[5,3]
scalar `ul2' = m[6,3]
scalar `hr3' = m[1,4]
scalar `ll3' = m[5,4]
scalar `ul3' = m[6,4]

forvalues i =1/3 {
quietly: putexcel set "Pooled results.xlsm", sheet("FIGURE sensitivity") modify
		quietly: putexcel K`irow'=(`e(N_sub)'), nformat(number)
		quietly: putexcel L`irow'=(`e(N_fail)'), nformat(number)
		quietly: putexcel E`irow'=(`hr`i''), nformat(number_d2)
		quietly: putexcel F`irow'=(`ll`i''), nformat(number_d2)
		quietly: putexcel G`irow'=(`ul`i''), nformat(number_d2)
		quietly: putexcel B`irow'=("Multiinf")
		quietly: putexcel C`irow'=("pooled")
		quietly: putexcel D`irow'=("Single vs multiple infections, infection No. `i'")
tab multiinf_timedep, matcell(x), if first_split!=-1 & second_split!=-1 & third_split!=-1
matrix list x
tab multiinf_timedep ensidementia, matcell(y), if first_split!=-1 & second_split!=-1 &
third_split!=-1 &_d==1

matrix list y
quietly: putexcel set "Pooled results.xlsm", sheet("FIGURE sensitivity") modify
		local j = `i' + 1
		quietly: putexcel M`irow'=(x[`j',1]), nformat(number)
		quietly: putexcel N`irow'=(y[`j',1]), nformat(number)
local ++irow
local ++irow
}
*P for trend
stcox multiinf_timedep 2.supu#cohort 2.ses#cohort 3.ses#cohort, strata(cohort)
quietly: matrix n = r(table)
matrix list n
tempname p
scalar `p' = n[4,1]
quietly: putexcel set "Pooled results.xlsm", sheet("FIGURE sensitivity") modify
		quietly: putexcel Q`prow'=(`p'), nformat(number)
```

```
*Dementia occurring from year 10 onwards
local i 1
foreach idg in anyinf toinenanyinf kolmasanyinf {
display `i'
replace _t0 = _t0 + 10 if multiinf_timedep==0 & `i'==1
drop if _t0>=_t
gen ensi`idg'pvm10v = ensi`idg'pvm + round(10*365.25) if ensi`idg'pvm<entrypvm
replace ensi`idg'pvm10v = entrypvm if ensi`idg'pvm10v<entrypvm
replace _t0 = (ensi`idg'pvm10v-syntpvm)/365.25 if ensi`idg'pvm10v!=. & multiinf_timedep==`i'
replace _t0 = _t0 + 10 if ensi`idg'pvm10v==. & multiinf_timedep==`i'
drop if _t0>=_t
local ++i
}

quietly: stdescribe
quietly: local failures = `r(N_fail)'
stcox i.multiinf_timedep 2.supu#cohort 2.ses#cohort 3.ses#cohort, strata(cohort)
quietly: matrix m = r(table)
matrix list m
tempname hr1 ll1 ul1 hr2 ll2 ul2 hr3 ll3 ul3
scalar `hr1' = m[1,2]
scalar `ll1' = m[5,2]
scalar `ul1' = m[6,2]
scalar `hr2' = m[1,3]
scalar `ll2' = m[5,3]
scalar `ul2' = m[6,3]
scalar `hr3' = m[1,4]
scalar `ll3' = m[5,4]
scalar `ul3' = m[6,4]

local irow 17
forvalues i =1/3 {
quietly: putexcel set "Pooled results.xlsm", sheet("FIGURE sensitivity") modify
        quietly: putexcel K`irow'=(`e(N_sub)'), nformat(number)
        quietly: putexcel L`irow'=(`e(N_fail)'), nformat(number)
        quietly: putexcel E`irow'=(`hr`i''), nformat(number_d2)
        quietly: putexcel F`irow'=(`ll`i''), nformat(number_d2)
        quietly: putexcel G`irow'=(`ul`i''), nformat(number_d2)
        quietly: putexcel B`irow'=("Multiinf")
        quietly: putexcel C`irow'=("pooled")
        quietly: putexcel D`irow'=("Dementia occurring more than 10 years after the
hospitalisation, infection No. `i'")
tab multiinf_timedep, matcell(x), if first_split!=-1 & second_split!=-1 & third_split!=-1
matrix list x
tab multiinf_timedep ensidementia, matcell(y), if first_split!=-1 & second_split!=-1 &
third_split!=-1 &_d==1
matrix list y
quietly: putexcel set "Pooled results.xlsm", sheet("FIGURE sensitivity") modify
        local j = `i' + 1
        quietly: putexcel M`irow'=(x[`j',1]), nformat(number)
        quietly: putexcel N`irow'=(y[`j',1]), nformat(number)
local ++irow
local ++irow
}

*P for trend
local ++prow
stcox multiinf_timedep 2.supu#cohort 2.ses#cohort 3.ses#cohort, strata(cohort)
```

```
                                        quietly: matrix n = r(table)
                                        matrix list n
                                        tempname p
                                        scalar `p' = n[4,1]
                                        quietly: putexcel set "Pooled results.xlsm", sheet("FIGURE sensitivity") modify
                                                        quietly: putexcel Q`prow'=(`p'), nformat(number)

log close

***** TIME-DEPENDENT ANALYSES: One or several different pathogens *******

capture log close
log using "One vs at least two different pathogens.log", replace

local dg_name `""Multiple organisms""'
local irow 82
local prow = `irow'

                                        quietly: use "Pooled infections_trimmattu", clear
                                        drop if ses==.
                                        egen ensibactviralpvm = rowmin(ensibactinfpvm ensiviralinfpvm)
                                        gen toinenbactviralpvm = ensibactviralpvm if ensibactinfpvm==ensiviralinfpvm &
ensibactinfpvm!=.
                                        replace toinenbactviralpvm = ensibactinfpvm if ensibactinfpvm>ensiviralinfpvm &
ensibactinfpvm!=.
                                        replace toinenbactviralpvm = ensiviralinfpvm if ensiviralinfpvm>ensibactinfpvm &
ensiviralinfpvm!=.
                                        gen anybactviral = 1 if ensibactviralpvm!=.
                                        gen toinenbactviral = 1 if toinenbactviralpvm!=.

                                        quietly: stset exitpvm, id(id) failure(ensidementia) origin(time syntpvm) enter(time entrypvm)
scale(365.25)
                                        quietly: keep id anybactviral supu cohort exitpvm ensidementia syntpvm entrypvm ensi*pvm
*toinen* _st _d _origin _t _t0 ses
                                        sum ensibactviralpvm toinenbactviralpvm
                                        tab anybactviral
                                        tab toinenbactviral

                                        stsplit first_split, at(0) after(time=ensibactviralpvm)
                                        gen multiinf_timedep =0
                                        tab multiinf_timedep
                                        replace multiinf_timedep = 1 if anybactviral==1 & first_split==0
                                        tab multiinf_timedep
                                        stsplit second_split, at(0) after(time=toinenbactviralpvm)
                                        replace multiinf_timedep = 2 if toinenbactviral==1 & second_split==0
                                        tab multiinf_timedep
                                        *browse id ensibactviralpvm toinenbactviralpvm multiinf_timedep _t0 _t

                                        quietly: stdescribe
                                        quietly: local failures = `r(N_fail)'
                                        stcox i.multiinf_timedep 2.supu#cohort 2.ses#cohort 3.ses#cohort, strata(cohort)
                                        quietly: matrix m = r(table)
                                        matrix list m
                                        tempname hr1 ll1 ul1 hr2 ll2 ul2
                                        scalar `hr1' = m[1,2]
                                        scalar `ll1' = m[5,2]
                                        scalar `ul1' = m[6,2]
                                        scalar `hr2' = m[1,3]
```

```
scalar `ll2' = m[5,3]
scalar `ul2' = m[6,3]

forvalues i =1/2 {
quietly: putexcel set "Pooled results.xlsm", sheet("FIGURE sensitivity") modify
            quietly: putexcel K`irow'=(`e(N_sub)'), nformat(number)
            quietly: putexcel L`irow'=(`e(N_fail)'), nformat(number)
            quietly: putexcel E`irow'=(`hr`i''), nformat(number_d2)
            quietly: putexcel F`irow'=(`ll`i''), nformat(number_d2)
            quietly: putexcel G`irow'=(`ul`i''), nformat(number_d2)
            quietly: putexcel B`irow'=("`idg_name'")
            quietly: putexcel C`irow'=("pooled")
            quietly: putexcel D`irow'=("Different micro-organisms, infection No. `i'")
tab multiinf_timedep, matcell(x), if first_split!=-1 & second_split!=-1
matrix list x
tab multiinf_timedep ensidementia, matcell(y), if first_split!=-1 & second_split!=-1 &_d==1
matrix list y
quietly: putexcel set "Pooled results.xlsm", sheet("FIGURE sensitivity") modify
            local j = `i' + 1
            quietly: putexcel M`irow'=(x[`j',1]), nformat(number)
            quietly: putexcel N`irow'=(y[`j',1]), nformat(number)
local ++irow
local ++irow
}
*P for trend
stcox multiinf_timedep 2.supu#cohort 2.ses#cohort 3.ses#cohort, strata(cohort)
quietly: matrix n = r(table)
matrix list n
tempname p
scalar `p' = n[4,1]
quietly: putexcel set "Pooled results.xlsm", sheet("FIGURE sensitivity") modify
            quietly: putexcel Q`prow'=(`p'), nformat(number)


*Dementia occurring from year 10 onwards
local i 1
foreach idg in ensibactviral toinenbactviral {
display `i'
replace _t0 = _t0 + 10 if multiinf_timedep==0 & `i'==1
drop if _t0>=_t
gen `idg'pvm10v = `idg'pvm + round(10*365.25) if `idg'pvm<entrypvm
replace `idg'pvm10v = entrypvm if `idg'pvm10v<entrypvm
replace _t0 = (`idg'pvm10v-syntpvm)/365.25 if `idg'pvm10v!=. & multiinf_timedep==`i'
replace _t0 = _t0 + 10 if `idg'pvm10v==. & multiinf_timedep==`i'
drop if _t0>=_t
local ++i
}

quietly: stdescribe
quietly: local failures = `r(N_fail)'
stcox i.multiinf_timedep 2.supu#cohort 2.ses#cohort 3.ses#cohort, strata(cohort)
quietly: matrix m = r(table)
matrix list m
tempname hr1 ll1 ul1 hr2 ll2 ul2
scalar `hr1' = m[1,2]
scalar `ll1' = m[5,2]
scalar `ul1' = m[6,2]
scalar `hr2' = m[1,3]
```

```
				scalar `ll2' = m[5,3]
				scalar `ul2' = m[6,3]

				local irow 83
				forvalues i =1/2 {
				quietly: putexcel set "Pooled results.xlsm", sheet("FIGURE sensitivity") modify
						quietly: putexcel K`irow'=(`e(N_sub)'), nformat(number)
						quietly: putexcel L`irow'=(`e(N_fail)'), nformat(number)
						quietly: putexcel E`irow'=(`hr`i''), nformat(number_d2)
						quietly: putexcel F`irow'=(`ll`i''), nformat(number_d2)
						quietly: putexcel G`irow'=(`ul`i''), nformat(number_d2)
						quietly: putexcel B`irow'=("`idg_name'")
						quietly: putexcel C`irow'=("pooled")
						quietly: putexcel D`irow'=("Dementia occurring more than 10 years after the
hospitalisation, infection No. `i'")
				tab multiinf_timedep, matcell(x), if first_split!=-1 & second_split!=-1
				matrix list x
				tab multiinf_timedep ensidementia, matcell(y), if first_split!=-1 & second_split!=-1  &_d==1
				matrix list y
				quietly: putexcel set "Pooled results.xlsm", sheet("FIGURE sensitivity") modify
						local j = `i' + 1
						quietly: putexcel M`irow'=(x[`j',1]), nformat(number)
						quietly: putexcel N`irow'=(y[`j',1]), nformat(number)
				local ++irow
				local ++irow
				}

				*P for trend
				local ++prow
				stcox multiinf_timedep 2.supu#cohort 2.ses#cohort 3.ses#cohort, strata(cohort)
				quietly: matrix n = r(table)
				matrix list n
				tempname p
				scalar `p' = n[4,1]
				quietly: putexcel set "Pooled results.xlsm", sheet("FIGURE sensitivity") modify
						quietly: putexcel Q`prow'=(`p'), nformat(number)

log close



***** TIME-DEPENDENT ANALYSES: NUMBER OF SIMULTANEOUS INFECTIONS *******

capture log close
log using "Number of infections.log", replace

local dg infcount
local n_dg: word count `dg'
local dg_name `""No. of infections""'

				quietly: use "Pooled infections_trimmattu", clear
				merge 1:1 id using "Infection count.dta"
				tab anyinfcount
				replace anyinfcount=0 if anyanyinf==0
				recode anyinfcount 3=2 4=2
				drop if ses==.
				gen ensiinfcountpvm = ensianyinfpvm
```

```
quietly: local irow = 23
quietly: local prow = `irow'
			forvalues i=1/`n_dg' {
						preserve
						quietly: local idg : word `i' of `dg'
						quietly: local idg_name : word  `i' of `dg_name'
						display ""
						display "`idg' followed by broad dementia, adjusted for sex and education and stratified by 
cohort"
						display ""
						display "Pooled IPD-analysis, normal model and 10-year exclusion"
						display ""
						display "$S_TIME  $S_DATE"
						display ""

						quietly: stset exitpvm, id(id) failure(ensidementia) origin(time syntpvm) enter(time entrypvm) 
scale(365.25)
						quietly: keep id any`idg' supu cohort exitpvm ensidementia syntpvm entrypvm ensi`idg'pvm  _st 
_d _origin _t _t0 ses
						tab any`idg'
						stsplit `idg'_split, at(0) after(time=ensi`idg'pvm)
						tab `idg'_split
						gen `idg'_timedep =0
						replace `idg'_timedep = any`idg' if inlist(any`idg',1,2,3) & `idg'_split==0
						tab `idg'_timedep
						tab any`idg' if `idg'_split!=-1

						quietly: stdescribe
						quietly: local failures = `r(N_fail)'
						stcox i.`idg'_timedep 2.supu#cohort 2.ses#cohort 3.ses#cohort, strata(cohort)
						quietly: matrix m = r(table)
						matrix list m
						tempname hr1 ll1 ul1 hr2 ll2 ul2 hr3 ll3 ul3
						scalar `hr1' = m[1,2]
						scalar `ll1' = m[5,2]
						scalar `ul1' = m[6,2]
						scalar `hr2' = m[1,3]
						scalar `ll2' = m[5,3]
						scalar `ul2' = m[6,3]
						scalar `hr3' = m[1,4]
						scalar `ll3' = m[5,4]
						scalar `ul3' = m[6,4]

						forvalues i =1/2 {
						quietly: putexcel set "Pooled results.xlsm", sheet("FIGURE sensitivity") modify
								quietly: putexcel K`irow'=(`e(N_sub)'), nformat(number)
								quietly: putexcel L`irow'=(`e(N_fail)'), nformat(number)
								quietly: putexcel E`irow'=(`hr`i''), nformat(number_d2)
								quietly: putexcel F`irow'=(`ll`i''), nformat(number_d2)
								quietly: putexcel G`irow'=(`ul`i''), nformat(number_d2)
								quietly: putexcel B`irow'=("Simultaneous infection diagnoses")
								quietly: putexcel C`irow'=("pooled")
								quietly: putexcel D`irow'=("No. of simultaneous infection diagnoses = `i'")
						tab `idg'_timedep, matcell(x), if `idg'_split!=-1
						matrix list x
						tab `idg'_timedep ensidementia, matcell(y), if `idg'_split!=-1
						matrix list y
						quietly: putexcel set "Pooled results.xlsm", sheet("FIGURE sensitivity") modify
```

```
			local j = `i' + 1
			quietly: putexcel M`irow'=(x[`j',1]), nformat(number)
			quietly: putexcel N`irow'=(y[`j',1]), nformat(number)
local ++irow
local ++irow
}

*P for trend
stcox `idg'_timedep 2.supu#cohort 2.ses#cohort 3.ses#cohort, strata(cohort)
quietly: matrix n = r(table)
matrix list n
tempname p
scalar `p' = n[4,1]
quietly: putexcel set "Pooled results.xlsm", sheet("FIGURE sensitivity") modify
			quietly: putexcel Q`prow'=(`p'), nformat(number)

local irow 24
*Dementia occurring from year 10 onwards
gen ensi`idg'pvm10v = ensi`idg'pvm + round(10*365.25) if ensi`idg'pvm<entrypvm
replace ensi`idg'pvm10v = entrypvm if ensi`idg'pvm10v<entrypvm
replace _t0 = (ensi`idg'pvm10v-syntpvm)/365.25 if ensi`idg'pvm10v!=.
replace _t0 = _t0 + 10 if ensi`idg'pvm10v==.
drop if _t0>=_t

quietly: stdescribe
quietly: local failures = `r(N_fail)'
stcox i.`idg'_timedep 2.supu#cohort 2.ses#cohort 3.ses#cohort, strata(cohort)
quietly: matrix m = r(table)
matrix list m
tempname hr1 ll1 ul1 hr2 ll2 ul2 hr3 ll3 ul3
scalar `hr1' = m[1,2]
scalar `ll1' = m[5,2]
scalar `ul1' = m[6,2]
scalar `hr2' = m[1,3]
scalar `ll2' = m[5,3]
scalar `ul2' = m[6,3]
scalar `hr3' = m[1,4]
scalar `ll3' = m[5,4]
scalar `ul3' = m[6,4]

forvalues i =1/3 {
quietly: putexcel set "Pooled results.xlsm", sheet("FIGURE sensitivity") modify
			quietly: putexcel K`irow'=(`e(N_sub)'), nformat(number)
			quietly: putexcel L`irow'=(`e(N_fail)'), nformat(number)
			quietly: putexcel E`irow'=(`hr`i''), nformat(number_d2)
			quietly: putexcel F`irow'=(`ll`i''), nformat(number_d2)
			quietly: putexcel G`irow'=(`ul`i''), nformat(number_d2)
			quietly: putexcel B`irow'=("Simultaneous infection diagnoses")
			quietly: putexcel C`irow'=("pooled")
			quietly: putexcel D`irow'=("No. of simultaneous infection diagnoses = `i'")
tab `idg'_timedep, matcell(x), if `idg'_split!=-1
matrix list x
tab `idg'_timedep ensidementia, matcell(y), if `idg'_split!=-1
matrix list y
quietly: putexcel set "Pooled results.xlsm", sheet("FIGURE sensitivity") modify
			local j = `i' + 1
			quietly: putexcel M`irow'=(x[`j',1]), nformat(number)
			quietly: putexcel N`irow'=(y[`j',1]), nformat(number)
```

```
				local ++irow
				local ++irow
				}
				*P for trend
				local ++prow
				stcox `idg'_timedep 2.supu#cohort 2.ses#cohort 3.ses#cohort, strata(cohort)
				quietly: matrix n = r(table)
				matrix list n
				tempname p
				scalar `p' = n[4,1]
				quietly: putexcel set "Pooled results.xlsm", sheet("FIGURE sensitivity") modify
						quietly: putexcel Q`prow'=(`p'), nformat(number)

				restore
				}
log close


***** TIME-DEPENDENT ANALYSES: FIGURE sensitivity, CNS vs other infections
capture log close
log using "Sensitivity, CNS infection vs other infection.log", replace
local dg anycns noncns
local n_dg: word count `dg'
local dg_name `""Any CNS infection vs not" "Any extra-CNS infection vs not""'

				quietly: use "Pooled infections_trimmattu", clear
				drop if ses==.

quietly: local irow = 46
		forvalues i=1/`n_dg' {
				preserve
				quietly: local idg : word `i' of `dg'
				quietly: local idg_name : word  `i' of `dg_name'
				display ""
				display "`idg' followed by broad dementia, adjusted for sex and education and stratified by
cohort"
				display ""
				display "Pooled IPD-analysis, normal model and 10-year exclusion"
				display ""
				display "$S_TIME  $S_DATE"
				display ""

				quietly: stset exitpvm, id(id) failure(ensidementia) origin(time syntpvm) enter(time entrypvm)
scale(365.25)
				quietly: keep id any`idg' supu cohort exitpvm ensidementia syntpvm entrypvm ensi`idg'pvm  _st
_d _origin _t _t0 ses
				tab any`idg'
				stsplit `idg'_split, at(0) after(time=ensi`idg'pvm)
				tab `idg'_split
				gen `idg'_timedep =0
				replace `idg'_timedep = 1 if any`idg'==1 & `idg'_split==0
				tab `idg'_timedep
				tab any`idg' if `idg'_split!=-1

				quietly: stdescribe
				quietly: local failures = `r(N_fail)'
				stcox `idg'_timedep 2.supu#cohort 2.ses#cohort 3.ses#cohort, strata(cohort)
				quietly: matrix m = r(table)
```

```
tempname hr ll ul
scalar `hr' = m[1,1]
scalar `ll' = m[5,1]
scalar `ul' = m[6,1]
quietly: putexcel set "Pooled results.xlsm", sheet("FIGURE sensitivity") modify
        quietly: putexcel K`irow'=(`e(N_sub)'), nformat(number)
        quietly: putexcel L`irow'=(`e(N_fail)'), nformat(number)
        quietly: putexcel E`irow'=(`hr'), nformat(number_d2)
        quietly: putexcel F`irow'=(`ll'), nformat(number_d2)
        quietly: putexcel G`irow'=(`ul'), nformat(number_d2)
        quietly: putexcel B`irow'=("`idg'")
        quietly: putexcel C`irow'=("pooled")
        quietly: putexcel D`irow'=("`idg_name'")
tab any`idg', matcell(x), if `idg'_split!=-1
matrix list x
quietly: tab any`idg' ensidementia, matcell(y), if `idg'_split!=-1 & _d==1
matrix list y
quietly: putexcel set "Pooled results.xlsm", sheet("FIGURE sensitivity") modify
        quietly: putexcel M`irow'=(x[2,1]), nformat(number)
        quietly: putexcel N`irow'=(y[2,1]), nformat(number)
local ++irow

*Dementia occurring from year 10 onwards
gen ensi`idg'pvm10v = ensi`idg'pvm + round(10*365.25) if ensi`idg'pvm<entrypvm
replace ensi`idg'pvm10v = entrypvm if ensi`idg'pvm10v<entrypvm
replace _t0 = (ensi`idg'pvm10v-syntpvm)/365.25 if ensi`idg'pvm10v!=.
replace _t0 = _t0 + 10 if ensi`idg'pvm10v==.
drop if _t0>=_t
quietly: stdescribe
quietly: local failures = `r(N_fail)'
stcox `idg'_timedep 2.supu#cohort 2.ses#cohort 3.ses#cohort, strata(cohort)
quietly: matrix m = r(table)
scalar `hr' = m[1,1]
scalar `ll' = m[5,1]
scalar `ul' = m[6,1]
quietly: putexcel set "Pooled results.xlsm", sheet("FIGURE sensitivity") modify
        quietly: putexcel K`irow'=(`e(N_sub)'), nformat(number)
        quietly: putexcel L`irow'=(`e(N_fail)'), nformat(number)
        quietly: putexcel E`irow'=(`hr'), nformat(number_d2)
        quietly: putexcel F`irow'=(`ll'), nformat(number_d2)
        quietly: putexcel G`irow'=(`ul'), nformat(number_d2)
        quietly: putexcel B`irow'=("   `idg'_10y_exclusion")
        quietly: putexcel C`irow'=("pooled")
        quietly: putexcel D`irow'=("   Dementia occurring more than 10 years after the
hospitalisation")
tab any`idg', matcell(x), if `idg'_split!=-1
matrix list x
quietly: tab any`idg' ensidementia, matcell(y), if `idg'_split!=-1 & _d==1
matrix list y
quietly: putexcel set "Pooled results.xlsm", sheet("FIGURE sensitivity") modify
        quietly: putexcel M`irow'=(x[2,1]), nformat(number)
        quietly: putexcel N`irow'=(y[2,1]), nformat(number)
local ++irow
local ++irow
restore
}
log close
```

```
version 16

***** TIME-DEPENDENT ANALYSES: Test for difference between infections

capture log close
log using "Test for difference.log", replace


		quietly: use "Pooled infections_trimmattu", clear
		drop if ses==.

		*Intra vs extracellular
		egen ensiintranotextracpvm = rowmin(ensiintracpvm ensiextracpvm)
		replace ensiintranotextracpvm = . if ensiintracpvm==ensiextracpvm
		gen byte anyintranotextrac = 1 if ensiintracpvm==ensiintranotextracpvm & ensiintranotextracpvm!=.
		replace anyintranotextrac = 0 if ensiextracpvm==ensiintranotextracpvm & ensiintranotextracpvm!=. & 
anyintranotextrac!=1
		sum ensiintranotextracpvm
		tab anyintranotextrac
		tab anyintrac
		tab anyextrac


		*Gram+ vs gram-
		egen ensigramplusnotminuspvm = rowmin(ensigrampluspvm ensigramminuspvm)
		replace ensigramplusnotminuspvm = . if ensigrampluspvm==ensigramminuspvm
		gen byte anygramplusnotminus = 1 if ensigrampluspvm==ensigramplusnotminuspvm & 
ensigramplusnotminuspvm!=.
		replace anygramplusnotminus = 0 if ensigramminuspvm==ensigramplusnotminuspvm & 
ensigramplusnotminuspvm!=. & anygramplusnotminus!=1
		sum ensigramplusnotminuspvm
		tab anygramplusnotminus
		tab anygramplus
		tab anygramminus


		*Bacterial infection with sepsis vs without sepsis
		gen byte anybactsepsisnotsepsis = 1 if ensibactsepsispvm==ensibactinfpvm & anybactsepsis==1
		replace anybactsepsisnotsepsis = 0 if ensibact_nonsepsispvm==ensibactinfpvm & anybactsepsisnotsepsis!=1 & 
anybact_nonsepsis==1
		gen ensibactsepsisnotsepsispvm = ensibactinfpvm
		sum ensibactsepsisnotsepsispvm
		tab anybactsepsisnotsepsis
		tab anybact_nonsepsis
		tab anybactsepsis

		*CNS infection vs non cns infection
		gen byte anycnsnotcns = 1 if ensianycnspvm==ensianyinfpvm & anyanycns==1
		replace anycnsnotcns = 0 if ensinoncnspvm==ensianyinfpvm & anycnsnotcns!=1 & anynoncns==1
		gen ensicnsnotcnspvm = ensianyinfpvm
		sum ensicnsnotcnspvm
		tab anycnsnotcns
		tab anyanycns
		tab anynoncns

		*Herpesvirus vs persistent viral infection
		egen ensiherpesnotpersviralpvm = rowmin(ensiherpespvm ensipersviralpvm)
		gen byte anyherpesnotpersviral = 1 if ensiherpespvm==ensiherpesnotpersviralpvm & anyherpes==1
```

```
replace anyherpesnotpersviral = 0 if ensipersviralpvm==ensiherpesnotpersviralpvm & anyherpesnotpersviral!=1 & anypersviral==1
sum ensiherpesnotpersviralpvm
tab anyherpesnotpersviral
tab anyherpes
tab anypersviral

*Herpesvirus vs acute viral infection
egen ensiherpesnotacuteviralpvm = rowmin(ensiherpespvm ensiacuteviralpvm)
gen byte anyherpesnotacuteviral = 1 if ensiherpespvm==ensiherpesnotacuteviralpvm & anyherpes==1
replace anyherpesnotacuteviral = 0 if ensiacuteviralpvm==ensiherpesnotacuteviralpvm & anyherpesnotacuteviral!=1 & anyacuteviral==1
sum ensiherpesnotacuteviralpvm
tab anyherpesnotacuteviral
tab anyherpes
tab anyacuteviral

*Non herpes persistent vs acute viral infection
egen ensipersnotacuteviralpvm = rowmin(ensipersviralpvm ensiacuteviralpvm)
gen byte anypersnotacuteviral = 1 if ensipersviralpvm==ensipersnotacuteviralpvm & anypersviral==1
replace anypersnotacuteviral = 0 if ensiacuteviralpvm==ensipersnotacuteviralpvm & anypersnotacuteviral!=1 & anyacuteviral==1
sum ensipersnotacuteviralpvm
tab anypersnotacuteviral
tab anypersviral
tab anyacuteviral

*Bacteria vs viruses
egen ensibactnotviralpvm = rowmin(ensibactinfpvm ensiviralinfpvm)
gen byte anybactnotviral = 1 if ensibactinfpvm==ensibactnotviralpvm & anybactinf==1
replace anybactnotviral = 0 if ensiviralinfpvm==ensibactnotviralpvm & anybactnotviral!=1 & anyviralinf==1
sum ensibactnotviralpvm
tab anybactnotviral
tab anybactinf
tab anyviralinf

*Type of viral infection
egen ensivirustypepvm = rowmin(ensiherpespvm ensipersviralpvm ensiacuteviralpvm)
gen byte anyvirustype = 1 if ensiherpespvm==ensivirustypepvm & anyviralinf==1 & ensivirustypepvm!=.
replace anyvirustype = 2 if ensipersviralpvm==ensivirustypepvm & anyviralinf==1 & anyvirustype==. & ensivirustypepvm!=.
replace anyvirustype = 3 if ensiacuteviralpvm==ensivirustypepvm & anyviralinf==1 & anyvirustype==. & ensivirustypepvm!=.
tab anyvirustype
tab anyviralinf
sum ensivirustypepvm ensiviralinfpvm
*viisi tapausta, joilla on virusinfektio, mutta jonka tyyppi ei ole tiedossa.

*Any persistent viral infection vs acute viral infection
gen ensiacutenotanypersviralpvm = ensivirustypepvm
recode anyvirustype 3=1 1=0 2=0, gen(anyacutenotanypersviral)


*Invasive vs localised
egen ensisysnotlocalpvm = rowmin(ensisysbactpvm ensilocalbactpvm)
gen byte anysysnotlocal = 1 if ensisysbactpvm==ensisysnotlocalpvm & anysysbact==1
replace anysysnotlocal = 0 if ensilocalbactpvm==ensisysnotlocalpvm & anysysnotlocal!=1 & anylocalbact==1
sum ensisysnotlocalpvm
```

```
		tab anysysnotlocal
		tab anysysbact
		tab anylocalbact




local dg sysnotlocal intranotextrac gramplusnotminus bactnotviral acutenotanypersviral cnsnotcns bactsepsisnotsepsis
local n_dg: word count `dg'
local dg_name `""Invasive vs localised bacterial infection" "Intra vs extracellular bacterial infection" "Gram-positive vs gram-negative bacterial infection" "Bacterial vs viral infection" "Acute vs any persisten viral infection" "CNS vs extra-CNS infection" "Septic vs not septic bacterial infection""'
quietly: local irow = 7
		forvalues i=1/`n_dg' {
				preserve
				quietly: local idg : word `i' of `dg'
				quietly: local idg_name : word  `i' of `dg_name'
				display ""
				display "`idg' followed by broad dementia, adjusted for sex and education and stratified by cohort"
				display ""
				display "Pooled IPD-analysis"
				display ""
				display "$S_TIME  $S_DATE"
				display ""


				quietly: stset exitpvm, id(id) failure(ensidementia) origin(time syntpvm) enter(time entrypvm) scale(365.25)
				quietly: keep id any`idg' ensi`idg'pvm supu cohort exitpvm ensidementia syntpvm entrypvm ensi`idg'pvm  _st _d _origin _t _t0 ses

				gen byte anyeither = ensi`idg'pvm !=.
				tab any`idg'
				tab anyeither

				stsplit `idg'_split, at(0) after(time=ensi`idg'pvm)
				tab `idg'_split
				gen `idg'_timedep =0
				replace `idg'_timedep = 1 if anyeither==1 & `idg'_split==0
				tab `idg'_timedep
				tab anyeither if `idg'_split!=-1

				quietly: stdescribe
				quietly: local failures = `r(N_fail)'

				tab anyeither if `idg'_split!=-1
				tab `idg'_timedep
				recode `idg'_timedep 1=2 if any`idg'==0
				tab `idg'_timedep
				stcox i.`idg'_timedep 2.supu#cohort 2.ses#cohort 3.ses#cohort, strata(cohort)
				test 1.`idg'_timedep = 2.`idg'_timedep

				quietly: putexcel set "Pooled results.xlsm", sheet("FIGURE compare") modify
						quietly: putexcel K`irow'=(`e(N_sub)'), nformat(number)
						quietly: putexcel L`irow'=(`e(N_fail)'), nformat(number)
						quietly: putexcel E`irow'=(`r(p)'), nformat(number_d2)
```

```
                quietly: putexcel B`irow'=("`idg'")
                quietly: putexcel C`irow'=("pooled")
                quietly: putexcel D`irow'=("`idg_name'")
        local ++irow

        *Dementia occurring from year 10 onwards
        gen ensi`idg'pvm10v = ensi`idg'pvm + round(10*365.25) if ensi`idg'pvm<entrypvm
        replace ensi`idg'pvm10v = entrypvm if ensi`idg'pvm10v<entrypvm
        replace _t0 = (ensi`idg'pvm10v-syntpvm)/365.25 if ensi`idg'pvm10v!=.
        replace _t0 = _t0 + 10 if ensi`idg'pvm10v==.
        drop if _t0>=_t
        stdescribe
        quietly: local failures = `r(N_fail)'

        stcox i.`idg'_timedep 2.supu#cohort 2.ses#cohort 3.ses#cohort, strata(cohort)
        test 1.`idg'_timedep = 2.`idg'_timedep

        quietly: putexcel set "Pooled results.xlsm", sheet("FIGURE compare") modify
                quietly: putexcel K`irow'=(`e(N_sub)'), nformat(number)
                quietly: putexcel L`irow'=(`e(N_fail)'), nformat(number)
                quietly: putexcel E`irow'=(`r(p)'), nformat(number_d2)
                quietly: putexcel B`irow'=("`idg'")
                quietly: putexcel C`irow'=("pooled")
                quietly: putexcel D`irow'=("`idg_name' from year 10 onwards")
        local ++irow
        local ++irow
        restore
        }

    log close

*Comparing viral infections
capture log close
log using "herpes vs other persistent viral.log", replace
local dg herpesnotpersviral
local n_dg: word count `dg'
local dg_name `""Herpesvirus infection vs other potentially persistent viral infection""'
quietly: local irow = 40
    forvalues i=1/`n_dg' {
        preserve
        quietly: local idg : word `i' of `dg'
        quietly: local idg_name : word  `i' of `dg_name'
        display ""
        display "`idg' followed by broad dementia, adjusted for sex and education and stratified by
cohort"
        display ""
        display "Pooled IPD-analysis"
        display ""
        display "$S_TIME  $S_DATE"
        display ""



        quietly: stset exitpvm, id(id) failure(ensidementia) origin(time syntpvm) enter(time entrypvm)
scale(365.25)
        quietly: keep id any`idg' ensi`idg'pvm supu cohort exitpvm ensidementia syntpvm entrypvm
ensi`idg'pvm  _st _d _origin _t _t0 ses

        gen byte anyeither = ensi`idg'pvm !=.
```

```
		tab any`idg'
		tab anyeither

		stsplit `idg'_split, at(0) after(time=ensi`idg'pvm)
		tab `idg'_split
		gen `idg'_timedep =0
		replace `idg'_timedep = 1 if anyeither==1 & `idg'_split==0
		tab `idg'_timedep
		tab anyeither if `idg'_split!=-1

		quietly: stdescribe
		quietly: local failures = `r(N_fail)'

		tab anyeither if `idg'_split!=-1
		tab `idg'_timedep
		recode `idg'_timedep 1=2 if any`idg'==0
		tab `idg'_timedep
		stcox i.`idg'_timedep 2.supu#cohort 2.ses#cohort 3.ses#cohort, strata(cohort)
		test 1.`idg'_timedep = 2.`idg'_timedep

		quietly: putexcel set "Pooled results.xlsm", sheet("FIGURE compare") modify
				quietly: putexcel K`irow'=(`e(N_sub)'), nformat(number)
				quietly: putexcel L`irow'=(`e(N_fail)'), nformat(number)
				quietly: putexcel E`irow'=(`r(p)'), nformat(number_d2)
				quietly: putexcel B`irow'=("`idg'")
				quietly: putexcel C`irow'=("pooled")
				quietly: putexcel D`irow'=("`idg_name'")
		local ++irow

		*Dementia occurring from year 10 onwards
		gen ensi`idg'pvm10v = ensi`idg'pvm + round(10*365.25) if ensi`idg'pvm<entrypvm
		replace ensi`idg'pvm10v = entrypvm if ensi`idg'pvm10v<entrypvm
		replace _t0 = (ensi`idg'pvm10v-syntpvm)/365.25 if ensi`idg'pvm10v!=.
		replace _t0 = _t0 + 10 if ensi`idg'pvm10v==.
		drop if _t0>=_t
		stdescribe
		quietly: local failures = `r(N_fail)'

		stcox i.`idg'_timedep 2.supu#cohort 2.ses#cohort 3.ses#cohort, strata(cohort)
		test 1.`idg'_timedep = 2.`idg'_timedep

		quietly: putexcel set "Pooled results.xlsm", sheet("FIGURE compare") modify
				quietly: putexcel K`irow'=(`e(N_sub)'), nformat(number)
				quietly: putexcel L`irow'=(`e(N_fail)'), nformat(number)
				quietly: putexcel E`irow'=(`r(p)'), nformat(number_d2)
				quietly: putexcel B`irow'=("`idg'")
				quietly: putexcel C`irow'=("pooled")
				quietly: putexcel D`irow'=("`idg_name' from year 10 onwards")
		local ++irow
		local ++irow
		restore
		}

	log close


capture log close
log using "Types of viral infection.log", replace
```

```
local dg virustype
local n_dg: word count `dg'
local dg_name `""Types of viral infection""'
quietly: local irow = 34
			forvalues i=1/`n_dg' {
						preserve
						quietly: local idg : word `i' of `dg'
						quietly: local idg_name : word  `i' of `dg_name'
						display ""
						display "`idg' followed by broad dementia, adjusted for sex and education and stratified by 
cohort"
						display ""
						display "Pooled IPD-analysis"
						display ""
						display "$S_TIME  $S_DATE"
						display ""

						quietly: stset exitpvm, id(id) failure(ensidementia) origin(time syntpvm) enter(time entrypvm) 
scale(365.25)
						quietly: keep id any`idg' *viralinf* supu cohort exitpvm ensidementia syntpvm entrypvm 
ensi`idg'pvm  _st _d _origin _t _t0 ses
						tab any`idg'
						stsplit `idg'_split, at(0) after(time=ensi`idg'pvm)
						tab `idg'_split
						gen `idg'_timedep =0
						replace `idg'_timedep = any`idg' if inlist(any`idg',1,2,3) & `idg'_split==0
						tab `idg'_timedep
						tab any`idg' if `idg'_split!=-1

						quietly: stdescribe
						quietly: local failures = `r(N_fail)'


						if "`idg'"=="virustype" {
						local comp "viralinf"
						replace anyviralinf = . if anyvirustype==.
						}
						tab any`comp' if `idg'_split!=-1
						tab `idg'_timedep
						recode `idg'_timedep 1=2 if any`comp'==0
						tab `idg'_timedep
						stcox i.`idg'_timedep 2.supu#cohort 2.ses#cohort 3.ses#cohort, strata(cohort)
						test 1.`idg'_timedep = 2.`idg'_timedep = 3.`idg'_timedep

						quietly: putexcel set "Pooled results.xlsm", sheet("FIGURE compare") modify
									quietly: putexcel K`irow'=(`e(N_sub)'), nformat(number)
									quietly: putexcel L`irow'=(`e(N_fail)'), nformat(number)
									quietly: putexcel E`irow'=(`r(p)'), nformat(number_d2)
									quietly: putexcel B`irow'=("`comp'")
									quietly: putexcel C`irow'=("pooled")
									quietly: putexcel D`irow'=("`idg_name'")
						local ++irow

						*Dementia occurring from year 10 onwards
						gen ensi`idg'pvm10v = ensi`idg'pvm + round(10*365.25) if ensi`idg'pvm<entrypvm
						replace ensi`idg'pvm10v = entrypvm if ensi`idg'pvm10v<entrypvm
						replace _t0 = (ensi`idg'pvm10v-syntpvm)/365.25 if ensi`idg'pvm10v!=.
```

```
replace _t0 = _t0 + 10 if ensi`idg'pvm10v==.
drop if _t0>=_t
stdescribe
quietly: local failures = `r(N_fail)'

stcox i.`idg'_timedep 2.supu#cohort 2.ses#cohort 3.ses#cohort, strata(cohort)
test 1.`idg'_timedep = 2.`idg'_timedep = 3.`idg'_timedep

quietly: putexcel set "Pooled results.xlsm", sheet("FIGURE compare") modify
    quietly: putexcel K`irow'=(`e(N_sub)'), nformat(number)
    quietly: putexcel L`irow'=(`e(N_fail)'), nformat(number)
    quietly: putexcel E`irow'=(`r(p)'), nformat(number_d2)
    quietly: putexcel B`irow'=("`idg'")
    quietly: putexcel C`irow'=("pooled")
    quietly: putexcel D`irow'=("`idg_name' from year 10 onwards")
local ++irow
local ++irow
restore
}

log close



***** NUMBER OF INFECTIONS *******

capture log close
log using "P for number of simultaneous infections.log", replace

local dg infcount
local n_dg: word count `dg'
local dg_name `""No. of infections""'

quietly: use "Pooled infections_trimmattu", clear
merge 1:1 id using "Infection count.dta"
tab anyinfcount
replace anyinfcount=0 if anyanyinf==0
recode anyinfcount 3=2 4=2
drop if ses==.
gen ensiinfcountpvm = ensianyinfpvm

quietly: local irow = 37
    forvalues i=1/`n_dg' {
        preserve
        quietly: local idg : word `i' of `dg'
        quietly: local idg_name : word  `i' of `dg_name'
        display ""
        display "`idg' followed by broad dementia, adjusted for sex and education and stratified by
cohort"
        display ""
        display "Pooled IPD-analysis, normal model and 10-year exclusion"
        display ""
        display "$S_TIME  $S_DATE"
        display ""

        quietly: stset exitpvm, id(id) failure(ensidementia) origin(time syntpvm) enter(time entrypvm)
scale(365.25)
```

```
                              quietly: keep id any`idg' supu cohort exitpvm ensidementia syntpvm entrypvm ensi`idg'pvm  _st
_d _origin _t _t0 ses
                              tab any`idg'
                              stsplit `idg'_split, at(0) after(time=ensi`idg'pvm)
                              tab `idg'_split
                              gen `idg'_timedep =0
                              replace `idg'_timedep = any`idg' if inlist(any`idg',1,2,3) & `idg'_split==0
                              tab `idg'_timedep
                              tab any`idg' if `idg'_split!=-1

                              quietly: stdescribe
                              quietly: local failures = `r(N_fail)'
                              stcox i.`idg'_timedep 2.supu#cohort 2.ses#cohort 3.ses#cohort, strata(cohort)
                              test 1.`idg'_timedep = 2.`idg'_timedep

                              quietly: putexcel set "Pooled results.xlsm", sheet("FIGURE compare") modify
                                          quietly: putexcel K`irow'=(`e(N_sub)'), nformat(number)
                                          quietly: putexcel L`irow'=(`e(N_fail)'), nformat(number)
                                          quietly: putexcel E`irow'=(`r(p)'), nformat(number_d2)
                                          quietly: putexcel B`irow'=("`idg'")
                                          quietly: putexcel C`irow'=("pooled")
                                          quietly: putexcel D`irow'=("`idg_name'")
                              local ++irow

                              *Dementia occurring from year 10 onwards
                              gen ensi`idg'pvm10v = ensi`idg'pvm + round(10*365.25) if ensi`idg'pvm<entrypvm
                              replace ensi`idg'pvm10v = entrypvm if ensi`idg'pvm10v<entrypvm
                              replace _t0 = (ensi`idg'pvm10v-syntpvm)/365.25 if ensi`idg'pvm10v!=.
                              replace _t0 = _t0 + 10 if ensi`idg'pvm10v==.
                              drop if _t0>=_t

                              quietly: stdescribe
                              quietly: local failures = `r(N_fail)'
                              stcox i.`idg'_timedep 2.supu#cohort 2.ses#cohort 3.ses#cohort, strata(cohort)
                              test 1.`idg'_timedep = 2.`idg'_timedep

                              quietly: putexcel set "Pooled results.xlsm", sheet("FIGURE compare") modify
                                          quietly: putexcel K`irow'=(`e(N_sub)'), nformat(number)
                                          quietly: putexcel L`irow'=(`e(N_fail)'), nformat(number)
                                          quietly: putexcel E`irow'=(`r(p)'), nformat(number_d2)
                                          quietly: putexcel B`irow'=("`idg'")
                                          quietly: putexcel C`irow'=("pooled")
                                          quietly: putexcel D`irow'=("`idg_name' from year 10 onwards")
                              local ++irow
                              local ++irow
                              restore
                              }
log close
```

```
********** REPLICATION ANALYSIS (UK BIOBANK) ***************

version 16

capture log close
log using "Sipila, infections and dementia, UKB replication $S_DATE.log", replace


/*Generate a wide dataset (one row per participant) with the following variables:

variable name:	variable type	description

id	integer
	participant id
dementia	binary (1/0)
	Dementia during follow-up. Please use the established definition in the cohort.
dementia_AD_nonAD	binary (1/2)	Type of
dementia: 1) Alzheimer's disease 2) other dementia
dementiatype	categorical	(1,2,3,4,5,6)	Type of first dementia diagnosis: 1) Alzheimer's
disease, 2) Frontotemporal dementia, 3) Parkinson's disease dementia, 4) Vascular dementia 5) Other specified dementia, 6)
Unspecified dementia
exitdate	date
	First of the following: date of first dementia diagnosis, date of death, end of follow-up
(dementia diagnoses that occur before study entry should be included if such data are available)
birthday	date
	Date of Birth
entrydate	date
	entry
sex	binary (1/2)
	Sex
ses	categorical	(1,2,3)
	Socioeconomic status or education (low, intermediate, high)
alcocl	categorical	(0,1,2,3)
	non-drinkers, moderate drinkers, intermediate drinkers, and heavy drinkers
smoking	categorical	(1,2,3)
	never smokers, ex-smokers, current smokers
bmi_who	categorical	(0,25,30)
	normal (≤24.9 kg/m2), overweight (25.0-29.9 kg/m2), obese (≥30.0 kg/m2).
hypertensiondate	date
	date of hypertension diagnosis
diabetesdate	date
	date of diabetes diagnosis

*/

*Store the data to Stata format
*Please record all definitions of variables if they differ from those suggested above.



*Trim the data

*load data

count
drop if exitdate<=entrydate
```

```
count
drop if ses==.
count

tab dementia
recode dementia .=0
tab dementia

ren anycnsdate ensianycnspvm
ren noncnsdate ensianynoncnspvm

ren anycns anyanycns
ren noncns anyanynoncns

order ensi* any*

foreach idg in anyinf bactinf viralinf hiv anycns anynoncns {
replace ensi`idg'pvm=. if ensi`idg'pvm>=exitdate
replace any`idg' = 0 if ensi`idg'pvm==.
sum ensi`idg'pvm
tab any`idg'
}

ren anyanynoncns anynoncns
ren ensianynoncnspvm ensinoncnspvm

*Create variables that have indentical names with those used in the primary analysis

capture gen diabetes_combpvm = diabetesdate
capture gen hypert_combpvm = hypertensiondate
capture gen ihd_combpvm = ihddate
capture gen cereb_combpvm = cerebdate
capture gen parkinson_combpvm = parkinsondate
capture gen tupakka = smoking
*alcocl does not change
*ses does not change
capture gen entrypvm = entrydate
capture gen exitpvm = exitdate
capture gen ensidementia = dementia
recode ensidementia 0=.
capture gen syntpvm = birthday
capture gen supu = sex
capture gen dementiatyyppi_laaja = dementiatype
capture gen byte cohort==1



*save data

*** Lag periods ***

* Random lag periods for those without an infection drawn from the distribution of the lag periods of those with an infection.

*load data

version 16

*Random order
```

```
*seed from random.org from range 1 - 1 000 000 000
set rng mt64s
set rngstream 1
set seed 421100945

gen double shuffle1 = runiform()
gen double shuffle2 = runiform()

sort shuffle1 shuffle2

*New pseudo-id-variable that preserves the random order
gen shuffle_id = _n

*Delay between study entry and time of infection
gen entry_to_inf = (ensianyinfpvm-entrypvm)/365.25
replace entry_to_inf = 0 if entry_to_inf < 0
bysort supu: sum entry_to_inf if entry_to_inf>0
sum entry_to_inf
capture drop entry_to_inf_cut
egen entry_to_inf_cut = cut(entry_to_inf), at(0 0.0009765625 2.5 5 7.5 100)
tab entry_to_inf_cut
by supu: tab entry_to_inf_cut
capture drop age
gen age = (entrypvm-syntpvm)/365.25
capture drop age_cut
egen age_cut = cut(age), at(18 50 60 100)
tab age_cut
bysort supu age_cut: tab entry_to_inf_cut

levelsof age_cut, local(age_groups)
levelsof supu, local(sexes)
levelsof entry_to_inf_cut, local(entry_lags)
local i 0
			foreach age of local age_groups {
						foreach sex of local sexes {
									local i 0
									foreach lag of local entry_lags {
												local ++i

												quietly: sum entry_to_inf if age_cut==`age' & supu==`sex' &
entry_to_inf_cut==`lag'

												if `i'==1 & `r(N)' == 0 {
												matrix mean_lag_`age'_`sex' = 0
												}
												else if `i'==1 {
												matrix mean_lag_`age'_`sex' = `r(mean)'
												}
												else if `r(N)' == 0 {
												matrix mean_lag_`age'_`sex' = mean_lag_`age'_`sex' \ 0
												}
												else {
												matrix mean_lag_`age'_`sex' = mean_lag_`age'_`sex' \ `r(mean)'
												}
												}
												matrix list mean_lag_`age'_`sex'

												}
```

```
}

*Return random order
capture drop sub_order
bysort age_cut supu anyanyinf (shuffle1 shuffle2): gen sub_order = _n

*Create a similar distribution of delays between study entry and start of follow up for those without infections as with those with infection
capture drop entry_lag
gen entry_lag = .
levelsof age_cut, local(age_groups)
levelsof supu, local(sexes)
levelsof entry_to_inf_cut, local(entry_lags)
        foreach age of local age_groups {
                foreach sex of local sexes {
                        local i 0
                        local cum = 0
                        sum id if age_cut==`age' & supu==`sex' & anyanyinf==0

                        local N_control = `r(N)'
                        sum entry_to_inf_cut if age_cut==`age' & supu==`sex' & anyanyinf==1

                        local N_`age'_`sex' = `r(N)'
                        foreach lag of local entry_lags {
                                di "age_group `age', sex `sex'"
                                local ++i
                                di `i'
                                sum entry_to_inf_cut if age_cut==`age' & supu==`sex' &
anyanyinf==1 & entry_to_inf_cut==`lag'
                                local cum = `cum' + `r(N)'
                                di `cum'
                                di `N_`age'_`sex''
                                di `N_control'
                                di mean_lag_`age'_`sex'[`i',1]
                                replace entry_lag = mean_lag_`age'_`sex'[`i',1] if ///
                                age_cut==`age' & supu==`sex' & anyanyinf==0 & entry_lag==. &
sub_order <= round((`cum' / `N_`age'_`sex'')*`N_control')
                                }
                                replace entry_lag = 0 if `N_`age'_`sex''==0 & entry_lag==. &
anyanyinf==0 & supu==`sex'
                                }
                                }

*For each infection variable, code it to be missing if the person has some other infection
local dg anyinf
foreach idg of local dg {
                tab any`idg'
                egen lag_entry_`idg' = rowmax(entrypvm ensi`idg'pvm) if any`idg'==1
                replace lag_entry_`idg' = entrypvm + round(entry_lag*365.25) if any`idg'==0
                sum lag_entry_`idg'
                }

local dg bactinf viralinf anycns noncns
foreach idg of local dg {
                tab any`idg'
                replace any`idg' = . if any`idg'!=1
                replace any`idg' = 0 if anyanyinf==0 & any`idg'!=1
                tab any`idg'
```

```
				egen lag_entry_`idg' = rowmax(entrypvm ensi`idg'pvm) if any`idg'==1
				replace lag_entry_`idg' = entrypvm + round(entry_lag*365.25) if any`idg'==0
				sum lag_entry_`idg'
				}


sum entry_lag
sum entry_to_inf
bysort supu age_cut: sum entry_lag entry_to_inf

save "UKB_lag.dta", replace




**** Analysis *****

use "UKB_lag.dta", clear


*Table 1


*Instal baselinetable package
capture net install st0524_1

preserve
stset exitdate, id(id) failure(dementia) origin(time birthday) enter(time lag_entry_anyinf) scale(365.25)
		keep if _st==1 & ses!=.
		keep if anyanyinf!=.
		count
		count if ensidementia==1

				capture label define sex 1"Men" 2"Women"
				label values sex sex

				capture gen followup = _t-_t0

				capture gen age_entry = (lag_entry_anyinf - syntpvm)/365.25
				capture egen age_entry_cat = cut(age_entry), at(18,40,50,60,100)
				tab age_entry_cat
				sum age_entry, de
				sum age_entry, de, if supu==1
				sum age_entry, de, if supu==2

				label define alcocl 0 "Non-drinker" 1 "Moderate" 2 "Intermediate" 3 "Heavy"
				label values alcocl alcocl

				foreach cm in hypert diabetes ihd cereb parkinson {
						capture gen `cm'_entry= `cm'_combpvm<=lag_entry_anyinf
						capture tab `cm'_entry
						}

				capture gen age_at_dementia = _t if _d==1

				stdescribe
				sum _t, de
				sum _t if _d==1, de
```

```
baselinetable ///
age_entry_cat(novarlabel afterhead("Age at entry, years")) ///
age_entry(cts novarlabel afterhead("Age at entry (IQR), years")) ///
sex(novarlabel afterhead("Sex")) ///
ses(novarlabel afterhead("Education/Socioeconomic status")) ///
hypert_entry(novarlabel afterhead("hypertensionn")) ///
diabetes_entry(novarlabel afterhead("Diabetes mellitus")) ///
ihd_entry(novarlabel afterhead("Ischaemic heart disease")) ///
cereb_entry(novarlabel afterhead("Cerebrovascular disease")) ///
parkinson_entry(novarlabel afterhead("Parkinson disease")) ///
smoking(varlabel afterhead("Smoking status")) ///
alcocl(novarlabel afterhead("Alcohol drinking")) ///
bmi_who(novarlabel afterhead("Body mass index")) ///
apoe(novarlabel afterhead("apolipoprotein E genotype")) ///
followup(cts novarlabel afterhead("Follow-up, median (IQR), years")) ///
dementia(novarlabel afterhead("Dementia")) ///
age_at_dementia(cts novarlabel afterhead("Age at dementia, median (IQR), years")) ///
, ctsvartab(p50 (p25-p75)) missing ///
exportexcel(SipilaUKBtable1, cell(A6) replace)
restore

*FIGURE 3
local dg anyinf bactinf viralinf
local n_dg: word count `dg'
local dg_name `""Any infectious disease" "Bacterial infection" "Viral infection""'
quietly: local irow = 7

forvalues i=1/`n_dg' {
quietly: local idg : word `i' of `dg'
quietly: local idg_name : word  `i' of `dg_name'
display ""
display "`idg' , adjusted for sex and education and other variables"
display ""
display "UKB replication analysis"
display ""
display "$S_TIME  $S_DATE"
display ""

/* Models
MODEL 1: adjusted for age (as the time scale), sex and ses
MODEL 2 = model 1 + those with HIV excluded
MODEL 3 = model 2 + those with missing data on alcohol, smoking, and bmi excluded
MODEL 4 = model 3 + adjusted for alcohol, smoking, bmim hypertension, diabetes
MODEL 5 = model 4 + those with data on apoe4 missing dropped
MODEL 6 = model 5 + adjusted for apoe4 genotype
*/

forvalues j=1/6 {

preserve

stset exitpvm, id(id) failure(ensidementia) origin(time syntpvm) enter(time lag_entry_`idg')
scale(365.25)
tab any`idg'
tab any`idg' _d
sum diabetes_combpvm
```

```
replace diabetes_combpvm = . if diabetes_combpvm>=exitpvm
sum diabetes_combpvm
gen byte dm=0
replace dm = 1 if diabetes_combpvm <= lag_entry_`idg'
tab dm
sum hypert_combpvm
replace hypert_combpvm = . if hypert_combpvm>=exitpvm
sum hypert_combpvm
gen byte htn = 0
replace htn = 1 if hypert_combpvm <= lag_entry_`idg'
tab htn


if `j'==1 {
di ""
di "MODEL 1: adjusted for age (as the time scale), sex and ses"
di ""
stdescribe
quietly: local failures = `r(N_fail)'
stcox any`idg' i.supu i.ses
}
else if `j'==2 {
di ""
di "MODEL 2 = model 1 + those with HIV excluded"
di ""
drop if anyhiv==1
stdescribe
quietly: local failures = `r(N_fail)'
stcox any`idg' i.supu i.ses
}
else if `j'==3 {
di ""
di "MODEL 3 = model 2 + those with missing data on alcohol, smoking, and bmi excluded"
di ""
drop if anyhiv==1
drop if tupakka==.
drop if alcocl==.
drop if bmi_who==.
stdescribe
quietly: local failures = `r(N_fail)'
stcox any`idg' i.supu i.ses
}
else if `j'==4 {
di ""
di "MODEL 4 = model 3 + adjusted for alcohol, smoking, bmim hypertension, diabetes (please note that this is model 3 in the published manuscript)"
di ""
drop if anyhiv==1
drop if tupakka==.
drop if alcocl==.
drop if bmi_who==.
stdescribe
quietly: local failures = `r(N_fail)'
stcox any`idg' i.supu i.ses i.tupakka i.alcocl i.bmi_who i.htn i.dm
}
else if `j'==5 {
di ""
di "MODEL 5 = model 4 + those with data on apoe4 missing dropped"
```

```
di ""
drop if anyhiv==1
drop if tupakka==.
drop if alcocl==.
drop if bmi_who==.
drop if apoe==.
stdescribe
quietly: local failures = `r(N_fail)'
stcox any`idg' i.supu i.ses i.tupakka i.alcocl i.bmi_who i.htn i.dm
}
else if `j'==6 {
di ""
di "MODEL 6 = model 5 + adjusted for apoe4 genotype (please note that this is model 4 in the published manuscript)"
di ""
drop if anyhiv==1
drop if tupakka==.
drop if alcocl==.
drop if bmi_who==.
drop if apoe==.
stdescribe
quietly: local failures = `r(N_fail)'
stcox any`idg' i.supu i.ses i.tupakka i.alcocl i.bmi_who i.htn i.dm i.apoe
}
quietly: matrix m = r(table)
tempname hr ll ul
scalar `hr' = m[1,1]
scalar `ll' = m[5,1]
scalar `ul' = m[6,1]
quietly: putexcel set "SipilaUKB_$S_DATE.xlsm", sheet("FIGURE UKB") modify
        quietly: putexcel K`irow'=(`e(N_sub)'), nformat(number)
        quietly: putexcel L`irow'=(`e(N_fail)'), nformat(number)
        quietly: putexcel E`irow'=(`hr'), nformat(number_d2)
        quietly: putexcel F`irow'=(`ll'), nformat(number_d2)
        quietly: putexcel G`irow'=(`ul'), nformat(number_d2)
        quietly: putexcel B`irow'=("`idg'")
        quietly: putexcel C`irow'=("UKB")
        quietly: putexcel D`irow'=("`idg_name' model `j'")
tab any`idg', matcell(x), if _st==1
matrix list x
quietly: tab any`idg' ensidementia, matcell(y), if _d==1
matrix list y
quietly: putexcel set "SipilaUKB_$S_DATE.xlsm", sheet("FIGURE UKB") modify
        quietly: putexcel M`irow'=(x[2,1]), nformat(number)
        quietly: putexcel N`irow'=(y[2,1]), nformat(number)
local ++irow
restore
}
local ++irow
}
```

```
**** Analysis by dementia subtypes *****

local dg anyinf bactinf viralinf
local n_dg: word count `dg'
```

```
local dg_name `""Any infectious disease" "Bacterial infection" "Viral infection""'

foreach outcome in AD VD FTD PD {
		if "`outcome'" == "AD" {
		local type=1
		}
		else if "`outcome'" == "VD" {
		local type=4
		}
		else if "`outcome'" == "FTD" {
		local type=2
		}
		else if "`outcome'" == "PD" {
		local type=3
		}

		quietly: local irow = 7

		forvalues i=1/`n_dg' {
				quietly: local idg : word `i' of `dg'
				quietly: local idg_name : word  `i' of `dg_name'
				display ""
				display "`idg' , adjusted for sex and education and other variables"
				display ""
				display "UKB replication analysis"
				display ""
				display "$S_TIME  $S_DATE"
				display ""

				/* Models
				MODEL 1: adjusted for age (as the time scale), sex and ses
				MODEL 2 = model 1 + those with HIV excluded
				MODEL 3 = model 2 + those with missing data on alcohol, smoking, and bmi excluded
				MODEL 4 = model 3 + adjusted for alcohol, smoking, bmim hypertension, diabetes (please note
that this is model 3 in the published manuscript)
				MODEL 5 = model 4 + those with data on apoe4 missing dropped
				MODEL 6 = model 5 + adjusted for apoe4 genotype (please note that this is model 4 in the
published manuscript)
				*/

		forvalues j=1/6 {

				preserve

				stset exitpvm, id(id) failure(dementiatype==`type') origin(time syntpvm) enter(time
lag_entry_`idg') scale(365.25)
				tab any`idg'
				tab any`idg' _d
				sum diabetes_combpvm
				replace diabetes_combpvm = . if diabetes_combpvm>=exitpvm
				sum diabetes_combpvm
				gen byte dm = 0
				replace dm = 1 if diabetes_combpvm <= lag_entry_`idg'
				tab dm
				sum hypert_combpvm
				replace hypert_combpvm = . if hypert_combpvm>=exitpvm
				sum hypert_combpvm
				gen byte htn = 0
```

```
replace htn = 1 if hypert_combpvm <= lag_entry_`idg'
tab htn

if `j'==1 {
di ""
di "MODEL 1: adjusted for age (as the time scale), sex and ses"
di ""
stdescribe
quietly: local failures = `r(N_fail)'
stcox any`idg' i.supu i.ses
}
else if `j'==2 {
di ""
di "MODEL 2 = model 1 + those with HIV excluded"
di ""
drop if anyhiv==1
stdescribe
quietly: local failures = `r(N_fail)'
stcox any`idg' i.supu i.ses
}
else if `j'==3 {
di ""
di "MODEL 3 = model 2 + those with missing data on alcohol, smoking, and bmi excluded"
di ""
drop if anyhiv==1
drop if tupakka==.
drop if alcocl==.
drop if bmi_who==.
stdescribe
quietly: local failures = `r(N_fail)'
stcox any`idg' i.supu i.ses
}
else if `j'==4 {
di ""
di "MODEL 4 = model 3 + adjusted for alcohol, smoking, bmim hypertension, diabetes (please
note that this is model 3 in the published manuscript)"
di ""
drop if anyhiv==1
drop if tupakka==.
drop if alcocl==.
drop if bmi_who==.
stdescribe
quietly: local failures = `r(N_fail)'
stcox any`idg' i.supu i.ses i.tupakka i.alcocl i.bmi_who i.htn i.dm
}
else if `j'==5 {
di ""
di "MODEL 5 = model 4 + those with data on apoe4 missing dropped"
di ""
drop if anyhiv==1
drop if tupakka==.
drop if alcocl==.
drop if bmi_who==.
drop if apoe==.
stdescribe
quietly: local failures = `r(N_fail)'
stcox any`idg' i.supu i.ses i.tupakka i.alcocl i.bmi_who i.htn i.dm
}
```

```
else if `j'==6 {
di ""
di "MODEL 6 = model 5 + adjusted for apoe4 genotype (please note that this is model 4 in the published manuscript)"
di ""
drop if anyhiv==1
drop if tupakka==.
drop if alcocl==.
drop if bmi_who==.
drop if apoe==.
stdescribe
quietly: local failures = `r(N_fail)'
stcox any`idg' i.supu i.ses i.tupakka i.alcocl i.bmi_who i.htn i.dm i.apoe
}
quietly: matrix m = r(table)
tempname hr ll ul
scalar `hr' = m[1,1]
scalar `ll' = m[5,1]
scalar `ul' = m[6,1]
quietly: putexcel set "SipilaUKB_$S_DATE.xlsm", sheet("FIGURE UKB, `outcome'") modify
	quietly: putexcel K`irow'=(`e(N_sub)'), nformat(number)
	quietly: putexcel L`irow'=(`e(N_fail)'), nformat(number)
	quietly: putexcel E`irow'=(`hr'), nformat(number_d2)
	quietly: putexcel F`irow'=(`ll'), nformat(number_d2)
	quietly: putexcel G`irow'=(`ul'), nformat(number_d2)
	quietly: putexcel B`irow'=("`idg'")
	quietly: putexcel C`irow'="UKB"
	quietly: putexcel D`irow'=("`idg_name' model `j'")
tab any`idg', matcell(x), if _st==1
matrix list x
quietly: tab any`idg' ensidementia, matcell(y), if _d==1
matrix list y
quietly: putexcel set "SipilaUKB_$S_DATE.xlsm", sheet("FIGURE UKB, `outcome'") modify
	quietly: putexcel M`irow'=(x[2,1]), nformat(number)
	quietly: putexcel N`irow'=(y[2,1]), nformat(number)
local ++irow
restore
}
local ++irow
}
}

log close


capture log close
log using "Sipila, UKB infection burden $S_DATE.log", replace

*Trim the data
use "UKB_lag.dta", clear
count
drop if exitdate<=entrydate
count
drop if ses==.
count

tab dementia
recode dementia .=0
```

```
tab dementia

foreach idg in anyinf bactinf viralinf hiv anycns noncns {
replace ensi`idg'pvm=. if ensi`idg'pvm>=exitdate
replace any`idg' = 0 if ensi`idg'pvm==.
replace any`idg' = . if ensi`idg'pvm==. & anyanyinf==1
sum ensi`idg'pvm
tab any`idg'
}

*Create variables that have identical names with those used in the primary analysis

capture gen diabetes_combpvm = diabetesdate
capture gen hypert_combpvm = hypertensiondate
capture gen tupakka = smoking
*alcocl does not change
*ses does not change
capture gen entrypvm = entrydate
capture gen exitpvm = exitdate
capture gen ensidementia = dementia
capture recode ensidementia 0=.
capture gen syntpvm = birthday
capture gen supu = sex
*dementiatype does not change

capture drop ensiinfcountpvm
gen ensiinfcountpvm = ensianyinfpvm


save "UKB_lag.dta", replace


**** Analysis *****


***** SINGLE VS MULTIPLE INFECTIONS *******

replace toinenanyinf=0 if toinenanyinf==.
replace kolmasanyinf=0 if kolmasanyinf==.

gen cohort=1

count
tab toinenanyinf, mis
tab kolmasanyinf, mis

tab anyanyinf if ensianyinfpvm<exitpvm & ensianyinfpvm < ensitoinenanyinfpvm
tab toinenanyinf if ensitoinenanyinfpvm<exitpvm & ensitoinenanyinfpvm < ensikolmasanyinfpvm
tab kolmasanyinf if ensikolmasanyinfpvm<exitpvm

tab anyanyinf if ensianyinfpvm<exitpvm & ensianyinfpvm < ensitoinenanyinfpvm & ensitoinenanyinfpvm>=exitpvm
tab toinenanyinf if ensitoinenanyinfpvm<exitpvm & ensitoinenanyinfpvm < ensikolmasanyinfpvm  &
ensikolmasanyinfpvm>=exitpvm
tab kolmasanyinf if ensikolmasanyinfpvm<exitpvm

tab ensidementia if ensianyinfpvm<exitpvm & ensitoinenanyinfpvm>=exitpvm
tab ensidementia if ensitoinenanyinfpvm<exitpvm & ensikolmasanyinfpvm>=exitpvm
tab ensidementia if ensikolmasanyinfpvm<exitpvm
```

```
sum ensianyinfpvm
replace ensianyinfpvm = . if ensianyinfpvm>=exitpvm
sum ensianyinfpvm

sum ensitoinenanyinfpvm
replace ensitoinenanyinfpvm = . if ensitoinenanyinfpvm>=exitpvm
sum ensitoinenanyinfpvm

sum ensikolmasanyinfpvm
replace ensikolmasanyinfpvm = . if ensikolmasanyinfpvm>=exitpvm
sum ensikolmasanyinfpvm

replace toinenanyinf = . if ensitoinenanyinfpvm==.
replace kolmasanyinf = . if ensikolmasanyinfpvm==.

local dg_name `""Multiple infections""'
local irow 16
local prow = `irow'
                    preserve

                    egen lag_entry_multiinf = rowmax(lag_entry_anyinf ensitoinenanyinfpvm ensikolmasanyinfpvm)
                    egen multiinfpvm = rowmax(ensianyinfpvm ensitoinenanyinfpvm ensikolmasanyinfpvm)

                    stset exitpvm, id(id) failure(ensidementia) origin(time syntpvm) enter(time lag_entry_multiinf)
scale(365.25)

                    gen byte multiinf = 0
                    tab multiinf
                    replace multiinf = 1 if anyanyinf==1
                    tab multiinf
                    replace multiinf = 2 if ensitoinenanyinfpvm!=.
                    replace multiinf = 3 if ensikolmasanyinfpvm!=.
                    tab multiinf

                    stdescribe
                    tempname hr1 ll1 ul1 hr2 ll2 ul2 hr3 ll3 ul3 N1 N2 N3 fail1 fail2 fail3
                    forvalues i =1/3 {
                    stcox i.multiinf 2.supu#cohort 2.ses#cohort 3.ses#cohort, strata(cohort), if inlist(multiinf,0,`i')
                    quietly: matrix m = r(table)
                    matrix list m
                    scalar `N`i'' = e(N_sub)
                    scalar `fail`i'' = e(N_fail)
                    scalar `hr`i'' = m[1,2]
                    scalar `ll`i'' = m[5,2]
                    scalar `ul`i'' = m[6,2]
                    }

                    forvalues i =1/3 {
                    quietly: putexcel set "SipilaUKB_$S_DATE.xlsm", sheet("FIGURE sensitivity dist") modify
                              quietly: putexcel K`irow'=(`N`i''), nformat(number)
                              quietly: putexcel L`irow'=(`fail`i''), nformat(number)
                              quietly: putexcel E`irow'=(`hr`i''), nformat(number_d2)
                              quietly: putexcel F`irow'=(`ll`i''), nformat(number_d2)
                              quietly: putexcel G`irow'=(`ul`i''), nformat(number_d2)
                              quietly: putexcel B`irow'=("Multiinf")
                              quietly: putexcel C`irow'=("UKB")
                              quietly: putexcel D`irow'=("Single vs multiple infections, infection No. `i'")
```

```
tab multiinf, matcell(x), if inlist(multiinf,0,`i') & _st==1
matrix list x
tab multiinf ensidementia, matcell(y), if inlist(multiinf,0,`i') & _d==1
matrix list y
quietly: putexcel set "SipilaUKB_$S_DATE.xlsm", sheet("FIGURE sensitivity dist") modify
        quietly: putexcel M`irow'=(x[2,1]), nformat(number)
        quietly: putexcel N`irow'=(y[2,1]), nformat(number)
local ++irow
local ++irow
}

*P for trend
stcox multiinf i.supu i.ses
quietly: matrix n = r(table)
matrix list n
tempname p
scalar `p' = n[4,1]
quietly: putexcel set "SipilaUKB_$S_DATE.xlsm", sheet("FIGURE sensitivity dist") modify
        quietly: putexcel O`prow'=(`p'), nformat(number_d4)


restore


***** One or several different pathogens *******

local dg_name `""Multiple organisms""'
local idg_name `""Multiple_organisms""'
local irow 37
local prow = `irow'

preserve
drop if ses==.
egen ensibactviralpvm = rowmin(ensibactinfpvm ensiviralinfpvm)
gen toinenbactviralpvm = ensibactviralpvm if ensibactinfpvm==ensiviralinfpvm &
ensibactinfpvm!=.
replace toinenbactviralpvm = ensibactinfpvm if ensibactinfpvm>ensiviralinfpvm &
ensibactinfpvm!=.
replace toinenbactviralpvm = ensiviralinfpvm if ensiviralinfpvm>ensibactinfpvm &
ensiviralinfpvm!=.
gen anybactviral = 1 if ensibactviralpvm!=.
gen toinenbactviral = 1 if toinenbactviralpvm!=.
egen lag_entry_bactviral = rowmax(lag_entry_anyinf ensibactviralpvm toinenbactviralpvm)

stset exitpvm, id(id) failure(ensidementia) origin(time syntpvm) enter(time lag_entry_bactviral)
scale(365.25)
quietly: keep id anybactviral supu cohort exitpvm ensidementia syntpvm entrypvm ensi*pvm
*toinen* _st _d _origin _t _t0 ses anyanyinf
sum ensibactviralpvm toinenbactviralpvm
tab anybactviral if _st==1
tab toinenbactviral if _st==1

gen byte multiinf = 0 if anyanyinf==0
tab multiinf
tab multiinf if _st==1
replace multiinf = 1 if anybactviral==1
tab multiinf
tab multiinf if _st==1
```

```
                                        replace multiinf = 2 if toinenbactviral==1
                                        tab multiinf
                                        tab multiinf if _st==1

                                        quietly: stdescribe
                                        stcox i.multiinf 2.supu#cohort 2.ses#cohort 3.ses#cohort, strata(cohort), if multiinf!=2
                                        quietly: matrix m = r(table)
                                        matrix list m
                                        tempname hr1 ll1 ul1 hr2 ll2 ul2 N1 fail1 N2 fail2
                                        scalar `hr1' = m[1,2]
                                        scalar `ll1' = m[5,2]
                                        scalar `ul1' = m[6,2]
                                        scalar `N1' = e(N_sub)
                                        scalar `fail1' = e(N_fail)
                                        stcox i.multiinf 2.supu#cohort 2.ses#cohort 3.ses#cohort, strata(cohort), if multiinf!=1
                                        quietly: matrix m = r(table)
                                        matrix list m
                                        scalar `hr2' = m[1,2]
                                        scalar `ll2' = m[5,2]
                                        scalar `ul2' = m[6,2]
                                        scalar `N2' = e(N_sub)
                                        scalar `fail2' = e(N_fail)

                                        forvalues i =1/2 {
                                        quietly: putexcel set "SipilaUKB_$S_DATE.xlsm", sheet("FIGURE sensitivity dist") modify
                                                        quietly: putexcel K`irow'=(`N`i''), nformat(number)
                                                        quietly: putexcel L`irow'=(`fail`i''), nformat(number)
                                                        quietly: putexcel E`irow'=(`hr`i''), nformat(number_d2)
                                                        quietly: putexcel F`irow'=(`ll`i''), nformat(number_d2)
                                                        quietly: putexcel G`irow'=(`ul`i''), nformat(number_d2)
                                                        quietly: putexcel B`irow'=("`idg_name'")
                                                        quietly: putexcel C`irow'="UKB"
                                                        quietly: putexcel D`irow'=("Different micro-organisms, infection No. `i'")
                                        tab multiinf, matcell(x), if _st==1 & inlist(multiinf,0,`i')
                                        matrix list x
                                        tab multiinf ensidementia, matcell(y), if _d==1 & inlist(multiinf,0,`i')
                                        matrix list y
                                        quietly: putexcel set "SipilaUKB_$S_DATE.xlsm", sheet("FIGURE sensitivity dist") modify
                                                        quietly: putexcel M`irow'=(x[2,1]), nformat(number)
                                                        quietly: putexcel N`irow'=(y[2,1]), nformat(number)
                                        local ++irow
                                        local ++irow
                                        }
                                        *P for trend
                                        stcox multiinf 2.supu#cohort 2.ses#cohort 3.ses#cohort, strata(cohort)
                                        quietly: matrix n = r(table)
                                        matrix list n
                                        tempname p
                                        scalar `p' = n[4,1]
                                        quietly: putexcel set "SipilaUKB_$S_DATE.xlsm", sheet("FIGURE sensitivity dist") modify
                                                        quietly: putexcel O`prow'=(`p'), nformat(number_d4)

                                        restore

***** NUMBER OF SIMULTANEOUS INFECTIONS *******

count
tab anyinfcount, mis
```

```
tab anyinfcount ensidementia, mis
replace anyinfcount = . if anyanyinf!=0 & ensianyinfpvm>=exitpvm
replace anyinfcount = 0 if anyanyinf==0
replace anyinfcount = 2 if anyinfcount > 2 & anyinfcount < .
tab anyinfcount, mis
tab anyinfcount ensidementia, mis

local dg infcount
local n_dg: word count `dg'
local dg_name `""No. of simultaneous infections""'

quietly: local irow = 23
local prow = `irow'
			forvalues i=1/`n_dg' {
						preserve
						quietly: local idg : word `i' of `dg'
						quietly: local idg_name : word  `i' of `dg_name'
						display ""
						display "`idg' followed by broad dementia, adjusted for sex and education"
						display ""
						display "Pooled IPD-analysis, normal model"
						display ""
						display "$S_TIME  $S_DATE"
						display ""

						stset exitpvm, id(id) failure(ensidementia) origin(time syntpvm) enter(time lag_entry_anyinf)
scale(365.25)

						tab any`idg'
						tab any`idg' _d

						quietly: stdescribe
						quietly: local failures = `r(N_fail)'
						stcox i.any`idg' 2.supu#cohort 2.ses#cohort 3.ses#cohort, strata(cohort), if any`idg'!=2
						quietly: matrix m = r(table)
						matrix list m
						tempname hr1 ll1 ul1 hr2 ll2 ul2 N1 fail1 N2 fail2
						scalar `hr1' = m[1,2]
						scalar `ll1' = m[5,2]
						scalar `ul1' = m[6,2]
						scalar `N1' = e(N_sub)
						scalar `fail1' = e(N_fail)
						stcox i.any`idg' 2.supu#cohort 2.ses#cohort 3.ses#cohort, strata(cohort), if any`idg'!=1
						quietly: matrix m = r(table)
						matrix list m
						scalar `hr2' = m[1,2]
						scalar `ll2' = m[5,2]
						scalar `ul2' = m[6,2]
						scalar `N2' = e(N_sub)
						scalar `fail2' = e(N_fail)

						forvalues i =1/2 {
						quietly: putexcel set "SipilaUKB_$S_DATE.xlsm", sheet("FIGURE sensitivity dist") modify
								quietly: putexcel K`irow'=(`N`i''), nformat(number)
								quietly: putexcel L`irow'=(`fail`i''), nformat(number)
								quietly: putexcel E`irow'=(`hr`i''), nformat(number_d2)
								quietly: putexcel F`irow'=(`ll`i''), nformat(number_d2)
								quietly: putexcel G`irow'=(`ul`i''), nformat(number_d2)
```

```
                quietly: putexcel B`irow'=("Simultaneous infection diagnoses")
                quietly: putexcel C`irow'=("UKB")
                quietly: putexcel D`irow'=("No. of simultaneous infection diagnoses = `i'")
        tab any`idg', matcell(x), if _st==1
        matrix list x
        tab any`idg' ensidementia, matcell(y), if _d==1
        matrix list y
        quietly: putexcel set "SipilaUKB_$S_DATE.xlsm", sheet("FIGURE sensitivity dist") modify
                local j = `i' + 1
                quietly: putexcel M`irow'=(x[`j',1]), nformat(number)
                quietly: putexcel N`irow'=(y[`j',1]), nformat(number)
        local ++irow
        local ++irow
        }

        *P for trend
        stcox any`idg' i.supu i.ses
        quietly: matrix n = r(table)
        matrix list n
        tempname p
        scalar `p' = n[4,1]
        quietly: putexcel set "SipilaUKB_$S_DATE.xlsm", sheet("FIGURE sensitivity dist") modify
                quietly: putexcel O`prow'=(`p'), nformat(number_d4)
        restore
        }


*FIGURE sensitivity, CNS vs other infections
count
tab anyanycns
tab anynoncns
replace anyanycns=0 if ensianycnspvm>=exitpvm
replace anynoncns=0 if ensinoncnspvm>=exitpvm
replace ensianycnspvm=. if ensianycnspvm>=exitpvm
replace ensinoncnspvm=. if ensinoncnspvm>=exitpvm

replace anyanycns = . if anyanycns==0 & anyanyinf==1
tab anyanycns

replace anynoncns = . if anynoncns==0 & anyanyinf==1
tab anynoncns

tab anyanycns
tab anynoncns
tab anyanycns ensidementia
tab anynoncns ensidementia



local dg anycns noncns
local n_dg: word count `dg'
local dg_name `""Any CNS infection" "Any extra-CNS infection""'
quietly: local irow = 28
    forvalues i=1/`n_dg' {
        preserve
        quietly: local idg : word `i' of `dg'
        quietly: local idg_name : word  `i' of `dg_name'
        display ""
        display "`idg' followed by broad dementia, adjusted for sex and education"
```

```
					display ""
					display "UKB replication analysis"
					display ""
					display "$S_TIME  $S_DATE"
					display ""

					stset exitpvm, id(id) failure(ensidementia) origin(time syntpvm) enter(time lag_entry_`idg') 
scale(365.25)
					tab any`idg'
					tab any`idg' _d

					stdescribe
					quietly: local failures = `r(N_fail)'
					stcox any`idg' i.supu i.ses
					quietly: matrix m = r(table)
					matrix list m
					tempname hr ll ul
					scalar `hr' = m[1,1]
					scalar `ll' = m[5,1]
					scalar `ul' = m[6,1]
					quietly: putexcel set "SipilaUKB_$S_DATE.xlsm", sheet("FIGURE sensitivity dist") modify
							quietly: putexcel K`irow'=(`e(N_sub)'), nformat(number)
							quietly: putexcel L`irow'=(`e(N_fail)'), nformat(number)
							quietly: putexcel E`irow'=(`hr'), nformat(number_d2)
							quietly: putexcel F`irow'=(`ll'), nformat(number_d2)
							quietly: putexcel G`irow'=(`ul'), nformat(number_d2)
							quietly: putexcel B`irow'=("`idg'")
							quietly: putexcel C`irow'=("UKB")
							quietly: putexcel D`irow'=("`idg_name'")
					tab any`idg', matcell(x), if _st==1
					matrix list x
					tab any`idg' ensidementia, matcell(y), if _d==1
					matrix list y
					quietly: putexcel set "SipilaUKB_$S_DATE.xlsm", sheet("FIGURE sensitivity dist") modify
							quietly: putexcel M`irow'=(x[2,1]), nformat(number)
							quietly: putexcel N`irow'=(y[2,1]), nformat(number)
					local ++irow
					local ++irow
					restore
					}

*Difference between CNS infections and extra-CNS infections

			*CNS infection vs non cns infection
			capture drop anycnsnotcns
			capture drop lag_entry_cnsnotcns
			capture gen byte anycnsnotcns = 1 if ensianycnspvm<=ensinoncnspvm & anyanycns==1 & 
ensianycnspvm<exitpvm
			replace anycnsnotcns = 0 if ensinoncnspvm<ensianycnspvm & anycnsnotcns!=1 & anynoncns==1 & 
ensinoncnspvm<exitpvm
			capture egen ensicnsnotcnspvm = rowmin(ensianycnspvm ensinoncnspvm)
			replace ensicnsnotcnspvm = . if ensicnsnotcnspvm >= exitpvm
			replace ensicnsnotcnspvm = . if anycnsnotcns!=1 & anycnsnotcns!=0
			gen lag_entry_cnsnotcns = lag_entry_anyinf if anyanyinf==0
			replace lag_entry_cnsnotcns = ensicnsnotcnspvm if ensicnsnotcnspvm!=. & (anycnsnotcns==1 | 
anycnsnotcns==0)
			count
			sum ensicnsnotcnspvm
```

```
tab anycnsnotcns
tab anyanycns
tab anynoncns
tab anycnsnotcns ensidementia
tab anyanycns ensidementia
tab anynoncns ensidementia

local dg cnsnotcns
local n_dg: word count `dg'
local dg_name `""CNS vs extra-CNS infection""'
quietly: local irow = 34
forvalues i=1/`n_dg' {
preserve
quietly: local idg : word `i' of `dg'
quietly: local idg_name : word  `i' of `dg_name'
display ""
display "`idg' followed by broad dementia, adjusted for sex and education"
display ""
display "Pooled IPD-analysis"
display ""
display "$S_TIME  $S_DATE"
display ""

stset exitpvm, id(id) failure(ensidementia) origin(time syntpvm) enter(time lag_entry_`idg')
scale(365.25)
keep if _st==1
gen byte anyeither = 1 if ensi`idg'pvm < exitpvm
replace anyeither = 0 if anyanyinf==0
tab any`idg'
tab any`idg' _d
tab anyeither
tab anyeither _d

stdescribe

gen `idg'_compare = 0 if anyanyinf==0
replace `idg'_compare = 1 if any`idg'==1
replace `idg'_compare = 2 if any`idg'==0
tab `idg'_compare
tab `idg'_compare _d
stcox i.`idg'_compare supu ses
test 1.`idg'_compare = 2.`idg'_compare

quietly: putexcel set "SipilaUKB_$S_DATE.xlsm", sheet("FIGURE sensitivity dist") modify
quietly: putexcel K`irow'=(`e(N_sub)'), nformat(number)
quietly: putexcel L`irow'=(`e(N_fail)'), nformat(number)
quietly: putexcel E`irow'=(`r(p)'), nformat(number_d2)
quietly: putexcel B`irow'=("`idg'")
quietly: putexcel C`irow'=("UKB")
quietly: putexcel D`irow'=("`idg_name'")
local ++irow

local ++irow
restore
}
```

**** Analysis ALZHEIMER'S DISEASE *****

***** SINGLE VS MULTIPLE INFECTIONS *******

```
local dg_name `""Multiple infections""'
local irow 16
local prow = `irow'
				preserve

				egen lag_entry_multiinf = rowmax(lag_entry_anyinf ensitoinenanyinfpvm ensikolmasanyinfpvm)
				egen multiinfpvm = rowmax(ensianyinfpvm ensitoinenanyinfpvm ensikolmasanyinfpvm)

				stset exitpvm, id(id) failure(dementiatype = 1) origin(time syntpvm) enter(time
lag_entry_multiinf) scale(365.25)

				gen byte multiinf = 0
				tab multiinf
				replace multiinf = 1 if anyanyinf==1
				tab multiinf
				replace multiinf = 2 if ensitoinenanyinfpvm!=.
				replace multiinf = 3 if ensikolmasanyinfpvm!=.
				tab multiinf

				stdescribe
				tempname hr1 ll1 ul1 hr2 ll2 ul2 hr3 ll3 ul3 N1 N2 N3 fail1 fail2 fail3
				forvalues i =1/3 {
				stcox i.multiinf 2.supu#cohort 2.ses#cohort 3.ses#cohort, strata(cohort), if inlist(multiinf,0,`i')
				quietly: matrix m = r(table)
				matrix list m
				scalar `N`i'' = e(N_sub)
				scalar `fail`i'' = e(N_fail)
				scalar `hr`i'' = m[1,2]
				scalar `ll`i'' = m[5,2]
				scalar `ul`i'' = m[6,2]
				}

				forvalues i =1/3 {
				quietly: putexcel set "SipilaUKB_$S_DATE.xlsm", sheet("FIGURE sens dist AD") modify
						quietly: putexcel K`irow'=(`N`i''), nformat(number)
						quietly: putexcel L`irow'=(`fail`i''), nformat(number)
						quietly: putexcel E`irow'=(`hr`i''), nformat(number_d2)
						quietly: putexcel F`irow'=(`ll`i''), nformat(number_d2)
						quietly: putexcel G`irow'=(`ul`i''), nformat(number_d2)
						quietly: putexcel B`irow'=("Multiinf")
						quietly: putexcel C`irow'=("UKB")
						quietly: putexcel D`irow'=("Single vs multiple infections, infection No. `i'")
				tab multiinf, matcell(x), if inlist(multiinf,0,`i') & _st==1
				matrix list x
				tab multiinf ensidementia, matcell(y), if inlist(multiinf,0,`i') & _d==1
				matrix list y
				quietly: putexcel set "SipilaUKB_$S_DATE.xlsm", sheet("FIGURE sens dist AD") modify
						quietly: putexcel M`irow'=(x[2,1]), nformat(number)
						quietly: putexcel N`irow'=(y[2,1]), nformat(number)

				local ++irow
```

```
local ++irow
}

*P for trend
stcox multiinf i.supu i.ses
quietly: matrix n = r(table)
matrix list n
tempname p
scalar `p' = n[4,1]
quietly: putexcel set "SipilaUKB_$S_DATE.xlsm", sheet("FIGURE sens dist AD") modify
	quietly: putexcel O`prow'=(`p'), nformat(number_d4)


restore
```

```
***** One or several different pathogens *******

local dg_name `""Multiple organisms""'
local idg_name `""Multiple_organisms""'
local irow 37
local prow = `irow'

preserve
drop if ses==.
egen ensibactviralpvm = rowmin(ensibactinfpvm ensiviralinfpvm)
gen toinenbactviralpvm = ensibactviralpvm if ensibactinfpvm==ensiviralinfpvm &
ensibactinfpvm!=.
replace toinenbactviralpvm = ensibactinfpvm if ensibactinfpvm>ensiviralinfpvm &
ensibactinfpvm!=.
replace toinenbactviralpvm = ensiviralinfpvm if ensiviralinfpvm>ensibactinfpvm &
ensiviralinfpvm!=.
gen anybactviral = 1 if ensibactviralpvm!=.
gen toinenbactviral = 1 if toinenbactviralpvm!=.
egen lag_entry_bactviral = rowmax(lag_entry_anyinf ensibactviralpvm toinenbactviralpvm)

stset exitpvm, id(id) failure(dementiatype = 1) origin(time syntpvm) enter(time
lag_entry_bactviral) scale(365.25)
quietly: keep id anybactviral supu cohort exitpvm ensidementia syntpvm entrypvm ensi*pvm
*toinen* _st _d _origin _t _t0 ses anyanyinf
sum ensibactviralpvm toinenbactviralpvm
tab anybactviral if _st==1
tab toinenbactviral if _st==1

gen byte multiinf = 0 if anyanyinf==0
tab multiinf
tab multiinf if _st==1
replace multiinf = 1 if anybactviral==1
tab multiinf
tab multiinf if _st==1
replace multiinf = 2 if toinenbactviral==1
tab multiinf
tab multiinf if _st==1

quietly: stdescribe
stcox i.multiinf 2.supu#cohort 2.ses#cohort 3.ses#cohort, strata(cohort), if multiinf!=2
quietly: matrix m = r(table)
matrix list m
```

```
tempname hr1 ll1 ul1 hr2 ll2 ul2 N1 fail1 N2 fail2
scalar `hr1' = m[1,2]
scalar `ll1' = m[5,2]
scalar `ul1' = m[6,2]
scalar `N1' = e(N_sub)
scalar `fail1' = e(N_fail)
stcox i.multiinf 2.supu#cohort 2.ses#cohort 3.ses#cohort, strata(cohort), if multiinf!=1
quietly: matrix m = r(table)
matrix list m
scalar `hr2' = m[1,2]
scalar `ll2' = m[5,2]
scalar `ul2' = m[6,2]
scalar `N2' = e(N_sub)
scalar `fail2' = e(N_fail)

forvalues i =1/2 {
quietly: putexcel set "SipilaUKB_$S_DATE.xlsm", sheet("FIGURE sens dist AD") modify
        quietly: putexcel K`irow'=(`N`i''), nformat(number)
        quietly: putexcel L`irow'=(`fail`i''), nformat(number)
        quietly: putexcel E`irow'=(`hr`i''), nformat(number_d2)
        quietly: putexcel F`irow'=(`ll`i''), nformat(number_d2)
        quietly: putexcel G`irow'=(`ul`i''), nformat(number_d2)
        quietly: putexcel B`irow'=("`idg_name'")
        quietly: putexcel C`irow'="UKB"
        quietly: putexcel D`irow'=("Different micro-organisms, infection No. `i'")
tab multiinf, matcell(x), if _st==1 & inlist(multiinf,0,`i')
matrix list x
tab multiinf ensidementia, matcell(y), if _d==1 & inlist(multiinf,0,`i')
matrix list y
quietly: putexcel set "SipilaUKB_$S_DATE.xlsm", sheet("FIGURE sens dist AD") modify
        quietly: putexcel M`irow'=(x[2,1]), nformat(number)
        quietly: putexcel N`irow'=(y[2,1]), nformat(number)
local ++irow
local ++irow
}
*P for trend
stcox multiinf 2.supu#cohort 2.ses#cohort 3.ses#cohort, strata(cohort)
quietly: matrix n = r(table)
matrix list n
tempname p
scalar `p' = n[4,1]
quietly: putexcel set "SipilaUKB_$S_DATE.xlsm", sheet("FIGURE sens dist AD") modify
        quietly: putexcel O`prow'=(`p'), nformat(number_d4)

restore
```

```
***** NUMBER OF SIMULTANEOUS INFECTIONS *******

local dg infcount
local n_dg: word count `dg'
local dg_name `""No. of simultaneous infections""'

quietly: local irow = 23
local prow = `irow'
    forvalues i=1/`n_dg' {
        preserve
        quietly: local idg : word `i' of `dg'
        quietly: local idg_name : word  `i' of `dg_name'
```

```
display ""
display "`idg' followed by broad dementia, adjusted for sex and education"
display ""
display "Pooled IPD-analysis, normal model"
display ""
display "$S_TIME  $S_DATE"
display ""

stset exitpvm, id(id) failure(dementiatype = 1) origin(time syntpvm) enter(time
lag_entry_anyinf) scale(365.25)

tab any`idg'
tab any`idg' _d

quietly: stdescribe
quietly: local failures = `r(N_fail)'
stcox i.any`idg' 2.supu#cohort 2.ses#cohort 3.ses#cohort, strata(cohort), if any`idg'!=2
quietly: matrix m = r(table)
matrix list m
tempname hr1 ll1 ul1 hr2 ll2 ul2 N1 fail1 N2 fail2
scalar `hr1' = m[1,2]
scalar `ll1' = m[5,2]
scalar `ul1' = m[6,2]
scalar `N1' = e(N_sub)
scalar `fail1' = e(N_fail)
stcox i.any`idg' 2.supu#cohort 2.ses#cohort 3.ses#cohort, strata(cohort), if any`idg'!=1
quietly: matrix m = r(table)
matrix list m
scalar `hr2' = m[1,2]
scalar `ll2' = m[5,2]
scalar `ul2' = m[6,2]
scalar `N2' = e(N_sub)
scalar `fail2' = e(N_fail)

forvalues i =1/2 {
quietly: putexcel set "SipilaUKB_$S_DATE.xlsm", sheet("FIGURE sens dist AD") modify
        quietly: putexcel K`irow'=(`N`i''), nformat(number)
        quietly: putexcel L`irow'=(`fail`i''), nformat(number)
        quietly: putexcel E`irow'=(`hr`i''), nformat(number_d2)
        quietly: putexcel F`irow'=(`ll`i''), nformat(number_d2)
        quietly: putexcel G`irow'=(`ul`i''), nformat(number_d2)
        quietly: putexcel B`irow'=("Simultaneous infection diagnoses")
        quietly: putexcel C`irow'=("UKB")
        quietly: putexcel D`irow'=("No. of simultaneous infection diagnoses = `i'")
tab any`idg', matcell(x), if _st==1
matrix list x
tab any`idg' ensidementia, matcell(y), if _d==1
matrix list y
quietly: putexcel set "SipilaUKB_$S_DATE.xlsm", sheet("FIGURE sens dist AD") modify
        local j = `i' + 1
        quietly: putexcel M`irow'=(x[`j',1]), nformat(number)
        quietly: putexcel N`irow'=(y[`j',1]), nformat(number)
local ++irow
local ++irow
}

*P for trend
stcox any`idg' i.supu i.ses
```

```
                quietly: matrix n = r(table)
                matrix list n
                tempname p
                scalar `p' = n[4,1]
                quietly: putexcel set "SipilaUKB_$S_DATE.xlsm", sheet("FIGURE sens dist AD") modify
                        quietly: putexcel O`prow'=(`p'), nformat(number_d4)
                restore
                }


*FIGURE sensitivity, CNS vs other infections

tab anyanycns
tab anynoncns
tab anyanycns dementiatype
tab anynoncns dementiatype


local dg anycns noncns
local n_dg: word count `dg'
local dg_name `""Any CNS infection" "Any extra-CNS infection""'
quietly: local irow = 28
        forvalues i=1/`n_dg' {
                preserve
                quietly: local idg : word `i' of `dg'
                quietly: local idg_name : word  `i' of `dg_name'
                display ""
                display "`idg' followed by broad dementia, adjusted for sex and education"
                display ""
                display "UKB replication analysis, normal model and 5-year exclusion"
                display ""
                display "$S_TIME  $S_DATE"
                display ""

                stset exitpvm, id(id) failure(dementiatype = 1) origin(time syntpvm) enter(time lag_entry_`idg')
scale(365.25)
                tab any`idg'
                tab any`idg' _d

                stdescribe
                quietly: local failures = `r(N_fail)'
                stcox any`idg' i.supu i.ses
                quietly: matrix m = r(table)
                matrix list m
                tempname hr ll ul
                scalar `hr' = m[1,1]
                scalar `ll' = m[5,1]
                scalar `ul' = m[6,1]
                quietly: putexcel set "SipilaUKB_$S_DATE.xlsm", sheet("FIGURE sens dist AD") modify
                        quietly: putexcel K`irow'=(`e(N_sub)'), nformat(number)
                        quietly: putexcel L`irow'=(`e(N_fail)'), nformat(number)
                        quietly: putexcel E`irow'=(`hr'), nformat(number_d2)
                        quietly: putexcel F`irow'=(`ll'), nformat(number_d2)
                        quietly: putexcel G`irow'=(`ul'), nformat(number_d2)
                        quietly: putexcel B`irow'=("`idg'")
                        quietly: putexcel C`irow'=("UKB")
                        quietly: putexcel D`irow'=("`idg_name'")
                tab any`idg', matcell(x), if _st==1
```

```
			matrix list x
			tab any`idg' ensidementia, matcell(y), if _d==1
			matrix list y
			quietly: putexcel set "SipilaUKB_$S_DATE.xlsm", sheet("FIGURE sens dist AD") modify
					quietly: putexcel M`irow'=(x[2,1]), nformat(number)
					quietly: putexcel N`irow'=(y[2,1]), nformat(number)
			local ++irow
			local ++irow
			restore
			}

*Difference between CNS infections and extra-CNS infections

		*CNS infection vs non cns infection
		tab anycnsnotcns
		tab anyanycns
		tab anynoncns
		tab anycnsnotcns dementiatype
		tab anyanycns dementiatype
		tab anynoncns dementiatype


local dg cnsnotcns
local n_dg: word count `dg'
local dg_name `""CNS vs extra-CNS infection""'
quietly: local irow = 34
		forvalues i=1/`n_dg' {
			preserve
			quietly: local idg : word `i' of `dg'
			quietly: local idg_name : word  `i' of `dg_name'
			display ""
			display "`idg' followed by broad dementia, adjusted for sex and education"
			display ""
			display "Pooled IPD-analysis"
			display ""
			display "$S_TIME  $S_DATE"
			display ""

			stset exitpvm, id(id) failure(dementiatype = 1) origin(time syntpvm) enter(time
lag_entry_anyinf) scale(365.25)

			gen byte anyeither = 1 if ensi`idg'pvm < exitpvm
			replace anyeither = 0 if anyanyinf==0
			tab any`idg'
			tab any`idg' _d
			tab anyeither
			tab anyeither _d

			stdescribe

			gen `idg'_compare = 0 if anyanyinf==0
			replace `idg'_compare = 1 if any`idg'==1
			replace `idg'_compare = 2 if any`idg'==0
			tab `idg'_compare
			tab `idg'_compare _d
			stcox i.`idg'_compare supu ses
			test 1.`idg'_compare = 2.`idg'_compare
```

```
quietly: putexcel set "SipilaUKB_$S_DATE.xlsm", sheet("FIGURE sens dist AD") modify
        quietly: putexcel K`irow'=(`e(N_sub)'), nformat(number)
        quietly: putexcel L`irow'=(`e(N_fail)'), nformat(number)
        quietly: putexcel E`irow'=(`r(p)'), nformat(number_d2)
        quietly: putexcel B`irow'=("`idg'")
        quietly: putexcel C`irow'=("UKB")
        quietly: putexcel D`irow'=("`idg_name'")
local ++irow

local ++irow
restore
}
```

```
**** Analysis VASCULAR DEMENTIA *****

***** SINGLE VS MULTIPLE INFECTIONS *******

local dg_name `""Multiple infections""'
local irow 16
local prow = `irow'
        preserve

        egen lag_entry_multiinf = rowmax(lag_entry_anyinf ensitoinenanyinfpvm ensikolmasanyinfpvm)
        egen multiinfpvm = rowmax(ensianyinfpvm ensitoinenanyinfpvm ensikolmasanyinfpvm)

        stset exitpvm, id(id) failure(dementiatype = 4) origin(time syntpvm) enter(time
lag_entry_multiinf) scale(365.25)

        gen byte multiinf = 0
        tab multiinf
        replace multiinf = 1 if anyanyinf==1
        tab multiinf
        replace multiinf = 2 if ensitoinenanyinfpvm!=.
        replace multiinf = 3 if ensikolmasanyinfpvm!=.
        tab multiinf

        stdescribe
        tempname hr1 ll1 ul1 hr2 ll2 ul2 hr3 ll3 ul3 N1 N2 N3 fail1 fail2 fail3
        forvalues i =1/3 {
        stcox i.multiinf 2.supu#cohort 2.ses#cohort 3.ses#cohort, strata(cohort), if inlist(multiinf,0,`i')
        quietly: matrix m = r(table)
        matrix list m
        scalar `N`i'' = e(N_sub)
        scalar `fail`i'' = e(N_fail)
        scalar `hr`i'' = m[1,2]
        scalar `ll`i'' = m[5,2]
        scalar `ul`i'' = m[6,2]
        }

        forvalues i =1/3 {
        quietly: putexcel set "SipilaUKB_$S_DATE.xlsm", sheet("FIGURE sens dist VaD") modify
                quietly: putexcel K`irow'=(`N`i''), nformat(number)
                quietly: putexcel L`irow'=(`fail`i''), nformat(number)
                quietly: putexcel E`irow'=(`hr`i''), nformat(number_d2)
```

```
quietly: putexcel F`irow'=(`ll`i''), nformat(number_d2)
quietly: putexcel G`irow'=(`ul`i''), nformat(number_d2)
quietly: putexcel B`irow'=("Multiinf")
quietly: putexcel C`irow'=("UKB")
quietly: putexcel D`irow'=("Single vs multiple infections, infection No. `i'")
tab multiinf, matcell(x), if inlist(multiinf,0,`i') & _st==1
matrix list x
tab multiinf ensidementia, matcell(y), if inlist(multiinf,0,`i') & _d==1
matrix list y
quietly: putexcel set "SipilaUKB_$S_DATE.xlsm", sheet("FIGURE sens dist VaD") modify
quietly: putexcel M`irow'=(x[2,1]), nformat(number)
quietly: putexcel N`irow'=(y[2,1]), nformat(number)

local ++irow
local ++irow
}

*P for trend
stcox multiinf i.supu i.ses
quietly: matrix n = r(table)
matrix list n
tempname p
scalar `p' = n[4,1]
quietly: putexcel set "SipilaUKB_$S_DATE.xlsm", sheet("FIGURE sens dist VaD") modify
quietly: putexcel O`prow'=(`p'), nformat(number_d4)


restore


***** One or several different pathogens *******

local dg_name `""Multiple organisms""'
local idg_name `""Multiple_organisms""'
local irow 37
local prow = `irow'

preserve
drop if ses==.
egen ensibactviralpvm = rowmin(ensibactinfpvm ensiviralinfpvm)
gen toinenbactviralpvm = ensibactviralpvm if ensibactinfpvm==ensiviralinfpvm & ensibactinfpvm!=.
replace toinenbactviralpvm = ensibactinfpvm if ensibactinfpvm>ensiviralinfpvm & ensibactinfpvm!=.
replace toinenbactviralpvm = ensiviralinfpvm if ensiviralinfpvm>ensibactinfpvm & ensiviralinfpvm!=.
gen anybactviral = 1 if ensibactviralpvm!=.
gen toinenbactviral = 1 if toinenbactviralpvm!=.
egen lag_entry_bactviral = rowmax(lag_entry_anyinf ensibactviralpvm toinenbactviralpvm)

stset exitpvm, id(id) failure(dementiatype = 4) origin(time syntpvm) enter(time lag_entry_bactviral) scale(365.25)
quietly: keep id anybactviral supu cohort exitpvm ensidementia syntpvm entrypvm ensi*pvm *toinen* _st _d _origin _t _t0 ses anyanyinf
sum ensibactviralpvm toinenbactviralpvm
tab anybactviral if _st==1
tab toinenbactviral if _st==1
```

```
gen byte multiinf = 0 if anyanyinf==0
tab multiinf
tab multiinf if _st==1
replace multiinf = 1 if anybactviral==1
tab multiinf
tab multiinf if _st==1
replace multiinf = 2 if toinenbactviral==1
tab multiinf
tab multiinf if _st==1

quietly: stdescribe
stcox i.multiinf 2.supu#cohort 2.ses#cohort 3.ses#cohort, strata(cohort), if multiinf!=2
quietly: matrix m = r(table)
matrix list m
tempname hr1 ll1 ul1 hr2 ll2 ul2 N1 fail1 N2 fail2
scalar `hr1' = m[1,2]
scalar `ll1' = m[5,2]
scalar `ul1' = m[6,2]
scalar `N1' = e(N_sub)
scalar `fail1' = e(N_fail)
stcox i.multiinf 2.supu#cohort 2.ses#cohort 3.ses#cohort, strata(cohort), if multiinf!=1
quietly: matrix m = r(table)
matrix list m
scalar `hr2' = m[1,2]
scalar `ll2' = m[5,2]
scalar `ul2' = m[6,2]
scalar `N2' = e(N_sub)
scalar `fail2' = e(N_fail)

forvalues i =1/2 {
quietly: putexcel set "SipilaUKB_$S_DATE.xlsm", sheet("FIGURE sens dist VaD") modify
		quietly: putexcel K`irow'=(`N`i''), nformat(number)
		quietly: putexcel L`irow'=(`fail`i''), nformat(number)
		quietly: putexcel E`irow'=(`hr`i''), nformat(number_d2)
		quietly: putexcel F`irow'=(`ll`i''), nformat(number_d2)
		quietly: putexcel G`irow'=(`ul`i''), nformat(number_d2)
		quietly: putexcel B`irow'=("`idg_name'")
		quietly: putexcel C`irow'="UKB"
		quietly: putexcel D`irow'=("Different micro-organisms, infection No. `i'")
tab multiinf, matcell(x), if _st==1 & inlist(multiinf,0,`i')
matrix list x
tab multiinf ensidementia, matcell(y), if _d==1 & inlist(multiinf,0,`i')
matrix list y
quietly: putexcel set "SipilaUKB_$S_DATE.xlsm", sheet("FIGURE sens dist VaD") modify
		quietly: putexcel M`irow'=(x[2,1]), nformat(number)
		quietly: putexcel N`irow'=(y[2,1]), nformat(number)
local ++irow
local ++irow
}
*P for trend
stcox multiinf 2.supu#cohort 2.ses#cohort 3.ses#cohort, strata(cohort)
quietly: matrix n = r(table)
matrix list n
tempname p
scalar `p' = n[4,1]
quietly: putexcel set "SipilaUKB_$S_DATE.xlsm", sheet("FIGURE sens dist VaD") modify
		quietly: putexcel O`prow'=(`p'), nformat(number_d4)
```

```
				restore

***** NUMBER OF SIMULTANEOUS INFECTIONS *******

count
tab anyinfcount, mis
tab anyinfcount dementiatype, mis
tab anyinfcount, mis
tab anyinfcount ensidementia, mis

local dg infcount
local n_dg: word count `dg'
local dg_name `""No. of simultaneous infections""'

quietly: local irow = 23
local prow = `irow'
		forvalues i=1/`n_dg' {
				preserve
				quietly: local idg : word `i' of `dg'
				quietly: local idg_name : word  `i' of `dg_name'
				display ""
				display "`idg' followed by broad dementia, adjusted for sex and education"
				display ""
				display "Pooled IPD-analysis, normal model"
				display ""
				display "$S_TIME  $S_DATE"
				display ""

				stset exitpvm, id(id) failure(dementiatype = 4) origin(time syntpvm) enter(time
lag_entry_anyinf) scale(365.25)

				tab any`idg'
				tab any`idg' _d

				quietly: stdescribe
				quietly: local failures = `r(N_fail)'
				stcox i.any`idg' 2.supu#cohort 2.ses#cohort 3.ses#cohort, strata(cohort), if any`idg'!=2
				quietly: matrix m = r(table)
				matrix list m
				tempname hr1 ll1 ul1 hr2 ll2 ul2 N1 fail1 N2 fail2
				scalar `hr1' = m[1,2]
				scalar `ll1' = m[5,2]
				scalar `ul1' = m[6,2]
				scalar `N1' = e(N_sub)
				scalar `fail1' = e(N_fail)
				stcox i.any`idg' 2.supu#cohort 2.ses#cohort 3.ses#cohort, strata(cohort), if any`idg'!=1
				quietly: matrix m = r(table)
				matrix list m
				scalar `hr2' = m[1,2]
				scalar `ll2' = m[5,2]
				scalar `ul2' = m[6,2]
				scalar `N2' = e(N_sub)
				scalar `fail2' = e(N_fail)

				forvalues i =1/2 {
				quietly: putexcel set "SipilaUKB_$S_DATE.xlsm", sheet("FIGURE sens dist VaD") modify
					quietly: putexcel K`irow'=(`N`i''), nformat(number)
					quietly: putexcel L`irow'=(`fail`i''), nformat(number)
```

```
					quietly: putexcel E`irow'=(`hr`i''), nformat(number_d2)
					quietly: putexcel F`irow'=(`ll`i''), nformat(number_d2)
					quietly: putexcel G`irow'=(`ul`i''), nformat(number_d2)
					quietly: putexcel B`irow'=("Simultaneous infection diagnoses")
					quietly: putexcel C`irow'=("UKB")
					quietly: putexcel D`irow'=("No. of simultaneous infection diagnoses = `i'")
			tab any`idg', matcell(x), if _st==1
			matrix list x
			tab any`idg' ensidementia, matcell(y), if _d==1
			matrix list y
			quietly: putexcel set "SipilaUKB_$S_DATE.xlsm", sheet("FIGURE sens dist VaD") modify
					local j = `i' + 1
					quietly: putexcel M`irow'=(x[`j',1]), nformat(number)
					quietly: putexcel N`irow'=(y[`j',1]), nformat(number)
			local ++irow
			local ++irow
			}

			*P for trend
			stcox any`idg' i.supu i.ses
			quietly: matrix n = r(table)
			matrix list n
			tempname p
			scalar `p' = n[4,1]
			quietly: putexcel set "SipilaUKB_$S_DATE.xlsm", sheet("FIGURE sens dist VaD") modify
					quietly: putexcel O`prow'=(`p'), nformat(number_d4)
			restore
			}


*FIGURE sensitivity, CNS vs other infections
tab anyanycns
tab anynoncns
tab anyanycns dementiatype
tab anynoncns dementiatype


local dg anycns noncns
local n_dg: word count `dg'
local dg_name `""Any CNS infection" "Any extra-CNS infection""'
quietly: local irow = 28
		forvalues i=1/`n_dg' {
			preserve
			quietly: local idg : word `i' of `dg'
			quietly: local idg_name : word  `i' of `dg_name'
			display ""
			display "`idg' followed by broad dementia, adjusted for sex and education"
			display ""
			display "UKB replication analysis, normal model and 5-year exclusion"
			display ""
			display "$S_TIME  $S_DATE"
			display ""

			stset exitpvm, id(id) failure(dementiatype = 4) origin(time syntpvm) enter(time lag_entry_`idg')
scale(365.25)
			tab any`idg'
			tab any`idg' _d
```

```
stdescribe
quietly: local failures = `r(N_fail)'
stcox any`idg' i.supu i.ses
quietly: matrix m = r(table)
matrix list m
tempname hr ll ul
scalar `hr' = m[1,1]
scalar `ll' = m[5,1]
scalar `ul' = m[6,1]
quietly: putexcel set "SipilaUKB_$S_DATE.xlsm", sheet("FIGURE sens dist VaD") modify
        quietly: putexcel K`irow'=(`e(N_sub)'), nformat(number)
        quietly: putexcel L`irow'=(`e(N_fail)'), nformat(number)
        quietly: putexcel E`irow'=(`hr'), nformat(number_d2)
        quietly: putexcel F`irow'=(`ll'), nformat(number_d2)
        quietly: putexcel G`irow'=(`ul'), nformat(number_d2)
        quietly: putexcel B`irow'=("`idg'")
        quietly: putexcel C`irow'=("UKB")
        quietly: putexcel D`irow'=("`idg_name'")
tab any`idg', matcell(x), if _st==1
matrix list x
tab any`idg' ensidementia, matcell(y), if _d==1
matrix list y
quietly: putexcel set "SipilaUKB_$S_DATE.xlsm", sheet("FIGURE sens dist VaD") modify
        quietly: putexcel M`irow'=(x[2,1]), nformat(number)
        quietly: putexcel N`irow'=(y[2,1]), nformat(number)
local ++irow
local ++irow
restore
}
```

```
*Difference between CNS infections and extra-CNS infections

        *CNS infection vs non cns infection
        tab anycnsnotcns
        tab anyanycns
        tab anynoncns
        tab anycnsnotcns dementiatype
        tab anyanycns dementiatype
        tab anynoncns dementiatype


local dg cnsnotcns
local n_dg: word count `dg'
local dg_name `""CNS vs extra-CNS infection""'
quietly: local irow = 34
        forvalues i=1/`n_dg' {
                preserve
                quietly: local idg : word `i' of `dg'
                quietly: local idg_name : word  `i' of `dg_name'
                display ""
                display "`idg' followed by broad dementia, adjusted for sex and education"
                display ""
                display "Pooled IPD-analysis"
                display ""
                display "$S_TIME  $S_DATE"
                display ""
```

```
				stset exitpvm, id(id) failure(dementiatype = 4) origin(time syntpvm) enter(time 
lag_entry_anyinf) scale(365.25)

				gen byte anyeither = 1 if ensi`idg'pvm < exitpvm
				replace anyeither = 0 if anyanyinf==0
				tab any`idg'
				tab any`idg' _d
				tab anyeither
				tab anyeither _d

				stdescribe

				gen `idg'_compare = 0 if anyanyinf==0
				replace `idg'_compare = 1 if any`idg'==1
				replace `idg'_compare = 2 if any`idg'==0
				tab `idg'_compare
				tab `idg'_compare _d
				stcox i.`idg'_compare supu ses
				test 1.`idg'_compare = 2.`idg'_compare

				quietly: putexcel set "SipilaUKB_$S_DATE.xlsm", sheet("FIGURE sens dist VaD") modify
						quietly: putexcel K`irow'=(`e(N_sub)'), nformat(number)
						quietly: putexcel L`irow'=(`e(N_fail)'), nformat(number)
						quietly: putexcel E`irow'=(`r(p)'), nformat(number_d2)
						quietly: putexcel B`irow'=("`idg'")
						quietly: putexcel C`irow'=("UKB")
						quietly: putexcel D`irow'=("`idg_name'")
				local ++irow

				local ++irow
				restore
				}
log close

*** eTABLE 4 (former TABLE 2 ***
capture log close
log using "TABLE2.log", replace

capture gen hypertensio_combpvm = hypert_combpvm

local dg anyinf
local eks `""no one" "hypertensio" "diabetes" "ihd" "cereb" "parkinson"'
local n_dg: word count `dg'
local n_eks: word count `eks'
local dg_name `""Gram negative bacteria" "Herpesviruses""'
local eks_name `""no one" "hypertension" "diabetes" "ischaemic heart disease" "cerebrovascular disease" "Parkinson´s disease"'
quietly: local irow = 8
		forvalues i=1/`n_dg' {
		forvalues j=1/`n_eks' {
				preserve
				quietly: local idg : word `i' of `dg'
				quietly: local idg_name : word  `i' of `dg_name'
				quietly: local ieks : word `j' of `eks'
				quietly: local ieks_name : word  `j' of `eks_name'
				display ""
				display "`idg' followed by broad dementia, adjusted for sex and education and stratified by 
cohort"
				display ""
```

```
display "Pooled IPD-analysis, normal model"
display ""
display "$S_TIME  $S_DATE"
display ""

if inlist("`ieks'","hypertensio", "diabetes", "ihd", "cereb", "parkinson") {
drop if `ieks'_combpvm <= lag_entry_anyinf
}

stset exitpvm, id(id) failure(ensidementia) origin(time syntpvm) enter(time lag_entry_anyinf) 
scale(365.25)
keep if _st==1

if "`ieks'"=="hypertensio" & "`idg'"=="anyinf" {
local irow = 25
}

quietly: capture keep id any`idg' supu cohort exitpvm ensidementia syntpvm entrypvm 
ensi`idg'pvm  _st _d _origin _t _t0 ses `ieks'_comb
tab any`idg'

quietly: stdescribe
quietly: local failures = `r(N_fail)'
stcox any`idg' 2.supu#cohort 2.ses#cohort 3.ses#cohort, strata(cohort)
quietly: matrix m = r(table)
tempname hr ll ul
scalar `hr' = m[1,1]
scalar `ll' = m[5,1]
scalar `ul' = m[6,1]
quietly: putexcel set "SipilaUKB_$S_DATE.xlsm", sheet("TABLE2") modify
		quietly: putexcel K`irow'=(`e(N_sub)'), nformat(number)
		quietly: putexcel L`irow'=(`e(N_fail)'), nformat(number)
		quietly: putexcel E`irow'=(`hr'), nformat(number_d2)
		quietly: putexcel F`irow'=(`ll'), nformat(number_d2)
		quietly: putexcel G`irow'=(`ul'), nformat(number_d2)
		quietly: putexcel B`irow'=("`idg'")
		quietly: putexcel C`irow'="UKB"
		quietly: putexcel D`irow'=("Those with `ieks_name' excluded")
tab any`idg', matcell(x), if _st==1
matrix list x
quietly: tab any`idg' _d, matcell(y), if _d==1
matrix list y
quietly: putexcel set "SipilaUKB_$S_DATE.xlsm", sheet("TABLE2") modify
		quietly: putexcel M`irow'=(x[2,1]), nformat(number)
		quietly: putexcel N`irow'=(y[2,1]), nformat(number)
local ++irow
restore
}
}

*Early- vs late-onset dementia
local idg anyinf
quietly: stset exitpvm, id(id) failure(ensidementia) origin(time syntpvm) enter(time 
lag_entry_anyinf) scale(365.25)
keep if _st==1
local idg anyinf
quietly: capture keep id any`idg' supu cohort exitpvm ensidementia syntpvm entrypvm 
ensi`idg'pvm  _st _d _origin _t _t0 ses
```

```
tab any`idg'

stsplit age65, at(64.999)
recode age65 64.999=1

preserve
keep if age65==0
local irow 12
quietly: stdescribe
quietly: local failures = `r(N_fail)'
stcox any`idg' 2.supu#cohort 2.ses#cohort 3.ses#cohort, strata(cohort)
quietly: matrix m = r(table)
tempname hr ll ul
scalar `hr' = m[1,1]
scalar `ll' = m[5,1]
scalar `ul' = m[6,1]
quietly: putexcel set "SipilaUKB_$S_DATE.xlsm", sheet("TABLE2") modify
        quietly: putexcel K`irow'=(`e(N_sub)'), nformat(number)
        quietly: putexcel L`irow'=(`e(N_fail)'), nformat(number)
        quietly: putexcel E`irow'=(`hr'), nformat(number_d2)
        quietly: putexcel F`irow'=(`ll'), nformat(number_d2)
        quietly: putexcel G`irow'=(`ul'), nformat(number_d2)
        quietly: putexcel B`irow'=("`idg'")
        quietly: putexcel C`irow'="UKB"
        quietly: putexcel D`irow'=("Early-onset dementia (onset before age 65)")
tab any`idg', matcell(x), if _st==1
matrix list x
quietly: tab any`idg' ensidementia, matcell(y), if _d==1
matrix list y
quietly: putexcel set "SipilaUKB_$S_DATE.xlsm", sheet("TABLE2") modify
        quietly: putexcel M`irow'=(x[2,1]), nformat(number)
        quietly: putexcel N`irow'=(y[2,1]), nformat(number)
restore

preserve
keep if age65==1
local irow 13
quietly: stdescribe
quietly: local failures = `r(N_fail)'
stcox any`idg' 2.supu#cohort 2.ses#cohort 3.ses#cohort, strata(cohort)
quietly: matrix m = r(table)
tempname hr ll ul
scalar `hr' = m[1,1]
scalar `ll' = m[5,1]
scalar `ul' = m[6,1]
quietly: putexcel set "SipilaUKB_$S_DATE.xlsm", sheet("TABLE2") modify
        quietly: putexcel K`irow'=(`e(N_sub)'), nformat(number)
        quietly: putexcel L`irow'=(`e(N_fail)'), nformat(number)
        quietly: putexcel E`irow'=(`hr'), nformat(number_d2)
        quietly: putexcel F`irow'=(`ll'), nformat(number_d2)
        quietly: putexcel G`irow'=(`ul'), nformat(number_d2)
        quietly: putexcel B`irow'=("`idg'")
        quietly: putexcel C`irow'="UKB"
        quietly: putexcel D`irow'=("Late-onset dementia (onset at or after age 65)")
tab any`idg', matcell(x), if _st==1
matrix list x
quietly: tab any`idg' ensidementia, matcell(y), if _d==1
matrix list y
```

```
				quietly: putexcel set "SipilaUKB_$S_DATE.xlsm", sheet("TABLE2") modify
						quietly: putexcel M`irow'=(x[2,1]), nformat(number)
						quietly: putexcel N`irow'=(y[2,1]), nformat(number)
				restore


*Type of dementia
		use "UKB_lag.dta", clear
		gen cohort=1

		tab dementiatyyppi_laaja anyanyinf if ensidementia==1, col chi
		local idg anyinf
		local irow 15
		local j 1
		foreach type in AD FTD PD VD other unspecified  {
				preserve

				display ""
				display "`type'"
				display ""
				local idg gramminus
				drop if ses==.

				stset exitpvm, id(id) failure(dementiatype==`j') origin(time syntpvm) enter(time
lag_entry_anyinf) scale(365.25)
				keep if _st==1

				local idg anyinf
				quietly: capture keep id any`idg' supu cohort exitpvm ensidementia syntpvm entrypvm
ensi`idg'pvm  _st _d _origin _t _t0 ses
				tab any`idg'

				quietly: stdescribe
				quietly: local failures = `r(N_fail)'
				stcox any`idg' 2.supu#cohort 2.ses#cohort 3.ses#cohort, strata(cohort)
				quietly: matrix m = r(table)
				tempname hr ll ul
				scalar `hr' = m[1,1]
				scalar `ll' = m[5,1]
				scalar `ul' = m[6,1]
				quietly: putexcel set "SipilaUKB_$S_DATE.xlsm", sheet("TABLE2") modify
						quietly: putexcel K`irow'=(`e(N_sub)'), nformat(number)
						quietly: putexcel L`irow'=(`e(N_fail)'), nformat(number)
						quietly: putexcel E`irow'=(`hr'), nformat(number_d2)
						quietly: putexcel F`irow'=(`ll'), nformat(number_d2)
						quietly: putexcel G`irow'=(`ul'), nformat(number_d2)
						quietly: putexcel B`irow'=("`idg'")
						quietly: putexcel C`irow'="UKB"
						quietly: putexcel D`irow'=("`type'")
				tab any`idg', matcell(x), if _st==1
				matrix list x
				quietly: tab any`idg' _d, matcell(y), if _d==1
				matrix list y
				quietly: putexcel set "SipilaUKB_$S_DATE.xlsm", sheet("TABLE2") modify
						quietly: putexcel M`irow'=(x[2,1]), nformat(number)
						quietly: putexcel N`irow'=(y[2,1]), nformat(number)
				local ++irow
				local ++j
```

```
				restore
				}

*Adjustments

*Potential confounders
local dg anyinf
local eks alcocl smoking bmi_who all
local n_dg: word count `dg'
local n_eks: word count `eks'
local dg_name `""Any infectious disease""'
local eks_name `""heavy drinking" "smoking" "body mass index" "all three""'
quietly: local irow = 31
		forvalues i=1/`n_dg' {
		forvalues j=1/`n_eks' {
				quietly: local idg : word `i' of `dg'
				quietly: local idg_name : word  `i' of `dg_name'
				quietly: local ieks : word `j' of `eks'
				quietly: local ieks_name : word  `j' of `eks_name'
				display ""
				display "$S_TIME  $S_DATE"
				display ""

				preserve
				drop if ses==.

				if "`ieks'" == "all" {
				drop if alcocl==. | smoking==. | bmi_who==.
				count
				}
				else {
				tab `ieks'
				drop if `ieks' ==.
				}
				stset exitdate, id(id) failure(dementia) origin(time birthday) enter(time lag_entry_`idg')
scale(365.25)
				keep if _st==1 & ses!=.
				tab any`idg'
				tab any`idg' _d

				*Those with data on the covariate available but without adjustment
				stdescribe
				stcox any`idg' i.sex i.ses
				quietly: matrix m = r(table)
				tempname hr ll ul
				scalar `hr' = m[1,1]
				scalar `ll' = m[5,1]
				scalar `ul' = m[6,1]
				quietly: putexcel set "SipilaUKB_$S_DATE.xlsm", sheet("TABLE2") modify
						quietly: putexcel K`irow'=(`e(N_sub)'), nformat(number)
						quietly: putexcel L`irow'=(`e(N_fail)'), nformat(number)
						quietly: putexcel E`irow'=(`hr'), nformat(number_d2)
						quietly: putexcel F`irow'=(`ll'), nformat(number_d2)
						quietly: putexcel G`irow'=(`ul'), nformat(number_d2)
						quietly: putexcel B`irow'=("`idg'")
						quietly: putexcel D`irow'=("Data available for `ieks_name'")
				tab any`idg', matcell(x), if _st==1
				matrix list x
```

```
					tab any`idg' ensidementia, matcell(y), if _d==1
					matrix list y
					quietly: putexcel set "SipilaUKB_$S_DATE.xlsm", sheet("TABLE2") modify
							quietly: putexcel M`irow'=(x[2,1]), nformat(number)
							quietly: putexcel N`irow'=(y[2,1]), nformat(number)
					local ++irow

					*Those with data on the covariate available and adjusted
					stdescribe
					quietly: local failures = `r(N_fail)'
					if "`ieks'" == "all" {
					stcox any`idg' sex i.ses i.alcocl i.smoking i.bmi_who
					}
					else  {
					stcox any`idg' sex i.ses i.`ieks'
					}
					quietly: matrix m = r(table)
					tempname hr ll ul
					scalar `hr' = m[1,1]
					scalar `ll' = m[5,1]
					scalar `ul' = m[6,1]
					quietly: putexcel set "SipilaUKB_$S_DATE.xlsm", sheet("TABLE2") modify
							quietly: putexcel K`irow'=(`e(N_sub)'), nformat(number)
							quietly: putexcel L`irow'=(`e(N_fail)'), nformat(number)
							quietly: putexcel E`irow'=(`hr'), nformat(number_d2)
							quietly: putexcel F`irow'=(`ll'), nformat(number_d2)
							quietly: putexcel G`irow'=(`ul'), nformat(number_d2)
							quietly: putexcel B`irow'=("`idg'")
							quietly: putexcel D`irow'=("Additionally adjusted for `ieks_name'")
					tab any`idg', matcell(x), if _st==1
					matrix list x
					tab any`idg' ensidementia, matcell(y), if _d==1
					matrix list y
					quietly: putexcel set "SipilaUKB_$S_DATE.xlsm", sheet("TABLE2") modify
							quietly: putexcel M`irow'=(x[2,1]), nformat(number)
							quietly: putexcel N`irow'=(y[2,1]), nformat(number)
					local ++irow
					local ++irow
					restore
}
}

*Potential period effect
gen syntvuosi=year(birthday)

local dg anyinf
local n_dg: word count `dg'
local dg_name `""Any infectious disease""'
local irow 22
		forvalues i=1/`n_dg' {
					quietly: local idg : word `i' of `dg'
					quietly: local idg_name : word  `i' of `dg_name'
					display ""
					display "`idg' followed by broad dementia, adjusted for sex and education and stratified by
cohort"
					display ""
					display "Pooled IPD-analysis, potential period effect"
					display ""
```

```
display "$S_TIME  $S_DATE"
display ""

drop if ses==.

quietly: stset exitpvm, id(id) failure(ensidementia) origin(time syntpvm) enter(time 
lag_entry_anyinf) scale(365.25)
keep if _st==1
quietly: keep id any`idg' supu cohort exitpvm ensidementia /*failure*/ syntpvm entrypvm 
ensi`idg'pvm  _st _d _origin _t _t0 ses syntvuosi
tab any`idg'

quietly: stdescribe
quietly: local failures = `r(N_fail)'
stcox any`idg' 2.supu#cohort 2.ses#cohort 3.ses#cohort c.syntvuosi#cohort, strata(cohort)

quietly: matrix m = r(table)
tempname hr ll ul
scalar `hr' = m[1,1]
scalar `ll' = m[5,1]
scalar `ul' = m[6,1]
quietly: putexcel set "SipilaUKB_$S_DATE.xlsm", sheet("TABLE2") modify
        quietly: putexcel K`irow'=(`e(N_sub)'), nformat(number)
        quietly: putexcel L`irow'=(`e(N_fail)'), nformat(number)
        quietly: putexcel E`irow'=(`hr'), nformat(number_d2)
        quietly: putexcel F`irow'=(`ll'), nformat(number_d2)
        quietly: putexcel G`irow'=(`ul'), nformat(number_d2)
        quietly: putexcel B`irow'=("`idg'")
        quietly: putexcel C`irow'="UKB"
        quietly: putexcel D`irow'=("Adjusted for period effect")
tab any`idg', matcell(x), if _st==1
matrix list x
quietly: tab any`idg' ensidementia, matcell(y), if _d==1
matrix list y
quietly: putexcel set "SipilaUKB_$S_DATE.xlsm", sheet("TABLE2") modify
        quietly: putexcel M`irow'=(x[2,1]), nformat(number)
        quietly: putexcel N`irow'=(y[2,1]), nformat(number)
local irow 65
}
log close




******** TIME-DEPENDENT ANALYSIS **************

capture log close
log using "Sipila, infections and dementia, UKB replication $S_DATE, time-dependent.log", replace

***** eFIGURE 12 (TIME-DEPENDENT VERSION OF FIGURE 3) ********

*load data

replace toinenanyinf=0 if toinenanyinf==.
replace kolmasanyinf=0 if kolmasanyinf==.

gen cohort=1
```

```
local dg anyinf bactinf viralinf
local n_dg: word count `dg'
local dg_name `""Any infectious disease" "Bacterial infection" "Viral infection""'
quietly: local irow = 7

		forvalues i=1/`n_dg' {
				quietly: local idg : word `i' of `dg'
				quietly: local idg_name : word  `i' of `dg_name'
				display ""
				display "`idg' , adjusted for sex and education and other variables"
				display ""
				display "UKB replication analysis"
				display ""
				display "$S_TIME  $S_DATE"
				display ""

				/* Models
				MODEL 1: adjusted for age (as the time scale), sex and ses
				MODEL 2 = model 1 + those with HIV excluded
				MODEL 3 = model 2 + those with missing data on alcohol, smoking, and bmi excluded
				MODEL 4 = model 3 + adjusted for alcohol, smoking, bmim hypertension, diabetes
				MODEL 5 = model 4 + those with data on apoe4 missing dropped
				MODEL 6 = model 5 + adjusted for apoe4 genotype
				*/

		forvalues j=1/6 {

				preserve

				stset exitpvm, id(id) failure(ensidementia) origin(time syntpvm) enter(time entrypvm)
scale(365.25)
				tab any`idg'
				tab any`idg' _d
				sum diabetes_combpvm
				replace diabetes_combpvm = . if diabetes_combpvm>=exitpvm
				sum diabetes_combpvm
				gen dm = 1 if diabetes_combpvm < exitpvm
				tab dm
				sum hypert_combpvm
				replace hypert_combpvm = . if hypert_combpvm>=exitpvm
				sum hypert_combpvm
				gen htn = 1 if hypert_combpvm < exitpvm
				tab htn

				stsplit `idg'_split, at(0) after(time=ensi`idg'pvm)
				tab `idg'_split
				gen `idg'_timedep =0
				replace `idg'_timedep = 1 if any`idg'==1 & `idg'_split==0
				tab `idg'_timedep
				tab any`idg' if `idg'_split!=-1

				stsplit dm_split, at(0) after(time=diabetes_combpvm)
				gen dm_timedep =0
				replace dm_timedep = 1 if dm==1 & dm_split==0
				tab dm_timedep
				tab dm_timedep if dm_split!=-1 & `idg'_split!=-1

				stsplit htn_split, at(0) after(time=hypert_combpvm)
```

```
gen htn_timedep =0
replace htn_timedep = 1 if htn==1 & htn_split==0
tab htn_timedep
tab htn_timedep if htn_split!=-1 & `idg'_split!=-1 & dm_split!=-1

if `j'==1 {
di ""
di "MODEL 1: adjusted for age (as the time scale), sex and ses"
di ""
stdescribe
quietly: local failures = `r(N_fail)'
stcox `idg'_timedep i.supu i.ses
}
else if `j'==2 {
di ""
di "MODEL 2 = model 1 + those with HIV excluded"
di ""
drop if anyhiv==1
stdescribe
quietly: local failures = `r(N_fail)'
stcox `idg'_timedep i.supu i.ses
}
else if `j'==3 {
di ""
di "MODEL 3 = model 2 + those with missing data on alcohol, smoking, and bmi excluded"
di ""
drop if anyhiv==1
drop if tupakka==.
drop if alcocl==.
drop if bmi_who==.
stdescribe
quietly: local failures = `r(N_fail)'
stcox `idg'_timedep i.supu i.ses
}
else if `j'==4 {
di ""
di "MODEL 4 = model 3 + adjusted for alcohol, smoking, bmim hypertension, diabetes"
di ""
drop if anyhiv==1
drop if tupakka==.
drop if alcocl==.
drop if bmi_who==.
stdescribe
quietly: local failures = `r(N_fail)'
stcox `idg'_timedep i.supu i.ses i.tupakka i.alcocl i.bmi_who i.htn_timedep i.dm_timedep
}
else if `j'==5 {
di ""
di "MODEL 5 = model 4 + those with data on apoe4 missing dropped"
di ""
drop if anyhiv==1
drop if tupakka==.
drop if alcocl==.
drop if bmi_who==.
drop if apoe==.
stdescribe
quietly: local failures = `r(N_fail)'
stcox `idg'_timedep i.supu i.ses i.tupakka i.alcocl i.bmi_who i.htn_timedep i.dm_timedep
```

```
}
else if `j'==6 {
di ""
di "MODEL 6 = model 5 + adjusted for apoe4 genotype"
di ""
drop if anyhiv==1
drop if tupakka==.
drop if alcocl==.
drop if bmi_who==.
drop if apoe==.
stdescribe
quietly: local failures = `r(N_fail)'
stcox `idg'_timedep i.supu i.ses i.tupakka i.alcocl i.bmi_who i.htn_timedep i.dm_timedep i.apoe
}
quietly: matrix m = r(table)
tempname hr ll ul
scalar `hr' = m[1,1]
scalar `ll' = m[5,1]
scalar `ul' = m[6,1]
quietly: putexcel set "SipilaUKB_time-dep_$S_DATE.xlsm", sheet("FIGURE UKB") modify
        quietly: putexcel K`irow'=(`e(N_sub)'), nformat(number)
        quietly: putexcel L`irow'=(`e(N_fail)'), nformat(number)
        quietly: putexcel E`irow'=(`hr'), nformat(number_d2)
        quietly: putexcel F`irow'=(`ll'), nformat(number_d2)
        quietly: putexcel G`irow'=(`ul'), nformat(number_d2)
        quietly: putexcel B`irow'=("`idg'")
        quietly: putexcel C`irow'=("UKB")
        quietly: putexcel D`irow'=("`idg_name' model `j'")
tab any`idg', matcell(x), if `idg'_split!=-1 & htn_split!=-1 & dm_split!=-1
matrix list x
quietly: tab any`idg' ensidementia, matcell(y), if `idg'_split!=-1 & _d==1 & htn_split!=-1 &
dm_split!=-1
matrix list y
quietly: putexcel set "SipilaUKB_time-dep_$S_DATE.xlsm", sheet("FIGURE UKB") modify
        quietly: putexcel M`irow'=(x[2,1]), nformat(number)
        quietly: putexcel N`irow'=(y[2,1]), nformat(number)
local ++irow
restore
}
local ++irow
}


**** By type of dementia *****

*load data

replace toinenanyinf=0 if toinenanyinf==.
replace kolmasanyinf=0 if kolmasanyinf==.

gen cohort=1

local dg anyinf bactinf viralinf
local n_dg: word count `dg'
local dg_name `""Any infectious disease" "Bacterial infection" "Viral infection""'

*Dementiatyypit 1 = AD, 2 = V
foreach outcome in AD VD FTD PD {
```

```
if "`outcome'" == "AD" {
local type=1
}
else if "`outcome'" == "VD" {
local type=4
}
else if "`outcome'" == "FTD" {
local type=2
}
else if "`outcome'" == "PD" {
local type=3
}

quietly: local irow = 7

forvalues i=1/`n_dg' {
        quietly: local idg : word `i' of `dg'
        quietly: local idg_name : word  `i' of `dg_name'
        display ""
        display "`idg' , adjusted for sex and education and other variables"
        display ""
        display "UKB replication analysis"
        display ""
        display "$S_TIME  $S_DATE"
        display ""

        /* Models
        MODEL 1: adjusted for age (as the time scale), sex and ses
        MODEL 2 = model 1 + those with HIV excluded
        MODEL 3 = model 2 + those with missing data on alcohol, smoking, and bmi excluded
        MODEL 4 = model 3 + adjusted for alcohol, smoking, bmim hypertension, diabetes
        MODEL 5 = model 4 + those with data on apoe4 missing dropped
        MODEL 6 = model 5 + adjusted for apoe4 genotype
        */

forvalues j=1/6 {

        preserve

        stset exitpvm, id(id) failure(dementiatype==`type') origin(time syntpvm) enter(time entrypvm)
scale(365.25)
        tab any`idg'
        tab any`idg' _d
        sum diabetes_combpvm
        replace diabetes_combpvm = . if diabetes_combpvm>=exitpvm
        sum diabetes_combpvm
        gen dm = 1 if diabetes_combpvm < exitpvm
        tab dm
        sum hypert_combpvm
        replace hypert_combpvm = . if hypert_combpvm>=exitpvm
        sum hypert_combpvm
        gen htn = 1 if hypert_combpvm < exitpvm
        tab htn

        stsplit `idg'_split, at(0) after(time=ensi`idg'pvm)
        tab `idg'_split
        gen `idg'_timedep =0
        replace `idg'_timedep = 1 if any`idg'==1 & `idg'_split==0
```

```
tab `idg'_timedep
tab any`idg' if `idg'_split!=-1

stsplit dm_split, at(0) after(time=diabetes_combpvm)
gen dm_timedep =0
replace dm_timedep = 1 if dm==1 & dm_split==0
tab dm_timedep
tab dm_timedep if dm_split!=-1 & `idg'_split!=-1

stsplit htn_split, at(0) after(time=hypert_combpvm)
gen htn_timedep =0
replace htn_timedep = 1 if htn==1 & htn_split==0
tab htn_timedep
tab htn_timedep if htn_split!=-1 & `idg'_split!=-1 & dm_split!=-1

if `j'==1 {
di ""
di "MODEL 1: adjusted for age (as the time scale), sex and ses"
di ""
stdescribe
quietly: local failures = `r(N_fail)'
stcox `idg'_timedep i.supu i.ses
}
else if `j'==2 {
di ""
di "MODEL 2 = model 1 + those with HIV excluded"
di ""
drop if anyhiv==1
stdescribe
quietly: local failures = `r(N_fail)'
stcox `idg'_timedep i.supu i.ses
}
else if `j'==3 {
di ""
di "MODEL 3 = model 2 + those with missing data on alcohol, smoking, and bmi excluded"
di ""
drop if anyhiv==1
drop if tupakka==.
drop if alcocl==.
drop if bmi_who==.
stdescribe
quietly: local failures = `r(N_fail)'
stcox `idg'_timedep i.supu i.ses
}
else if `j'==4 {
di ""
di "MODEL 4 = model 3 + adjusted for alcohol, smoking, bmim hypertension, diabetes"
di ""
drop if anyhiv==1
drop if tupakka==.
drop if alcocl==.
drop if bmi_who==.
stdescribe
quietly: local failures = `r(N_fail)'
stcox `idg'_timedep i.supu i.ses i.tupakka i.alcocl i.bmi_who i.htn_timedep i.dm_timedep
}
else if `j'==5 {
di ""
```

```
di "MODEL 5 = model 4 + those with data on apoe4 missing dropped"
di ""
drop if anyhiv==1
drop if tupakka==.
drop if alcocl==.
drop if bmi_who==.
drop if apoe==.
stdescribe
quietly: local failures = `r(N_fail)'
stcox `idg'_timedep i.supu i.ses i.tupakka i.alcocl i.bmi_who i.htn_timedep i.dm_timedep
}
else if `j'==6 {
di ""
di "MODEL 6 = model 5 + adjusted for apoe4 genotype"
di ""
drop if anyhiv==1
drop if tupakka==.
drop if alcocl==.
drop if bmi_who==.
drop if apoe==.
stdescribe
quietly: local failures = `r(N_fail)'
stcox `idg'_timedep i.supu i.ses i.tupakka i.alcocl i.bmi_who i.htn_timedep i.dm_timedep i.apoe
}
quietly: matrix m = r(table)
tempname hr ll ul
scalar `hr' = m[1,1]
scalar `ll' = m[5,1]
scalar `ul' = m[6,1]
quietly: putexcel set "SipilaUKB_time-dep_$S_DATE.xlsm", sheet("FIGURE UKB, `outcome'")
modify
        quietly: putexcel K`irow'=(`e(N_sub)'), nformat(number)
        quietly: putexcel L`irow'=(`e(N_fail)'), nformat(number)
        quietly: putexcel E`irow'=(`hr'), nformat(number_d2)
        quietly: putexcel F`irow'=(`ll'), nformat(number_d2)
        quietly: putexcel G`irow'=(`ul'), nformat(number_d2)
        quietly: putexcel B`irow'=("`idg'")
        quietly: putexcel C`irow'=("UKB")
        quietly: putexcel D`irow'=("`idg_name' model `j'")
tab any`idg', matcell(x), if `idg'_split!=-1 & htn_split!=-1 & dm_split!=-1
matrix list x
quietly: tab any`idg' ensidementia, matcell(y), if `idg'_split!=-1 & _d==1 & htn_split!=-1 &
dm_split!=-1
matrix list y
quietly: putexcel set "SipilaUKB_time-dep_$S_DATE.xlsm", sheet("FIGURE UKB, `outcome'")
modify
        quietly: putexcel M`irow'=(x[2,1]), nformat(number)
        quietly: putexcel N`irow'=(y[2,1]), nformat(number)
local ++irow
restore
}
local ++irow
}
}

log close
```

```
capture log close
log using "Sipila, UKB infection burden $S_DATE, time-dependent.log", replace

replace toinenanyinf=0 if toinenanyinf==.
replace kolmasanyinf=0 if kolmasanyinf==.

capture gen cohort=1

***** TIME-DEPENDENT: SINGLE VS MULTIPLE INFECTIONS *******

count
tab toinenanyinf, mis
tab kolmasanyinf, mis

tab anyanyinf if ensianyinfpvm<exitpvm & ensianyinfpvm < ensitoinenanyinfpvm
tab toinenanyinf if ensitoinenanyinfpvm<exitpvm & ensitoinenanyinfpvm < ensikolmasanyinfpvm
tab kolmasanyinf if ensikolmasanyinfpvm<exitpvm

tab anyanyinf if ensianyinfpvm<exitpvm & ensianyinfpvm < ensitoinenanyinfpvm & ensitoinenanyinfpvm>=exitpvm
tab toinenanyinf if ensitoinenanyinfpvm<exitpvm & ensitoinenanyinfpvm < ensikolmasanyinfpvm  &
ensikolmasanyinfpvm>=exitpvm
tab kolmasanyinf if ensikolmasanyinfpvm<exitpvm

tab ensidementia if ensianyinfpvm<exitpvm & ensitoinenanyinfpvm>=exitpvm
tab ensidementia if ensitoinenanyinfpvm<exitpvm & ensikolmasanyinfpvm>=exitpvm
tab ensidementia if ensikolmasanyinfpvm<exitpvm

sum ensianyinfpvm
replace ensianyinfpvm = . if ensianyinfpvm>=exitpvm
sum ensianyinfpvm

sum ensitoinenanyinfpvm
replace ensitoinenanyinfpvm = . if ensitoinenanyinfpvm>=exitpvm
sum ensitoinenanyinfpvm

sum ensikolmasanyinfpvm
replace ensikolmasanyinfpvm = . if ensikolmasanyinfpvm>=exitpvm
sum ensikolmasanyinfpvm

replace toinenanyinf = . if ensitoinenanyinfpvm==.
replace kolmasanyinf = . if ensikolmasanyinfpvm==.

local dg_name `""Multiple infections""'
local irow 16
local prow = `irow'
                    preserve
                    stset exitpvm, id(id) failure(ensidementia) origin(time syntpvm) enter(time entrypvm)
scale(365.25)

                    stsplit first_split, at(0) after(time=ensianyinfpvm)
                    gen multiinf_timedep =0
                    tab multiinf_timedep
                    replace multiinf_timedep = 1 if anyanyinf==1 & first_split==0
                    tab multiinf_timedep
                    stsplit second_split, at(0) after(time=ensitoinenanyinfpvm)
                    replace multiinf_timedep = 2 if toinenanyinf==1 & second_split==0
                    tab multiinf_timedep
                    stsplit third_split, at(0) after(time=ensikolmasanyinfpvm)
```

```
replace multiinf_timedep = 3 if kolmasanyinf==1 & third_split==0
tab multiinf_timedep

stdescribe
quietly: local failures = `r(N_fail)'
stcox i.multiinf_timedep i.supu i.ses
quietly: matrix m = r(table)
matrix list m
tempname hr1 ll1 ul1 hr2 ll2 ul2 hr3 ll3 ul3
scalar `hr1' = m[1,2]
scalar `ll1' = m[5,2]
scalar `ul1' = m[6,2]
scalar `hr2' = m[1,3]
scalar `ll2' = m[5,3]
scalar `ul2' = m[6,3]
scalar `hr3' = m[1,4]
scalar `ll3' = m[5,4]
scalar `ul3' = m[6,4]

forvalues i =1/3 {
quietly: putexcel set "UKB infection burden time-dep $S_DATE.xlsm", sheet("FIGURE sensitivity")
modify
        quietly: putexcel K`irow'=(`e(N_sub)'), nformat(number)
        quietly: putexcel L`irow'=(`e(N_fail)'), nformat(number)
        quietly: putexcel E`irow'=(`hr`i''), nformat(number_d2)
        quietly: putexcel F`irow'=(`ll`i''), nformat(number_d2)
        quietly: putexcel G`irow'=(`ul`i''), nformat(number_d2)
        quietly: putexcel B`irow'=("Multiinf")
        quietly: putexcel C`irow'=("UKB")
        quietly: putexcel D`irow'=("Single vs multiple infections, infection No. `i'")
tab multiinf_timedep, matcell(x), if first_split!=-1 & second_split!=-1 & third_split!=-1
matrix list x
tab multiinf_timedep ensidementia, matcell(y), if first_split!=-1 & second_split!=-1 &
third_split!=-1 &_d==1
matrix list y
quietly: putexcel set "UKB infection burden time-dep $S_DATE.xlsm", sheet("FIGURE sensitivity")
modify
        local j = `i' + 1
        quietly: putexcel M`irow'=(x[`j',1]), nformat(number)
        quietly: putexcel N`irow'=(y[`j',1]), nformat(number)

local ++irow
local ++irow
}

*P for trend
stcox multiinf_timedep i.supu i.ses
quietly: matrix n = r(table)
matrix list n
tempname p
scalar `p' = n[4,1]
quietly: putexcel set "UKB infection burden time-dep $S_DATE.xlsm", sheet("FIGURE sensitivity")
modify
        quietly: putexcel O`prow'=(`p'), nformat(number)


restore
```

*****TIME-DEPENDENT: One or several different pathogens *******

```
local dg_name `""Multiple organisms""'
local idg_name `""Multiple_organisms""'
local irow 37
local prow = `irow'

				preserve
				drop if ses==.
				egen ensibactviralpvm = rowmin(ensibactinfpvm ensiviralinfpvm)
				gen toinenbactviralpvm = ensibactviralpvm if ensibactinfpvm==ensiviralinfpvm & 
ensibactinfpvm!=.
				replace toinenbactviralpvm = ensibactinfpvm if ensibactinfpvm>ensiviralinfpvm & 
ensibactinfpvm!=.
				replace toinenbactviralpvm = ensiviralinfpvm if ensiviralinfpvm>ensibactinfpvm & 
ensiviralinfpvm!=.
				gen anybactviral = 1 if ensibactviralpvm!=.
				gen toinenbactviral = 1 if toinenbactviralpvm!=.

				quietly: stset exitpvm, id(id) failure(ensidementia) origin(time syntpvm) enter(time entrypvm) 
scale(365.25)
				quietly: keep id anybactviral supu cohort exitpvm ensidementia syntpvm entrypvm ensi*pvm 
*toinen* _st _d _origin _t _t0 ses
				sum ensibactviralpvm toinenbactviralpvm
				tab anybactviral
				tab anybactviral if toinenbactviral!=1
				tab toinenbactviral
				tab anybactviral ensidementia
				tab anybactviral ensidementia if toinenbactviral!=1
				tab toinenbactviral ensidementia

				stsplit first_split, at(0) after(time=ensibactviralpvm)
				gen multiinf_timedep =0
				tab multiinf_timedep
				replace multiinf_timedep = 1 if anybactviral==1 & first_split==0
				tab multiinf_timedep
				stsplit second_split, at(0) after(time=toinenbactviralpvm)
				replace multiinf_timedep = 2 if toinenbactviral==1 & second_split==0
				tab multiinf_timedep

				quietly: stdescribe
				quietly: local failures = `r(N_fail)'
				stcox i.multiinf_timedep 2.supu#cohort 2.ses#cohort 3.ses#cohort, strata(cohort)
				quietly: matrix m = r(table)
				matrix list m
				tempname hr1 ll1 ul1 hr2 ll2 ul2
				scalar `hr1' = m[1,2]
				scalar `ll1' = m[5,2]
				scalar `ul1' = m[6,2]
				scalar `hr2' = m[1,3]
				scalar `ll2' = m[5,3]
				scalar `ul2' = m[6,3]

				forvalues i =1/2 {
				quietly: putexcel set "UKB infection burden time-dep $S_DATE.xlsm", sheet("FIGURE sensitivity") 
modify
						quietly: putexcel K`irow'=(`e(N_sub)'), nformat(number)
```

```
					quietly: putexcel L`irow'=(`e(N_fail)'), nformat(number)
					quietly: putexcel E`irow'=(`hr`i''), nformat(number_d2)
					quietly: putexcel F`irow'=(`ll`i''), nformat(number_d2)
					quietly: putexcel G`irow'=(`ul`i''), nformat(number_d2)
					quietly: putexcel B`irow'=("`idg_name'")
					quietly: putexcel C`irow'=("UKB")
					quietly: putexcel D`irow'=("Different micro-organisms, infection No. `i'")
				tab multiinf_timedep, matcell(x), if first_split!=-1 & second_split!=-1
				matrix list x
				tab multiinf_timedep ensidementia, matcell(y), if first_split!=-1 & second_split!=-1 &_d==1
				matrix list y
				quietly: putexcel set "UKB infection burden time-dep $S_DATE.xlsm", sheet("FIGURE sensitivity")
modify
					local j = `i' + 1
					quietly: putexcel M`irow'=(x[`j',1]), nformat(number)
					quietly: putexcel N`irow'=(y[`j',1]), nformat(number)
				local ++irow
				local ++irow
				}
				*P for trend
				stcox multiinf_timedep 2.supu#cohort 2.ses#cohort 3.ses#cohort, strata(cohort)
				quietly: matrix n = r(table)
				matrix list n
				tempname p
				scalar `p' = n[4,1]
				quietly: putexcel set "UKB infection burden time-dep $S_DATE.xlsm", sheet("FIGURE sensitivity")
modify
					quietly: putexcel Q`prow'=(`p'), nformat(number)


				restore

*****TIME-DEPENDENT:  NUMBER OF SIMULTANEOUS INFECTIONS *******


count
tab anyinfcount, mis
tab anyinfcount ensidementia, mis
replace anyinfcount = . if ensianyinfpvm>=exitpvm
tab anyinfcount, mis
tab anyinfcount ensidementia, mis
capture gen ensiinfcountpvm=ensianyinfpvm if anyinfcount!=0 & anyinfcount!=.

local dg infcount
local n_dg: word count `dg'
local dg_name `""No. of simultaneous infections""'

quietly: local irow = 23
local prow = `irow'
		forvalues i=1/`n_dg' {
				preserve
				quietly: local idg : word `i' of `dg'
				quietly: local idg_name : word  `i' of `dg_name'
				display ""
				display "`idg' followed by broad dementia, adjusted for sex and education"
				display ""
				display "Pooled IPD-analysis, normal model"
				display ""
```

```
display "$S_TIME  $S_DATE"
display ""

quietly: stset exitpvm, id(id) failure(ensidementia) origin(time syntpvm) enter(time entrypvm)
scale(365.25)
tab any`idg'
replace any`idg' = 2 if any`idg'>2 & any`idg'<.
tab any`idg'
stsplit `idg'_split, at(0) after(time=ensi`idg'pvm)
tab `idg'_split
gen `idg'_timedep =0
replace `idg'_timedep = any`idg' if inlist(any`idg',1,2) & `idg'_split==0
tab `idg'_timedep
tab any`idg' if `idg'_split!=-1

stdescribe
quietly: local failures = `r(N_fail)'
stcox i.`idg'_timedep i.supu i.ses
quietly: matrix m = r(table)
matrix list m
tempname hr1 ll1 ul1 hr2 ll2 ul2 hr3 ll3 ul3
scalar `hr1' = m[1,2]
scalar `ll1' = m[5,2]
scalar `ul1' = m[6,2]
scalar `hr2' = m[1,3]
scalar `ll2' = m[5,3]
scalar `ul2' = m[6,3]
scalar `hr3' = m[1,4]
scalar `ll3' = m[5,4]
scalar `ul3' = m[6,4]

forvalues i =1/2 {
quietly: putexcel set "UKB infection burden time-dep $S_DATE.xlsm", sheet("FIGURE sensitivity")
modify
        quietly: putexcel K`irow'=(`e(N_sub)'), nformat(number)
        quietly: putexcel L`irow'=(`e(N_fail)'), nformat(number)
        quietly: putexcel E`irow'=(`hr`i''), nformat(number_d2)
        quietly: putexcel F`irow'=(`ll`i''), nformat(number_d2)
        quietly: putexcel G`irow'=(`ul`i''), nformat(number_d2)
        quietly: putexcel B`irow'=("Simultaneous infection diagnoses")
        quietly: putexcel C`irow'=("UKB")
        quietly: putexcel D`irow'=("No. of simultaneous infection diagnoses = `i'")
tab `idg'_timedep, matcell(x), if `idg'_split!=-1
matrix list x
tab `idg'_timedep ensidementia, matcell(y), if `idg'_split!=-1 & _d==1
matrix list y
quietly: putexcel set "UKB infection burden time-dep $S_DATE.xlsm", sheet("FIGURE sensitivity")
modify
        local j = `i' + 1
        quietly: putexcel M`irow'=(x[`j',1]), nformat(number)
        quietly: putexcel N`irow'=(y[`j',1]), nformat(number)
local ++irow
local ++irow
}

*P for trend
stcox `idg'_timedep i.supu i.ses
quietly: matrix n = r(table)
```

```
matrix list n
tempname p
scalar `p' = n[4,1]
quietly: putexcel set "UKB infection burden time-dep $S_DATE.xlsm", sheet("FIGURE sensitivity")
modify
quietly: putexcel O`prow'=(`p'), nformat(number)

local irow 24

restore
}


*TIME-DEPENDENT: FIGURE sensitivity, CNS vs other infections
count
tab anyanycns
recode anyanycns .=0
tab anyanycns
tab anynoncns
recode anynoncns .=0
tab anynoncns
replace anyanycns=0 if ensianycnspvm>=exitpvm
replace anynoncns=0 if ensinoncnspvm>=exitpvm
replace ensianycnspvm=. if ensianycnspvm>=exitpvm
replace ensinoncnspvm=. if ensinoncnspvm>=exitpvm
tab anyanycns
tab anynoncns
tab anyanycns ensidementia
tab anynoncns ensidementia


local dg anycns noncns
local n_dg: word count `dg'
local dg_name `""Any CNS infection" "Any extra-CNS infection""'
quietly: local irow = 28
	forvalues i=1/`n_dg' {
		preserve
		quietly: local idg : word `i' of `dg'
		quietly: local idg_name : word  `i' of `dg_name'
		display ""
		display "`idg' followed by broad dementia, adjusted for sex and education"
		display ""
		display "UKB replication analysis, normal model and 5-year exclusion"
		display ""
		display "$S_TIME  $S_DATE"
		display ""

		stset exitpvm, id(id) failure(ensidementia) origin(time syntpvm) enter(time entrypvm)
scale(365.25)
		tab any`idg'
		stsplit `idg'_split, at(0) after(time=ensi`idg'pvm)
		tab `idg'_split
		gen `idg'_timedep =0
		replace `idg'_timedep = 1 if any`idg'==1 & `idg'_split==0
		tab `idg'_timedep
		tab any`idg' if `idg'_split!=-1

		stdescribe
```

```
                        quietly: local failures = `r(N_fail)'
                        stcox `idg'_timedep i.supu i.ses
                        quietly: matrix m = r(table)
                        matrix list m
                        tempname hr ll ul
                        scalar `hr' = m[1,1]
                        scalar `ll' = m[5,1]
                        scalar `ul' = m[6,1]
                        quietly: putexcel set "UKB infection burden time-dep $S_DATE.xlsm", sheet("FIGURE sensitivity")
modify
                                quietly: putexcel K`irow'=(`e(N_sub)'), nformat(number)
                                quietly: putexcel L`irow'=(`e(N_fail)'), nformat(number)
                                quietly: putexcel E`irow'=(`hr'), nformat(number_d2)
                                quietly: putexcel F`irow'=(`ll'), nformat(number_d2)
                                quietly: putexcel G`irow'=(`ul'), nformat(number_d2)
                                quietly: putexcel B`irow'=("`idg'")
                                quietly: putexcel C`irow'=("UKB")
                                quietly: putexcel D`irow'=("`idg_name'")
                        tab any`idg', matcell(x), if `idg'_split!=-1
                        matrix list x
                        tab any`idg' ensidementia, matcell(y), if `idg'_split!=-1 & _d==1
                        matrix list y
                        quietly: putexcel set "UKB infection burden time-dep $S_DATE.xlsm", sheet("FIGURE sensitivity")
modify
                                quietly: putexcel M`irow'=(x[2,1]), nformat(number)
                                quietly: putexcel N`irow'=(y[2,1]), nformat(number)
                        local ++irow
                        local ++irow
                        restore
                        }

*TIME-DEPENDENT: Difference between CNS infections and extra-CNS infections

                *CNS infection vs non cns infection
                capture gen byte anycnsnotcns = 1 if ensianycnspvm==ensianyinfpvm & anyanycns==1 &
ensianyinfpvm<exitpvm
                replace anycnsnotcns = 0 if ensinoncnspvm==ensianyinfpvm & anycnsnotcns!=1 & anynoncns==1 &
ensianyinfpvm<exitpvm
                capture gen ensicnsnotcnspvm = ensianyinfpvm if ensianyinfpvm < exitpvm
                count
                sum ensicnsnotcnspvm
                tab anycnsnotcns
                tab anyanycns
                tab anynoncns
                tab anycnsnotcns ensidementia
                tab anyanycns ensidementia
                tab anynoncns ensidementia


local dg cnsnotcns
local n_dg: word count `dg'
local dg_name `""CNS vs extra-CNS infection""'
quietly: local irow = 34
                forvalues i=1/`n_dg' {
                        preserve
                        quietly: local idg : word `i' of `dg'
                        quietly: local idg_name : word  `i' of `dg_name'
                        display ""
```

```
display "`idg' followed by broad dementia, adjusted for sex and education"
display ""
display "Pooled IPD-analysis"
display ""
display "$S_TIME  $S_DATE"
display ""

stset exitpvm, id(id) failure(ensidementia) origin(time syntpvm) enter(time entrypvm)
scale(365.25)

gen byte anyeither = ensi`idg'pvm < exitpvm
tab any`idg'
tab anyeither

stsplit `idg'_split, at(0) after(time=ensi`idg'pvm)
tab `idg'_split
gen `idg'_timedep =0
replace `idg'_timedep = 1 if anyeither==1 & `idg'_split==0
tab `idg'_timedep
tab anyeither if `idg'_split!=-1

stdescribe
quietly: local failures = `r(N_fail)'

tab anyeither if `idg'_split!=-1
tab `idg'_timedep
recode `idg'_timedep 1=2 if any`idg'==0
tab `idg'_timedep
stcox i.`idg'_timedep supu ses
test 1.`idg'_timedep = 2.`idg'_timedep

quietly: putexcel set "UKB infection burden time-dep $S_DATE.xlsm", sheet("FIGURE sensitivity")
modify
        quietly: putexcel K`irow'=(`e(N_sub)'), nformat(number)
        quietly: putexcel L`irow'=(`e(N_fail)'), nformat(number)
        quietly: putexcel E`irow'=(`r(p)'), nformat(number_d2)
        quietly: putexcel B`irow'=("`idg'")
        quietly: putexcel C`irow'=("UKB")
        quietly: putexcel D`irow'=("`idg_name'")
local ++irow

local ++irow
restore
}
```

```
****TIME-DEPENDENT:  Analysis ALZHEIMER'S DISEASE *****


*****TIME-DEPENDENT:  SINGLE VS MULTIPLE INFECTIONS *******

count
tab toinenanyinf, mis
tab kolmasanyinf, mis

tab anyanyinf if ensianyinfpvm<exitpvm & ensianyinfpvm < ensitoinenanyinfpvm
tab toinenanyinf if ensitoinenanyinfpvm<exitpvm & ensitoinenanyinfpvm < ensikolmasanyinfpvm
tab kolmasanyinf if ensikolmasanyinfpvm<exitpvm

tab anyanyinf if ensianyinfpvm<exitpvm & ensianyinfpvm < ensitoinenanyinfpvm & ensitoinenanyinfpvm>=exitpvm
tab toinenanyinf if ensitoinenanyinfpvm<exitpvm & ensitoinenanyinfpvm < ensikolmasanyinfpvm  & 
ensikolmasanyinfpvm>=exitpvm
tab kolmasanyinf if ensikolmasanyinfpvm<exitpvm

tab dementiatype if ensianyinfpvm<exitpvm & ensitoinenanyinfpvm>=exitpvm
tab dementiatype if ensitoinenanyinfpvm<exitpvm & ensikolmasanyinfpvm>=exitpvm
tab dementiatype if ensikolmasanyinfpvm<exitpvm

sum ensianyinfpvm
replace ensianyinfpvm = . if ensianyinfpvm>=exitpvm
sum ensianyinfpvm

sum ensitoinenanyinfpvm
replace ensitoinenanyinfpvm = . if ensitoinenanyinfpvm>=exitpvm
sum ensitoinenanyinfpvm

sum ensikolmasanyinfpvm
replace ensikolmasanyinfpvm = . if ensikolmasanyinfpvm>=exitpvm
sum ensikolmasanyinfpvm

replace toinenanyinf = . if ensitoinenanyinfpvm==.
replace kolmasanyinf = . if ensikolmasanyinfpvm==.

local dg_name `""Multiple infections""'
local irow 16
local prow = `irow'
                                        preserve
                                        stset exitpvm, id(id) failure(dementiatype==1) origin(time syntpvm) enter(time entrypvm) 
scale(365.25)

                                        stsplit first_split, at(0) after(time=ensianyinfpvm)
                                        gen multiinf_timedep =0
                                        tab multiinf_timedep
                                        replace multiinf_timedep = 1 if anyanyinf==1 & first_split==0
```

```
tab multiinf_timedep
stsplit second_split, at(0) after(time=ensitoinenanyinfpvm)
replace multiinf_timedep = 2 if toinenanyinf==1 & second_split==0
tab multiinf_timedep
stsplit third_split, at(0) after(time=ensikolmasanyinfpvm)
replace multiinf_timedep = 3 if kolmasanyinf==1 & third_split==0
tab multiinf_timedep

stdescribe
quietly: local failures = `r(N_fail)'
stcox i.multiinf_timedep i.supu i.ses
quietly: matrix m = r(table)
matrix list m
tempname hr1 ll1 ul1 hr2 ll2 ul2 hr3 ll3 ul3
scalar `hr1' = m[1,2]
scalar `ll1' = m[5,2]
scalar `ul1' = m[6,2]
scalar `hr2' = m[1,3]
scalar `ll2' = m[5,3]
scalar `ul2' = m[6,3]
scalar `hr3' = m[1,4]
scalar `ll3' = m[5,4]
scalar `ul3' = m[6,4]

forvalues i =1/3 {
quietly: putexcel set "UKB infection burden time-dep $S_DATE.xlsm", sheet("FIGURE sensitivity
AD") modify
        quietly: putexcel K`irow'=(`e(N_sub)'), nformat(number)
        quietly: putexcel L`irow'=(`e(N_fail)'), nformat(number)
        quietly: putexcel E`irow'=(`hr`i''), nformat(number_d2)
        quietly: putexcel F`irow'=(`ll`i''), nformat(number_d2)
        quietly: putexcel G`irow'=(`ul`i''), nformat(number_d2)
        quietly: putexcel B`irow'=("Multiinf")
        quietly: putexcel C`irow'=("UKB")
        quietly: putexcel D`irow'=("Single vs multiple infections, infection No. `i'")
tab multiinf_timedep, matcell(x), if first_split!=-1 & second_split!=-1 & third_split!=-1
matrix list x
tab multiinf_timedep dementiatype, matcell(y), if first_split!=-1 & second_split!=-1 &
third_split!=-1 &_d==1
matrix list y
quietly: putexcel set "UKB infection burden time-dep $S_DATE.xlsm", sheet("FIGURE sensitivity
AD") modify
        local j = `i' + 1
        quietly: putexcel M`irow'=(x[`j',1]), nformat(number)
        quietly: putexcel N`irow'=(y[`j',1]), nformat(number)

local ++irow
local ++irow
}

*P for trend
stcox multiinf_timedep i.supu i.ses
quietly: matrix n = r(table)
matrix list n
tempname p
scalar `p' = n[4,1]
quietly: putexcel set "UKB infection burden time-dep $S_DATE.xlsm", sheet("FIGURE sensitivity
AD") modify
```

```
quietly: putexcel O`prow'=(`p'), nformat(number)

restore
```

*****TIME-DEPENDENT: One or several different pathogens *******

```
local dg_name `""Multiple organisms""'
local idg_name `""Multiple_organisms""'
local irow 37
local prow = `irow'

preserve
drop if ses==.
egen ensibactviralpvm = rowmin(ensibactinfpvm ensiviralinfpvm)
gen toinenbactviralpvm = ensibactviralpvm if ensibactinfpvm==ensiviralinfpvm &
ensibactinfpvm!=.
replace toinenbactviralpvm = ensibactinfpvm if ensibactinfpvm>ensiviralinfpvm &
ensibactinfpvm!=.
replace toinenbactviralpvm = ensiviralinfpvm if ensiviralinfpvm>ensibactinfpvm &
ensiviralinfpvm!=.
gen anybactviral = 1 if ensibactviralpvm!=.
gen toinenbactviral = 1 if toinenbactviralpvm!=.
tab anybactviral
tab anybactviral if toinenbactviral!=1
tab toinenbactviral
tab anybactviral dementiatype
tab anybactviral dementiatype if toinenbactviral!=1
tab toinenbactviral dementiatype

quietly: stset exitpvm, id(id) failure(dementiatype==1) origin(time syntpvm) enter(time
entrypvm) scale(365.25)
quietly: keep id anybactviral supu cohort exitpvm ensidementia syntpvm entrypvm ensi*pvm
*toinen* _st _d _origin _t _t0 ses
sum ensibactviralpvm toinenbactviralpvm
tab anybactviral
tab toinenbactviral

stsplit first_split, at(0) after(time=ensibactviralpvm)
gen multiinf_timedep =0
tab multiinf_timedep
replace multiinf_timedep = 1 if anybactviral==1 & first_split==0
tab multiinf_timedep
stsplit second_split, at(0) after(time=toinenbactviralpvm)
replace multiinf_timedep = 2 if toinenbactviral==1 & second_split==0
tab multiinf_timedep

quietly: stdescribe
quietly: local failures = `r(N_fail)'
stcox i.multiinf_timedep 2.supu#cohort 2.ses#cohort 3.ses#cohort, strata(cohort)
quietly: matrix m = r(table)
matrix list m
tempname hr1 ll1 ul1 hr2 ll2 ul2
scalar `hr1' = m[1,2]
scalar `ll1' = m[5,2]
scalar `ul1' = m[6,2]
scalar `hr2' = m[1,3]
```

```
					scalar `ll2' = m[5,3]
					scalar `ul2' = m[6,3]

					forvalues i =1/2 {
					quietly: putexcel set "UKB infection burden time-dep $S_DATE.xlsm", sheet("FIGURE sensitivity
AD") modify
							quietly: putexcel K`irow'=(`e(N_sub)'), nformat(number)
							quietly: putexcel L`irow'=(`e(N_fail)'), nformat(number)
							quietly: putexcel E`irow'=(`hr`i''), nformat(number_d2)
							quietly: putexcel F`irow'=(`ll`i''), nformat(number_d2)
							quietly: putexcel G`irow'=(`ul`i''), nformat(number_d2)
							quietly: putexcel B`irow'=("`idg_name'")
							quietly: putexcel C`irow'=("UKB")
							quietly: putexcel D`irow'=("Different micro-organisms, infection No. `i'")
					tab multiinf_timedep, matcell(x), if first_split!=-1 & second_split!=-1
					matrix list x
					tab multiinf_timedep ensidementia, matcell(y), if first_split!=-1 & second_split!=-1 &_d==1
					matrix list y
					quietly: putexcel set "UKB infection burden time-dep $S_DATE.xlsm", sheet("FIGURE sensitivity
AD") modify
							local j = `i' + 1
							quietly: putexcel M`irow'=(x[`j',1]), nformat(number)
							quietly: putexcel N`irow'=(y[`j',1]), nformat(number)
					local ++irow
					local ++irow
					}
					*P for trend
					stcox multiinf_timedep 2.supu#cohort 2.ses#cohort 3.ses#cohort, strata(cohort)
					quietly: matrix n = r(table)
					matrix list n
					tempname p
					scalar `p' = n[4,1]
					quietly: putexcel set "UKB infection burden time-dep $S_DATE.xlsm", sheet("FIGURE sensitivity
AD") modify
							quietly: putexcel Q`prow'=(`p'), nformat(number)


					restore



*****TIME-DEPENDENT:  NUMBER OF SIMULTANEOUS INFECTIONS *******

count
tab anyinfcount, mis
tab anyinfcount dementiatype, mis
replace anyinfcount = . if ensianyinfpvm>=exitpvm
tab anyinfcount, mis
tab anyinfcount dementiatype, mis

local dg infcount
local n_dg: word count `dg'
local dg_name `""No. of simultaneous infections""'

quietly: local irow = 23
local prow = `irow'
		forvalues i=1/`n_dg' {
					preserve
```

```
quietly: local idg : word `i' of `dg'
quietly: local idg_name : word  `i' of `dg_name'
display ""
display "`idg' followed by broad dementia, adjusted for sex and education"
display ""
display "Pooled IPD-analysis, normal model"
display ""
display "$S_TIME  $S_DATE"
display ""

quietly: stset exitpvm, id(id) failure(dementiatype==1) origin(time syntpvm) enter(time
entrypvm) scale(365.25)
tab any`idg'
replace any`idg' = 2 if any`idg'>2 & any`idg'<.
tab any`idg'
stsplit `idg'_split, at(0) after(time=ensi`idg'pvm)
tab `idg'_split
gen `idg'_timedep =0
replace `idg'_timedep = any`idg' if inlist(any`idg',1,2) & `idg'_split==0
tab `idg'_timedep
tab any`idg' if `idg'_split!=-1

stdescribe
quietly: local failures = `r(N_fail)'
stcox i.`idg'_timedep i.supu i.ses
quietly: matrix m = r(table)
matrix list m
tempname hr1 ll1 ul1 hr2 ll2 ul2 hr3 ll3 ul3
scalar `hr1' = m[1,2]
scalar `ll1' = m[5,2]
scalar `ul1' = m[6,2]
scalar `hr2' = m[1,3]
scalar `ll2' = m[5,3]
scalar `ul2' = m[6,3]
scalar `hr3' = m[1,4]
scalar `ll3' = m[5,4]
scalar `ul3' = m[6,4]

forvalues i =1/2 {
quietly: putexcel set "UKB infection burden time-dep $S_DATE.xlsm", sheet("FIGURE sensitivity
AD") modify
        quietly: putexcel K`irow'=(`e(N_sub)'), nformat(number)
        quietly: putexcel L`irow'=(`e(N_fail)'), nformat(number)
        quietly: putexcel E`irow'=(`hr`i''), nformat(number_d2)
        quietly: putexcel F`irow'=(`ll`i''), nformat(number_d2)
        quietly: putexcel G`irow'=(`ul`i''), nformat(number_d2)
        quietly: putexcel B`irow'=("Simultaneous infection diagnoses")
        quietly: putexcel C`irow'=("UKB")
        quietly: putexcel D`irow'=("No. of simultaneous infection diagnoses = `i'")
tab `idg'_timedep, matcell(x), if `idg'_split!=-1
matrix list x
tab `idg'_timedep dementiatype, matcell(y), if `idg'_split!=-1 & _d==1
matrix list y
quietly: putexcel set "UKB infection burden time-dep $S_DATE.xlsm", sheet("FIGURE sensitivity
AD") modify
        local j = `i' + 1
        quietly: putexcel M`irow'=(x[`j',1]), nformat(number)
        quietly: putexcel N`irow'=(y[`j',1]), nformat(number)
```

```
				local ++irow
				local ++irow
				}

				*P for trend
				stcox `idg'_timedep i.supu i.ses
				quietly: matrix n = r(table)
				matrix list n
				tempname p
				scalar `p' = n[4,1]
				quietly: putexcel set "UKB infection burden time-dep $S_DATE.xlsm", sheet("FIGURE sensitivity
AD") modify
						quietly: putexcel O`prow'=(`p'), nformat(number)

				local irow 24
				restore
				}


*TIME-DEPENDENT: FIGURE sensitivity, CNS vs other infections
count
tab anyanycns
recode anyanycns .=0
tab anyanycns
tab anynoncns
recode anynoncns .=0
tab anynoncns
replace anyanycns=0 if ensianycnspvm>=exitpvm
replace anynoncns=0 if ensinoncnspvm>=exitpvm
replace ensianycnspvm=. if ensianycnspvm>=exitpvm
replace ensinoncnspvm=. if ensinoncnspvm>=exitpvm
tab anyanycns
tab anynoncns
tab anyanycns dementiatype
tab anynoncns dementiatype


local dg anycns noncns
local n_dg: word count `dg'
local dg_name `""Any CNS infection" "Any extra-CNS infection""'
quietly: local irow = 28
		forvalues i=1/`n_dg' {
				preserve
				quietly: local idg : word `i' of `dg'
				quietly: local idg_name : word  `i' of `dg_name'
				display ""
				display "`idg' followed by broad dementia, adjusted for sex and education"
				display ""
				display "UKB replication analysis, normal model and 5-year exclusion"
				display ""
				display "$S_TIME  $S_DATE"
				display ""

				stset exitpvm, id(id) failure(dementiatype==1) origin(time syntpvm) enter(time entrypvm)
scale(365.25)
				tab any`idg'
				stsplit `idg'_split, at(0) after(time=ensi`idg'pvm)
				tab `idg'_split
```

```
					gen `idg'_timedep =0
					replace `idg'_timedep = 1 if any`idg'==1 & `idg'_split==0
					tab `idg'_timedep
					tab any`idg' if `idg'_split!=-1

					stdescribe
					quietly: local failures = `r(N_fail)'
					stcox `idg'_timedep i.supu i.ses
					quietly: matrix m = r(table)
					matrix list m
					tempname hr ll ul
					scalar `hr' = m[1,1]
					scalar `ll' = m[5,1]
					scalar `ul' = m[6,1]
					quietly: putexcel set "UKB infection burden time-dep $S_DATE.xlsm", sheet("FIGURE sensitivity
AD") modify
						quietly: putexcel K`irow'=(`e(N_sub)'), nformat(number)
						quietly: putexcel L`irow'=(`e(N_fail)'), nformat(number)
						quietly: putexcel E`irow'=(`hr'), nformat(number_d2)
						quietly: putexcel F`irow'=(`ll'), nformat(number_d2)
						quietly: putexcel G`irow'=(`ul'), nformat(number_d2)
						quietly: putexcel B`irow'=("`idg'")
						quietly: putexcel C`irow'=("UKB")
						quietly: putexcel D`irow'=("`idg_name'")
					tab any`idg', matcell(x), if `idg'_split!=-1
					matrix list x
					tab any`idg' dementiatype, matcell(y), if `idg'_split!=-1 & _d==1
					matrix list y
					quietly: putexcel set "UKB infection burden time-dep $S_DATE.xlsm", sheet("FIGURE sensitivity
AD") modify
						quietly: putexcel M`irow'=(x[2,1]), nformat(number)
						quietly: putexcel N`irow'=(y[2,1]), nformat(number)
					local ++irow
					local ++irow
					restore
					}

*TIME-DEPENDENT: Difference between CNS infections and extra-CNS infections

		*CNS infection vs non cns infection
		capture gen byte anycnsnotcns = 1 if ensianycnspvm==ensianyinfpvm & anyanycns==1 &
ensianyinfpvm<exitpvm
		replace anycnsnotcns = 0 if ensinoncnspvm==ensianyinfpvm & anycnsnotcns!=1 & anynoncns==1 &
ensianyinfpvm<exitpvm
		capture gen ensicnsnotcnspvm = ensianyinfpvm if ensianyinfpvm < exitpvm
		count
		sum ensicnsnotcnspvm
		tab anycnsnotcns
		tab anyanycns
		tab anynoncns
		tab anycnsnotcns dementiatype
		tab anyanycns dementiatype
		tab anynoncns dementiatype


local dg cnsnotcns
local n_dg: word count `dg'
local dg_name `""CNS vs extra-CNS infection""'
```

```
quietly: local irow = 34
		forvalues i=1/`n_dg' {
				preserve
				quietly: local idg : word `i' of `dg'
				quietly: local idg_name : word  `i' of `dg_name'
				display ""
				display "`idg' followed by broad dementia, adjusted for sex and education"
				display ""
				display "Pooled IPD-analysis"
				display ""
				display "$S_TIME  $S_DATE"
				display ""

				stset exitpvm, id(id) failure(dementiatype==1) origin(time syntpvm) enter(time entrypvm)
scale(365.25)

				gen byte anyeither = ensi`idg'pvm < exitpvm
				tab any`idg'
				tab anyeither

				stsplit `idg'_split, at(0) after(time=ensi`idg'pvm)
				tab `idg'_split
				gen `idg'_timedep =0
				replace `idg'_timedep = 1 if anyeither==1 & `idg'_split==0
				tab `idg'_timedep
				tab anyeither if `idg'_split!=-1

				stdescribe
				quietly: local failures = `r(N_fail)'

				tab anyeither if `idg'_split!=-1
				tab `idg'_timedep
				recode `idg'_timedep 1=2 if any`idg'==0
				tab `idg'_timedep
				stcox i.`idg'_timedep supu ses
				test 1.`idg'_timedep = 2.`idg'_timedep

				quietly: putexcel set "UKB infection burden time-dep $S_DATE.xlsm", sheet("FIGURE sensitivity
AD") modify
						quietly: putexcel K`irow'=(`e(N_sub)'), nformat(number)
						quietly: putexcel L`irow'=(`e(N_fail)'), nformat(number)
						quietly: putexcel E`irow'=(`r(p)'), nformat(number_d2)
						quietly: putexcel B`irow'=("`idg'")
						quietly: putexcel C`irow'=("UKB")
						quietly: putexcel D`irow'=("`idg_name'")
				local ++irow

				local ++irow
				restore
				}
```

**** TIME-DEPENDENT: Analysis VASCULAR DEMENTIA *****

*****TIME-DEPENDENT: SINGLE VS MULTIPLE INFECTIONS *******

```
count
tab toinenanyinf, mis
tab kolmasanyinf, mis

tab anyanyinf if ensianyinfpvm<exitpvm & ensianyinfpvm < ensitoinenanyinfpvm
tab toinenanyinf if ensitoinenanyinfpvm<exitpvm & ensitoinenanyinfpvm < ensikolmasanyinfpvm
tab kolmasanyinf if ensikolmasanyinfpvm<exitpvm

tab anyanyinf if ensianyinfpvm<exitpvm & ensianyinfpvm < ensitoinenanyinfpvm & ensitoinenanyinfpvm>=exitpvm
tab toinenanyinf if ensitoinenanyinfpvm<exitpvm & ensitoinenanyinfpvm < ensikolmasanyinfpvm  &
ensikolmasanyinfpvm>=exitpvm
tab kolmasanyinf if ensikolmasanyinfpvm<exitpvm

tab dementiatype if ensianyinfpvm<exitpvm & ensitoinenanyinfpvm>=exitpvm
tab dementiatype if ensitoinenanyinfpvm<exitpvm & ensikolmasanyinfpvm>=exitpvm
tab dementiatype if ensikolmasanyinfpvm<exitpvm

sum ensianyinfpvm
replace ensianyinfpvm = . if ensianyinfpvm>=exitpvm
sum ensianyinfpvm

sum ensitoinenanyinfpvm
replace ensitoinenanyinfpvm = . if ensitoinenanyinfpvm>=exitpvm
sum ensitoinenanyinfpvm

sum ensikolmasanyinfpvm
replace ensikolmasanyinfpvm = . if ensikolmasanyinfpvm>=exitpvm
sum ensikolmasanyinfpvm

replace toinenanyinf = . if ensitoinenanyinfpvm==.
replace kolmasanyinf = . if ensikolmasanyinfpvm==.

local dg_name `""Multiple infections""'
local irow 16
local prow = `irow'
				preserve
				stset exitpvm, id(id) failure(dementiatype==4) origin(time syntpvm) enter(time entrypvm)
scale(365.25)

				stsplit first_split, at(0) after(time=ensianyinfpvm)
				gen multiinf_timedep =0
				tab multiinf_timedep
				replace multiinf_timedep = 1 if anyanyinf==1 & first_split==0
				tab multiinf_timedep
				stsplit second_split, at(0) after(time=ensitoinenanyinfpvm)
```

```
replace multiinf_timedep = 2 if toinenanyinf==1 & second_split==0
tab multiinf_timedep
stsplit third_split, at(0) after(time=ensikolmasanyinfpvm)
replace multiinf_timedep = 3 if kolmasanyinf==1 & third_split==0
tab multiinf_timedep

stdescribe
quietly: local failures = `r(N_fail)'
stcox i.multiinf_timedep i.supu i.ses
quietly: matrix m = r(table)
matrix list m
tempname hr1 ll1 ul1 hr2 ll2 ul2 hr3 ll3 ul3
scalar `hr1' = m[1,2]
scalar `ll1' = m[5,2]
scalar `ul1' = m[6,2]
scalar `hr2' = m[1,3]
scalar `ll2' = m[5,3]
scalar `ul2' = m[6,3]
scalar `hr3' = m[1,4]
scalar `ll3' = m[5,4]
scalar `ul3' = m[6,4]

forvalues i =1/3 {
quietly: putexcel set "UKB infection burden time-dep $S_DATE.xlsm", sheet("FIGURE sensitivity
VaD") modify
        quietly: putexcel K`irow'=(`e(N_sub)'), nformat(number)
        quietly: putexcel L`irow'=(`e(N_fail)'), nformat(number)
        quietly: putexcel E`irow'=(`hr`i''), nformat(number_d2)
        quietly: putexcel F`irow'=(`ll`i''), nformat(number_d2)
        quietly: putexcel G`irow'=(`ul`i''), nformat(number_d2)
        quietly: putexcel B`irow'=("Multiinf")
        quietly: putexcel C`irow'=("UKB")
        quietly: putexcel D`irow'=("Single vs multiple infections, infection No. `i'")
tab multiinf_timedep, matcell(x), if first_split!=-1 & second_split!=-1 & third_split!=-1
matrix list x
tab multiinf_timedep dementiatype, matcell(y), if first_split!=-1 & second_split!=-1 &
third_split!=-1 &_d==1
matrix list y
quietly: putexcel set "UKB infection burden time-dep $S_DATE.xlsm", sheet("FIGURE sensitivity
VaD") modify
        local j = `i' + 1
        quietly: putexcel M`irow'=(x[`j',1]), nformat(number)
        quietly: putexcel N`irow'=(y[`j',1]), nformat(number)

local ++irow
local ++irow
}

*P for trend
stcox multiinf_timedep i.supu i.ses
quietly: matrix n = r(table)
matrix list n
tempname p
scalar `p' = n[4,1]
quietly: putexcel set "UKB infection burden time-dep $S_DATE.xlsm", sheet("FIGURE sensitivity
VaD") modify
        quietly: putexcel O`prow'=(`p'), nformat(number)
```

```
*Dementia occurring from year 5 onwards
di "Dementia occurring from year 5 onwards"
local i 1
foreach idg in anyinf toinenanyinf kolmasanyinf {
display `i'
replace _t0 = _t0 + 5 if multiinf_timedep==0 & `i'==1
drop if _t0>=_t
gen ensi`idg'pvm5v = ensi`idg'pvm + round(5*365.25) if ensi`idg'pvm<entrypvm
replace ensi`idg'pvm5v = entrypvm if ensi`idg'pvm5v<entrypvm
replace _t0 = (ensi`idg'pvm5v-syntpvm)/365.25 if ensi`idg'pvm5v!=. & multiinf_timedep==`i'
replace _t0 = _t0 + 5 if ensi`idg'pvm5v==. & multiinf_timedep==`i'
drop if _t0>=_t
local ++i
}

stdescribe
quietly: local failures = `r(N_fail)'
stcox i.multiinf_timedep 2.supu#cohort 2.ses#cohort 3.ses#cohort, strata(cohort)
quietly: matrix m = r(table)
matrix list m
tempname hr1 ll1 ul1 hr2 ll2 ul2 hr3 ll3 ul3
scalar `hr1' = m[1,2]
scalar `ll1' = m[5,2]
scalar `ul1' = m[6,2]
scalar `hr2' = m[1,3]
scalar `ll2' = m[5,3]
scalar `ul2' = m[6,3]
scalar `hr3' = m[1,4]
scalar `ll3' = m[5,4]
scalar `ul3' = m[6,4]

local irow 17
forvalues i =1/3 {
quietly: putexcel set "UKB infection burden time-dep $S_DATE.xlsm", sheet("FIGURE sensitivity
VaD") modify
        quietly: putexcel K`irow'=(`e(N_sub)'), nformat(number)
        quietly: putexcel L`irow'=(`e(N_fail)'), nformat(number)
        quietly: putexcel E`irow'=(`hr`i''), nformat(number_d2)
        quietly: putexcel F`irow'=(`ll`i''), nformat(number_d2)
        quietly: putexcel G`irow'=(`ul`i''), nformat(number_d2)
        quietly: putexcel B`irow'=("Multiinf")
        quietly: putexcel C`irow'=("UKB")
        quietly: putexcel D`irow'=("Dementia occurring more than 5 years after the
hospitalisation, infection No. `i'")
tab multiinf_timedep, matcell(x), if first_split!=-1 & second_split!=-1 & third_split!=-1
matrix list x
tab multiinf_timedep dementiatype, matcell(y), if first_split!=-1 & second_split!=-1 &
third_split!=-1 &_d==1
matrix list y
quietly: putexcel set "UKB infection burden time-dep $S_DATE.xlsm", sheet("FIGURE sensitivity
VaD") modify
        local j = `i' + 1
        quietly: putexcel M`irow'=(x[`j',1]), nformat(number)
        quietly: putexcel N`irow'=(y[`j',1]), nformat(number)
local ++irow
local ++irow
}
```

```
*P for trend
local ++prow
stcox multiinf_timedep i.supu i.ses
quietly: matrix n = r(table)
matrix list n
tempname p
scalar `p' = n[4,1]
quietly: putexcel set "UKB infection burden time-dep $S_DATE.xlsm", sheet("FIGURE sensitivity VaD") modify
        quietly: putexcel O`prow'=(`p'), nformat(number)
restore
```

*****TIME-DEPENDENT: One or several different pathogens *******

```
local dg_name `""Multiple organisms""'
local idg_name `""Multiple_organisms""'
local irow 37
local prow = `irow'

preserve
drop if ses==.
egen ensibactviralpvm = rowmin(ensibactinfpvm ensiviralinfpvm)
gen toinenbactviralpvm = ensibactviralpvm if ensibactinfpvm==ensiviralinfpvm & ensibactinfpvm!=.
replace toinenbactviralpvm = ensibactinfpvm if ensibactinfpvm>ensiviralinfpvm & ensibactinfpvm!=.
replace toinenbactviralpvm = ensiviralinfpvm if ensiviralinfpvm>ensibactinfpvm & ensiviralinfpvm!=.
gen anybactviral = 1 if ensibactviralpvm!=.
gen toinenbactviral = 1 if toinenbactviralpvm!=.
tab anybactviral
tab anybactviral if toinenbactviral!=1
tab toinenbactviral
tab anybactviral dementiatype
tab anybactviral dementiatype if toinenbactviral!=1
tab toinenbactviral dementiatype

quietly: stset exitpvm, id(id) failure(dementiatype==4) origin(time syntpvm) enter(time entrypvm) scale(365.25)
quietly: keep id anybactviral supu cohort exitpvm ensidementia syntpvm entrypvm ensi*pvm *toinen* _st _d _origin _t _t0 ses
sum ensibactviralpvm toinenbactviralpvm
tab anybactviral
tab toinenbactviral

stsplit first_split, at(0) after(time=ensibactviralpvm)
gen multiinf_timedep =0
tab multiinf_timedep
replace multiinf_timedep = 1 if anybactviral==1 & first_split==0
tab multiinf_timedep
stsplit second_split, at(0) after(time=toinenbactviralpvm)
replace multiinf_timedep = 2 if toinenbactviral==1 & second_split==0
tab multiinf_timedep

quietly: stdescribe
quietly: local failures = `r(N_fail)'
stcox i.multiinf_timedep 2.supu#cohort 2.ses#cohort 3.ses#cohort, strata(cohort)
```

```
				quietly: matrix m = r(table)
				matrix list m
				tempname hr1 ll1 ul1 hr2 ll2 ul2
				scalar `hr1' = m[1,2]
				scalar `ll1' = m[5,2]
				scalar `ul1' = m[6,2]
				scalar `hr2' = m[1,3]
				scalar `ll2' = m[5,3]
				scalar `ul2' = m[6,3]

				forvalues i =1/2 {
				quietly: putexcel set "UKB infection burden time-dep $S_DATE.xlsm", sheet("FIGURE sensitivity
VaD") modify
						quietly: putexcel K`irow'=(`e(N_sub)'), nformat(number)
						quietly: putexcel L`irow'=(`e(N_fail)'), nformat(number)
						quietly: putexcel E`irow'=(`hr`i''), nformat(number_d2)
						quietly: putexcel F`irow'=(`ll`i''), nformat(number_d2)
						quietly: putexcel G`irow'=(`ul`i''), nformat(number_d2)
						quietly: putexcel B`irow'=("`idg_name'")
						quietly: putexcel C`irow'=("UKB")
						quietly: putexcel D`irow'=("Different micro-organisms, infection No. `i'")
				tab multiinf_timedep, matcell(x), if first_split!=-1 & second_split!=-1
				matrix list x
				tab multiinf_timedep ensidementia, matcell(y), if first_split!=-1 & second_split!=-1 &_d==1
				matrix list y
				quietly: putexcel set "UKB infection burden time-dep $S_DATE.xlsm", sheet("FIGURE sensitivity
VaD") modify
						local j = `i' + 1
						quietly: putexcel M`irow'=(x[`j',1]), nformat(number)
						quietly: putexcel N`irow'=(y[`j',1]), nformat(number)
				local ++irow
				local ++irow
				}
				*P for trend
				stcox multiinf_timedep 2.supu#cohort 2.ses#cohort 3.ses#cohort, strata(cohort)
				quietly: matrix n = r(table)
				matrix list n
				tempname p
				scalar `p' = n[4,1]
				quietly: putexcel set "UKB infection burden time-dep $S_DATE.xlsm", sheet("FIGURE sensitivity
VaD") modify
						quietly: putexcel Q`prow'=(`p'), nformat(number)


				restore


*****TIME-DEPENDENT:  NUMBER OF SIMULTANEOUS INFECTIONS *******

count
tab anyinfcount, mis
tab anyinfcount dementiatype, mis
replace anyinfcount = . if ensianyinfpvm>=exitpvm
tab anyinfcount, mis
tab anyinfcount dementiatype, mis

local dg infcount
local n_dg: word count `dg'
```

```
local dg_name `""No. of simultaneous infections""'

quietly: local irow = 23
local prow = `irow'
		forvalues i=1/`n_dg' {
				preserve
				quietly: local idg : word `i' of `dg'
				quietly: local idg_name : word  `i' of `dg_name'
				display ""
				display "`idg' followed by broad dementia, adjusted for sex and education"
				display ""
				display "Pooled IPD-analysis, normal model"
				display ""
				display "$S_TIME  $S_DATE"
				display ""

				quietly: stset exitpvm, id(id) failure(dementiatype==4) origin(time syntpvm) enter(time
entrypvm) scale(365.25)
				tab any`idg'
				replace any`idg' = 2 if any`idg'>2 & any`idg'<.
				tab any`idg'
				stsplit `idg'_split, at(0) after(time=ensi`idg'pvm)
				tab `idg'_split
				gen `idg'_timedep =0
				replace `idg'_timedep = any`idg' if inlist(any`idg',1,2) & `idg'_split==0
				tab `idg'_timedep
				tab any`idg' if `idg'_split!=-1

				stdescribe
				quietly: local failures = `r(N_fail)'
				stcox i.`idg'_timedep i.supu i.ses
				quietly: matrix m = r(table)
				matrix list m
				tempname hr1 ll1 ul1 hr2 ll2 ul2 hr3 ll3 ul3
				scalar `hr1' = m[1,2]
				scalar `ll1' = m[5,2]
				scalar `ul1' = m[6,2]
				scalar `hr2' = m[1,3]
				scalar `ll2' = m[5,3]
				scalar `ul2' = m[6,3]
				scalar `hr3' = m[1,4]
				scalar `ll3' = m[5,4]
				scalar `ul3' = m[6,4]

				forvalues i =1/2 {
				quietly: putexcel set "UKB infection burden time-dep $S_DATE.xlsm", sheet("FIGURE sensitivity
VaD") modify
					quietly: putexcel K`irow'=(`e(N_sub)'), nformat(number)
					quietly: putexcel L`irow'=(`e(N_fail)'), nformat(number)
					quietly: putexcel E`irow'=(`hr`i''), nformat(number_d2)
					quietly: putexcel F`irow'=(`ll`i''), nformat(number_d2)
					quietly: putexcel G`irow'=(`ul`i''), nformat(number_d2)
					quietly: putexcel B`irow'=("Simultaneous infection diagnoses")
					quietly: putexcel C`irow'=("UKB")
					quietly: putexcel D`irow'=("No. of simultaneous infection diagnoses = `i'")
				tab `idg'_timedep, matcell(x), if `idg'_split!=-1
				matrix list x
				tab `idg'_timedep dementiatype, matcell(y), if `idg'_split!=-1 & _d==1
```

```
					matrix list y
					quietly: putexcel set "UKB infection burden time-dep $S_DATE.xlsm", sheet("FIGURE sensitivity 
VaD") modify
							local j = `i' + 1
							quietly: putexcel M`irow'=(x[`j',1]), nformat(number)
							quietly: putexcel N`irow'=(y[`j',1]), nformat(number)
					local ++irow
					local ++irow
					}

					*P for trend
					stcox `idg'_timedep i.supu i.ses
					quietly: matrix n = r(table)
					matrix list n
					tempname p
					scalar `p' = n[4,1]
					quietly: putexcel set "UKB infection burden time-dep $S_DATE.xlsm", sheet("FIGURE sensitivity 
VaD") modify
							quietly: putexcel O`prow'=(`p'), nformat(number)

					restore
					}


*TIME-DEPENDENT: FIGURE sensitivity, CNS vs other infections
count
tab anyanycns
recode anyanycns .=0
tab anyanycns
tab anynoncns
recode anynoncns .=0
tab anynoncns
replace anyanycns=0 if ensianycnspvm>=exitpvm
replace anynoncns=0 if ensinoncnspvm>=exitpvm
replace ensianycnspvm=. if ensianycnspvm>=exitpvm
replace ensinoncnspvm=. if ensinoncnspvm>=exitpvm
tab anyanycns
tab anynoncns
tab anyanycns dementiatype
tab anynoncns dementiatype


local dg anycns noncns
local n_dg: word count `dg'
local dg_name `""Any CNS infection" "Any extra-CNS infection""'
quietly: local irow = 28
			forvalues i=1/`n_dg' {
					preserve
					quietly: local idg : word `i' of `dg'
					quietly: local idg_name : word  `i' of `dg_name'
					display ""
					display "`idg' followed by broad dementia, adjusted for sex and education"
					display ""
					display "UKB replication analysis, normal model and 5-year exclusion"
					display ""
					display "$S_TIME  $S_DATE"
					display ""
```

```
                    stset exitpvm, id(id) failure(dementiatype==4) origin(time syntpvm) enter(time entrypvm)
scale(365.25)
                    tab any`idg'
                    stsplit `idg'_split, at(0) after(time=ensi`idg'pvm)
                    tab `idg'_split
                    gen `idg'_timedep =0
                    replace `idg'_timedep = 1 if any`idg'==1 & `idg'_split==0
                    tab `idg'_timedep
                    tab any`idg' if `idg'_split!=-1

                    stdescribe
                    quietly: local failures = `r(N_fail)'
                    stcox `idg'_timedep i.supu i.ses
                    quietly: matrix m = r(table)
                    matrix list m
                    tempname hr ll ul
                    scalar `hr' = m[1,1]
                    scalar `ll' = m[5,1]
                    scalar `ul' = m[6,1]
                    quietly: putexcel set "UKB infection burden time-dep $S_DATE.xlsm", sheet("FIGURE sensitivity
VaD") modify
                            quietly: putexcel K`irow'=(`e(N_sub)'), nformat(number)
                            quietly: putexcel L`irow'=(`e(N_fail)'), nformat(number)
                            quietly: putexcel E`irow'=(`hr'), nformat(number_d2)
                            quietly: putexcel F`irow'=(`ll'), nformat(number_d2)
                            quietly: putexcel G`irow'=(`ul'), nformat(number_d2)
                            quietly: putexcel B`irow'=("`idg'")
                            quietly: putexcel C`irow'=("UKB")
                            quietly: putexcel D`irow'=("`idg_name'")
                    tab any`idg', matcell(x), if `idg'_split!=-1
                    matrix list x
                    tab any`idg' dementiatype, matcell(y), if `idg'_split!=-1 & _d==1
                    matrix list y
                    quietly: putexcel set "UKB infection burden time-dep $S_DATE.xlsm", sheet("FIGURE sensitivity
VaD") modify
                            quietly: putexcel M`irow'=(x[2,1]), nformat(number)
                            quietly: putexcel N`irow'=(y[2,1]), nformat(number)
                    local ++irow
                    local ++irow
                    restore
                    }

*TIME-DEPENDENT: Difference between CNS infections and extra-CNS infections

            *CNS infection vs non cns infection
            capture gen byte anycnsnotcns = 1 if ensianycnspvm==ensianyinfpvm & anyanycns==1 &
ensianyinfpvm<exitpvm
            replace anycnsnotcns = 0 if ensinoncnspvm==ensianyinfpvm & anycnsnotcns!=1 & anynoncns==1 &
ensianyinfpvm<exitpvm
            capture gen ensicnsnotcnspvm = ensianyinfpvm if ensianyinfpvm < exitpvm
            count
            sum ensicnsnotcnspvm
            tab anycnsnotcns
            tab anyanycns
            tab anynoncns
            tab anycnsnotcns dementiatype
            tab anyanycns dementiatype
            tab anynoncns dementiatype
```

```
local dg cnsnotcns
local n_dg: word count `dg'
local dg_name `""CNS vs extra-CNS infection""'
quietly: local irow = 34
			forvalues i=1/`n_dg' {
					preserve
					quietly: local idg : word `i' of `dg'
					quietly: local idg_name : word  `i' of `dg_name'
					display ""
					display "`idg' followed by broad dementia, adjusted for sex and education"
					display ""
					display "Pooled IPD-analysis"
					display ""
					display "$S_TIME  $S_DATE"
					display ""

					stset exitpvm, id(id) failure(dementiatype==4) origin(time syntpvm) enter(time entrypvm)
scale(365.25)

					gen byte anyeither = ensi`idg'pvm < exitpvm
					tab any`idg'
					tab anyeither

					stsplit `idg'_split, at(0) after(time=ensi`idg'pvm)
					tab `idg'_split
					gen `idg'_timedep =0
					replace `idg'_timedep = 1 if anyeither==1 & `idg'_split==0
					tab `idg'_timedep
					tab anyeither if `idg'_split!=-1

					stdescribe
					quietly: local failures = `r(N_fail)'

					tab anyeither if `idg'_split!=-1
					tab `idg'_timedep
					recode `idg'_timedep 1=2 if any`idg'==0
					tab `idg'_timedep
					stcox i.`idg'_timedep supu ses
					test 1.`idg'_timedep = 2.`idg'_timedep

					quietly: putexcel set "UKB infection burden time-dep $S_DATE.xlsm", sheet("FIGURE sensitivity
VaD") modify
							quietly: putexcel K`irow'=(`e(N_sub)'), nformat(number)
							quietly: putexcel L`irow'=(`e(N_fail)'), nformat(number)
							quietly: putexcel E`irow'=(`r(p)'), nformat(number_d2)
							quietly: putexcel B`irow'=("`idg'")
							quietly: putexcel C`irow'=("UKB")
							quietly: putexcel D`irow'=("`idg_name'")
					local ++irow

					restore
					}


log close
```
